# The association between dengue incidences and provincial-level weather variables in Thailand from 2001 to 2014

**DOI:** 10.1371/journal.pone.0226945

**Published:** 2019-12-26

**Authors:** Romrawin Chumpu, Nirattaya Khamsemanan, Cholwich Nattee

**Affiliations:** Sirindhorn International Institute of Technology, Thammasat University, Pathum Thani, Thailand; Faculty of Science, Ain Shams University (ASU), EGYPT

## Abstract

Dengue and dengue hemorrhagic pose significant burdens in many tropical countries. Dengue incidences have perpetually increased, leading to an annual (uncertain) peak. Dengue cases cause an enormous public health problem in Thailand because there is no anti-viral drug against the dengue virus. Searching for means to reduce the dengue incidences is a challenging and appropriate strategy for primary prevention in a dengue outbreak. This study constructs the best predictive model from past statistical dengue incidences at the provincial level and studies the relationships among dengue incidences and weather variables. We conducted experiments for 65 provinces (out of 77 provinces) in Thailand since there is no dengue information for the remaining provinces. Predictive models were constructed using weekly data during 2001-2014. The training set are data during 2001-2013, and the test set is the data from 2014. Collected data were separated into two parts: current dengue cases as the dependent variable, and weather variables and previous dengue cases as the independent variables. Eight weather variables are used in our models: average pressure, maximum temperature, minimum temperature, average humidity, precipitation, vaporization, wind direction, wind power. Each weather variable includes the current week and one to three weeks of lag time. A total of 32 independent weather variables are used for each province. The previous one to three weeks of dengue cases are also used as independent variables. There is a total of 35 independent variables. Predictive models were constructed using five methods: Poisson regression, negative binomial regression, quasi-likelihood regression, ARIMA(3,1,4) and SARIMA(2,0,1)(0,2,0). The best model is determined by combinations of 1–12 variables, which are 232,989,800 models for each province. We construct a total of 15,144,337,000 models. The best model is selected by the average from high to low of the coefficient of determination (R2) and the lowest root mean square error (RMSE). From our results, the one-week lag previous case variable is the most frequent in 55 provinces out of a total of 65 provinces (coefficient of determinations with a minimum of 0.257 and a maximum of 0.954, average of 0.6383, 95% CI: 0.57313 to 0.70355). The most influential weather variable is precipitation, which is used in most of the provinces, followed by wind direction, wind power, and barometric pressure. The results confirm the common knowledge that dengue incidences occur most often during the rainy season. It also shows that wind direction, wind power, and barometric pressure also have influences on the number of dengue cases. These three weather variables may help adult mosquitos to survive longer and spread dengue. In conclusion, The most influential factor for further cases is the number of dengue cases. However, weather variables are also needed to obtain better results. Predictions of the number of dengue cases should be done locally, not at the national level. The best models of different provinces use different sets of weather variables. Our model has an accuracy that is sufficient for the real prediction of future dengue incidences, to prepare for and protect against severe dengue outbreaks.

## Introduction

### Dengue disease

Dengue is a mosquito-borne disease, in which Aedes aegypti is the main vector. This type of mosquito is commonly found in tropical countries. Dengue fever (DF) and dengue hemorrhagic fever (DHF), a severe form of the disease, are caused by four dengue serotypes represented by DEN 1, 2, 3, and 4 [1–4]. Reinfections of different serotypes can cause severe illnesses or deaths.

Once infected, it can take 3–14 days for the virus to incubate. Dengue fever is the initial stage of dengue cases. Symptoms of dengue fever include a high fever, body and muscular aches, nausea, vomiting, skin rash, and fatigue. Dengue fever can last 5 to 7 days. Some patients may recover. However, the disease may develop into the next lethal stage called dengue hemorrhagic fever. Clinical manifestations of DHF include reduced blood pressure due to plasma leakage from capillaries and bleeding due to low platelet counts and impairment of platelet functions. If their blood pressure is extremely low, patients may enter the stage of dengue shock syndrome, lose consciousness, and pass into the last stage of shock.

The World Health Organization (WHO) reports that during the last decade, there were approximately 390 million dengue incidences per year (284–528 million, 95% CI). About 25% of those incidences, or about 96 million people, had serious symptoms [5]. Approximately 10,000 to 20,000 people die from dengue disease each year. Almost 75 percent of the global dengue cases are in Southeast Asia and the Western Pacific Region [4]. In Southeast Asia, all four serotypes of dengue virus have been reported in the Philippines, Thailand, Malaysia, Vietnam, and eastern India [6]. Previous studies proposed that host factors play an important role in the pathogenesis of severe manifestations of dengue infection [7]. Dengue incidences in Southeast Asia were uncontrolled and predominant in children during 1970–1990s [7–9]. A previous study [10] showed the global burden of dengue. The rate of incidences increased during 1990–2013, and most patients are adult. Unfortunately, there are no cures for dengue. Consequently, dengue is regarded as one of the top three deadly infectious diseases. Prevention is one of the best ways to fight the disease. In this work, we believe that understanding the relationships among the disease and other factors such as weather and the prediction of future dengue infections are appropriate methods for the prevention of dengue.

### Dengue disease in Thailand

**Fig 1:**
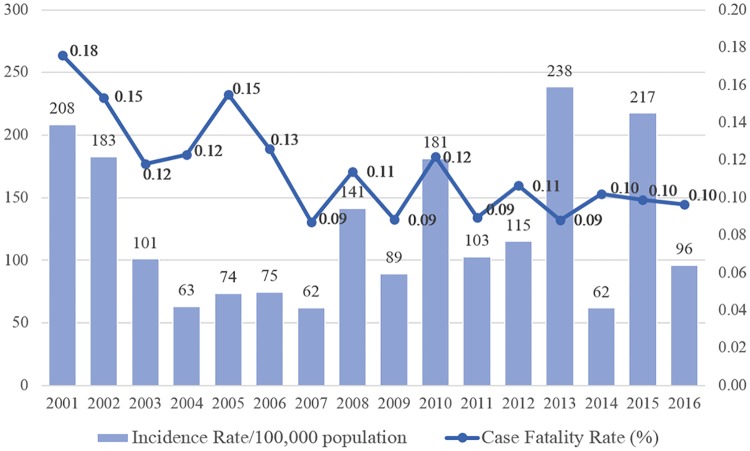
Dengue incidences in Thailand during 2001–2016.

Dengue cases in Thailand have increased continuously over the last 60 years, since the first dengue incidence was recorded in Bangkok in 1954 [7, 11]. The main areas of infections are semi-urban or urban areas where there is abandoned water storage. Dengue and dengue hemorrhagic fever are also the leading cause of hospitalizations and deaths among children in Thailand [8, 9]. Records from the Bureau of Epidemiology of Thailand showed that dengue was endemic as a biannual peak during 1958–1967. After 1973, the trends were indefinable. The highest number of dengue incidences occurred in 2013 with 154,444 cases and 136 deaths (241.03 cases per 100,000 population and the fatality rate was 0.21) (Fig 1). The top three highest months were July, June, and August, during the rainy season in descending order. Most patients are 15-24 years old (29.16%), 10-14 years (21.31%), and 5-9 years (13.74). The top ten highest provinces per 100,000 population are, in descending order, Chiang Rai (1,110.58, fatality rate was 0.75), Mae Hong Son (768.29, fatality rate was 0.41), Chiang Mai (694.47, fatality rate was 0.49), Phuket (639.54, fatality rate was 0.57), Phang Nga (463.65, fatality rate was 0.78), Krabi (456.81, fatality rate was 0.46), Lampang (453.18, fatality rate was 0.26), Loei (440.70, fatality rate was 1.12), Songkhla (412.29, fatality rate was 0.80), and Nakhon Phanom (332.73, fatality rate was 0.28). These ten provinces had case ratios that were higher than the country average in 2013.

Dengue disease is a large economic burden for Thailand. The work by Clark et al. [12] reveals that the financial loss for dengue infections is higher than the average income in Thailand.

### Dengue and weather factors

Dengue viral pathogens typically live in tropical regions with warm temperatures such as India, Africa, Brazil, Saudi Arabia, Malaysia, Sri Lanka, and Asian countries [4, 13]. Many studies show that weather factors have strong associations with dengue incidences. The study by Naish et. al. [14] reports that temperature, rainfall, and relative humidity have potential effects on the transmission of dengue. In addition, the spatial-temporal patterns and social ecology also have an association with the severity of dengue transmission [14, 15]. Weather factors can potentially have delayed effects on the number of dengue cases [16, 17]. Vapor pressure with a change in geographical limits also has an association with dengue transmission [15].

Due to its severe burden and its strong association with weather variables, many works have created models to study the relationships among dengue incidences and weather variations. Many factors such as humidity, rainfall, and pressure are included in the studies. Statistical models such as the negative binomial regression model have been used in previous studies [18, 19]. Non-linear models have also been used in a previous study [20]. Many models also include the lag time to study the delayed effects of weather variables [1, 4, 6, 14, 16–19, 21–30]. The minimum temperature has a high association with dengue incidences in previous studies [21, 22, 25, 28].

This study creates provincial-level models to find the relationships of weather parameters and dengue incidences. These models can also be used to predict dengue pandemics, locally. Forecasting the future dengue trends would aid the public health department in reducing the dengue burden of Thailand.

## Materials and methods

### Geological details of Thailand

Thailand is a country in a tropical region, north of the equator line. The capital city of Thailand is Bangkok. Thailand covers an area of 513,120 square kilometers, located at latitude 15.8700°N (5–21°N) and longitude 100.9925°E (97–106°E). Thailand has various topographies, geographies, and weather variations. Provinces are the primary local government units. Thailand is composed of 77 provinces with an average area of 6,663.90 square kilometers. Nakorn Ratchasima covers the largest area of 20,494 square kilometers, and Samut Songkhram covers the smallest area of 417 square kilometers. Due to the location and coverage area of Thailand, almost all regions of Thailand have a tropical savannah climate. Only the southern region has a tropical monsoon climate [31]. Because of this, the weather within a province does not vary greatly.

**Fig 2:**
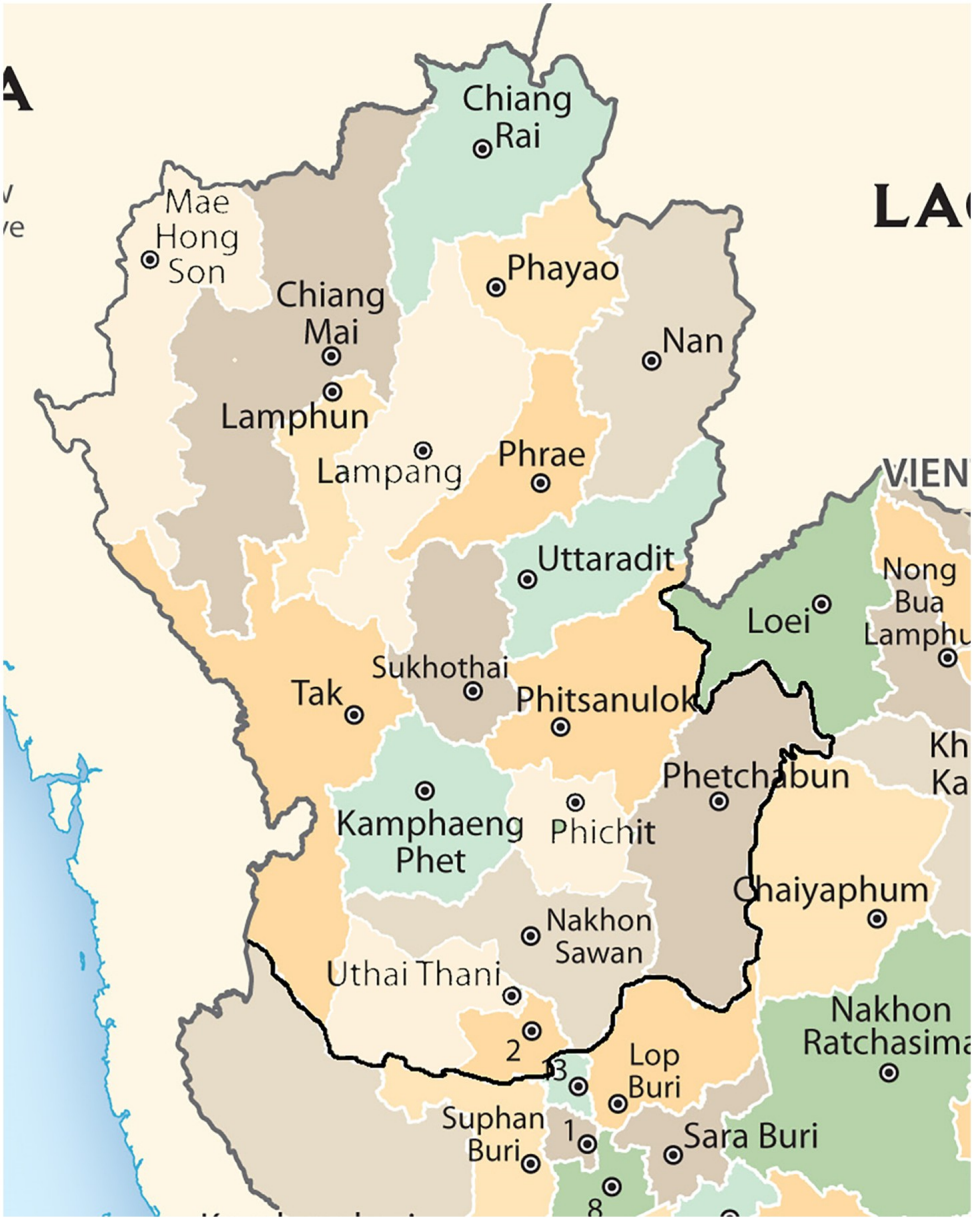
Map showing the northern region of Thailand.

The northern region of Thailand covers an area of 170,000 square kilometers. This region contains high mountains and various types of forests, including flat river basins. This area is separated into 17 provinces: Chiang Mai, Chiang Rai, Nan, Phrae, Payao, Mae Hong Son, Lampang, Lamphun, Uttaradit, Kamphaeng Phet, Tak, Nakhon Sawan, Phichit, Pitsanulok, Phetchabun, Sukhothai, and Uthai Thani. The general climate of the northern region consists of three seasons: summer season (March–May), rainy season (June–September), and winter season (October–February). Because the northern region is mostly on a plateau of 800–1200 square kilometers, the average temperature is approximately 25°C. Rainfall begins in June because of the southwest monsoons. Fig 2 shows a map of the northern region of Thailand.

**Fig 3:**
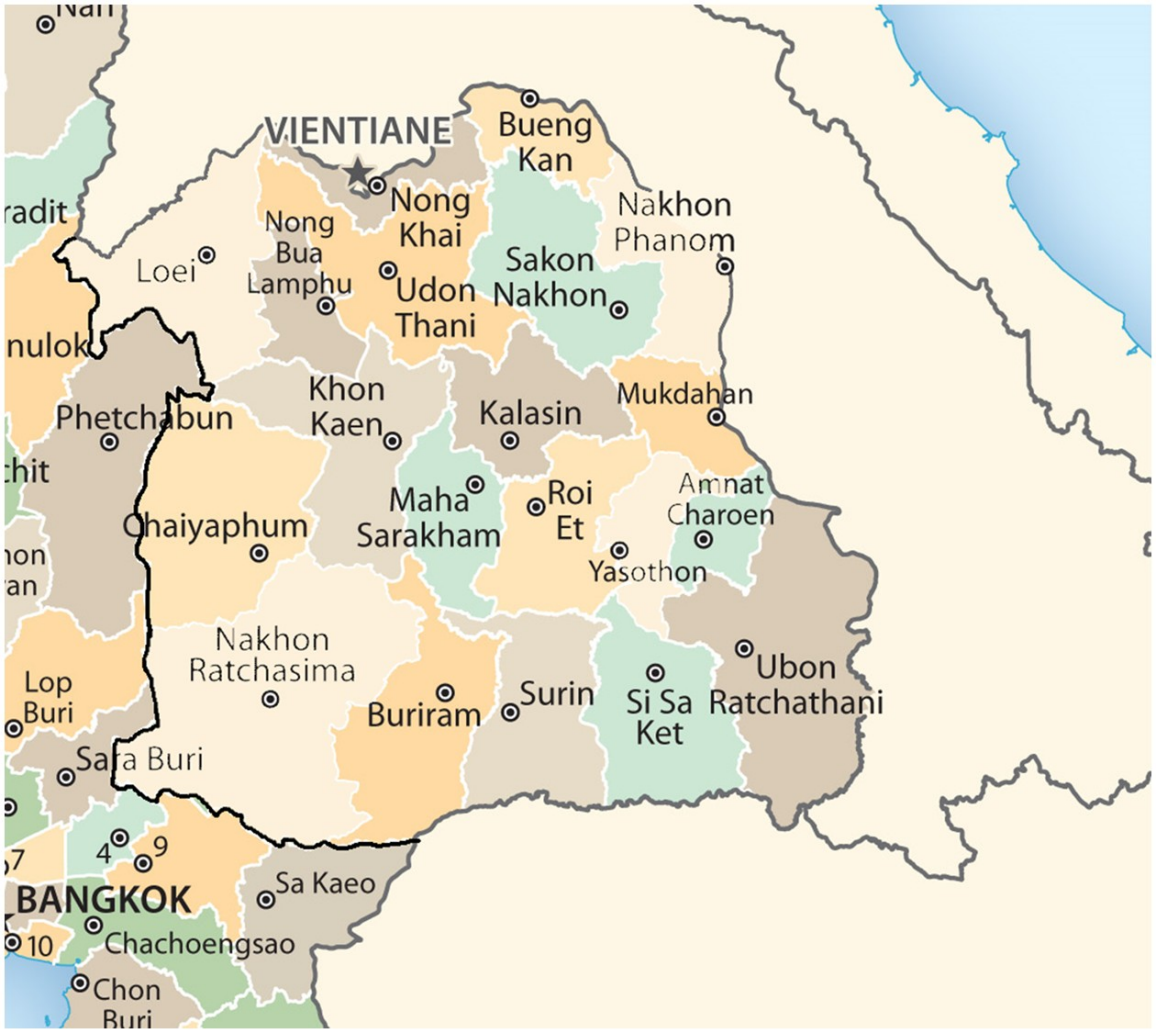
Map showing the northeastern region of Thailand.

The northeastern region of Thailand covers an area of 172,500 square kilometers. The area consists of 20 provinces: Kalasin, Khon Kaen, Chaiyaphum, Nakhon Phanom, Nakhon Ratchasima, Bueng Kan, Buri Ram, Maha Sarakham, Mukdahan, Yasothon, Roi Et, Loei, Si Sa Ket, Sakon Nakhon, Surin, Nong Khai, Nong Bua Lam Phu, Amnat Charoen, Udon Thani, and Ubon Ratchathani. Most areas are on the Khorat plateau. The climate seasons are similar to those of the northern region except that the northeast monsoons begin in November. The average temperature is slightly higher than in the northern region with slightly less rainfall. Fig 3 shows a map of the north-eastern region of Thailand.

**Fig 4:**
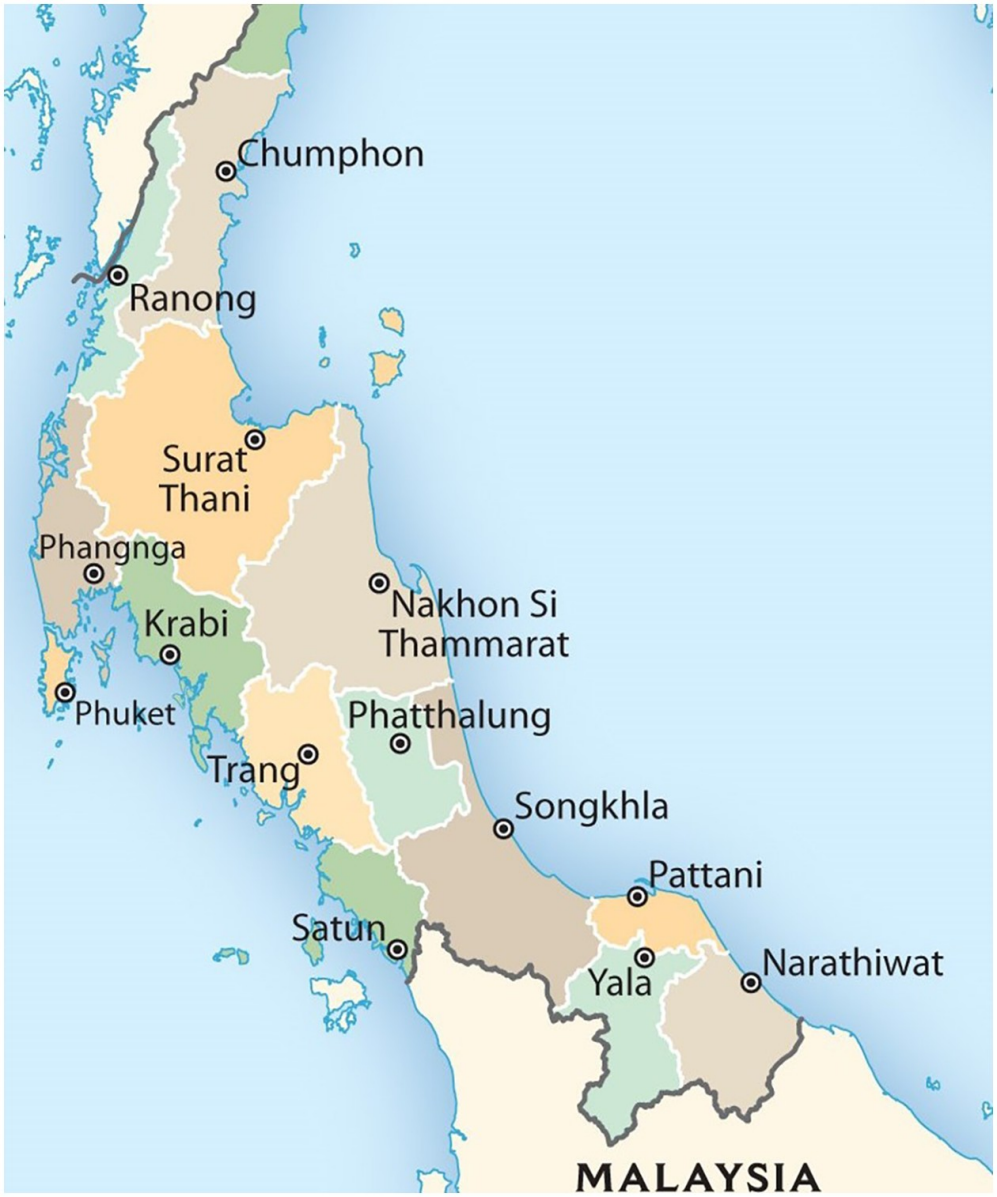
Map showing the southern region of Thailand.

The southern part of Thailand includes an area of 70,715 square kilometers including 14 provinces: Chumphon, Krabi, Trang, Nakhon Si Thammarat, Narathiwat, Pattani, Phang Nga, Phattalung, Phuket, Yala, Ranong, Songkhla, Satun, and Surat Thani. The population density is approximately 130 people per square kilometer. The southern region of Thailand is located between the Andaman Sea and the Gulf of Thailand. The climate of the southern region follows the tropical monsoons. The highest temperature is typically 39.7°C in Trang and the lowest temperature is 12.1°C in Chumphon. The rainy season is longer in the southern region than the other regions, and the southern region rarely has winter-like weather. The average temperature and average rainfall are the highest among all regions in Thailand. Fig 4 shows a map of the southern region of Thailand.

**Fig 5:**
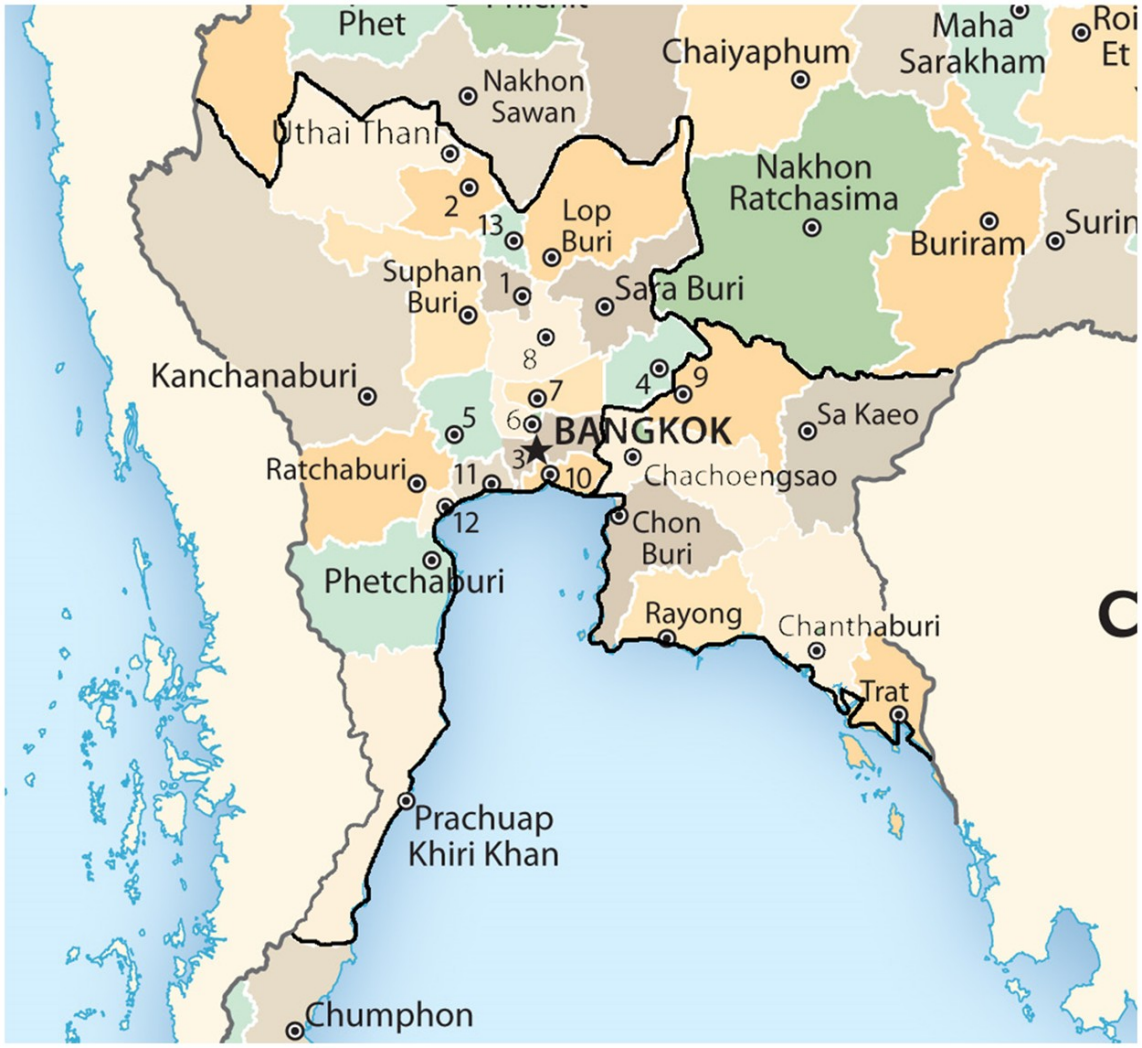
Map showing the central and eastern regions of Thailand.

The central and eastern areas of Thailand consist of 26 provinces: Bangkok, Kanchanaburi, Chanthaburi, Chachoengsao, Chon Buri, Chai Nat, Trat, Nakhon Nayok, Nakhon Pathom, Nonthaburi, Pathum Thani, Prachuap Khiri Khan, Prachin Buri, Phra Nakhon Sri Ayutthaya, Phetchaburi, Rayong, Ratchaburi, Lop Buri, Samut Prakan, Samut Songkhram, Samut Sakhon, Sa Keao, Saraburi, Sing Buri, Suphan Buri, and Ang Thong. The central area has lowland plains where agriculture and industrial stations are located. The climate and seasonal weather are like the northern and northeastern areas, except for two different monsoons in May and November. The average temperature is higher than the northern region but lower than the southern region. The central region of Thailand has a high population density, compared to the other regions. Fig 5 shows a map of the central and eastern regions of Thailand.

### Data

#### Dengue data

The dengue incidence data used in this study come from the Bureau of Epidemiology (BOE), Ministry of Public Health (MOPH), Thailand. The data is in weekly periods from the first week of January 2001 to the last week of December 2014. They are clinically suspected cases reported to the MOPH by the National Disease Surveillance Report 506. Since the highest number of dengue incidences is from 2013 (see Fig 1), dengue data from 2001 to 2013 are used as a training set, whereas 2014 data are used as a test set in this study.

**Data availability:** The dengue incidence data can be obtained through the BOE National Disease Surveillance website.

#### Weather data

**Fig 6:**
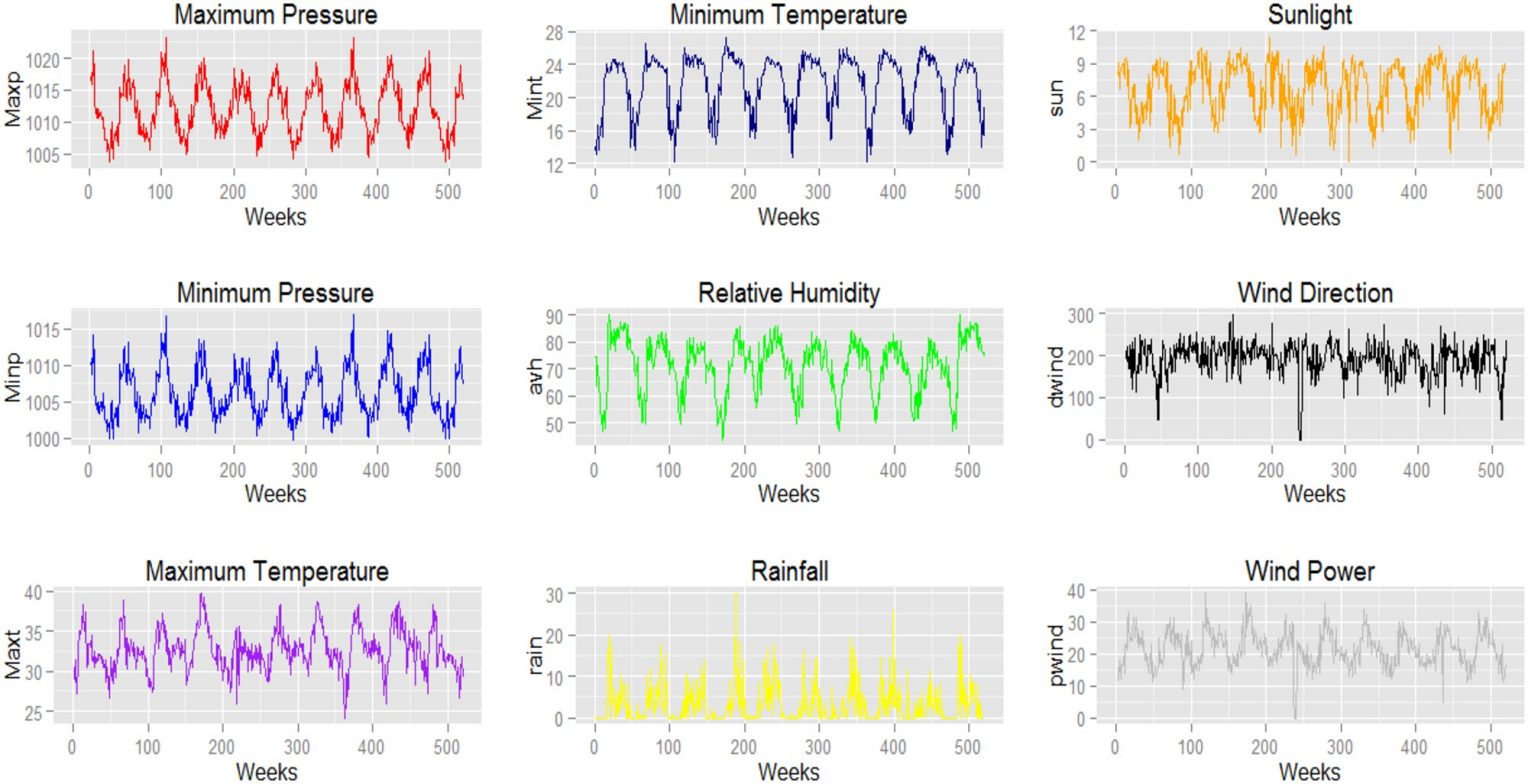
Time series plot of weekly maximum temperature, minimum temperature, precipitation, minimum pressure, relative humidity and wind direction in Chiang Mai, Thailand during 2001-2014.

The weekly provincial-level weather data that was used in this study are from the Thai Meteorological Department. Eight weather parameters are considered in each model: average temperature (*avp*), maximum temperature (*maxt*), minimum temperature (*mint*), average humidity (*avh*), precipitation (*rain*), vaporization (*vapor*), wind direction (*dwind*), and wind power (*pwind*). Fig 6 shows plots of weekly weather data for Chiang Mai from January 2001 to December 2014.

**Data availability:** The weather data can be obtained (with a fee) at the Thai Meteorological Department, 4353 Sukhumvit Road, Bangna, Bangkok, Thailand.

**Fig 7:**
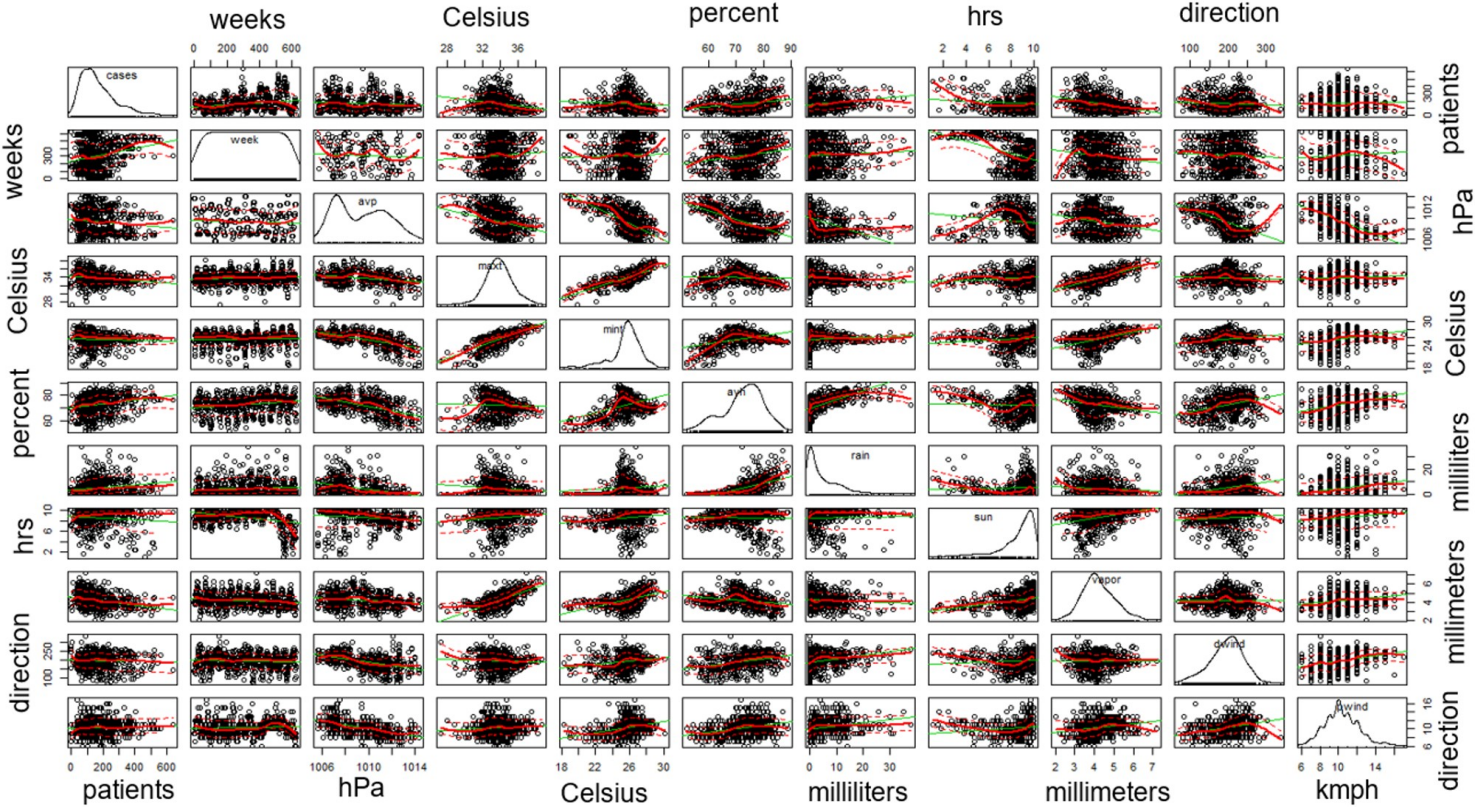
Pairwise scatter plots of the relations between dengue cases (patients), time series (weeks), pressure (hPa), maximum temperature (degree Celsius), minimum temperature (degree Celsius), humidity (percent), precipitation (ml), vaporization (ml), wind direction (direction), and wind power (kmph) of Bangkok, respectively, from column 1 to column 10 and row 1 to row 10. The diagonal of table represents the distribution of these variables. Each non-diagonal scatter plot shows the bivariate correlation between local column variable and local row variable.

We show the data used in this study in Figs 6 and 7. Fig 6 shows the distribution of dengue cases throughout Thailand in February 2013 when the highest number of dengue cases occurred. The dengue cases were plotted on different base maps, i.e. a province boundary map, the geographical map, population density map, and plain map. Fig 7 shows correlations between dengue cases and weather variables for Bangkok from January 2001 to December 2014. Every variables including dengue cases (patients), time series (weeks), pressure (hPa), maximum temperature (degree Celsius), minimum temperature (degree Celsius), humidity (percent), precipitation (ml), vaporization (ml), wind direction (direction), and wind power (kmph), are plotted horizontally from column one to column ten, and vertically from row one to row ten. The distribution of these variable are shown at the diagonal. Each scatter plot shows the correlation of each local column variable and local row variable.

Our study also includes a one to three-week lag time for each variable. Hence, the total independent weather variables are 32 variables. The effects of a one to three-week lag time on dengue cases are also used to construct the models. Therefore, we use a total of 35 independent variables. Thailand consists of a total of 77 provinces. However, dengue and weather data were recorded for only 65 provinces. Due to this data limitation, our study was conducted on 65 provinces in Thailand.

### Statistical analysis and modeling

In this study, we use generalized linear models (Poisson, negative binomial, and quasi-likelihood regression) and time series analysis (ARIMA, and SARIMA) to create the best fitted models for weather variations and dengue incidences.

#### Generalized linear models

Three Generalized linear models (GLMs) used in this study: Poisson, negative binomial, and quasi-likelihood regression, allow data that are not in a normal distribution and nonnegative integers. They are commonly used in epidemiology or biostatistical research [16, 18, 19, 21, 23, 25, 26, 29, 30, 32]. Excess zero counts, which cause overdispersion of the data, are allowed in these three methods.

Poisson regression is usually used to model countable data. The model allows overdispersion data, which is caused by excess zeros. A link function of the Poisson regression is the logarithmic function. Poisson regression is based on the normal distribution and assumes that the mean and variance are equal to zero.

Negative binomial regression is a subset of the Poisson regression method. This method assumes that the variance is not equal to the mean. This model is used for overdispersion data. The model is based on the Poisson-gamma mixture distribution, which also assumes that the data follows the normal distribution.

Quasi-likelihood is used for overdispersion data or parameters. Quasi-likelihood models ignore the assumption of a probability distribution for the data. In other words, it is not based on a normal distribution. Quasi-likelihood only requires that the variance is a function of the mean.

The GLM regression model used in this study is shown below:

$$\begin{array}{ccc} \ln(cases) & = & \beta_{0}+\sum_{i=0}^{3}(\beta_{1,i})\times (avp{)}_{i}+\sum_{i=0}^{3}(\beta_{2,i})\times (maxt{)}_{i}+\sum_{i=0}^{3}(\beta_{3,i})\times (mint{)}_{i}\\ & & +\sum_{i=0}^{3}(\beta_{4,i})\times (rain{)}_{i}+\sum_{i=0}^{3}(\beta_{5,i})\times (avh{)}_{i}+\sum_{i=0}^{3}(\beta_{6,i})\times (vapor{)}_{i}\\ & & +\sum_{i=0}^{3}(\beta_{7,i})\times (dwind{)}_{i}+\sum_{i=0}^{3}(\beta_{8,i})\times (pwind{)}_{i}\\ & & +\sum_{i=1}^{3}(\beta_{9,i})\times (cases{)}_{i} \end{array}$$ (1)

#### Time series analysis

The Autoregressive Integrated Moving Average (ARIMA) is a generalization of an autoregressive moving average (ARMA) model. Autoregressive (AR) includes all selected lag times, as possible. The moving average (MA) refers to the mean of the lag time and its error. Both models (AR and MA) are fitted to time series data either to better understand the data or to predict future incidences. ARIMA models are properly applied in cases where the data show evidence of non-stationarity.

The Seasonal Autoregressive Integrated Moving Average (SARIMA) is a type of ARIMA where seasons may have some effects on the outputs.

#### Constructing prediction models

The 35 independent variables that are used to construct the prediction models are: average temperature (*avp*), maximum temperature (*maxt*), minimum temperature (*mint*), average humidity (*avh*), precipitation (*rain*), vaporization (*vapor*), wind direction (*ddwind*), wind power (*pwind*), 1-week lag average temperature (*laggedavp1*), 2-week lag average temperature (*laggedavp2*), 3-week lag average temperature (*laggedavp3*), 1-week lag maximum temperature (*laggedmaxt1*), 2-week lag maximum temperature (*laggedmaxt2*), 3-week lag maximum temperature (*laggedmaxt3*), 1-week lag minimum temperature (*laggedmint1*), 2-week lag minimum temperature (*laggedmint2*), 3-week lag minimum temperature (*laggedmint3*), 1-week lag average humidity (*laggedavh1*), 2-week lag average humidity (*laggedavh2*), 3-week lag average humidity (*laggedavh3*), 1-week lag precipitation (*laggedrain1*), 2-week lag precipitation (*laggedrain2*), 3-week lag precipitation (*laggedrain3*), 1-week lag vaporization (*laggedvapor1*), 2-week lag vaporization (*laggedvapor2*), 3-week lag vaporization (*laggedvapor3*), 1-week lag wind direction (*laggeddwind1*), 2-week lag wind direction (*laggeddwind2*), 3-week lag wind direction (*laggeddwind3*), 1-week lag wind power (*laggedpwind1*), 2-week lag wind power (*laggedpwind2*), 3-week lag wind power (*laggedpwind3*), 1-week lag dengue cases (*laggedcases1*), 2-week lag dengue cases (*laggedcases2*), and 3-week lag dengue cases (*laggedcases3*). The outputs of the models are current dengue cases (*cases*).

Not all 35 independent variables are pertinent to dengue incidences. Therefore, we have conducted an exhaustive search to find the most relevant subset of potential variables that results in the best-fit prediction model for each province. From previous studies [19, 24–28], the prediction models use 4–9 independent variables. For prediction models with up to 9 independent variables, we have conducted an exhaustive search over all combinations. This means that for each type of GLM model (Poisson regression, Negative binomial regression, quasi-likelihood regression) in a province, there are $C_{1}^{35}+C_{2}^{35}+\cdots +C_{9}^{35}=77,663,191$ models constructed. For models with 10 to 12 independent variables, we use the Forward Selection method to test the next variables, for selecting the best model prediction. Hence, for each province, a total of 77, 663, 191 + 26 + 25 + 24 = 77, 663, 266 GLM models are created.

From the combination of the variables from 1–12 variables for each method, 77, 663, 266 models per method of GLM are created. We select from 1 to 9 variables because previous studies show that the best model usually uses 4 to 9 variables [19, 24–28]. We construct a total of 15, 144, 337, 000 models in this study. Each province uses a total of 232, 989, 800 models. We first use a combination of from 1 to 9 variables. Because of a limitation of computer performance, the next 10 to 12 variables continuously use the Forward Selection method to test the next variables, for selecting the best model prediction. Adding the two models from time series analysis (SARIMA and ARIMA), there are 232, 989, 798 + 2 = 232, 989, 800 prediction models constructed per province. Hence, for all 65 provinces, we have constructed 232, 989, 800 × 65, which is 15, 144, 337, 000 models, in total, for Thailand.

The computers that were used in this study have 2.2 GHz to 2.5 GHz CPUs. All models are created using R (programming language) version 3.4.3. We use 37 computers for calculating the models at the same time. It takes approximately 232, 989, 800/(120 × 60 × 60 × 24) = 22.472 days of computing time to construct all the models for a province. For all provinces of Thailand, it takes approximately 2 months to complete all experiments.

## Results and analysis

A summary of statistical analyses of the best models for all 65 provinces is shown in Tables 1 and 2. Significant values of the GLM models of the top mortality rate provinces in 2013 (Chiang Rai, Mae Hong Son, Chiang Mai, Phuket, Phang Nga, Krabi, Lampang, Loei, Khon Kean, Songkhla, Nakhon Phanom, and Bangkok) are shown in Tables 3 and 4.

**Table 1:** Summary of statistical analysis of the best models for 65 provinces in alphabetical order from Bangkok to Phuket: An individual province’s best model, accuracy by coefficient of determination, and variables used in the best model ranked by coefficient magnitude.

Province	M	Cases			avp				mint				maxt				avh				rain				vapor				dwind				pwind				*R*^2^
		*l*1	*l*2	*l*3	*l*0	*l*1	*l*2	*l*3	*l*0	*l*1	*l*2	*l*3	*l*0	*l*1	*l*2	*l*3	*l*0	*l*1	*l*2	*l*3	*l*0	*l*1	*l*2	*l*3	*l*0	*l*1	*l*2	*l*3	*l*0	*l*1	*l*2	*l*3	*l*0	*l*1	*l*2	*l*3	
Bangkok	Q	2	3						5						4						7			1								8			6		0.724
Buriram	Q	1						5				7		2	6	4		8			3																0.896
Chachoengsao	Q		1	3								7									4				2								5	8		6	0.391
Chai Nat	Q	1	2	7	3												8				6			4									5				0.139
Chaiyaphum	Q	2		3					6				5							7				8				1	4								0.837
Chanthaburi	Q	2	4	3	1							7		8		6					5																0.836
Chiang Mai	Q	1		2					4				7							6				3				5	8								0.937
Chiang Rai	Q	1				7											5								2		3					6				4	0.899
Chonburi	Q	1		3									2									8		7		6			4					5			0.789
Chumphon	P	2			1	3	4															8	7							10	5	9	6				0.618
Kalasin	NB	1		2		6		4																							8	5	7			3	0.708
Kamphaeng Phet	Q	1			2		8	4					9					5			11										10		6		7	3	0.880
Kanchanaburi	NB	2	3		1								5		4																	7				6	0.509
Khon Kean	Q	1	2																		6					4	5	3									0.902
Krabi	NB				1		3		4	6	5																				2						0.637
Lampang	Q	1	2	3													4			7	6	8				5											0.954
Lamphun	Q	1	2												6					7		3		8	5		4										0.819
Loei	Q	1	2																						3			4			5	6					0.928
Lopburi	Q	1						2					5			3								4					6		7						0.505
Mae Hong Son	NB	4		10	1								8	5		6		2	9				7					3			11						0.909
Maha Sarakham	Q	1		2																4							3						8	6	5	7	0.913
Mukdahan	Q	1																			5	8	7	6				4		3	2						0.851
Nakhon Pathom	Q	1	2								5				4		7				9	8								3			6				0.830
Nakhon Phanom	NB	1															3					4	6	5							2						0.607
Nakhon Ratchasima	Q	2				1											3								5		7					3				6	0.303
Nakhon Sawan	Q	1	2													4	7							8				3				9	5			6	0.614
Nakhon Si Thammarat	Q	1	4	5	7	6	8						2												3						9						0.884
Nan	Q	1	2	6																8	4		7			3	5										0.863
Narathiwat	Q	1	2		5	6						4				7					3					8						9					0.788
Nong Khai	P	3				2										1			4		7		8						6			5					0.746
Pathum Thani	Q		1	2						3		5				4						8		7		6											0.574
Pattani	Q	1	2	5	7					6								3					8	4													0.882
Phangnga	Q	1	2	7			8												6									3						5	4		0.430
Phatthalung	NB	1																		5			7	6	4				8			3		2			0.434
Phayao	NB		1																6													4	5	3		2	0.473
Phetchabun	Q	1						4			3												6							5		7			2		0.521
Phetchaburi	Q	1		2		5			3		4	6									9	7	8														0.524
Phichit	P				1		4		5		2																3		8				7		6		0.715
Phitsanulok	Q	1	2	4																		5					3			7	8	6					0.221
Phrae	P	3	4		1		8	6			2																		7			5					0.864
Phuket	Q	1		2		4			3										8	7														6	5		0.794

*R*^2^: coefficient of determination, Q: Quasi-likelihood regression model, P: Poisson regression model, NB: Negative binomial regression model, *l*0–3: Lag 0–3

**Table 2:** Summary of statistical analysis of the best models for 65 provinces in alphabetical order from Pha Nakhon Sri Ayutthaya to Yala an individual province’s best model, accuracy by coefficient of determination, and variables used in the best model ranked by coefficient magnitude.

Province	M	Cases			avp				mint				maxt				avh				rain				vapor				dwind				pwind				*R*^2^
		*l*1	*l*2	*l*3	*l*0	*l*1	*l*2	*l*3	*l*0	*l*1	*l*2	*l*3	*l*0	*l*1	*l*2	*l*3	*l*0	*l*1	*l*2	*l*3	*l*0	*l*1	*l*2	*l*3	*l*0	*l*1	*l*2	*l*3	*l*0	*l*1	*l*2	*l*3	*l*0	*l*1	*l*2	*l*3	
Pha Nakhon Sri Ayutthaya	NB	1	2				4															7			3									5	6		0.225
Prachin Buri	NB			2				1												5								3		6			4				0.759
Prachuap Khiri Khan	Q	2		3		1						4								7											8		6			5	0.481
Ranong	P		3	4	1											2		5								6	7	8									0.506
Ratchaburi	Q	2	3		1								4		7										6			5			9					8	0.739
Rayong	NB		1	3											4			2					6												5		0.714
Roi Et	Q	2	3	5			1										4				8			7												6	0.755
Sa Kaeo	NB	2	3	5				1												4			6														0.317
Sakon Nakhon	Q	1	5					3										7			6	4												2			0.026
Samut Prakan	Q	1	2																		5			4											3		0.495
Satun	NB	2	3		5						1		4		6									7													0.270
Si Sa Ket	Q		1					3											2					5									6	7	4		0.565
Songkhla	NB	2	4						1			5							7	6								3									0.510
Sukhothai	NB			2		1											5				6		4											3			0.642
Suphan Buri	Q	1	2	3					4	6					7	5					8																0.299
Surat Thani	Q	1		2				4									3					7	6	8				5									0.692
Surin	Q	1	3	2															5					6		4											0.700
Tak	NB	2														1										3	4									5	0.769
Trang	P	4			2								3								5					1		7				6					0.534
Trat	NB		3			1					2														5			4						6			0.541
Ubon Ratchathani	Q	1	2														4			5								3				8		7	6		0.682
Udon Thani	Q	1	4	6														3			7			5			2										0.257
Uttaradit	Q	1		2	8															6						5						7		4		3	0.454
Yala	Q	1					4			3		5				2								6													0.631

*R*^2^: coefficient of determination, Q: Quasi-likelihood regression model, P: Poisson regression model, NB: Negative binomial regression model, *l*0–3: Lag 0–3

**Table 3:** Statistical analysis of negative binomial, Poisson, quasi-likelihood regression model presents the coefficient of model prediction for the most incidences and morality rate provinces in 2013 (Chiang Rai, Mae Hong Son, Chiang Mai, Phuket, Phang Nga, Krabi). The coefficient of determination and root mean square error are compared.

Variables	Lag	Chiang Rai			Mae Hong Son			Chiang Mai			Phuket			Phang Nga			Krabi		
		NB	P	Q	NB	P	Q	NB	P	Q	NB	P	Q	NB	P	Q	NB	P	Q
Intercept	N/A	−9.21	−32.44	−26.94	98.09	323.25	257.97	−30.42	−8.53	−35.67	−4.79	−5.85	158.06	−2.19	−2.81	−7.09	431.79	347.20	7177.00
*cases*	1	0.03		0.90	0.04		0.65	0.01	0.00	1.21			0.78	0.04	0.03	0.60			
	2						0.29							0.02	0.01	0.25			
	3		0.01		0.01					−0.26			0.16	0.00	0.01	0.05			
*avp*	0	−0.08	−0.08		−0.11	−0.16	−0.26										−0.24		
	1		0.11	0.02				0.03					−0.18						
	2								0.00							0.00	−0.19	−0.34	−5.03
	3					−0.17													−2.07
*mint*	0							0.07		1.21							0.05		
	1					−0.06		0.14					0.80				0.01	0.05	
	2					0.06					0.10	0.12					−0.03	0.00	
	3	0.16	0.32			0.12					0.12	0.11							
*maxt*	0				−0.02				0.20	0.06									
	1				0.17									0.04					
	2	0.03				−0.02			0.05										
	3		−0.06		0.14		0.06												
*avh*	0			0.02					0.08										
	1				0.07	0.06	0.15												
	2	0.04			0.02								−0.05	0.02	0.05	0.06			
	3						−0.13			0.07			0.07						
*rain*	0						−0.10		−0.03									0.00	−0.09
	1						−0.13								0.00			0.00	
	2				0.02	−0.03	−0.07								−0.01			0.00	
	3					−0.04				−0.93	0.01	0.01							−0.07
*vapor*	0	0.08		1.16				−0.11				0.05			0.07				
	1						0.12												
	2			−0.27															
	3				−0.05					0.77	−0.06			0.09	0.07	0.66			
*dwind*	0	0.00								0.03		0.00							0.01
	1	0.00	0.00								0.00							0.00	
	2				0.00	−0.01											0.05		
	3	0.00		0.02								0.00						0.00	
*pwind*	0					0.01	0.10						−0.13						
	1					−0.03										0.07			
	2						0.11				0.07	0.06	0.15			−0.10		0.04	0.85
	3		0.12	0.28								0.04							
Test set (2014)	RMSE	12.50	11.12	7.27	3.82	5.66	4.38	49.55	55.24	29.91	6.69	6.37	4.35	2.91	3.47	2.22	8.43	9.43	9.47
	*R*^2^	0.84	0.76	0.90	0.91	0.80	0.88	0.78	0.83	0.94	0.51	0.56	0.79	0.02	−0.39	0.43	0.64	0.55	0.54
ARIMA (3,1,4)	RMSE	19.46			18.82			140.00			10.19			14.22			15.93		
	*R*^2^	0.28			0.21			−0.17			0.13			−22.37			−0.29		
SARIMA(2,0,1) (0,2,0)52	RMSE	21.79			43.08			101.45			49.65			28.28			32.49		
	*R*^2^	0.08			0.00			0.27			0.00			−91.45			−4.39		

RMSE: Root mean square error, *R*^2^: coefficient of determination, Q: Quasi-likelihood regression model, P: Poisson regression model, NB: Negative binomial regression model

**Table 4:** Statistical analysis of negative binomial, Poisson, quasi-likelihood regression model presents the coefficient of model prediction for the most incidences and morality rate provinces in 2013 (Lampang, Loei, Khon Kean, Nakhon Phanom, Songkhla, Bangkok). The coefficient of determination and root mean square error are compared.

Variables	Lag	Lampang			Loei			Khon Kean			Nakhon Phanom			Songkhla			Bangkok		
		NB	P	Q	NB	P	Q	NB	P	Q	NB	P	Q	NB	P	Q	NB	P	Q
Intercept		360.55	−2.45	−4.05	−14.58	248.60	−2.75	257.00	276.20	−4.15	−0.74	−0.51	70.08	−1.65	−2.71	189.23	4.97	4.45	58.41
*cases*	1			0.90			0.66			0.68	0.05	0.02	0.94	0.01	0.01	0.63	0.00	0.00	0.67
	2		0.02	0.34			0.25			0.26					0.00				0.12
	3			−0.32										0.00	0.00	0.25		0.00	
*avp*	0	−0.02						−0.06	−0.07										
	1	−0.09						−0.06	−0.06				−0.07			−0.62			
	2	−0.24																	
	3					−0.25		−0.13	−0.14							0.44			
*mint*	0													0.15	0.10		0.02	0.02	3.33
	1														0.07				
	2														0.05				
	3													0.06	0.05				
*maxt*	0	−0.13																	
	1																		
	2								−0.03							−0.16	−0.03	−0.02	−3.63
	3														−0.01				
*avh*	0					0.02					0.02	0.01							
	1		0.02	0.12	0.08							0.02							
	2													0.01					
	3			−0.07	0.07	0.02						0.00		−0.01					
*rain*	0	−0.02	0.00	−0.07						−0.05		0.00			0.00				0.34
	1	−0.01		−0.04							0.00		−0.03						
	2	0.00							−0.01		0.00				0.00				
	3		0.01								0.00						0.02	0.01	2.16
*vapor*	0				0.25	0.26	0.66										−0.05	−0.03	
	1			0.44	0.54	0.17				0.71									
	2				0.24	0.15		−0.11		−0.45						0.58			
	3				0.51	0.21	0.48	−0.09		0.95				−0.08		−0.27			
*dwind*	0		0.00																
	1		0.00																
	2		0.00			0.00	0.00				0.00		0.01						−0.02
	3		0.00				0.00	−6.47	0.00										
*pwind*	0							−0.01	−0.01										
	1	−0.01							−0.01										
	2																		0.57
	3																		
Test set (2014)	RMSE	60.62	38.35	13.53	38.73	40.76	11.70	43.75	43.66	15.79	2.08	4.09	2.10	11.16	13.18	11.92	37.02	40.73	28.05
	*R*^2^	0.08	0.63	0.95	0.22	0.13	0.93	0.25	0.25	0.90	0.61	−0.52	0.60	0.51	0.32	0.44	0.52	0.42	0.72
ARIMA (3,1,4)	RMSE	3.19			4.54			13.02			4.74			35.53			60.60		
	*R*^2^	−0.48			−4.38			−2.19			−1.04			−3.97			0.29		
SARIMA (2,0,1)(0,2,0)52	RMSE	108.20			77.27			82.22			74.13			85.22			204.04		
	*R*^2^	−1699.14			−1559.44			−126.16			−498.86			−27.60			0.00		

RMSE: Root mean square error, *R*^2^: coefficient of determination, Q: Quasi-likelihood regression model, P: Poisson regression model, NB: Negative binomial regression model

The detailed results of each province are provided in S1 Appendix including:

Scatter plot for dengue cases (*cases*) vs. selected independent variables, using the weekly period during January 2001–December 2013 (*week*), average pressure (*avp*), maximum temperature (*maxt*), minimum temperature (*mint*), average humidity (*avh*), precipitation (*rain*), vaporization of water (*vapor*), wind direction (*dwind*), and wind power (*pwind*).
Line plot for dengue incidences vs. weeks: the plot shows trends of dengue incidences in each year as a stationary time series and a histogram of dengue incidences starting from January 2001 to December 2013 (624 weeks).
Three-dimensional scatter plot between dengue incidences and weather effects starting from January 2001 to December 2013.
(a) Two plots for lag-time of dengue incidences and ACF vs. PACF, calculated from the ARIMA model (b) Summary plots of time series analysis, multiple plots include a plot of the predicted model vs. time, plot of ACF residual vs. lag-time of dengue incidences, residual Q-Q plot of standard residual, and *p*-value for Ljung-Box statistics of PACF over the training data starting from January 2001 to December 2013.
Comparison table of all methods by the highest coefficient of determination (*R*^2^) and the lowest prediction error (RMSE).
Plots of observed and predicted dengue cases of the best fit model of each technique over the test set data starting from January 2014 to December 2014.
(a) Plot between observed and predicted dengue incidences over weekly time by the best model of ARIMA and (b) SARIMA time series analysis from January 2014 to December 2014.
Coefficients and significant values of the best-fit GLM models, Negative Binomial, Poisson and Quasi-likelihood regression model are presented in alphabetical order.

### Best-fit prediction model analysis

Our prediction models for all 65 provinces have an average coefficient of determination of 0.6339 (95%CI: 0.57802, 0.6898), with a minimum of 0.026 at Sakon Nakhon and a maximum of 0.954 at Lampang. Our experimental results reveal that the highest-accuracy models for all 65 provinces are one of the GLM models. Moreover, the best models of most provinces (44 out of 65) are based on quasi-likelihood regression. The best models of 15 provinces are based on negative binomial regression and the best model of 6 provinces is based on Poisson regression. This finding suggests that best describe the relationship of weather variables and dengue data should not have distribution assumptions. In most provinces, the data is not normally distributed, since most models are based on quasi-likelihood, not negative binomial or Poisson regression, Both of which assume the normal distribution. In our study, time series analysis is inferior to GLM models in all provinces. This suggests that time series analysis may not be the best type of model to describe the relationships among weather variables and dengue incidences. Evidence for this inferiority can be found in Chiang Rai. From a previous study in Chiang Rai, Thailand [29], the SARIMA method was used for constructing a prediction model. They did not report the coefficient of determination between the predicted cases and actual cases. From the Chiang Rai analysis in S1 Appendix, the Quasi-Likelihood prediction model matches the observed data better than the SARIMA prediction model. In fact, for Chiang Rai data, SARIMA has the least coefficient of determination between the predicted cases and actual cases among all 5 techniques used in this study. Since all the provincial-level best-fit prediction models are GLM, we can indicate how influential an independent variable is to dengue incidences from its statistical *p*-value.

#### Previous cases, variable analysis

From our study, the highest significant variable associated with dengue incidences is the positive 1-week-lag dengue cases in most of the provinces in Thailand, except in Phichit, Sukhothai, Si Sa Ket, Chachoengsao, Trat, Pathum Thani, Prachin Buri, Rayong, Krabi, and Ranong provinces. The best-fit model for all provinces contains some weather factors.

The one-week-lag time variable in most provinces has a positive association with predicted dengue incidences. In time series analysis, we fitted the Autocorrelation functions (ACF) and Partial Autocorrelation Functions (PACF). An in-depth PACF analysis of each province can be found in S1 Appendix. From the results, 1-week-lag cases provide the highest correlation among all provinces. The best model that contains the 1-week-lag case variable reported a coefficient of determination of a minimum of 0.257 and a maximum of 0.954 (average of 0.6383, 95%CI: 0.57313 to 0.70355). However, in some provinces, the highest significant variables are not the 1-week-lag cases but the 2-week-lag cases (such as Ranong, Si Sa Ket, Chachoengsao, Trat, Pathum Thani, and Rayong) or 3-week-lag cases (such as Sukhothai and Prachin Buri). These results show that the effects of previous cases on dengue incidences can be for up to 3 weeks. Our results show that the PACF provide a strong possible number of lag times, from 1–4 weeks of lag time, which presents the highest relationship at a lag of one week. This indicates the significance of past cases associated with current cases as a serial relationship in Thailand. Our results show the strong relationship of the period of a small lag time, from one to three weeks. Our findings for the relationships of small lag time and dengue incidences agrees with the classic SIR model, which considers the small-time delay that causes the threshold for epidemic diseases. Our results also support the fact that mosquitoes, as a dengue disease carrier, could contain the initial dengue viral vector for 1–14 days. This is also known as the extrinsic incubation period. A period of one month presents over-critical dengue incidences by vertical transmission of dengue virus due to the rapid infection from adult to offspring mosquitoes. Dengue cases at the first lag time are a predictive factor for most of the best model predictions. Because the stability of the dengue virus is in endemic equilibrium depending on delay times, a delay time of from 7 to 14 days is taken as the incubation time for the infective virus to transform into an active viral disease. Thus, vector diseases could stay in the carriers through the next 1 or 2 weeks. The importance of previous cases is shown in the literature [16, 17, 21, 24, 27, 33].

### Weather variable analysis

Even though previous cases have strong effects on dengue cases in most provinces, the best-fit model of two provinces, i.e., Phichit and Krabi, contain solely weather variables, with no previous case variables. The best-fit prediction model yields a high coefficient of determination of 0.715 (Phichit) and 0.637 (Krabi). These two provinces indicate that previous cases (alone) are not enough to accurately predict future dengue cases. Weather variables play non-negligible roles in highly accurate prediction models.

Although the previous dengue cases are significantly associated with dengue incidences, weather variables do have merit in the prediction models. In fact, the best models for all provinces contain some weather variables. The most frequent weather variable among all provinces is the current-week precipitation, which is used in 20 provinces. The second frequent weather variable is the 3-week-lag precipitation, which is associated with predicted dengue incidences. In some provinces, like Bangkok, both the current-week precipitation and the 3-week-lag precipitation are used in the best-fit prediction model. Our results show that precipitation for up to three weeks has a high (positive and negative) influence on dengue incidences. Although heavy precipitation could convey the mosquito’s larva away at the current week, the amount of precipitation can substantially affect a mosquito’s habitat by increasing water storages for the eggs, which has positive correlation with the 3-week-lag precipitation. This duration corresponds to the life cycle of mosquitos which take up to 14 days to become an adult period. High precipitation for 30 days has a substantial effect, rapidly increasing the risk of infection for dengue, as shown in our pairwise plots. This may be because precipitation initially creates the offspring habitats for mosquito reproduction. More precipitation induces an increased risk of infection. In contrast, massive precipitation can decrease mosquito habitats by destroying the mosquitoes’ offspring in water storage or convey mosquito larvae away. Therefore, our results present both positive and negative relationships for precipitation, depending on the location in each province. Our findings strongly show that precipitation is the most influential weather factor for all provinces of Thailand. From Fig 7, some of the highest cases occur at low precipitation. However, theses cases contains higher 1 to 3-week-lag precipitation. High cases may also correspond to the life cycle of mosquitos which take up to 14 days for a female mosquito to become fully grown.

Wind direction and wind power are also influential weather variables. For example, the best-fit model of Phayao province, which uses both wind direction and power factor, shows the highest dengue incidences for 5–6 knots of wind power. This suggests that wind power is significant for the dispersion of dengue by mosquitos. The positive coefficient in many provinces shows that higher wind power may affect dengue incidences. The more wind power on water surface, the larger the region of evaporation. The increase in humidity may help adult mosquitos to survive longer and spread dengue. Wind direction variables are most influential in provinces located in northern and northeastern Thailand. These regions are mountainous areas. Human populations are not spread equally in all directions. A suitable wind direction may help distribute dengue carriers to a more populated area. This can result in a wider spread of dengue incidences.

Current and lag pressure effects are minor weather variables for all provinces of Thailand. In our results, pressure has negative associations to dengue cases. Average pressure also has a negative association in many provinces, especially in the southern and northern regions. Because the northern provinces are mountainous, there is a seasonal pattern leading to a decrease in local pressure and temperature. Lower pressure intensifies the water storage in the environment by increasing precipitation. The seasonal pattern in the northern provinces causes an increase in the number of mosquitoes, leading to more dengue viral cases while increasing the residence time of incubation, which indicates a pressure-controlled variable.

For the temperature and time-lag effects, our results show that the maximum temperatures from current to 3 week-lag time are more influential in Thailand than the effects of minimum temperatures. However, the best-fit model of a province with a high dengue mortality rate usually contains an influence of the minimum temperature. For example, in Songkhla province, the minimum-temperature variable indicates that there is a high dengue case occurrence at approximately 24–26 degrees Celsius. Previous studies have shown that the minimum temperature has a positive association with dengue incidences. The association of dengue incidences with temperature change has been widely studied across lag times of up to 20 weeks. The minimum temperature has a significant impact on dengue epidemics. Our results show that the current-week minimum temperature is highly significant for the southern, eastern, and central regions.

Beyond the weather variables used in this study, the diurnal range of temperature is an important weather factor that influences dengue transmission. The correlation between dengue incidences and diurnal ranges of temperature has been studied in Bangladesh and Sri Lanka [34, 35]. Including this variable in future models may help improve the accuracy of the models.

### Regional analysis

In the northern region, the 1-week-lag case variable is the highest significant factor associated with dengue incidence in most of the provinces, except Phichit and Sukhothai. Phichit province was not included for the previous case variable in the best model. For Sukhothai province, the highest significant variable is 3-week-lag dengue cases. Considering overall regional weather, the highest significant weather variables are ordered as the following: negative current-week pressure, 3-week-lag wind power, 2-week-lag vaporization, and 3-week-lag wind direction. The accuracy by the coefficient of determination in this region is (on average) 0.7208 (95%CI: 0.6067, 0.8349), with a minimum of 0.22 for Phitsanulok to a maximum of 0.954 for Lampang. The northern provinces are mountainous and are higher from sea level than the other provinces. The pressures are lower so that there may be an increase in the mosquito population and the dengue virus. For example, in Lampang province, the results show a high dengue incidence at low pressure. The most frequently used weather variables in the northern region are the 3-week-lag humidity and 3-week-lag wind direction. Humidity, temperature, and dengue cases have a positive correlation. In Tak province, the results show that high humidity and high temperature increase the dengue cases. The results also support the strong positive relation of the wind direction with dengue cases. The second frequent weather variable in the northern region is the 3-week-lag wind power. As seen in Phrae province, the results show that moderate wind power leads to high dengue cases. Other frequent weather variables are the negative current-week pressure, positive 2-week-lag rainfall, and negative 2-week-lag vaporization. These values are also supported by Mae Hong Son province. For general weather effects, the best model for fitting the northern region is the quasi-likelihood model (10 provinces out of 16 provinces), followed by negative binomial regression, Poisson regression model, and time series analysis. The quasi-likelihood regression method yields an average coefficient of determination of 0.7161 (95%CI:0.5374, 0.8947). Negative binomial regression has a coefficient of determination from 0.473 for Phayao to 0.909 for Mae Hong Son province with an average of 0.69825 (95%CI: 0.4029, 0.9935). The Poisson regression coefficient of determination is 0.715 for Phichit and 0.864 for Phrae province with an average of 0.7895 (95%CI: 0.1571, 1.7361). Time series analysis is well-fit only in Chiang Mai, Chiang Rai, Mae Hong Son and Kamphaeng Phet provinces, where the coefficient of determination is from 0.081 in Chiang Mai to 0.724 in Kamphaeng Phet. For Phichit province, the results show that the previous cases have no significance for predicting dengue incidences. The association of the cases is small in the first row and column of the pairwise plot.

In the northeastern region, the most significant previous case variable is the positive 1-week-lag case variable, except for Si Sa Ket province. In Si Sa Ket province, the positive 2-week-lag case variable is the most significant previous case variable. The coefficient of determination in the northeastern region is from 0.026 for Sakon Nakhon to 0.929 for Loei province, with an average of 0.6672 (95%CI: 0.5267, 0.8076). For determining the overall weather effects, the most significant weather variable is the positive 3-week-lag vaporization. The northeastern region is a high-level plain area called the Khorat plateau, in which sunlight can easily penetrate to the land, compared with other regions, leading to higher vaporization. When vaporization rapidly increases, temperature and dengue incidences also proportional increase, as shown in the best-fit model of Chaiyaphum province. Other significant weather variables are the negative 1-week-lag pressure and the 2-week-lag wind direction. The significance of the first-lag pressure to dengue incidences was supported by Wongkoon et al., 2011. Nakhon Ratchasima and Nong Khai provinces have a high dengue incidence at low pressure. The northeastern region depends on the 1-week-lag pressure, supporting the results of Nong Khai province. For the 2-week-lag wind direction, the direction is approximately 180 to 200 degrees (from Nong Khai and Nakhon Phanom provinces), which refer to the southeast wind from the South China Sea in the local monsoon climate system [36, 37]. The wind supports the pathway of dengue transmission of the northeastern area before passing through other regions. The most frequent weather variables are the negative current week and the 3-week-lag precipitation. Our results of Udon Thani show that low precipitation induces a high number of dengue cases. The second weather variable is the 3-week-lag vaporization. The best model in the northeastern region is the quasi-likelihood model, followed by negative binomial regression, Poisson regression model, and time series analysis. Time series analysis cannot be used to predict dengue incidences in this region. The quasi-likelihood regression method (13 provinces out of a total of 16 provinces) has a coefficient of determination from 0.026 to 0.929 with an average of 0.6626 (95%CI: 0.4856, 0.8397). The negative binomial regression method has a coefficient of determination from 0.607 for Nakhon Phanom to 0.708 for Kalasin, and the coefficient of determination of the Poisson regression is 0.746 for Nong Khai province.

In the central and eastern regions, the most significant variable is the positive 1-week-lag cases, except Chachoengsao, Trat, Pathum Thani, Prachin Buri, and Rayong provinces. The coefficient of determination in the central and eastern regions is from 0.138 for Chai Nat to 0.836 for Chanthaburi province, with an average of 0.5440 (95%CI: 0.4417, 0.6464). In the central and eastern regions, the most significant weather variables are the negative current week and 1-week-lag pressure variables. Another frequent variable is the 2-week-lag maximum temperature. In Trat province, the maximum temperature is linearly associated with dengue cases, but the relation to pressure is the reverse. The central and eastern regions are a low-level plain and the Gulf of Thailand (Meteorological Development Bureau, 2015). The temperature in this region favors mosquitoes. Therefore, the temperature factors are important for dengue incidences. The most frequent weather variables in this region are the positive current week, 3-week-lag precipitation, and 2-week-lag maximum temperature. This area is the center point of all rivers in Thailand. When precipitation rises, it can cause an increase in dengue incidences in the area. As shown for Bangkok, the plot shows the relationship of precipitation to dengue cases, with the highest cases at low precipitation. Another frequent weather variable is the 3-week-lag wind power. As shown in Prachuap Khiri Khan, wind power in a certain direction increases dengue incidences. The best selection models in the central and eastern regions are the quasi-likelihood model, negative binomial regression model, and time series analysis. Poisson regression is not used in this region. For the quasi-likelihood regression model, the coefficient of determination is from the lowest (0.138) to the highest (0.836) with an average of 0.5420 (95%CI: 0.4146, 0.6695). The negative binomial regression model has a coefficient of determination from 0.225 for Pra Nakhon Sri Ayutthaya to 0.759 for Prachin Buri province (Average of 0.5496, 95%CI: 0.2877, 0.8115). Time series analysis is used to fit only Bangkok, Chanthaburi, and Ratchaburi province, where the coefficient of determination is from 0.287 to 0.472. From the result of Chanthaburi, the predictions of time series analysis by SARIMA have sufficient dengue incidences.

In the southern region, the most significant previous case variable is the positive 1-week-lag case variable, except for Krabi and Ranong provinces. In Ranong, the 2-week-lag cases are the most influential variable. Nevertheless, the best-fit model for Krabi province does not include the previous cases. The coefficient of determination is from 0.271 for Satun to 0.884 for Nakhon Si Thammarat, with an average of 0.614857 (95% CI: 0.51033, 0.7194). The most significant weather variable is the negative current-week pressure. As shown in the model of Chumphon province, the pressure factor shows a relationship to dengue cases, in which a low pressure leads to high incidences. Since the topography of the area is a peninsula between the Andaman Sea and the South China Sea, the pressure change has a significant effect on each individual location. The second significant weather variable is the positive current-week maximum temperature. The southern region has a tropical rainforest climate system where the temperature is warm throughout the year. Therefore, the effects of temperature are influential, as shown in Trang province. When the temperature increases, the rate of virus infection also increases. Other significant weather variables are the negative 1-week-lag case and the 3-week-lag vaporization. This can be seen in the model of Trang province where water vapor plays an important role in dengue incidences at moderate vaporization. The most frequent weather variables in the southern region are the negative current week, 1-week-lag case, and 2-week-lag pressure factors which all have the same significance. Other frequent weather variables are the 2-week-lag precipitation and 3-week-lag vaporization. This can be observed from the model of Trang province. In this region, quasi-likelihood regression is the best model method, followed by negative binomial regression, Poisson regression, and time series analysis. The quasi-likelihood regression model can predict the results. The coefficients of determination are from 0.43 for Phang Nga to 0.884 for Nakhon Si Thammarat (average of 0.4630, 95% CI: 0.2196, 0.7064). Negative binomial regression yields a coefficient of determination from 0.271 for Satun to 0.637 for Krabi (average of 0.5527, 95% CI: 0.4079, 0.6975). Also, Poisson regression yields a coefficient of determination from 0.506 for Ranong to 0.618 for Chumphon province. For Krabi province, which is independent of the previous case variables, the best model consists of the negative current-week pressure, positive 1-week-lag pressure, negative 2-week-lag pressure, and the positive 2-week-lag wind direction at high significance. The results show the substantial effects of pressure and wind direction. The wind direction was supported by the topography of the surrounding seas that are parallel to the southern area [37].

### High mortality, provincial analysis

From the best-fit model of provinces with the highest dengue incidences in 2013, the most significant variable is the 1-week-lag case variable, except for Krabi province. The best predictive model for Krabi province does not include the previous case variables as we explained for the southern region. In provinces that have many patients, the most significant weather variables are the positive current-week minimum temperature and the 3-week-lag vaporization as shown in the model for Mae Hong Son province. There is an increase of dengue incidences when both the minimum temperature and the vaporization increase. In addition, the effects of wind direction are also significant. The model of Nakhon Phanom shows that the direction of the wind which passes through this region has a strong influence on the number of dengue incidences. The most frequent weather variable for the selected provinces is the 3-week-lag vaporization as shown in the model of Mae Hong Son province. Other frequent weather variables are the positive current-week minimum temperature and the 2-week-lag wind direction. The best-fit model in these selected provinces is quasi-likelihood regression which has coefficients of determination from 0.43 to 0.954 for Lampang (0.78625, 95%CI: 0.6747, 0.8978). Negative binomial regression is used in some provinces which yields coefficients determination from 0.51 for Songkhla to 0.909 for Mae Hong Son province (0.7855, 95%CI: 0.4333, 1.13768). Time series analysis is performed only in Chiang Rai, Mae Hong Son, Chiang Mai, Phuket, and Bangkok. Chiang Rai has coefficients of determination from 0.081 to 0.277. The selected provinces were supported by previous studies [9, 29].

### Limitation

The dengue case data used in our study are clinically suspected cases collected from every state hospital in Thailand. Some of the cases maybe infections with similar clinical manifestation, like Chikungunya and Leptospirosis.

## Conclusion

We constructed predictive models to forecast provincial-level future dengue cases based on weather and dengue incidences from 2001 to 2014 in Thailand. There are a total of 35 independent variables, which include the one to three week previous cases and the 0 to 3 week lag for each weather variable (average temperature, maximum temperature, minimum temperature, average humidity, precipitation, vaporization, wind direction, and wind power). By using a combination of 1–12 variables to find the best model, we create a total of 77,663,266 models for one method of the generalized linear model. We create predictive models based on three statistical regression models and two time-series analysis methods: Poisson, negative binomial, quasi-likelihood regression models and ARIMA and SARIMA series analysis methods. The models for each province were combined for 2–12 independent variables. In total, 232,989,800 models are built for a province. We construct 15,144,337,000 models for all provinces in Thailand. For all provinces, the model with the highest coefficient of determination is the quasi-likelihood regression model, which is chosen as the predictive model. We then use the three best predictive models to further analyze the relations among weather variables and dengue cases using pairwise scatter plots, line plots, and ACF and PACF to investigate our results. The results show an average coefficient of determination of 0.6339 (95%CI: 0.57802, 0.6898) for all provinces, which are from a minimum of 0.026 in Sakon Nakhon to a maximum of 0.954 in Lampang.

From our study, we found that the 1-week-lag case variable (coefficient of determination from a minimum of 0.026 to a maximum of 0.954, average of 0.6383, 95%CI: 0.57313 to 0.70355) is the best predictor associated with dengue incidences for all provinces. However, some provinces are predictable without previous case variables. The 2-week-lag case (coefficient of determination from a minimum of 0.391 to a maximum of 0.714, average of 0.5485, 95%CI: 0.4384, 0.6585) and 3-week lag case (coefficient of determination from a minimum of 0.642 to a maximum of 0.759, average of 0.7005, 95%CI: 0.0428, 1.4438) variables are the most influential variables in many provinces.

For weather variables, the most frequent weather variable is the current-week precipitation, followed by the 3-week-lag precipitation, 3-week-lag wind direction, and the negative current-week pressure. This shows that the precipitation (up to a 3-week lag) significantly affects an outbreak of dengue, especially for a tropical country like Thailand.

Our results show that in each province, a different set of weather variables should be used in the best prediction model. Different geographical and spatial locations may be the cause of local effects that lead to a different dengue model. Each province has its unique local factors that cannot be generalized to other provinces. Our results indicate that the independent weather variables needed to accurately predict future dengue cases are not fixed. These variables are locally determined. Hence, the best prediction models should be constructed at a local level, such as the provincial level, not on a larger scale, such as the national level. The application of this study provides high accuracy from the predictive model for the predicted weather parameters. The public health institute may use these models in considering future strategies and hospital preparation. Thailand is a small country in Southeast Asia and our results show different independent variables in each province. The individual geographical locations are the factors that could be important for dengue incidences. We can observe the relations of variables overlapping (by areas) in the same province. Weather variables of the model are locally determined so that a province-level model provides an appropriate and accurate model. A dengue prediction model should be determined on a small scale, not on a large scale such as the national level. In conclusion, the best model prediction depends on the geographical variability of each model location. With the presented accuracy, our models can be used to predict the dengue incidences at the provincial level in the future.

## Supporting information

S1 Appendix Complete provincial level analysis of weather variables and dengue cases. (PDF)

## References

[1] Gubler DJ. Dengue and dengue hemorrhagic fever. Clin Microbiol Rev. 1998;11(3):480–496. 10.1128/CMR.11.3.480 9665979 PMC88892

[2] Gubler DJ. Epidemic dengue/dengue hemorrhagic fever as a public health, social and economic problem in the 21st century. Trends Microbiol. 2002;10(2):100–103. 10.1016/s0966-842x(01)02288-0 11827812

[3] Rigau-Pérez JG, Clark GG, Gubler DJ, Reiter P, Sanders EJ, Vorndam AV. Dengue and dengue haemorrhagic fever. Lancet. 1998;352(9132):971–977. 10.1016/s0140-6736(97)12483-7 9752834

[4] Singh M, Chakraborty A, Kumar S, Kumar A. The epidemiology of dengue viral infection in developing countries: A systematic review. J Health Res Rev. 2017;4(3):104–107. 10.4103/jhrr.jhrr_24_17

[5] World Health Organization. Dengue and severe dengue; 13 September 2018 [cited 1 February 2019]. Available from: https://www.who.int/en/news-room/fact-sheets/detail/dengue-and-severe-dengue.

[6] Halstead SB. Mosquito-borne haemorrhagic fevers of South and South-East Asia. B World Health Organ. 1966;35(1):3–15. PMC2476178 5297536

[7] Hammon WM, Rundnick A, Sather GE. Viruses Associated with Epidemic Hemorrhagic Fevers of the Philippines and Thailand. Science. 1960;131(3407):1102–1103. 10.1126/science.131.3407.1102 14399343

[8] Chareonsook O, Foy HM, Teeraratkul A, Silarug N. Changing epidemiology of dengue hemorrhagic fever in Thailand. Epidemiol Infect. 1999;122(1):161–166. 10.1017/s0950268898001617 10098800 PMC2809602

[9] Nimmannitya S, Halstead SB, Cohen SN, Margiotta MR. Dengue and Chikungunya Virus Infection in Man in Thailand, 1962–1964. Am J Trop Med Hyg. 1969;18(6):954–971. 5355242 10.4269/ajtmh.1969.18.954

[10] Stanaway JD, Shepard DS, Undurraga EA, Halasa YA, Coffeng LE, Brady OJ, et al The global burden of dengue: an analysis from the Global Burden of Disease Study 2013. Lancet Infect Dis. 2016;16(6):712–723. 10.1016/S1473-3099(16)00026-8 26874619 PMC5012511

[11] Barbazan P, Yoksan S, Gonzalez JP. Dengue hemorrhagic fever epidemiology in Thailand: description and forecasting of epidemics. Microb Infect. 2002;4(7):699–705. 10.1016/S1286-4579(02)01589-7 12067829

[12] Clark DV, Mammen MP, Nisalak A, Puthimethee V, Endy TP. Economic impact of dengue fever/dengue hemorrhagic fever in Thailand at the family and population levels. Am J Trop Med Hyg. 2005;72(6):786–791. 10.4269/ajtmh.2005.72.786 15964964

[13] Khetarpal N, Khanna I. Dengue fever: causes, complications, and vaccine strategies. J Immunol Res. 2016; 10.1155/2016/6803098 27525287 PMC4971387

[14] Naish S, Dale P, Mackenzie JS, McBride J, Mengersen K, Tong S. Climate change and dengue: a critical and systematic review of quantitative modelling approaches. BMC Infect Dis. 2014;14(1):167 10.1186/1471-2334-14-167 24669859 PMC3986908

[15] Hales S, de Wet N, Maindonald J, Woodward A. Potential effect of population and climate changes on global distribution of dengue fever: an empirical model. Lancet. 2002;360(9336):830–834. 10.1016/S0140-6736(02)09964-6 12243917

[16] Chen SC, Liao CM, Chio CP, Chou HH, You SH, Cheng YH. Lagged temperature effect with mosquito transmission potential explains dengue variability in southern Taiwan: Insights from a statistical analysis. Sci Total Environ. 2010;408(19):4069–4075. 10.1016/j.scitotenv.2010.05.021 20542536

[17] Wang Z, Chan HM, Hibberd ML, Lee GKK. Delayed Effects of Climate Variables on Incidence of Dengue in Singapore during 2000-2010. APCBEE Proc. 2012;1:22–26. 10.1016/j.apcbee.2012.03.005

[18] Choi Y, Tang CS, McIver L, Hashizume M, Chan V, Abeyasinghe RR, et al Effects of weather factors on dengue fever incidence and implications for interventions in Cambodia. BMC Public Health. 2016;16(1):241 10.1186/s12889-016-2923-2 26955944 PMC4784273

[19] Huang X, Williams G, Clements ACA, Hu W. Imported Dengue Cases, Weather Variation and Autochthonous Dengue Incidence in Cairns, Australia. PLoS One. 2013;8(12):1–7. 10.1371/journal.pone.0081887 PMC3862568 24349148

[20] Wu X, Lang L, Ma W, Song T, Kang M, He J, et al Non-linear effects of mean temperature and relative humidity on dengue incidence in Guangzhou, China. Sci Total Environ. 2018;628-629:766–771. 10.1016/j.scitotenv.2018.02.136 29454216

[21] Cheong YL, Burkart K, Leitão PJ, Lakes T. Assessing Weather Effects on Dengue Disease in Malaysia. Int J Environ Res Public Health. 2013;10(12):6319–6334. 10.3390/ijerph10126319 24287855 PMC3881116

[22] Fairos W, Azaki W, Alias L, Yap B. Modelling dengue fever (DF) and dengue haemorrhagic fever (DHF) outbreak using Poisson and Negative Binomial model. World Acad Sci Eng Technol. 2010;62:903–908.

[23] Hii YL, Zhu H, Ng N, Ng LC, Rocklöv J. Forecast of Dengue Incidence Using Temperature and Rainfall. PLoS Negl Trop Dis. 2012;6(11):1–9. 10.1371/journal.pntd.0001908 PMC3510154 23209852

[24] Hii YL. Climate and dengue fever: early warning based on temperature and rainfall [Doctoral Thesis]. Department of Public Health and Clinical Medicine, Faculty of Medicine, Umeå University; 2013.

[25] Hii YL, Rocklöv J, Ng N, Tang CS, Pang FY, Sauerborn R. Climate variability and increase in intensity and magnitude of dengue incidence in Singapore. Glob Health Action. 2009;2(1):2036 10.3402/gha.v2i0.2036 PMC2799326 20052380

[26] Lee HS, Nguyen-Viet H, Nam VS, Lee M, Won S, Duc PP, et al Seasonal patterns of dengue fever and associated climate factors in 4 provinces in Vietnam from 1994 to 2013. BMC Infect Dis. 2017;17(1):218 10.1186/s12879-017-2326-8 28320341 PMC5359841

[27] Li C, Wang X, Wu X, Liu J, Ji D, Du J. Modeling and projection of dengue fever cases in Guangzhou based on variation of weather factors. Sci Total Environ. 2017;605-606:867–873. 10.1016/j.scitotenv.2017.06.181 28683431

[28] Lu L, Lin H, Tian L, Yang W, Sun J, Liu Q. Time series analysis of dengue fever and weather in Guangzhou, China. BMC Public Health. 2009;9(1):395 10.1186/1471-2458-9-395 19860867 PMC2771015

[29] Wongkoon S, Jaroensutasinee M, Jaroensutasinee K. Climatic variability and dengue virus transmission in Chiang Rai, Thailand. Biomedica. 2011;27(19):5–13.

[30] Wu PC, Guo HR, Lung SC, Lin CY, Su HJ. Weather as an effective predictor for occurrence of dengue fever in Taiwan. Acta Trop. 2007;103(1):50–57. 10.1016/j.actatropica.2007.05.014. 17612499

[31] Beck HE, Zimmermann NE, McVicar TR, Vergopolan N, Berg A, Wood EF. Present and future Köppen-Geiger climate classification maps at 1-km resolution. Sci Data. 2018;5. 10.1038/sdata.2018.214 PMC6207062 30375988

[32] Arcari P, Tapper N, Pfueller S. Regional variability in relationships between climate and dengue/DHF in Indonesia. Singap J Trop Geogr. 2007;28(3):251–272. 10.1111/j.1467-9493.2007.00300.x

[33] Beretta E, Hara T, Ma W, Takeuchi Y. Global asymptotic stability of an SIR epidemic model with distributed time delay. Nonlinear analysis, theory, methods & applications. 2001;47(6):4107–4115. 10.1016/S0362-546X(01)00528-4

[34] Sharmin S, Glass K, Viennet E, Harley D. Interaction of Mean Temperature and Daily Fluctuation Influences Dengue Incidence in Dhaka, Bangladesh. PLOS Negl Trop Dis. 2015;9(7):1–13. 10.1371/journal.pntd.0003901 PMC4498835 26161895

[35] Ehelepola NDB, Ariyaratne K. The correlation between dengue incidence and diurnal ranges of temperature of Colombo district, Sri Lanka 2005-2014. Glob Health Action. 2016;9(32267). 10.3402/gha.v9.32267 PMC5002035 27566717

[36] Chu PC, Qi Y, Chen Y, Shi P, Mao Q. South China Sea Wind-Wave Characteristics. Part I: Validation of Wavewatch-III Using TOPEX/Poseidon Data. Journal of Atmospheric and Oceanic Technology. 2004;21(11):1718–1733. 10.1175/JTECH1661.1

[37] Meteorological Department of Thailand. The climate of Thailand; 2015 [cited 1 February 2019]. Available from: https://www.tmd.go.th/en/archive/thailand_climate.pdf.

