# Supplementary material for: The association between dengue incidences and provincial-level weather variables in Thailand from 2001 to 2014
Source: PLoS One. 2019 Dec 26;14(12):e0226945. doi: 10.1371/journal.pone.0226945 (PMC6932763; doi:10.1371/journal.pone.0226945)
Supplement: S1 Appendix — (PDF) [file pone.0226945.s001.pdf]

S1 Appendix: Complete Provincial Level Analysis of Weather Variables and Dengue Cases

The following are the detailed results of our experiment at the provincial level, alphabetically ordered.

Bangkok

Bangkok is the capital city of Thailand, located at coordinate 13°45′09″N 100°29′39″E in the central region. The city covers an area of 1,568.737 km^2. Density of population is around 5,300 people per km^2. Weather in Bangkok has tropical savanna climate under the South Asian monsoon system. Temperatures are perennially warm in range from the low of 22°C (71.6°F) in December to the high of 35.4 °C (95.7°F) in April. Rainy season begins with the arrival of the southwest monsoon around mid-May, and lasts until mid-October. The averaged rainfall is generally high in September approximately 334.3 mm. Relative humidity is high in rainy season from May-September, meanwhile relatively low in hot and cold season from March-May and December-February respectively.

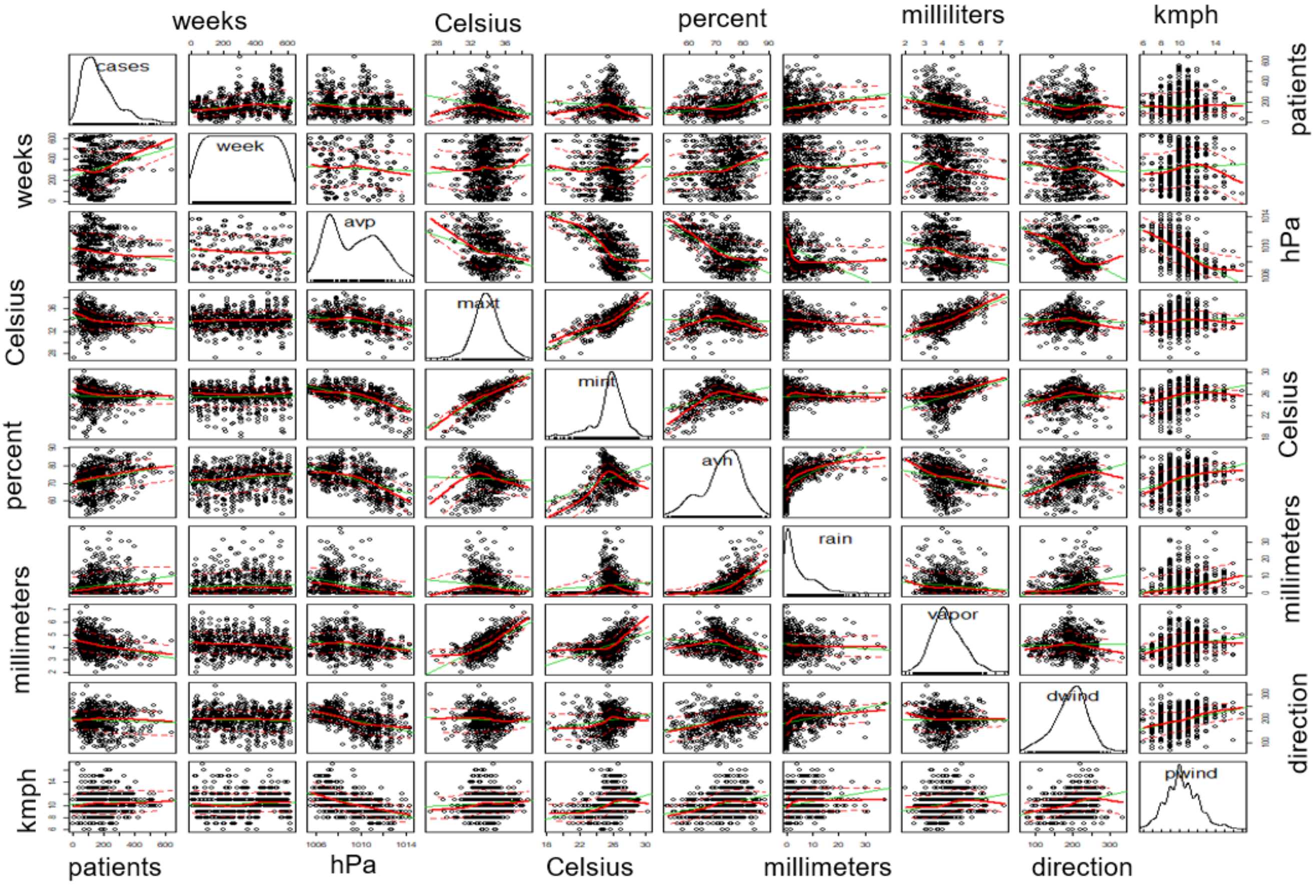

Figure 1: Scatter plot between dengue cases (cases) and selected independent variables, which are the weekly period starting from January 2001 – December 2013 (week), average pressure (avp), maximum temperature (maxt), minimum temperature (mint), average humidity (avh), precipitation (rain), vaporization of water (vapor), wind direction (dwind), and wind power (pwind). The plot visualizes pairwise hundred relationships of training set in Bangkok.

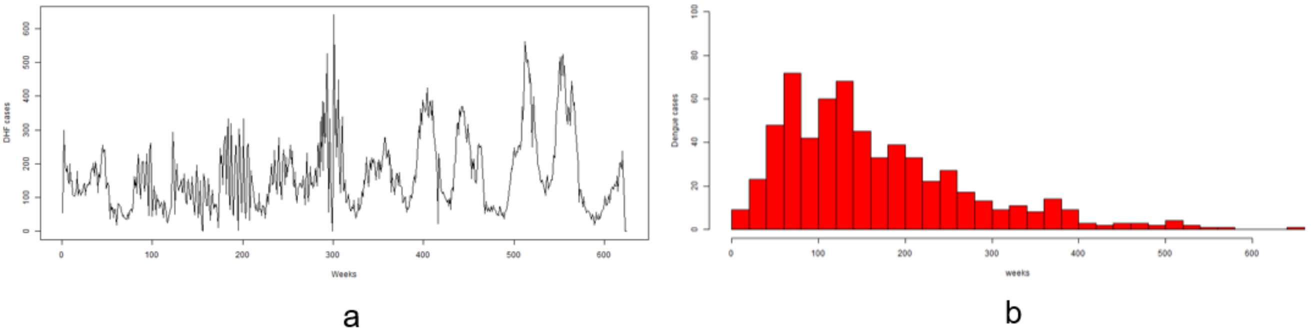

Figure 2: (a) Line plot between dengue incidences and weeks, the plot shows trends of dengue incidences in each year as stationary time series. b) Histogram of dengue incidences in Bangkok starting from January 2001 to December 2013 (624 weeks).

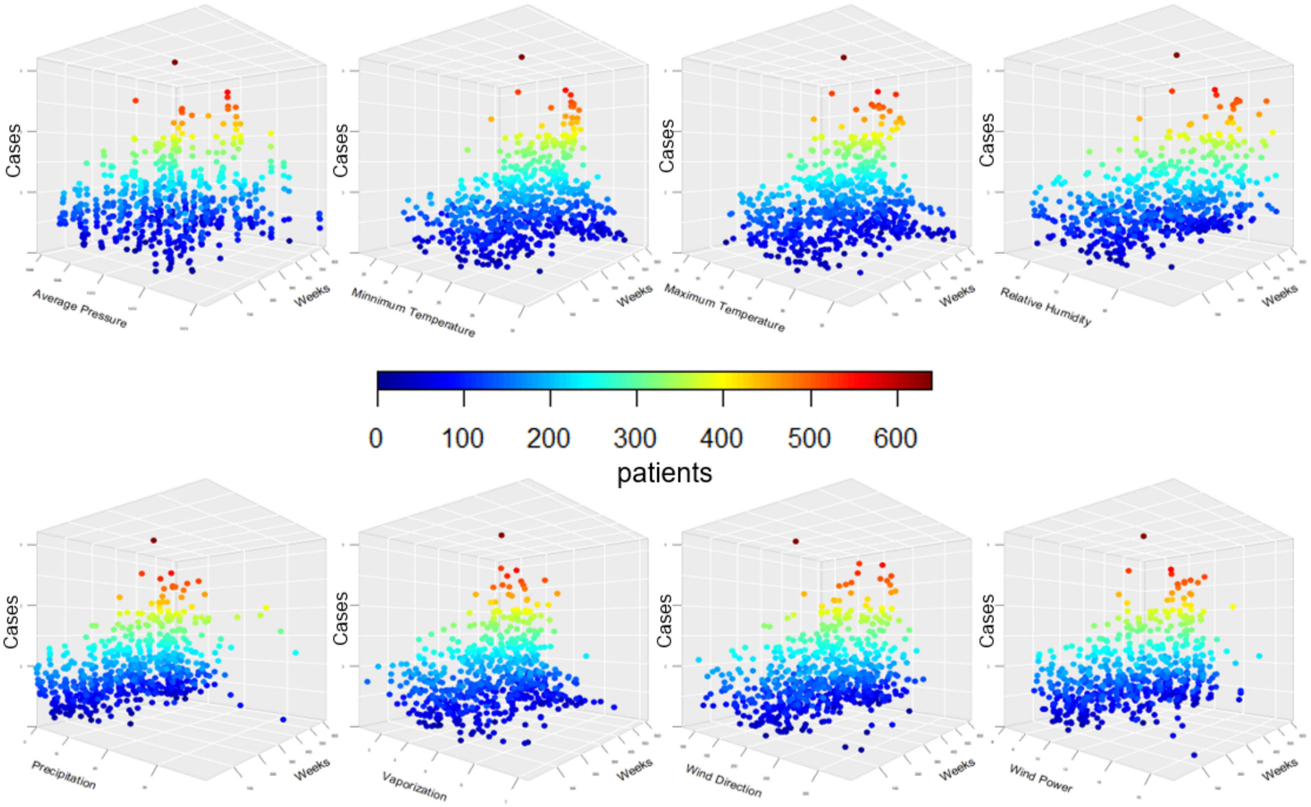

Figure 3: Three-dimensional scatter plot between dengue incidences and weather effects starting from January 2001 to December 2013 of Bangkok.

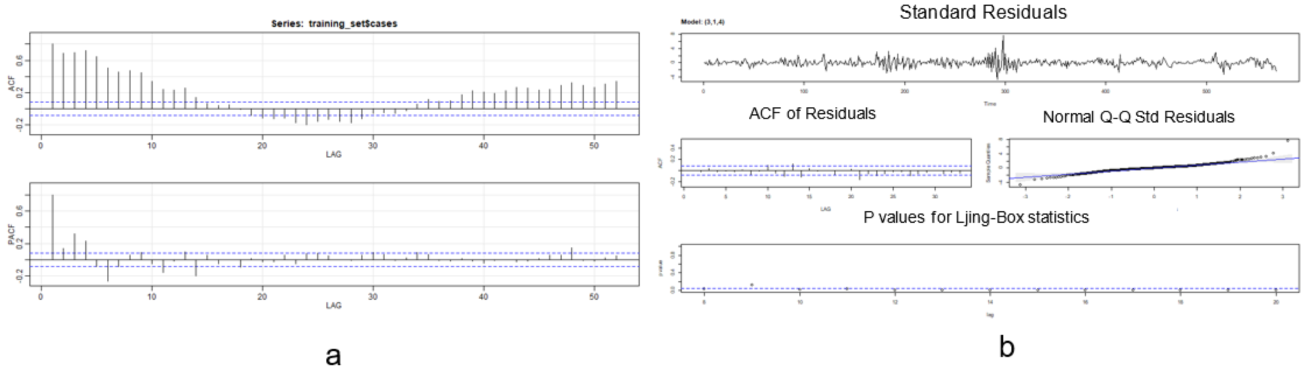

Figure 4: (a) Two plots between lag-time of dengue incidences and ACF and PACF relationship calculated from ARIMA model (b) Summary plots of time series analysis, multiple plots include the plot of predicted model over the time, the plot of ACF residual over lag-time of dengue incidences, residual Q-Q plot of standard residual, and p-value for Ljung-Box statistics of PACF relationship in Bangkok over the training data starting from January 2001 to December 2013.

The best model of Bangkok is based on quasi-likelihood method. The correlation coefficient on the test set in 2014 is 0.724 (95%CI: 0.6746, 0.8349). The best model of Bangkok uses 8 variables. The most significant variables are 1-week-lag cases, following by 3-week-lag precipitation, 2-week-lag cases, current week minimum temperature and 2-week-lag maximum temperature. Other variables which have less significant are, current week precipitation, wind direction and wind power. Time series methods by ARIMA and SARIMA yield the correlation coeffect of 0.288 and unpredictable respectively.

Table 1: Comparison table of all methods by the highest correlation coefficient (R^2) and the lowest prediction error (RMSE) in Bangkok.

**Methods**	**R-squared (R^2)**	**Root mean square error (RMSE)**
Poisson Regression	0.41839	40.7271
Negative Binomial Regression	0.51934	37.0241
Quasi-likelihood Regression	0.72414	28.0486
ARIMA (3,1,4)	0.28762	60.5988
SARIMA (2,0,1)(0,2,0)$_{52}$	-13.59762	204.037

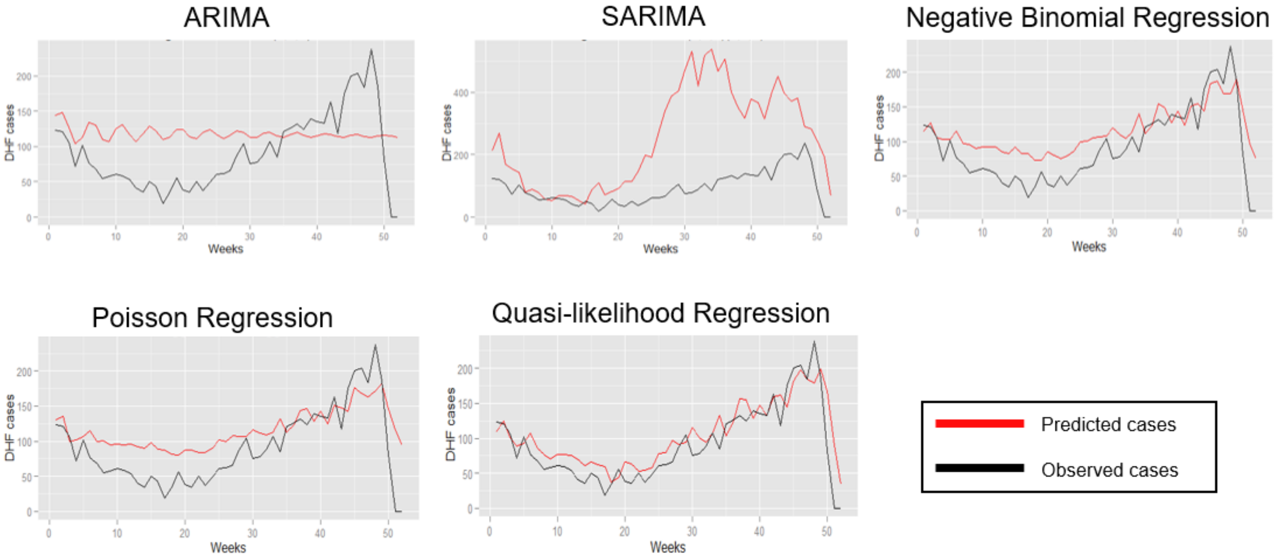

Figure 5: Plots between dengue cases and weeks, the black line represents the observed dengue cases, and the red line represents the predicted dengue cases of the best fit model of each technique over the test set data starting from January 2014 to December 2014.

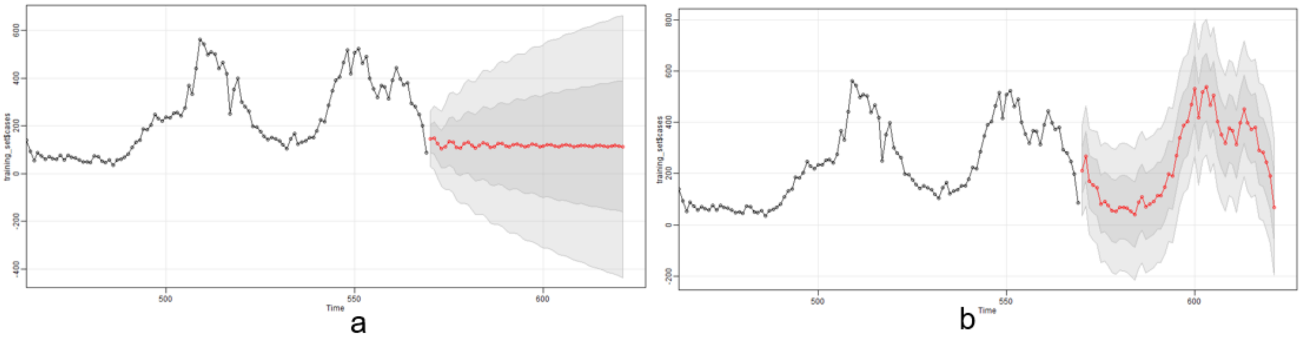

Figure 6: (a) Plot between dengue incidences over weekly time by the best model of ARIMA and (b) SARIMA time series analysis, the black line represents training set data starting from January 2012 to December 2013, and the red line represents the forecasted dengue incidences from January 2014 to December 2014.

Table 2: Coefficients and significant values of best fit GLM models, Negative Binomial, Poisson and Quasi-likelihood regression model of Bangkok. The table summarizes coefficients of each independent variables which are composed in best fit model of each method. The significant of each variable is labelled by asterisks under the coefficients. The most important factor is marked as three asterisks which p-value ranges from 0 to 0.001. The second important factor is marked as two asterisks which p-value ranges from 0.001 to 0.01. The third important factor is marked as an asterisk which p-value ranges from 0.01 to 0.1. The least important is also marked as a dot which p-value ranges from 0.1 to 1.

**Independent variables**	**Lag**	**Coefficients/Significant**		
		NB	Poisson	Quasi
Intercept		4.97363 ***	4.4519 ***	58.4065
Cases	1	0.00389 ***	0.002659 ***	0.66804 ***
	2			0.12172 **
	3		0.001173 ***	
Average Pressure	0			
	1			
	2			
	3			
Minimum Temperature	0	0.01888	0.02379 ***	3.33458 .
	1			
	2			
	3			
Maximum Temperature	0			
	1			
	2	-0.02793 *	-0.01970 ***	-3.62766 .
	3			
Relative Humidity	0			
	1			
	2			
	3			
Precipitation	0			0.34435
	1			
	2			
	3	0.01523 ***	0.01330 ***	2.16393 ***
Vaporization	0	-0.0525 .	-0.03321 ***	
	1			
	2			
	3			
Wind Direction	0			
	1			
	2			-0.01529
	3			
Wind Power	0			
	1			
	2			0.56522
	3			

Buriram

Buriram locates in northeastern area of Thailand at 14°59′39″N 103°06′08″E. The city covers an area of 10,323 km^2. Total population in Buri Ram is approximately 27,150 people in 2018. Density of population is 154.2 per km^2. Temperatures in Buriram are generally low from 17.65 to 18.30 °C in December to January. The highest temperature is 36.45°C (97.7°F) in April. Rainy season begins with the arrival of the southwest monsoon around mid-May. The average precipitation around the year is 1,358.27 mm. Relative humidity is high in rainy season from May-September.

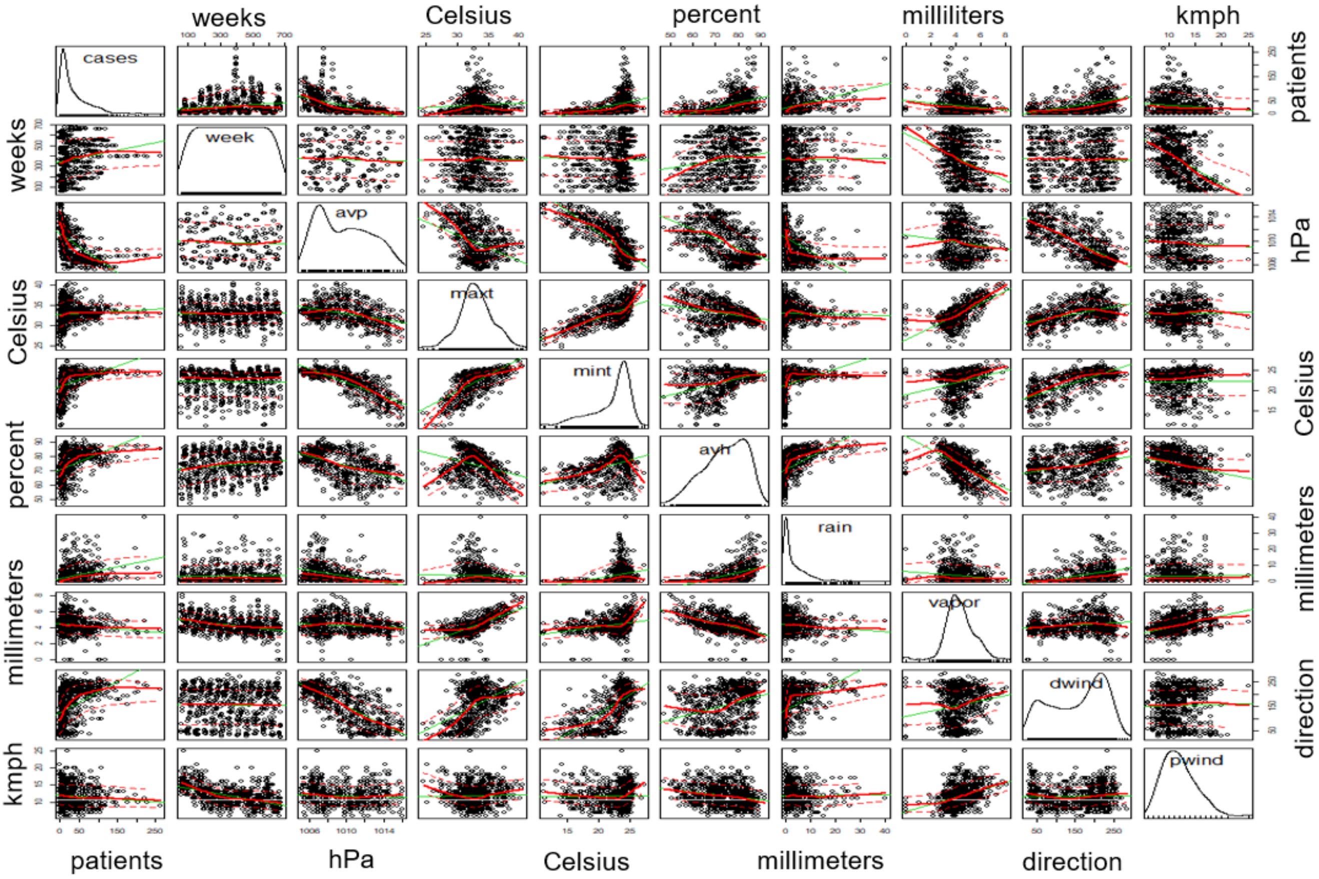

Figure 7: Scatter plot between dengue cases (cases) and selected independent variables, which are the weekly period starting from January 2001 – December 2013 (week), average pressure (avp), maximum temperature (maxt), minimum temperature (mint), average humidity (avh), precipitation (rain), vaporization of water (vapor), wind direction (dwind), and wind power (pwind). The plot visualizes pairwise hundred relationships of training set in Buriram.

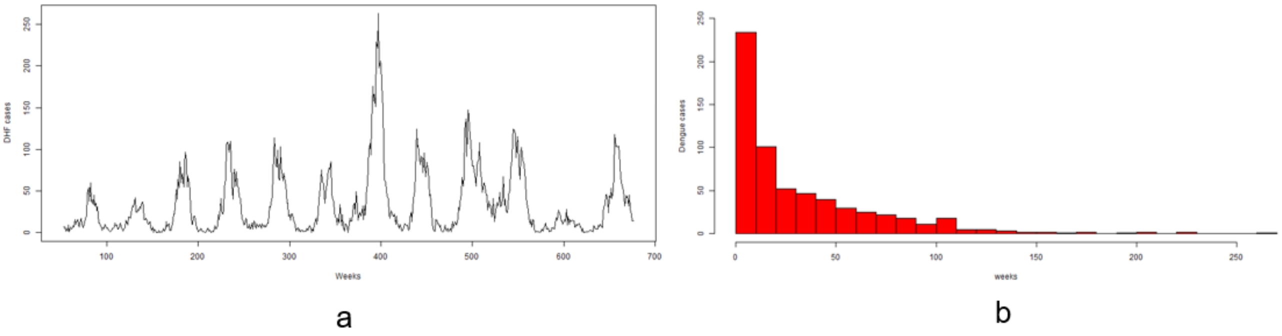

Figure 8: (a) Line plot between dengue incidences and weeks, the plot shows the trends of dengue incidences in each year as stationary time series. (b) Histogram of dengue incidences in Buriram starting from January 2001 to December 2013 (624 weeks).

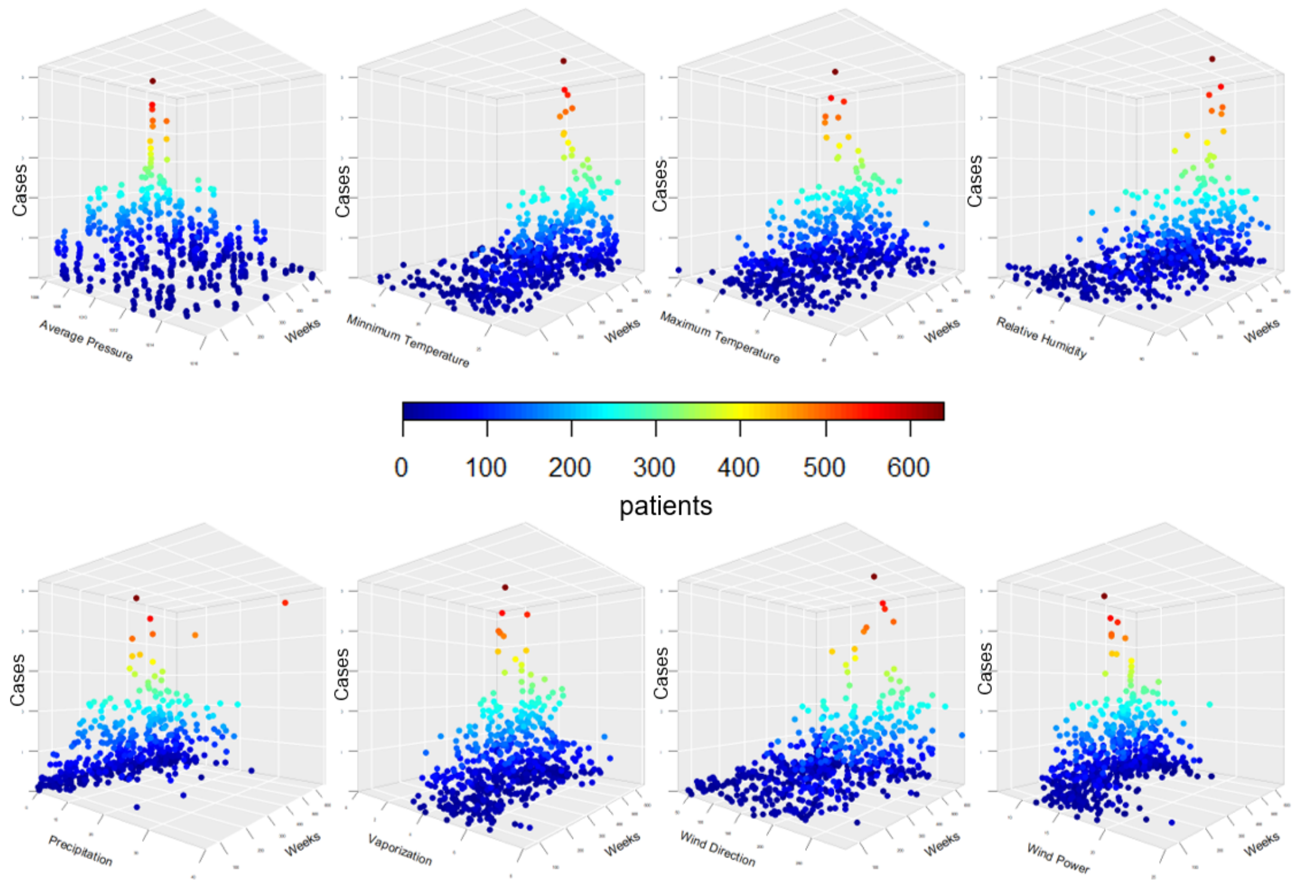

Figure 9: Three-dimensional scatter plot between dengue incidences and weather effects starting from January 2001 to December 2013 of Buriram.

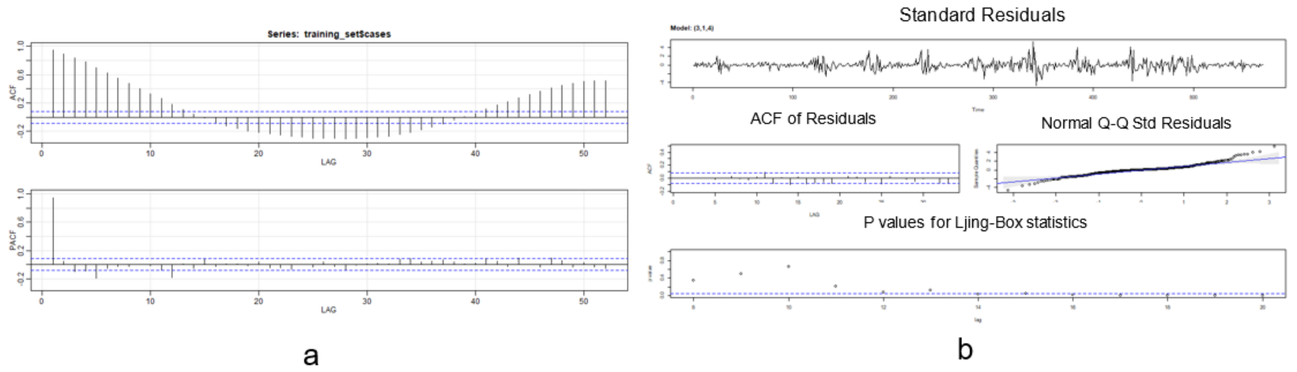

Figure 10: (a) Two plots between lag-time of dengue incidences and ACF and PACF relationship calculated from ARIMA model (b) Summary plots of time series analysis, multiple plots include the plot of predicted model over the time, the plot of ACF residual over lag-time of dengue incidence, residual Q-Q plot of standard residual, and p-value for Ljung-Box statistics of PACF relationship in Buriram over the training data starting from January 2001 to December 2013.

For Buriram, the best model is based on quasi-likelihood regression model which presents the correlation coefficient of 0.896 (95%CI: 0.7505, 0.9330). The model consists of 8 variables. The most significant variable is 1-week-lag cases, following by 1-week-lag maximum temperature, current week precipitation. Other variables are 2-week-lag average pressure, 2-week-lag maximum temperature, 3-week-lag maximum temperature and 1-week-lag relative humidity. Time series methods by ARIMA and SARIMA yield unpredictable results.

Table 3: Comparison table of all methods by the highest correlation coefficient (R^2) and the lowest prediction error (RMSE) in Buriram.

Methods	R-squared (R^2)	Root mean square error (RMSE)
Poisson Regression	0.8442167	12.52911
Negative Binomial Regression	0.8173055	13.56821
Quasi-likelihood Regression	0.8957245	10.25064
ARIMA (3,1,4)	-1.109725	46.10761
SARIMA $(2,0,1)(0,2,0)_{52}$	-0.5491684	39.51018

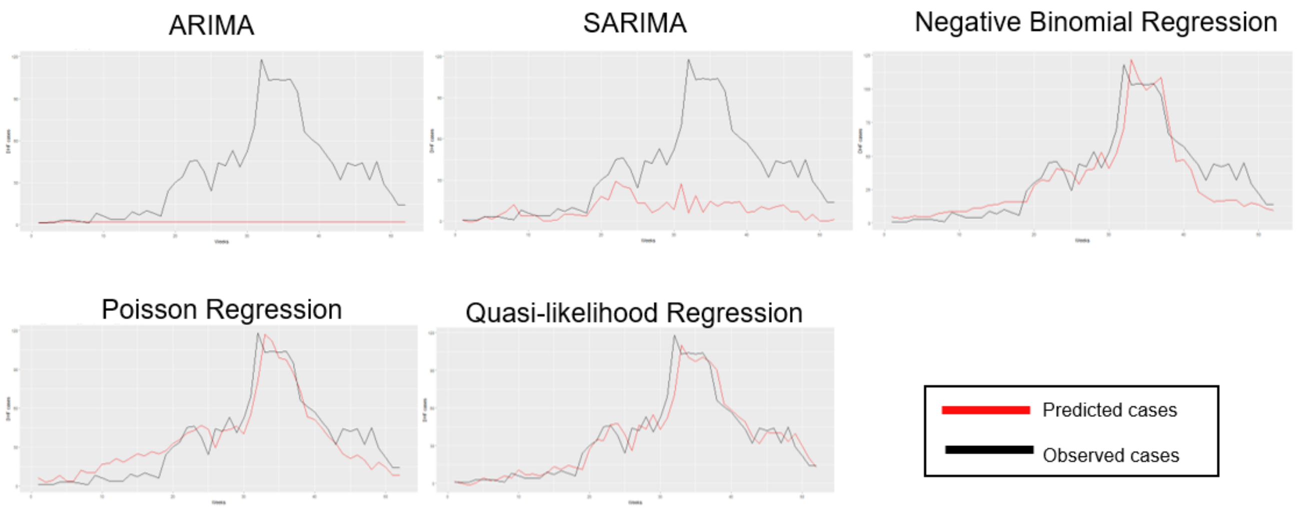

Figure 11: Plots between dengue cases and weeks, the black line represents the observed dengue cases, and the red line represents the predicted dengue cases of the best fit model of each technique over the test set data starting from January 2014 to December 2014.

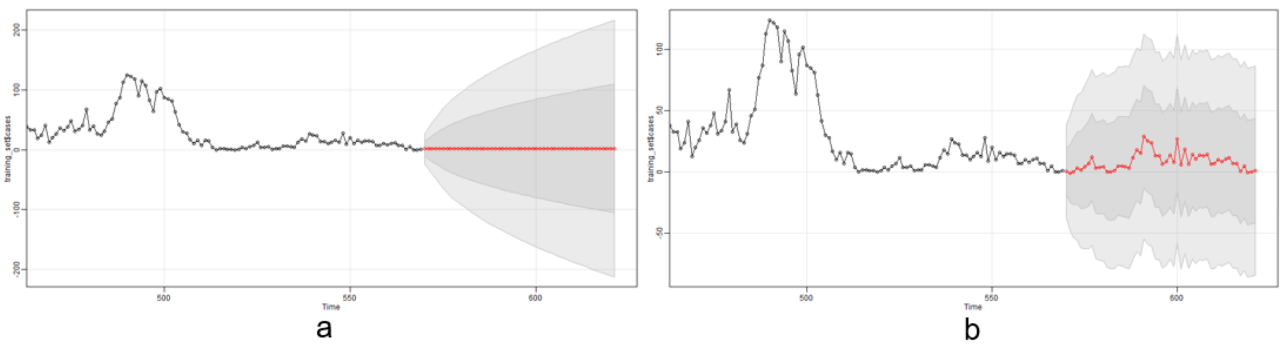

Figure 12: (a) Plot between dengue incidences over weekly time by the best model of ARIMA and (b) SARIMA time series analysis, the black line represents training set data starting from January 2012 to December 2013, and the red line represents the forecasted dengue incidences from January 2014 to December 2014.

Table 4: Coefficients and significant values of best fit GLM models, Negative Binomial, Poisson and Quasi-likelihood regression model of Buriram. The table summarizes coefficients of each independent variables which are composed in best fit model of each method. The significant of each variable is labelled by asterisks under the coefficients. The most important factor is marked as three asterisks which p-value ranges from 0 to 0.001. The second important factor is marked as two asterisks which p-value ranges from 0.001 to 0.01. The third important factor is marked as an asterisk which p-value ranges from 0.01 to 0.1. The least important is also marked as a dot which p-value ranges from 0.1 to 1.

**Independent variables**	**Lag**	**Coefficients/Significant**		
		NB	Poisson	Quasi
Intercept		123.5 ***	-1.481205 ***	544.36610
Cases	1	0.01738 ***	0.0116863 ***	0.92231 ***
	2			
	3			
Average Pressure	0			
	1			
	2	-0.1212 ***		-0.56945
	3			
Minimum Temperature	0	0.05907 ***	0.1275689 ***	
	1			
	2			
	3		0.099995 ***	-0.39707
Maximum Temperature	0			
	1			0.79318 *
	2		-0.0151662 **	-0.46524
	3			0.67908
Relative Humidity	0			
	1	0.003542		0.12810
	2			
	3			
Precipitation	0	-0.005301	-0.005314 ***	-0.19568 .
	1			
	2			
	3			
Vaporization	0		-0.0497328 ***	
	1			
	2			
	3		-0.0272550 *	
Wind Direction	0			
	1			
	2			
	3			
Wind Power	0			
	1			
	2			
	3	-0.01896 *	-0.0037083	

Chachoengsao

Chachoengsao is located in south-central region of Thailand at 13°41′25″N 101°04′13″E. Chachoengsao covers an area of 5,351 km^2. Total populations in Chachoengsao are 709,889 people in 2017. The density of population is 132.7 per km^2. Chachoengsao's weather behaves as tropical savanna climate under the South Asian monsoon system. Temperature is high in April around 29.7 °C. Low temperature of 25.7°C presents in December. The rainy season begins with the arrival of the southwest monsoon around mid-May. September shows the highest average rainfall. Relative humidity is high in rainy season from May-September. Other factors present the relation as shown in 13 below.

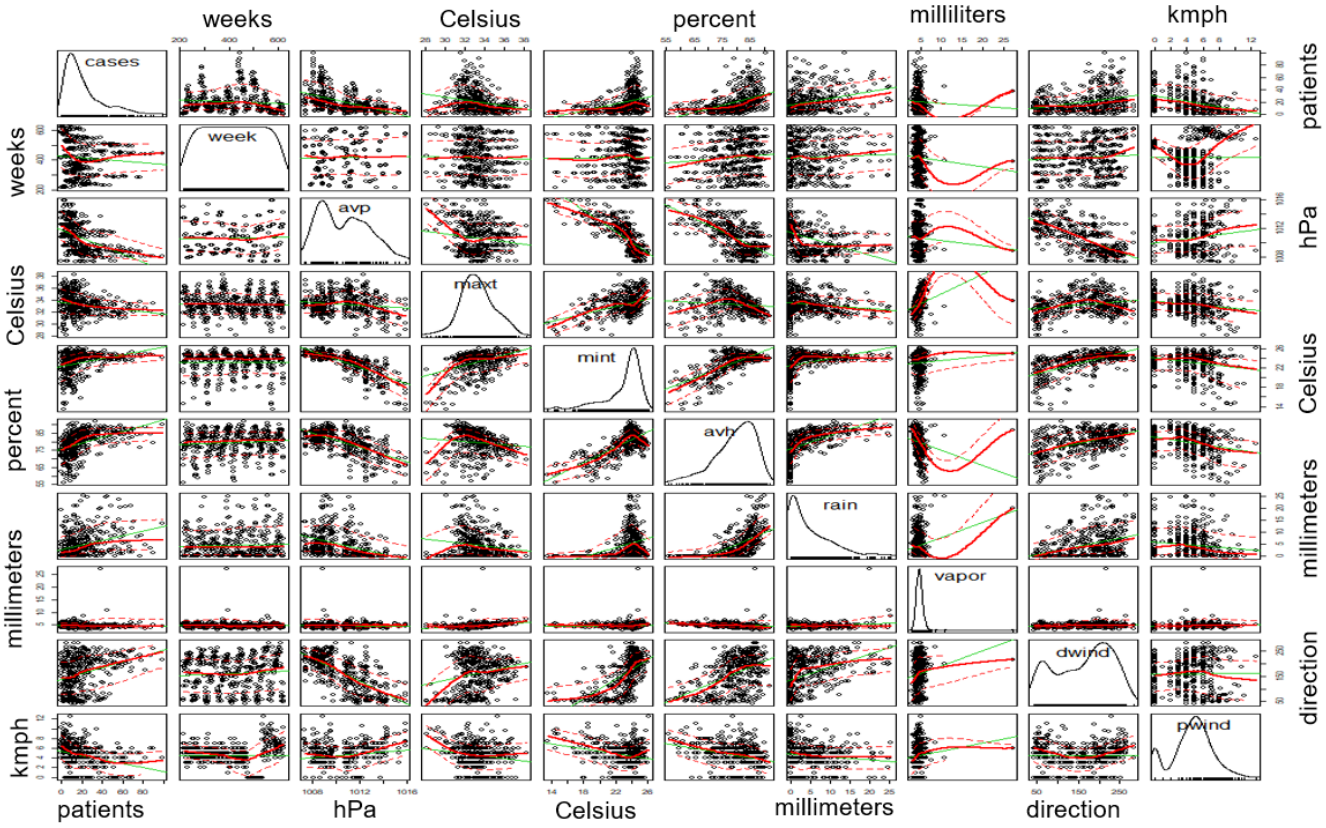

Figure 13: Scatter plot between dengue cases (cases) and selected independent variables, which are the weekly period starting from January 2001 – December 2013 (week), average pressure (avp), maximum temperature (maxt), minimum temperature (mint), average humidity (avh), precipitation (rain), vaporization of water (vapor), wind direction (dwind), and wind power (pwind). The plot visualizes pairwise hundred relationships of training set in Chachoengsao.

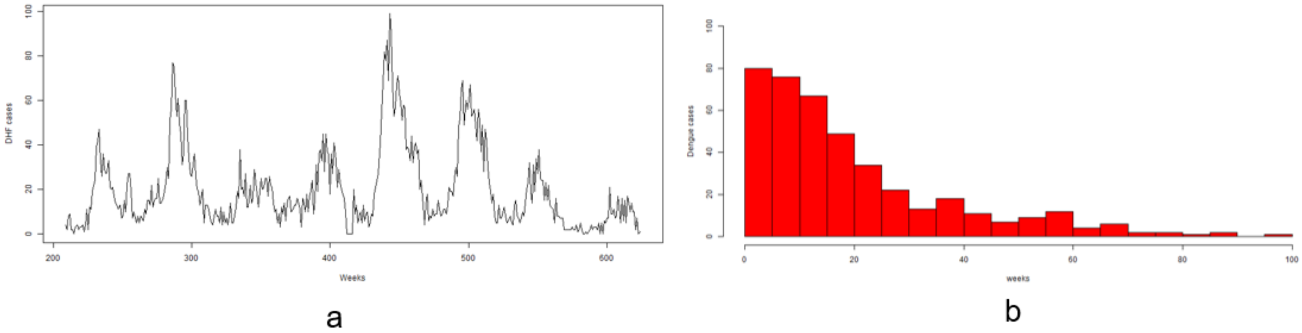

Figure 14: (a) Line plot between dengue incidences and weeks, the plot shows trends of dengue incidences in each year as stationary time series. (b) Histogram of dengue incidences in Chachoengsao starting from January 2001 to December 2013 (624 weeks).

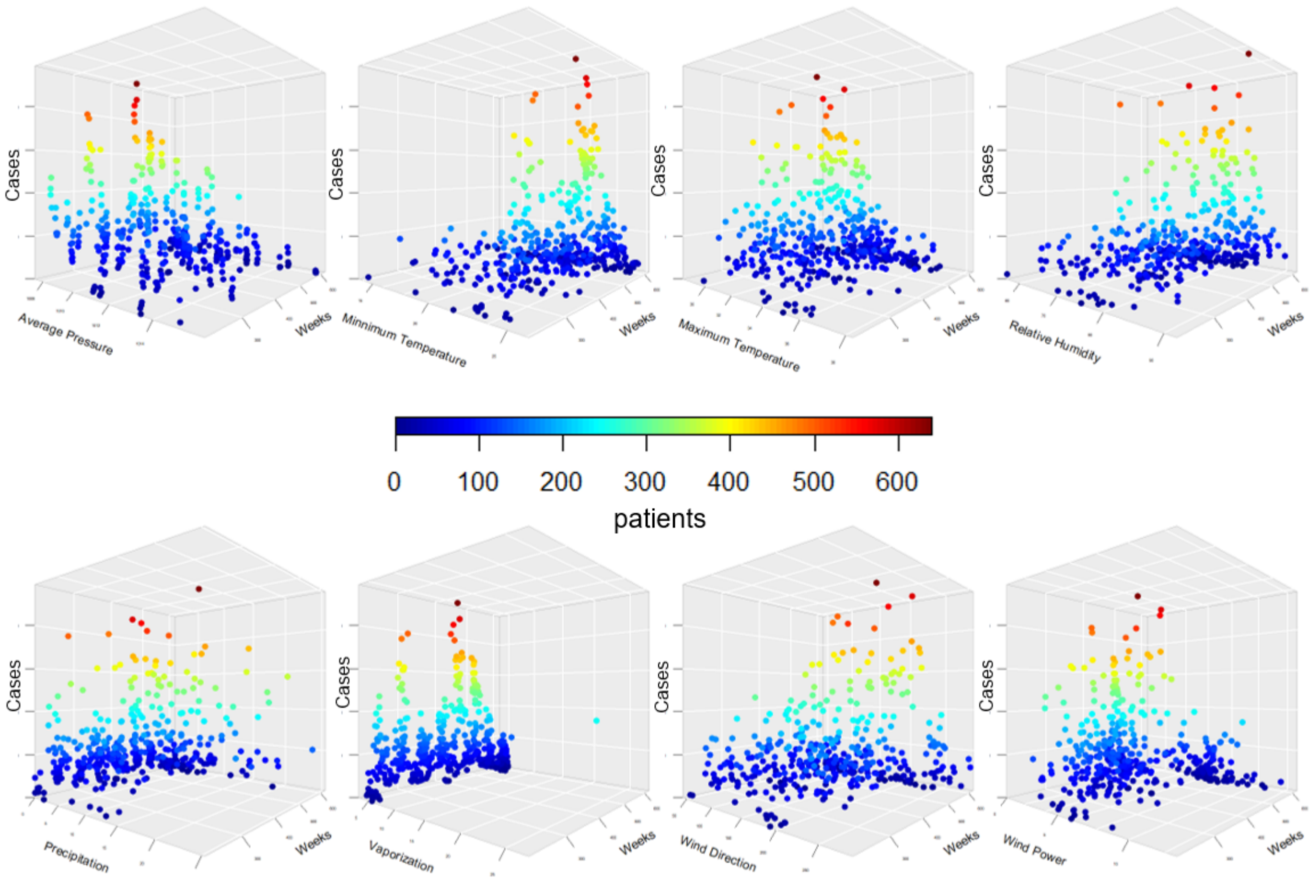

Figure 15: Three-dimensional scatter plot between dengue incidences and weather effects starting from January 2001 to December 2013 of Chachoengsao.

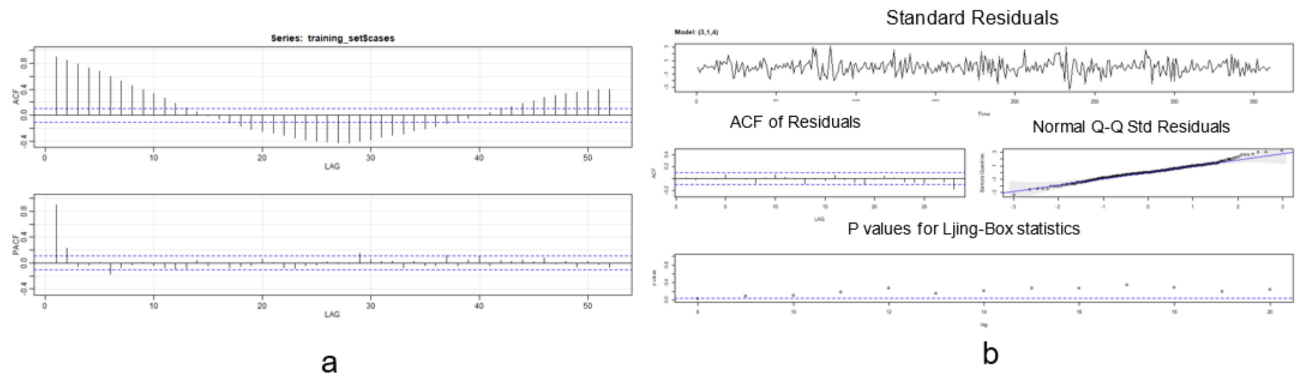

Figure 16: (a) Two plots between lag-time of dengue incidences and ACF and PACF relationship calculated from ARIMA model (b) Summary plots of time series analysis, multiple plots include the plot of predicted model over the time, the plot of ACF residual over lag-time of dengue incidences, residual Q-Q plot of standard residual, and p-value for Ljung-Box statistics of PACF relationship in Chachoengsao over the training data starting from January 2001 to December 2013.

For Chachoengsao, the best model is based on quasi-likelihood method. Total nine variables are comprised in the best model. The most significant variable is 2-week-lag cases, following by 3-week-lag cases, current week vaporization, current week precipitation. The correlation coefficient on the test set in 2014 is 0.391 (95%CI: 0.0154, 0.7646). Other variables which have less significant consist of 3-week-lag minimum temperature and current week, 1-week-lag, 3-week-lag of wind power. Time series methods by ARIMA and SARIMA yield the unpredictable values.

Table 5: Comparison table of all methods by the highest correlation coefficient (R^2) and the lowest prediction error (RMSE) in Chachoengsao.

**Methods**	**R-squared (R^2)**	**Root mean square error (RMSE)**
Poisson Regression	-0.2093143	6.223512
Negative Binomial Regression	-0.4203013	5.742683
Quasi-likelihood Regression	0.3915074	4.073549
ARIMA (3,1,4)	-1.121021	7.605325
SARIMA $(2,0,1)(0,2,0)_{52}$	-3.226851	10.73628

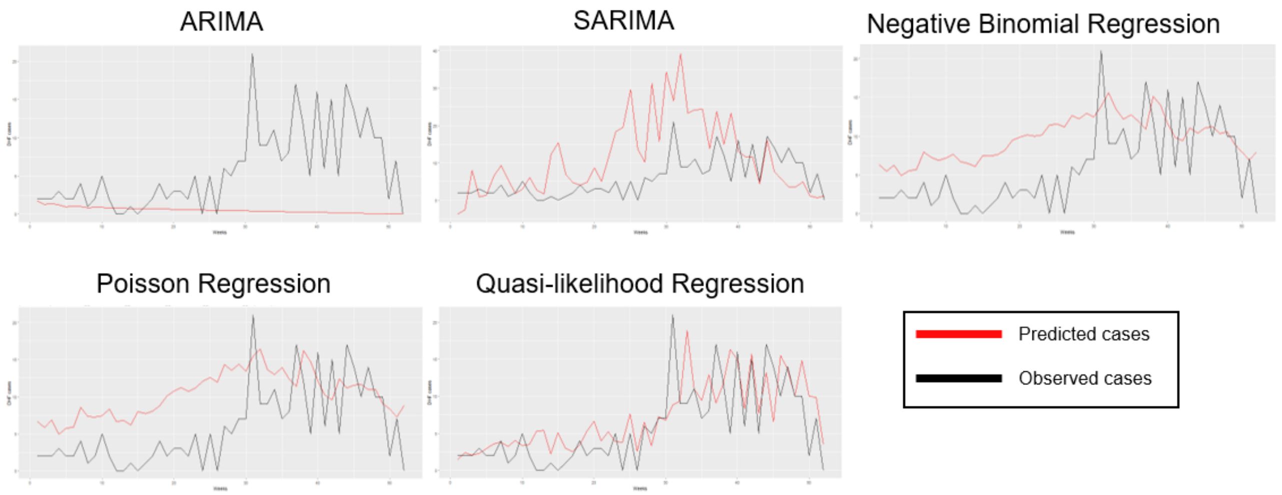

Figure 17: Plots between dengue cases and weeks, the black line represents the observed dengue cases, and the red line represents the predicted dengue cases of the best fit model of each technique over the test set data starting from January 2014 to December 2014.

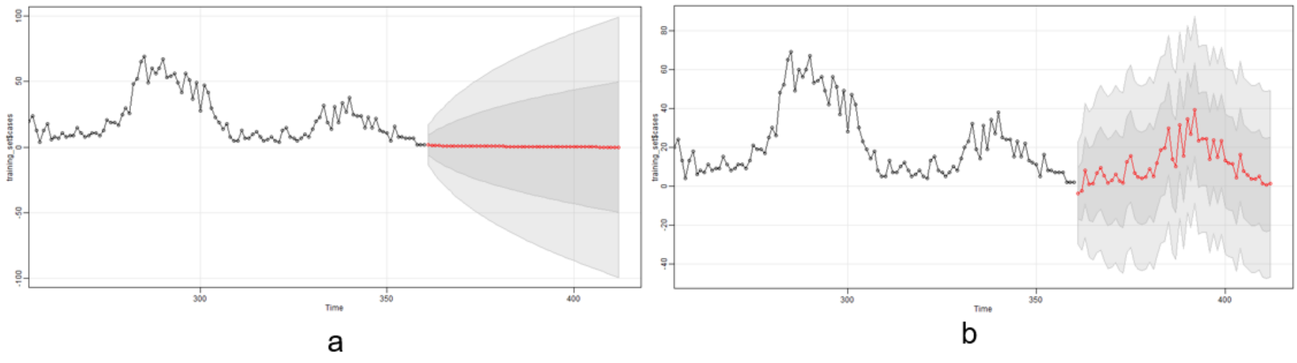

Figure 18: (a) Plot between dengue incidences over weekly time by the best model of ARIMA and (b) SARIMA time series analysis, the black line represents training set data starting from January 2012 to December 2013, and the red line represents the forecasted dengue incidences from January 2014 to December 2014.

Table 6: Coefficients and significant values of best fit GLM models, Negative Binomial, Poisson and Quasi-likelihood regression model of Chachoengsao. The table summarizes coefficients of each independent variables which are composed in best fit model of each method. The significant of each variable is labelled by asterisks under the coefficients. The most important factor is marked as three asterisks which p-value ranges from 0 to 0.001. The second important factor is marked as two asterisks which p-value ranges from 0.001 to 0.01. The third important factor is marked as an asterisk which p-value ranges from 0.01 to 0.1. The least important is also marked as a dot which p-value ranges from 0.1 to 1.

**Independent variables**	**Lag**	**Coefficients/Significant**		
		NB	Poisson	Quasi
Intercept		120.0<br>***	136.3<br>***	1.536364
Cases	1	0.01907<br>***	0.01703<br>***	
	2	0.009617<br>***	0.006580<br>***	0.726566<br>***
	3			0.124185<br>*
Average Pressure	0	-0.1150<br>***	-0.1304<br>***	
	1			
	2			
	3			
Minimum Temperature	0			
	1			
	2			
	3			-0.021501
Maximum Temperature	0			
	1			
	2	-0.04029<br>**	-0.05512<br>***	
	3			
Relative Humidity	0			
	1			
	2			
	3			
Precipitation	0			0.167374<br>.
	1			
	2			
	3			
Vaporization	0			0.744763<br>*
	1			
	2			
	3		-0.0272550<br>*	
Wind Direction	0			
	1	0.0005471	-0.0004224	
	2			
	3			
Wind Power	0		-0.0004018	-0.280000
	1			-0.009942
	2			
	3	-0.02441<br>**	-0.03417<br>***	-0.149007

Chai Nat

Chai Nat is located in the central region of Thailand at 15°11′14″N 100°07′42″ E. Chai Nat covers an area of 2,470 km^2. The density of population is 130 per km^2. Total populations in Chai Nat are 332,283 people. Temperature is in the range from the low of 8.3 to 12.2 ° C in December to February. High temperature of 41.4° C presents in April. July is the wettest month, with rainfall of around 87.7 mm. Relative humidity is high in rainy season from May-September. The rainy season begins with the arrival of the southwest monsoon around mid-May.

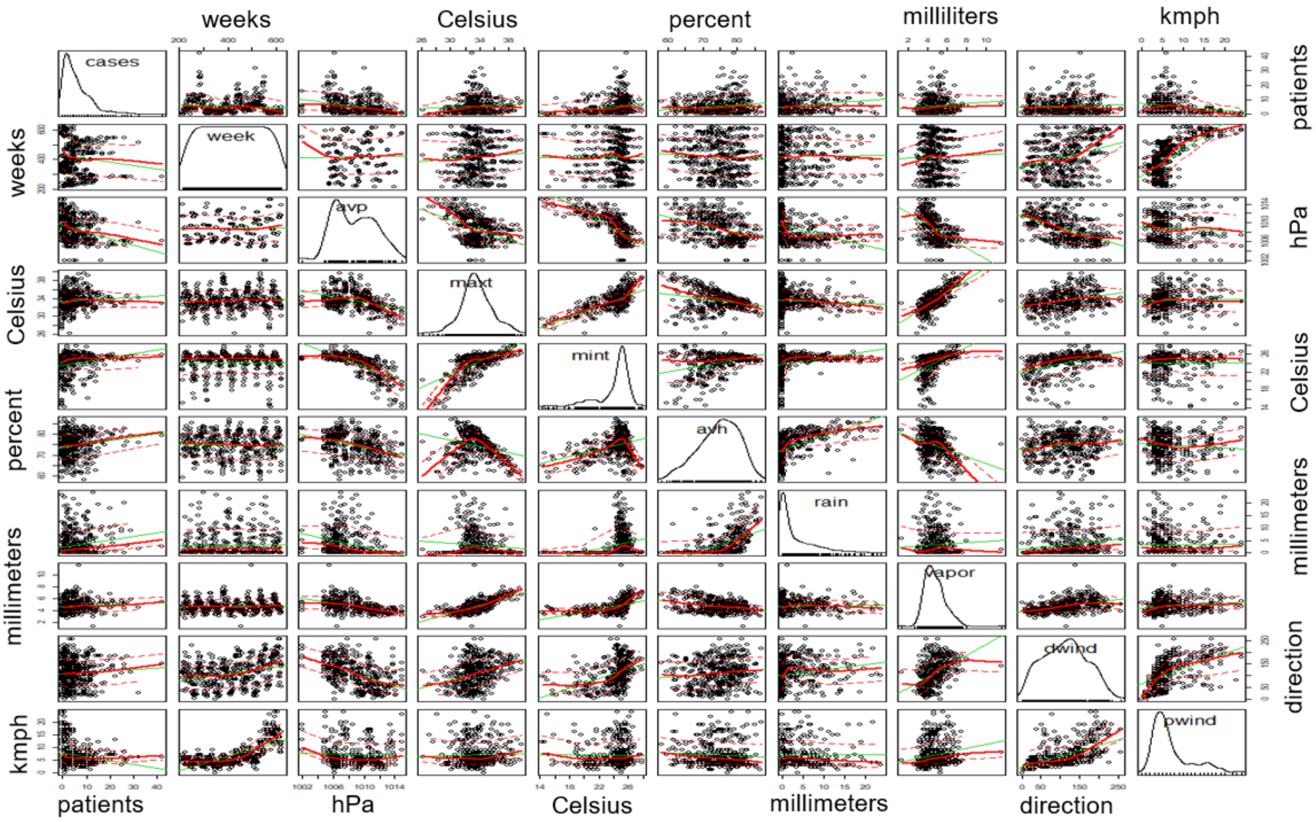

Figure 19: Scatter plot between dengue cases (cases) and selected independent variables, which are the weekly period starting from January 2001 – December 2013 (week), average pressure (avp), maximum temperature (maxt), minimum temperature (mint), average humidity (avh), precipitation (rain), vaporization of water (vapor), wind direction (dwind), and wind power (pwind). The plot visualizes pairwise hundred relationships of training set in Chai Nat.

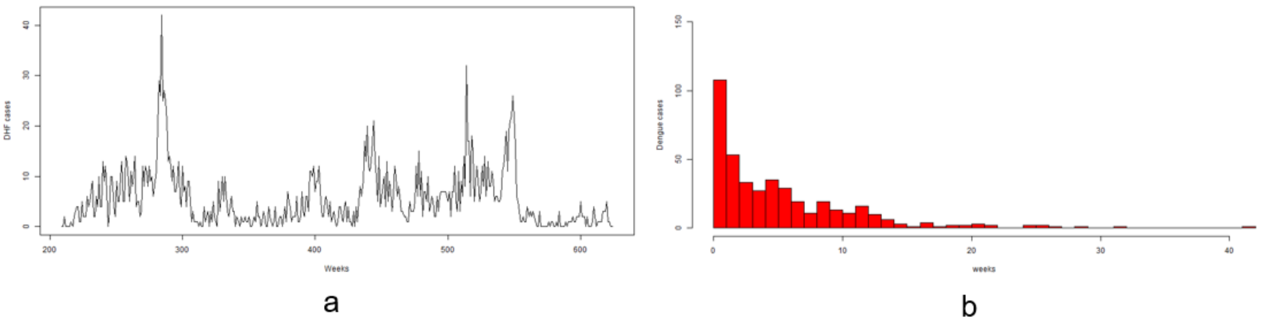

Figure 20: (a) Line plot between dengue incidences and weeks, the plot shows trends of dengue incidences in each year as stationary time series. (b) Histogram of dengue incidences in Chai Nat starting from January 2001 to December 2013 (624 weeks).

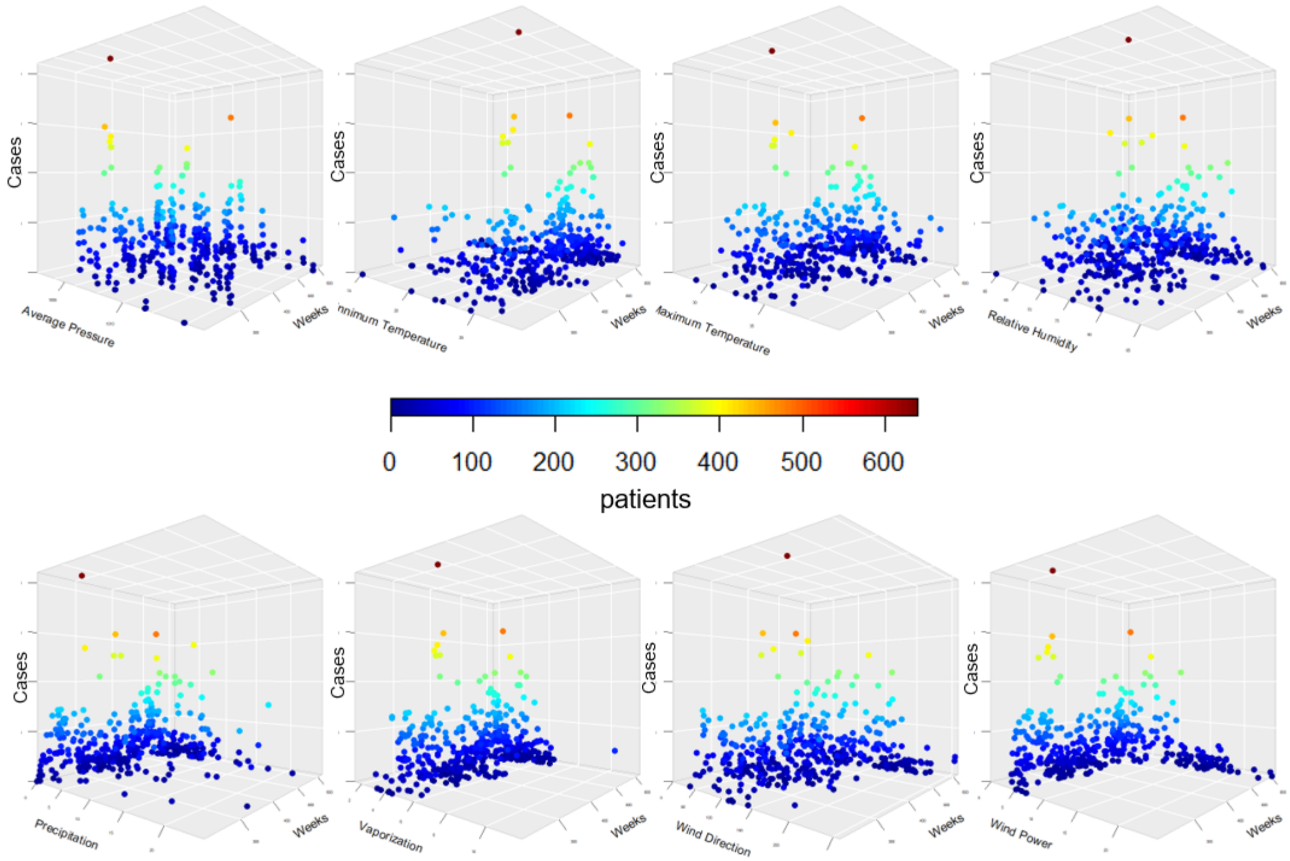

Figure 21: Three-dimensional scatter plot between dengue incidences and weather effects starting from January 2001 to December 2013 of Chai Nat.

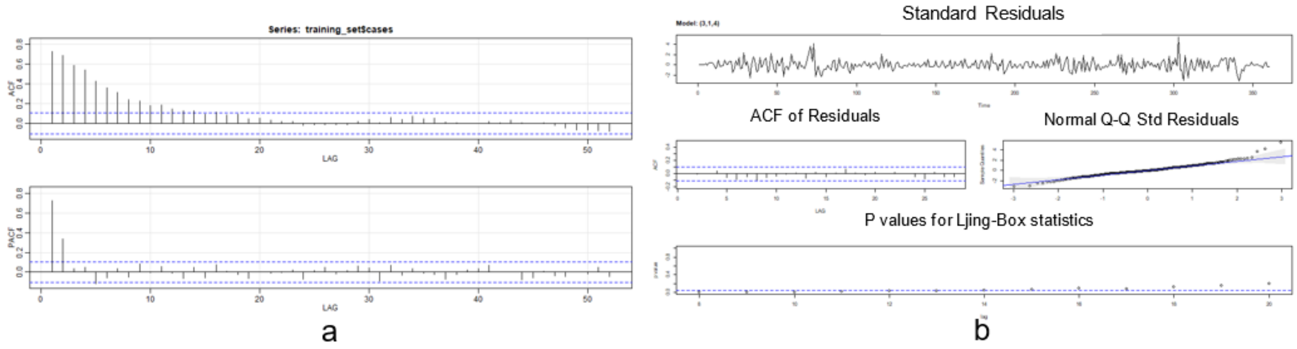

Figure 22: (a) Two plots between lag-time of dengue incidences and ACF and PACF relationship calculated from ARIMA model (b) Summary plots of time series analysis, multiple plots include the plot of predicted model over the time, the plot of ACF residual over lag-time of dengue incidences, residual Q-Q plot of standard residual, and p-value for Ljung-Box statistics of PACF relationship in Chai Nat over the training data starting from January 2001 to December 2013.

The best model of Chai Nat is based on quasi-likelihood method. The correlation coefficient on the test set in 2014 is 0.1388 (95%CI: 0.04051, 0.4768). The significant of the variables associated with p-value statistical calculation are shown in Table 7. The best model of Chai Nat uses 8 variables. The most significant variables are 1-week-lag cases and 2-week-lag cases. Other variables are, 3-week-lag cases, current week average pressure, negatively current week relative humidity, current week precipitation, 3-week-lag precipitation and current week wind power. Time series methods by ARIMA and SARIMA yield the correlation coefficient of -0.598291 and -46.02626 respectively.

Table 7: Comparison table of all methods by the highest correlation coefficient (R^2) and the lowest prediction error (RMSE) in Chai Nat.

**Methods**	**R-squared (R^2)**	**Root mean square error (RMSE)**
Poisson Regression	-1.525926	2.013269
Negative Binomial Regression	-0.4778875	1.53997
Quasi-likelihood Regression	0.1387914	1.175562
ARIMA (3,1,4)	-0.598291	1.601472
SARIMA $(2,0,1)(0,2,0)_{52}$	-46.02626	8.686837

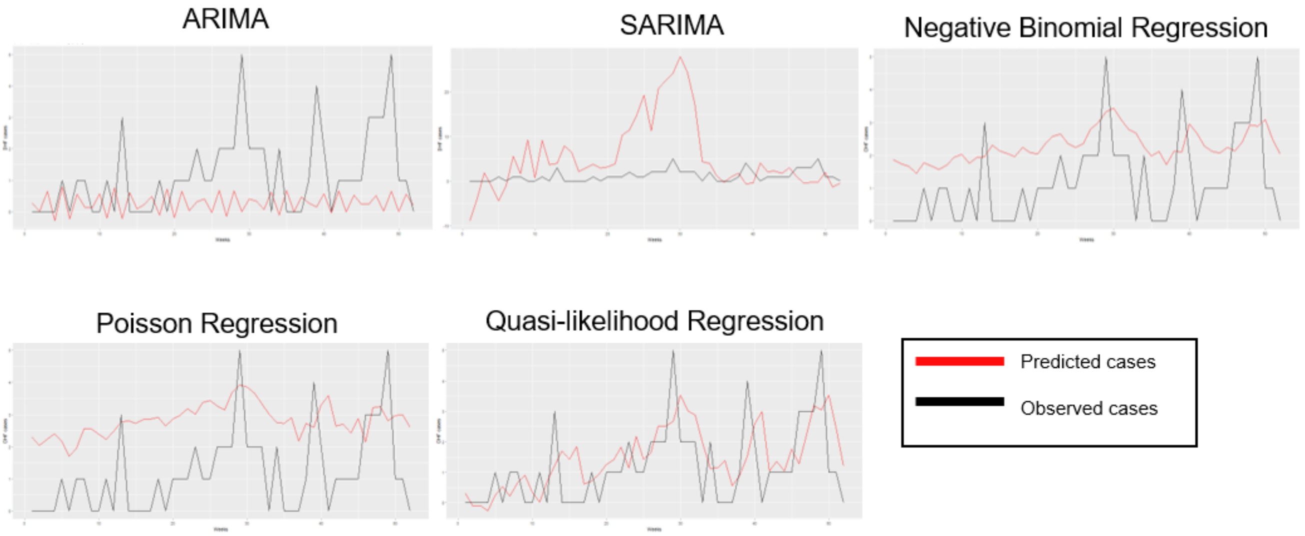

Figure 23: Plots between dengue cases and weeks, the black line represents the observed dengue cases, and the red line represents the predicted dengue cases of the best fit model of each technique over the test set data starting from January 2014 to December 2014.

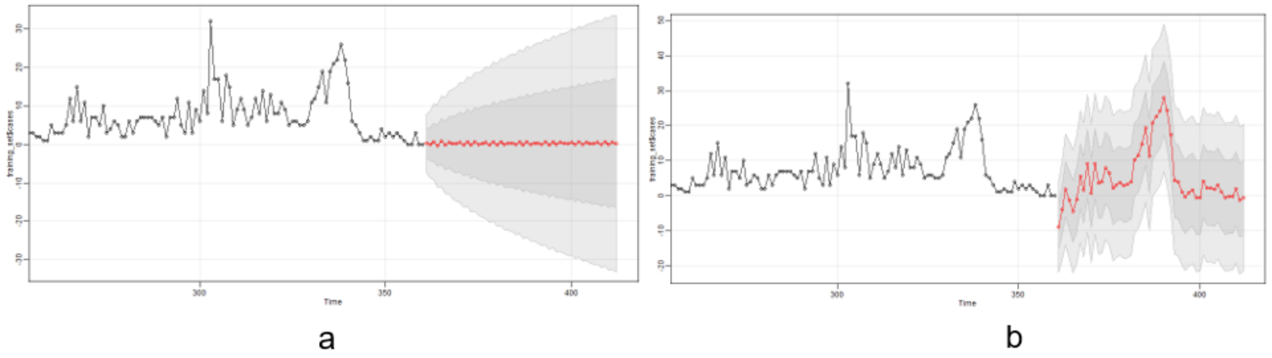

Figure 24: (a) Plot between dengue incidences over weekly time by the best model of ARIMA and (b) SARIMA time series analysis, the black line represents training set data starting from January 2012 to December 2013, and the red line represents the forecasted dengue incidences from January 2014 to December 2014.

Table 8: Coefficients and significant values of best fit GLM models, Negative Binomial, Poisson and Quasi-likelihood regression model of Chai Nat. The table summarizes coefficients of each independent variables which are composed in best fit model of each method. The significant of each variable is labelled by asterisks under the coefficients. The most important factor is marked as three asterisks which p-value ranges from 0 to 0.001. The second important factor is marked as two asterisks which p-value ranges from 0.001 to 0.01. The third important factor is marked as an asterisk which p-value ranges from 0.01 to 0.1. The least important is also marked as a dot which p-value ranges from 0.1 to 1.

**Independent variables**	**Lag**	**Coefficients/Significant**		
		NB	Poisson	Quasi
Intercept		59.050131 ***	61.8943297 ***	93.79910
Cases	1	0.057808 ***	0.0455314 ***	0.45194 ***
	2	0.043129 ***	0.0330883 ***	0.31231 ***
	3			0.04023
Average Pressure	0			-0.08913
	1		-0.0590922 ***	
	2	-0.056508 ***		
	3			
Minimum Temperature	0			
	1			
	2			
	3			
Maximum Temperature	0			
	1			
	2			
	3			
Relative Humidity	0			-0.03666
	1	-0.011621 .	-0.0127142 *	
	2			
	3			
Precipitation	0		0.0107838 *	0.05099
	1			
	2			
	3			0.06728
Vaporization	0			
	1			
	2			
	3			
Wind Direction	0		-0.0010064	
	1			
	2			
	3			
Wind Power	0	-0.014322		-0.05758
	1			
	2	-0.015447	-0.0143059 .	
	3			

Chaiyaphum

Chaiyaphum is located in the northeastern area of Thailand at 15°48′20″N 102°01′52″E. Chaiyaphum covers an area of 12,778 km^2. The total population are 1,137,049 people and the density of population is 89.0 people per km^2. Chaiyaphum has tropical savanna climate. Temperature in April is very hot around 42.6°C, range from the low of 6.8°C in December to the high of 36.4°C in April. The rainy season begins with the arrival of the southwest monsoon around mid-May. September is the wettest month, with an average rainfall of 230.0 mm. Relative humidity is high in rainy season from May-September. Humidity presents in range of 59-81 percent throughout the year.

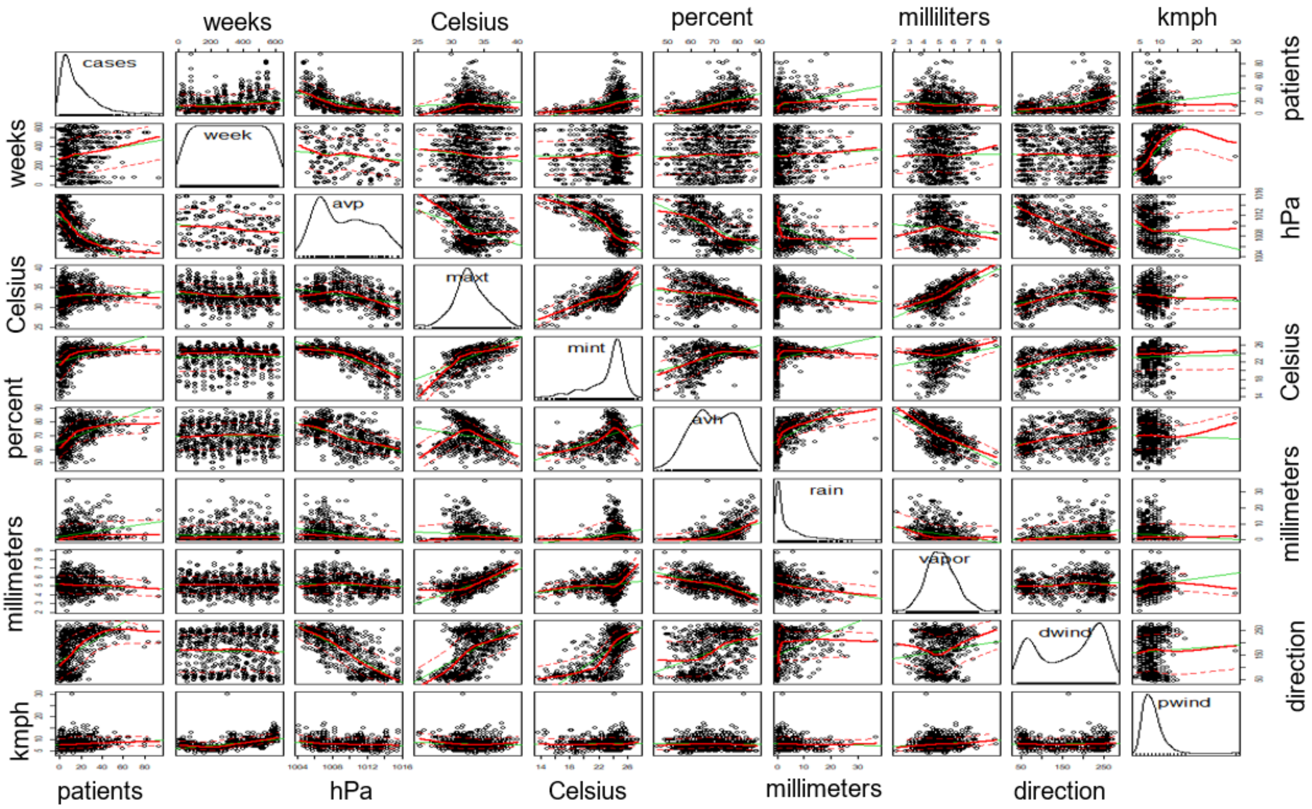

Figure 25: Scatter plot between dengue cases (cases) and selected independent variables, which are the weekly period starting from January 2001 – December 2013 (week), average pressure (avp), maximum temperature (maxt), minimum temperature (mint), average humidity (avh), precipitation (rain), vaporization of water (vapor), wind direction (dwind), and wind power (pwind). The plot visualizes pairwise hundred relationships of training set in Chaiyaphum.

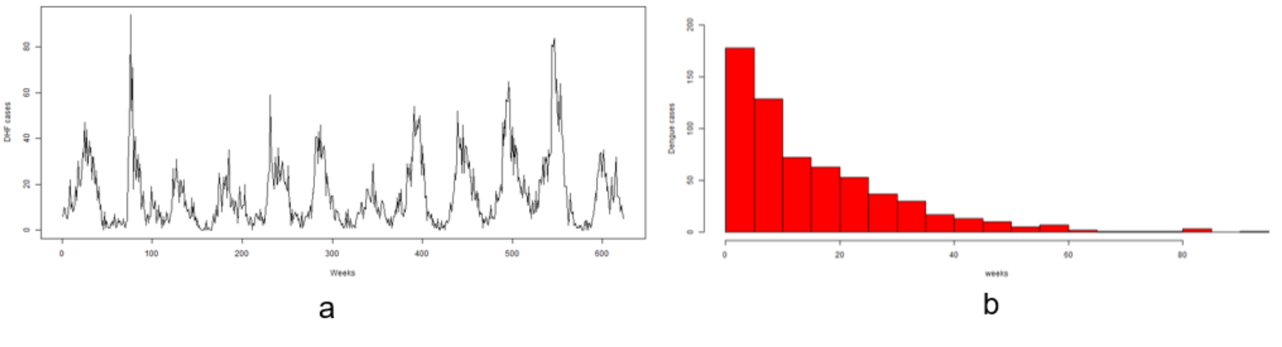

Figure 26: (a) Line plot between dengue incidences and weeks, the plot shows trends of dengue incidences in each year as stationary time series. (b) Histogram of dengue incidences in Chaiyaphum starting from January 2001 to December 2013 (624 weeks).

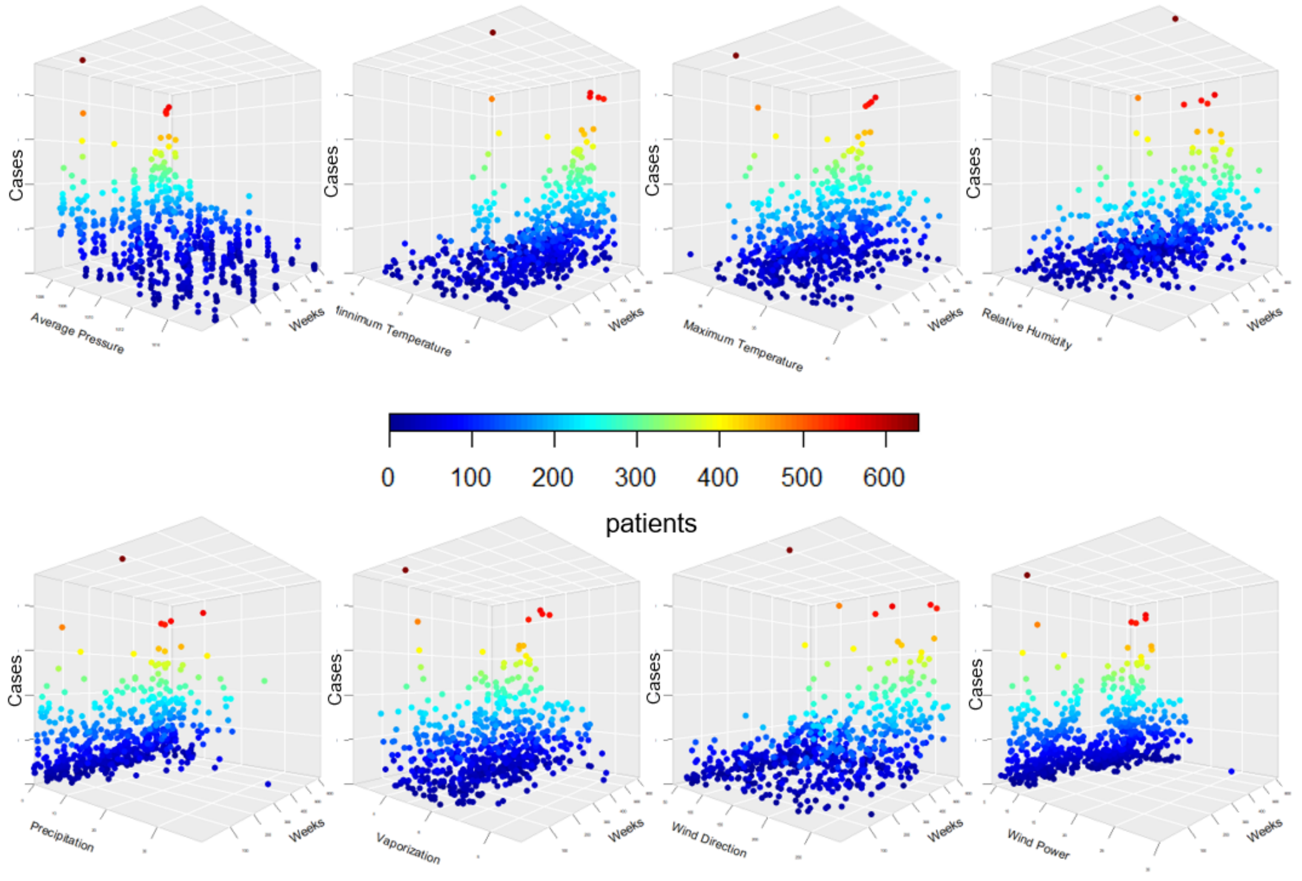

Figure 27: Three-dimensional scatter plot between dengue incidences and weather effects starting from January 2001 to December 2013 of Chaiyaphum.

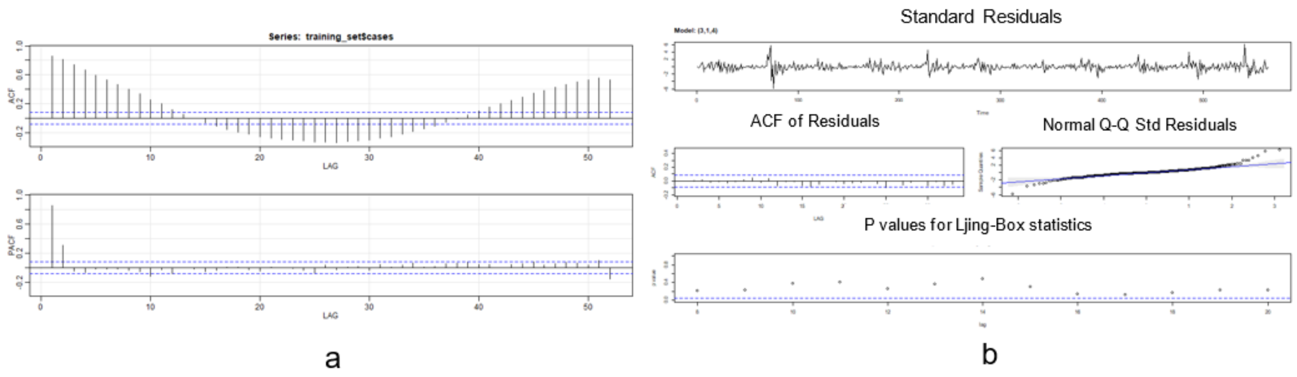

Figure 28: (a) Two plots between lag-time of dengue incidences and ACF and PACF relationship calculated from ARIMA model (b) Summary plots of time series analysis, multiple plots include the plot of predicted model over the time, the plot of ACF residual over lag-time of dengue incidences, residual Q-Q plot of standard residual, and p-value for Ljung-Box statistics of PACF relationship in Chaiyaphum over the training data starting from January 2001 to December 2013.

For Chaiyaphum, the best model is based on quasi-likelihood method. The correlation coefficient on the test set in 2014 is 0.837 (95%CI: 0.7015, 0.8874). The best model uses 8 variables. The most significant variables are 1-week-lag cases and 3-week-lag vaporization, following by current week minimum temperature, current week maximum temperature, 3-week-lag relative humidity and 3-week-lag precipitation significantly. Time series methods by ARIMA and SARIMA yield the correlation coefficient of -0.1716022 and -4.464697 respectively.

Table 9: Comparison table of all methods by the highest correlation coefficient (R^2) and the lowest prediction error (RMSE) in Chaiyaphum.

**Methods**	**R-squared (R^2)**	**Root mean square error (RMSE)**
Poisson Regression	0.8074607	4.723233
Negative Binomial Regression	0.8030094	4.777518
Quasi-likelihood Regression	0.8372657	4.342293
ARIMA (3,1,4)	-0.1716022	11.65117
SARIMA $(2,0,1)(0,2,0)_{52}$	-4.464697	25.16301

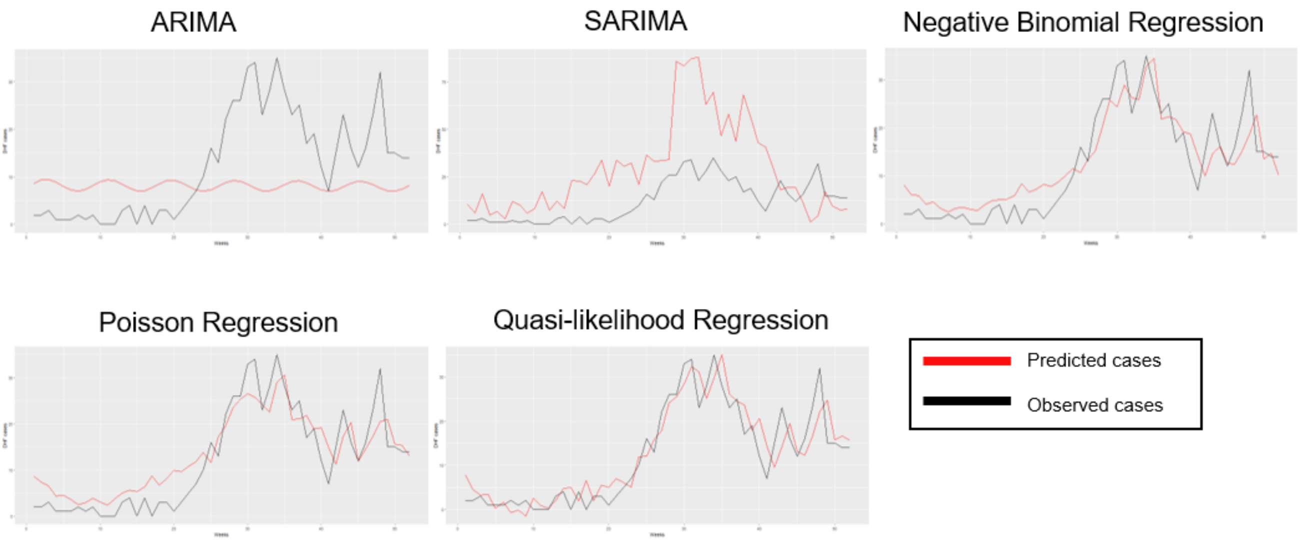

Figure 29: Plots between dengue cases and weeks, the black line represents the observed dengue cases, and the red line represents the predicted dengue cases of the best fit model of each technique over the test set data starting from January 2014 to December 2014.

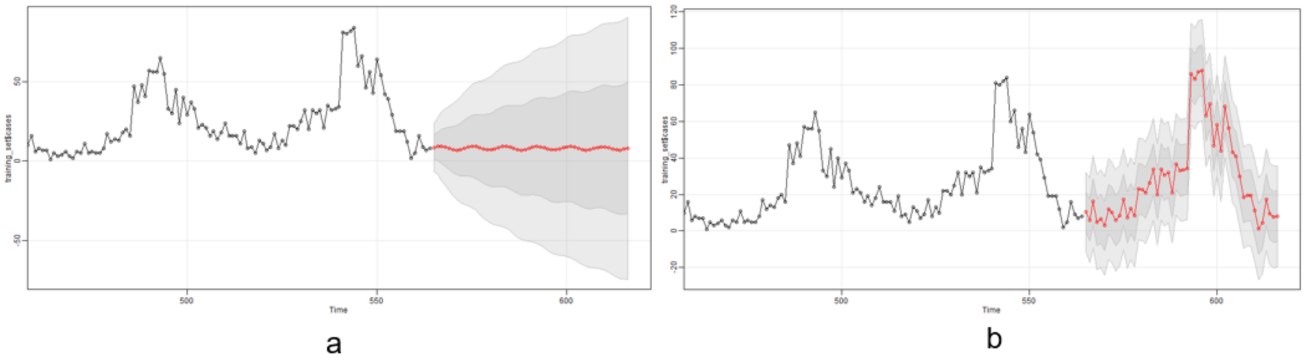

Figure 30: (a) Plot between dengue incidences over weekly time by the best model of ARIMA and (b) SARIMA time series analysis, the black line represents training set data starting from January 2012 to December 2013, and the red line represents the forecasted dengue incidences from January 2014 to December 2014.

Table 10: Coefficients and significant values of best fit GLM models, Negative Binomial, Poisson and Quasi-likelihood regression model of Chaiyaphum. The table summarizes coefficients of each independent variables which are composed in best fit model of each method. The significant of each variable is labelled by asterisks under the coefficients. The most important factor is marked as three asterisks which p-value ranges from 0 to 0.001. The second important factor is marked as two asterisks which p-value ranges from 0.001 to 0.01. The third important factor is marked as an asterisk which p-value ranges from 0.01 to 0.1. The least important is also marked as a dot which p-value ranges from 0.1 to 1.

**Independent variables**	**Lag**	**Coefficients/Significant**		
		NB	Poisson	Quasi
Intercept		-1.464940 ***	-2.9443841 ***	-11.299219
Cases	1	0.031279 ***	0.0265854 ***	0.696991 ***
	2			
	3	0.008045 ***		0.101372 **
Average Pressure	0			
	1			
	2			
	3			
Minimum Temperature	0			0.225505
	1			
	2	0.084096 ***	0.0865485 ***	
	3			
Maximum Temperature	0			-0.315880
	1			
	2			
	3			
Relative Humidity	0	0.013019 ***	0.0141223 ***	
	1			
	2			
	3		0.0171914 ***	0.111484
Precipitation	0			
	1			
	2			
	3		-0.0096811 ***	-0.096794
Vaporization	0			
	1			
	2			
	3	0.063943 *	0.1479662 ***	1.833132 ***
Wind Direction	0			0.017631 **
	1			
	2			
	3			
Wind Power	0			
	1			
	2		0.0147865 **	
	3	0.016265		

Chanthaburi

Chanthaburi is located in the eastern region of Thailand at 12°36′31″N 102°16′14″E. Chanthaburi covers an area of 6,338 km^2. The total populations are 527,350 people. The density of population is 83.2 people per km^2. Weather in Chanthaburi has tropical monsoon climate system. Rainfall is light and infrequent in winter from December to January. Temperatures are in the range 13.1°C in December to 33.9°C in April. The highest rainfall presents in June of 512.6 mm. Relative humidity are high in rainy season and low in winter. January is the highest month of sunshine hours.

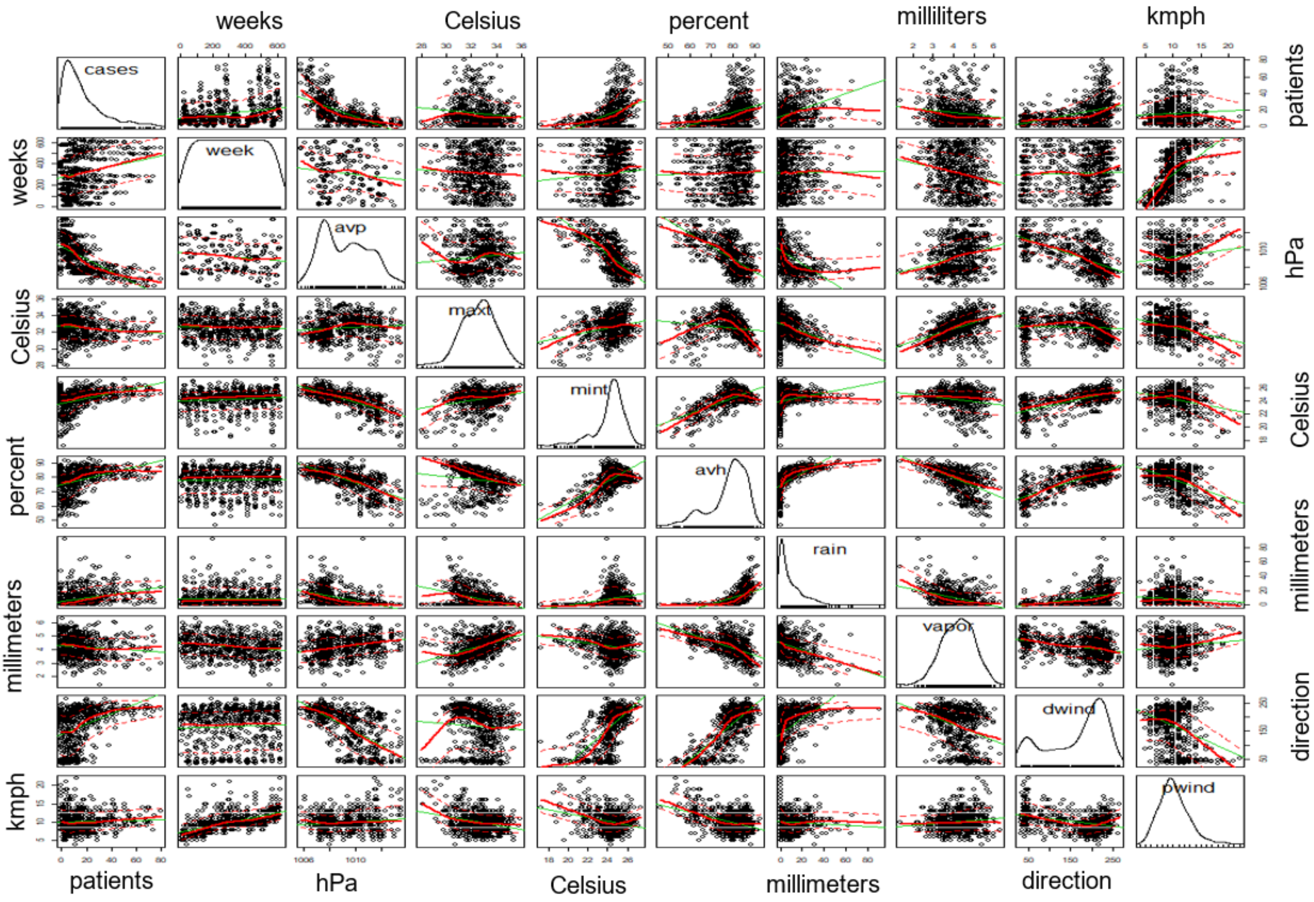

Figure 31: Scatter plot between dengue cases (cases) and selected independent variables, which are the weekly period starting from January 2001 – December 2013 (week), average pressure (avp), maximum temperature (maxt), minimum temperature (mint), average humidity (avh), precipitation (rain), vaporization of water (vapor), wind direction (dwind), and wind power (pwind). The plot visualizes pairwise hundred relationships of training set in Chanthaburi.

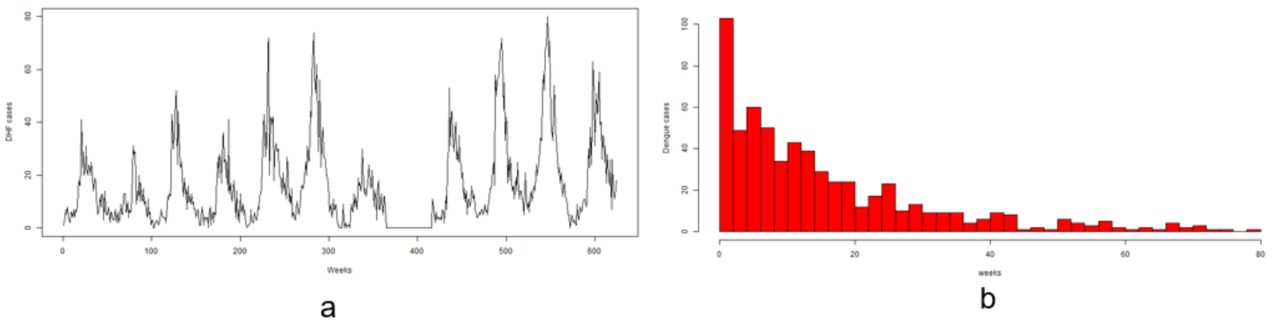

Figure 32: (a) Line plot between dengue incidences and weeks, the plot shows trends of dengue incidences in each year as stationary time series. (b) Histogram of dengue incidences in Chanthaburi starting from January 2001 to December 2013 (624 weeks).

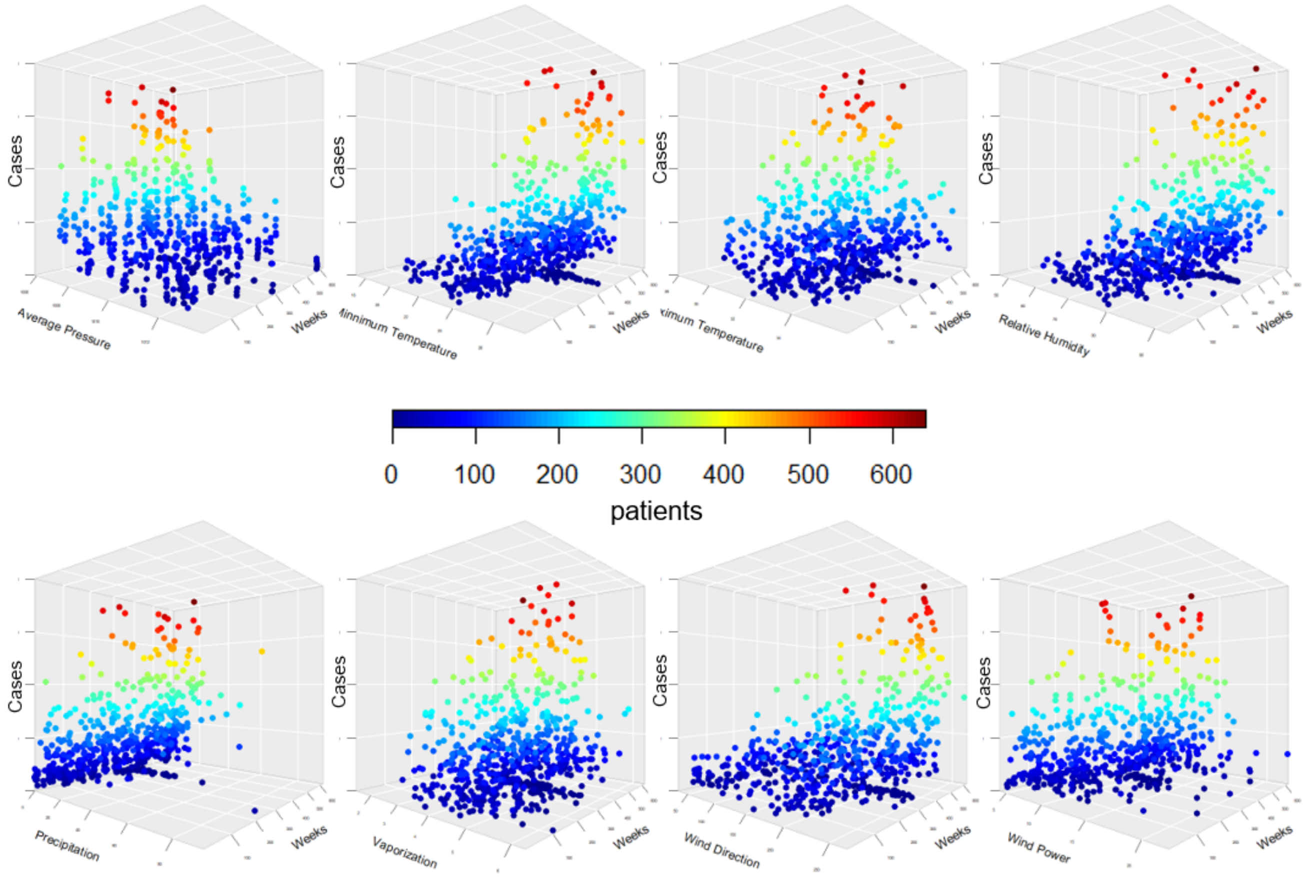

Figure 33: Three-dimensional scatter plot between dengue incidences and weather effects starting from January 2001 to December 2013 of Chanthaburi.

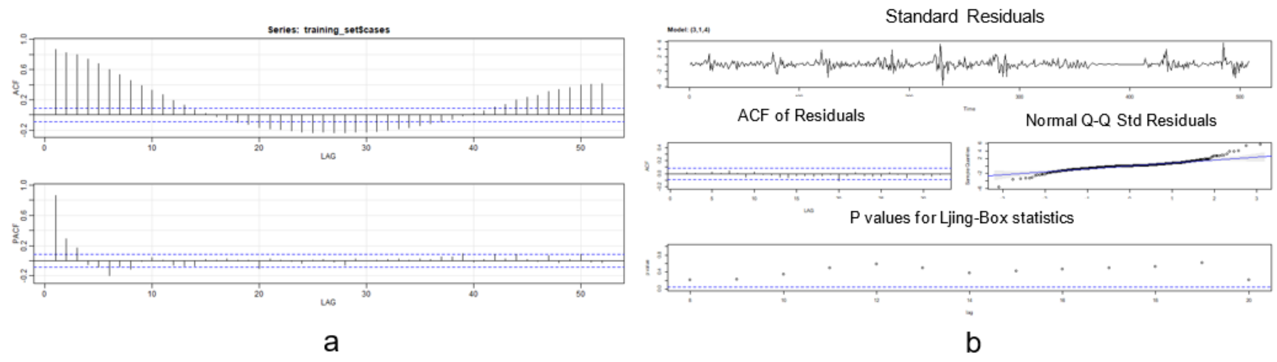

Figure 34: (a) Two plots between lag-time of dengue incidences and ACF and PACF relationship calculated from ARIMA model (b) Summary plots of time series analysis, multiple plots include the plot of predicted model over the time, the plot of ACF residual over lag-time of dengue incidences, residual Q-Q plot of standard residual, and p-value for Ljung-Box statistics of PACF relationship in Chanthaburi over the training data starting from January 2001 to December 2013.

For Chanthaburi, the best model is based on quasi-likelihood method. The model of consists of 8 variables. The most significant variable are 1-week-lag cases, 2-week-lag cases, 3-week-lag cases and current week average. Other variables which have less significant are, current week precipitation, 3-week-lag and 1-week-lag maximum temperature and negatively 3-week-lag minimum temperature. The correlation coefficient on the test set in 2014 is 0.836 (95%CI: 0.7835, 0.8889). The best fit model of time series methods by ARIMA and SARIMA yield the correlation coefficient of -0.1044972 and 0.4721719 respectively.

Table 11: Comparison table of all methods by the highest correlation coefficient (R^2) and the lowest prediction error (RMSE) in chanthaburi.

**Methods**	**R-squared (R^2)**	**Root mean square error (RMSE)**
Poisson Regression	0.7085356	10.02817
Negative Binomial Regression	0.4489555	13.78866
Quasi-likelihood Regression	0.8358667	7.525358
ARIMA (3,1,4)	-0.1044972	19.52141
SARIMA $(2,0,1)(0,2,0)_{52}$	0.4721719	13.49507

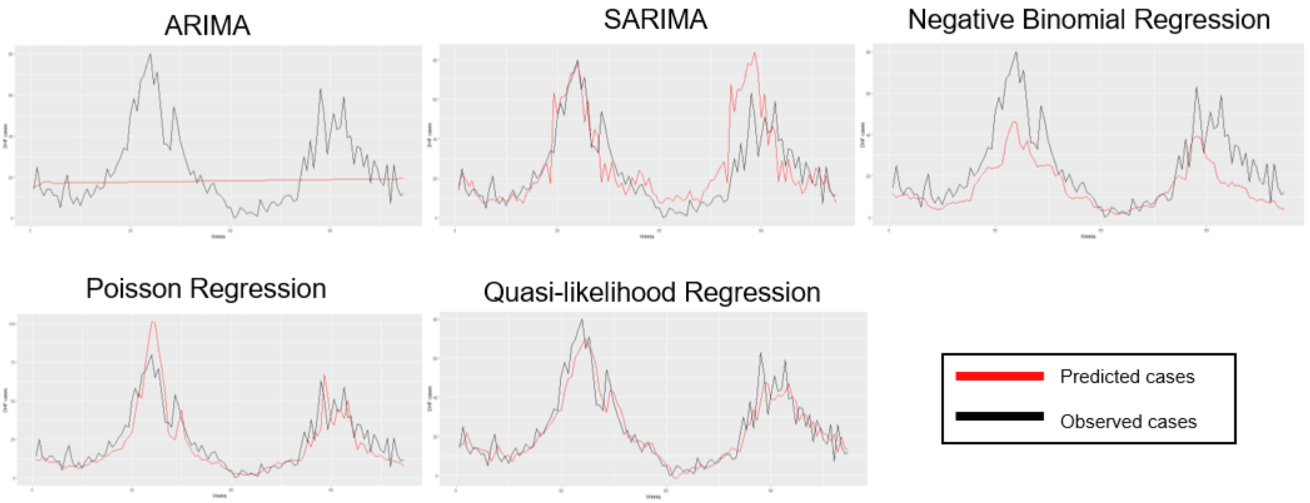

Figure 35: Plots between dengue cases and weeks, the black line represents the observed dengue cases, and the red line represents the predicted dengue cases of the best fit model of each technique over the test set data starting from January 2014 to December 2014.

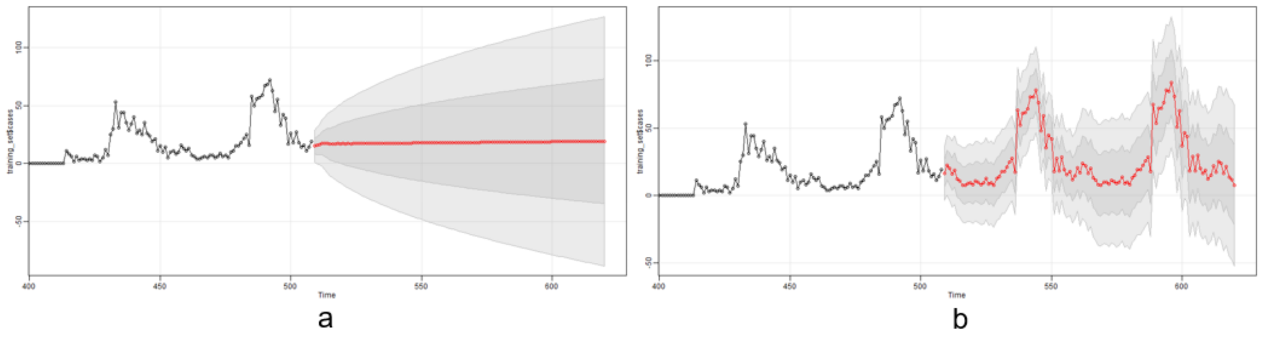

Figure 36: (a) Plot between dengue incidences over weekly time by the best model of ARIMA and (b) SARIMA time series analysis, the black line represents training set data starting from January 2012 to December 2013, and the red line represents the forecasted dengue incidences from January 2014 to December 2014.

Table 12: Coefficients and significant values of best fit GLM models, Negative Binomial, Poisson and Quasi-likelihood regression model of Chanthaburi. The table summarizes coefficients of each independent variables which are composed in best fit model of each method. The significant of each variable is labelled by asterisks under the coefficients. The most important factor is marked as three asterisks which p-value ranges from 0 to 0.001. The second important factor is marked as two asterisks which p-value ranges from 0.001 to 0.01. The third important factor is marked as an asterisk which p-value ranges from 0.01 to 0.1. The least important is also marked as a dot which p-value ranges from 0.1 to 1.

**Independent variables**	**Lag**	**Coefficients/Significant**		
		NB	Poisson	Quasi
Intercept		433.8 ***	-3.630395 ***	1223.59197 ***
Cases	1		0.021564 ***	0.51822 ***
	2		0.013326 ***	0.17151 ***
	3			0.19061 ***
Average Pressure	0			-1.22235 ***
	1	-0.2766 ***		
	2	-0.09522		
	3	-0.05727		
Minimum Temperature	0			
	1			
	2		0.072033 ***	
	3		0.041079 **	-0.45196
Maximum Temperature	0	0.05222	0.117344 ***	
	1			0.21020
	2			
	3			0.50716 .
Relative Humidity	0			
	1			
	2			
	3			
Precipitation	0	0.004471		-0.07192 *
	1			
	2			
	3			
Vaporization	0		0.036790 *	
	1			
	2			
	3			
Wind Direction	0			
	1			
	2			
	3	-0.0008245		
Wind Power	0			
	1			
	2			
	3		-0.024371 ***	

Chiang Mai

Chiang Mai is in northern region Thailand at 18°47′43″N 98°59′55″E. Chiang Mai covers an area of 20,107 km^2. Total population are 1,678,284 people. The density of population is 83.5 km^2. Weather in Chiang Mai has tropical savanna climate under the South Asian monsoon system which controls more tropical wet and dry climate. Temperature is in the range from the low of 15.7°C in December to the high of 41.4°C in April. The rainy season begins with the arrival of the southwest monsoon around mid-May. The humidity presents the average of 95 percent. Precipitation occurs from mid-May to August. The average precipitation is in the range of 211.4 - 216.9 mm annually.

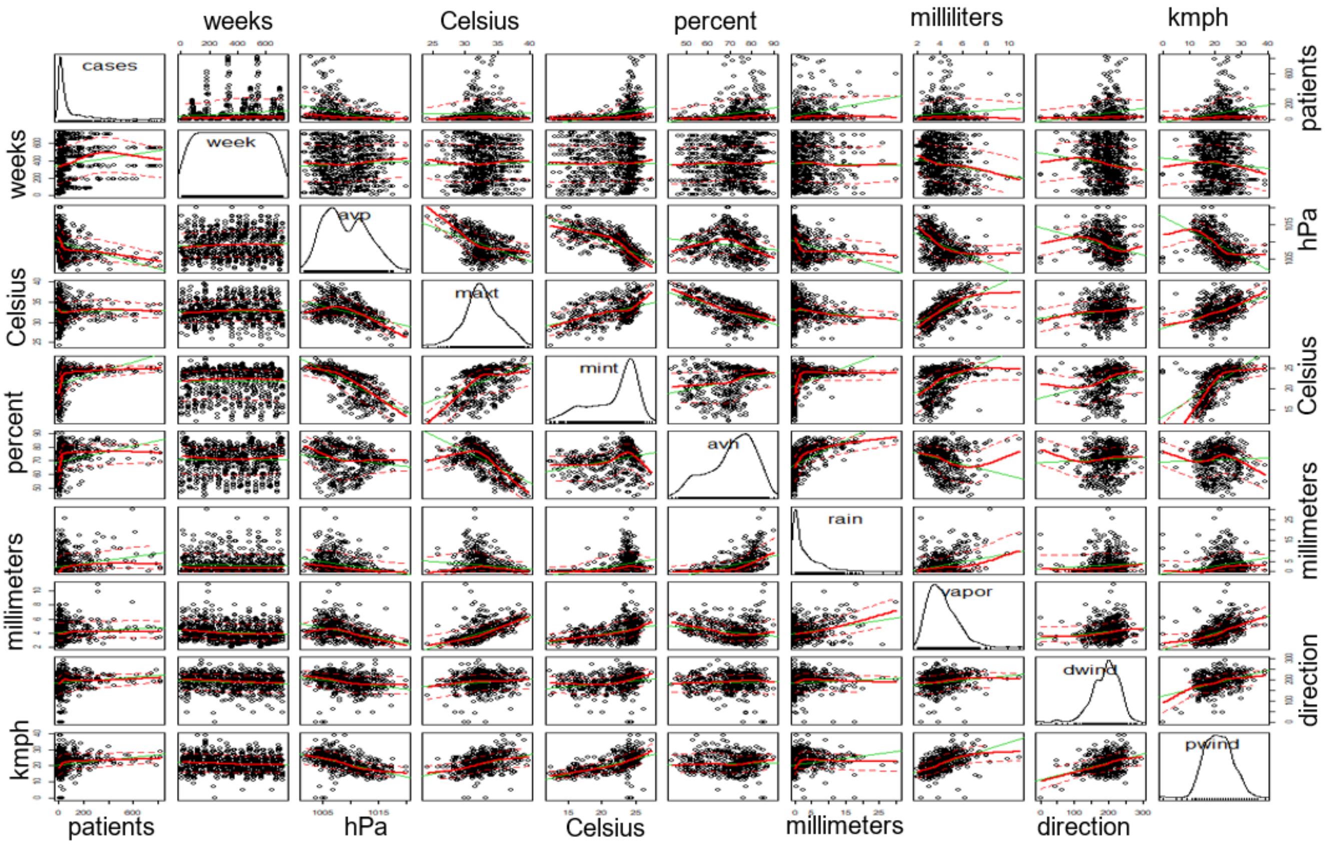

Figure 37: Scatter plot between dengue cases (cases) and selected independent variables, which are the weekly period starting from January 2001 – December 2013 (week), average pressure (avp), maximum temperature (maxt), minimum temperature (mint), average humidity (avh), precipitation (rain), vaporization of water (vapor), wind direction (dwind), and wind power (pwind). The plot visualizes pairwise hundred relationships of training set in Chiang Mai.

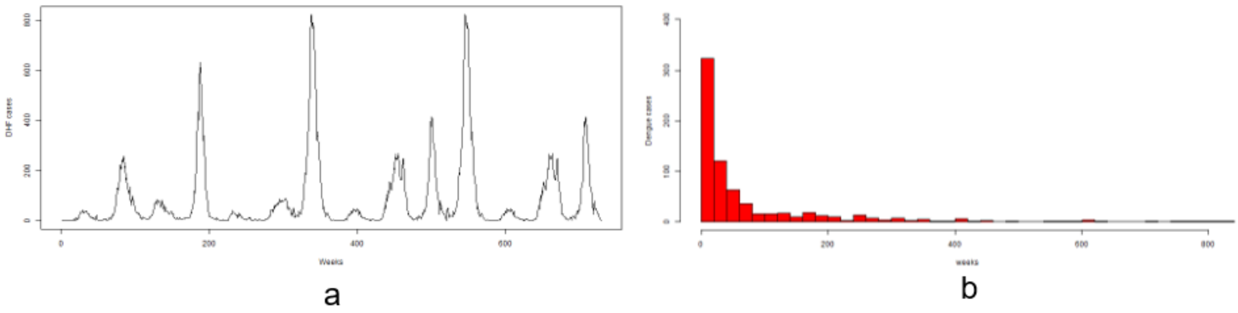

Figure 38: (a) Line plot between dengue incidences and weeks, the plot shows trends of dengue incidences in each year as stationary time series. (b) Histogram of dengue incidences in Chiang Mai starting from January 2001 to December 2013 (624 weeks).

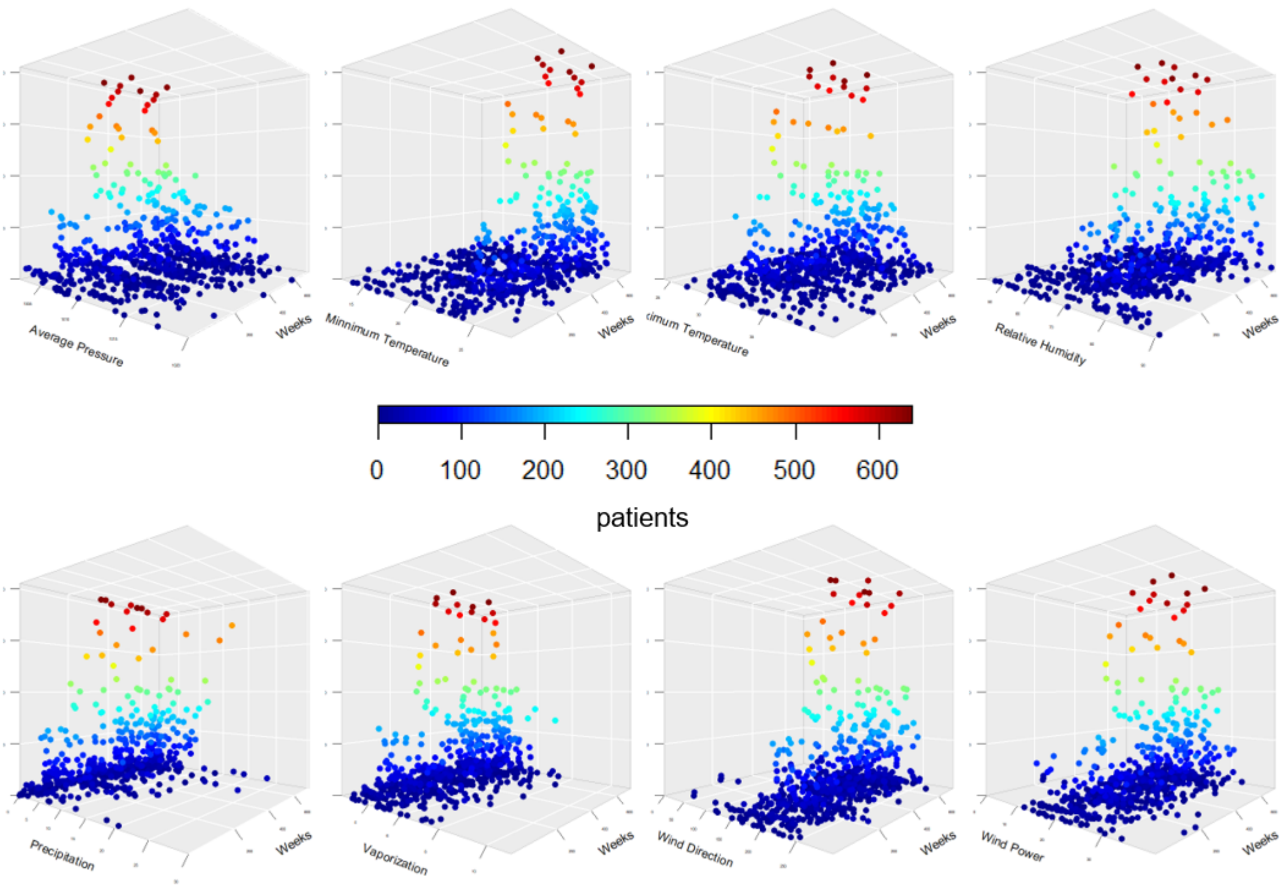

Figure 39: Three-dimensional scatter plot between dengue incidences and weather effects starting from January 2001 to December 2013 of Chiang Mai.

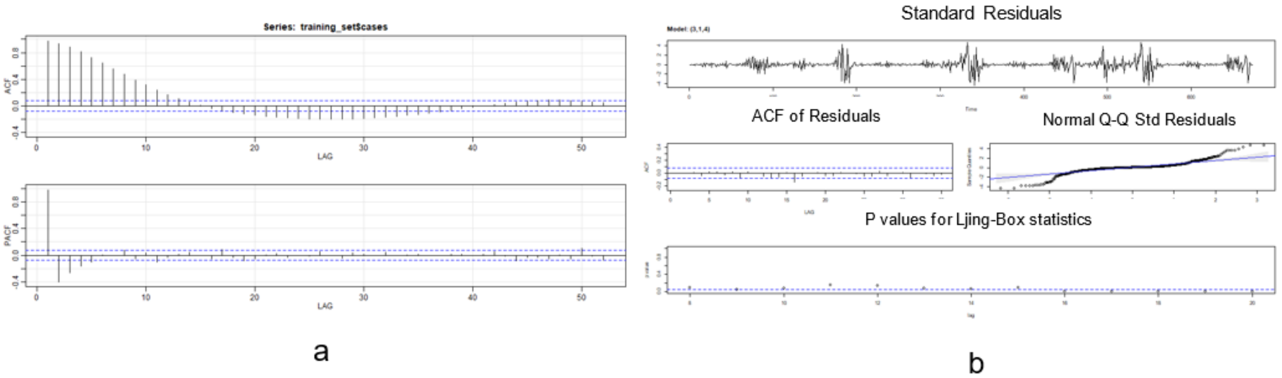

Figure 40: (a) Two plots between lag-time of dengue incidences and ACF and PACF relationship calculated from ARIMA model (b) Summary plots of time series analysis, multiple plots include the plot of predicted model over the time, the plot of ACF residual over lag-time of dengue incidences, residual Q-Q plot of standard residual, and p-value for Ljung-Box statistics of PACF relationship in Chiang Mai over the training data starting from January 2001 to December 2013.

For Chiang Mai, the best model is also based on quasi-likelihood regression. The variables of the best model are significantly included by 1-week-lag cases, 3-week-lag cases, current week minimum temperature and 3-week lag precipitation respectively. Other component variables are current week minimum temperature, 3-week-lag relative humidity, 3-week lag vaporization and current week wind direction. The correlation coefficient of this model yields 0.931 accuracy (95%CI: 0.8959, 0.9696). Quasi-likelihood and time series analysis are compared with correlation coefficient by the result of ARIMA and SRIMA, -0.3845301 and 0.2729779 respectively.

Table 13: Comparison table of all methods by the highest correlation coefficient (R^2) and the lowest prediction error (RMSE) in Chiang Mai.

Methods	R-squared (R^2)	Root mean square error (RMSE)
Poisson Regression	0.8265549	49.54987
Negative Binomial Regression	0.7844067	55.24324
Quasi-likelihood Regression	0.9367849	29.91383
ARIMA (3,1,4)	-0.3845301	139.9951
SARIMA $(2,0,1)(0,2,0)_{52}$	0.2729779	101.4461

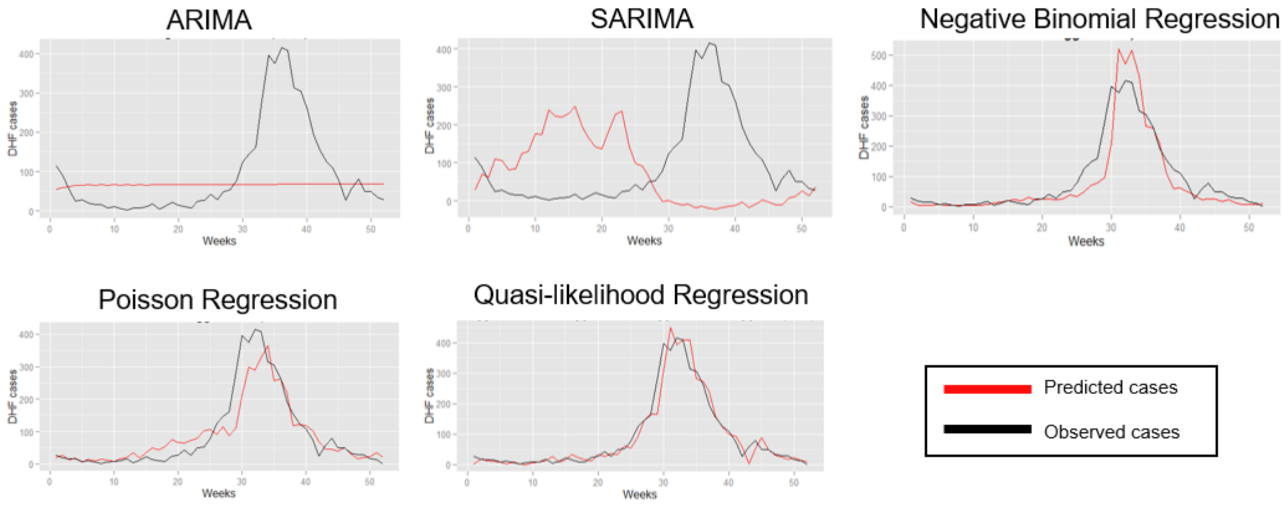

Figure 41: Plots between dengue cases and weeks, the black line represents the observed dengue cases, and the red line represents the predicted dengue cases of the best fit model of each technique over the test set data starting from January 2014 to December 2014.

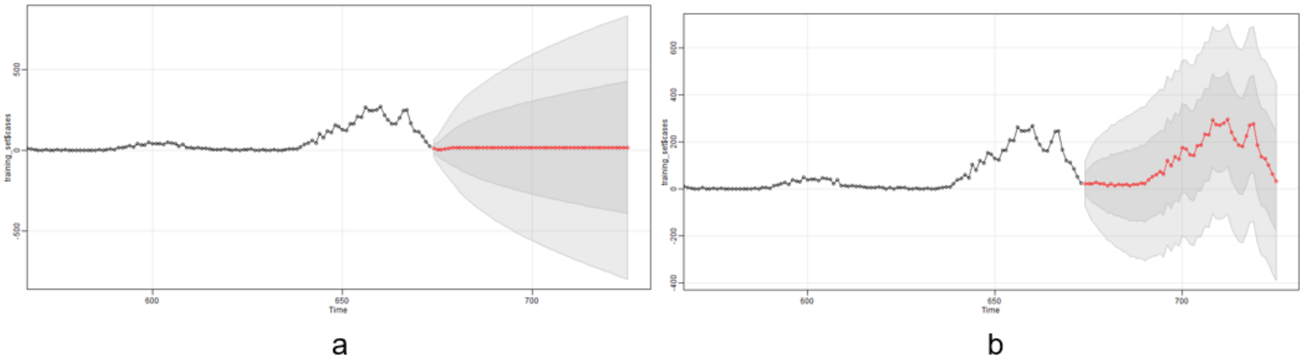

Figure 42: (a) Plot between dengue incidences over weekly time by the best model of ARIMA and (b) SARIMA time series analysis, the black line represents training set data starting from January 2012 to December 2013, and the red line represents the forecasted dengue incidences from January 2014 to December 2014.

Table 14: Coefficients and significant values of best fit GLM models, Negative Binomial, Poisson and Quasi-likelihood regression model of Chiang Mai. The table summarizes coefficients of each independent variables which are composed in best fit model of each method. The significant of each variable is labelled by asterisks under the coefficients. The most important factor is marked as three asterisks which p-value ranges from 0 to 0.001. The second important factor is marked as two asterisks which p-value ranges from 0.001 to 0.01. The third important factor is marked as an asterisk which p-value ranges from 0.01 to 0.1. The least important is also marked as a dot which p-value ranges from 0.1 to 1.

**Independent variables**	**Lag**	**Coefficients/Significant**		
		NB	Poisson	Quasi
Intercept		-30.4152 *	-8.52762 ***	-35.6738
Cases	1	0.007463 ***	0.004067 ***	1.21427 ***
	2			
	3			-0.26087 ***
Average Pressure	0			
	1	0.028899 *		
	2		-0.00167	
	3			
Minimum Temperature	0	0.068606 **		1.21268 **
	1	0.143503 **		
	2			
	3			
Maximum Temperature	0		0.200302 ***	0.05518
	1			
	2		0.054238 ***	
	3			
Relative Humidity	0		0.076745 ***	
	1			
	2			
	3			0.06801
Precipitation	0		-0.02594 ***	
	1			
	2			
	3			-0.93022 **
Vaporization	0	-0.11043 ***		
	1			
	2			
	3			0.77040
Wind Direction	0			0.03154
	1			
	2			
	3			
Wind Power	0			
	1			
	2			
	3			

Ching Rai

Chiang Rai is a province located in northern region of Thailand, in the mountainous geography at 19°54′34″N 99°49′39″E. Chiang Rai covers an area of 11,678 km^2. Total population are 1,207,699 people. The density of population is 103.0 km^2. Weather in Chiang Rai has tropical savanna climate under the South Asian monsoon system which controls more tropical wet and dry climate. The average temperature throughout the year is around 23°C. Temperature is in the range from the low of 12.8°C in January to the high of 40.5°C in April. The rainy season begins with the arrival of the southwest monsoon around mid-May. The humidity presents the average of 94 percent. Precipitation starts to rise from mid-May to August. The average precipitation is in the range of 310.9 - 358.4 mm annually.

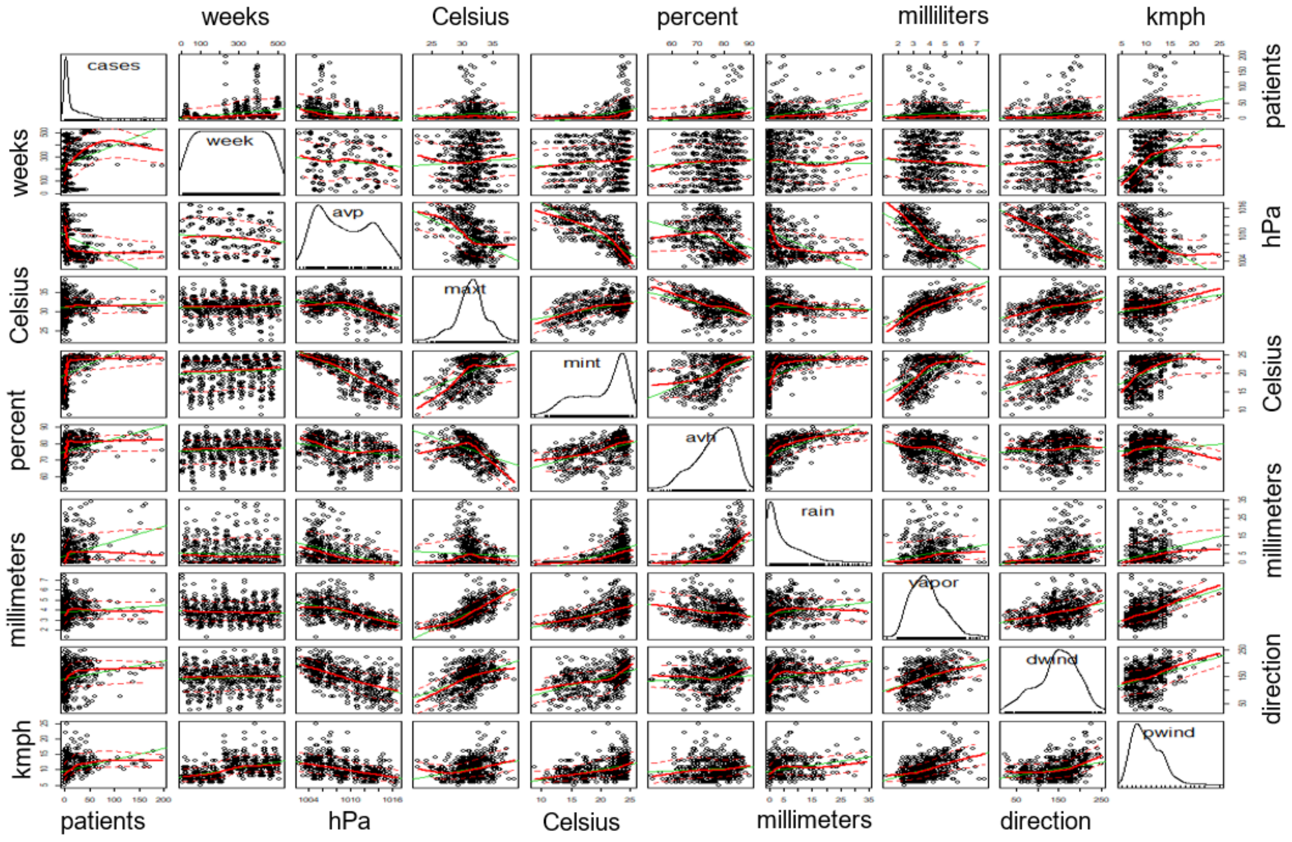

Figure 43: Scatter plot between dengue cases (cases) and selected independent variables, which are the weekly period starting from January 2001 – December 2013 (week), average pressure (avp), maximum temperature (maxt), minimum temperature (mint), average humidity (avh), precipitation (rain), vaporization of water (vapor), wind direction (dwind), and wind power (pwind). The plot visualizes pairwise hundred relationships of training set in Chiang Rai.

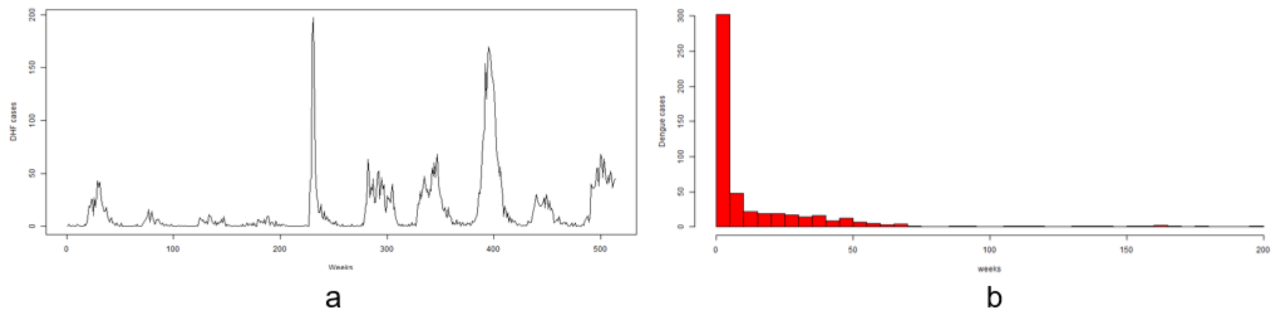

Figure 44: (a) Line plot between dengue incidences and weeks, the plot shows trends of dengue incidences in each year as stationary time series. (b) Histogram of dengue incidences in Chiang Rai starting from January 2001 to December 2013 (624 weeks).

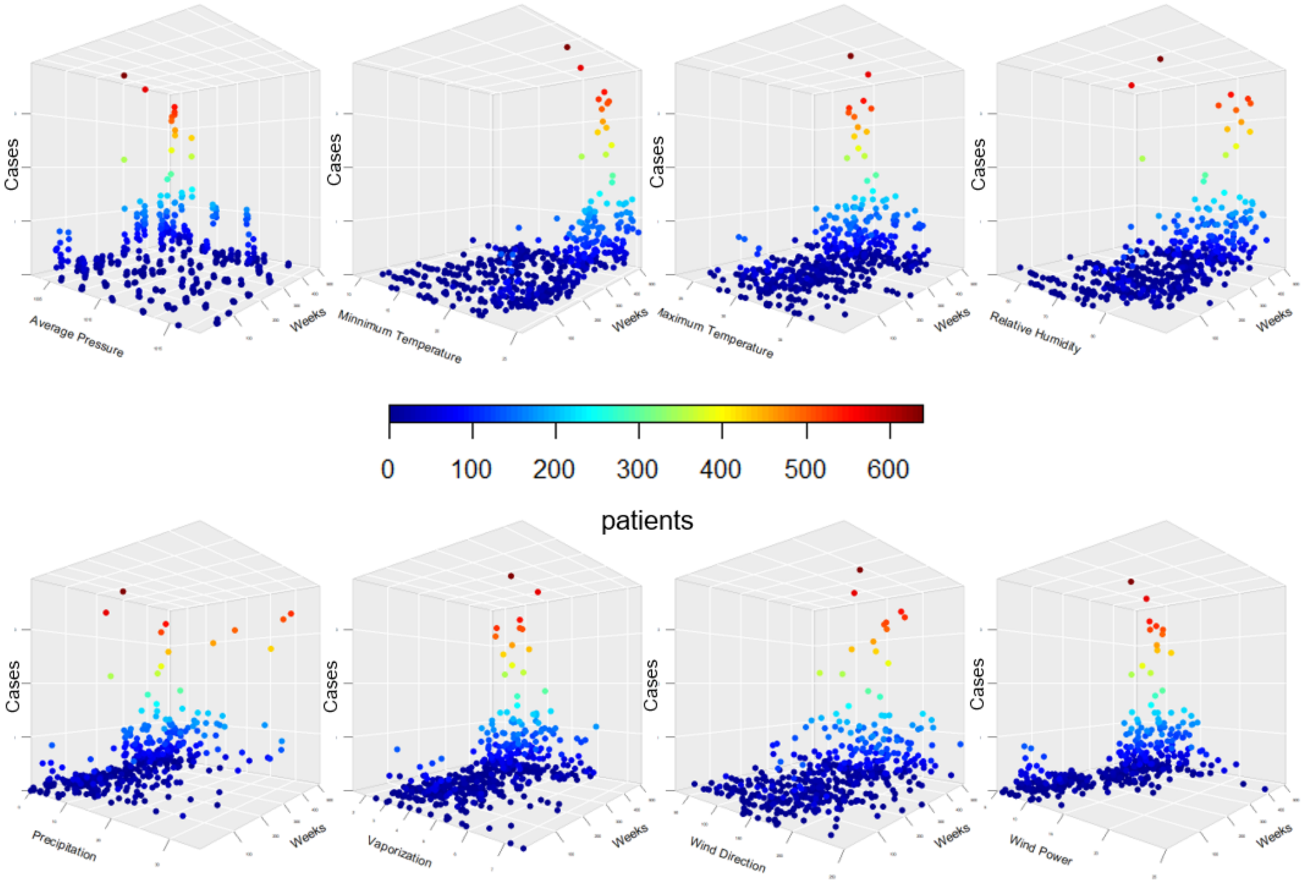

Figure 45: Three-dimensional scatter plot between dengue incidences and weather effects starting from January 2001 to December 2013 of Chiang Rai.

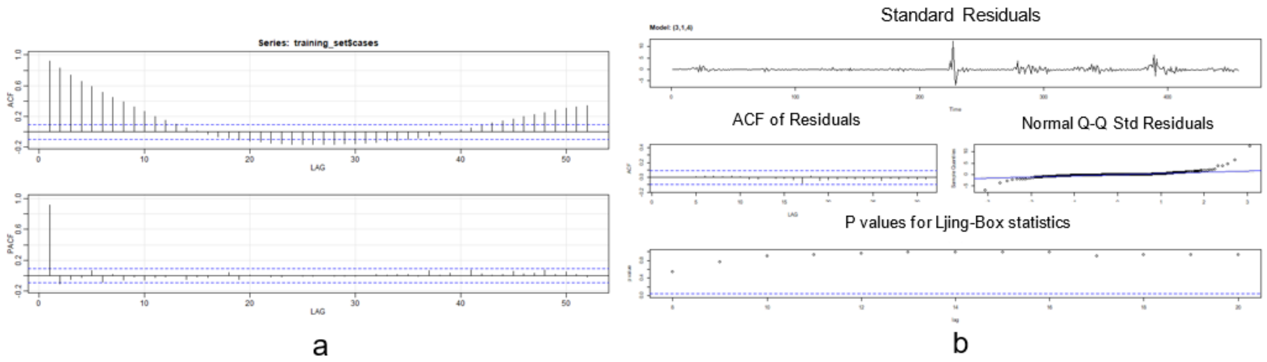

Figure 46: (a) Two plots between lag-time of dengue incidences and ACF and PACF relationship calculated from ARIMA model (b) Summary plots of time series analysis, multiple plots include the plot of predicted model over the time, the plot of ACF residual over lag-time of dengue incidences, residual Q-Q plot of standard residual, and p-value for Ljung-Box statistics of PACF relationship in Chiang Rai over the training data starting from January 2001 to December 2013.

The results of best fit model in Chiang Rai is based on quasi-likelihood method. The correlation coefficient presents 0.899 (95%CI: 0.8242, 0.9507). Chiang Rai model are consisted of 7 variables. The most significant consistency presents as 1-week-lag cases. Other variables include current week average pressure, current week relative humidity, current week vaporization, 2-week-lag vaporization and 3-week-lag wind power. Comparison to times series analysis, ARIMA methods present the correlation coefficient 0.277 and SRIMA presents 0.081.

Table 15: Comparison table of all methods by the highest correlation coefficient (R^2) and the lowest prediction error (RMSE) in Chiang Rai.

**Methods**	**R-squared (R^2)**	**Root mean square error (RMSE)**
Poisson Regression	0.7638945	11.11735
Negative Binomial Regression	0.835330	12.49723
Quasi-likelihood Regression	0.899159	7.265496
ARIMA (3,1,4)	0.2767596	19.45761
SARIMA $(2,0,1)(0,2,0)_{52}$	0.0809553	21.78708

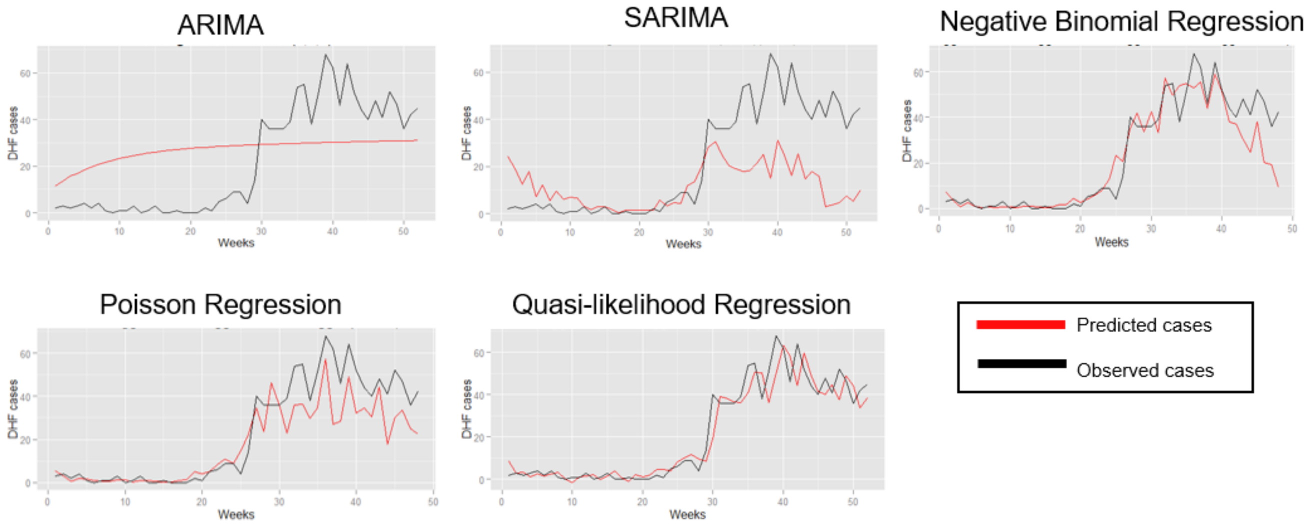

Figure 47: Plots between dengue cases and weeks, the black line represents the observed dengue cases, and the red line represents the predicted dengue cases of the best fit model of each technique over the test set data starting from January 2014 to December 2014.

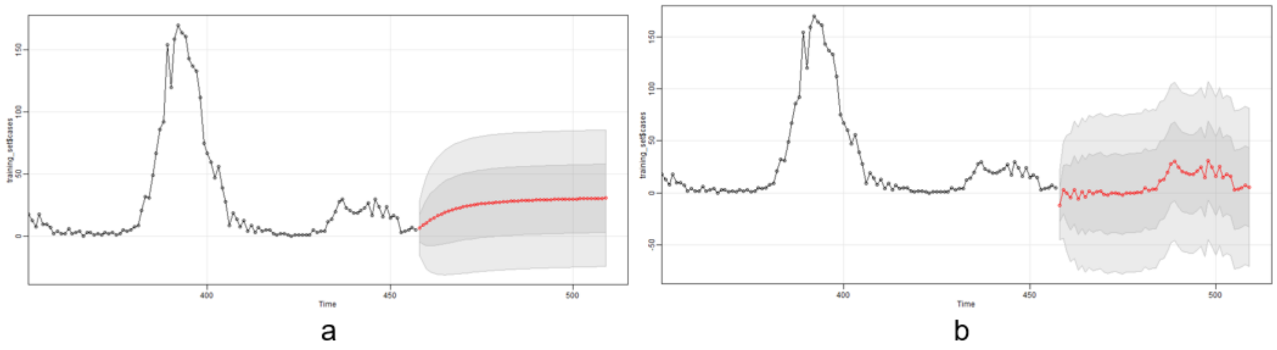

Figure 48: (a) Plot between dengue incidences over weekly time by the best model of ARIMA and (b) SARIMA time series analysis, the black line represents training set data starting from January 2012 to December 2013, and the red line represents the forecasted dengue incidences from January 2014 to December 2014.

Table 16: Coefficients and significant values of best fit GLM models, Negative Binomial, Poisson and Quasi-likelihood regression model of Chiang Rai. The table summarizes coefficients of each independent variables which are composed in best fit model of each method. The significant of each variable is labelled by asterisks under the coefficients. The most important factor is marked as three asterisks which p-value ranges from 0 to 0.001. The second important factor is marked as two asterisks which p-value ranges from 0.001 to 0.01. The third important factor is marked as an asterisk which p-value ranges from 0.01 to 0.1. The least important is also marked as a dot which p-value ranges from 0.1 to 1.

**Independent variables**	**Lag**	**Coefficients/Significant**		
		NB	Poisson	Quasi
Intercept		-9.20670 ***	-32.44427 ***	-26.9352
Cases	1	0.0318611 ***		0.89884 ***
	2			
	3		0.011953 ***	
Average Pressure	0	-0.077345	-0.082805 ***	
	1		0.110325 ***	0.01747
	2			
	3			
Minimum Temperature	0			
	1			
	2			
	3	0.162877 ***	0.315904 ***	
Maximum Temperature	0			
	1			
	2	0.0274712		
	3		-0.062183 ***	
Relative Humidity	0			0.02460
	1			
	2	0.044549 **		
	3			
Precipitation	0			
	1			
	2			
	3			
Vaporization	0	0.075895		1.15801
	1			
	2			-0.27494
	3			
Wind Direction	0	0.001159		
	1	0.002213	0.003469 ***	
	2			
	3	-0.000835		0.01830
Wind Power	0			
	1			
	2			
	3		0.119506 ***	0.27942

Chonburi

Chon buri is located in the central region of Thailand at coordinate of 13°21′40″N 100°59′06″E. Chon buri covers an area of 4,363 km^2. Total population are 1,421,425 people. The density of population is 326.0 people per km^2. Weather in Chon buri follows tropical savanna climate system. The highest temperature is in April approximately 39.9° C. The low temperature presents in winter from December to March (13 to 17°C). The monsoon season starts from May through October. The highest sunshine hours are in January. Humidity presents in range of 66-79 percent throughout the year.

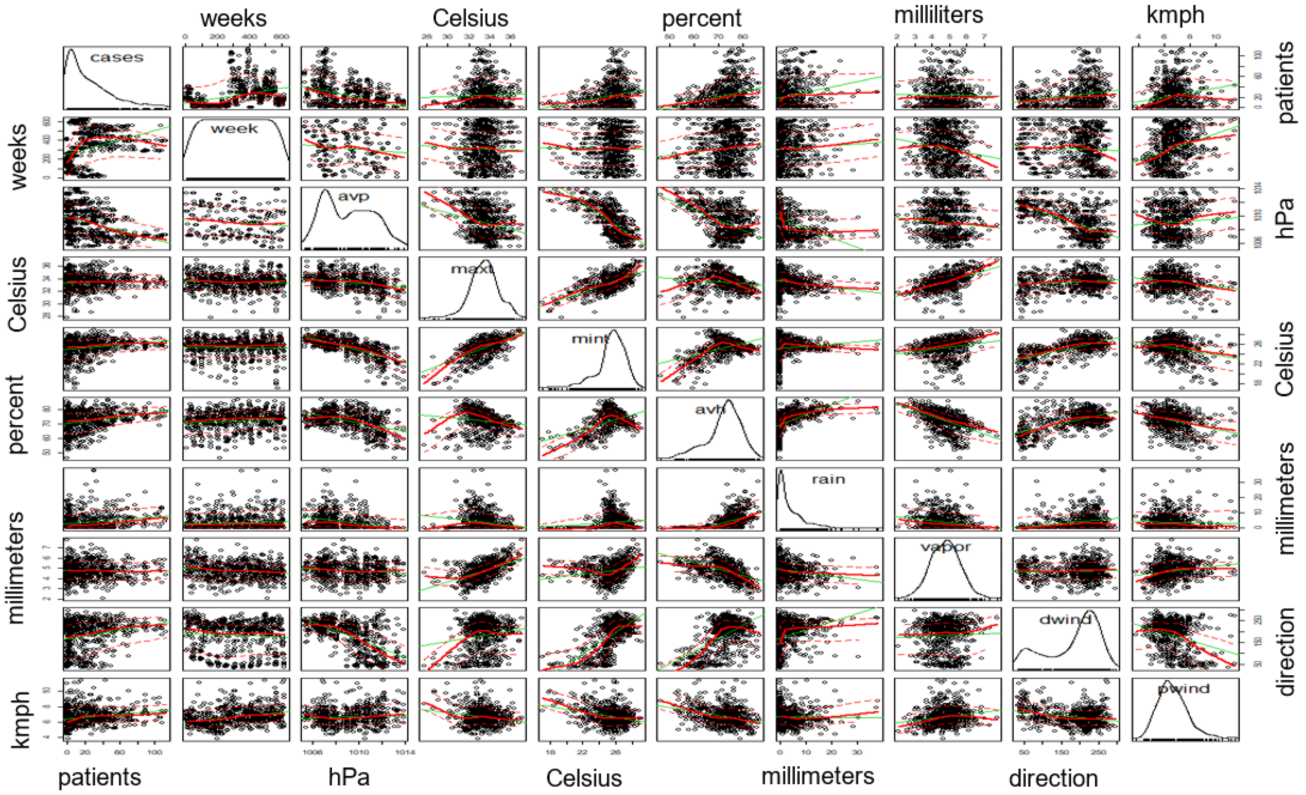

Figure 49: Scatter plot between dengue cases (cases) and selected independent variables, which are the weekly period starting from January 2001 – December 2013 (week), average pressure (avp), maximum temperature (maxt), minimum temperature (mint), average humidity (avh), precipitation (rain), vaporization of water (vapor), wind direction (dwind), and wind power (pwind). The plot visualizes pairwise hundred relationships of training set in Chonburi.

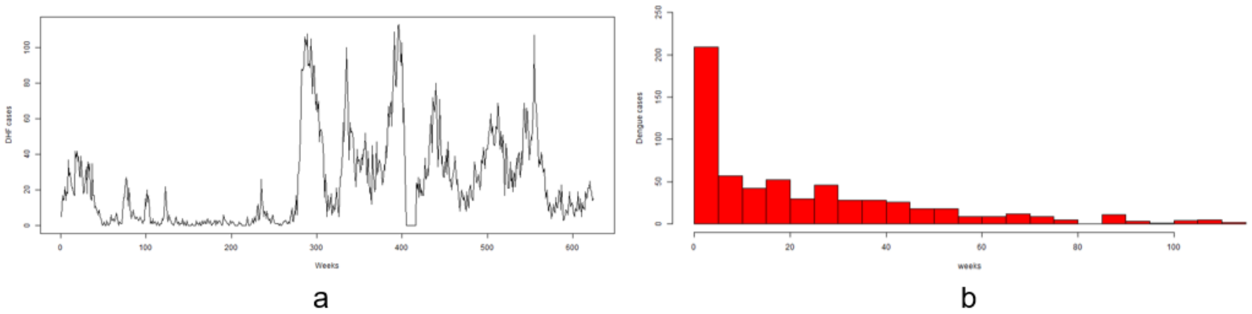

Figure 50: (a) Line plot between dengue incidences and weeks, the plot shows trends of dengue incidences in each year as stationary time series. (b) Histogram of dengue incidences in Chonburi starting from January 2001 to December 2013 (624 weeks).

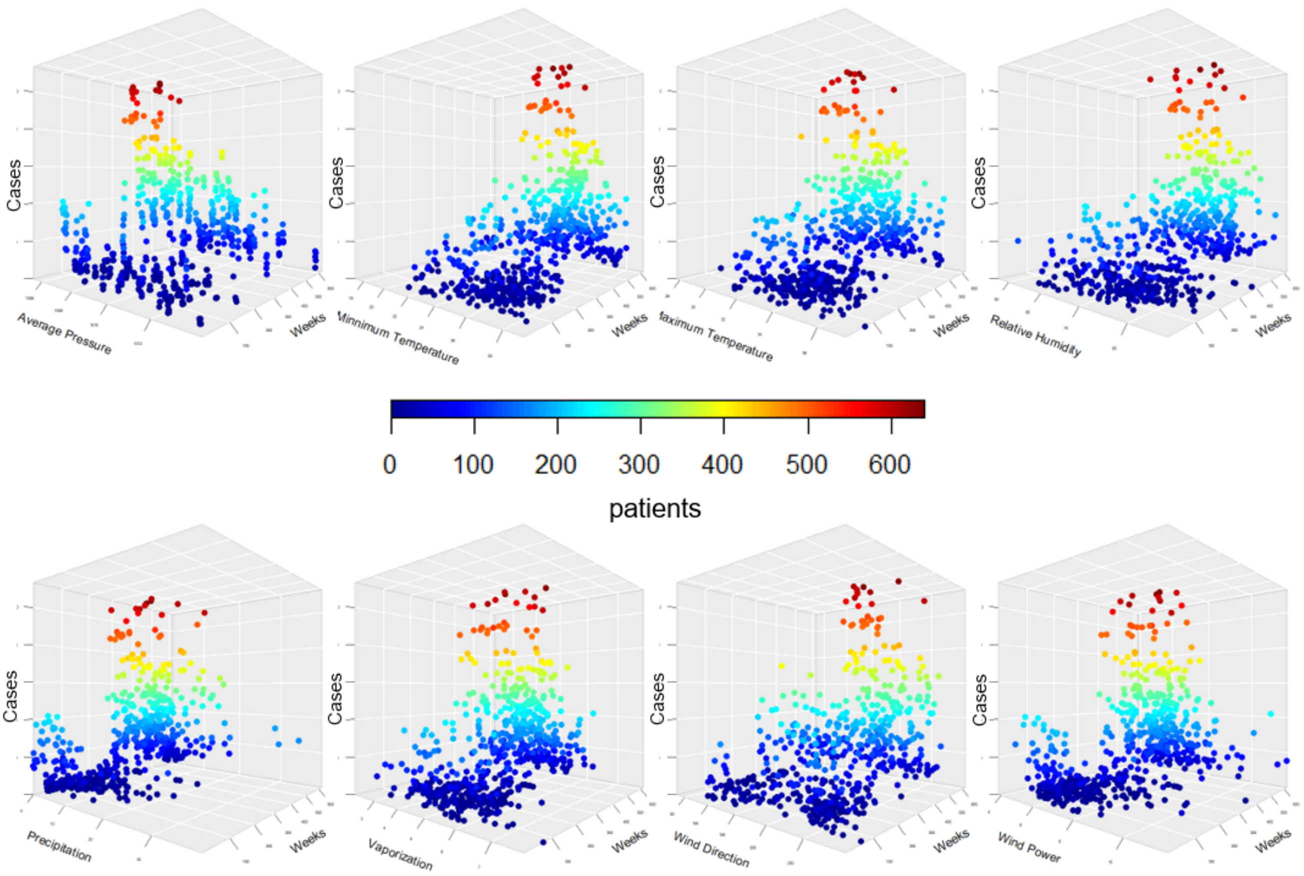

Figure 51: Three-dimensional scatter plot between dengue incidences and weather effects starting from January 2001 to December 2013 of Chonburi.

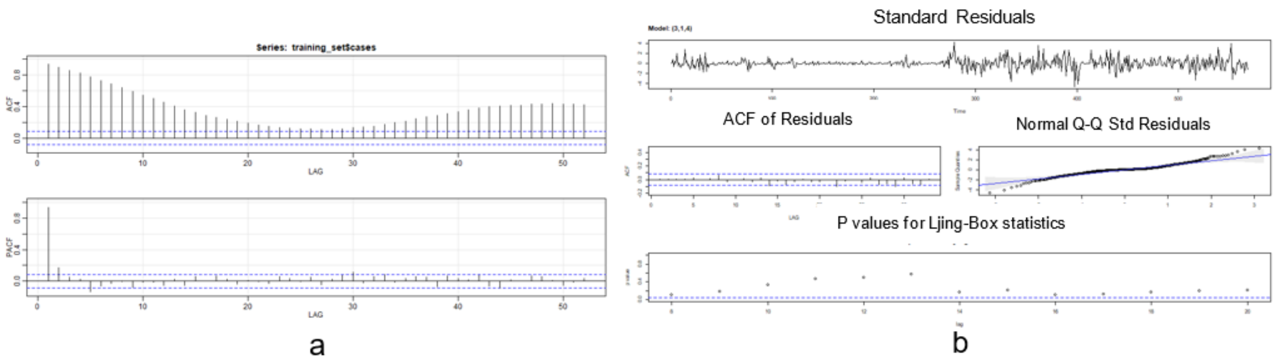

Figure 52: (a) Two plots between lag-time of dengue incidences and ACF and PACF relationship calculated from ARIMA model (b) Summary plots of time series analysis, multiple plots include the plot of predicted model over the time, the plot of ACF residual over lag-time of dengue incidences, residual Q-Q plot of standard residual, and p-value for Ljung-Box statistics of PACF relationship in Chonburi over the training data starting from January 2001 to December 2013.

For Chon buri, the best model is based on quasi-likelihood method. The correlation coefficient on the test set in 2014 is 0.789 (95%CI: 0.72631, 0.8614). The model consists of 8 variables. The most significant variable is 1-week-lag cases, following by 3-week-lag cases, current week maximum temperature and current week wind direction. Other variables which have less significant are, 1-week-lag and 3-week-lag precipitation, 1-week-lag vaporization, and 1- week-lag wind power. Time series methods by ARIMA and SARIMA yield the correlation coefficient of -0.2876191 and -35.81589 respectively.

Table 17: Comparison table of all methods by the highest correlation coefficient (R^2) and the lowest prediction error (RMSE) in Chonburi.

**Methods**	**R-squared (R^2)**	**Root mean square error (RMSE)**
Poisson Regression	0.6371511	12.12885
Negative Binomial Regression	0.1490683	18.57394
Quasi-likelihood Regression	0.7894088	9.240104
ARIMA (3,1,4)	-0.2876191	5.991899
SARIMA $(2,0,1)(0,2,0)_{52}$	-35.81589	32.0397

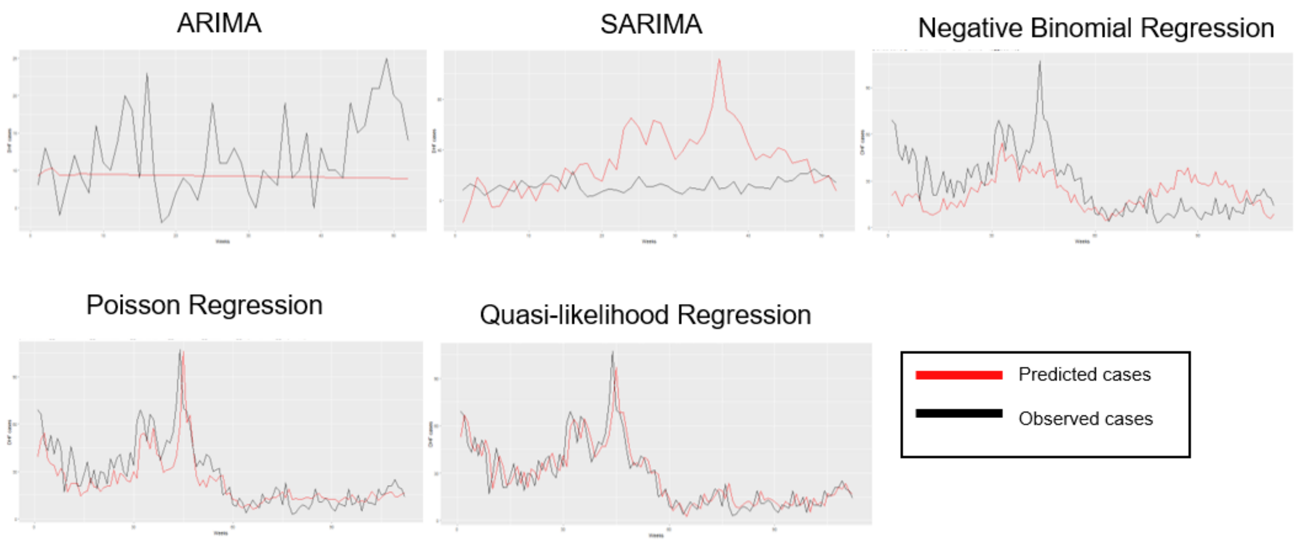

Figure 53: Plots between dengue cases and weeks, the black line represents the observed dengue cases, and the red line represents the predicted dengue cases of the best fit model of each technique over the test set data starting from January 2014 to December 2014.

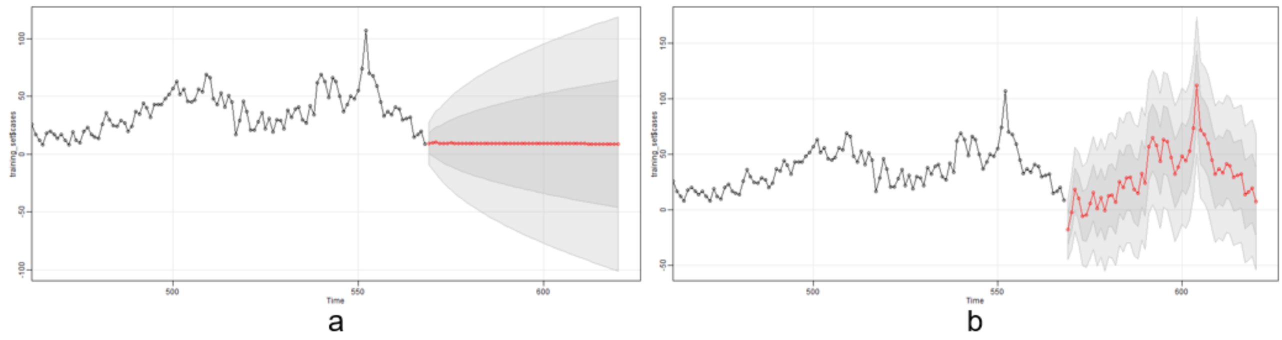

Figure 54: (a) Plot between dengue incidences over weekly time by the best model of ARIMA and (b) SARIMA time series analysis, the black line represents training set data starting from January 2012 to December 2013, and the red line represents the forecasted dengue incidences from January 2014 to December 2014.

Table 18: Coefficients and significant values of best fit GLM models, Negative Binomial, Poisson and Quasi-likelihood regression model of chonburi. The table summarizes coefficients of each independent variables which are composed in best fit model of each method. The significant of each variable is labelled by asterisks under the coefficients. The most important factor is marked as three asterisks which p-value ranges from 0 to 0.001. The second important factor is marked as two asterisks which p-value ranges from 0.001 to 0.01. The third important factor is marked as an asterisk which p-value ranges from 0.01 to 0.1. The least important is also marked as a dot which p-value ranges from 0.1 to 1.

**Independent variables**	**Lag**	**Coefficients/Significant**		
		NB	Poisson	Quasi
Intercept		218.427538 ***	-3.4148510 ***	-33.439279 **
Cases	1		0.0209519 ***	0.835284 ***
	2			
	3		0.0029085 ***	0.109129 **
Average Pressure	0	-0.216901 ***		
	1			
	2			
	3			
Minimum Temperature	0	-0.147361 *		
	1			
	2			
	3			
Maximum Temperature	0	0.088350		0.965367 **
	1			
	2		0.1009298 ***	
	3			
Relative Humidity	0	0.025870 *		
	1			
	2		0.0215892 ***	
	3	0.028978 **		
Precipitation	0			
	1		-0.0057095 **	-0.049877
	2			
	3			0.072607
Vaporization	0			
	1			-0.289207
	2			
	3			
Wind Direction	0	0.001202		0.010154 .
	1			
	2			
	3			
Wind Power	0			
	1		0.0372977 ***	0.290924
	2		0.0649010 ***	
	3			

Chumphon

Chumphon is located in southern Thailand at 10°29′38″N 99°10′48″E. Chumphon covers an area of 6,009 km^2. Total population are 500,575 people. The density of population is 83.3 people per km^2. Weather in Chumphon has tropical rainforest climate system. Temperature in Chumphon has the highest of 38.8°C in April and the lowest of 13.0°C in December. Chumphon has a dry season that runs from December through March and a wet season. Precipitation occurs from mid-May to August. Rainfall are roughly 287.9 mm in November. Humidity presents in the range of 78-84 percent throughout the year. March is the highest sunshine hours.

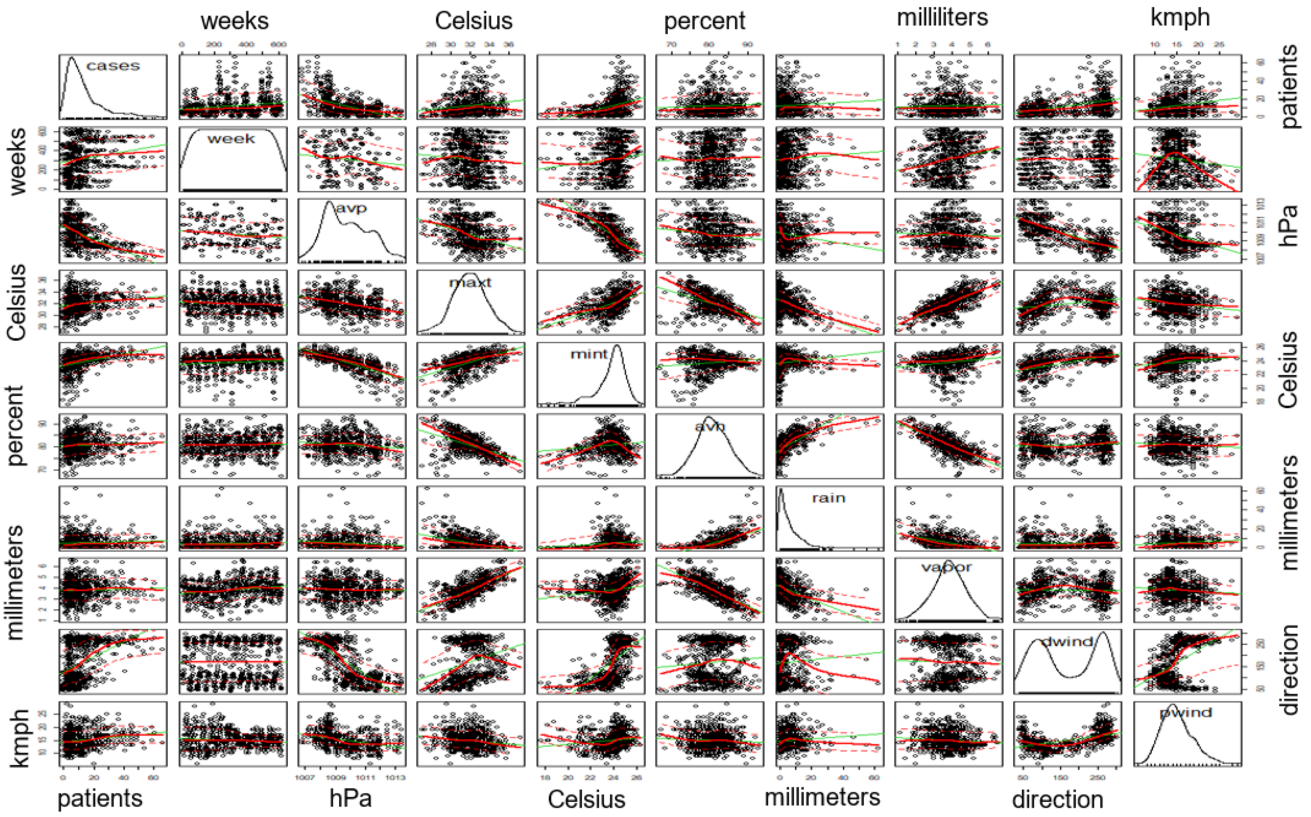

Figure 55: Scatter plot between dengue cases (cases) and selected independent variables, which are the weekly period starting from January 2001 – December 2013 (week), average pressure (avp), maximum temperature (maxt), minimum temperature (mint), average humidity (avh), precipitation (rain), vaporization of water (vapor), wind direction (dwind), and wind power (pwind). The plot visualizes pairwise hundred relationships of training set in Chumphon.

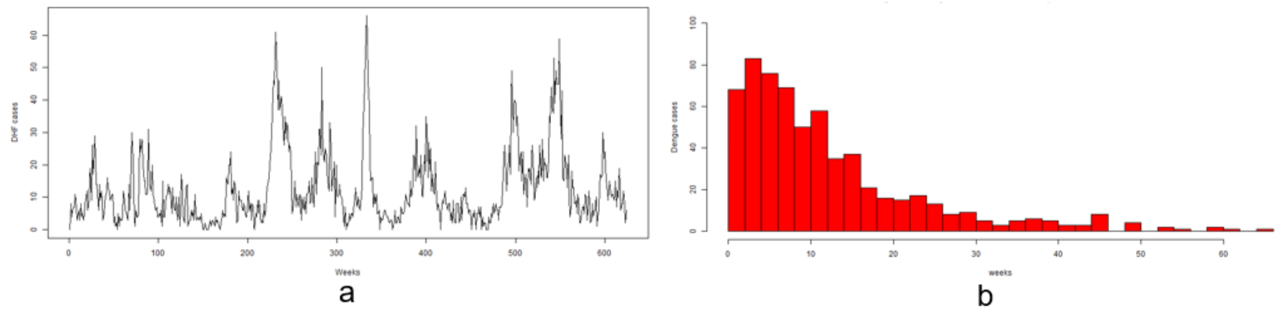

Figure 56: (a) Line plot between dengue incidences and weeks, the plot shows trends of dengue incidences in each year as stationary time series. (b) Histogram of dengue incidences in Chumphon starting from January 2001 to December 2013 (624 weeks).

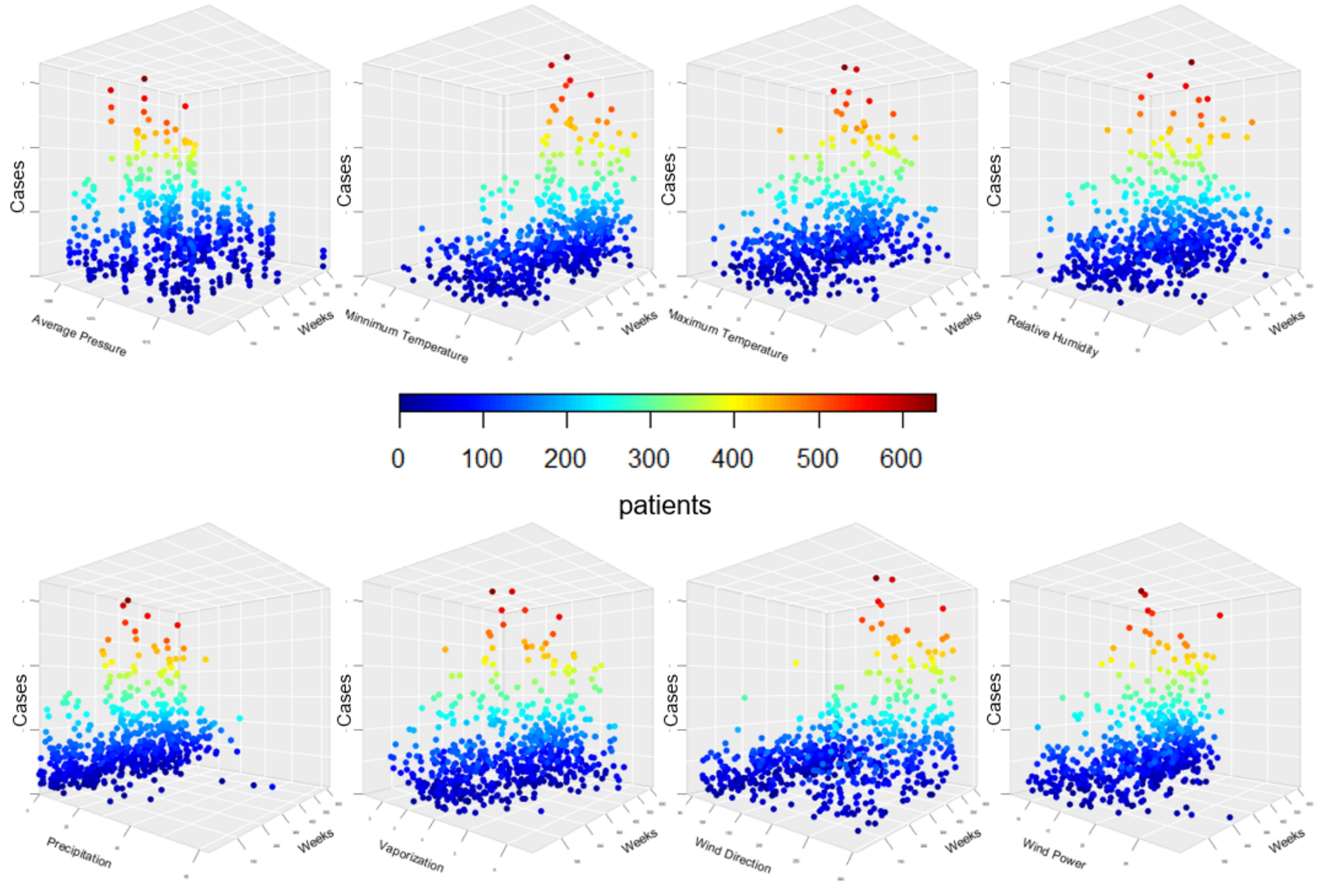

Figure 57: Three-dimensional scatter plot between dengue incidences and weather effects starting from January 2001 to December 2013 of Chumphon.

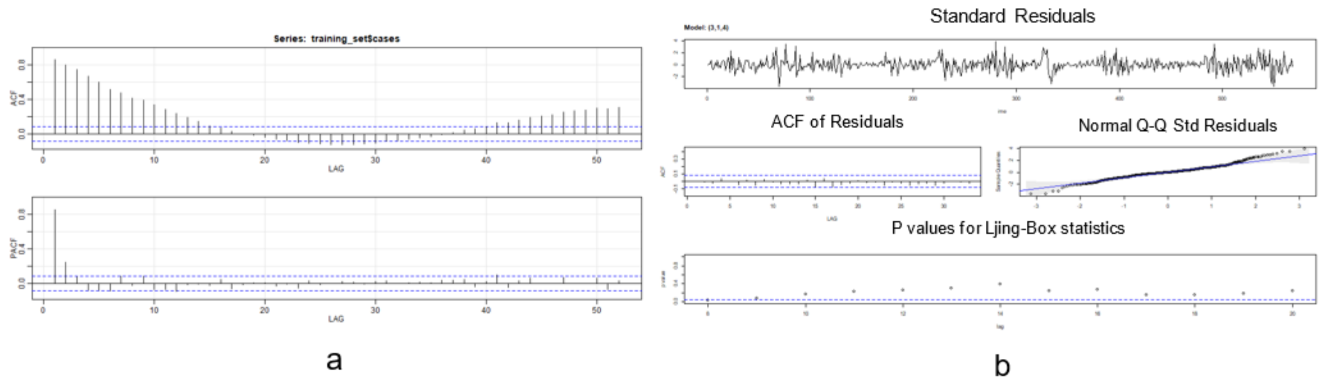

Figure 58: (a) Two plots between lag-time of dengue incidences and ACF and PACF relationship calculated from ARIMA model (b) Summary plots of time series analysis, multiple plots include the plot of predicted model over the time, the plot of ACF residual over lag-time of dengue incidences, residual Q-Q plot of standard residual, and p-value for Ljung-Box statistics of PACF relationship in Chumphon over the training data starting from January 2001 to December 2013.

The best model of Chumphon is based on Poisson regression method. The correlation coefficient on the test set in 2014 is 0.618 (95%CI: 0.5953, 0.8276). The significant of the variables associated with p-value statistical calculation are shown in Table 19. The best model of Chumphon uses 10 variables. The most significant variable are 1-week-lag cases, current week average pressure, following by 1-week-lag and 2-week-lag average pressure. Other variables which has less significant are, 1-week-lag and 2-week-lag precipitation, 1-week-lag, 2-week-lag and 3-week-lag wind direction, and 1-week-lag wind power. Time series methods by ARIMA and SARIMA yield the correlation coeffect of -0.00705413 and -7.57398 respectively.

Table 19: Comparison table of all methods by the highest correlation coefficient (R^2) and the lowest prediction error (RMSE) in Chumphon.

**Methods**	**R-squared (R^2)**	**Root mean square error (RMSE)**
Poisson Regression	0.6177131	3.665905
Negative Binomial Regression	0.5991986	3.753626
Quasi-likelihood Regression	0.5466187	3.992255
ARIMA (3,1,4)	-0.00705413	5.949943
SARIMA $(2,0,1)(0,2,0)_{52}$	-7.57398	17.36112

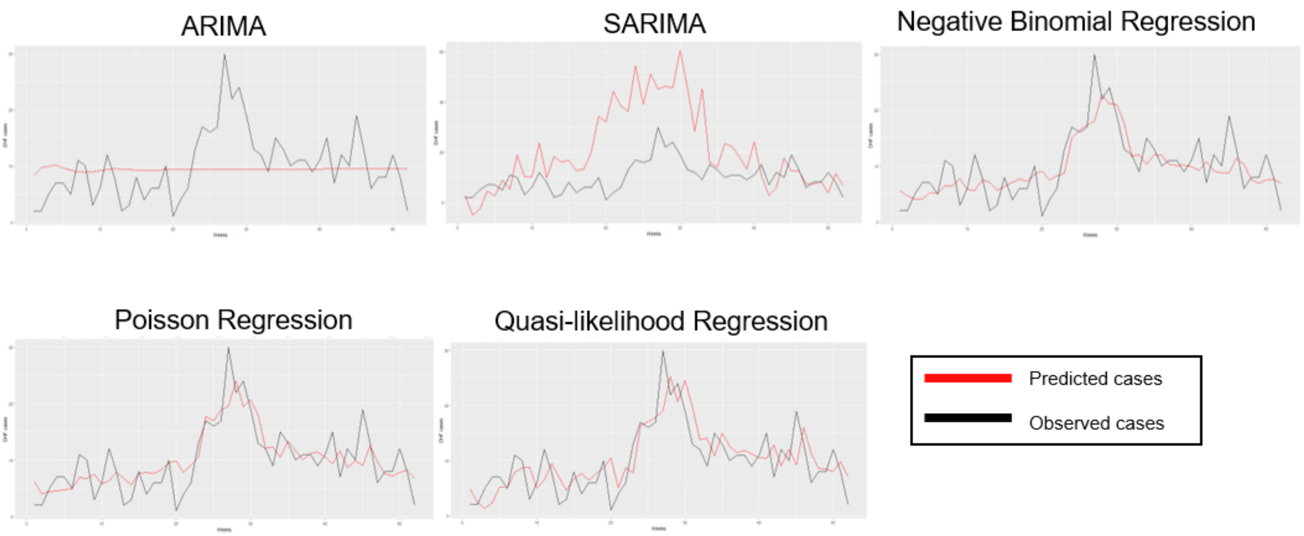

Figure 59: Plots between dengue cases and weeks, the black line represents the observed dengue cases, and the red line represents the predicted dengue cases of the best fit model of each technique over the test set data starting from January 2014 to December 2014.

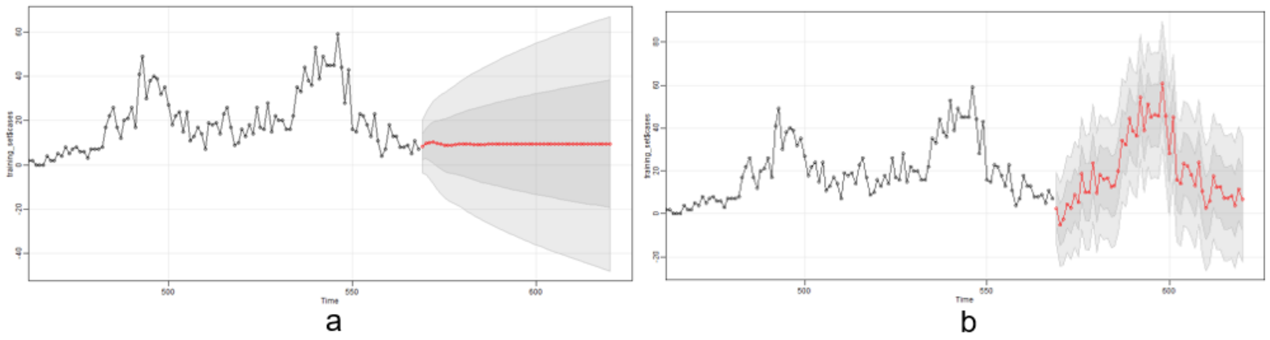

Figure 60: (a) Plot between dengue incidences over weekly time by the best model of ARIMA and (b) SARIMA time series analysis, the black line represents training set data starting from January 2012 to December 2013, and the red line represents the forecasted dengue incidences from January 2014 to December 2014.

Table 20: Coefficients and significant values of best fit GLM models, Negative Binomial, Poisson and Quasi-likelihood regression model of Chumphon. The table summarizes coefficients of each independent variables which are composed in best fit model of each method. The significant of each variable is labelled by asterisks under the coefficients. The most important factor is marked as three asterisks which p-value ranges from 0 to 0.001. The second important factor is marked as two asterisks which p-value ranges from 0.001 to 0.01. The third important factor is marked as an asterisk which p-value ranges from 0.01 to 0.1. The least important is also marked as a dot which p-value ranges from 0.1 to 1.

**Independent variables**	**Lag**	**Coefficients/Significant**		
		NB	Poisson	Quasi
Intercept		91.8392555 **	126.0 ***	814.350538 *
Cases	1	0.0341081 ***	0.03589 ***	0.670290 ***
	2			
	3			0.170551 ***
Average Pressure	0	-0.1036930 *	-0.1508 ***	-1.332421 *
	1	0.0573652	0.09359 *	0.852604
	2	-0.0414711	-0.06531 *	-0.302654
	3			
Minimum Temperature	0			-0.080396
	1			
	2			
	3			
Maximum Temperature	0			
	1			
	2			
	3			
Relative Humidity	0			
	1	-0.0134603 .		-0.164468 .
	2			
	3	-0.0031839		-0.072475
Precipitation	0			
	1	0.008200 .	0.002420	0.085544 .
	2	0.0020440	0.002655	0.022117
	3			0.015078
Vaporization	0			
	1			
	2			
	3			
Wind Direction	0	0.0003751		0.003161
	1	0.0002994	0.0003305	
	2		0.0004619 .	
	3	0.0003087	-0.009362	
Wind Power	0	-0.0127870		-0.112647
	1	-0.0022736	-0.003163	-0.043955
	2	-0.0088409		
	3			

Kalasin

Kalasin is located in the northeastern continent of Thailand at coordinate of 16°26′3″N 103°30′33″E. Kalasin covers an area of 6,947 km^2. Total population are 984,907 people. The density of population is approximately 142.0 people per km^2. Weather in Kalasin has tropical savanna climate under the South Asian monsoon system. Temperature is high in May approximately 42.3 °C and low temperatures are from December to February (5.5-10.2 °C). Winters are dry and warm. The monsoon season begins from May until October. The highest rainfall presents in August around 266.5 mm. Humidity is in range from 65-83 percent throughout the year. The highest sunshine hours are in January and March.

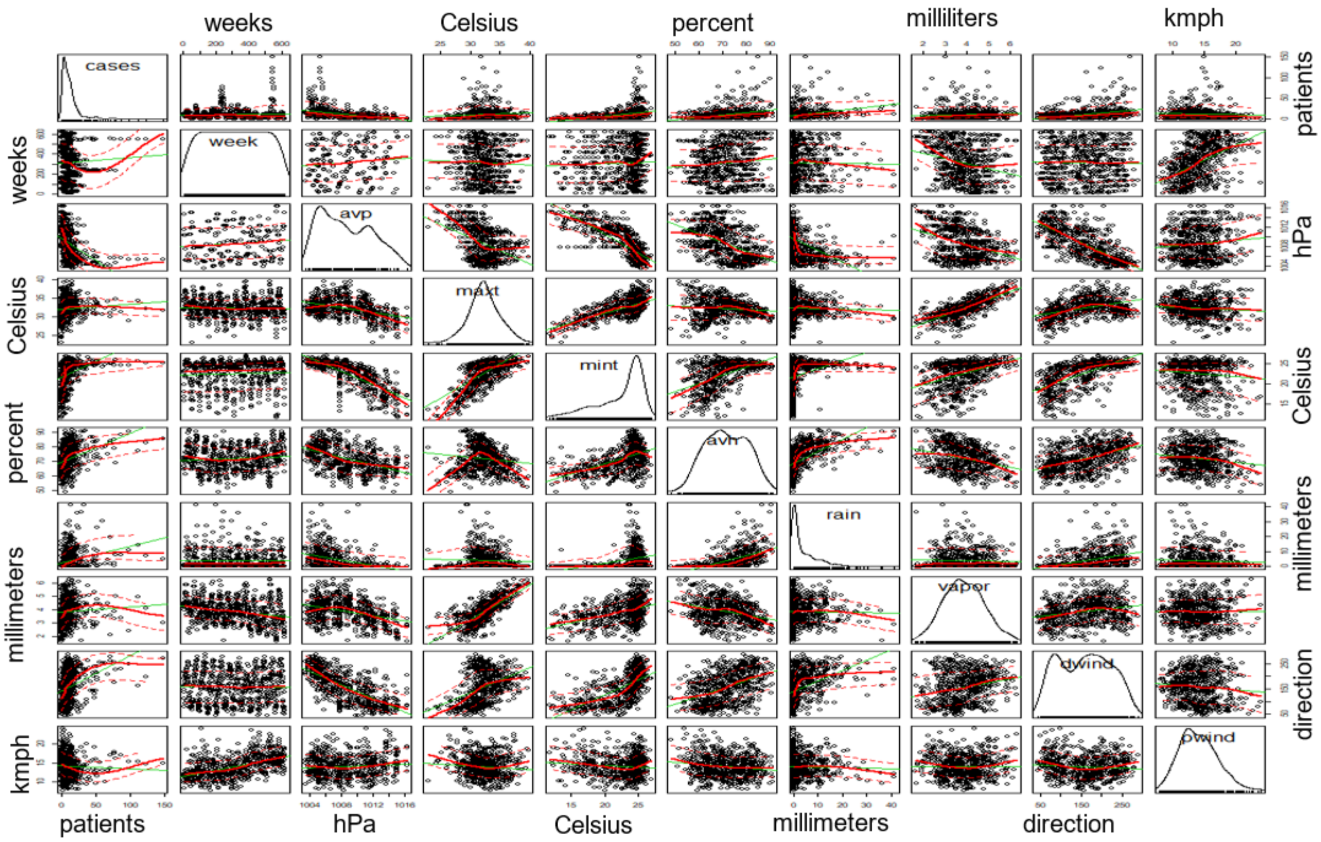

Figure 61: Scatter plot between dengue cases (cases) and selected independent variables, which are the weekly period starting from January 2001 – December 2013 (week), average pressure (avp), maximum temperature (maxt), minimum temperature (mint), average humidity (avh), precipitation (rain), vaporization of water (vapor), wind direction (dwind), and wind power (pwind). The plot visualizes pairwise hundred relationships of training set in Kalasin.

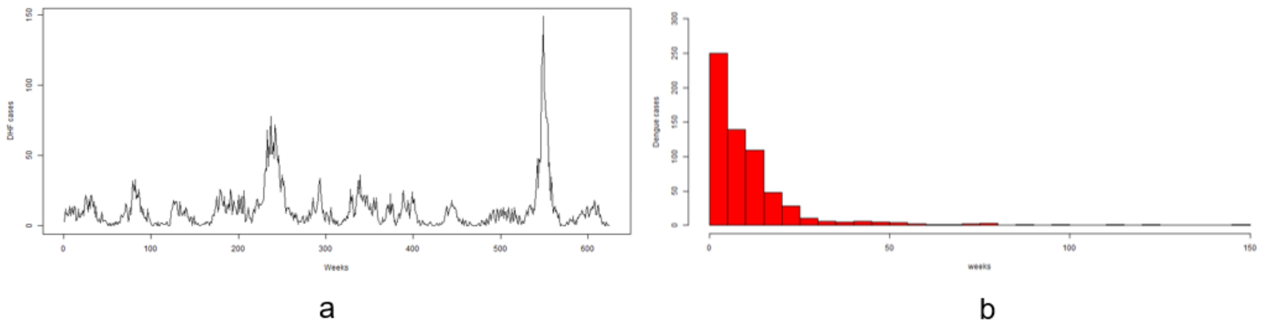

Figure 62: (a) Line plot between dengue incidences and weeks, the plot shows trends of dengue incidences in each year as stationary time series. (b) Histogram of dengue incidences in Kalasin starting from January 2001 to December 2013 (624 weeks).

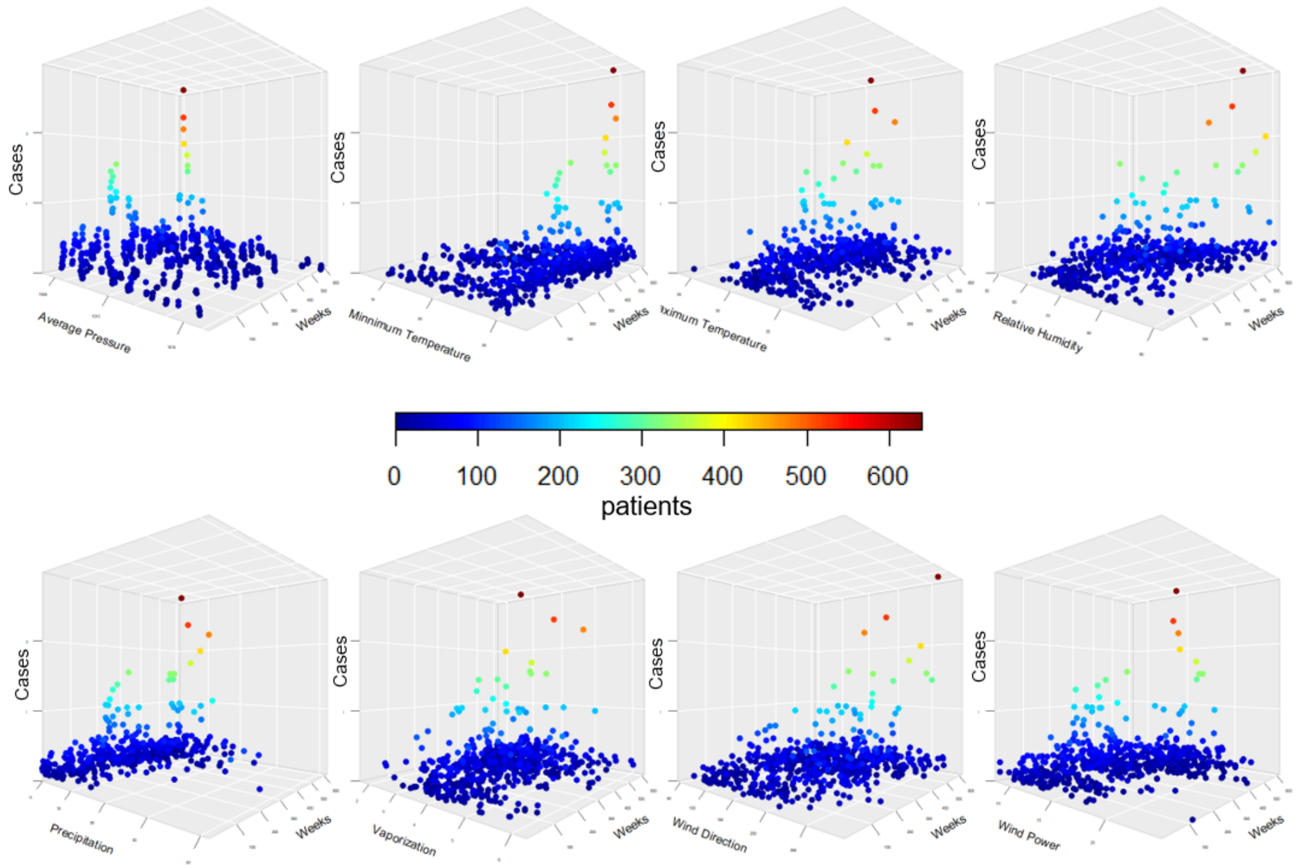

Figure 63: Three-dimensional scatter plot between dengue incidences and weather effects starting from January 2001 to December 2013 of Kalasin.

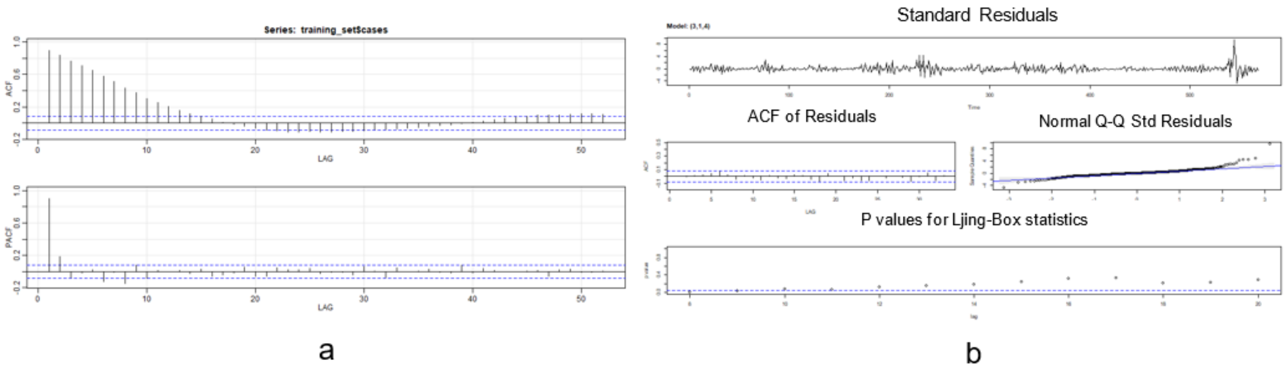

Figure 64: (a) Two plots between lag-time of dengue incidences and ACF and PACF relationship calculated from ARIMA model (b) Summary plots of time series analysis, multiple plots include the plot of predicted model over the time, the plot of ACF residual over lag-time of dengue incidences, residual Q-Q plot of standard residual, and p-value for Ljung-Box statistics of PACF relationship in Kalasin over the training data starting from January 2001 to December 2013.

For Kalasin, the best model is based on negative binomial method. The correlation coefficient on the test set in 2014 is 0.708 (95%CI: 0.6775, 0.8202). The best model of Kalasin uses 8 variables. The most significant variable is 1-week-lag cases, following by 3-week-lag cases and 3-week-lag wind power. Other variables in the model are, 1-week-lag and 3-week-lag average pressure, 2-week-lag and 3-week-lag wind direction, and current week wind power. Time series methods by ARIMA and SARIMA yield the correlation coefficient of -0.7371941 and -76.17176 respectively.

Table 21: Comparison table of all methods by the highest correlation coefficient (R^2) and the lowest prediction error (RMSE) in Kalasin.

Methods	R-squared (R^2)	Root mean square error (RMSE)
Poisson Regression	0.6751769	2.734948
Negative Binomial Regression	0.7081896	2.592245
Quasi-likelihood Regression	0.6547423	2.819664
ARIMA (3,1,4)	-0.7371941	6.324844
SARIMA $(2,0,1)(0,2,0)_{52}$	-76.17176	42.15556

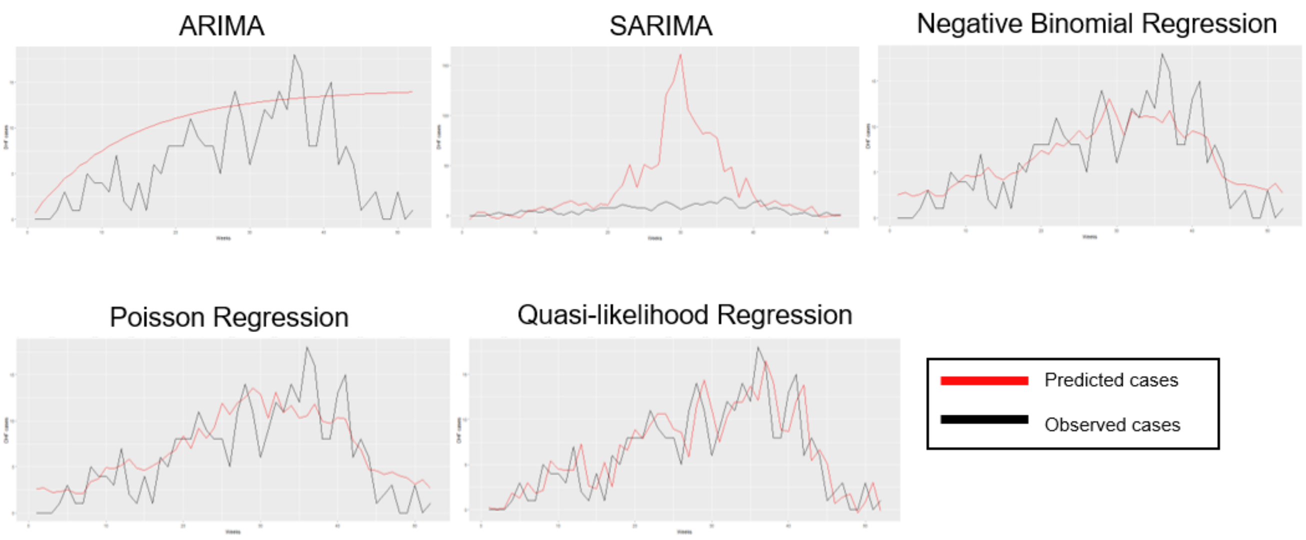

Figure 65: Plots between dengue cases and weeks, the black line represents the observed dengue cases, and the red line represents the predicted dengue cases of the best fit model of each technique over the test set data starting from January 2014 to December 2014.

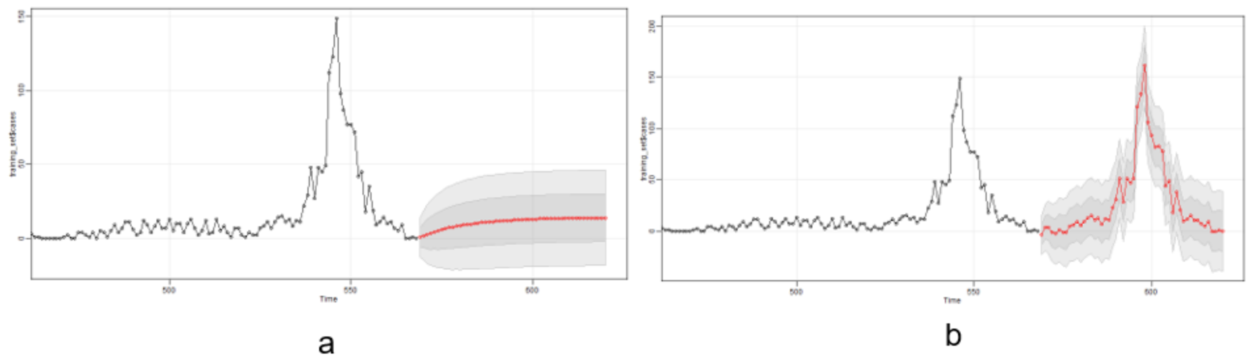

Figure 66: (a) Plot between dengue incidences over weekly time by the best model of ARIMA and (b) SARIMA time series analysis, the black line represents training set data starting from January 2012 to December 2013, and the red line represents the forecasted dengue incidences from January 2014 to December 2014.

Table 22: Coefficients and significant values of best fit GLM models, Negative Binomial, Poisson and Quasi-likelihood regression model of Kalasin. The table summarizes coefficients of each independent variables which are composed in best fit model of each method. The significant of each variable is labelled by asterisks under the coefficients. The most important factor is marked as three asterisks which p-value ranges from 0 to 0.001. The second important factor is marked as two asterisks which p-value ranges from 0.001 to 0.01. The third important factor is marked as an asterisk which p-value ranges from 0.01 to 0.1. The least important is also marked as a dot which p-value ranges from 0.1 to 1.

**Independent variables**	**Lag**	**Coefficients/Significant**		
		NB	Poisson	Quasi
Intercept		74.6792633 ***	130.2 ***	3.22817
Cases	1	0.0325928 ***	0.01906 ***	0.84186 ***
	2			
	3	0.0070481 *	0.004483 ***	0.05307
Average Pressure	0			
	1	-0.0301021	-0.07198 ***	
	2			
	3	-0.0421940 .	-0.05480 ***	
Minimum Temperature	0			
	1			
	2			
	3			
Maximum Temperature	0			
	1			
	2			
	3			
Relative Humidity	0			
	1			-0.02843
	2			-0.02908
	3			
Precipitation	0			
	1			
	2			
	3			
Vaporization	0			
	1			
	2			0.01712
	3			
Wind Direction	0			
	1		-0.0006986 *	
	2	0.0011236	0.0005848 .	0.01460 **
	3	0.0012221 .	0.0007552 *	
Wind Power	0	-0.0126264	-0.005479	
	1		-0.01219 *	-0.01902
	2			
	3	-0.0225431 *	-0.02130 ***	

Kamphaeng Phet

Kamphaeng Phet is located in northern region of Thailand at 16°28′52″N 99°31′20″E. Kamphaeng Phet covers an area of 8,607 km^2. Total population are 729,522 people. The density of population is 85.0 people per km^2. Weather in Kamphaeng Phet has tropical savanna climate under the South Asian monsoon system which controls more tropical wet and dry climate. Temperature is in the range from the low of 10.1°C in December to the high of 43.0°C in April. The rainy season begins with the arrival of the southwest monsoon around mid-May. The humidity presents the average of 76 percent. Precipitation occurs from mid-May to August. The highest precipitation is in September 268.8 mm annually. The longest sunshine hours are in March.

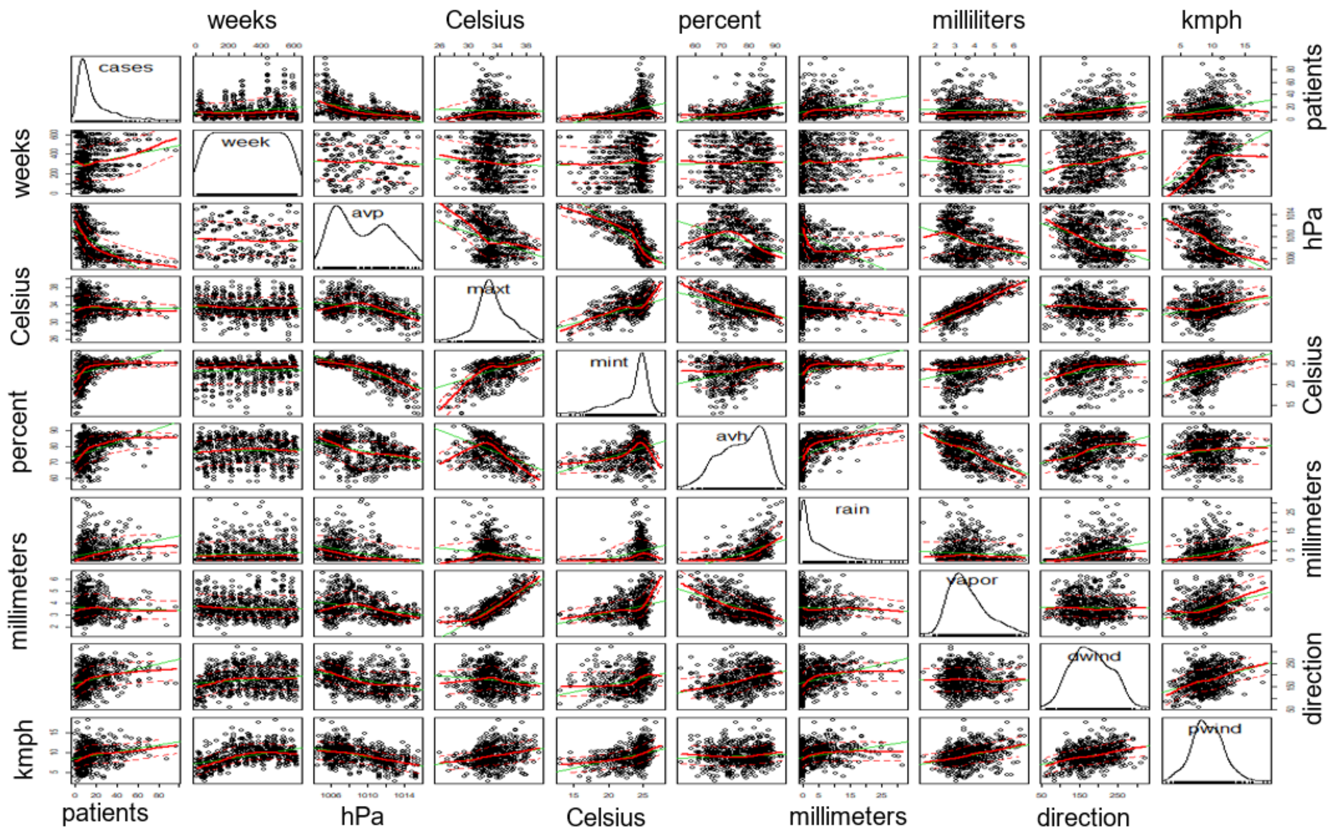

Figure 67: Scatter plot between dengue cases (cases) and selected independent variables, which are the weekly period starting from January 2001 – December 2013 (week), average pressure (avp), maximum temperature (maxt), minimum temperature (mint), average humidity (avh), precipitation (rain), vaporization of water (vapor), wind direction (dwind), and wind power (pwind). The plot visualizes pairwise hundred relationships of training set in Kamphaeng Phet.

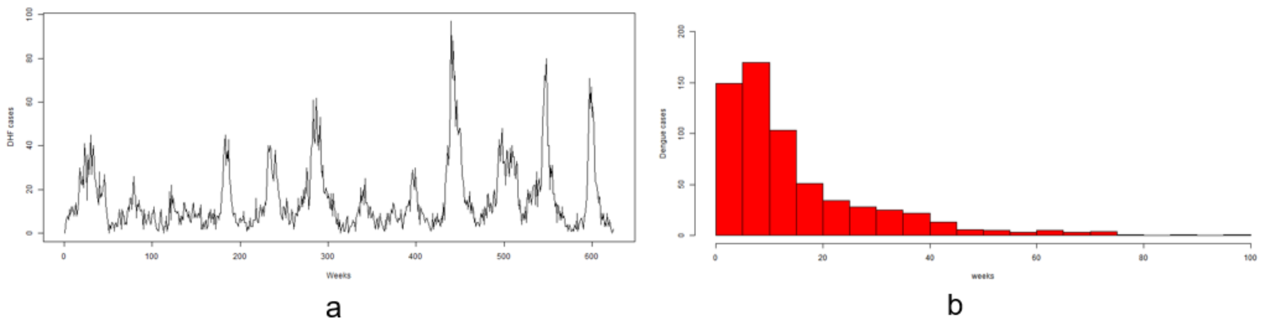

Figure 68: (a) Line plot between dengue incidences and weeks, the plot shows trends of dengue incidences in each year as stationary time series. (b) Histogram of dengue incidences in Kamphaeng Phet starting from January 2001 to December 2013 (624 weeks).

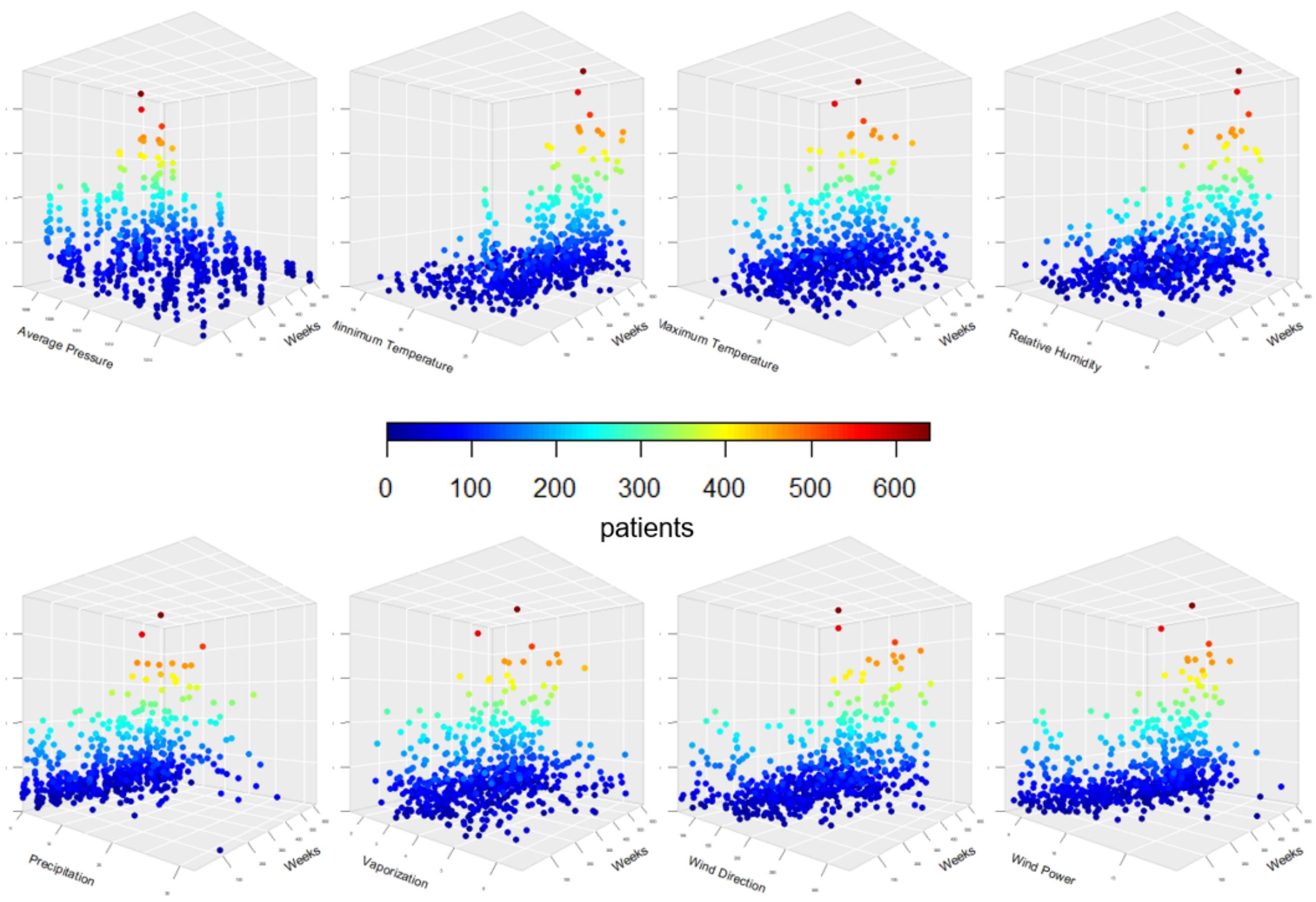

Figure 69: Three-dimensional scatter plot between dengue incidences and weather effects starting from January 2001 to December 2013 of Kamphaeng Phet.

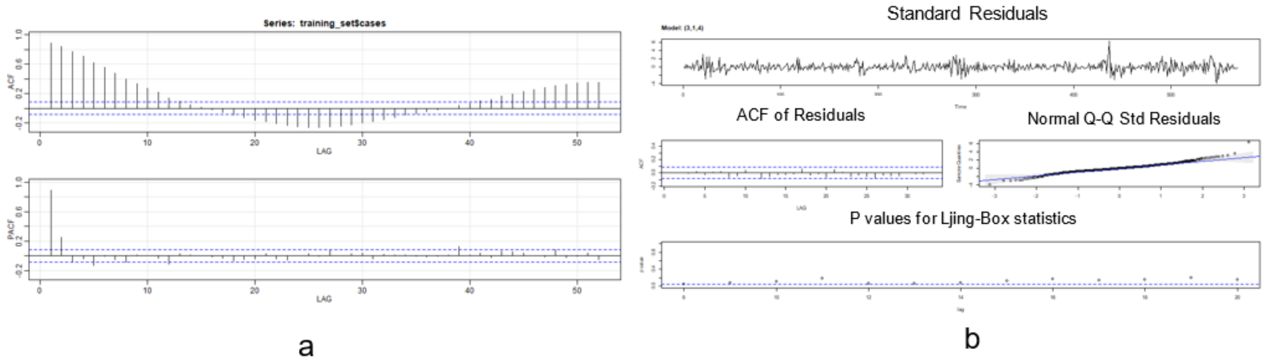

Figure 70: (a) Two plots between lag-time of dengue incidences and ACF and PACF relationship calculated from ARIMA model (b) Summary plots of time series analysis, multiple plots include the plot of predicted model over the time, the plot of ACF residual over lag-time of dengue incidences, residual Q-Q plot of standard residual, and p-value for Ljung-Box statistics of PACF relationship in Kamphaeng Phet over the training data starting from January 2001 to December 2013.

For Kamphaeng Phet, the best model is based on quasi-likelihood method. The correlation coefficient on the test set in 2014 is 0.880 (95%CI: 0.7263, 0.9045). The best model uses 11 variables. The most significant variable is 1-week-lag cases, following by current week average pressure, 3-week-lag average pressure and 1-week-lag relative humidity. Other variables which have less significant are, 2-week-lag average pressure, current week precipitation, 2-week-lag wind direction, current week, 2-week-lag and 3-week-lag wind power. Time series methods by ARIMA and SARIMA yield the correlation coefficient of -0.4554061 and 0.7238176 respectively.

Table 23: Comparison table of all methods by the highest correlation coefficient (R^2) and the lowest prediction error (RMSE) in Kamphaeng Phet.

**Methods**	**R-squared (R^2)**	**Root mean square error (RMSE)**
Poisson Regression	0.8735894	6.671759
Negative Binomial Regression	0.8469747	7.340578
Quasi-likelihood Regression	0.8806165	6.48367
ARIMA (3,1,4)	-0.4554061	22.63814
SARIMA $(2,0,1)(0,2,0)_{52}$	0.7238176	9.861584

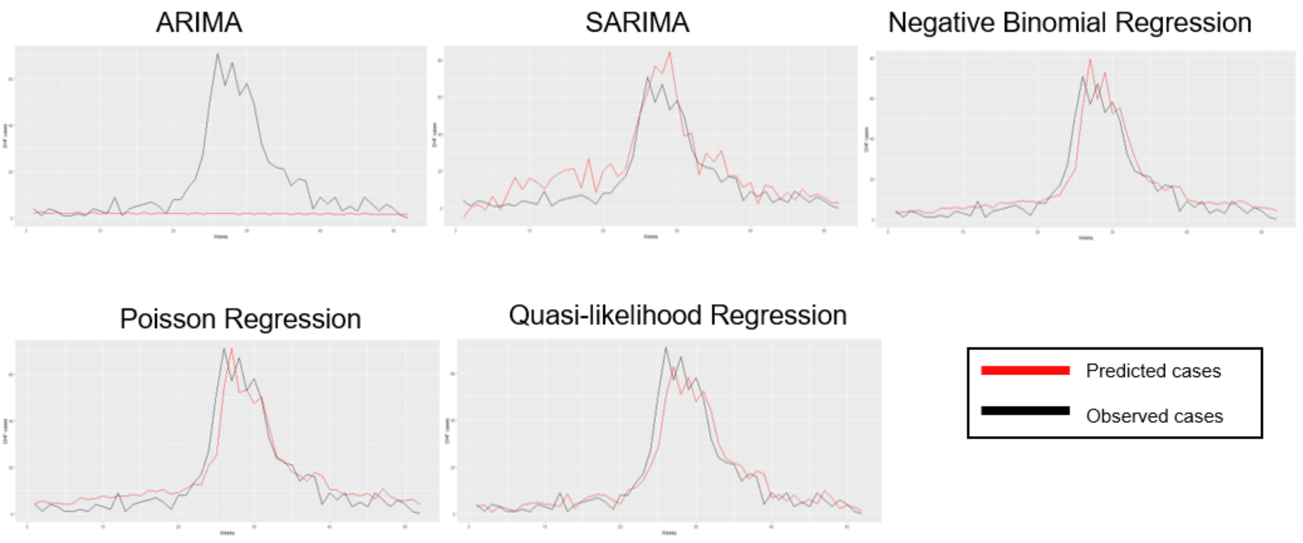

Figure 71: Plots between dengue cases and weeks, the black line represents the observed dengue cases, and the red line represents the predicted dengue cases of the best fit model of each technique over the test set data starting from January 2014 to December 2014.

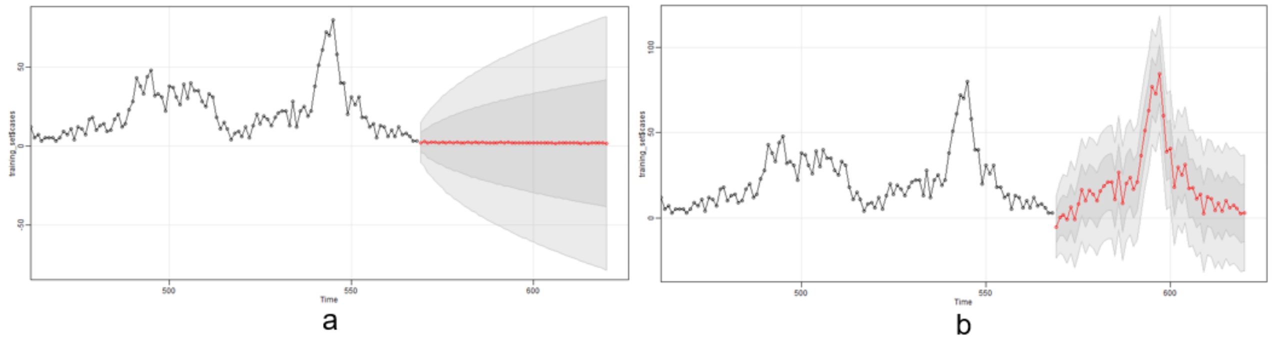

Figure 72: (a) Plot between dengue incidences over weekly time by the best model of ARIMA and (b) SARIMA time series analysis, the black line represents training set data starting from January 2012 to December 2013, and the red line represents the forecasted dengue incidences from January 2014 to December 2014.

Table 24: Coefficients and significant values of best fit GLM models, Negative Binomial, Poisson and Quasi-likelihood regression model of Kamphaeng Phet. The table summarizes coefficients of each independent variables which are composed in best fit model of each method. The significant of each variable is labelled by asterisks under the coefficients. The most important factor is marked as three asterisks which p-value ranges from 0 to 0.001. The second important factor is marked as two asterisks which p-value ranges from 0.001 to 0.01. The third important factor is marked as an asterisk which p-value ranges from 0.01 to 0.1. The least important is also marked as a dot which p-value ranges from 0.1 to 1.

**Independent variables**	**Lag**	**Coefficients/Significant**		
		NB	Poisson	Quasi
Intercept		32.8314578 *	79.3977287 ***	293.407249
Cases	1	0.028077 ***	0.0260527 ***	0.826669 ***
	2			
	3	0.0064953 ***		
Average Pressure	0	-0.0695318 ***	-0.0788274 ***	-0.772303 **
	1			
	2			-0.100438
	3	0.0338099 .		0.575226 .
Minimum Temperature	0			
	1	0.0664330 ***		
	2			
	3			
Maximum Temperature	0		0.0360491 ***	0.014976
	1			
	2		0.0098584	
	3		-0.0194668 *	
Relative Humidity	0			
	1	0.0175361 **	0.0173504 ***	0.094624 .
	2	0.0069891		
	3	0.0112132 *		
Precipitation	0	-0.0046506		-0.005238
	1		-0.0002629	
	2	-0.0006596		
	3			
Vaporization	0			
	1			
	2	0.0508617		
	3			
Wind Direction	0	-0.0002155	-0.0009259 **	
	1		0.0008237 **	
	2	-0.0001629		-0.001489
	3	-0.0004036	-0.0001661	
Wind Power	0		-0.0111386 .	-0.180946
	1			
	2		-0.0022482	0.121417
	3	0.0174389 .	0.0238393 ***	0.292761 .

Kanchanaburi

Kanchanaburi is located in the central region of Thailand at coordinate 14°01′10″N 99°31′52″E. Kanchanaburi covers an area of 19,483 km^2. Total population are 848,198 people. The density of population is 44.0 people per km^2. Weather in Kanchanaburi follows tropical savanna climate system. The highest temperature is in April approximately 43.5°C. The low temperature presents in winter from December to February (9.2-14.1°C). The monsoon season starts from May through August. The highest sunshine hours are in January. Humidity presents around 61-80 percent throughout the year.

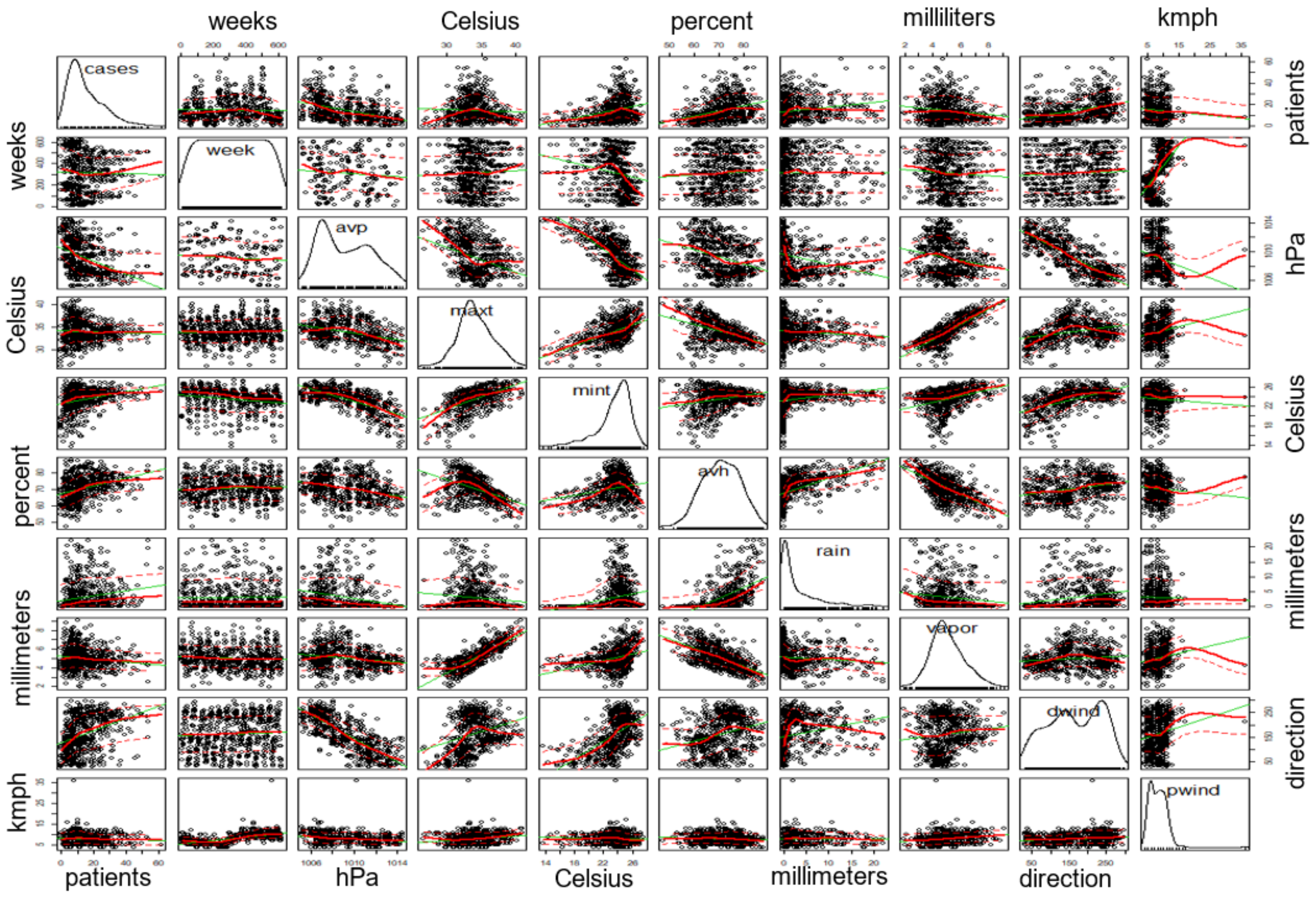

Figure 73: Scatter plot between dengue cases (cases) and selected independent variables, which are the weekly period starting from January 2001 – December 2013 (week), average pressure (avp), maximum temperature (maxt), minimum temperature (mint), average humidity (avh), precipitation (rain), vaporization of water (vapor), wind direction (dwind), and wind power (pwind). The plot visualizes pairwise hundred relationships of training set in Kanchanaburi.

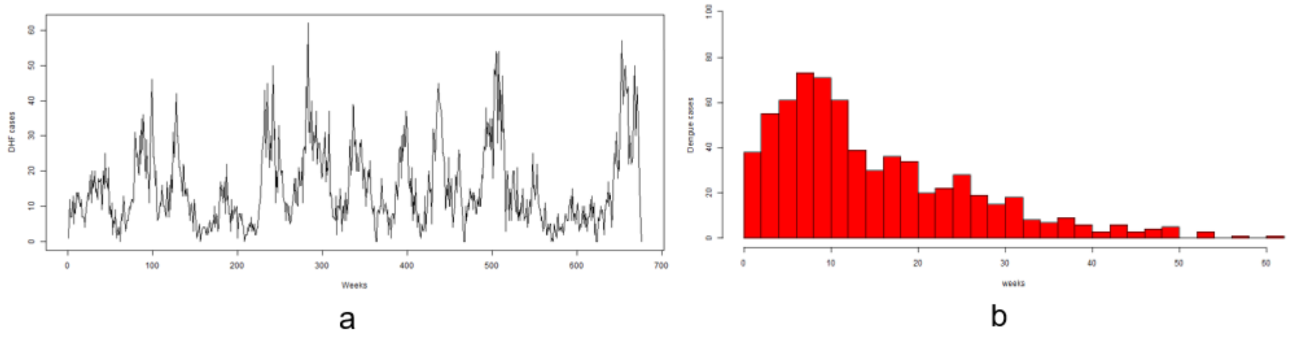

Figure 74: (a) Line plot between dengue incidences and weeks, the plot shows trends of dengue incidences in each year as stationary time series. (b) Histogram of dengue incidences in Kanchanaburi starting from January 2001 to December 2013 (624 weeks).

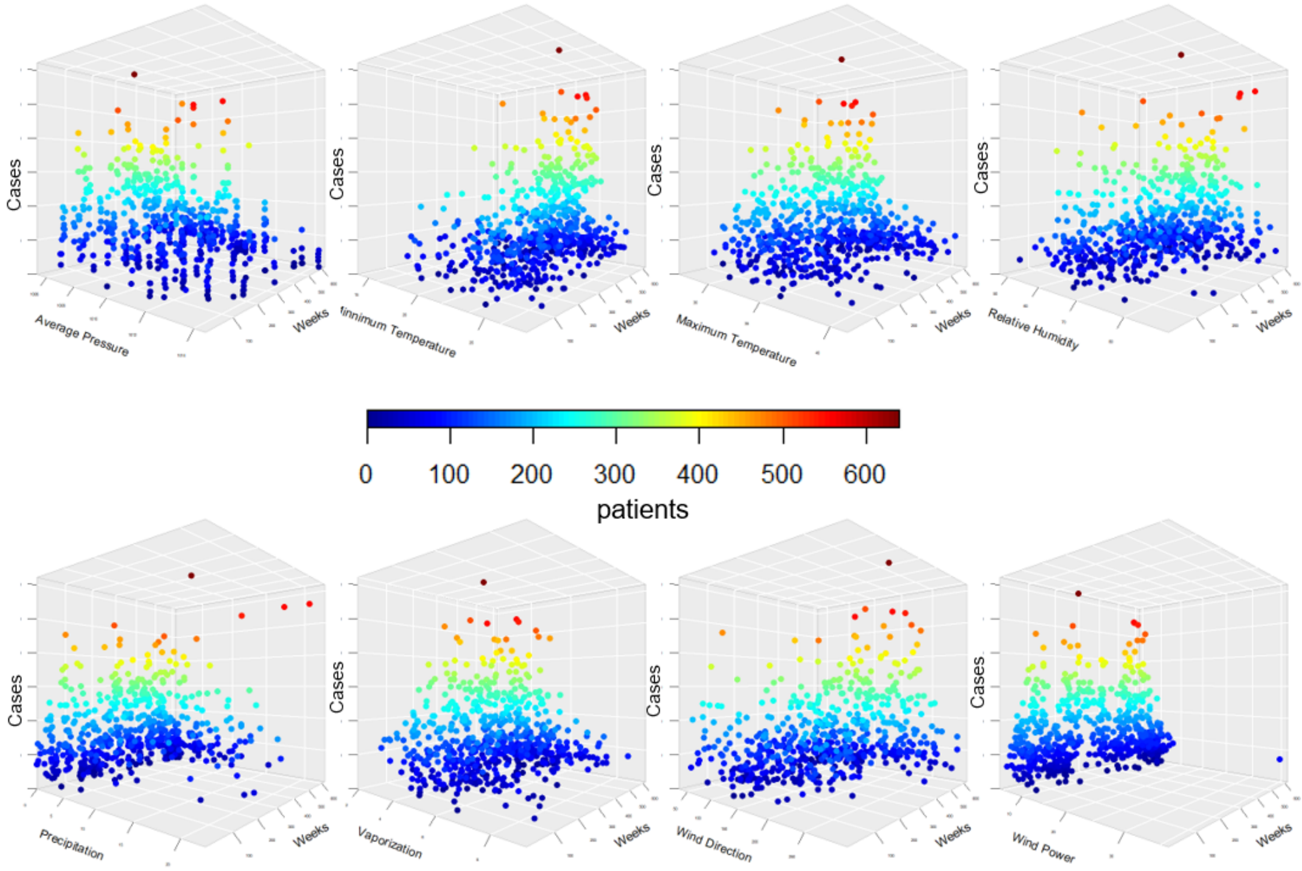

Figure 75: Three-dimensional scatter plot between dengue incidences and weather effects starting from January 2001 to December 2013 of Kanchanaburi.

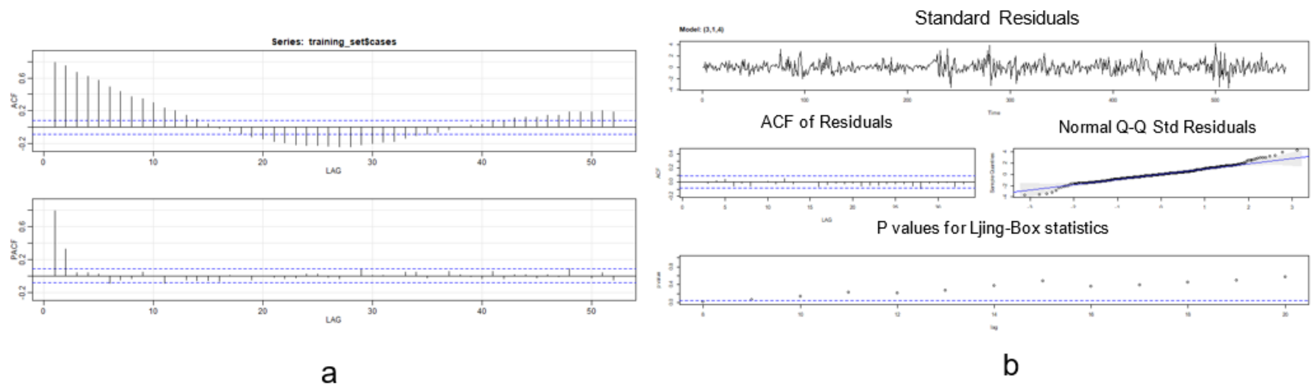

Figure 76: (a) Two plots between lag-time of dengue incidences and ACF and PACF relationship calculated from ARIMA model (b) Summary plots of time series analysis, multiple plots include the plot of predicted model over the time, the plot of ACF residual over lag-time of dengue incidences, residual Q-Q plot of standard residual, and p-value for Ljung-Box statistics of PACF relationship in Kanchanaburi over the training data starting from January 2001 to December 2013.

For Kanchanaburi, the best model is based on Negative Binomial regression method. The correlation coefficient on the test set in 2014 is 0.509 (95%CI: 0.4373, 0.5806). The best model consists of 7 variables. The most significant variables are 1-week-lag cases, 2-week-lag cases and 3-week-lag cases, following by 2-week-lag maximum temperature. Other variables which have less significant are, current week maximum temperature, 3-week-lag wind direction and 3-week-lag wind power. Time series methods by ARIMA and SARIMA yield the correlation coefficient of -2.875803 and -2.849987 respectively.

Table 25: Comparison table of all methods by the highest correlation coefficient (R^2) and the lowest prediction error (RMSE) in Kanchanaburi.

Methods	R-squared (R^2)	Root mean square error (RMSE)
Poisson Regression	0.4822253	5.125198
Negative Binomial Regression	0.5089173	4.991344
Quasi-likelihood Regression	0.4974719	5.049175
ARIMA (3,1,4)	-2.875803	6.562305
SARIMA $(2,0,1)(0,2,0)_{52}$	-2.849987	6.540413

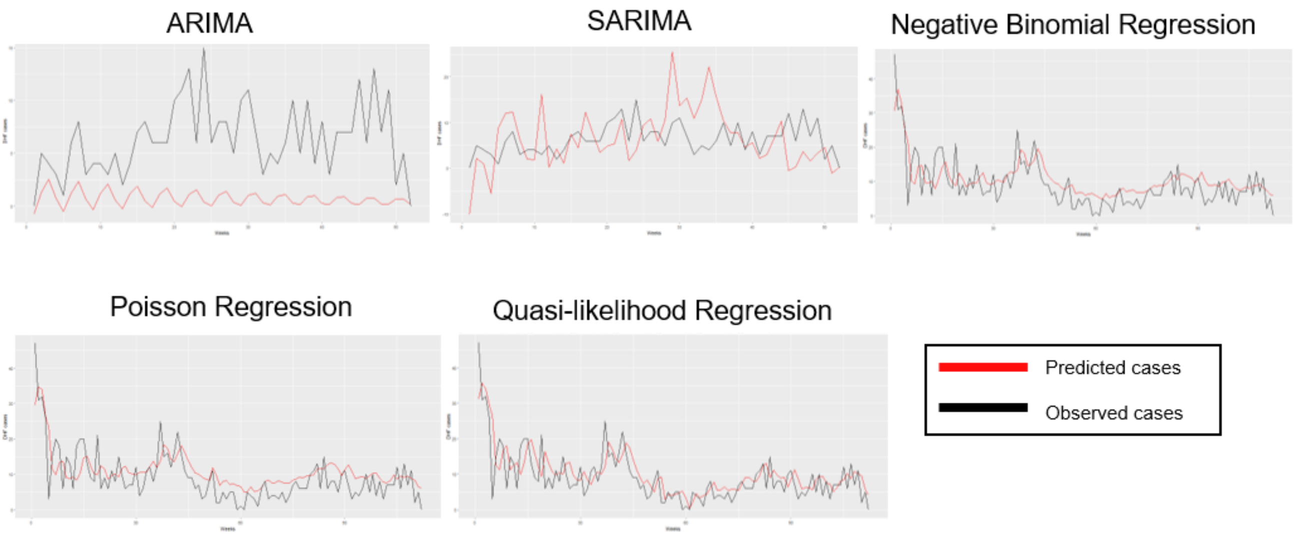

Figure 77: Plots between dengue cases and weeks, the black line represents the observed dengue cases, and the red line represents the predicted dengue cases of the best fit model of each technique over the test set data starting from January 2014 to December 2014.

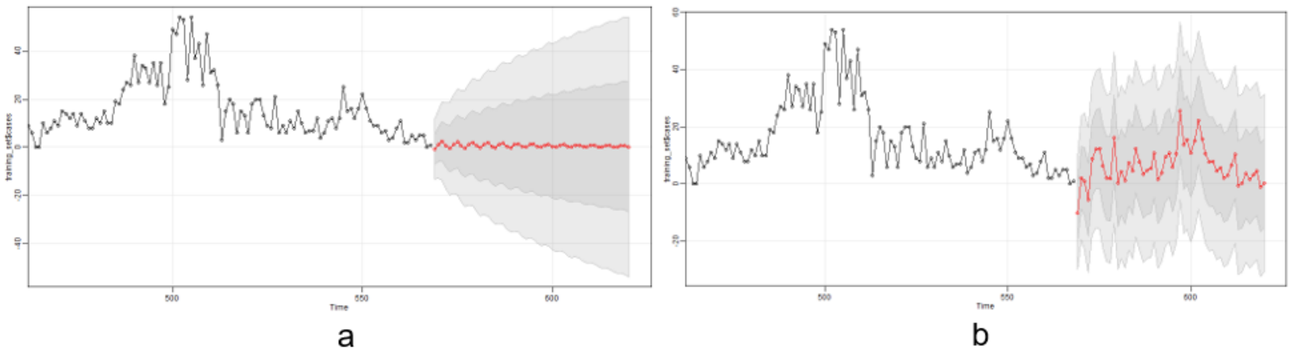

Figure 78: (a) Plot between dengue incidences over weekly time by the best model of ARIMA and (b) SARIMA time series analysis, the black line represents training set data starting from January 2012 to December 2013, and the red line represents the forecasted dengue incidences from January 2014 to December 2014.

Table 26: Coefficients and significant values of best fit GLM models, Negative Binomial, Poisson and Quasi-likelihood regression model of Kanchanaburi. The table summarizes coefficients of each independent variables which are composed in best fit model of each method. The significant of each variable is labelled by asterisks under the coefficients. The most important factor is marked as three asterisks which p-value ranges from 0 to 0.001. The second important factor is marked as two asterisks which p-value ranges from 0.001 to 0.01. The third important factor is marked as an asterisk which p-value ranges from 0.01 to 0.1. The least important is also marked as a dot which p-value ranges from 0.1 to 1.

**Independent variables**	**Lag**	**Coefficients/Significant**		
		NB	Poisson	Quasi
Intercept		55.6146731 ***	66.4777085 ***	-5.18661
Cases	1	0.0279743 ***	0.0238320 ***	0.51637 ***
	2	0.0180969 ***	0.0168304 ***	0.32630 ***
	3			0.03472
Average Pressure	0	-0.0527899 ***	-0.0634912 ***	
	1			
	2			
	3			
Minimum Temperature	0		0.0336182 ***	
	1			
	2			
	3			
Maximum Temperature	0	0.0121851		0.37334 *
	1			
	2	-0.0261014 *	-0.0326199 ***	-0.15240
	3			
Relative Humidity	0			
	1			
	2			
	3			
Precipitation	0			0.07718
	1			
	2			
	3			
Vaporization	0			
	1			
	2			
	3			
Wind Direction	0			
	1		-0.0006196 *	
	2			
	3	0.0005657		
Wind Power	0			
	1		0.0096040	0.06365
	2			
	3	-0.0102275	-0.0103119	-0.13985

Khon Kaen

Khon Kean is the largest province located in the northeastern continent of Thailand at coordinate of 16°26′N 102°50′E. Khon Kean covers an area of 10,886 km^2. Total population are 1,790,049 people. The density of population is approximately 164.0 people per km^2. Weather in Khon Kean has tropical savanna climate under the South Asian monsoon system. Temperature is high in April approximately 42.6°C and low temperatures are from December to January (8.2-11.1°C). Winters are dry and warm. The monsoon season begins from May until October. The highest rainfall presents in September around 232.0 mm. Humidity is in range from 61-83 percent throughout the year. The highest sunshine hours are in January and March.

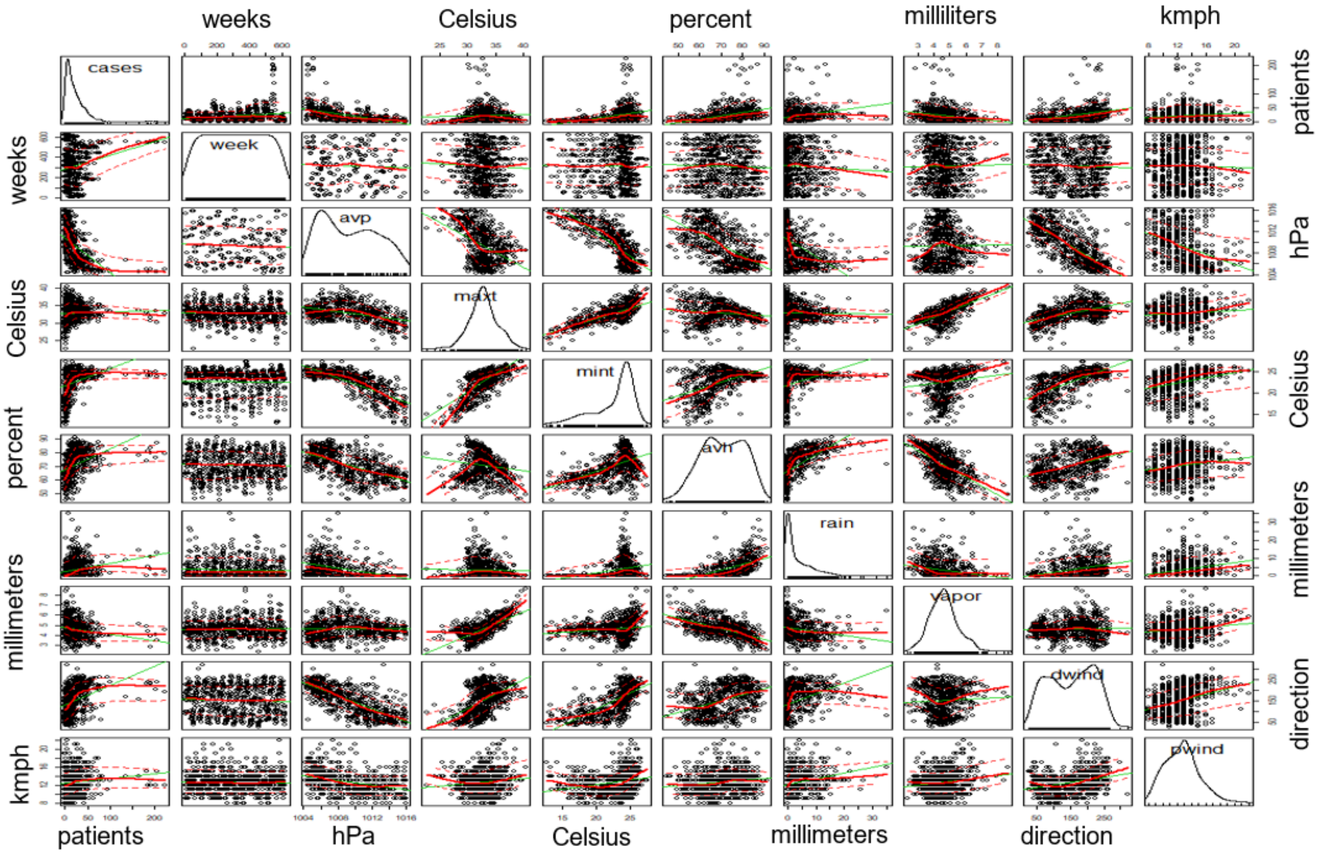

Figure 79: Scatter plot between dengue cases (cases) and selected independent variables, which are the weekly period starting from January 2001 – December 2013 (week), average pressure (avp), maximum temperature (maxt), minimum temperature (mint), average humidity (avh), precipitation (rain), vaporization of water (vapor), wind direction (dwind), and wind power (pwind). The plot visualizes pairwise hundred relationships of training set in Khon Kaen.

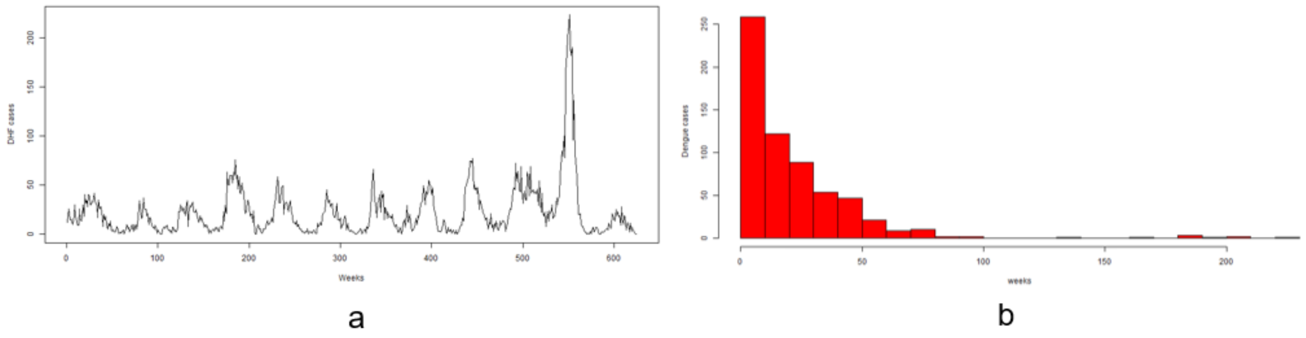

Figure 80: (a) Line plot between dengue incidences and weeks, the plot shows trends of dengue incidences in each year as stationary time series. (b) Histogram of dengue incidences in Khon Kaen starting from January 2001 to December 2013 (624 weeks).

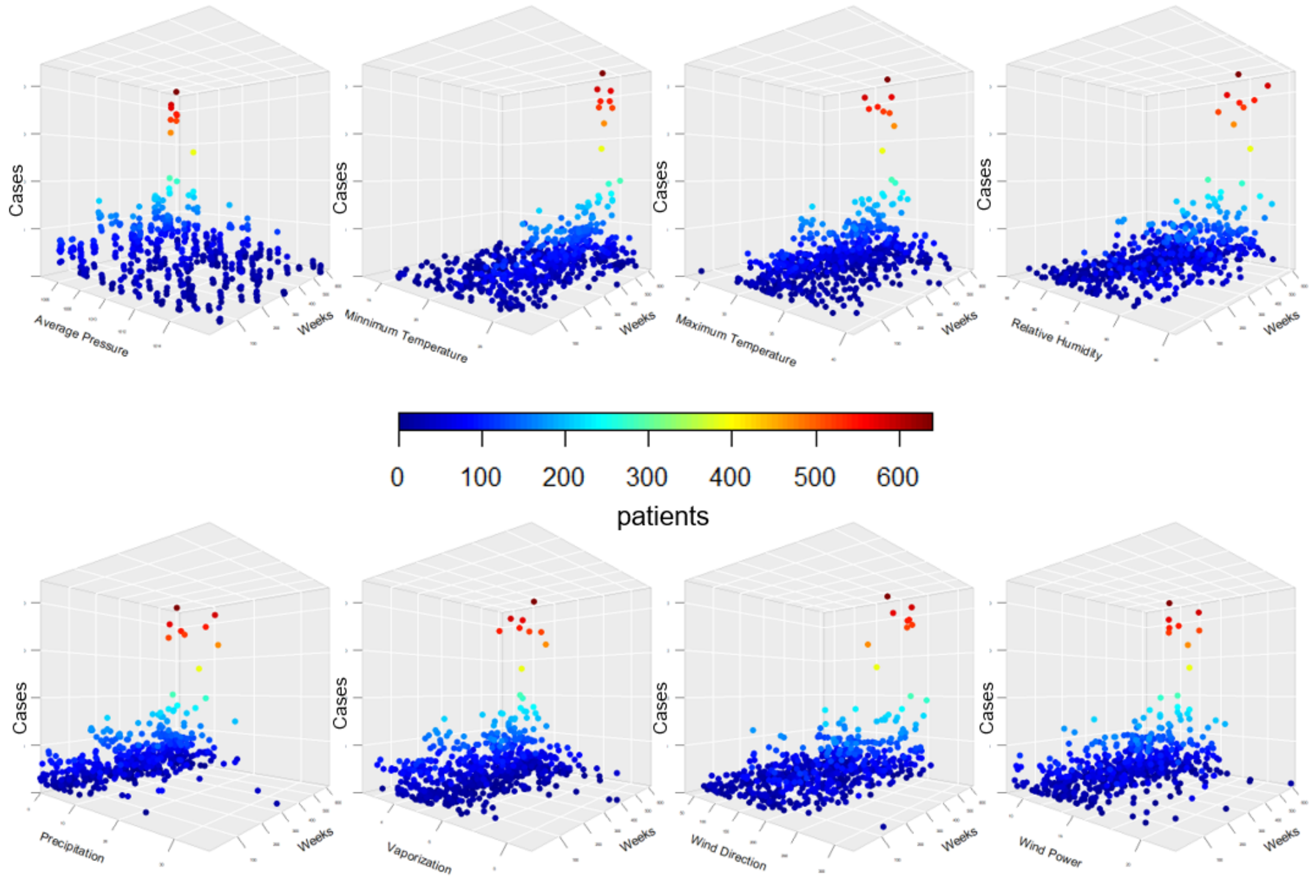

Figure 81: Three-dimensional scatter plot between dengue incidences and weather effects starting from January 2001 to December 2013 of Khon Kaen.

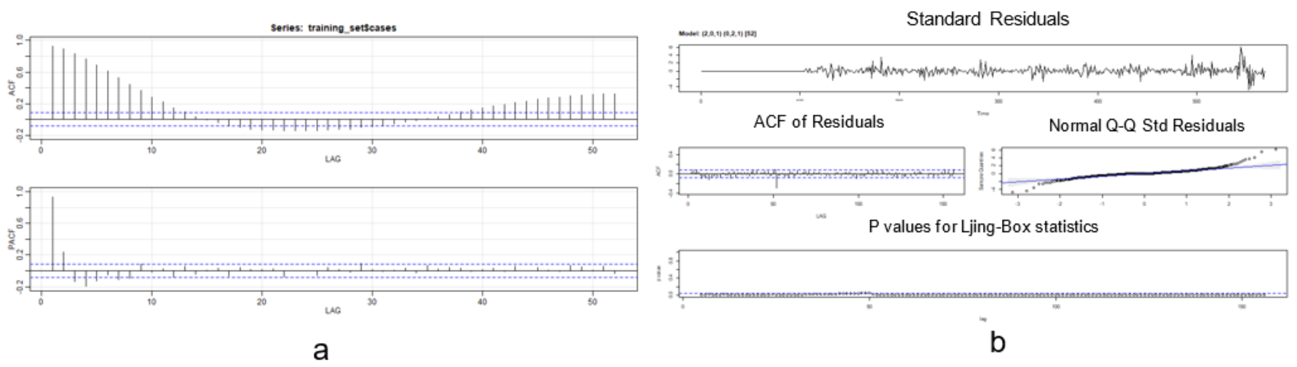

Figure 82: (a) Two plots between lag-time of dengue incidences and ACF and PACF relationship calculated from ARIMA model (b) Summary plots of time series analysis, multiple plots include the plot of predicted model over the time, the plot of ACF residual over lag-time of dengue incidences, residual Q-Q plot of standard residual, and p-value for Ljung-Box statistics of PACF relationship in Khon Kaen over the training data starting from January 2001 to December 2013.

For Khon Kaen, the best model is based on quasi-likelihood method. The correlation coefficient on the test set in 2014 is 0.902 (95%CI: 0.8639, 0.9400). The highest significant variables are 1-week-lag cases, 2-week-lag cases and 3-week-lag vaporization. Other predictive variables are current week precipitation, 1-week-lag and 2-week-lag precipitation. Fitting by time series analysis yields -2.186133 for ARIMA and -126.1624 for SARIMA.

Table 27: Comparison table of all methods by the highest correlation coefficient (R^2) and the lowest prediction error (RMSE) in khonkaen.

Methods	R-squared (R^2)	Root mean square error (RMSE)
Poisson Regression	0.2499161	43.66159
Negative Binomial Regression	0.2468952	43.74942
Quasi-likelihood Regression	0.901873	15.79206
ARIMA (3,1,4)	-2.186133	13.01528
SARIMA $(2,0,1)(0,2,0)_{52}$	-126.1624	82.22447

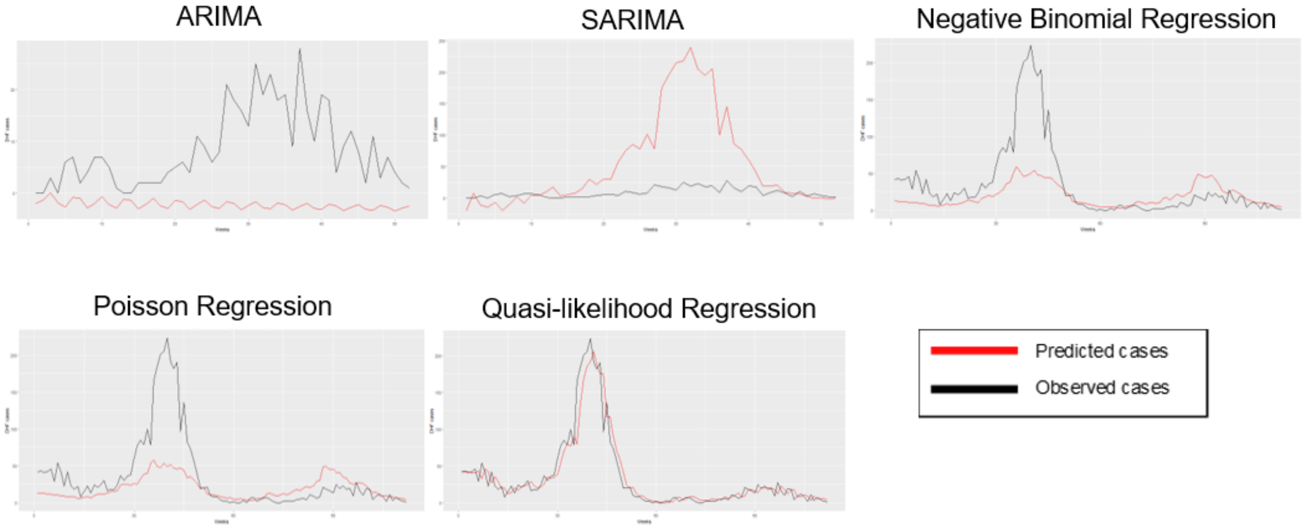

Figure 83: Plots between dengue cases and weeks, the black line represents the observed dengue cases, and the red line represents the predicted dengue cases of the best fit model of each technique over the test set data starting from January 2014 to December 2014.

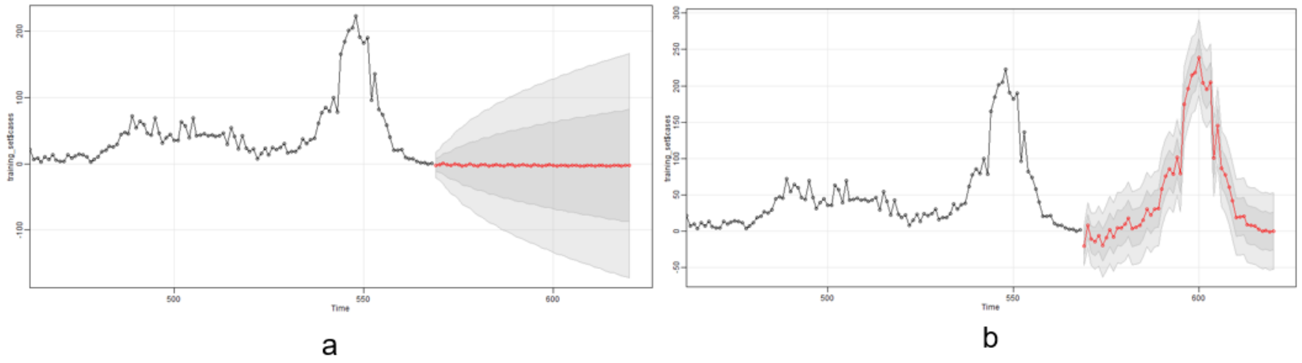

Figure 84: (a) Plot between dengue incidences over weekly time by the best model of ARIMA and (b) SARIMA time series analysis, the black line represents training set data starting from January 2012 to December 2013, and the red line represents the forecasted dengue incidences from January 2014 to December 2014.

Table 28: Coefficients and significant values of best fit GLM models, Negative Binomial, Poisson and Quasi-likelihood regression model of Khon Kaen. The table summarizes coefficients of each independent variables which are composed in best fit model of each method. The significant of each variable is labelled by asterisks under the coefficients. The most important factor is marked as three asterisks which p-value ranges from 0 to 0.001. The second important factor is marked as two asterisks which p-value ranges from 0.001 to 0.01. The third important factor is marked as an asterisk which p-value ranges from 0.01 to 0.1. The least important is also marked as a dot which p-value ranges from 0.1 to 1.

**Independent variables**	**Lag**	**Coefficients/Significant**		
		NB	Poisson	Quasi
Intercept		257.0 ***	276.2 ***	-4.15091 .
Cases	1			0.68334 ***
	2			0.25650 ***
	3			
Average Pressure	0	-0.05642 *	-0.06917 ***	
	1	-0.06055 .	-0.05626 ***	
	2			
	3	-0.1338 ***	-0.1440 ***	
Minimum Temperature	0			
	1			
	2			
	3			
Maximum Temperature	0			
	1			
	2		-0.03451 ***	
	3			
Relative Humidity	0			
	1			
	2			
	3			
Precipitation	0			-0.05136
	1			
	2		-0.005103 *	
	3			
Vaporization	0			
	1			0.71003
	2	-0.1074 **		-0.45476
	3	-0.09190 *		0.95281 *
Wind Direction	0			
	1			
	2			
	3	-6.465	-0.0007662 **	
Wind Power	0	-0.01018	-0.01262 **	
	1		-0.01039 *	
	2			
	3			

Krabi

Krabi is located in the southern region of Thailand at coordinate of 8°3′33″N 98°55′08″E. Krabi covers an area of 4,709 km^2. Total population are 456,811 people. The density of population is 97.0 people per km^2. General weather in Krabi is under tropical monsoon climate. The highest temperature is in April approximately 38.9°C. Temperature is mostly warm to hot throughout the year. Krabi has a short dry season in February and March. The highest rainfall presents in November around 545.9 mm. Humidity is in range from 73-88 percent throughout the year. The highest sunshine hours are in February.

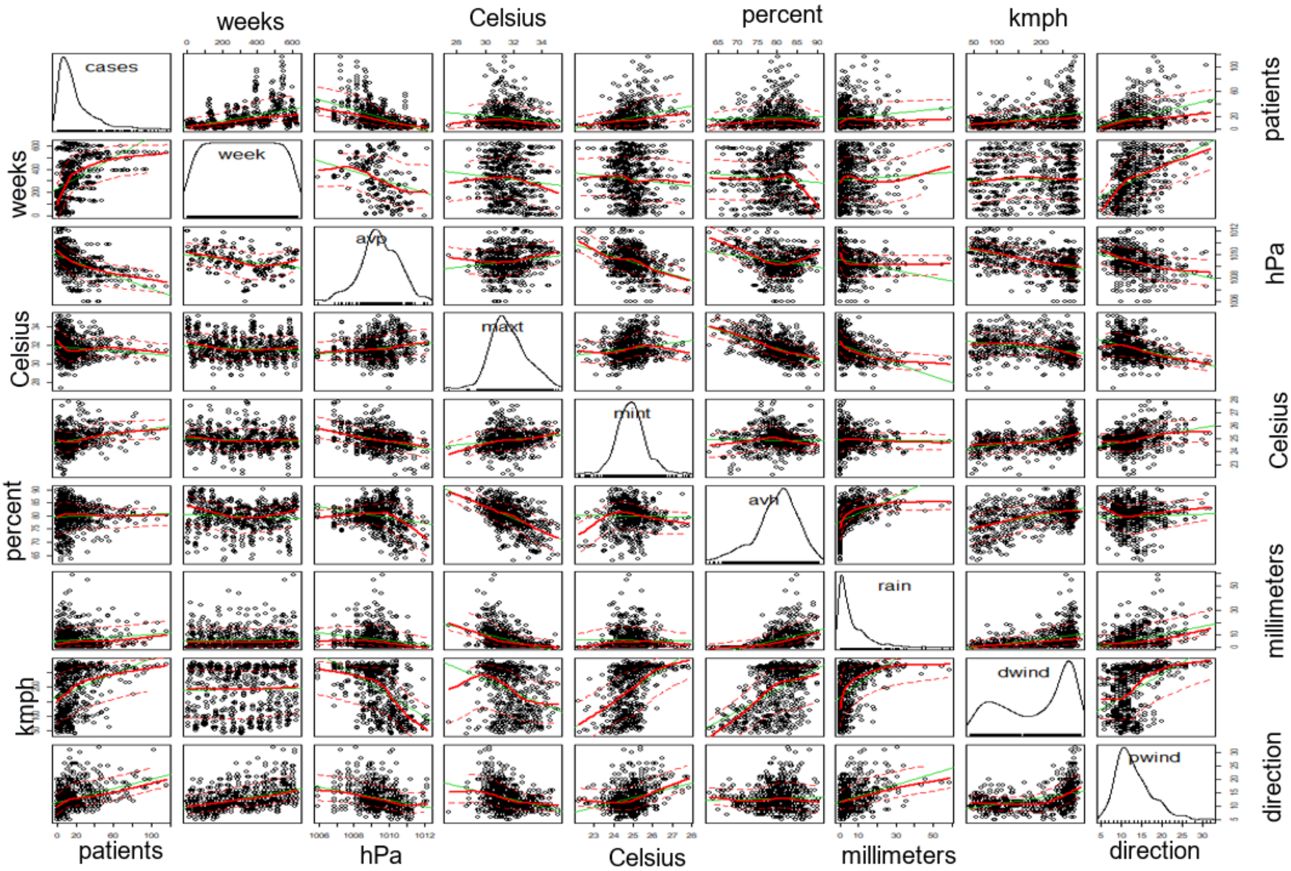

Figure 85: Scatter plot between dengue cases (cases) and selected independent variables, which are the weekly period starting from January 2001 – December 2013 (week), average pressure (avp), maximum temperature (maxt), minimum temperature (mint), average humidity (avh), precipitation (rain), vaporization of water (vapor), wind direction (dwind), and wind power (pwind). The plot visualizes pairwise hundred relationships of training set in Krabi.

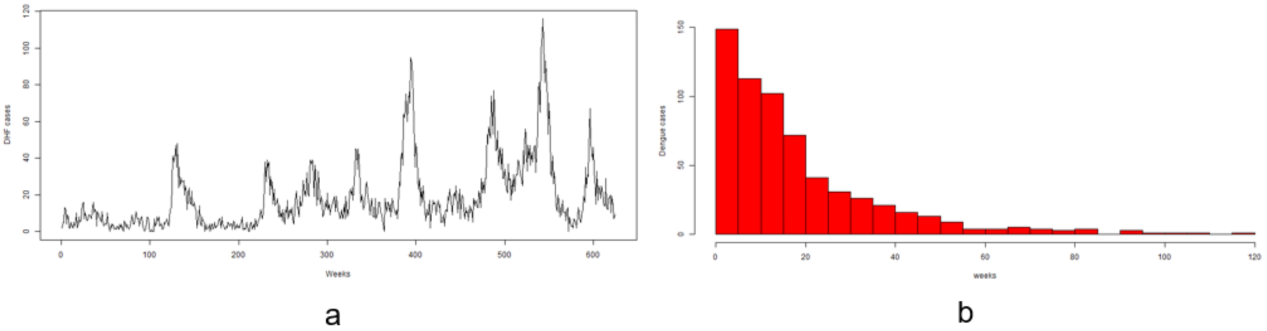

Figure 86: (a) Line plot between dengue incidences and weeks, the plot shows trends of dengue incidences in each year as stationary time series. (b) Histogram of dengue incidences in Krabi starting from January 2001 to December 2013 (624 weeks).

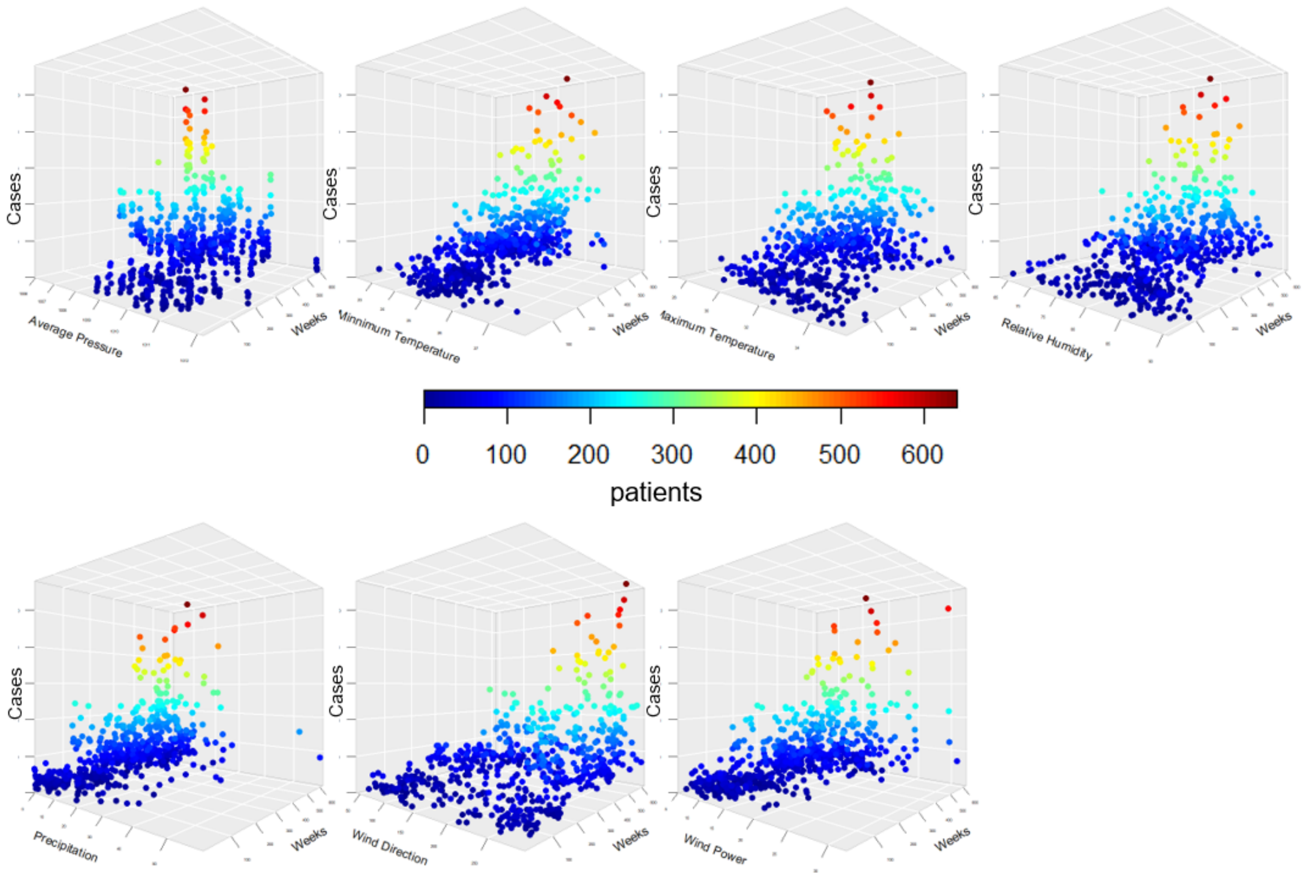

Figure 87: Three-dimensional scatter plot between dengue incidences and weather effects starting from January 2001 to December 2013 of Krabi.

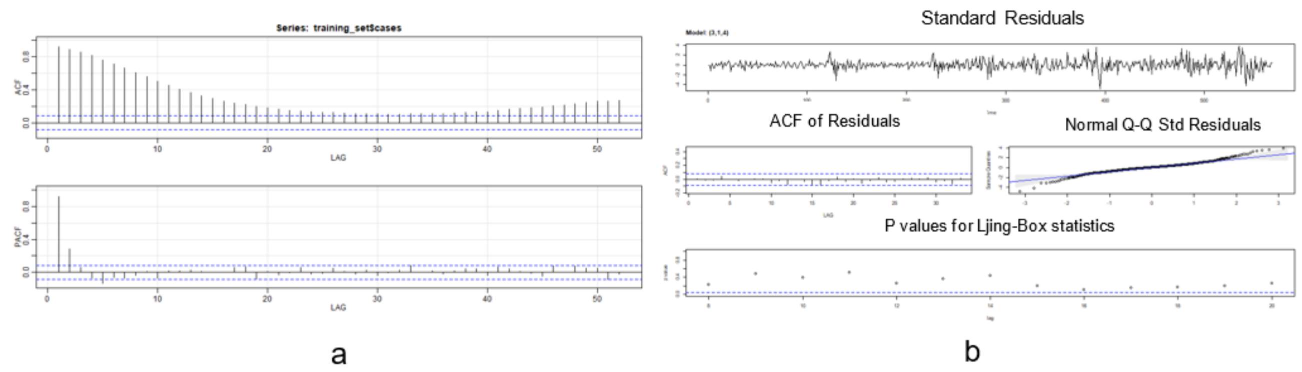

Figure 88: (a) Two plots between lag-time of dengue incidences and ACF and PACF relationship calculated from ARIMA model (b) Summary plots of time series analysis, multiple plots include the plot of predicted model over the time, the plot of ACF residual over lag-time of dengue incidences, residual Q-Q plot of standard residual, and p-value for Ljung-Box statistics of PACF relationship in Krabi over the training data starting from January 2001 to December 2013.

The best model of Krabi is based on Negative Binomial regression method. The correlation coefficient on the test set in 2014 is 0.637 (95%CI: 0.5576, 0.7164). The best model uses 6 variables. The most significant variables are current week average pressure and 2-week-lag wind direction, following by 2-week-lag average pressure. Other variables which have less significant are, current week, 1-week-lag and 2-week-lag minimum temperature. Time series methods by ARIMA and SARIMA yield the correlation coefficient of -0.2944943 and -4.385417 respectively.

Table 29: Comparison table of all methods by the highest correlation coefficient (R^2) and the lowest prediction error (RMSE) in Krabi.

**Methods**	**R-squared (R^2)**	**Root mean square error (RMSE)**
Poisson Regression	0.5466658	9.427592
Negative Binomial Regression	0.6372911	8.432778
Quasi-likelihood Regression	0.5428815	9.46686
ARIMA (3,1,4)	-0.2944943	15.93095
SARIMA $(2,0,1)(0,2,0)_{52}$	-4.385417	32.49385

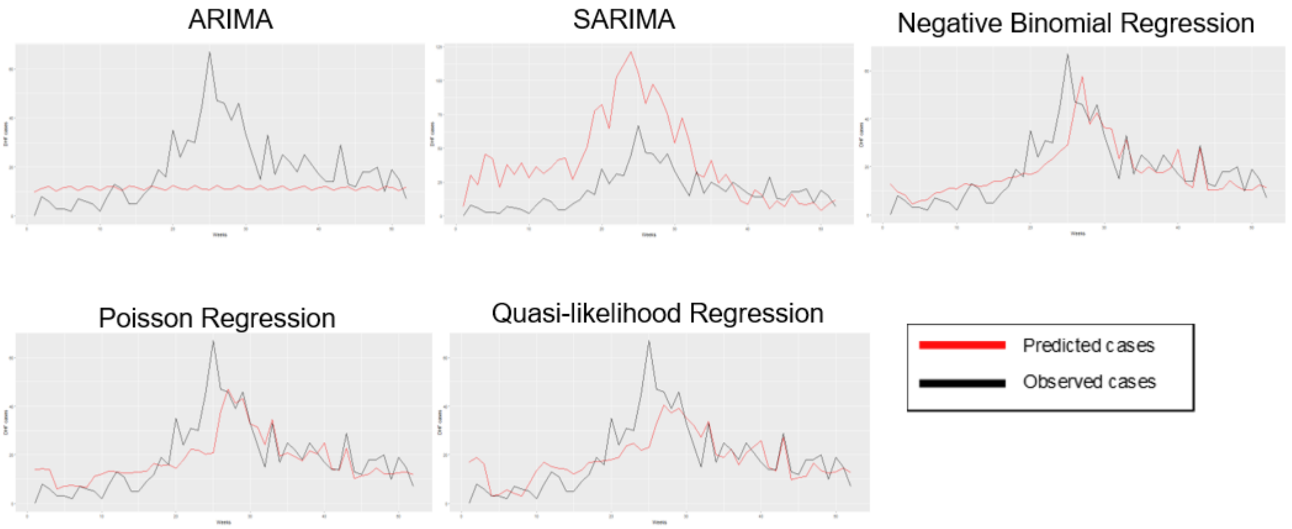

Figure 89: Plots between dengue cases and weeks, the black line represents the observed dengue cases, and the red line represents the predicted dengue cases of the best fit model of each technique over the test set data starting from January 2014 to December 2014.

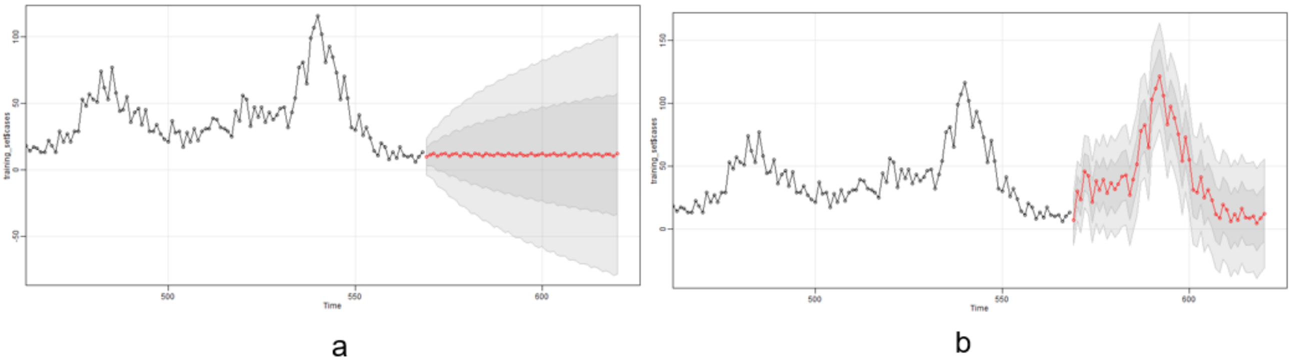

Figure 90: (a) Plot between dengue incidences over weekly time by the best model of ARIMA and (b) SARIMA time series analysis, the black line represents training set data starting from January 2012 to December 2013, and the red line represents the forecasted dengue incidences from January 2014 to December 2014.

Table 30: Coefficients and significant values of best fit GLM models, Negative Binomial, Poisson and Quasi-likelihood regression model of Krabi. The table summarizes coefficients of each independent variables which are composed in best fit model of each method. The significant of each variable is labelled by asterisks under the coefficients. The most important factor is marked as three asterisks which p-value ranges from 0 to 0.001. The second important factor is marked as two asterisks which p-value ranges from 0.001 to 0.01. The third important factor is marked as an asterisk which p-value ranges from 0.01 to 0.1. The least important is also marked as a dot which p-value ranges from 0.1 to 1.

Independent variables	Lag	Coefficients/Significant		
		NB	Poisson	Quasi
Intercept		431.789561 ***	347.2 ***	7177 ***
Cases	1			
	2			
	3			
Average Pressure	0	-0.241256 ***		
	1			
	2	-0.185091 **	-0.3429 ***	-5.029 **
	3			-2.074
Minimum Temperature	0	0.052701		
	1	0.008011	0.04977 **	
	2	-0.033189	-0.003299	
	3			
Maximum Temperature	0			
	1			
	2			
	3			
Relative Humidity	0			
	1			
	2			
	3			
Precipitation	0		-0.0004277	-0.08547
	1		0.003061 *	
	2		-0.0002709	
	3			-0.07493
Vaporization	0			
	1			
	2			
	3			
Wind Direction	0			0.005056
	1		0.0004696 *	
	2	0.050437 ***		
	3		-0.0004657 *	
Wind Power	0			
	1			
	2		0.03821 ***	0.8454 ***
	3			

Lampang

Lampang is a province located in northern region of Thailand at 18°18′N 99°30′E. Lampang covers an area of 12,534 km^2. Total population are 753,013 people. The density of population is 60.0 people per km^2. Weather in Lampang has tropical savanna climate under the South Asian monsoon system which controls more tropical wet and dry climate. Temperature is in the range from the low of 2.8°C in December to the high of 43.5°C in April. The rainy season begins with the arrival of the southwest monsoon around mid-May. The humidity presents the average of 73 percent. Precipitation occurs from mid-May to August. The highest precipitation is in September 211.6 mm annually. The longest sunshine hours are in March.

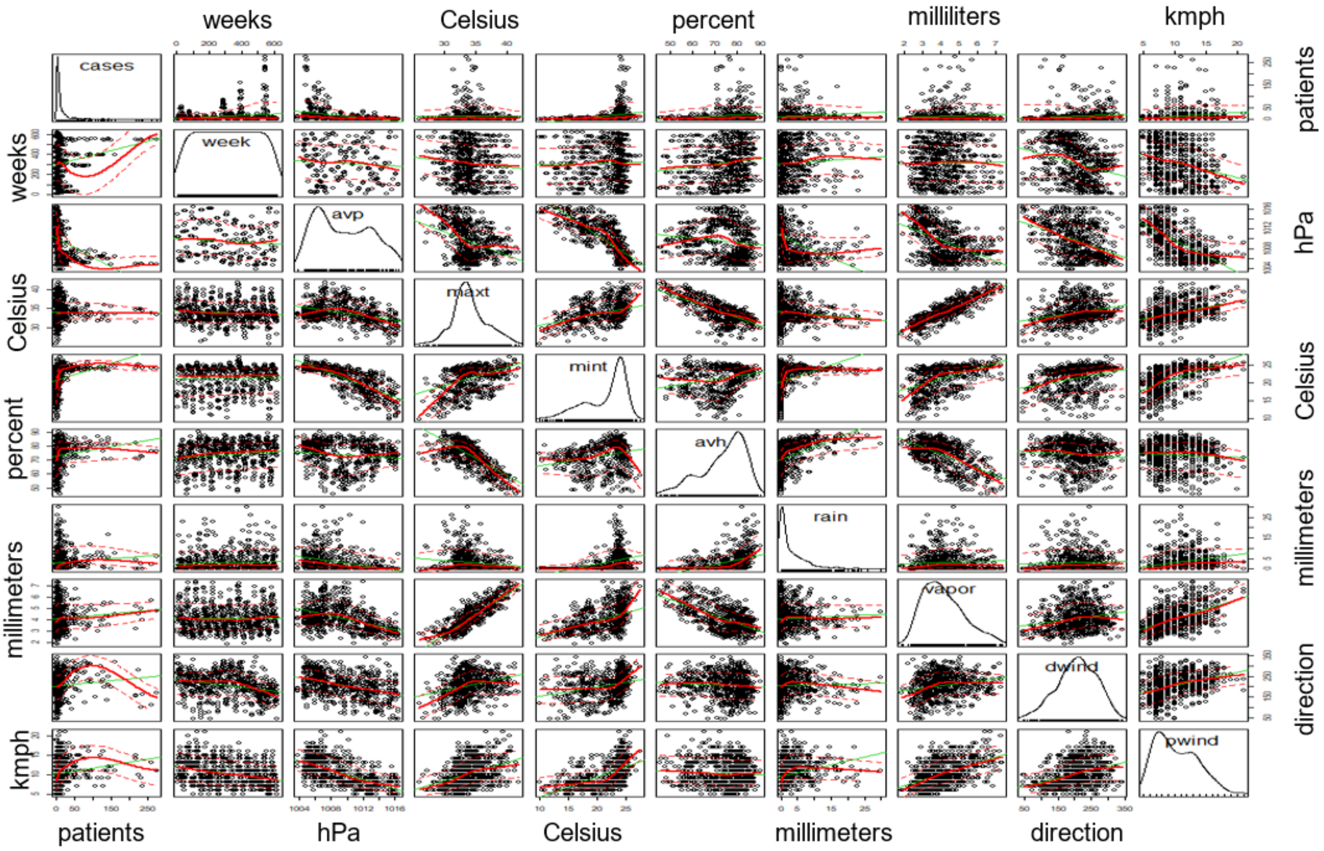

Figure 91: Scatter plot between dengue cases (cases) and selected independent variables, which are the weekly period starting from January 2001 – December 2013 (week), average pressure (avp), maximum temperature (maxt), minimum temperature (mint), average humidity (avh), precipitation (rain), vaporization of water (vapor), wind direction (dwind), and wind power (pwind). The plot visualizes pairwise hundred relationships of training set in Lampang.

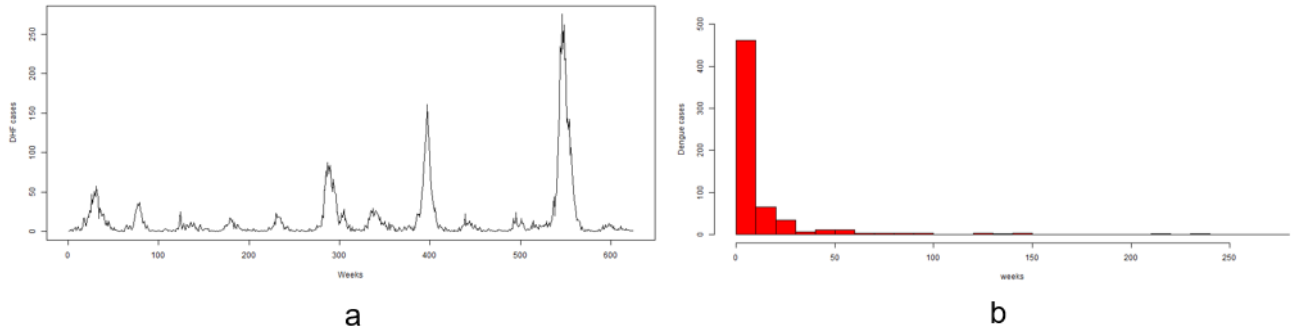

Figure 92: (a) Line plot between dengue incidences and weeks, the plot shows trends of dengue incidences in each year as stationary time series. (b) Histogram of dengue incidences in Lampang starting from January 2001 to December 2013 (624 weeks).

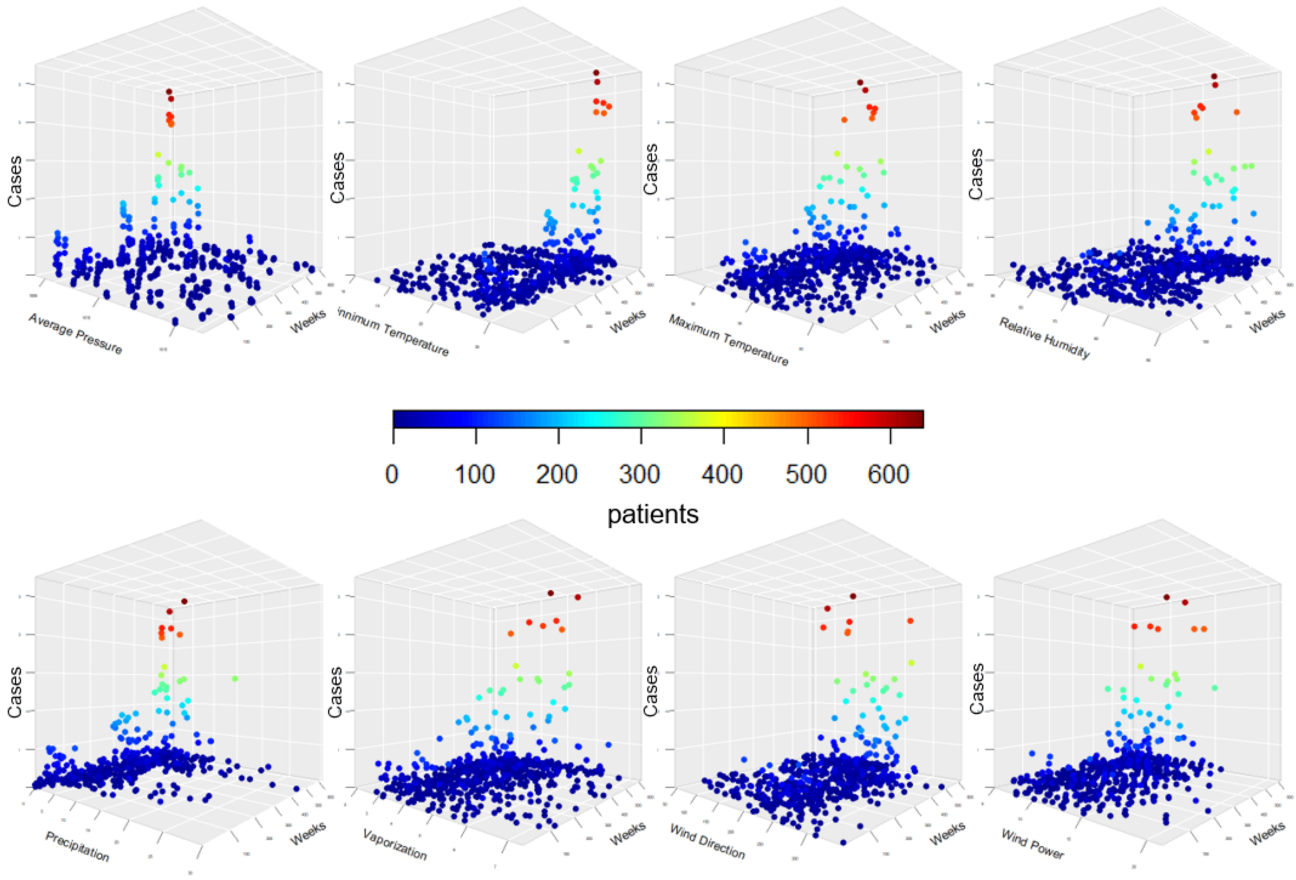

Figure 93: Three-dimensional scatter plot between dengue incidences and weather effects starting from January 2001 to December 2013 of Lampang.

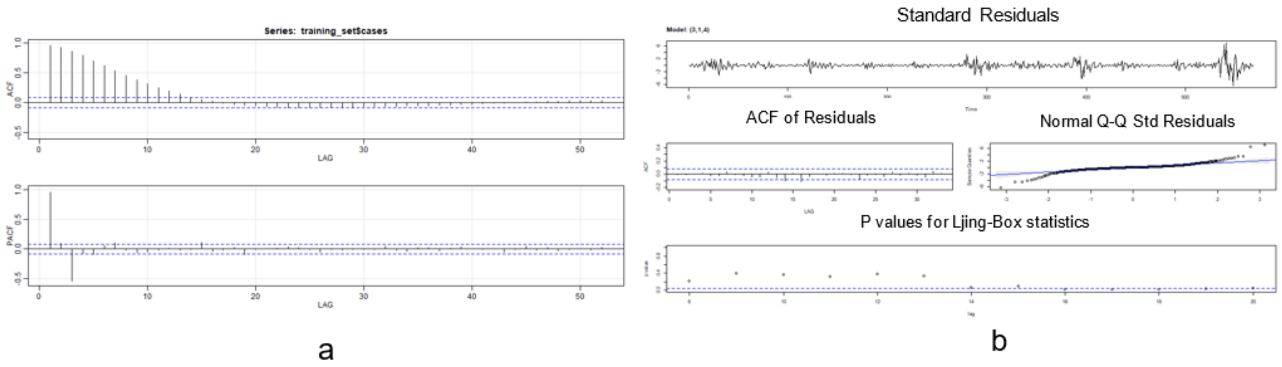

Figure 94: (a) Two plots between lag-time of dengue incidences and ACF and PACF relationship calculated from ARIMA model (b) Summary plots of time series analysis, multiple plots include the plot of predicted model over the time, the plot of ACF residual over lag-time of dengue incidences, residual Q-Q plot of standard residual, and p-value for Ljung-Box statistics of PACF relationship in Lampang over the training data starting from January 2001 to December 2013.

For Lampang, the best model is based on quasi-likelihood method. The correlation coefficient on the test set in 2014 is 0.954 (95%CI: 0.9265, 0.9815). The model consists of 8 variables. The most significant variables are 1-week-lag cases, 2-week-lag cases and 3-week-lag cases, following by 1-week-lag relative humidity. Other variables which has less significant are, 3- week-lag relative humidity, current week and 1-week-lag precipitation, and 1-week-lag vaporization. Time series methods by ARIMA and SARIMA yield the correlation coefficient of -0.4779792 and -1699.139 respectively.

Table 31: Comparison table of all methods by the highest correlation coefficient (R^2) and the lowest prediction error (RMSE) in Lampang.

Methods	R-squared (R^2)	Root mean square error (RMSE)
Poisson Regression	0.6328202	38.34761
Negative Binomial Regression	0.08247806	60.61877
Quasi-likelihood Regression	0.9542602	13.53462
ARIMA (3,1,4)	-0.4779792	3.190134
SARIMA $(2,0,1)(0,2,0)_{52}$	-1699.139	108.1975

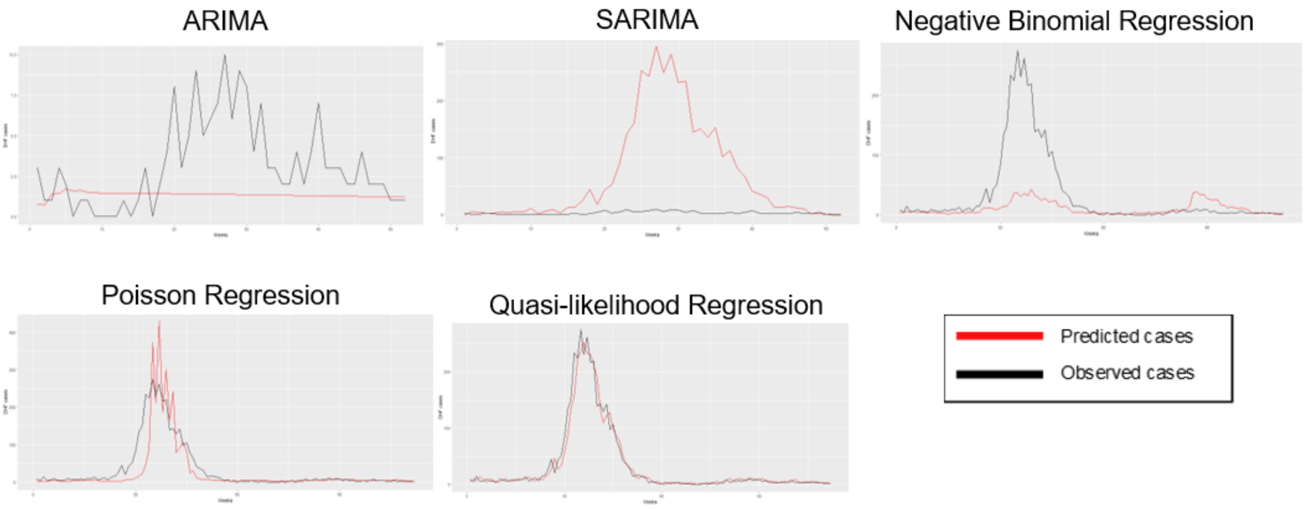

Figure 95: Plots between dengue cases and weeks, the black line represents the observed dengue cases, and the red line represents the predicted dengue cases of the best fit model of each technique over the test set data starting from January 2014 to December 2014.

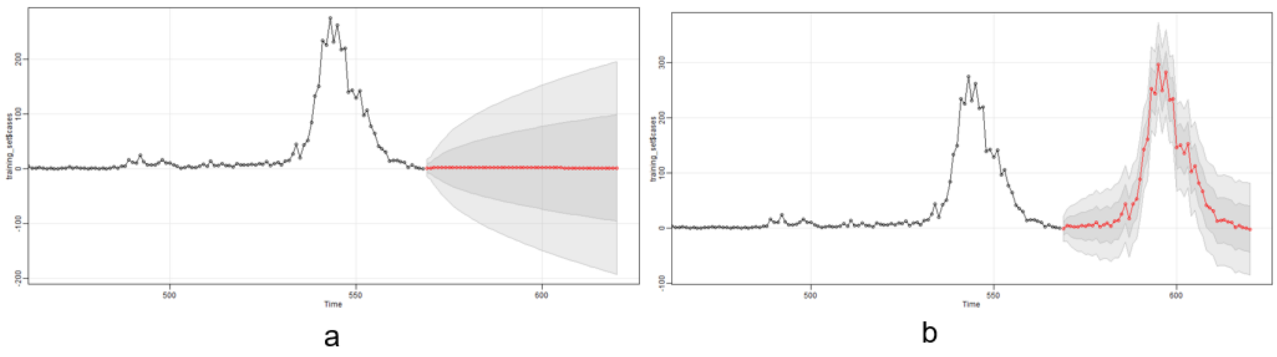

Figure 96: (a) Plot between dengue incidences over weekly time by the best model of ARIMA and (b) SARIMA time series analysis, the black line represents training set data starting from January 2012 to December 2013, and the red line represents the forecasted dengue incidences from January 2014 to December 2014.

Table 32: Coefficients and significant values of best fit GLM models, Negative Binomial, Poisson and Quasi-likelihood regression model of Lampang. The table summarizes coefficients of each independent variables which are composed in best fit model of each method. The significant of each variable is labelled by asterisks under the coefficients. The most important factor is marked as three asterisks which p-value ranges from 0 to 0.001. The second important factor is marked as two asterisks which p-value ranges from 0.001 to 0.01. The third important factor is marked as an asterisk which p-value ranges from 0.01 to 0.1. The least important is also marked as a dot which p-value ranges from 0.1 to 1.

**Independent variables**	**Lag**	**Coefficients/Significant**		
		NB	Poisson	Quasi
Intercept		360.553049 ***	-2.4496618 ***	-4.05444
Cases	1			0.90057 ***
	2		0.0188248 ***	0.34388 ***
	3			-0.31606 ***
Average Pressure	0	-0.017802		
	1	-0.092619		
	2	-0.240348 ***		
	3			
Minimum Temperature	0			
	1			
	2			
	3			
Maximum Temperature	0	-0.127344 ***		
	1			
	2			
	3			
Relative Humidity	0			
	1		0.0220818 ***	0.11616 .
	2			
	3			-0.07001
Precipitation	0	-0.023047 *	0.0045603	-0.07072
	1	-0.006916		-0.04351
	2	-0.002352		
	3		0.0101788 **	
Vaporization	0			
	1			0.43881
	2			
	3			
Wind Direction	0		0.0041689 ***	
	1		0.0033746 ***	
	2		0.0019154 ***	
	3		0.0020883 ***	
Wind Power	0			
	1	-0.012147		
	2			
	3			

Lamphun

Lamphun is located in northern Thailand at 18°35′11″N 99°0′43″E. Lamphun covers an area of 4,506 km^2. Total population are 405,468 people. The density of population is 90.0 people per km^2. Weather in Lamphun has tropical savanna climate under the South Asian monsoon system which controls more tropical wet and dry climate. Temperature is in the range from the low of 2.8°C in January to the high of 42.6°C in April. The rainy season begins with the arrival of the southwest monsoon around mid-May. The humidity presents from 54-83 percent throughout the year. Precipitation occurs from mid-May to August. The highest precipitation is in September of 208.2 *mm* annually.

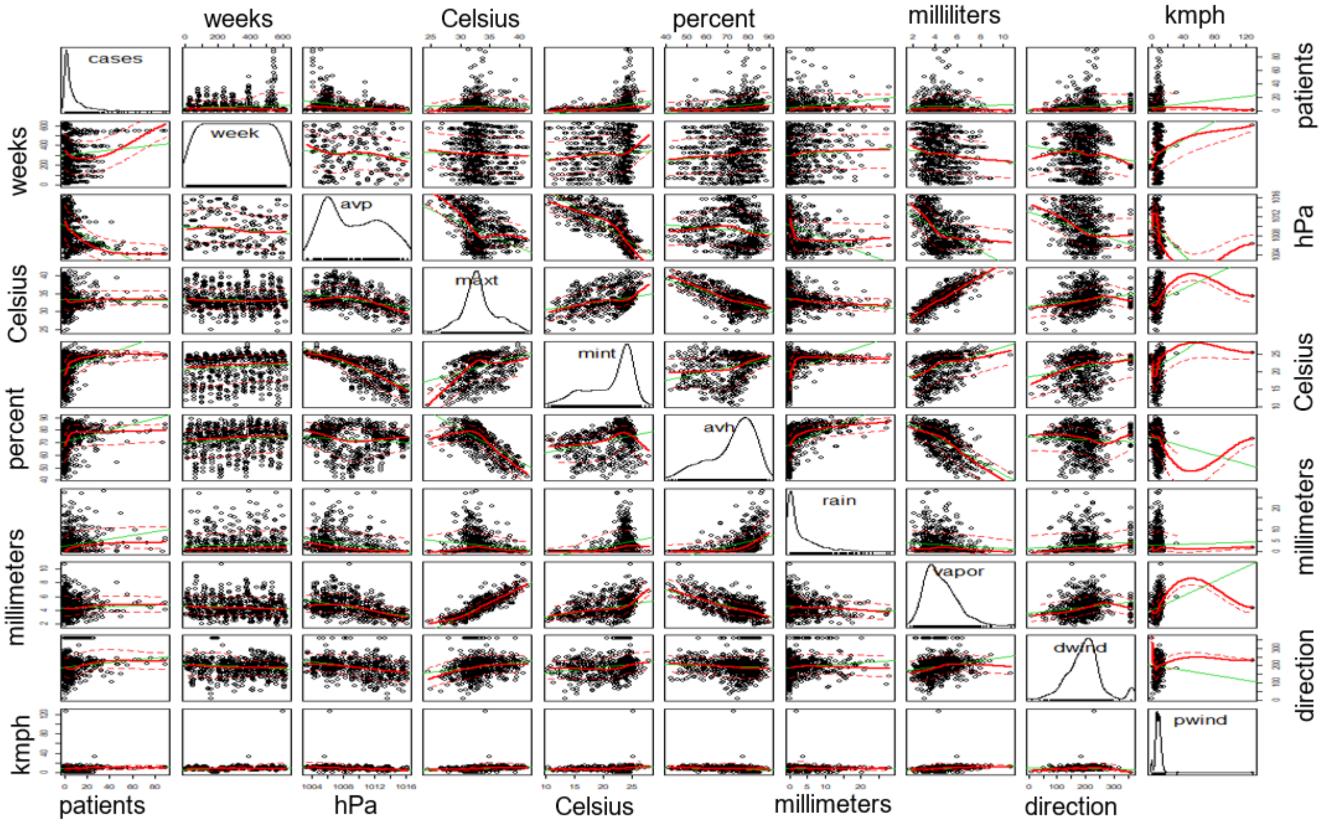

Figure 97: Scatter plot between dengue cases (cases) and selected independent variables, which are the weekly period starting from January 2001 – December 2013 (week), average pressure (avp), maximum temperature (maxt), minimum temperature (mint), average humidity (avh), precipitation (rain), vaporization of water (vapor), wind direction (dwind), and wind power (pwind). The plot visualizes pairwise hundred relationships of training set in Lamphun.

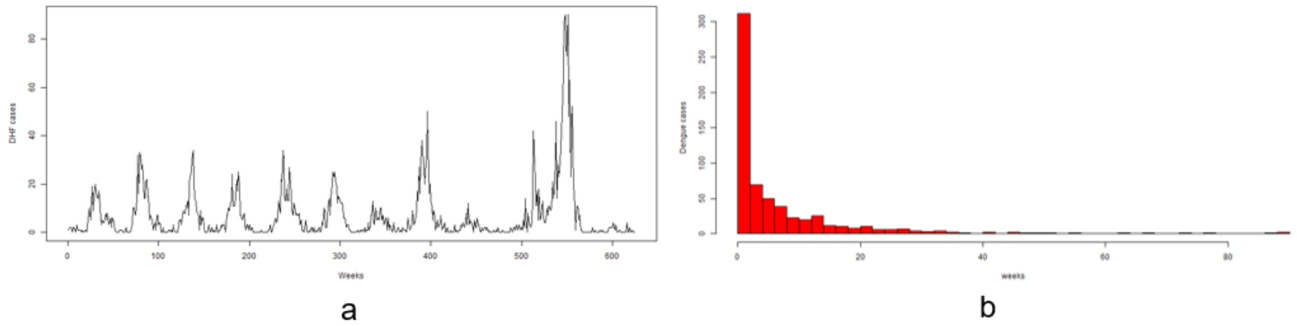

Figure 98: (a) Line plot between dengue incidences and weeks, the plot shows trends of dengue incidences in each year as stationary time series. (b) Histogram of dengue incidences in Lamphun starting from January 2001 to December 2013 (624 weeks).

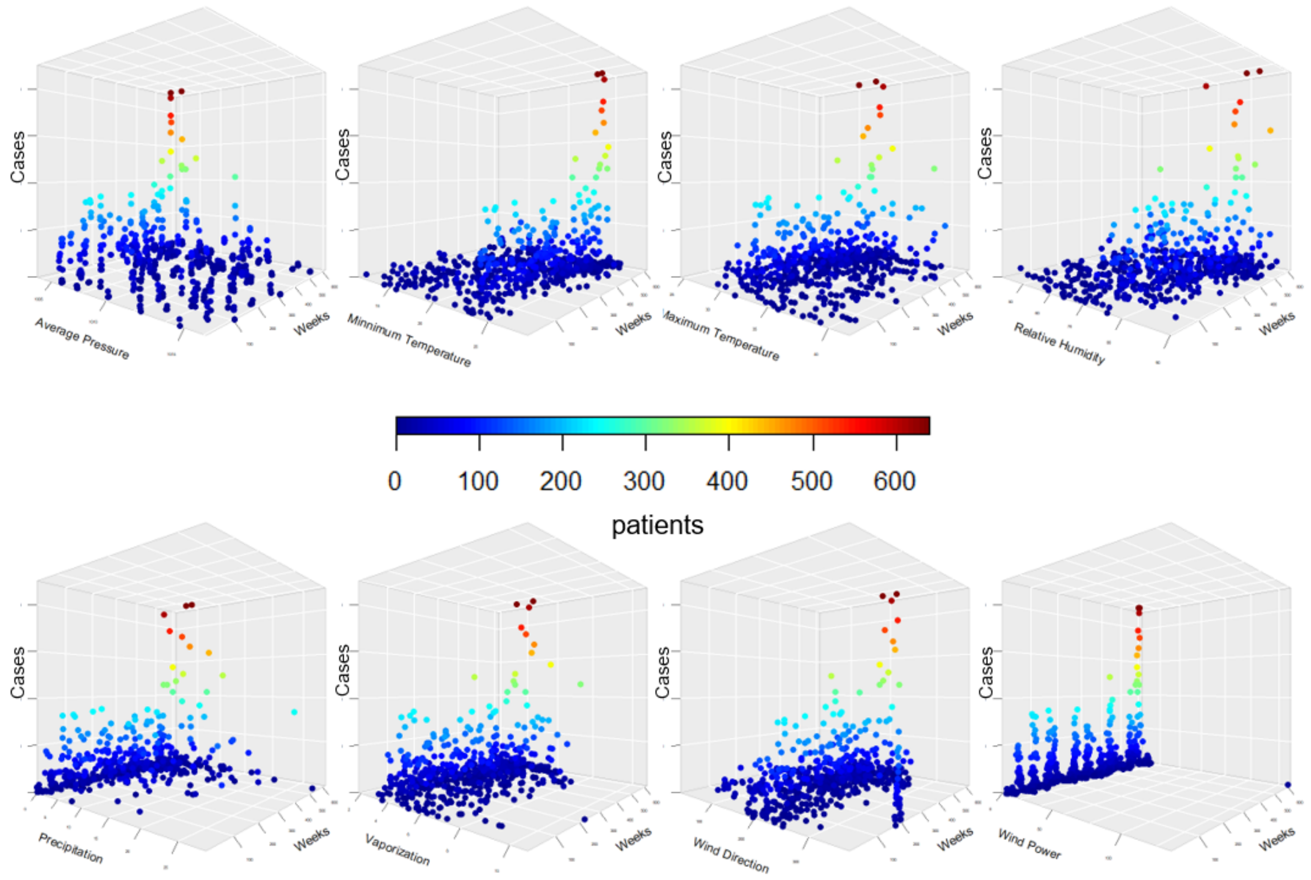

Figure 99: Three-dimensional scatter plot between dengue incidences and weather effects starting from January 2001 to December 2013 of Lamphun.

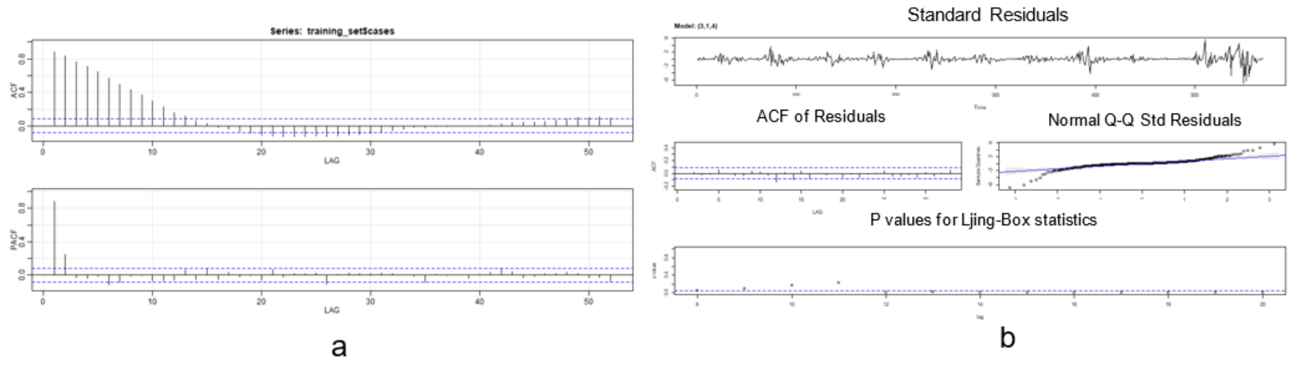

Figure 100: (a) Two plots between lag-time of dengue incidences and ACF and PACF relationship calculated from ARIMA model (b) Summary plots of time series analysis, multiple plots include the plot of predicted model over the time, the plot of ACF residual over lag-time of dengue incidences, residual Q-Q plot of standard residual, and p-value for Ljung-Box statistics of PACF relationship in Lamphun over the training data starting from January 2001 to December 2013.

For Lamphun, the best model is based on quasi-likelihood method. The correlation coefficient on the test set in 2014 is 0.819 (95%CI: 0.7911, 0.8469). The model of consists of 8 variables. The most significant variables are 1-week-lag cases, 2-week-lag cases, following by 1-week-lag precipitation, 2-week-lag vaporization. Other variables which has less significant are, 2-week-lag maximum temperature, 3-week-lag relative humidity, 3-week-lag precipitation and current week vaporization. Time series methods by ARIMA and SARIMA yield the correlation coefficient of -0.3857469 and -1204.527 respectively.

Table 33: Comparison table of all methods by the highest correlation coefficient (R^2) and the lowest prediction error (RMSE) in Lamphun.

**Methods**	**R-squared (R^2)**	**Root mean square error (RMSE)**
Poisson Regression	0.1192882	19.96606
Negative Binomial Regression	0.1391349	19.73981
Quasi-likelihood Regression	0.8189355	9.052991
ARIMA (3,1,4)	-0.3857469	1.237246
SARIMA $(2,0,1)(0,2,0)_{52}$	-1204.527	36.49242

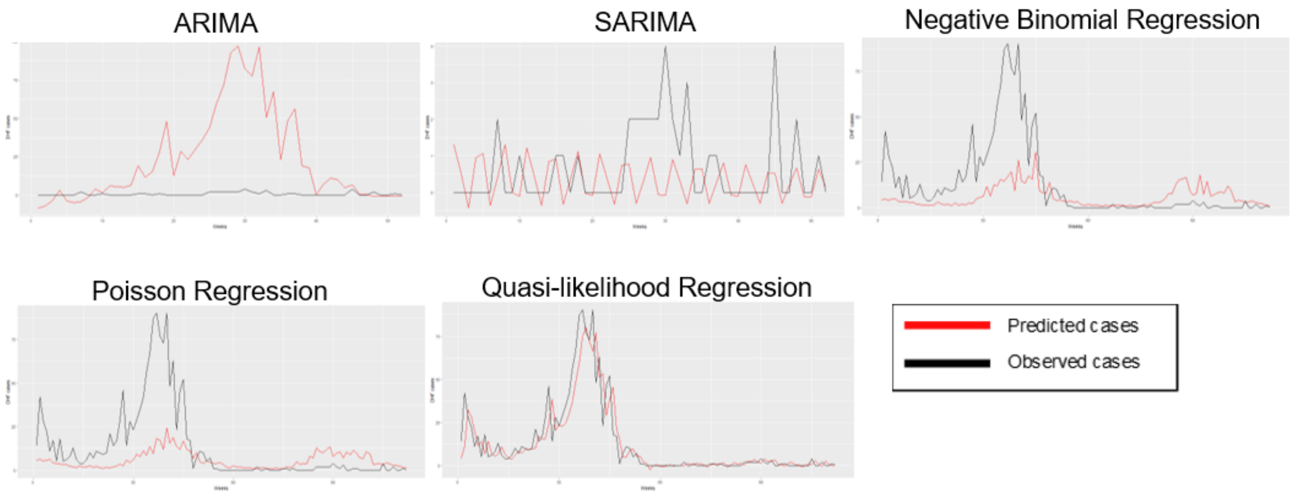

Figure 101: Plots between dengue cases and weeks, the black line represents the observed dengue cases, and the red line represents the predicted dengue cases of the best fit model of each technique over the test set data starting from January 2014 to December 2014.

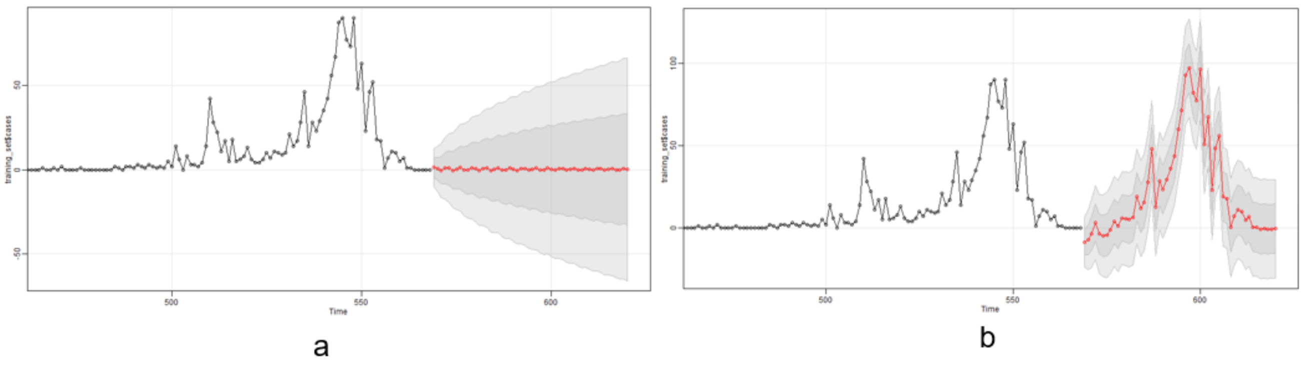

Figure 102: (a) Plot between dengue incidences over weekly time by the best model of ARIMA and (b) SARIMA time series analysis, the black line represents training set data starting from January 2012 to December 2013, and the red line represents the forecasted dengue incidences from January 2014 to December 2014.

Table 34: Coefficients and significant values of best fit GLM models, Negative Binomial, Poisson and Quasi-likelihood regression model of Lamphun. The table summarizes coefficients of each independent variables which are composed in best fit model of each method. The significant of each variable is labelled by asterisks under the coefficients. The most important factor is marked as three asterisks which p-value ranges from 0 to 0.001. The second important factor is marked as two asterisks which p-value ranges from 0.001 to 0.01. The third important factor is marked as an asterisk which p-value ranges from 0.01 to 0.1. The least important is also marked as a dot which p-value ranges from 0.1 to 1.

**Independent variables**	**Lag**	**Coefficients/Significant**		
		NB	Poisson	Quasi
Intercept		169.3714 ***	160.152138 ***	-3.87073
Cases	1			0.69988 ***
	2			0.18484 ***
	3			
Average Pressure	0		-0.164917 ***	
	1	-0.174884 ***		
	2			
	3			
Minimum Temperature	0			
	1			
	2			
	3			
Maximum Temperature	0			
	1			
	2			-0.06472
	3			
Relative Humidity	0		0.041624 ***	
	1			
	2			
	3	0.091503 ***	0.048836 ***	0.04537
Precipitation	0			
	1		-0.010472 **	-0.10907 **
	2		-0.015495 ***	
	3	-0.025973 *	0.030033 ***	0.01762
Vaporization	0		0.097174 ***	0.17955
	1			
	2			0.64058 *
	3	0.420044 ***	0.183547 ***	
Wind Direction	0			
	1			
	2			
	3			
Wind Power	0			
	1			
	2			
	3			

Loei

Loei is in the north of northeastern continent of Thailand at coordinate of 17°29′07″N 101°43′49″E. Loei covers an area of 11,425 km^2. Total population are 634,513 people. The density of population is approximately 56.0 people per km^2. Weather in Loei has tropical savanna climate under the South Asian monsoon system. Temperature is high in April approximately 42.6 °C and starts to low temperature from December to February (4.9-6.6°C). Winters are dry and warm. The monsoon season begins from late-April through October. The highest rainfall presents in August around 235.0 mm. Humidity is in range from 61-81 percent throughout the year. The highest sunshine hours are in March.

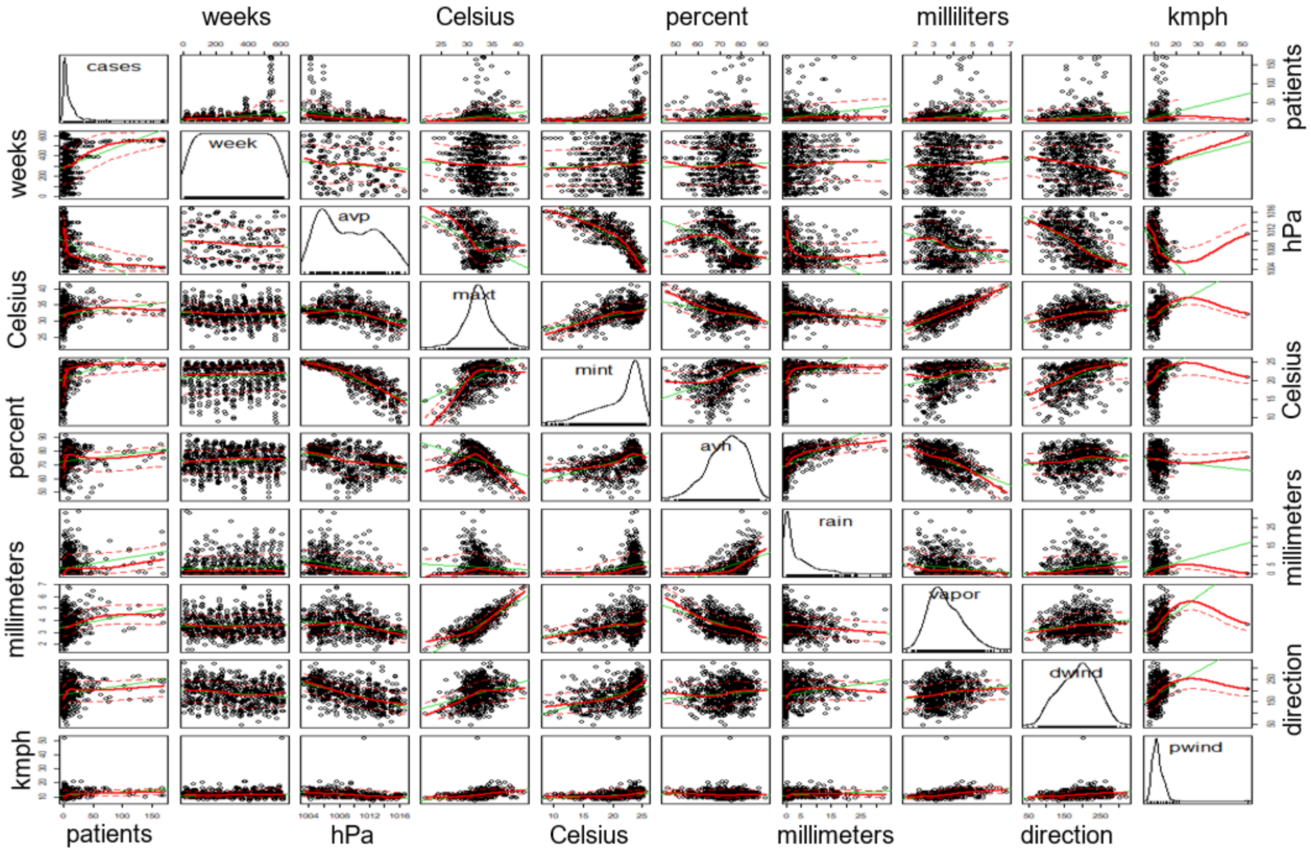

Figure 103: Scatter plot between dengue cases (cases) and selected independent variables, which are the weekly period starting from January 2001 – December 2013 (week), average pressure (avp), maximum temperature (maxt), minimum temperature (mint), average humidity (avh), precipitation (rain), vaporization of water (vapor), wind direction (dwind), and wind power (pwind). The plot visualizes pairwise hundred relationships of training set in Loei.

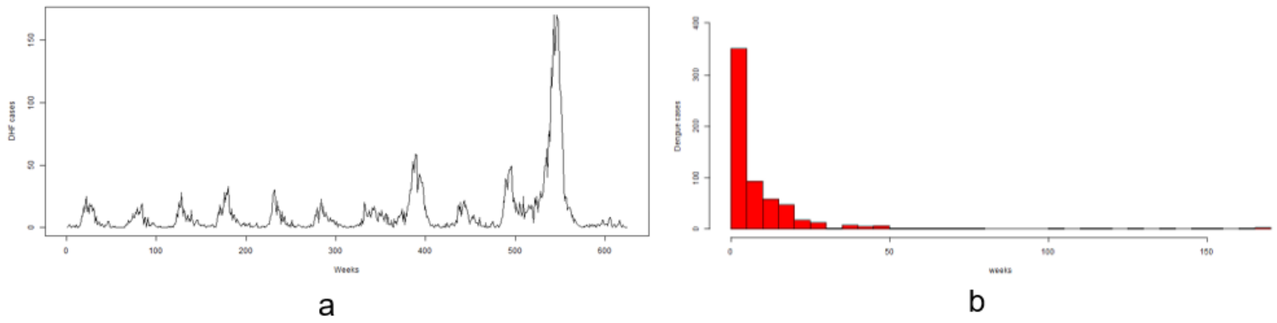

Figure 104: (a) Line plot between dengue incidences and weeks, the plot shows trends of dengue incidences in each year as stationary time series. (b) Histogram of dengue incidences in Loei starting from January 2001 to December 2013 (624 weeks).

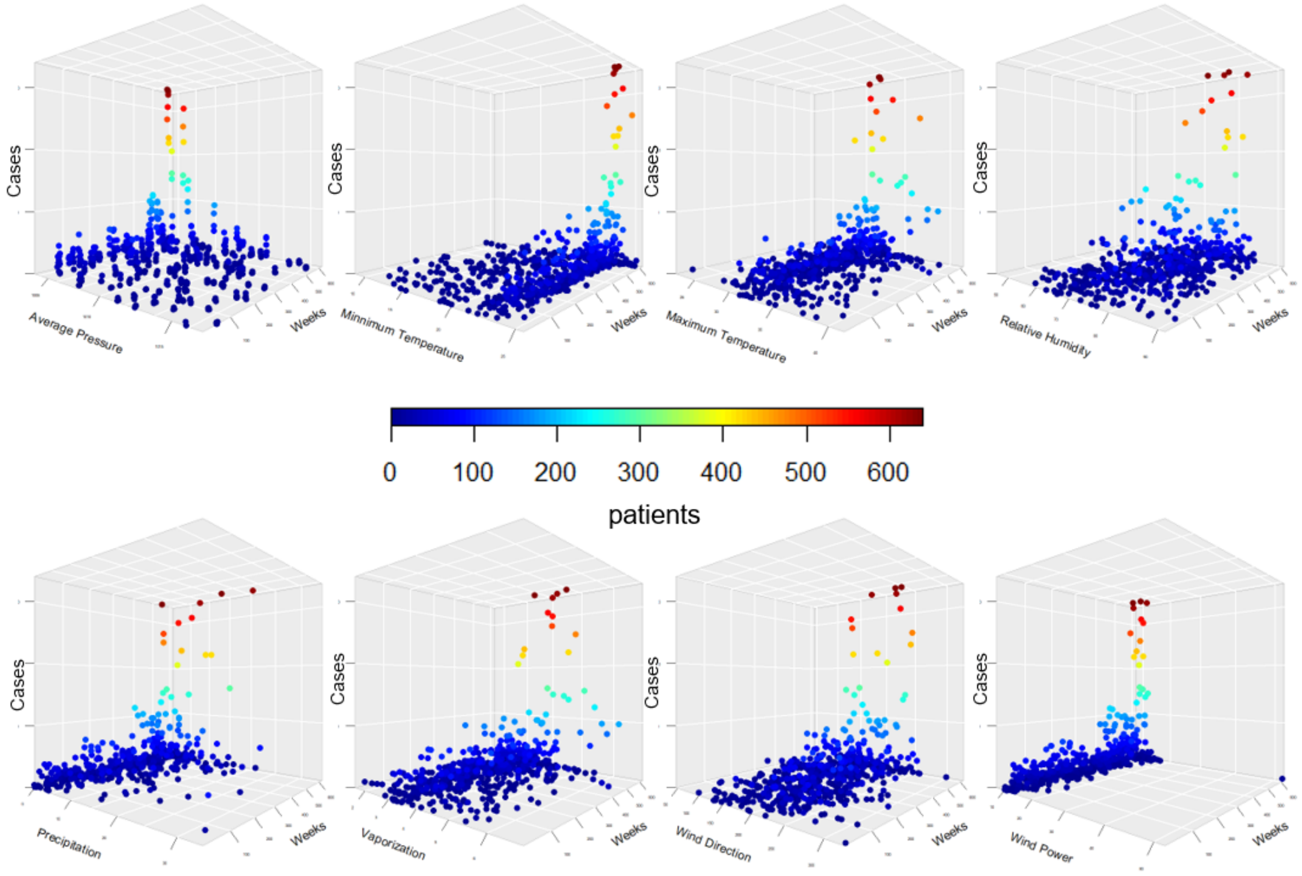

Figure 105: Three-dimensional scatter plot between dengue incidences and weather effects starting from January 2001 to December 2013 of Loei.

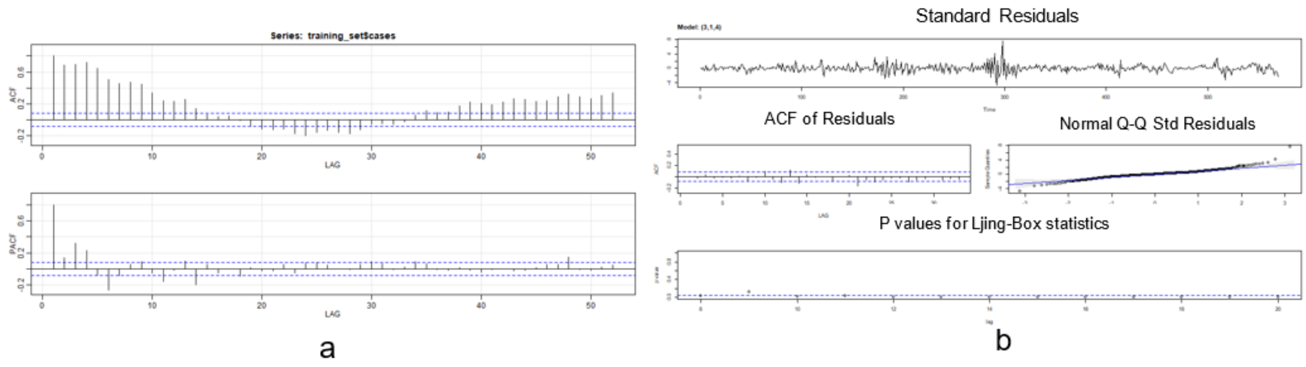

Figure 106: (a) Two plots between lag-time of dengue incidences and ACF and PACF relationship calculated from ARIMA model (b) Summary plots of time series analysis, multiple plots include the plot of predicted model over the time, the plot of ACF residual over lag-time of dengue incidences, residual Q-Q plot of standard residual, and p-value for Ljung-Box statistics of PACF relationship in Loei over the training data starting from January 2001 to December 2013.

The best model of Loei is based on quasi-likelihood method. The correlation coefficient on the test set in 2014 is 0.928 (95%CI: 0.6813, 0.8287). The best model consists of 8 variables. The most significant variables are 1-week-lag cases, 2-week-lag case, following by current week vaporization and 3-week-lag vaporization. Other variables are, 2-week-lag and 3-week-lag wind direction. Time series methods by ARIMA and SARIMA yield the correlation coefficient of -4.376512 and -1559.44 respectively.

Table 35: Comparison table of all methods by the highest correlation coefficient (R^2) and the lowest prediction error (RMSE) in Loei.

Methods	R-squared (R^2)	Root mean square error (RMSE)
Poisson Regression	0.1336408	40.7564
Negative Binomial Regression	0.2177061	38.7286
Quasi-likelihood Regression	0.9286471	11.69641
ARIMA (3,1,4)	-4.376512	4.535803
SARIMA $(2,0,1)(0,2,0)_{52}$	-1559.44	77.27295

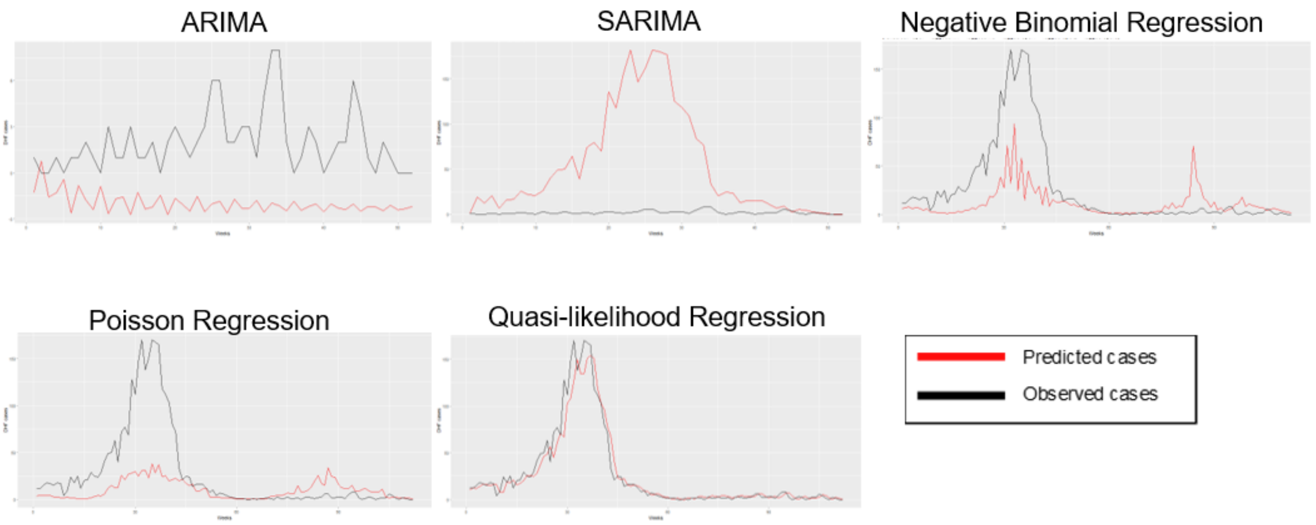

Figure 107: Plots between dengue cases and weeks, the black line represents the observed dengue cases, and the red line represents the predicted dengue cases of the best fit model of each technique over the test set data starting from January 2014 to December 2014.

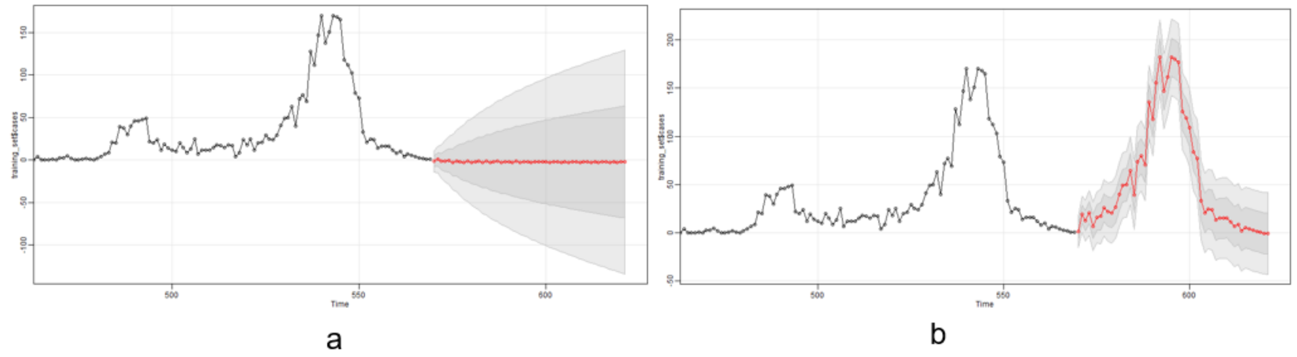

Figure 108: (a) Plot between dengue incidences over weekly time by the best model of ARIMA and (b) SARIMA time series analysis, the black line represents training set data starting from January 2012 to December 2013, and the red line represents the forecasted dengue incidences from January 2014 to December 2014.

Table 36: Coefficients and significant values of best fit GLM models, Negative Binomial, Poisson and Quasi-likelihood regression model of Loei. The table summarizes coefficients of each independent variables which are composed in best fit model of each method. The significant of each variable is labelled by asterisks under the coefficients. The most important factor is marked as three asterisks which p-value ranges from 0 to 0.001. The second important factor is marked as two asterisks which p-value ranges from 0.001 to 0.01. The third important factor is marked as an asterisk which p-value ranges from 0.01 to 0.1. The least important is also marked as a dot which p-value ranges from 0.1 to 1.

**Independent variables**	**Lag**	**Coefficients/Significant**		
		NB	Poisson	Quasi
Intercept		-14.579097 ***	248.6 ***	-2.750888 *
Cases	1			0.656318 ***
	2			0.248310 ***
	3			
Average Pressure	0			
	1			
	2			
	3		-0.2502 ***	
Minimum Temperature	0			
	1			
	2			
	3			
Maximum Temperature	0			
	1			
	2			
	3			
Relative Humidity	0		0.01544 ***	
	1	0.079330 ***		
	2			
	3	0.067210 ***	0.02490 ***	
Precipitation	0			
	1			
	2			
	3			
Vaporization	0	0.252570 ***	0.2615 ***	0.662734 **
	1	0.543171 ***	0.1706 ***	
	2	0.243458 ***	0.1546 ***	
	3	0.508525 ***	0.2073 ***	0.477490 .
Wind Direction	0			
	1			
	2		-0.002088 ***	-0.001780
	3			-0.001051
Wind Power	0			
	1			
	2			
	3			

Lopburi

Lopburi is located in the central region of Thailand at coordinate of 14°48′0″N 100°37′37″E. Lopburi covers an area of 6,200 km^2. Total population are 754,406 people. The density of population is 122.0 people per km^2. Weather in Lopburi follows tropical savanna climate system. The highest temperature is in April approximately 41.4°C. The low temperature presents in winter from December to February (4.5-6.9°C). The monsoon season starts from May through August. The highest sunshine hours are in January. Humidity presents around 63-81 percent throughout the year.

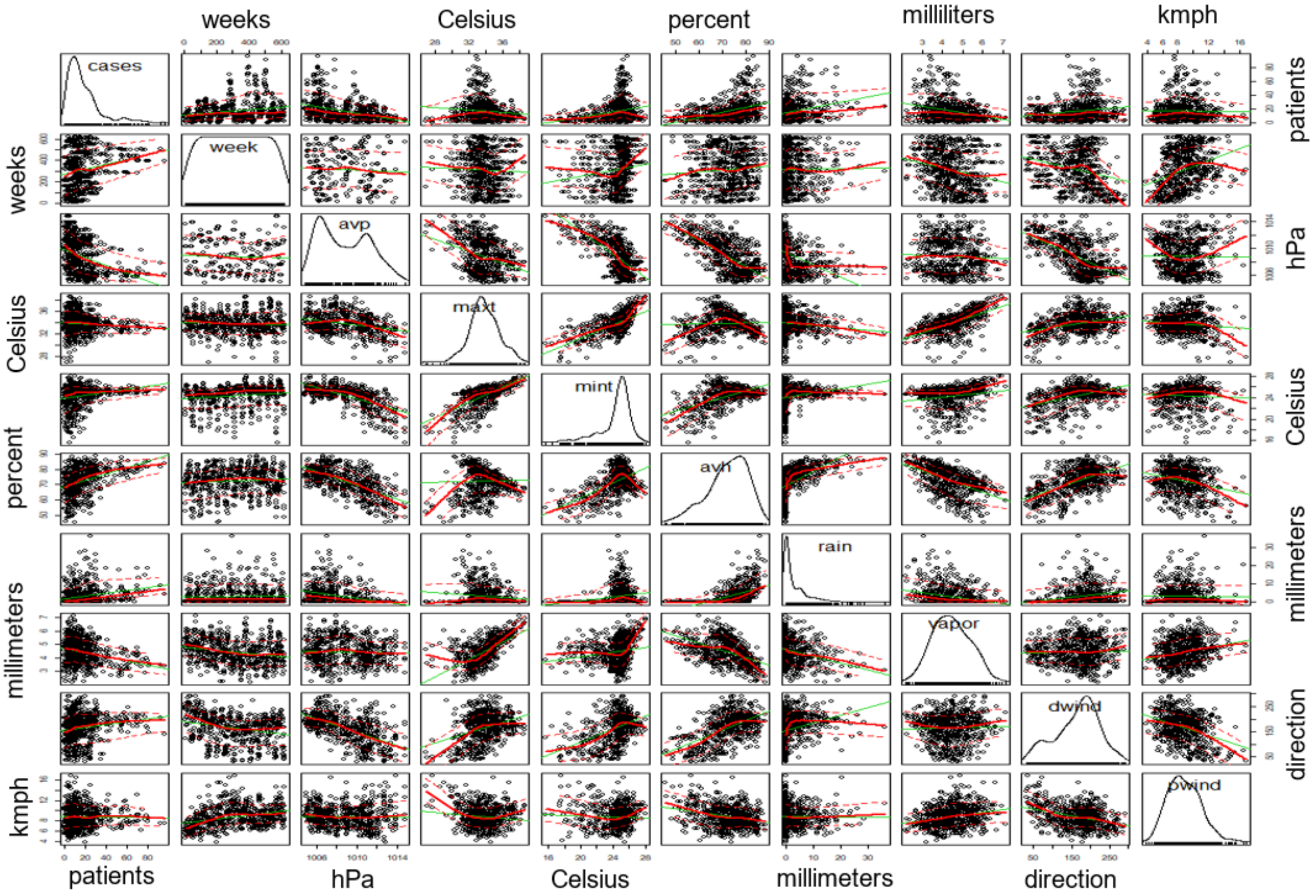

Figure 109: Scatter plot between dengue cases (cases) and selected independent variables, which are the weekly period starting from January 2001 – December 2013 (week), average pressure (avp), maximum temperature (maxt), minimum temperature (mint), average humidity (avh), precipitation (rain), vaporization of water (vapor), wind direction (dwind), and wind power (pwind). The plot visualizes pairwise hundred relationships of training set in Lopburi.

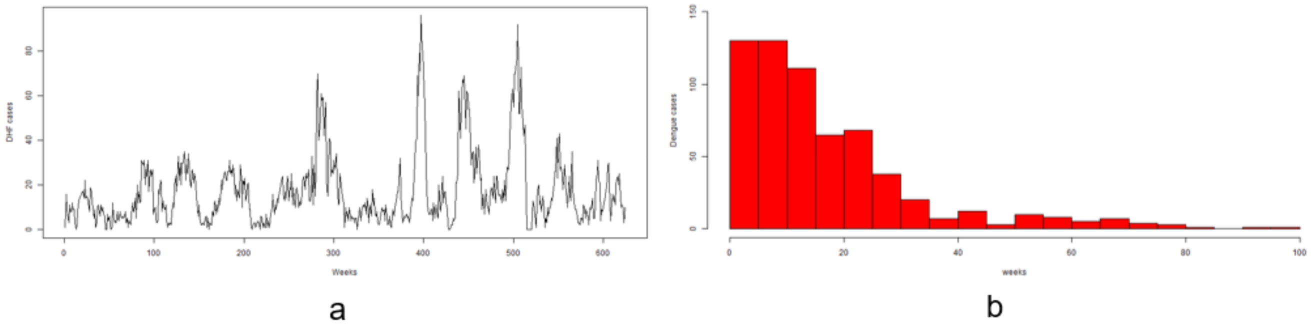

Figure 110: (a) Line plot between dengue incidences and weeks, the plot shows trends of dengue incidences in each year as stationary time series. (b) Histogram of dengue incidences in Lopburi starting from January 2001 to December 2013 (624 weeks).

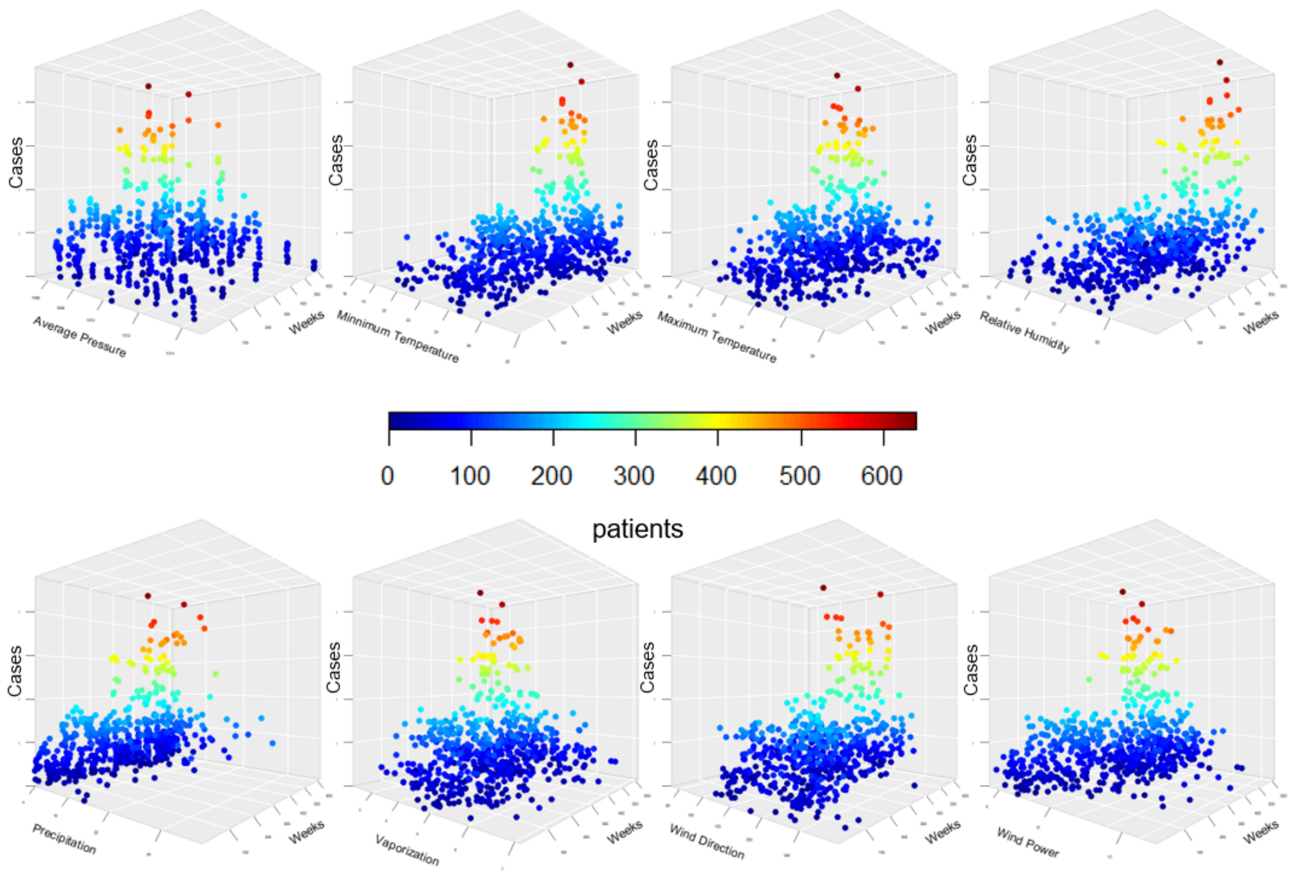

Figure 111: Three-dimensional scatter plot between dengue incidences and weather effects starting from January 2001 to December 2013 of Lopburi.

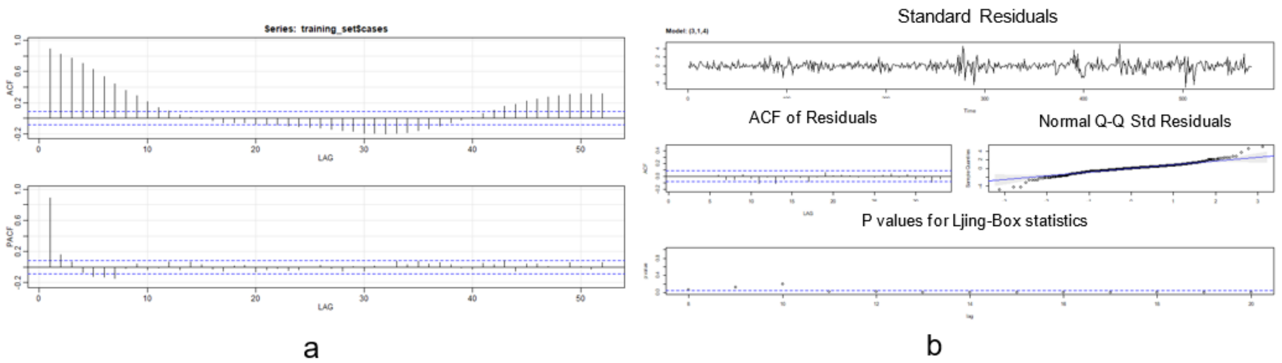

Figure 112: (a) Two plots between lag-time of dengue incidences and ACF and PACF relationship calculated from ARIMA model (b) Summary plots of time series analysis, multiple plots include the plot of predicted model over the time, the plot of ACF residual over lag-time of dengue incidences, residual Q-Q plot of standard residual, and p-value for Ljung-Box statistics of PACF relationship in Lopburi over the training data starting from January 2001 to December 2013.

For Lopburi, the best model is based on quasi-likelihood method. The correlation coefficient on the test set in 2014 is 0.50 (95%CI: 0.4624, 0.6496). The model uses 8 variables. The most significant variable is 1-week-lag cases, following by 3-week-lag average pressure, 3-week-lag maximum temperature and 3-week-lag precipitation. Other variables which have less significant are, current week maximum temperature, current week relative humidity, current week and 2-week-lag wind direction. Time series methods by ARIMA and SARIMA yield the correlation coeffect of -2.160863 and -1.539306 respectively.

Table 37: Comparison table of all methods by the highest correlation coefficient (R^2) and the lowest prediction error (RMSE) in Lopburi.

Methods	R-squared (R^2)	Root mean square error (RMSE)
Poisson Regression	0.4681088	5.567762
Negative Binomial Regression	0.453226	5.64512
Quasi-likelihood Regression	0.50557	5.368113
ARIMA (3,1,4)	-2.160863	13.57288
SARIMA $(2,0,1)(0,2,0)_{52}$	-1.539306	12.16541

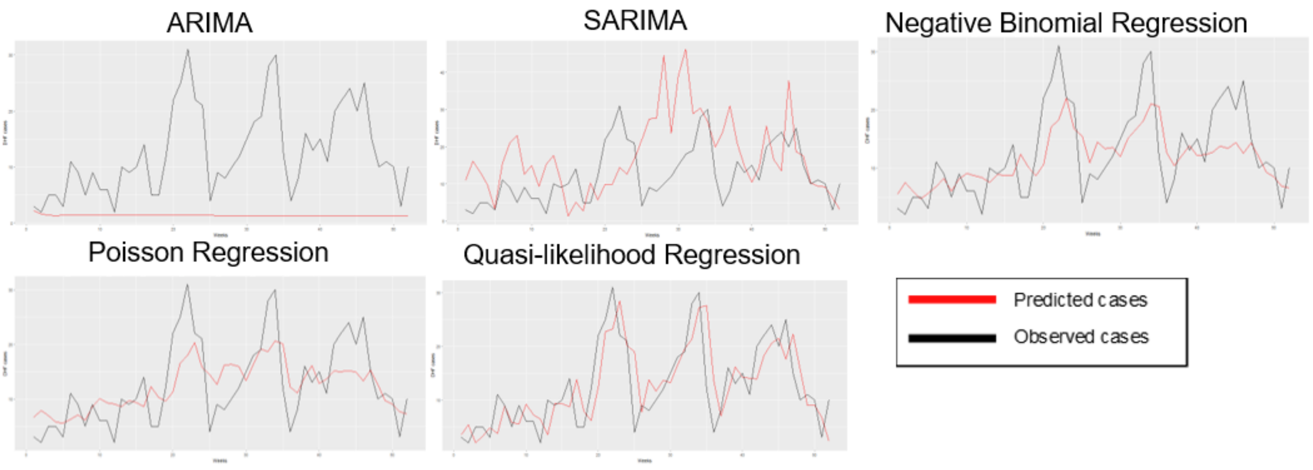

Figure 113: Plots between dengue cases and weeks, the black line represents the observed dengue cases, and the red line represents the predicted dengue cases of the best fit model of each technique over the test set data starting from January 2014 to December 2014.

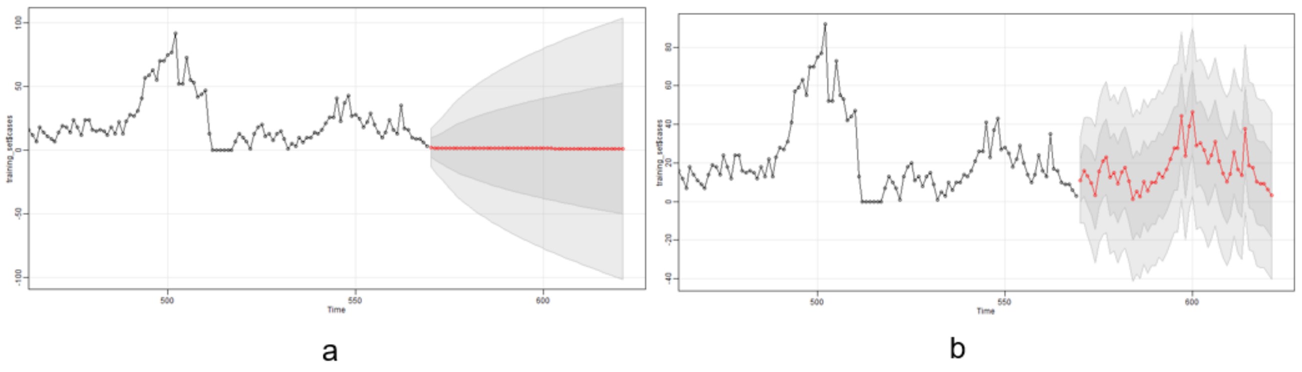

Figure 114: (a) Plot between dengue incidences over weekly time by the best model of ARIMA and (b) SARIMA time series analysis, the black line represents training set data starting from January 2012 to December 2013, and the red line represents the forecasted dengue incidences from January 2014 to December 2014.

Table 38: Coefficients and significant values of best fit GLM models, Negative Binomial, Poisson and Quasi-likelihood regression model of Lopburi. The table summarizes coefficients of each independent variables which are composed in best fit model of each method. The significant of each variable is labelled by asterisks under the coefficients. The most important factor is marked as three asterisks which p-value ranges from 0 to 0.001. The second important factor is marked as two asterisks which p-value ranges from 0.001 to 0.01. The third important factor is marked as an asterisk which p-value ranges from 0.01 to 0.1. The least important is also marked as a dot which p-value ranges from 0.1 to 1.

**Independent variables**	**Lag**	**Coefficients/Significant**		
		NB	Poisson	Quasi
Intercept		70.594727<br>***	96.5341220<br>***	707.286134<br>**
Cases	1	0.031341<br>***	0.0263230<br>***	0.843835<br>***
	2			
	3	0.004172<br>.		
Average Pressure	0			
	1			
	2			
	3	-0.067036<br>***	-0.0928802<br>***	-0.688398<br>**
Minimum Temperature	0	0.051866<br>**		
	1		0.0287281<br>**	
	2			
	3			
Maximum Temperature	0		0.0205271<br>*	0.284809
	1			
	2			
	3	-0.050357<br>**	-0.0570512<br>***	-0.506522<br>*
Relative Humidity	0			-0.046447
	1	-0.007868<br>*	-0.0053680<br>*	
	2			
	3		0.0058130<br>**	
Precipitation	0			
	1			
	2			
	3	0.008944<br>.		0.160791<br>*
Vaporization	0			
	1			
	2			
	3			
Wind Direction	0			0.010961
	1			
	2		-0.0008804<br>***	-0.009669
	3			
Wind Power	0			
	1			
	2			
	3			

Mae Hong Son

Mae Hong Son is a remote, mountainous province in northern Thailand at 9°18′4″N 97°58′12″E. Mae Hong Son covers an area of 12,681 km^2. Total population are 248,178 people. The density of population is 20.0 km^2. Weather at Mae Hong Son has tropical savanna climate under the South Asian monsoon system. Temperature is in the range from the low of 13.8°C in January to the high of 44.6°C in April. The rainy season begins with the arrival of the southwest monsoon around mid-May. The humidity presents the average of 95 percent. Precipitation occurs from mid-May to August. The average precipitation is in the range of 226.9 - 239.3 mm annually

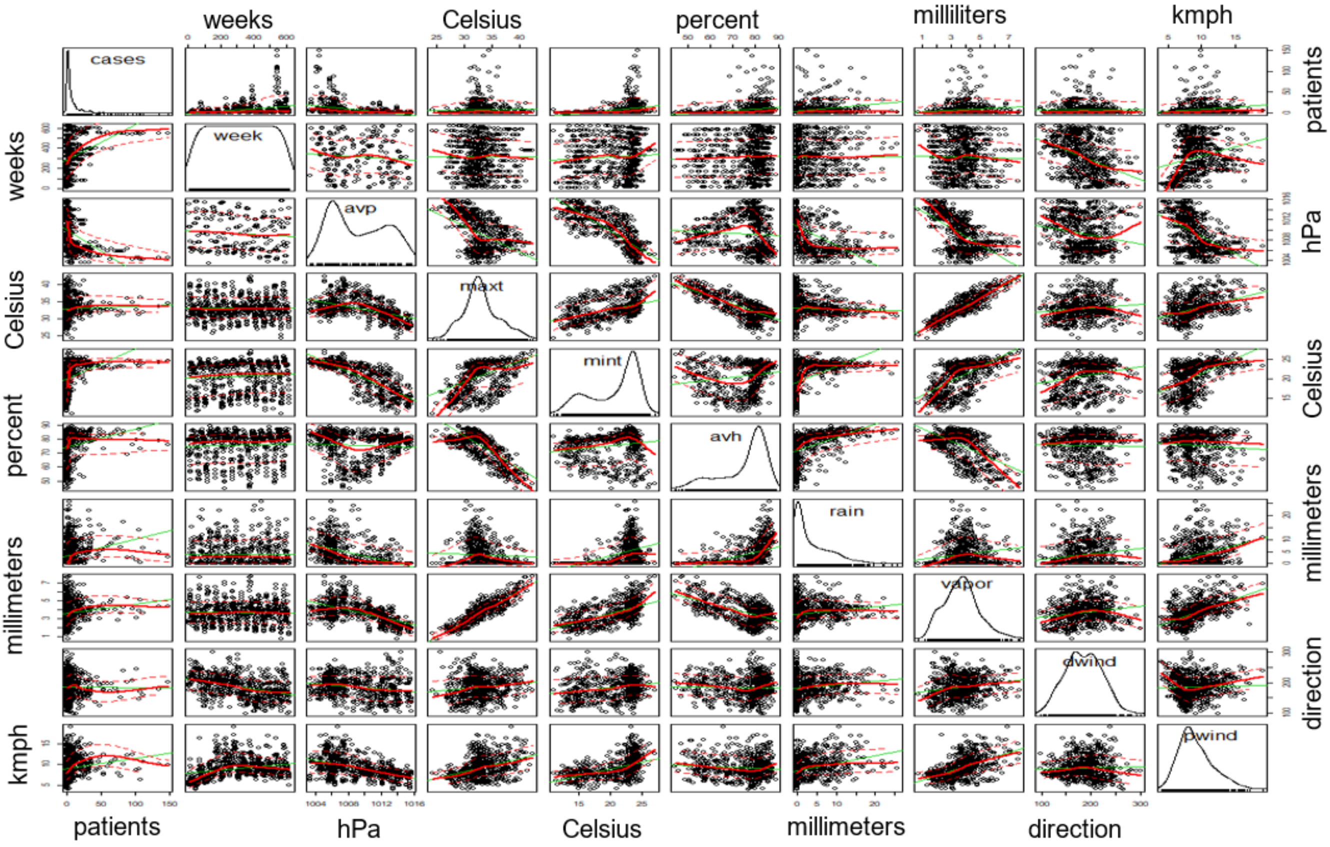

Figure 115: Scatter plot between dengue cases (cases) and selected independent variables, which are the weekly period starting from January 2001 – December 2013 (week), average pressure (avp), maximum temperature (maxt), minimum temperature (mint), average humidity (avh), precipitation (rain), vaporization of water (vapor), wind direction (dwind), and wind power (pwind). The plot visualizes pairwise hundred relationships of training set in Mae Hong Son.

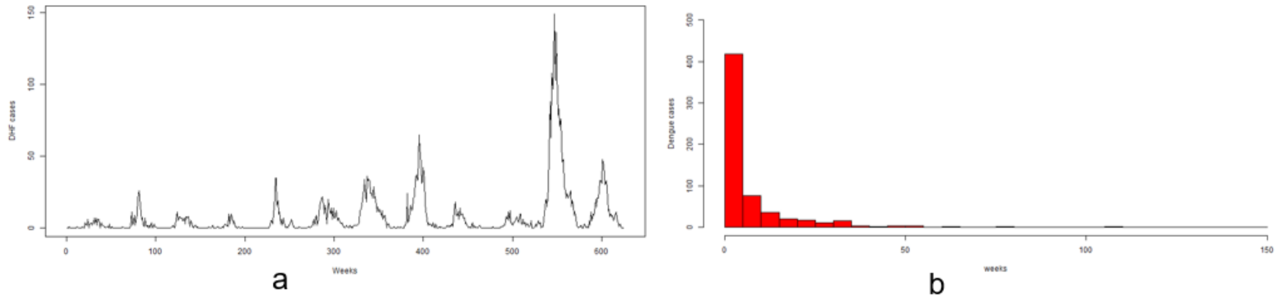

Figure 116: (a) Line plot between dengue incidences and weeks, the plot shows trends of dengue incidences in each year as stationary time series. (b) Histogram of dengue incidences in Mae Hong Son starting from January 2001 to December 2013 (624 weeks).

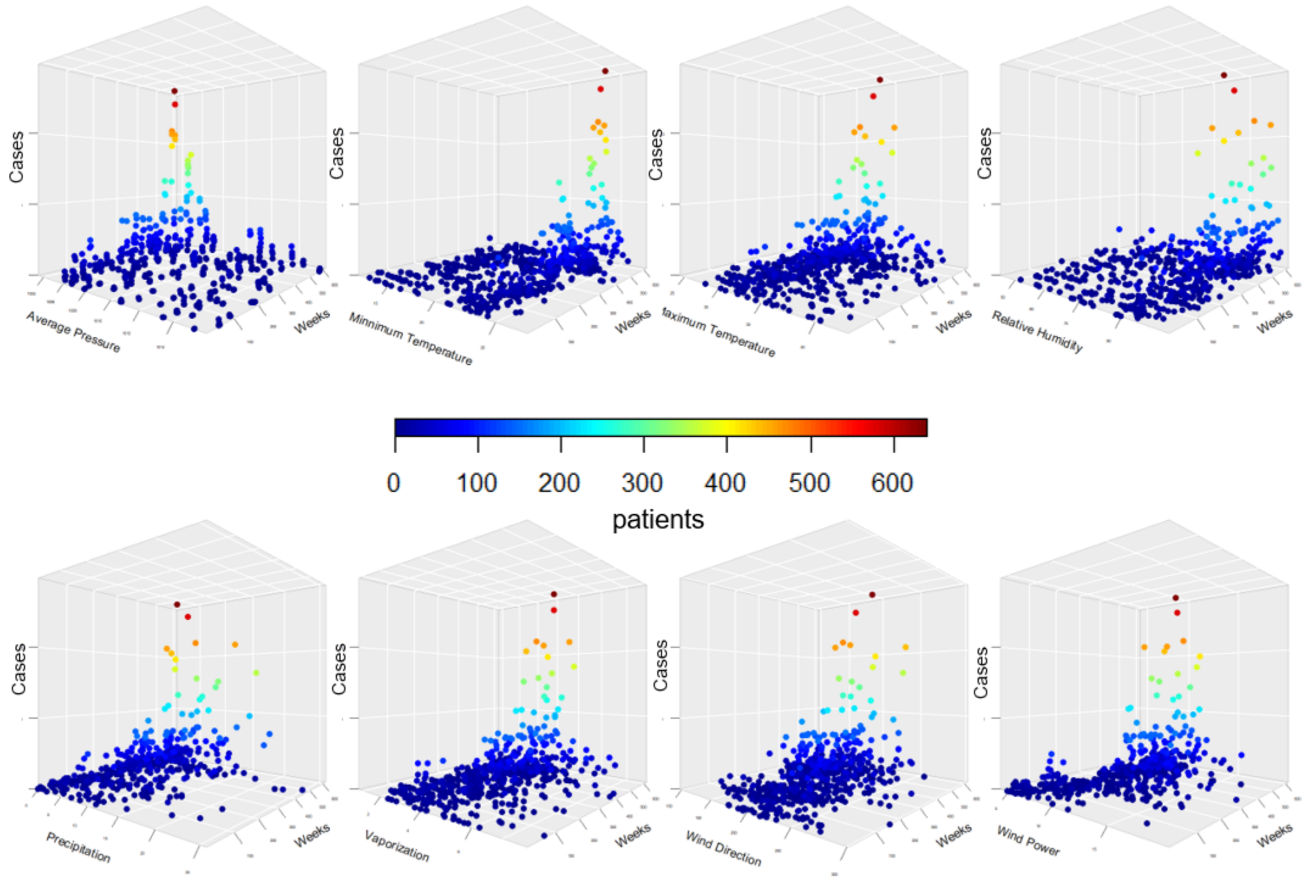

Figure 117: Three-dimensional scatter plot between dengue incidences and weather effects starting from January 2001 to December 2013 of Mae Hong Son.

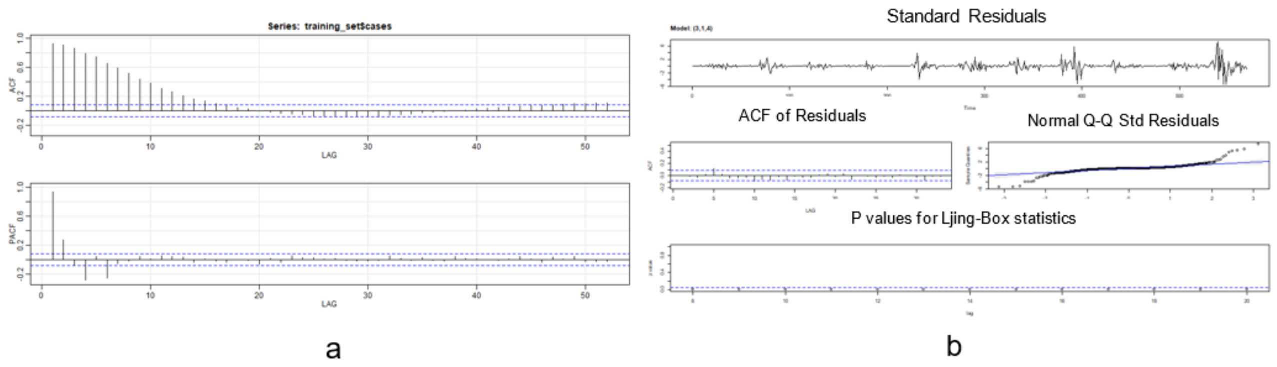

Figure 118: (a) Two plots between lag-time of dengue incidences and ACF and PACF relationship calculated from ARIMA model (b) Summary plots of time series analysis, multiple plots include the plot of predicted model over the time, the plot of ACF residual over lag-time of dengue incidences, residual Q-Q plot of standard residual, and p-value for Ljung-Box statistics of PACF relationship in Mae Hong Son over the training data starting from January 2001 to December 2013.

The best model in Mae Hong Son is based on Negative Binomial regression method which is included the 11 variables. The significant variables are 1-week-lag cases, 1-week-lag relative humidity, 3-week-lag vaporization, current week average pressure and 1-week-lag maximum temperature. Other less significant variables include 3-week-lag cases, current week maximum temperature, 3-week-lag maximum temperature, 2-week-lag precipitation and 2-week-lag precipitation. This correlation coefficient results 0.909 (95%CI: 0.8546, 0.9634). For time series methods show the correlation coefficient in 0.209 for ARIMA.

Table 39: Comparison table of all methods by the highest correlation coefficient (R^2) and the lowest prediction error (RMSE) in Mae Hong Son.

**Methods**	**R-squared (R^2)**	**Root mean square error (RMSE)**
Poisson Regression	0.8002993	5.659586
Negative Binomial Regression	0.909180	3.816679
Quasi-likelihood Regression	0.880623	4.375779
ARIMA (3,1,4)	0.209259	18.82427
SARIMA $(2,0,1)(0,2,0)_{52}$	-10.57309	43.08435

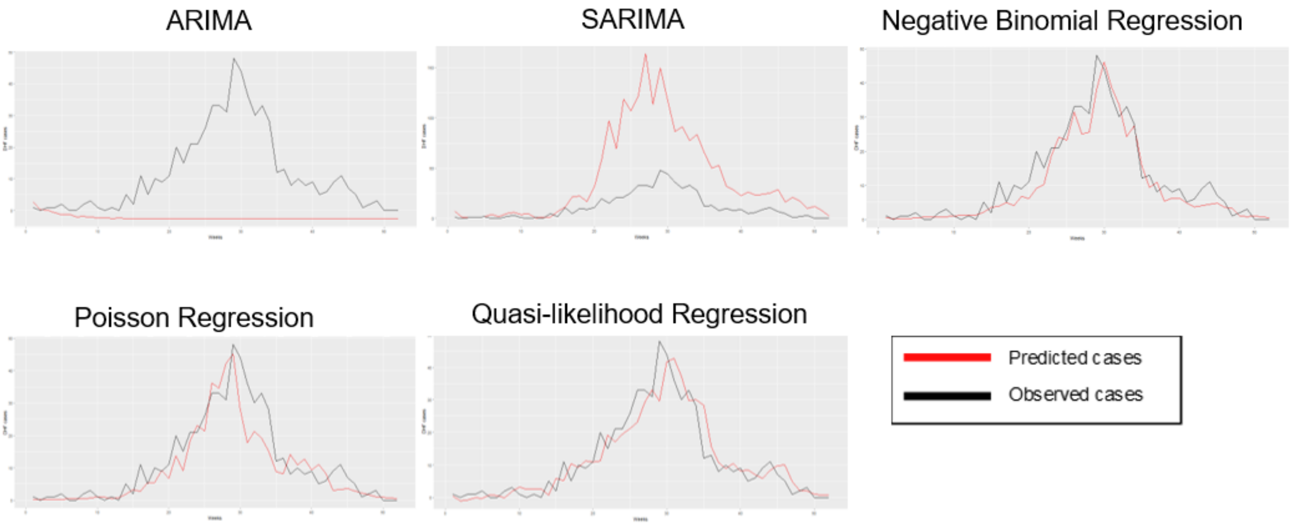

Figure 119: Plots between dengue cases and weeks, the black line represents the observed dengue cases, and the red line represents the predicted dengue cases of the best fit model of each technique over the test set data starting from January 2014 to December 2014.

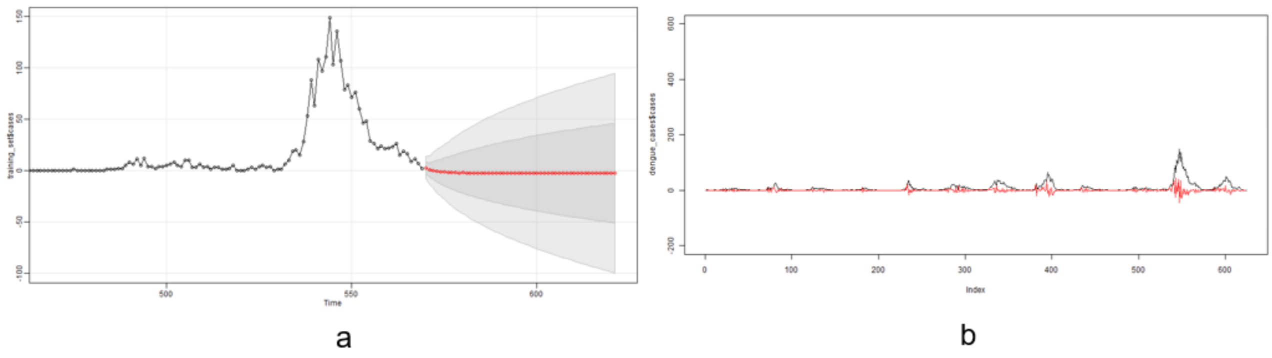

Figure 120: (a) Plot between dengue incidences over weekly time by the best model of ARIMA and (b) SARIMA time series analysis, the black line represents training set data starting from January 2012 to December 2013, and the red line represents the forecasted dengue incidences from January 2014 to December 2014.

Table 40: Coefficients and significant values of best fit GLM models, Negative Binomial, Poisson and Quasi-likelihood regression model of Mae Hong Son. The table summarizes coefficients of each independent variables which are composed in best fit model of each method. The significant of each variable is labelled by asterisks under the coefficients. The most important factor is marked as three asterisks which p-value ranges from 0 to 0.001. The second important factor is marked as two asterisks which p-value ranges from 0.001 to 0.01. The third important factor is marked as an asterisk which p-value ranges from 0.01 to 0.1. The least important is also marked as a dot which p-value ranges from 0.1 to 1.

**Independent variables**	**Lag**	**Coefficients/Significant**		
		NB	Poisson	Quasi
Intercept		98.09115 ***	323.25190 ***	257.9685 *
Cases	1	0.041865 ***		0.64935 ***
	2			0.29196 ***
	3	0.009083		
Average Pressure	0	-0.11263 ***	-0.155131 ***	-0.25769 *
	1			
	2			
	3		-0.173219	
Minimum Temperature	0			
	1		-0.064650 **	
	2		0.056970 *	
	3		0.118821 ***	
Maximum Temperature	0	-0.01867		
	1	0.170972 **		
	2		-0.021709 .	
	3	0.144054		0.061162
Relative Humidity	0			
	1	0.073996 ***	0.059676 ***	0.14765 *
	2	0.017529		
	3			-0.12951 **
Precipitation	0			-0.10476
	1			-0.13390
	2	0.019947	-0.031029 ***	-0.07029 .
	3		-0.036309 ***	
Vaporization	0			
	1			0.12184
	2			
	3	-0.04902 ***		
Wind Direction	0			
	1			
	2	-0.00107	-0.005136 ***	
	3			
Wind Power	0		0.010499 ***	0.09613
	1		-0.029072 ***	
	2			0.11454
	3			

Maha Sarakham

Maha Sarakham is located in the northeastern continent of Thailand at coordinate of 16°10′38″N 103°18′03″E. Maha Sarakham covers an area of 5,292 km^2. Total population are 960,588 people. The density of population is approximately 182.0 people per km^2. Weather in Maha Sarakham has tropical savanna climate under the South Asian monsoon system. Temperature is high in April approximately 42.5 °C and starts to low temperature from December to February (7.7-11.1°C). In winter, Maha Sarakham is dry and warm. The monsoon season begins from May through October. The highest rainfall presents in August around 350.7 mm. Humidity is in range from 62-84 percent throughout the year. The highest sunshine hours are in January and March.

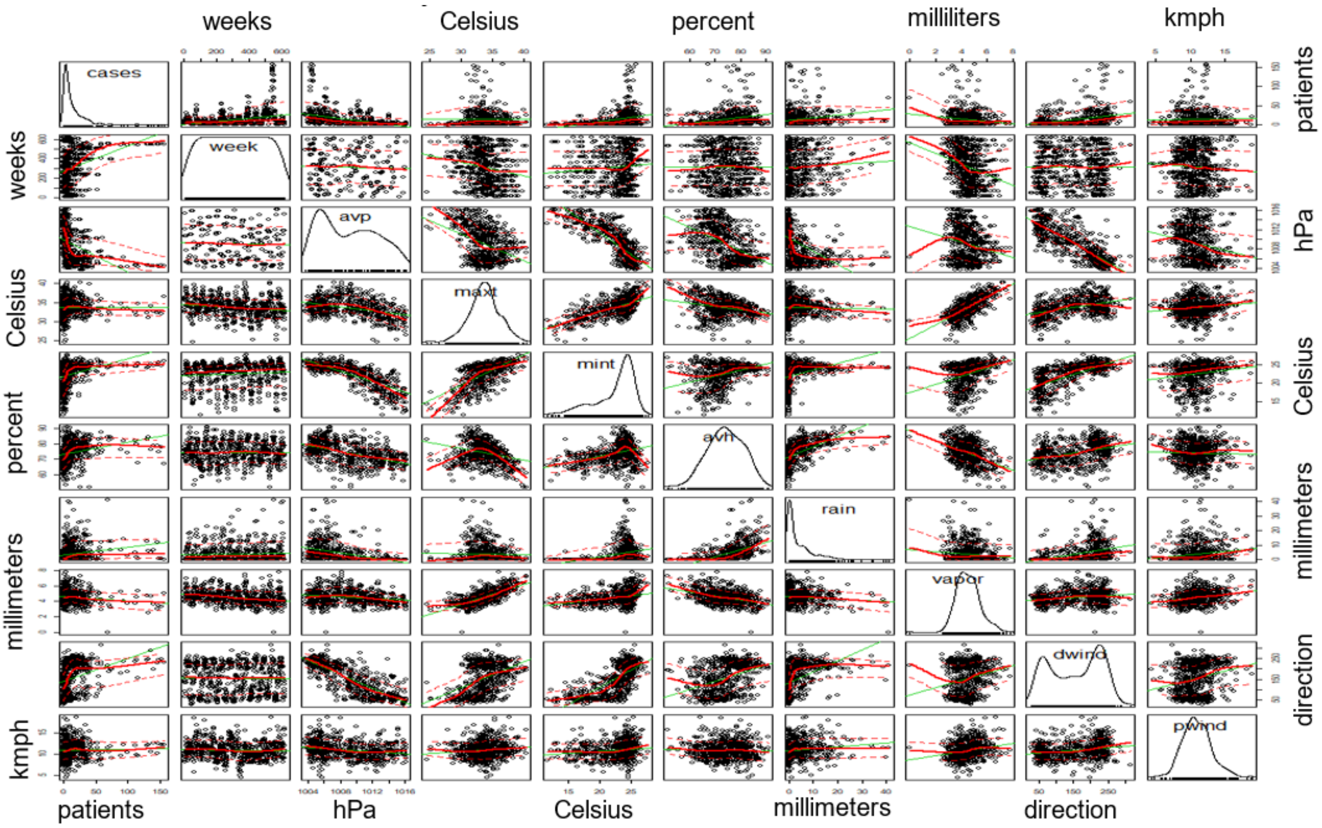

Figure 121: Scatter plot between dengue cases (cases) and selected independent variables, which are the weekly period starting from January 2001 – December 2013 (week), average pressure (avp), maximum temperature (maxt), minimum temperature (mint), average humidity (avh), precipitation (rain), vaporization of water (vapor), wind direction (dwind), and wind power (pwind). The plot visualizes pairwise hundred relationships of training set in Maha Sarakham.

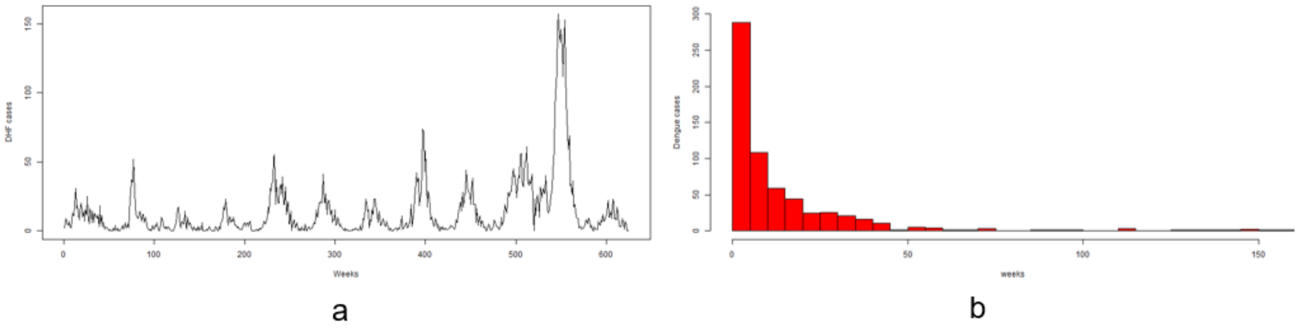

Figure 122: (a) Line plot between dengue incidences and weeks, the plot shows trends of dengue incidences in each year as stationary time series. (b) Histogram of dengue incidences in Maha Sarakham starting from January 2001 to December 2013 (624 weeks).

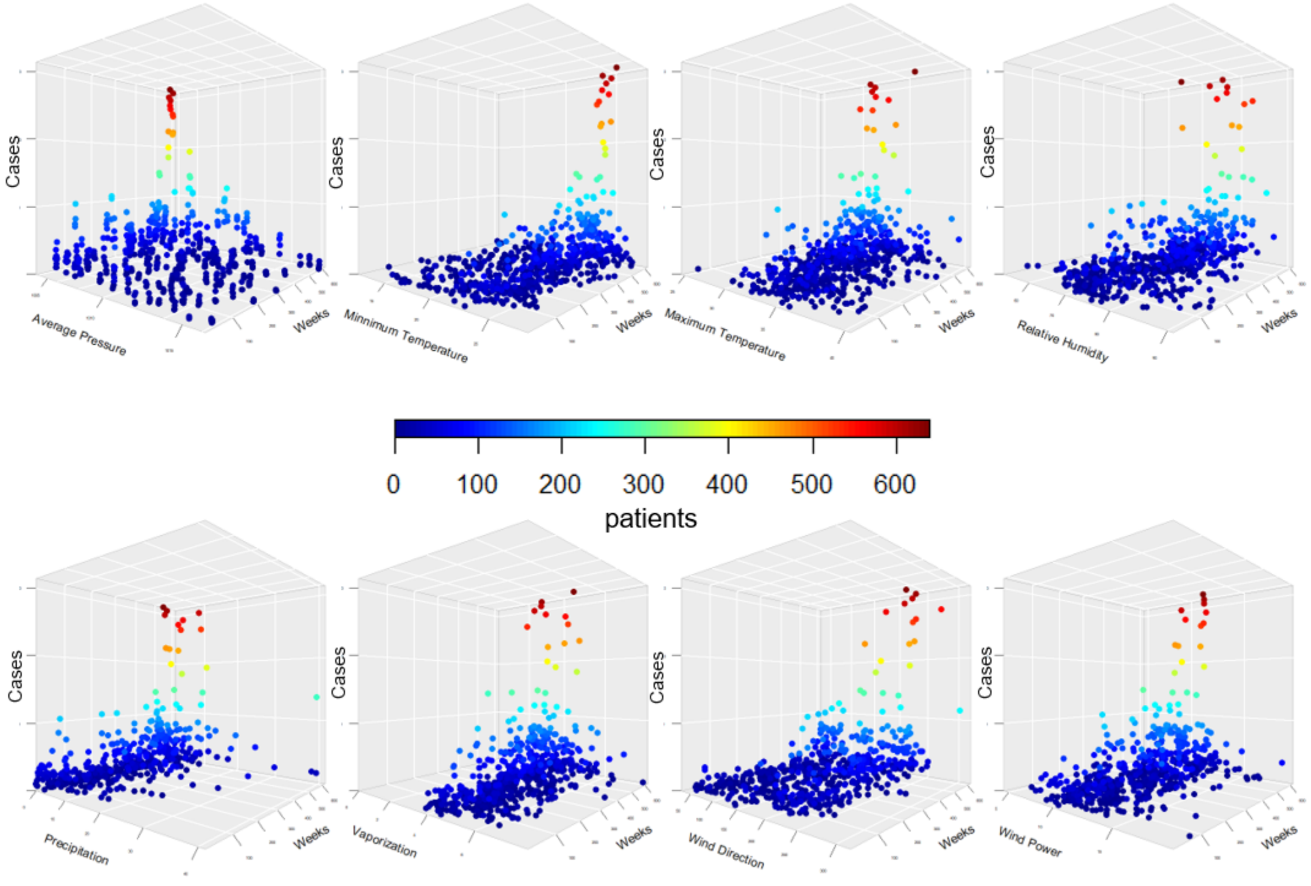

Figure 123: Three-dimensional scatter plot between dengue incidences and weather effects starting from January 2001 to December 2013 of Maha Sarakham.

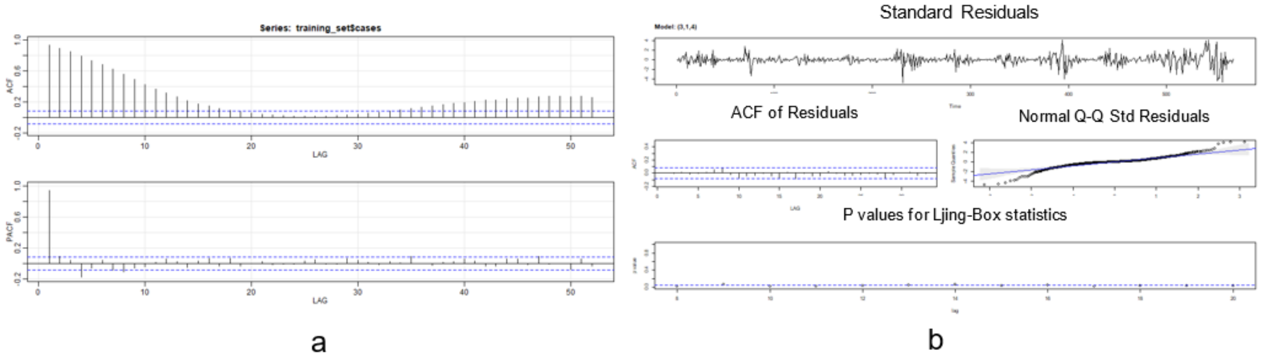

Figure 124: (a) Two plots between lag-time of dengue incidences and ACF and PACF relationship calculated from ARIMA model (b) Summary plots of time series analysis, multiple plots include the plot of predicted model over the time, the plot of ACF residual over lag-time of dengue incidences, residual Q-Q plot of standard residual, and p-value for Ljung-Box statistics of PACF relationship in Maha Sarakham over the training data starting from January 2001 to December 2013.

Maha Sarakham province, the best model is based on quasi-likelihood method. The correlation coefficient on the test set in 2014 is 0.913 (95%CI: 0.8816, 0.9444). The model uses 8 variables. The most significant variable are 1-week-lag cases and 3-week-lag cases. Other variables which have less significant are, 3-week-lag relative humidity,1-week-lag vaporization, current week, 1-week-lag, 2-week-lag and 3-week-lag wind power. Time series methods by ARIMA and SARIMA yield the correlation coefficient of -0.9186835 and -163.507 respectively.

Table 41: Comparison table of all methods by the highest correlation coefficient (R^2) and the lowest prediction error (RMSE) in Maha Sarakham.

Methods	R-squared (R^2)	Root mean square error (RMSE)
Poisson Regression	0.0549594	38.78763
Negative Binomial Regression	0.06803789	38.5183
Quasi-likelihood Regression	0.9129852	11.76968
ARIMA (3,1,4)	-0.9186835	7.471653
SARIMA $(2,0,1)(0,2,0)_{52}$	-163.507	69.18424

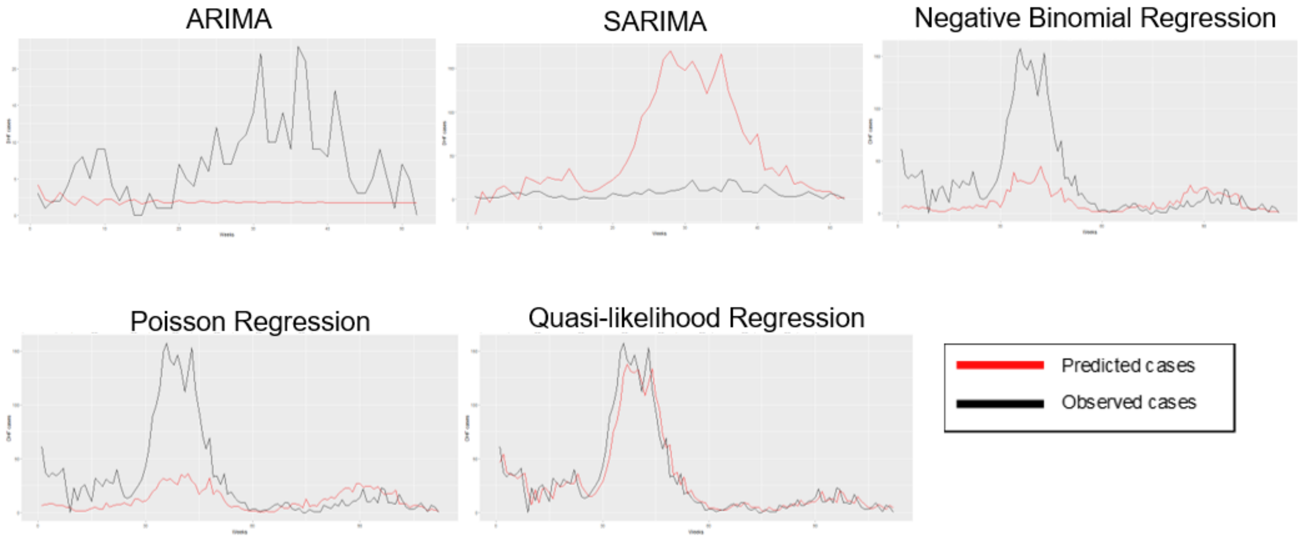

Figure 125: Plots between dengue cases and weeks, the black line represents the observed dengue cases, and the red line represents the predicted dengue cases of the best fit model of each technique over the test set data starting from January 2014 to December 2014.

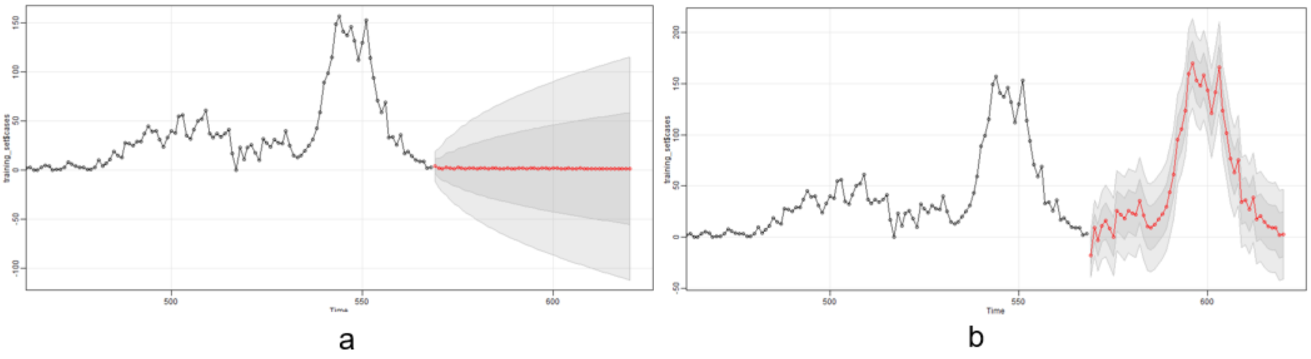

Figure 126: (a) Plot between dengue incidences over weekly time by the best model of ARIMA and (b) SARIMA time series analysis, the black line represents training set data starting from January 2012 to December 2013, and the red line represents the forecasted dengue incidences from January 2014 to December 2014.

Table 42: Coefficients and significant values of best fit GLM models, Negative Binomial, Poisson and Quasi-likelihood regression model of Maha Sarakham. The table summarizes coefficients of each independent variables which are composed in best fit model of each method. The significant of each variable is labelled by asterisks under the coefficients. The most important factor is marked as three asterisks which p-value ranges from 0 to 0.001. The second important factor is marked as two asterisks which p-value ranges from 0.001 to 0.01. The third important factor is marked as an asterisk which p-value ranges from 0.01 to 0.1. The least important is also marked as a dot which p-value ranges from 0.1 to 1.

**Independent variables**	**Lag**	**Coefficients/Significant**		
		NB	Poisson	Quasi
Intercept		293.49855 ***	204.6	0.493445
Cases	1			0.752293 ***
	2			
	3			0.159734 ***
Average Pressure	0	-0.28737 ***	-0.2022 ***	
	1			
	2			
	3			
Minimum Temperature	0			
	1		0.1756 ***	
	2			
	3			
Maximum Temperature	0			
	1			
	2			
	3	0.03527		
Relative Humidity	0			
	1			
	2			
	3			-0.22222
Precipitation	0			
	1			
	2			
	3			
Vaporization	0	-0.04178	-0.08231 ***	
	1	-0.09046	-0.1131 ***	0.248603
	2	-0.12036 .	-0.1169 ***	
	3	-0.17758 *	-0.1331 ***	
Wind Direction	0			
	1		-0.001525 ***	
	2			
	3			
Wind Power	0			-0.002151
	1			-0.128408
	2			0.130834
	3	-0.07344 ***	-0.02998 ***	0.094190

Mukdahan

Mukdahan is located in the northeastern continent of Thailand at coordinate of 16°32′35″N 104°43′22″E. Mukdahan covers an area of 4,340 km^2. Total population are 346,016 people. The density of population is approximately 80.0 people per km^2. Weather in Mukdahan has tropical savanna climate under the South Asian monsoon system. Temperature is high in April approximately 42.5°C and starts to low temperature from December to February (7.7-11.1°C). Winters are dry and warm. The monsoon season begins from May through October. The highest rainfall presents in August around 350.7 mm. Humidity is in range from 62-84 percent throughout the year. The highest sunshine hours are in January and March.

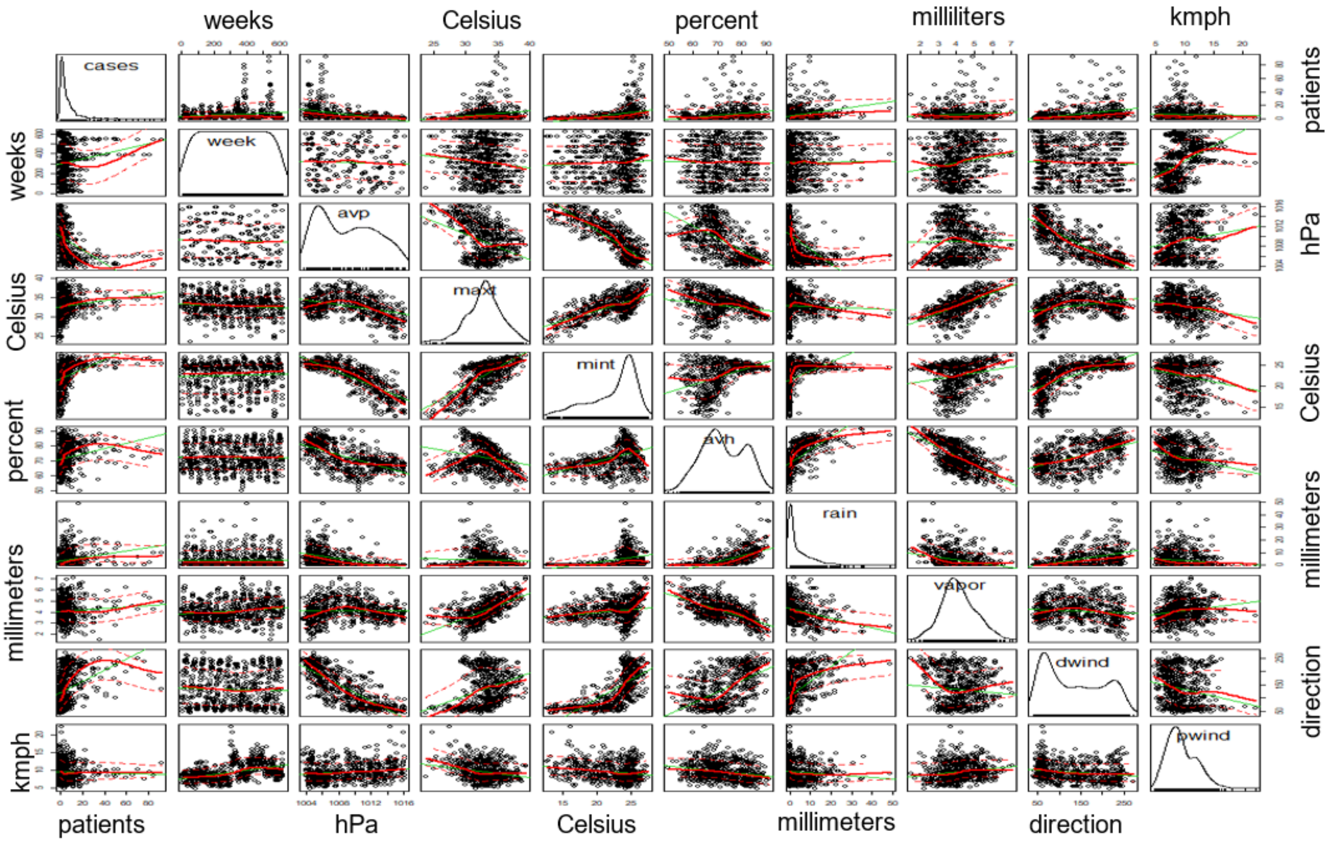

Figure 127: Scatter plot between dengue cases (cases) and selected independent variables, which are the weekly period starting from January 2001 – December 2013 (week), average pressure (avp), maximum temperature (maxt), minimum temperature (mint), average humidity (avh), precipitation (rain), vaporization of water (vapor), wind direction (dwind), and wind power (pwind). The plot visualizes pairwise hundred relationships of training set in Mukdahan.

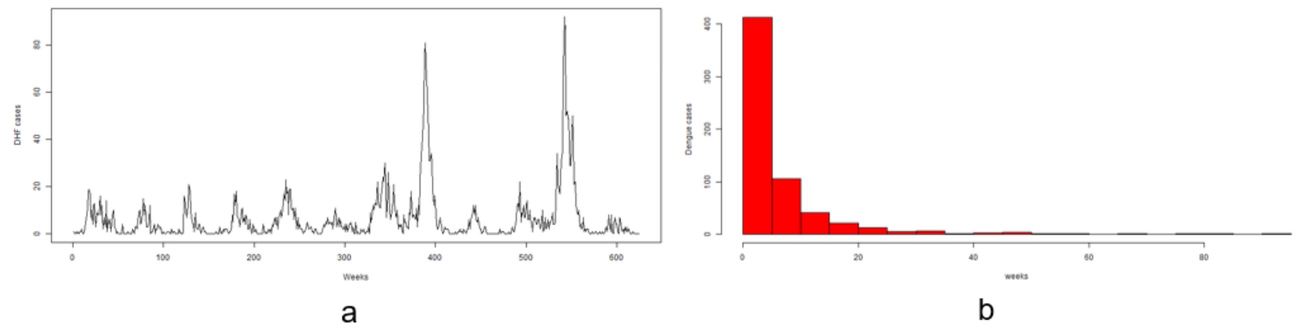

Figure 128: (a) Line plot between dengue incidences and weeks, the plot shows trends of dengue incidences in each year as stationary time series. (b) Histogram of dengue incidences in Mukdahan starting from January 2001 to December 2013 (624 weeks).

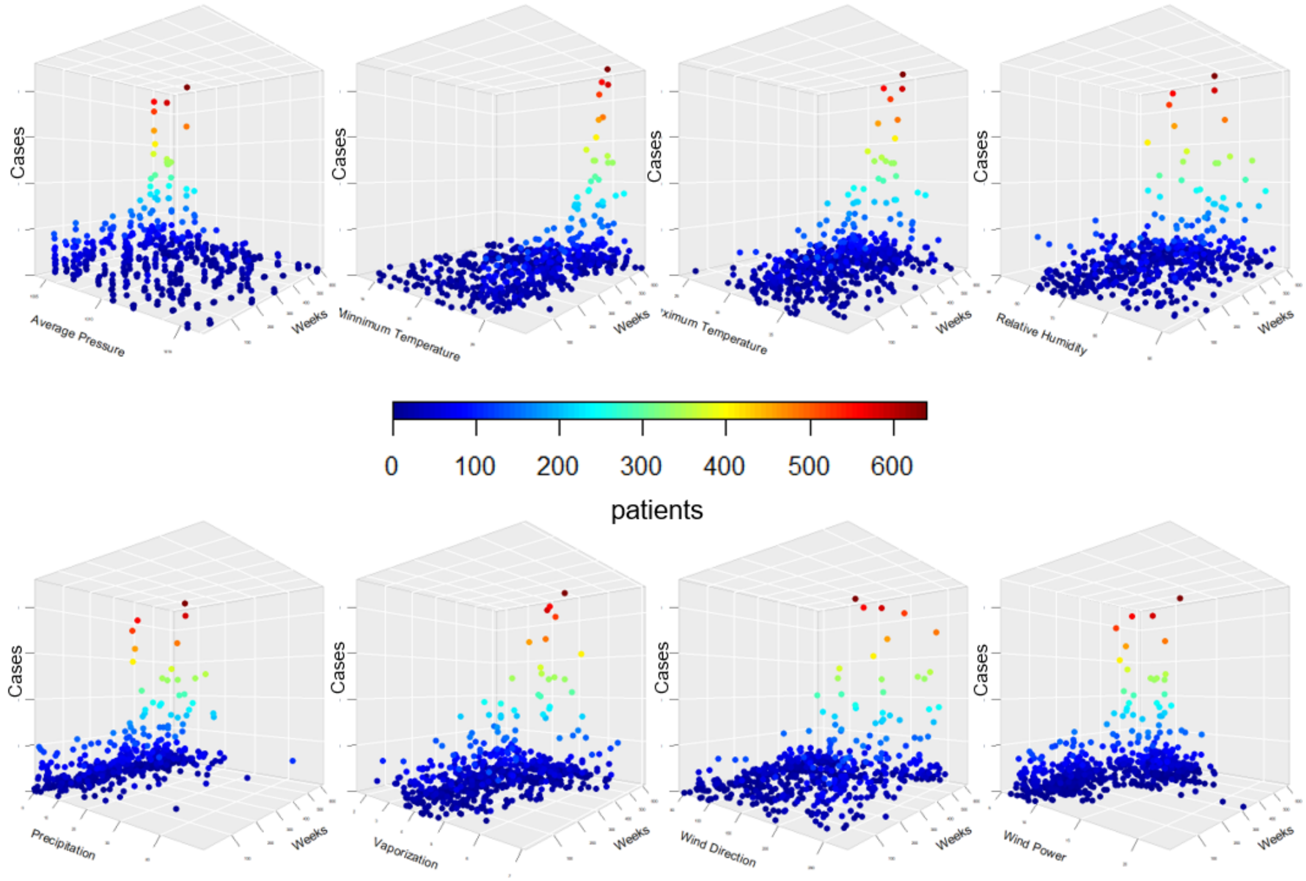

Figure 129: Three-dimensional scatter plot between dengue incidences and weather effects starting from January 2001 to December 2013 of Mukdahan.

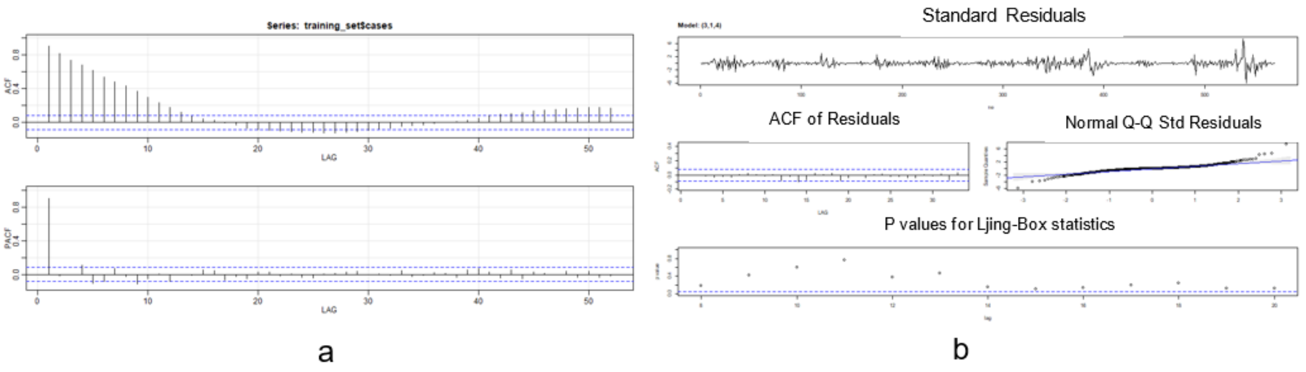

Figure 130: (a) Two plots between lag-time of dengue incidences and ACF and PACF relationship calculated from ARIMA model (b) Summary plots of time series analysis, multiple plots include the plot of predicted model over the time, the plot of ACF residual over lag-time of dengue incidences, residual Q-Q plot of standard residual, and p-value for Ljung-Box statistics of PACF relationship in mukdahan over the training data starting from January 2001 to December 2013.

For Mukdahan, the best model is based on quasi-likelihood method. The correlation coefficient on the test set in 2014 is 0.851 (95%CI: 0.8078, 0.8942). The model uses 8 variables. The most significant variables are 1-week-lag cases and 2-week-lag wind direction, following by 1-week-lag wind direction and 3-week-lag vaporization. Other variables which have less significant are, current week, 1-week-lag, 2-week-lag and 3-week-lag precipitation. Time series methods by ARIMA and SARIMA yield the correlation coefficient of -0.5827129 and -174.4691 respectively.

Table 43: Comparison table of all methods by the highest correlation coefficient (R^2) and the lowest prediction error (RMSE) in mukdahan.

Methods	R-squared (R^2)	Root mean square error (RMSE)
Poisson Regression	0.7977626	7.857146
Negative Binomial Regression	0.2092936	15.53609
Quasi-likelihood Regression	0.8511291	6.741228
ARIMA (3,1,4)	-0.5827129	2.810087
SARIMA $(2,0,1)(0,2,0)_{52}$	-174.4691	29.58824

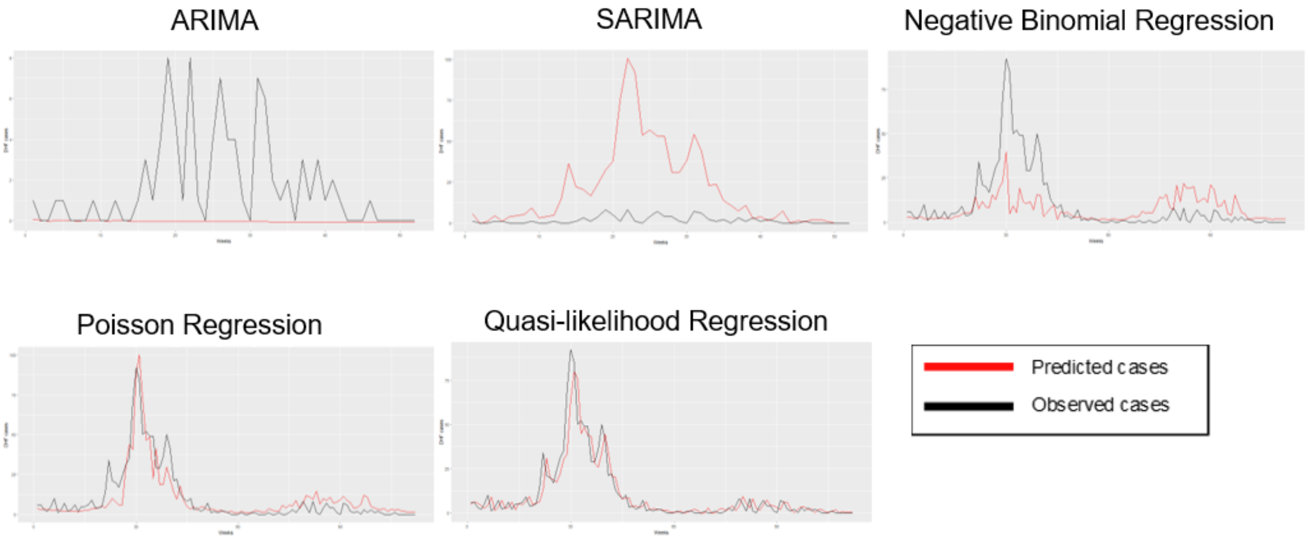

Figure 131: Plots between dengue cases and weeks, the black line represents the observed dengue cases, and the red line represents the predicted dengue cases of the best fit model of each technique over the test set data starting from January 2014 to December 2014.

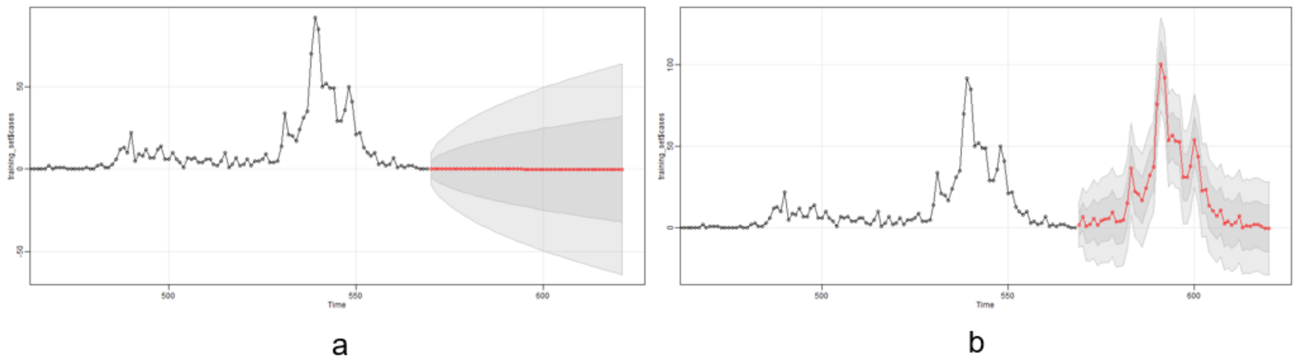

Figure 132: (a) Plot between dengue incidences over weekly time by the best model of ARIMA and (b) SARIMA time series analysis, the black line represents training set data starting from January 2012 to December 2013, and the red line represents the forecasted dengue incidences from January 2014 to December 2014.

Table 44: Coefficients and significant values of best fit GLM models, Negative Binomial, Poisson and Quasi-likelihood regression model of Mukdahan. The table summarizes coefficients of each independent variables which are composed in best fit model of each method. The significant of each variable is labelled by asterisks under the coefficients. The most important factor is marked as three asterisks which p-value ranges from 0 to 0.001. The second important factor is marked as two asterisks which p-value ranges from 0.001 to 0.01. The third important factor is marked as an asterisk which p-value ranges from 0.01 to 0.1. The least important is also marked as a dot which p-value ranges from 0.1 to 1.

**Independent variables**	**Lag**	**Coefficients/Significant**		
		NB	Poisson	Quasi
Intercept		-2.0308544 ***	-6.6301581 ***	-1.422878
Cases	1		0.0345032 ***	0.875705 ***
	2		-0.0089544 *	
	3		0.0056194 .	
Average Pressure	0			
	1			
	2			
	3			
Minimum Temperature	0			
	1			
	2			
	3			
Maximum Temperature	0		0.0581093 ***	
	1			
	2			
	3			
Relative Humidity	0		0.0501252 ***	
	1			
	2			
	3			
Precipitation	0			0.39400
	1	0.0150874 .		-0.013386
	2	0.0124686	0.0067852 *	0.030896
	3	0.0407455 ***		0.043822
Vaporization	0	0.2068392 ***		
	1			
	2			
	3	0.3097542 ***	0.4134155 ***	0.494554 *
Wind Direction	0		0.0041059 ***	
	1	0.0081916 ***		0.014590 **
	2			-0.016718 ***
	3			
Wind Power	0			
	1			
	2			
	3			

Nakhon Pathom

Nakhon Pathom is located in the central continent of Thailand at coordinate of 13°49′14″N 100°03′45″E. Nakhon Pathom covers an area of 2,168 km^2. Total population are 891,071 people. The density of population is 411.0 people per km^2. Weather in Nakhon Pathom follows tropical savanna climate system. The highest temperature presents in April. The low temperature presents in winter from December to March. The monsoon season starts from May through August. The highest sunshine hours are in January.

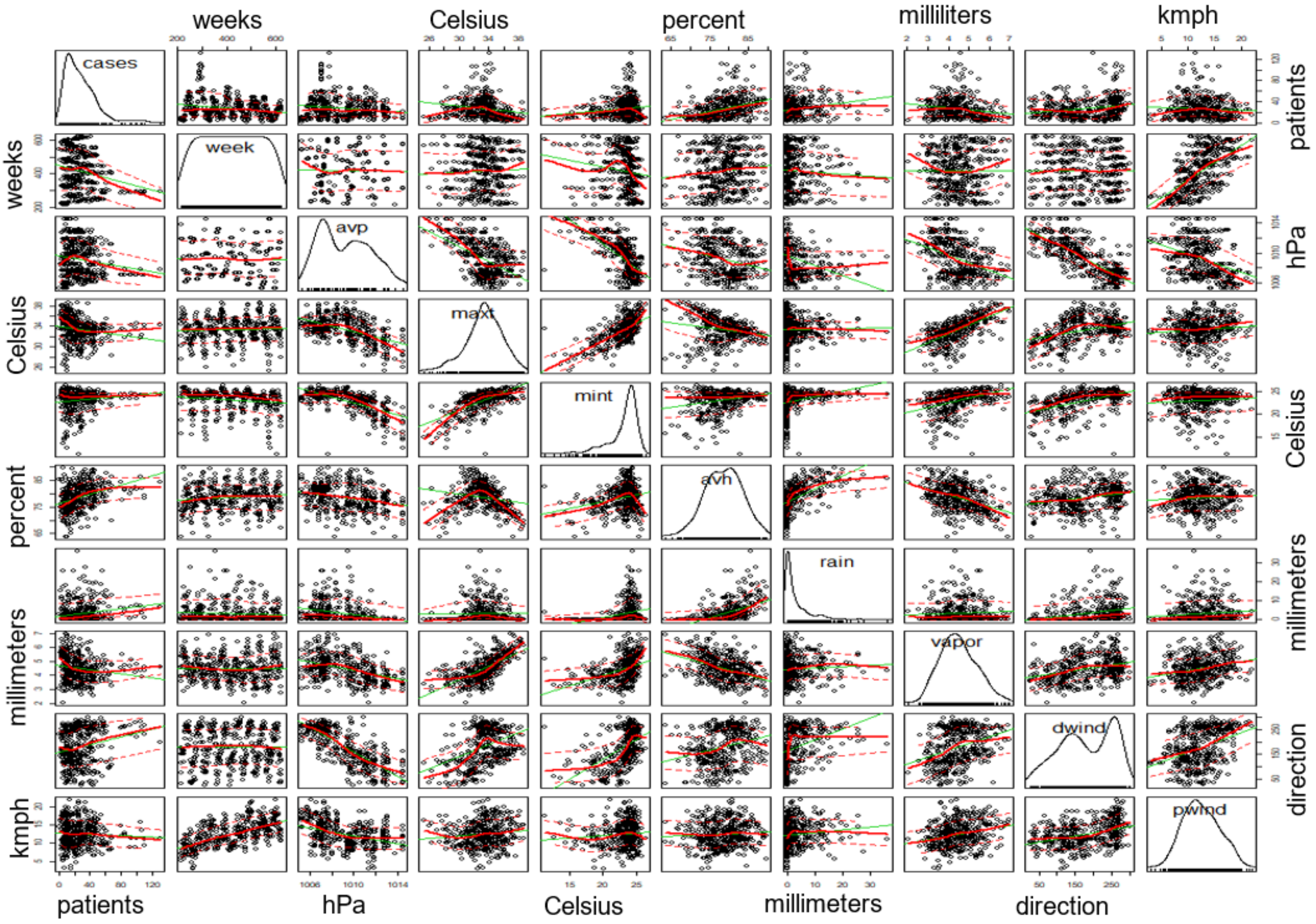

Figure 133: Scatter plot between dengue cases (cases) and selected independent variables, which are the weekly period starting from January 2001 – December 2013 (week), average pressure (avp), maximum temperature (maxt), minimum temperature (mint), average humidity (avh), precipitation (rain), vaporization of water (vapor), wind direction (dwind), and wind power (pwind). The plot visualizes pairwise hundred relationships of training set in Nakhon Pathom.

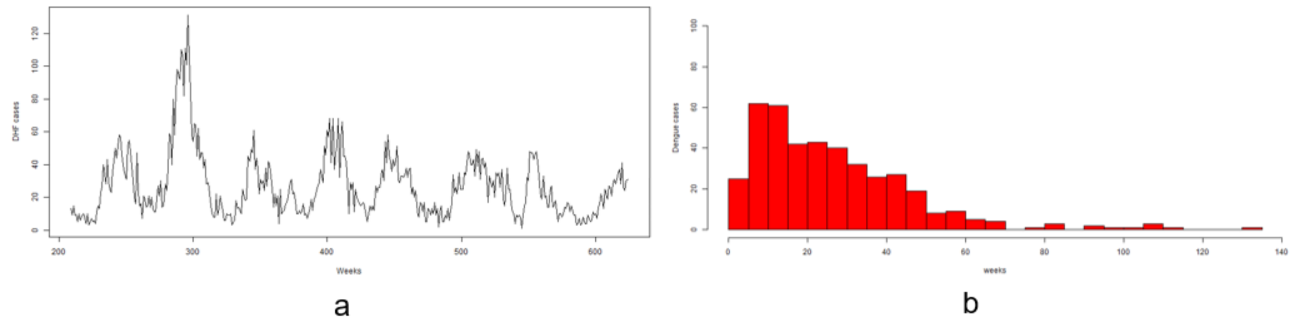

Figure 134: (a) Line plot between dengue incidences and weeks, the plot shows trends of dengue incidences in each year as stationary time series. (b) Histogram of dengue incidences in Nakhon Pathom starting from January 2001 to December 2013 (624 weeks).

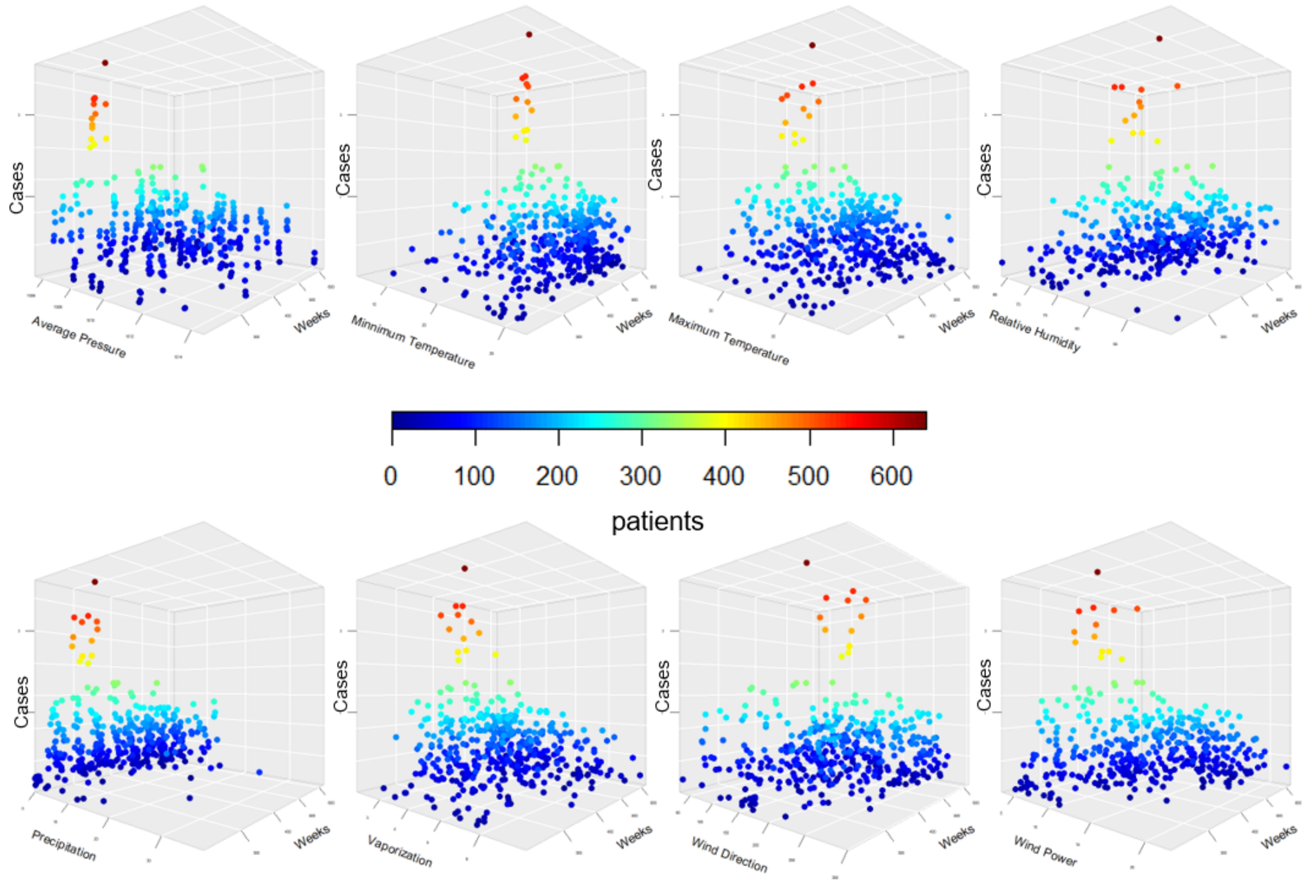

Figure 135: Three-dimensional scatter plot between dengue incidences and weather effects starting from January 2001 to December 2013 of Nakhon Pathom.

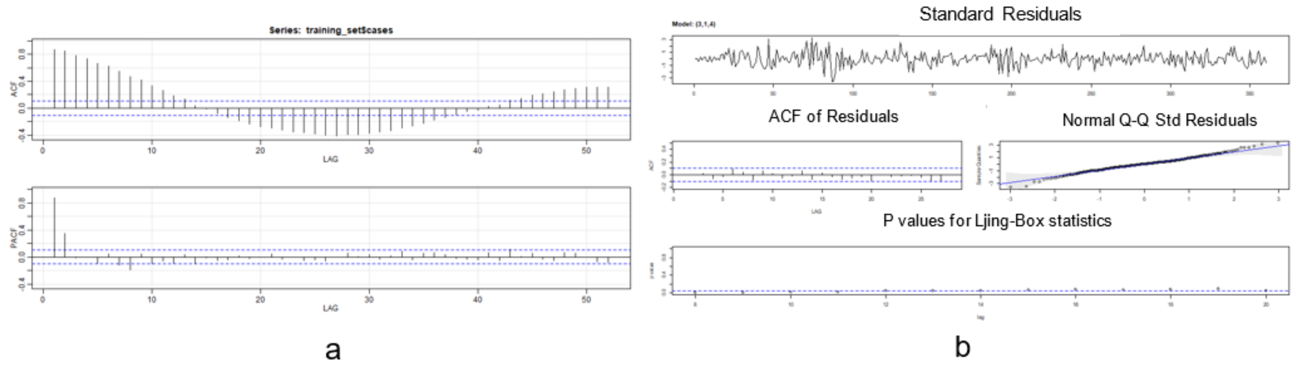

Figure 136: (a) Two plots between lag-time of dengue incidences and ACF and PACF relationship calculated from ARIMA model (b) Summary plots of time series analysis, multiple plots include the plot of predicted model over the time, the plot of ACF residual over lag-time of dengue incidences, residual Q-Q plot of standard residual, and p-value for Ljung-Box statistics of PACF relationship in Nakhon Pathom over the training data starting from January 2001 to December 2013.

For Nakhon Pathom, the best model is based on quasi-likelihood method. The correlation coefficient on the test set in 2014 is 0.83 (95%CI: 0.7646, 0.8954). The model consists of 9 variables. The most significant variables are 1-week-lag cases and 3-week-lag cases, following by 1-week-lag wind direction and 2-week-lag maximum temperature. Other variables which have less significant are, 2-week-lag minimum temperature, 1-week-lag relative humidity, current week and 1-week-lag precipitation, and current week wind power. Time series methods by ARIMA and SARIMA yield the correlation coeffect of -0.1792762 and -1.200071 respectively.

Table 45: Comparison table of all methods by the highest correlation coefficient (R^2) and the lowest prediction error (RMSE) in nakhonpathom.

**Methods**	**R-squared (R^2)**	**Root mean square error (RMSE)**
Poisson Regression	0.6595091	5.804468
Negative Binomial Regression	0.6992245	5.455454
Quasi-likelihood Regression	0.8301454	4.099664
ARIMA (3,1,4)	-0.1792762	10.80233
SARIMA $(2,0,1)(0,2,0)_{52}$	-1.200071	14.75462

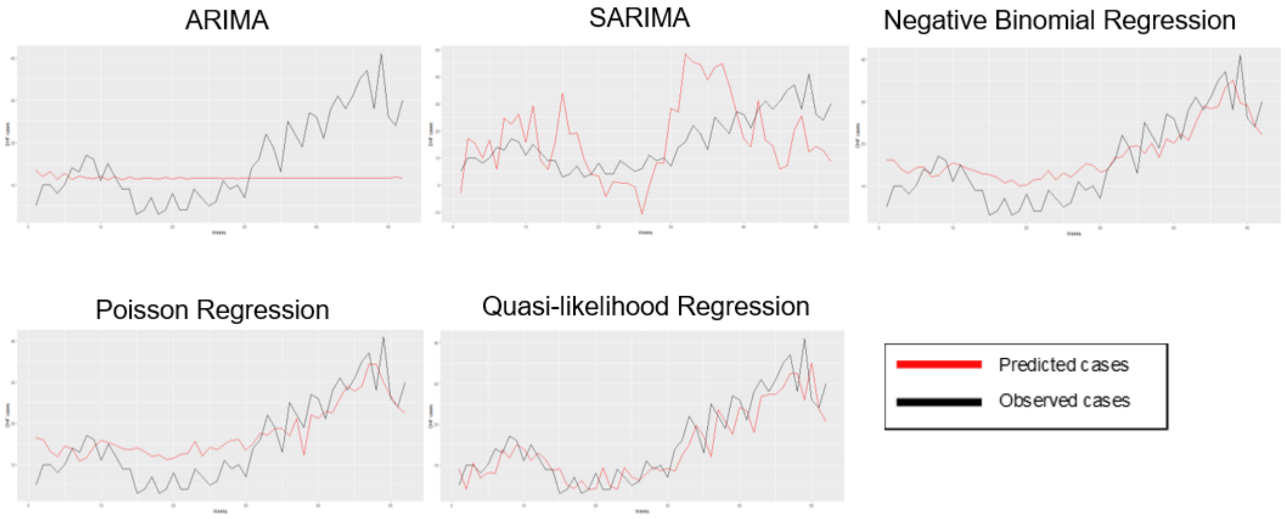

Figure 137: Plots between dengue cases and weeks, the black line represents the observed dengue cases, and the red line represents the predicted dengue cases of the best fit model of each technique over the test set data starting from January 2014 to December 2014.

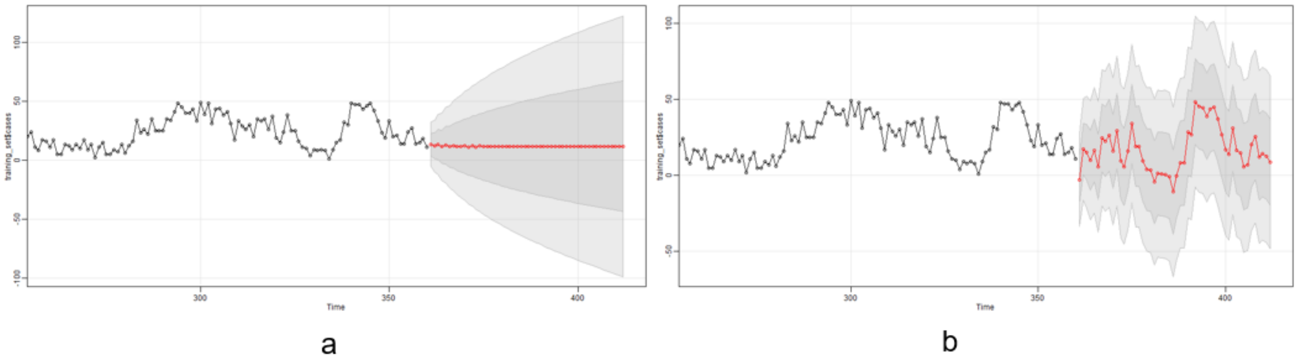

Figure 138: (a) Plot between dengue incidences over weekly time by the best model of ARIMA and (b) SARIMA time series analysis, the black line represents training set data starting from January 2012 to December 2013, and the red line represents the forecasted dengue incidences from January 2014 to December 2014.

Table 46: Coefficients and significant values of best fit GLM models, Negative Binomial, Poisson and Quasi-likelihood regression model of Nakhon Pathom. The table summarizes coefficients of each independent variables which are composed in best fit model of each method. The significant of each variable is labelled by asterisks under the coefficients. The most important factor is marked as three asterisks which p-value ranges from 0 to 0.001. The second important factor is marked as two asterisks which p-value ranges from 0.001 to 0.01. The third important factor is marked as an asterisk which p-value ranges from 0.01 to 0.1. The least important is also marked as a dot which p-value ranges from 0.1 to 1.

**Independent variables**	**Lag**	**Coefficients/Significant**		
		NB	Poisson	Quasi
Intercept		1.601714	1.8081460	6.172082
		**	***	
Cases	1	0.015544	0.0129578	0.684838
		***	***	***
	2	0.008664	0.0068857	
		***	***	
	3			0.166089
				***
Average Pressure	0			
	1			
	2			
	3			
Minimum Temperature	0			
	1			
	2	0.027117	0.0555694	0.374272
		.	***	
	3			
Maximum Temperature	0			
	1			
	2	-0.036784	-0.0499234	-0.613041
		*	***	.
	3			
Relative Humidity	0			
	1			0.080701
	2			
	3	0.019620	0.0169148	
		***	***	
Precipitation	0			0.031084
	1	0.006261	0.0043199	0.040941
			*	
	2		0.0030005	
	3			
Vaporization	0	-0.016098	-0.0332996	
			*	
	1			
	2			
	3			
Wind Direction	0			
	1			0.027960
				**
	2			
	3			
Wind Power	0			-0.150402
	1			
	2			
	3			

Nakhon Phanom

Nakhon Phanom is located in the northeastern region of Thailand at coordinate of 17°24′25″N 104°46′51″E. Nakhon Phanom covers an area of 5,513 km^2. Total population are 713,351 people. The density of population is approximately 129.0 people per km^2. Weather in Nakhon Phanom has tropical savanna climate under the South Asian monsoon system. Temperature is high in April approximately 42.0°C and low temperatures are from December to February (4.3-8.2°C). Winter in Nakhon Phanom is dry and warm. The monsoon season begins from May through October. The highest rainfall presents in August around 252.2 mm. Humidity is in range from 67-88 percent throughout the year. The highest sunshine hours are in January

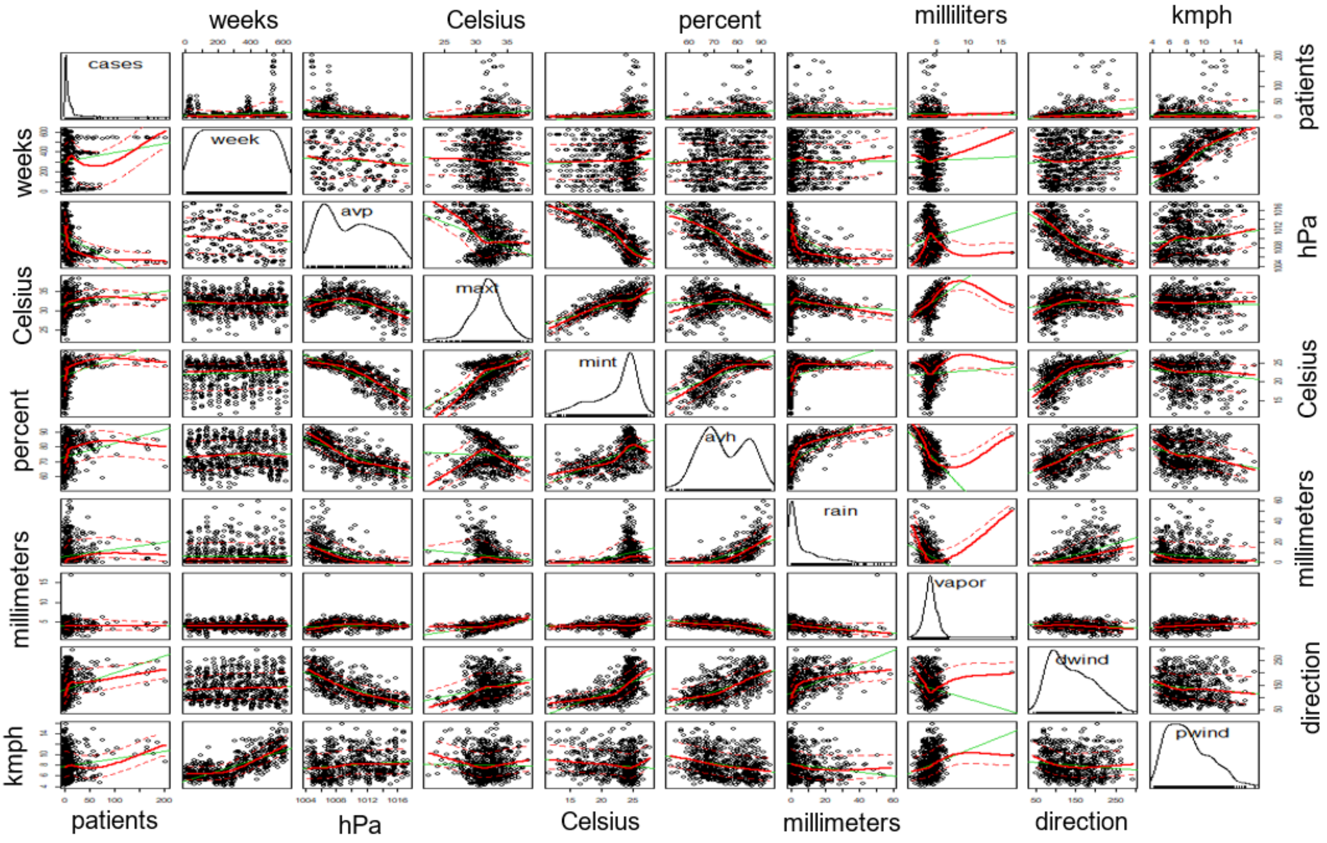

Figure 139: Scatter plot between dengue cases (cases) and selected independent variables, which are the weekly period starting from January 2001 – December 2013 (week), average pressure (avp), maximum temperature (maxt), minimum temperature (mint), average humidity (avh), precipitation (rain), vaporization of water (vapor), wind direction (dwind), and wind power (pwind). The plot visualizes pairwise hundred relationships of training set in Nakhon Phanom.

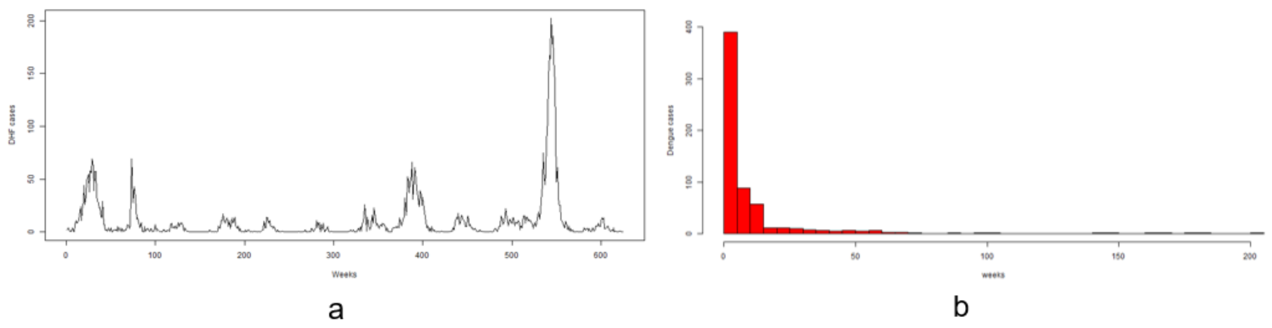

Figure 140: (a) Line plot between dengue incidences and weeks, the plot shows trends of dengue incidences in each year as stationary time series. (b) Histogram of dengue incidences in Nakhon Phanom starting from January 2001 to December 2013 (624 weeks).

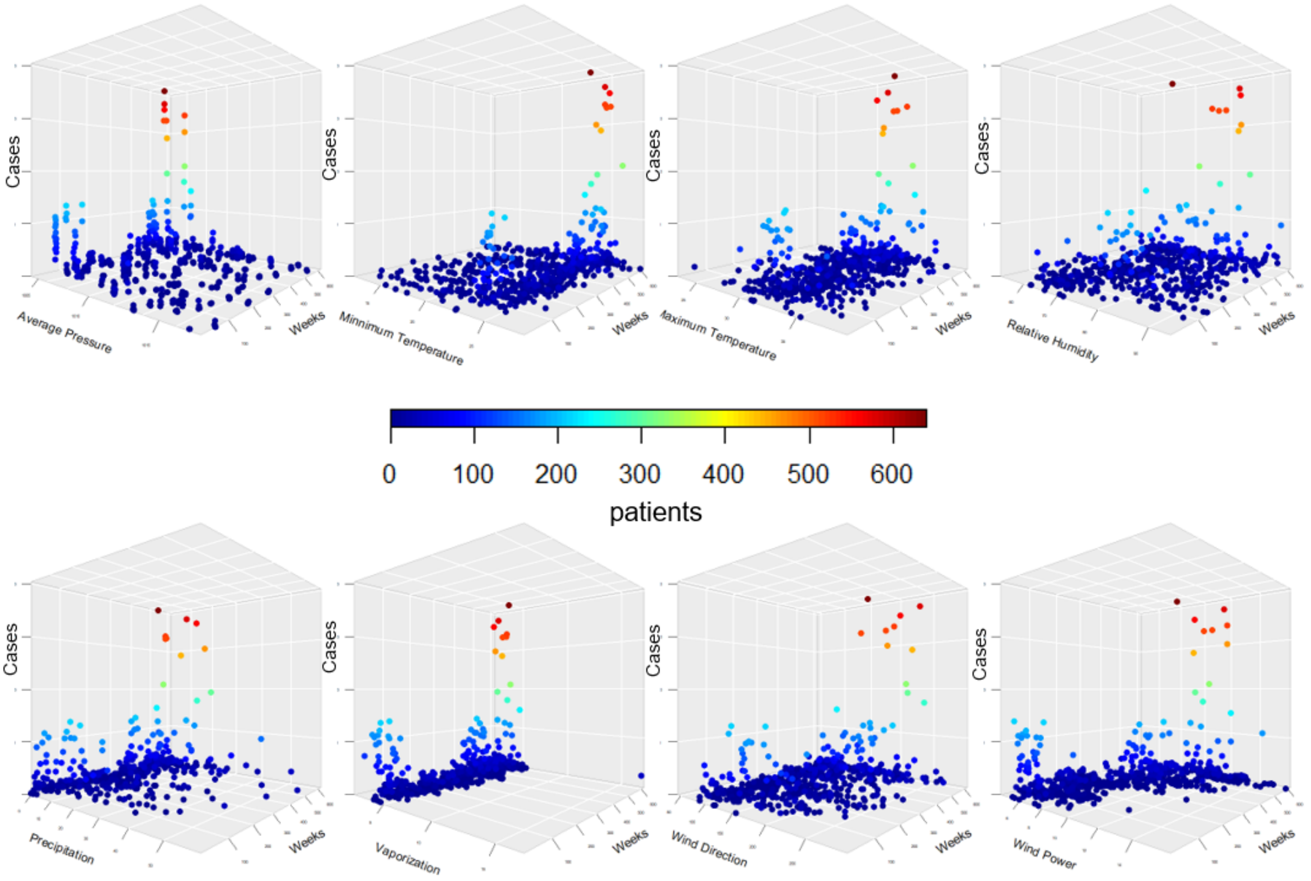

Figure 141: Three-dimensional scatter plot between dengue incidences and weather effects starting from January 2001 to December 2013 of Nakhon Phanom.

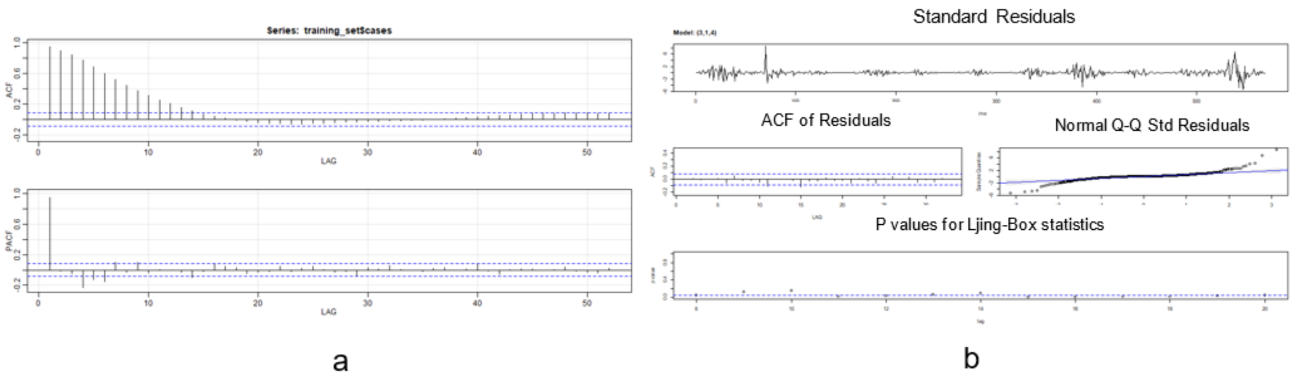

Figure 142: (a) Two plots between lag-time of dengue incidences and ACF and PACF relationship calculated from ARIMA model (b) Summary plots of time series analysis, multiple plots include the plot of predicted model over the time, the plot of ACF residual over lag-time of dengue incidences, residual Q-Q plot of standard residual, and p-value for Ljung-Box statistics of PACF relationship in Nakhon Phanom over the training data starting from January 2001 to December 2013.

For Nakhon Phanom, the best model is based on negative binomial method. The correlation coefficient on the test set in 2014 is 0.607 (95%CI: 0.5014, 0.7126). The model consists of 6 variables. The most significant variable are 1-week-lag cases and 2-week-lag wind direction, following by current week relative humidity. Other variables are, 1-week-lag, 2-week-lag and 3-week-lag precipitation. Time series methods by ARIMA and SARIMA yield the correlation coefficient of -1.044843 and -498.8625 respectively.

Table 47: Comparison table of all methods by the highest correlation coefficient (R^2) and the lowest prediction error (RMSE) in nakhonphanom.

Methods	R-squared (R^2)	Root mean square error (RMSE)
Poisson Regression	-0.5226896	4.091525
Negative Binomial Regression	0.6074892	2.077329
Quasi-likelihood Regression	0.5978702	2.102629
ARIMA (3,1,4)	-1.044843	4.741431
SARIMA $(2,0,1)(0,2,0)_{52}$	-498.8625	74.13184

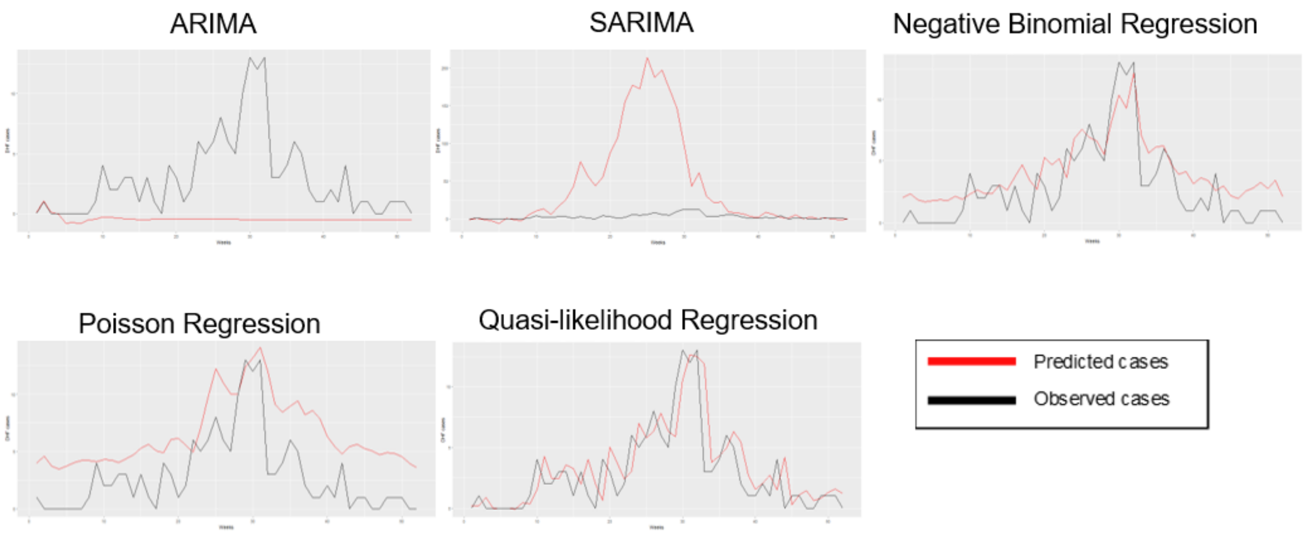

Figure 143: Plots between dengue cases and weeks, the black line represents the observed dengue cases, and the red line represents the predicted dengue cases of the best fit model of each technique over the test set data starting from January 2014 to December 2014.

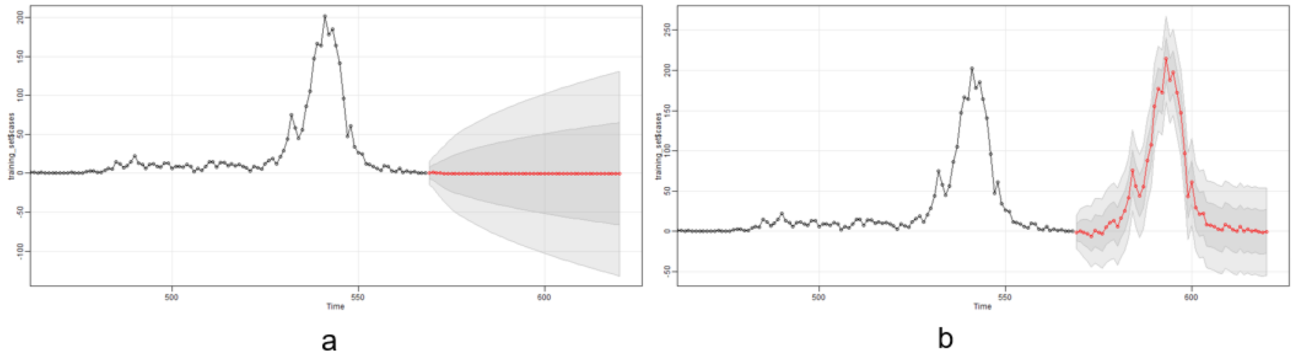

Figure 144: (a) Plot between dengue incidences over weekly time by the best model of ARIMA and (b) SARIMA time series analysis, the black line represents training set data starting from January 2012 to December 2013, and the red line represents the forecasted dengue incidences from January 2014 to December 2014.

Table 48: Coefficients and significant values of best fit GLM models, Negative Binomial, Poisson and Quasi-likelihood regression model of Nakhon Phanom. The table summarizes coefficients of each independent variables which are composed in best fit model of each method. The significant of each variable is labelled by asterisks under the coefficients. The most important factor is marked as three asterisks which p-value ranges from 0 to 0.001. The second important factor is marked as two asterisks which p-value ranges from 0.001 to 0.01. The third important factor is marked as an asterisk which p-value ranges from 0.01 to 0.1. The least important is also marked as a dot which p-value ranges from 0.1 to 1.

**Independent variables**	**Lag**	**Coefficients/Significant**		
		NB	Poisson	Quasi
Intercept		-0.7352260	-0.5101268 **	70.082608
Cases	1	0.0501751 ***	0.0198100 ***	0.943413 ***
	2			
	3			
Average Pressure	0			
	1			-0.069323
	2			
	3			
Minimum Temperature	0			
	1			
	2			
	3			
Maximum Temperature	0			
	1			
	2			
	3			
Relative Humidity	0	0.0162832 *	0.0089535 **	
	1		0.0220193 ***	
	2			
	3		-0.0005611	
Precipitation	0		0.0030784 .	
	1	-0.0032703		-0.031944
	2	0.0008901		
	3	-0.0025511		
Vaporization	0			
	1			
	2			
	3			
Wind Direction	0			
	1			
	2	0.0049184 ***		0.005497
	3			
Wind Power	0			
	1			
	2			
	3			

Nakhon Ratchasima

Nakhon Ratchasima is located in the northeastern continent of Thailand at coordinate of 14°58′50″N 102°06′00″E. Nakhon Ratchasima covers an area of 20,494 km^2. Total population are 2,620,517 people. The density of population is approximately 128.0 people per km^2. Weather in Nakhon Ratchasima has tropical savanna climate under the South Asian monsoon system. Temperature is high in April approximately 42.2 °C and starts to low temperature from December to February (2.7- 16.1°C). Winters are dry and warm. The monsoon season begins from May through October. The highest rainfall presents in August around 252.2 mm. Humidity is in range from 61-80 percent throughout the year. The highest sunshine hours are in January.

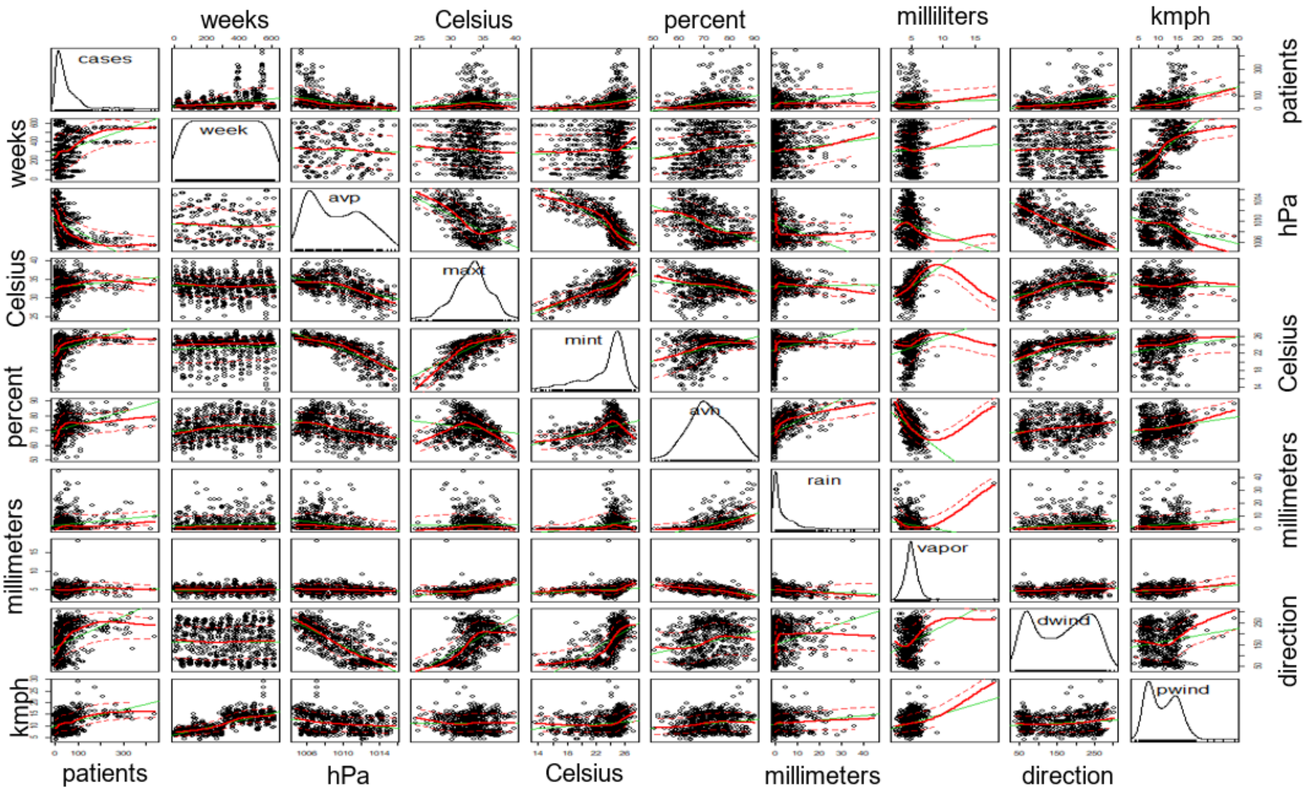

Figure 145: Scatter plot between dengue cases (cases) and selected independent variables, which are the weekly period starting from January 2001 – December 2013 (week), average pressure (avp), maximum temperature (maxt), minimum temperature (mint), average humidity (avh), precipitation (rain), vaporization of water (vapor), wind direction (dwind), and wind power (pwind). The plot visualizes pairwise hundred relationships of training set in Nakhon Ratchasima.

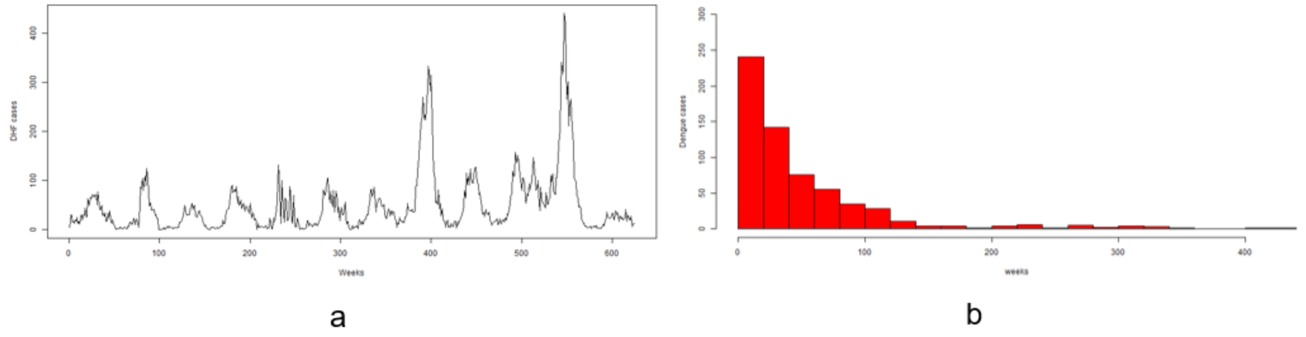

Figure 146: (a) Line plot between dengue incidences and weeks, the plot shows trends of dengue incidences in each year as stationary time series. (b) Histogram of dengue incidences in Nakhon Ratchasima starting from January 2001 to December 2013 (624 weeks).

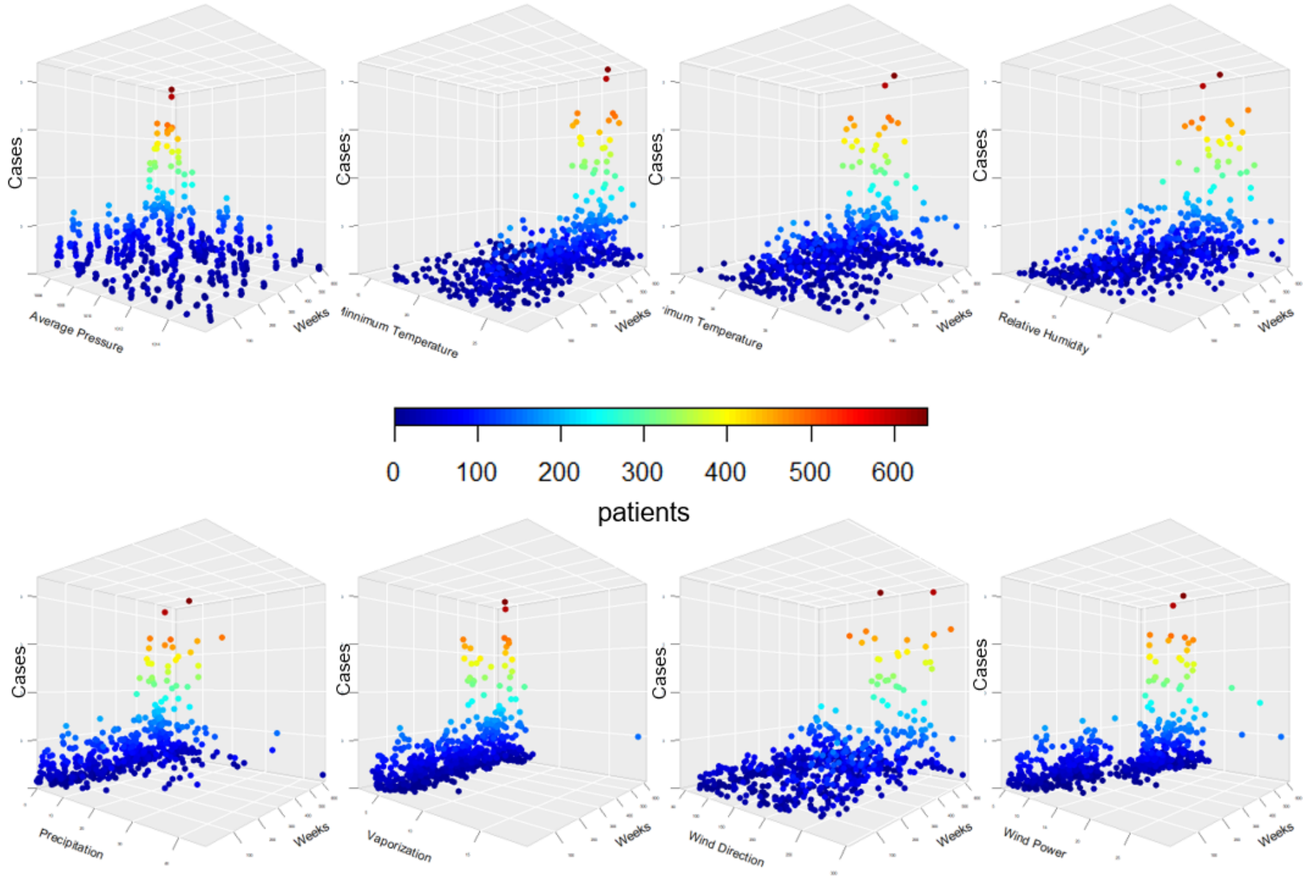

Figure 147: Three-dimensional scatter plot between dengue incidences and weather effects starting from January 2001 to December 2013 of Nakhon Ratchasima.

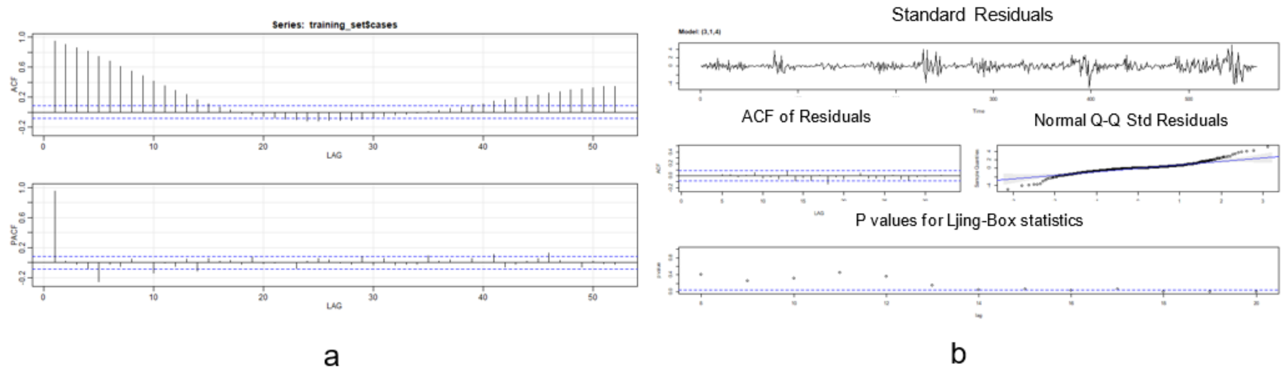

Figure 148: (a) Two plots between lag-time of dengue incidences and ACF and PACF relationship calculated from ARIMA model (b) Summary plots of time series analysis, multiple plots include the plot of predicted model over the time, the plot of ACF residual over lag-time of dengue incidences, residual Q-Q plot of standard residual, and p-value for Ljung-Box statistics of PACF relationship in Nakhon Ratchasima over the training data starting from January 2001 to December 2013.

For Nakhon Ratchasima, the best model is based on quasi-likelihood method. The correlation coefficient on the test set in 2014 is 0.30 (95%CI: 0.1961, 0.4099). The model consists of 7 variables. The most significant variables are 1-week-lag cases, 1-week-lag average pressure, following by current week relative humidity and 3-week-lag wind direction. Other variables which have less significant are, current week and 2-week-lag vaporization, 3-week-lag wind power. Time series methods by ARIMA and SARIMA yield the correlation coefficient of -1.584944 and -213.6987 respectively.

Table 49: Comparison table of all methods by the highest correlation coefficient (R^2) and the lowest prediction error (RMSE) in nakhonratchasima.

Methods	R-squared (R^2)	Root mean square error (RMSE)
Poisson Regression	-5.450173	28.50505
Negative Binomial Regression	-15.50038	45.59143
Quasi-likelihood Regression	0.3031361	9.369365
ARIMA (3,1,4)	-1.584944	18.0452
SARIMA $(2,0,1)(0,2,0)_{52}$	-213.6987	164.4564

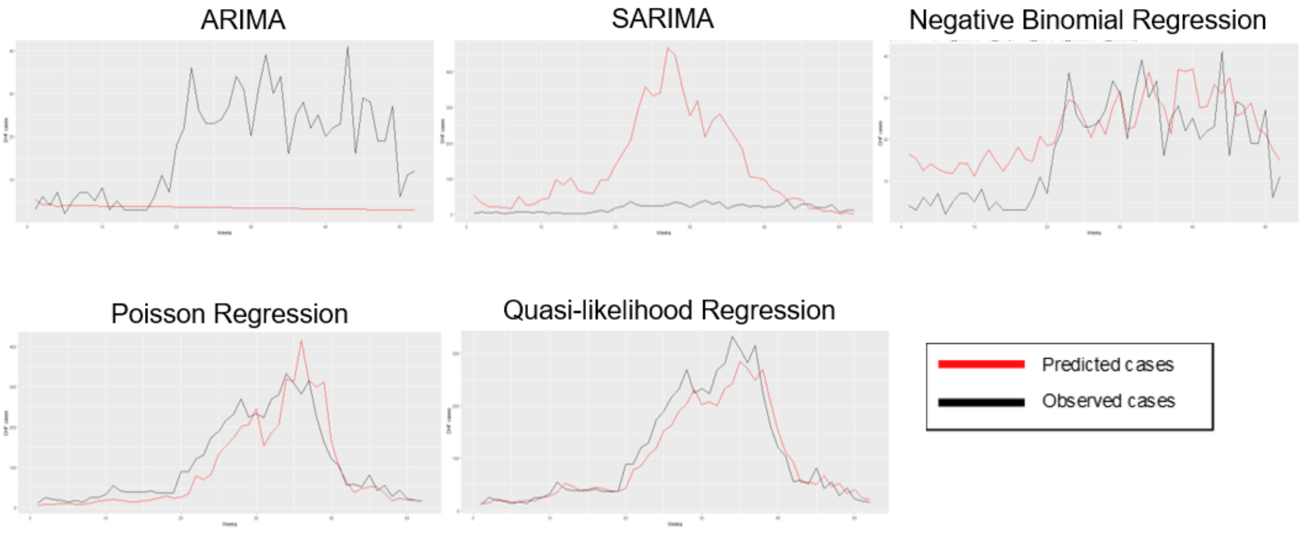

Figure 149: Plots between dengue cases and weeks, the black line represents the observed dengue cases, and the red line represents the predicted dengue cases of the best fit model of each technique over the test set data starting from January 2014 to December 2014.

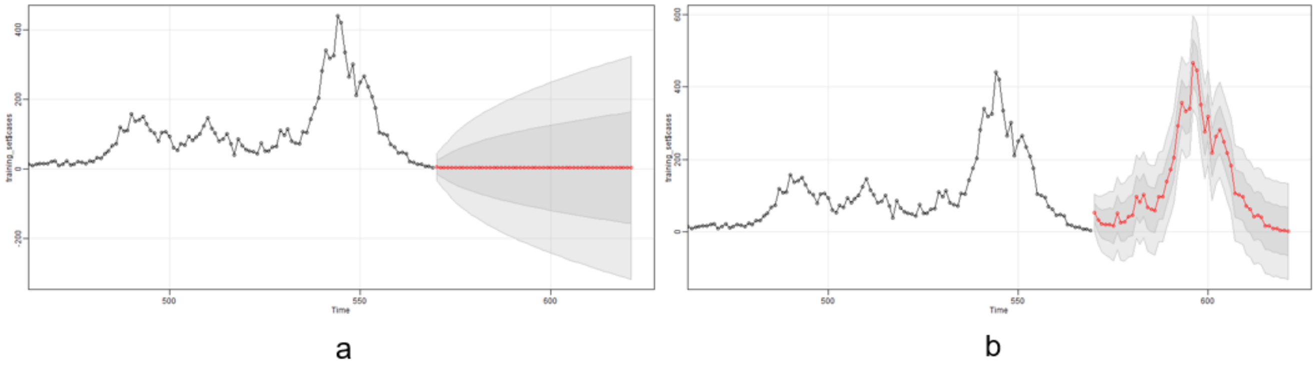

Figure 150: (a) Plot between dengue incidences over weekly time by the best model of ARIMA and (b) SARIMA time series analysis, the black line represents training set data starting from January 2012 to December 2013, and the red line represents the forecasted dengue incidences from January 2014 to December 2014.

Table 50: Coefficients and significant values of best fit GLM models, Negative Binomial, Poisson and Quasi-likelihood regression model of Nakhon Ratchasima. The table summarizes coefficients of each independent variables which are composed in best fit model of each method. The significant of each variable is labelled by asterisks under the coefficients. The most important factor is marked as three asterisks which p-value ranges from 0 to 0.001. The second important factor is marked as two asterisks which p-value ranges from 0.001 to 0.01. The third important factor is marked as an asterisk which p-value ranges from 0.01 to 0.1. The least important is also marked as a dot which p-value ranges from 0.1 to 1.

**Independent variables**	**Lag**	**Coefficients/Significant**		
		NB	Poisson	Quasi
Intercept		-3.6859271 ***	96.6719462 ***	2480.51195 ***
Cases	1			0.92341 ***
	2			
	3		0.0054179 ***	
Average Pressure	0		-0.0662334 ***	
	1		-0.0298608 **	-2.42209 ***
	2			
	3			
Minimum Temperature	0			
	1			
	2			
	3	0.2438295 ***	0.2057537 ***	
Maximum Temperature	0			
	1			
	2	0.0358294 .		
	3	-0.0768761 *	-0.0460262 ***	
Relative Humidity	0			-0.38722 *
	1			
	2			
	3	0.0287379 ***		
Precipitation	0			
	1			
	2			
	3			
Vaporization	0	0.0350905		-0.39132
	1			
	2			-0.25958
	3			
Wind Direction	0	0.0025335 ***		
	1	0.0023200 **	-0.0004695 **	
	2			
	3	-0.0003225		-0.03075 .
Wind Power	0			
	1			
	2			
	3		0.0319711 ***	0.34821

Nakhon Sawan

Nakhon Sawan is located in the central of Thailand at coordinate of 15°42′48″N 100°08′07″E. Nakhon Sawan covers an area of 9,598 km^2. Total population are 1,072,756 people. The density of population is 112.0 people per km^2. Weather in Nakhon Sawan follows tropical savanna climate system. The highest temperature is in April approximately 42.4°C. The low temperature presents in winter from December to February approximately 4.5-11.9°C. The monsoon season starts from May through October. September is the wettest months (237.0 mm). The highest sunshine hours are in January. Humidity presents around 62-83 percent throughout the year

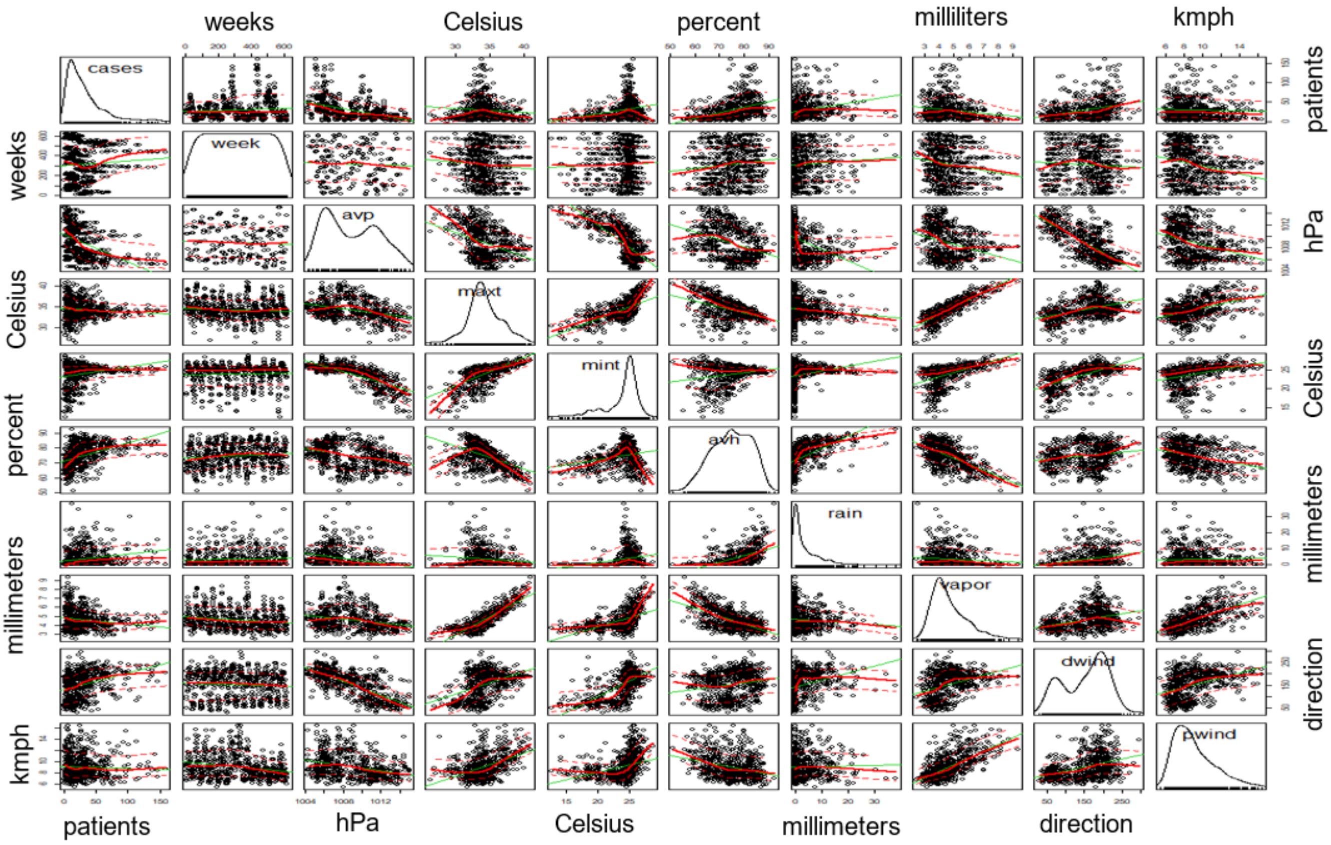

Figure 151: Scatter plot between dengue cases (cases) and selected independent variables, which are the weekly period starting from January 2001 – December 2013 (week), average pressure (avp), maximum temperature (maxt), minimum temperature (mint), average humidity (avh), precipitation (rain), vaporization of water (vapor), wind direction (dwind), and wind power (pwind). The plot visualizes pairwise hundred relationships of training set in Nakhon Sawan.

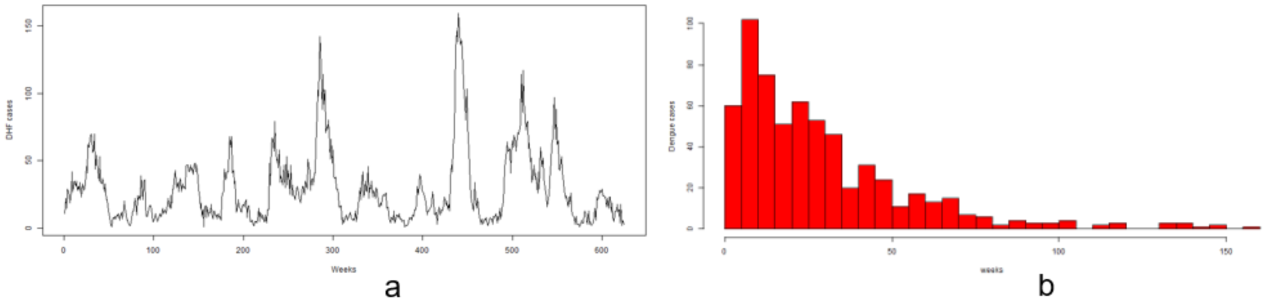

Figure 152: (a) Line plot between dengue incidences and weeks, the plot shows trends of dengue incidences in each year as stationary time series. (b) Histogram of dengue incidences in Nakhon Sawan starting from January 2001 to December 2013 (624 weeks).

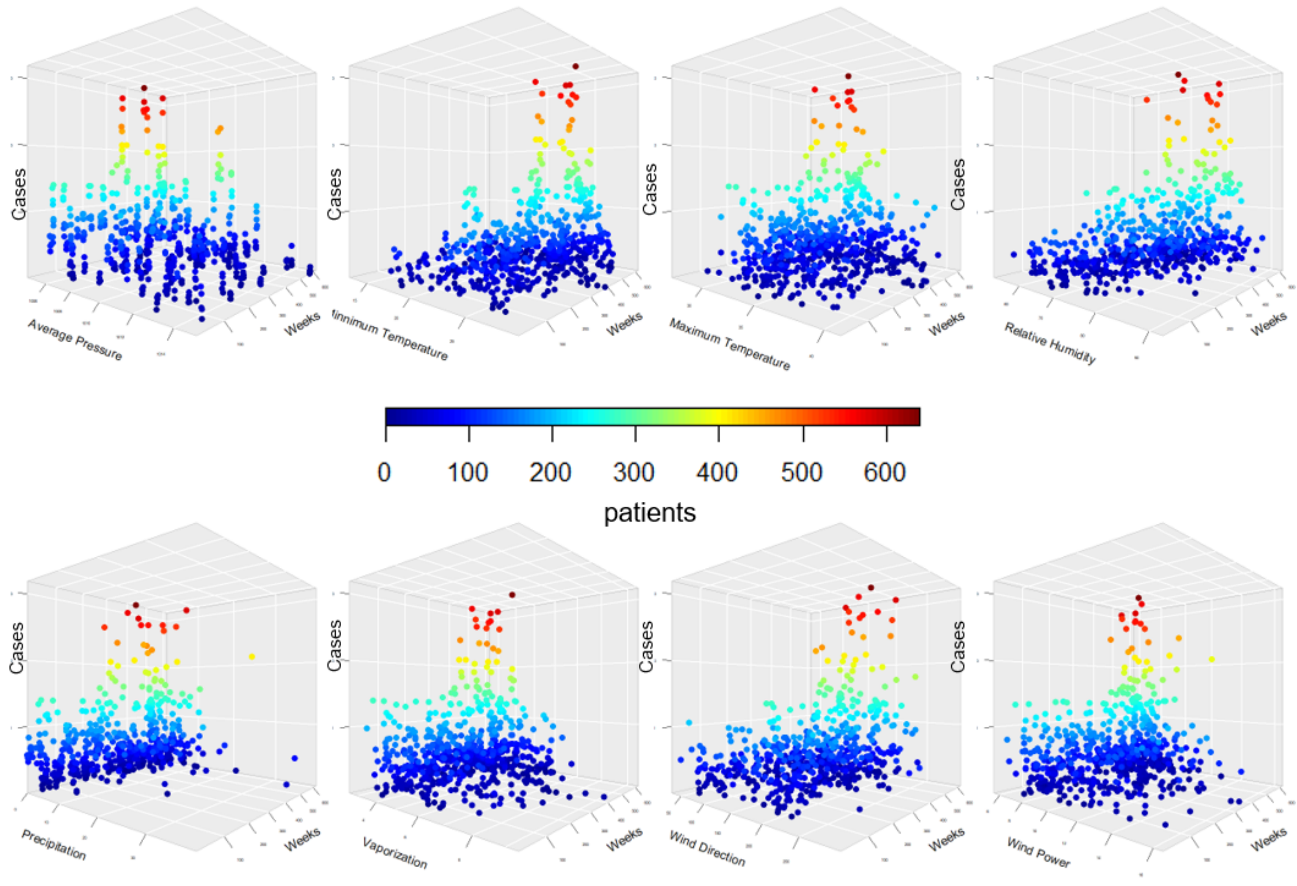

Figure 153: Three-dimensional scatter plot between dengue incidences and weather effects starting from January 2001 to December 2013 of Nakhon Sawan.

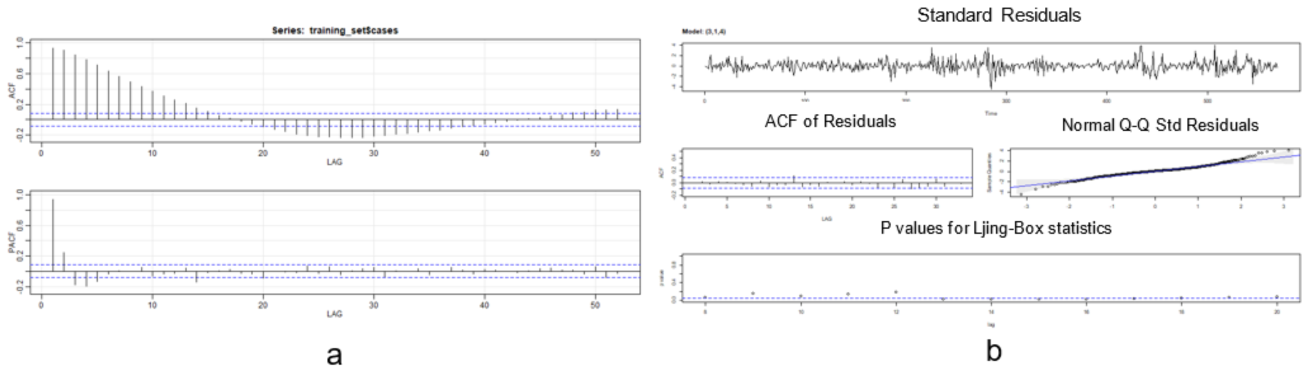

Figure 154: (a) Two plots between lag-time of dengue incidences and ACF and PACF relationship calculated from ARIMA model (b) Summary plots of time series analysis, multiple plots include the plot of predicted model over the time, the plot of ACF residual over lag-time of dengue incidences, residual Q-Q plot of standard residual, and p-value for Ljung-Box statistics of PACF relationship in Nakhon Sawan over the training data starting from January 2001 to December 2013.

The best model of Nakhon Sawan is based on quasi-likelihood method. The correlation coefficient on the test set in 2014 is 0.614 (95%CI: 0.5138, 0.7142). The best model uses 9 variables. The most significant variables are 1-week-lag cases and 2-week-lag cases. Other variables are, 3-week-lag maximum temperature, current week relative humidity, 2-week-lag precipitation, 3-week-lag vaporization, 3-week-lag wind direction, current week and 3-week-lag wind power. Time series methods by ARIMA and SARIMA yield the correlation coefficient of -1.361922 and -3.139921 respectively.

Table 51: Comparison table of all methods by the highest correlation coefficient (R^2) and the lowest prediction error (RMSE) in Nakhon Sawan.

**Methods**	**R-squared (R^2)**	**Root mean square error (RMSE)**
Poisson Regression	0.2196838	7.48201
Negative Binomial Regression	0.5416973	5.73402
Quasi-likelihood Regression	0.6136943	5.264396
ARIMA (3,1,4)	-1.361922	13.01714
SARIMA $(2,0,1)(0,2,0)_{52}$	-3.139921	17.23372

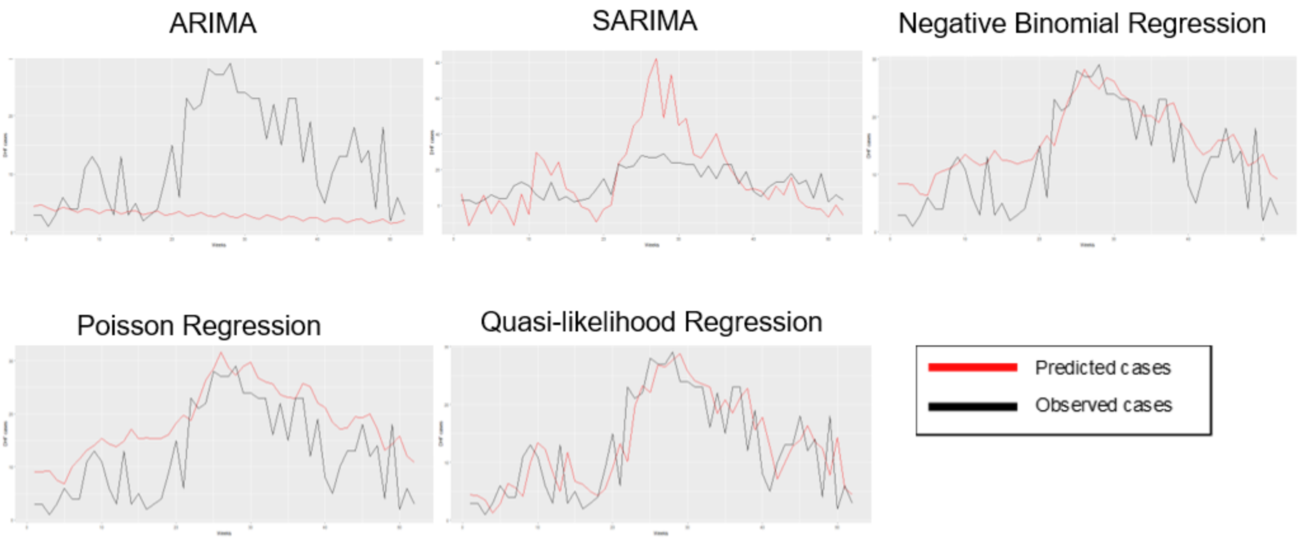

Figure 155: Plots between dengue cases and weeks, the black line represents the observed dengue cases, and the red line represents the predicted dengue cases of the best fit model of each technique over the test set data starting from January 2014 to December 2014.

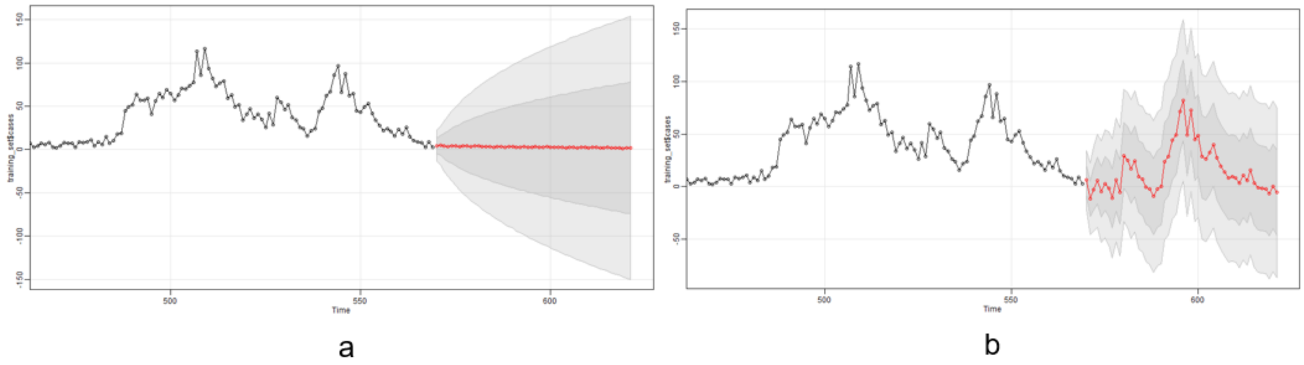

Figure 156: (a) Plot between dengue incidences over weekly time by the best model of ARIMA and (b) SARIMA time series analysis, the black line represents training set data starting from January 2012 to December 2013, and the red line represents the forecasted dengue incidences from January 2014 to December 2014.

Table 52: Coefficients and significant values of best fit GLM models, Negative Binomial, Poisson and Quasi-likelihood regression model of Nakhon Sawan. The table summarizes coefficients of each independent variables which are composed in best fit model of each method. The significant of each variable is labelled by asterisks under the coefficients. The most important factor is marked as three asterisks which p-value ranges from 0 to 0.001. The second important factor is marked as two asterisks which p-value ranges from 0.001 to 0.01. The third important factor is marked as an asterisk which p-value ranges from 0.01 to 0.1. The least important is also marked as a dot which p-value ranges from 0.1 to 1.

**Independent variables**	**Lag**	**Coefficients/Significant**		
		NB	Poisson	Quasi
Intercept		36.2992398 **	29.9246817 ***	-1.1016498
Cases	1	0.0155547 ***	0.0120132 ***	0.6986384 ***
	2	0.0071570 ***	0.0044656 ***	0.2485593 ***
	3			
Average Pressure	0	-0.0329201 **	-0.0265545 ***	
	1			
	2			
	3			
Minimum Temperature	0			
	1	0.0697755 ***	0.0750744 ***	
	2		0.0182944 *	
	3			
Maximum Temperature	0			
	1	-0.0327112 *	-0.0396276 ***	
	2	-0.0328753 **	-0.0376005 ***	
	3			-0.2380460
Relative Humidity	0			0.0748130
	1			
	2			
	3			
Precipitation	0			
	1			
	2	-0.0023996	-0.0014539	0.0350461
	3			
Vaporization	0			
	1			
	2			
	3			0.9869123
Wind Direction	0		-0.0001021	
	1		0.0003019	
	2		-0.0002209	
	3	-0.0001913		-0.0004154
Wind Power	0			0.2118136
	1			
	2			
	3			-0.1419392

Nakhon Si Thammarat

Nakhon Si Thammarat is located in southern region Thailand at 8°26′11″N 99°57′47″E. Nakhon Si Thammarat covers an area of 9,943 km^2. Total population are 1,544,028 people. The density of population is 155.0 people per km^2. Weather in Nakhon Si Thammarat has tropical rainforest climate system. Temperature in Nakhon Si Thammarat has the highest of 38.9 °C in April and the lowest of 18.0 °C in January. Nakhon Si Thammarat has a dry season that runs from December through March and a wet season. Precipitation occurs from mid-May to August. Rainfall are roughly 631.2 mm in November. Humidity presents in the range of 79-87 percent throughout the year. March is the highest sunshine hours

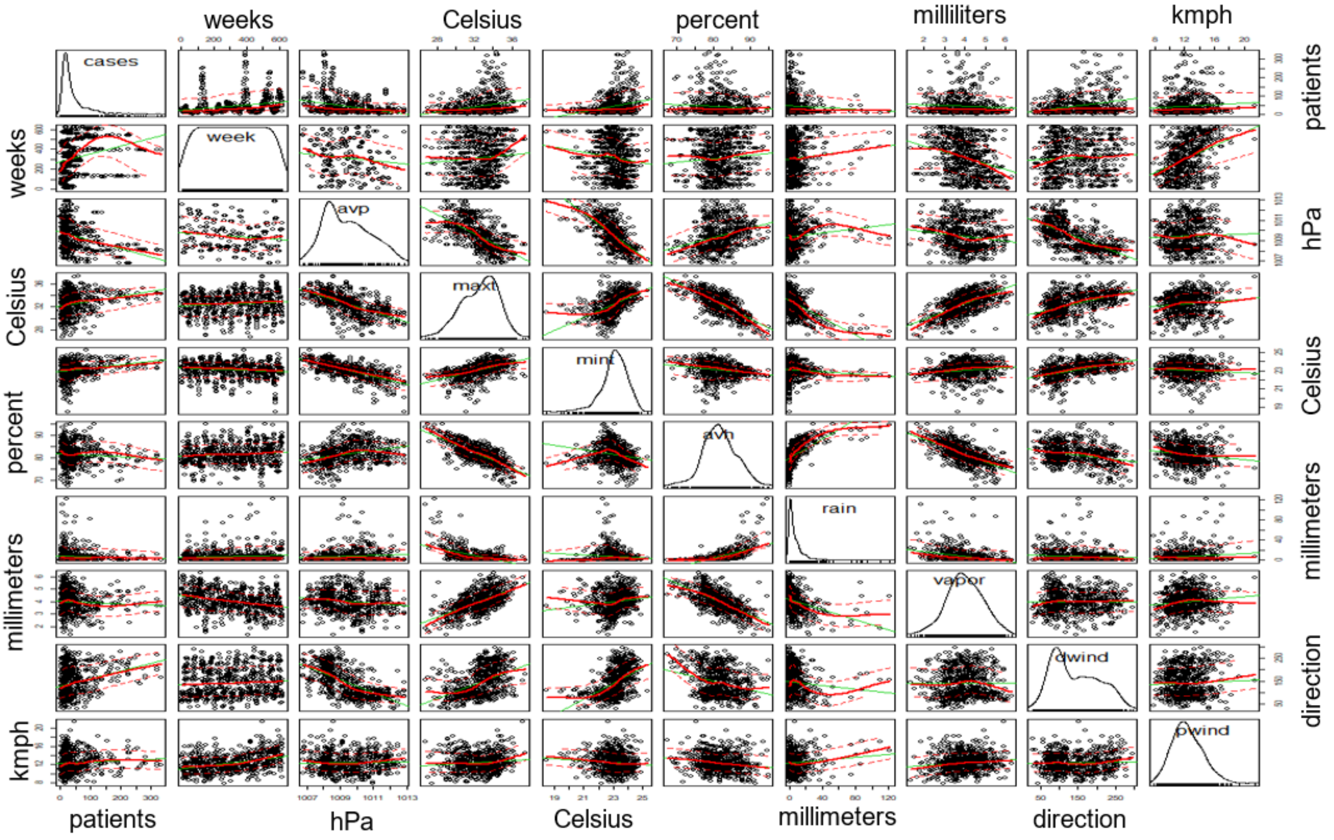

Figure 157: Scatter plot between dengue cases (cases) and selected independent variables, which are the weekly period starting from January 2001 – December 2013 (week), average pressure (avp), maximum temperature (maxt), minimum temperature (mint), average humidity (avh), precipitation (rain), vaporization of water (vapor), wind direction (dwind), and wind power (pwind). The plot visualizes pairwise hundred relationships of training set in Nakhon Si Thammarat.

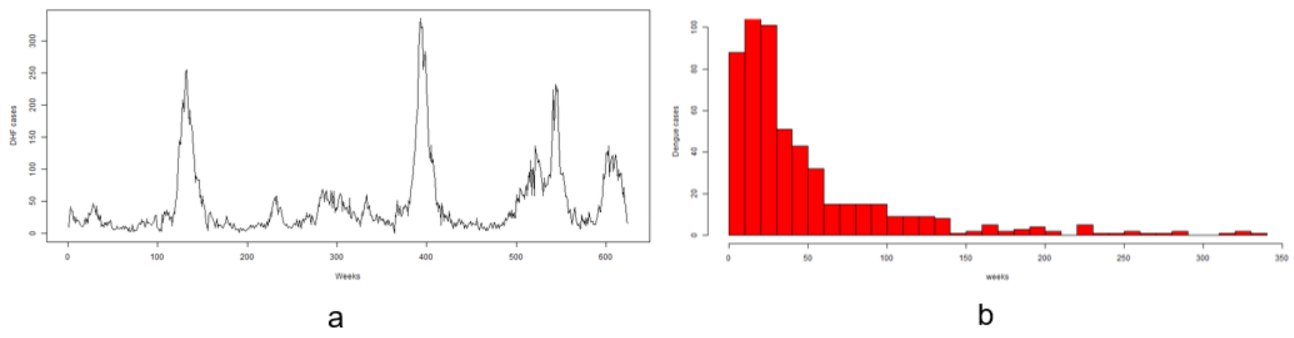

Figure 158: (a) Line plot between dengue incidences and weeks, the plot shows trends of dengue incidences in each year as stationary time series. (b) Histogram of dengue incidences in Nakhon Si Thammarat starting from January 2001 to December 2013 (624 weeks).

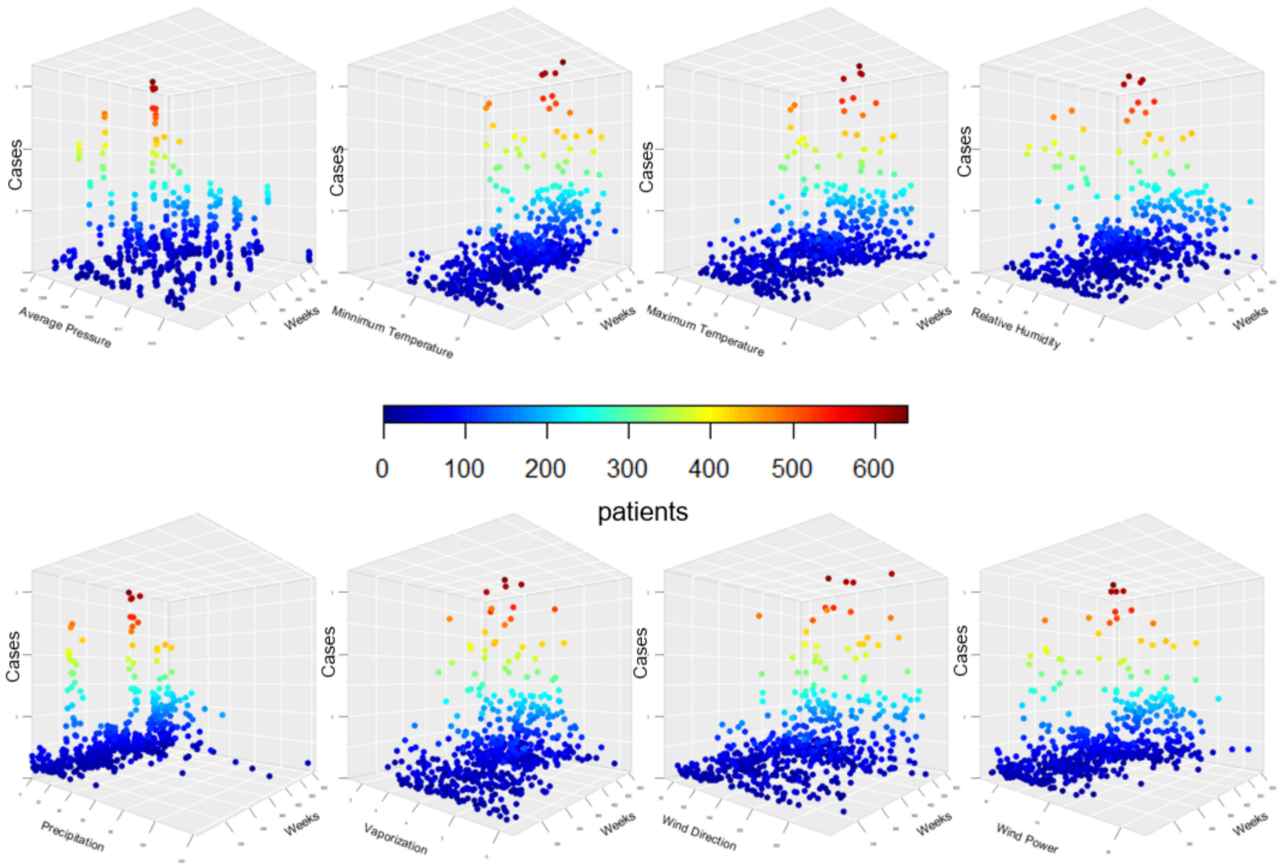

Figure 159: Three-dimensional scatter plot between dengue incidences and weather effects starting from January 2001 to December 2013 of Nakhon Si Thammarat.

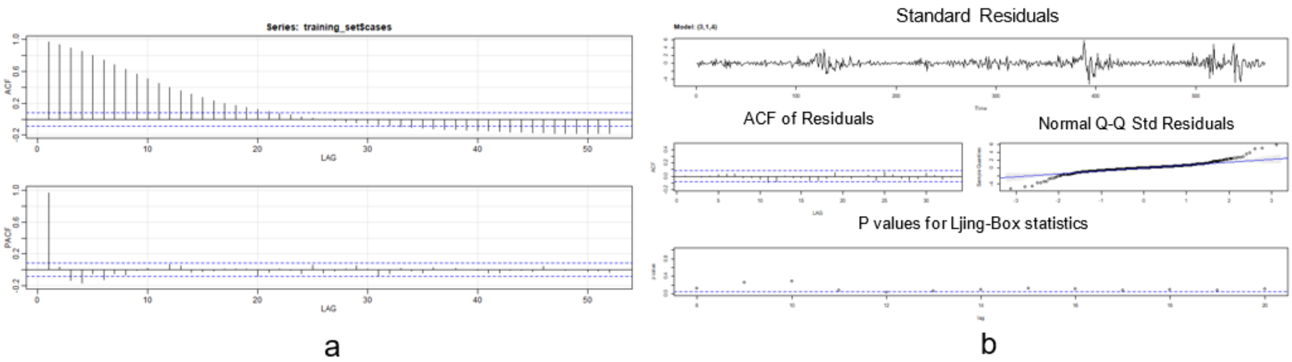

Figure 160: (a) Two plots between lag-time of dengue incidences and ACF and PACF relationship calculated from ARIMA model (b) Summary plots of time series analysis, multiple plots include the plot of predicted model over the time, the plot of ACF residual over lag-time of dengue incidences, residual Q-Q plot of standard residual, and p-value for Ljung-Box statistics of PACF relationship in Nakhon Si Thammarat over the training data starting from January 2001 to December 2013.

For Nakhon Si Thammarat, the best model is based on quasi-likelihood method. The correlation coefficient on the test set in 2014 is 0.884 (95%CI: 0.8363, 0.9317). The significant of the variables associated with p-value statistical calculation are shown in 53. The best model of Nakhon Si Thammarat uses 8 variables. The most significant variables are 1-week-lag cases, following by 3-week-lag precipitation, 2-week-lag cases, current week minimum temperature and 2-week-lag maximum temperature. Other variables which have less significant are, current week precipitation, wind direction and wind power. Time series methods by ARIMA and SARIMA yield the correlation coefficient of -2.024591 and -2.315421 respectively.

Table 53: Comparison table of all methods by the highest correlation coefficient (R^2) and the lowest prediction error (RMSE) in nakhonsithammarat.

Methods	R-squared (R^2)	Root mean square error (RMSE)
Poisson Regression	0.7044812	22.18263
Negative Binomial Regression	0.7896384	18.71559
Quasi-likelihood Regression	0.884229	13.88418
ARIMA (3,1,4)	-2.024591	70.96656
SARIMA $(2,0,1)(0,2,0)_{52}$	-2.315421	74.30017

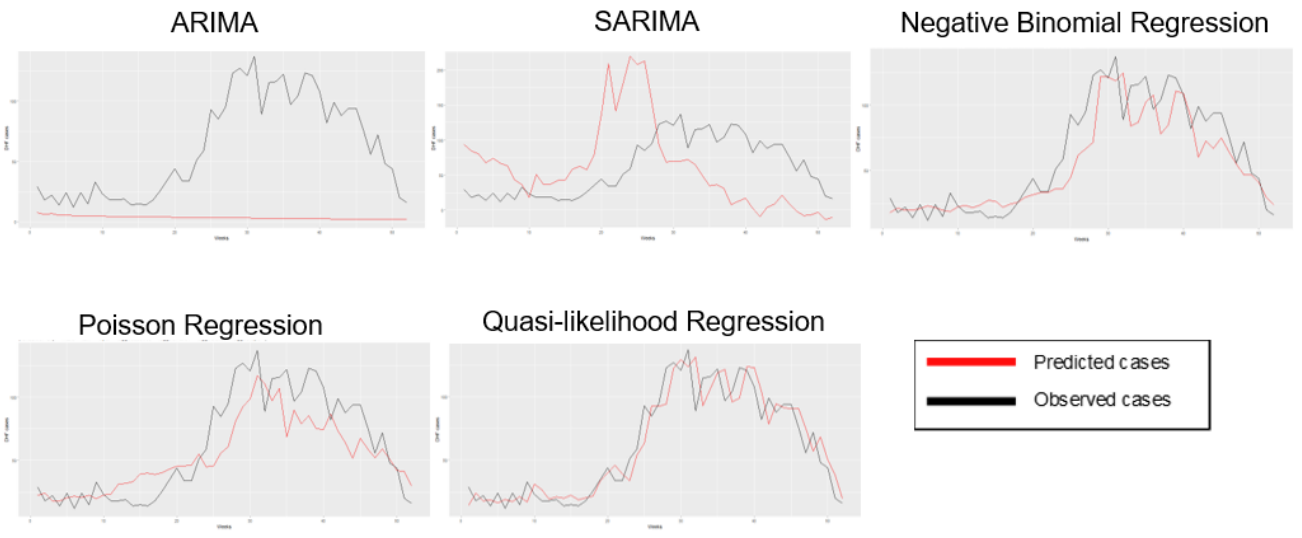

Figure 161: Plots between dengue cases and weeks, the black line represents the observed dengue cases, and the red line represents the predicted dengue cases of the best fit model of each technique over the test set data starting from January 2014 to December 2014.

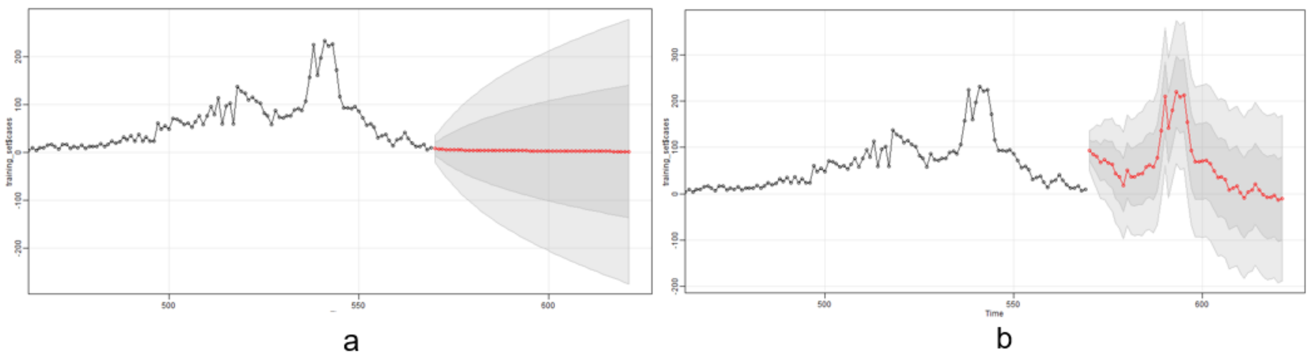

Figure 162: (a) Plot between dengue incidences over weekly time by the best model of ARIMA and (b) SARIMA time series analysis, the black line represents training set data starting from January 2012 to December 2013, and the red line represents the forecasted dengue incidences from January 2014 to December 2014.

Table 54: Coefficients and significant values of best fit GLM models, Negative Binomial, Poisson and Quasi-likelihood regression model of Nakhon Si Thammarat. The table summarizes coefficients of each independent variables which are composed in best fit model of each method. The significant of each variable is labelled by asterisks under the coefficients. The most important factor is marked as three asterisks which p-value ranges from 0 to 0.001. The second important factor is marked as two asterisks which p-value ranges from 0.001 to 0.01. The third important factor is marked as an asterisk which p-value ranges from 0.01 to 0.1. The least important is also marked as a dot which p-value ranges from 0.1 to 1.

**Independent variables**	**Lag**	**Coefficients/Significant**		
		NB	Poisson	Quasi
Intercept		-63.375993 *	-56.32 ***	-412.07945
Cases	1	0.010394 ***		0.91796 ***
	2	0.003951 **		0.16238 **
	3		0.008703 ***	-0.12406 **
Average Pressure	0		0.05230 ***	2.30246
	1			-3.41559 .
	2	0.062052 **		1.47238
	3			
Minimum Temperature	0			
	1			
	2			
	3			
Maximum Temperature	0	0.128518 ***	0.1298 ***	1.90528 **
	1		0.09985 ***	
	2			
	3			
Relative Humidity	0			
	1			
	2			
	3			
Precipitation	0	0.005322 *	0.006612 ***	
	1		0.005271 ***	
	2			
	3			
Vaporization	0	-0.145851 ***	-0.1025 ***	-2.04998 *
	1		-0.1190 ***	
	2			
	3	-0.028642		
Wind Direction	0			
	1			-0.01668
	2			
	3			
Wind Power	0			
	1			
	2			
	3			

Nan

Nan is located in the northern region of Thailand at coordinate of 18°47′N 100°47′E. Nan covers an area of 11,472 km^2. Total population are 478,264 people. The density of population is 42.0 people per km^2. Weather in Nan has tropical savanna climate under the South Asian monsoon system Temperature is in the range from the low of 2.7°C in January to the high of 43.0°C in April. The monsoon season runs with the arrival of the southwest monsoon from late-April through early October. The humidity presents the average of 65-84 percent. Precipitation starts to rise from mid-May to August. The highest precipitation is in August of 273.2 mm. The longest sunshine hours are in March.

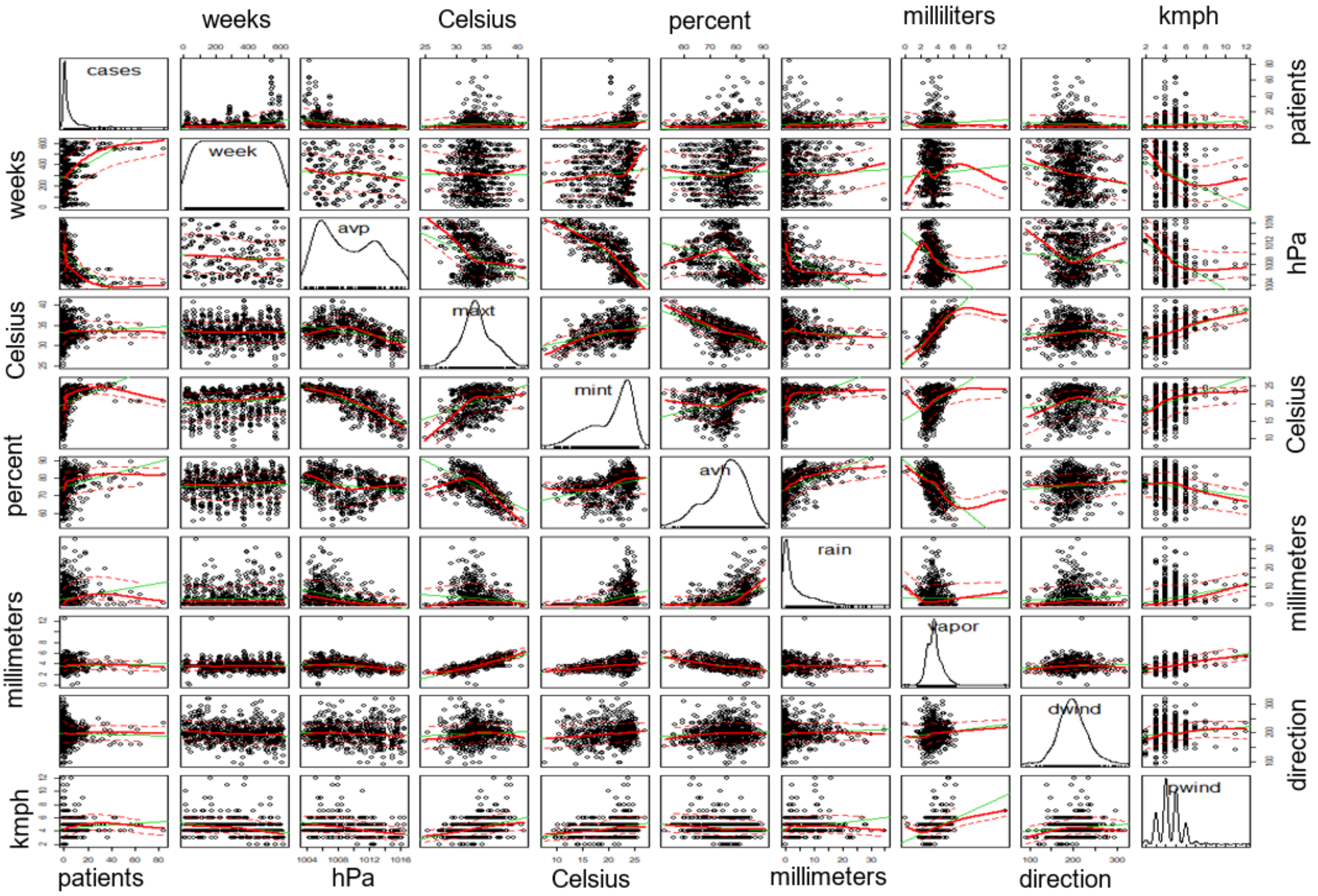

Figure 163: Scatter plot between dengue cases (cases) and selected independent variables, which are the weekly period starting from January 2001 – December 2013 (week), average pressure (avp), maximum temperature (maxt), minimum temperature (mint), average humidity (avh), precipitation (rain), vaporization of water (vapor), wind direction (dwind), and wind power (pwind). The plot visualizes pairwise hundred relationships of training set in Nan.

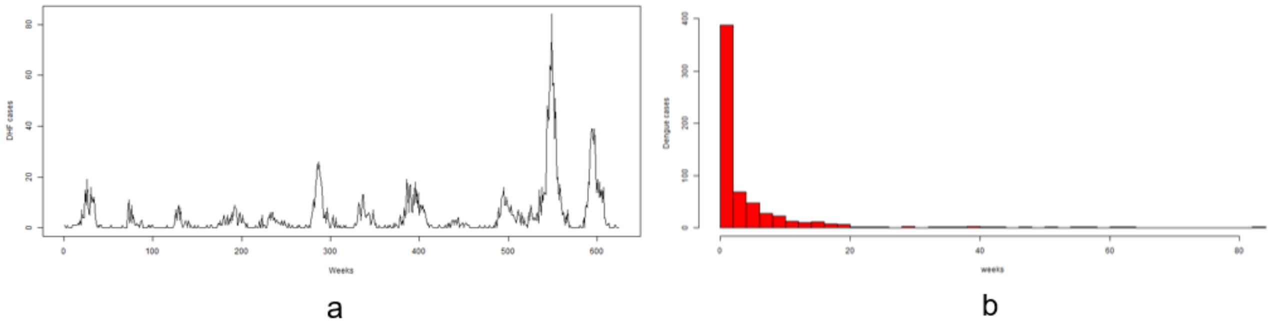

Figure 164: (a) Line plot between dengue incidences and weeks, the plot shows trends of dengue incidences in each year as stationary time series. (b) Histogram of dengue incidences in Nan starting from January 2001 to December 2013 (624 weeks).

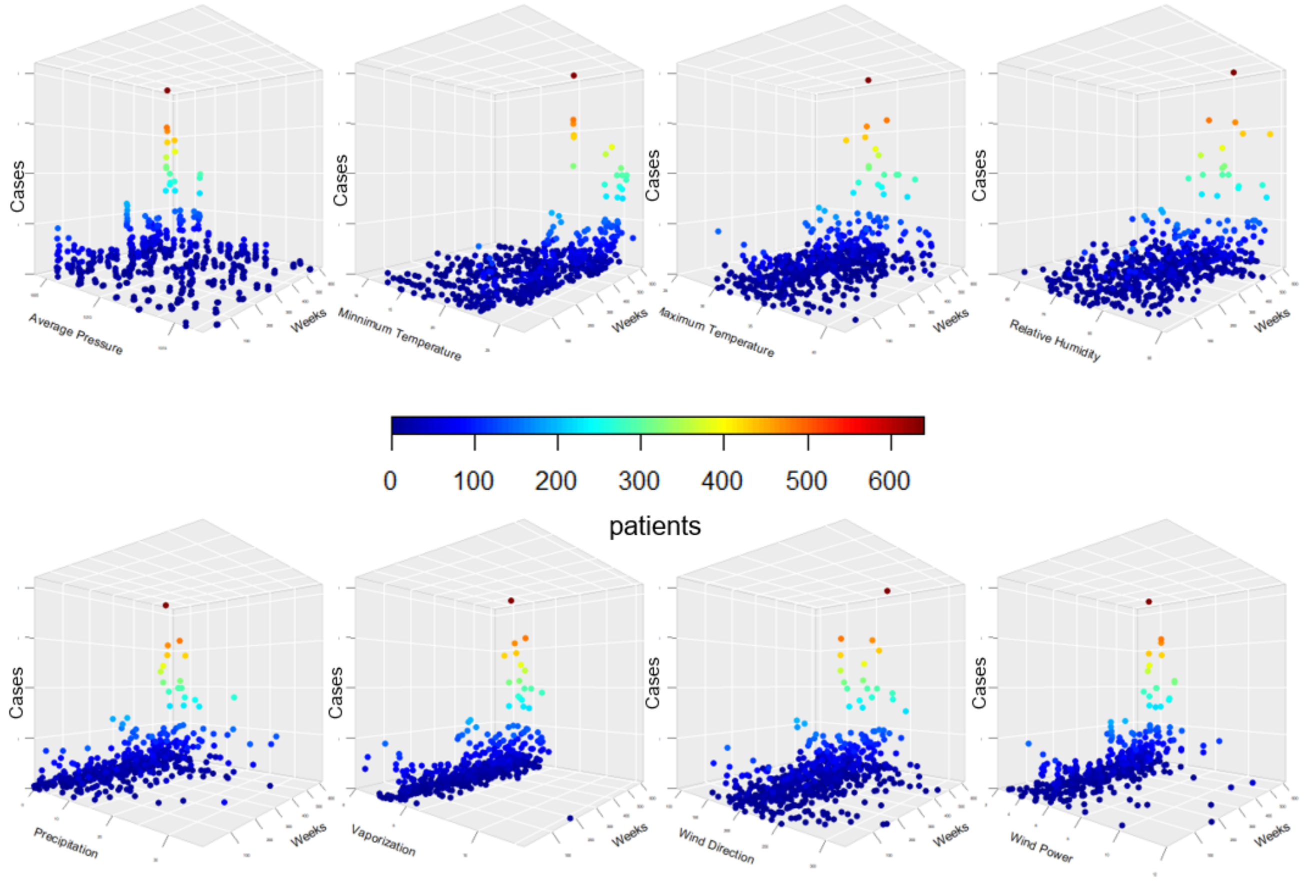

Figure 165: Three-dimensional scatter plot between dengue incidences and weather effects starting from January 2001 to December 2013 of Nan.

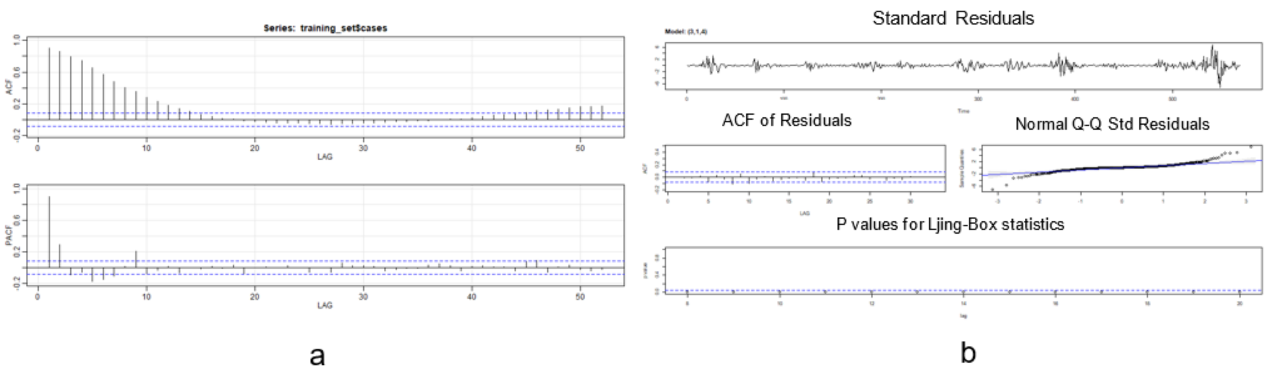

Figure 166: (a) Two plots between lag-time of dengue incidences and ACF and PACF relationship calculated from ARIMA model (b) Summary plots of time series analysis, multiple plots include the plot of predicted model over the time, the plot of ACF residual over lag-time of dengue incidences, residual Q-Q plot of standard residual, and p-value for Ljung-Box statistics of PACF relationship in Nan over the training data starting from January 2001 to December 2013.

For Nan, the best model is based on quasi-likelihood method. The correlation coefficient on the test set in 2014 is 0.863 (95%CI: 0.8138, 0.9122). The significant of the variables associated with p-value statistical calculation are shown in 55. The model uses 8 variables. The most significant variable are 1-week-lag cases, 2-week-lag cases, following by 1-week-lag vaporization and current week precipitation. Other variables which has less significant are, 3-week-lag cases, 3-week-lag relative humidity, 2-week-lag precipitation, 2-week-lag vaporization. Time series methods by ARIMA and SARIMA yield the correlation coefficient of -0.7170207 and -1.713051 respectively.

Table 55: Comparison table of all methods by the highest correlation coefficient (R^2) and the lowest prediction error (RMSE) in nan.

**Methods**	**R-squared (R^2)**	**Root mean square error (RMSE)**
Poisson Regression	0.8408704	4.885419
Negative Binomial Regression	0.3979536	9.502575
Quasi-likelihood Regression	0.8633953	4.526466
ARIMA (3,1,4)	-0.7170207	16.04775
SARIMA $(2,0,1)(0,2,0)_{52}$	-1.713051	20.1723

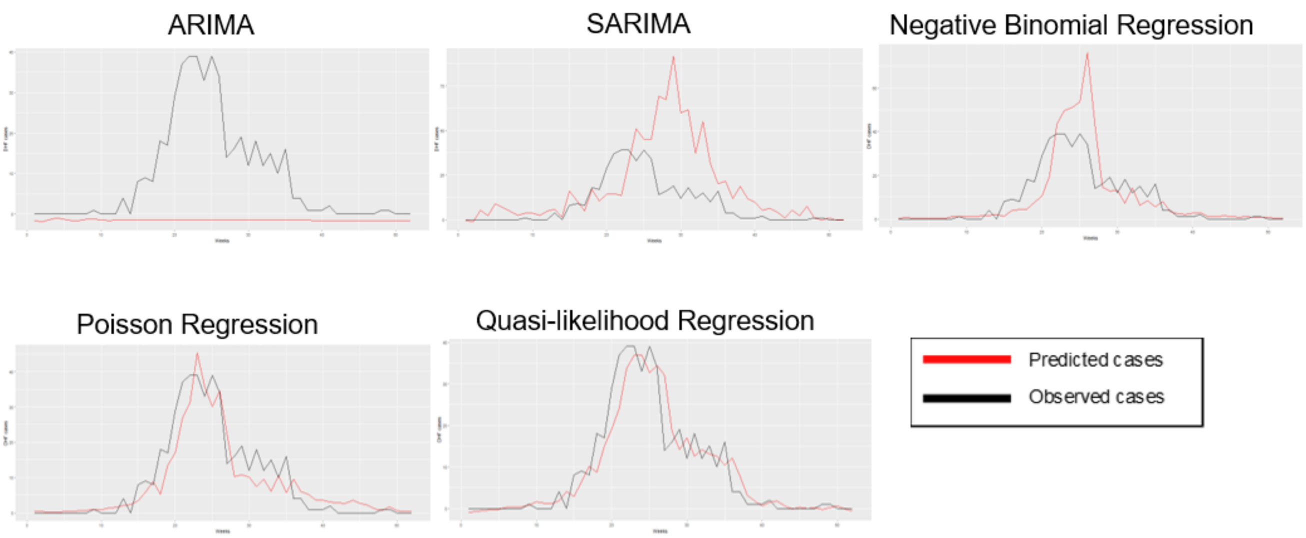

Figure 167: Plots between dengue cases and weeks, the black line represents the observed dengue cases, and the red line represents the predicted dengue cases of the best fit model of each technique over the test set data starting from January 2014 to December 2014.

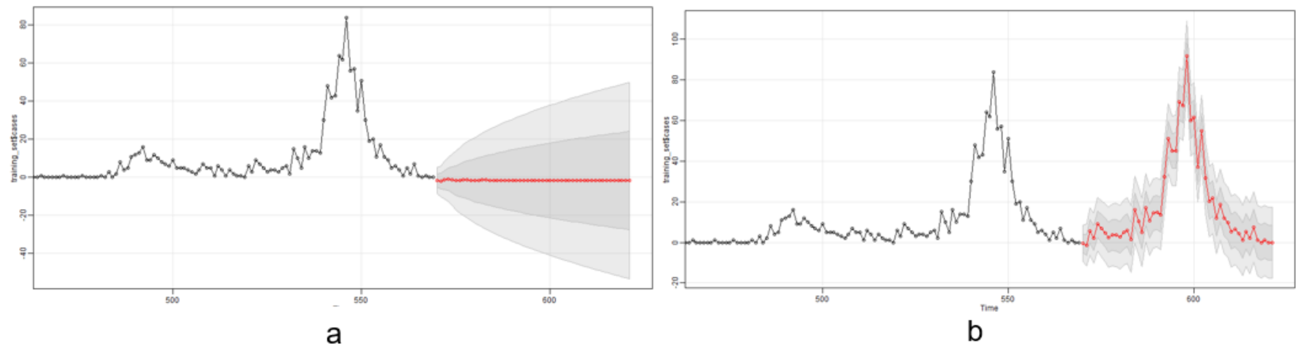

Figure 168: (a) Plot between dengue incidences over weekly time by the best model of ARIMA and (b) SARIMA time series analysis, the black line represents training set data starting from January 2012 to December 2013, and the red line represents the forecasted dengue incidences from January 2014 to December 2014.

Table 56: Coefficients and significant values of best fit GLM models, Negative Binomial, Poisson and Quasi-likelihood regression model of Nan. The table summarizes coefficients of each independent variables which are composed in best fit model of each method. The significant of each variable is labelled by asterisks under the coefficients. The most important factor is marked as three asterisks which p-value ranges from 0 to 0.001. The second important factor is marked as two asterisks which p-value ranges from 0.001 to 0.01. The third important factor is marked as an asterisk which p-value ranges from 0.01 to 0.1. The least important is also marked as a dot which p-value ranges from 0.1 to 1.

**Independent variables**	**Lag**	**Coefficients/Significant**		
		NB	Poisson	Quasi
Intercept		185.687698 ***	-9.029220 ***	-0.31152
Cases	1	0.074164 ***	0.047545 ***	0.62750 ***
	2			0.36197 ***
	3		0.005830 *	-0.06184
Average Pressure	0			
	1			
	2	-0.185388 ***		
	3			
Minimum Temperature	0		0.178470 ***	
	1			
	2			
	3			
Maximum Temperature	0		0.053238 ***	
	1			
	2			
	3			
Relative Humidity	0			
	1	0.023118 **	0.040202 ***	
	2			
	3			-0.02592
Precipitation	0		-0.006014	-0.04884 .
	1			
	2			0.04258
	3			
Vaporization	0			
	1	0.306678 ***	0.208863 ***	0.47474 *
	2			0.24848
	3		0.091281 ***	
Wind Direction	0			
	1	-0.004093 **		
	2			
	3			
Wind Power	0	-0.064704		
	1			
	2			
	3			

Narathiwat

Narathiwat is located in southern region of Thailand at 6°51′59″ N 101°15′03″ E. Narathiwat covers an area of 4,475 km^2. Total population are 775,799 people. The density of population is 173.0 people per km^2. Weather in Narathiwat has tropical monsoon climate system. Temperature in Narathiwat presents the highest of 39°C in May and the lowest of 18.8°C in March. Narathiwat has a dry season that runs from December through March and a wet season that covers the other eight months. The rainy season begins with the arrival of the southwest monsoon around mid-May. Precipitation occurs from mid-May to August. Rainfall are roughly 562.5 mm in December. Humidity presents in the range of 81-87 percent throughout the year. March is the highest sunshine hours

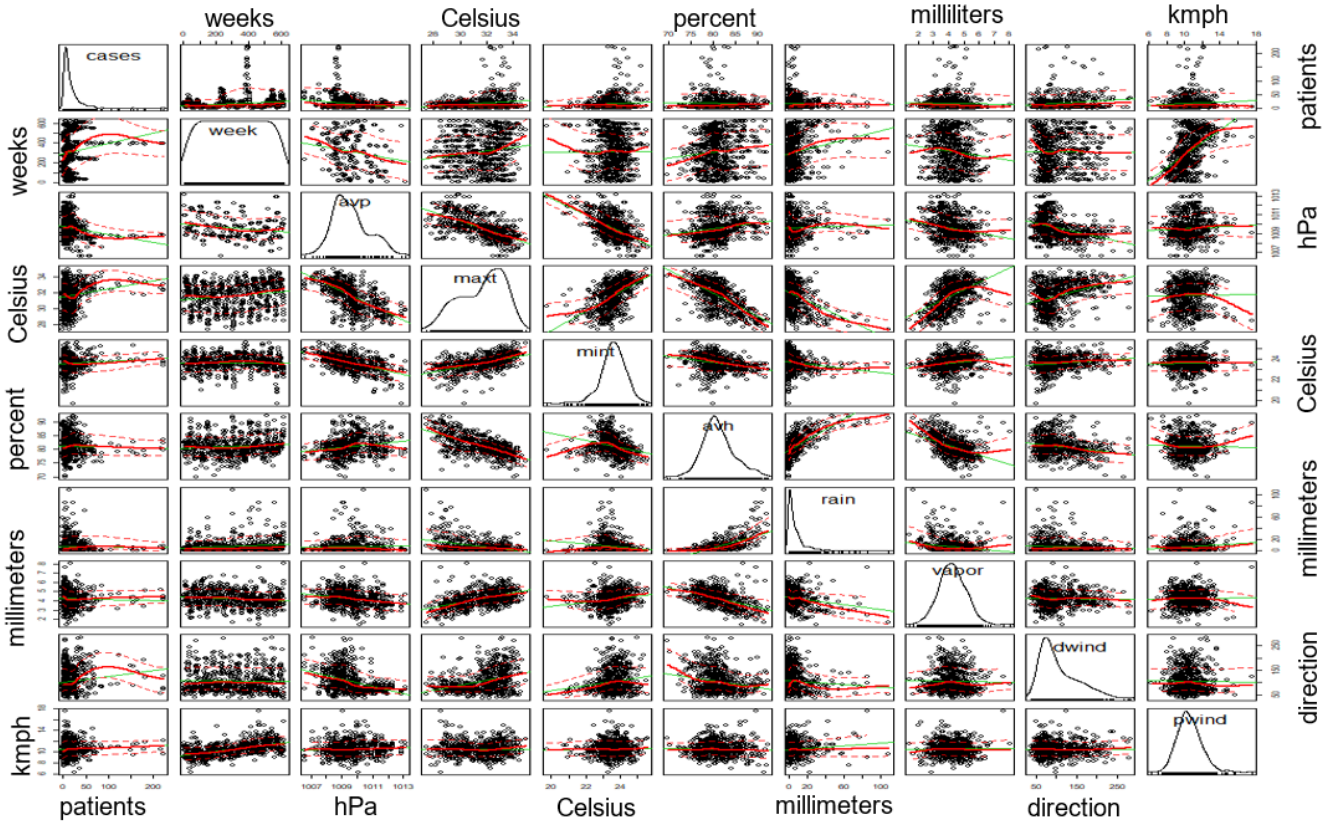

Figure 169: Scatter plot between dengue cases (cases) and selected independent variables, which are the weekly period starting from January 2001 – December 2013 (week), average pressure (avp), maximum temperature (maxt), minimum temperature (mint), average humidity (avh), precipitation (rain), vaporization of water (vapor), wind direction (dwind), and wind power (pwind). The plot visualizes pairwise hundred relationships of training set in Narathiwat.

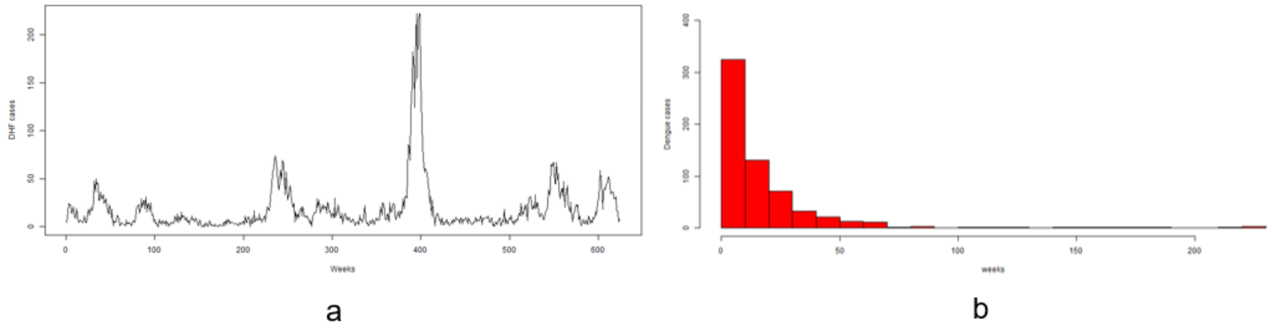

Figure 170: (a) Line plot between dengue incidences and weeks, the plot shows trends of dengue incidences in each year as stationary time series. (b) Histogram of dengue incidences in Narathiwat starting from January 2001 to December 2013 (624 weeks).

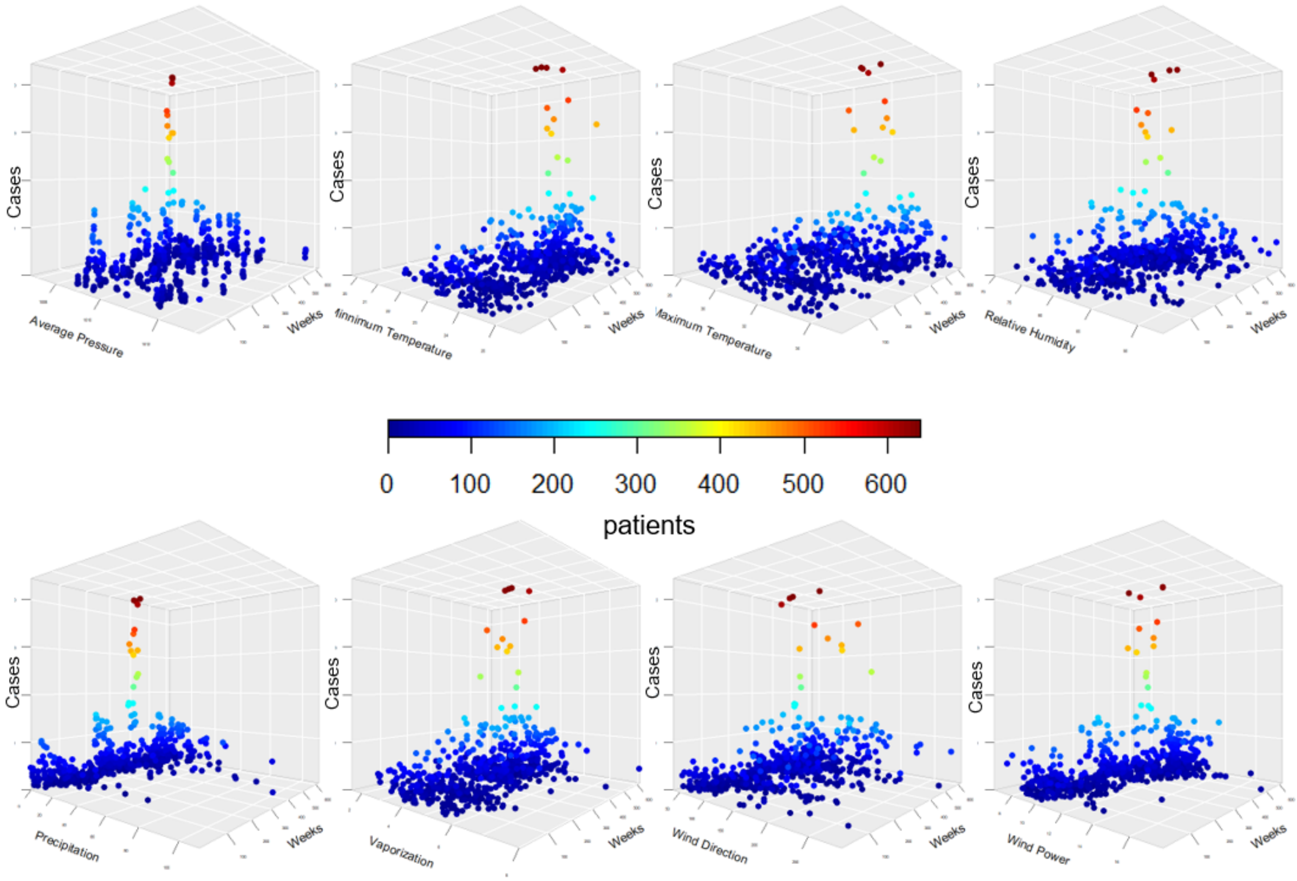

Figure 171: Three-dimensional scatter plot between dengue incidences and weather effects starting from January 2001 to December 2013 of Narathiwat.

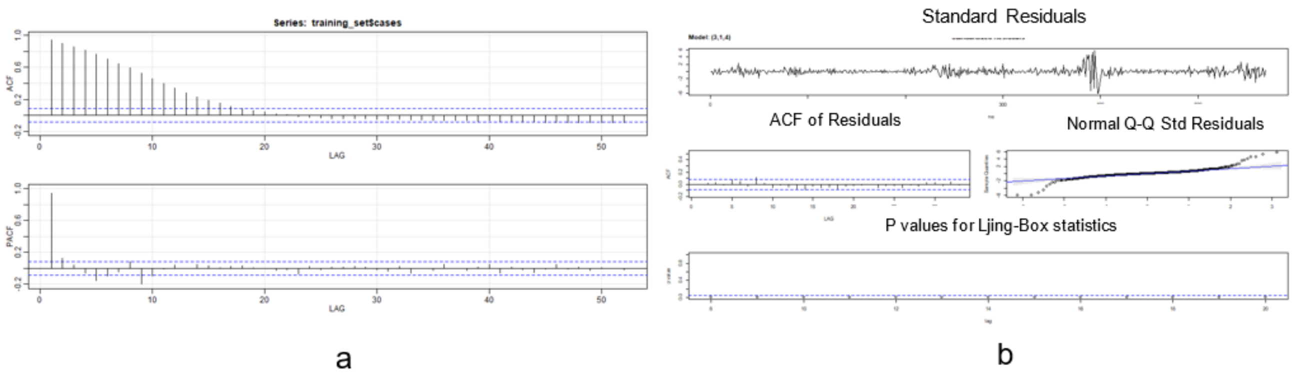

Figure 172: (a) Two plots between lag-time of dengue incidences and ACF and PACF relationship calculated from ARIMA model (b) Summary plots of time series analysis, multiple plots include the plot of predicted model over the time, the plot of ACF residual over lag-time of dengue incidences, residual Q-Q plot of standard residual, and p-value for Ljung-Box statistics of PACF relationship in Narathiwat over the training data starting from January 2001 to December 2013.

he best model of Narathiwat is based on quasi-likelihood method. The correlation coefficient on the test set in 2014 is 0.788 (95%CI: 0.7377, 0.8383). The model uses 9 variables. The most significant variables are 1-week-lag cases, following by 1-week-lag cases. Other variables which have less significant are, current week and 1-week-lag average pressure, 3-week-lag minimum temperature, 3-week-lag maximum temperature, current week precipitation, 1-week-lag vaporization, 3-week-lag wind direction. Time series methods by ARIMA and SARIMA yield the correlation coefficient of -0.532565 and -0.384156 respectively.

Table 57: Comparison table of all methods by the highest correlation coefficient (R^2) and the lowest prediction error (RMSE) in Narathiwat.

Methods	R-squared (R^2)	Root mean square error (RMSE)
Poisson Regression	0.5219849	11.16108
Negative Binomial Regression	0.6276711	9.850281
Quasi-likelihood Regression	0.7882371	7.428655
ARIMA (3,1,4)	-0.532565	19.98456
SARIMA $(2,0,1)(0,2,0)_{52}$	-0.384156	18.99231

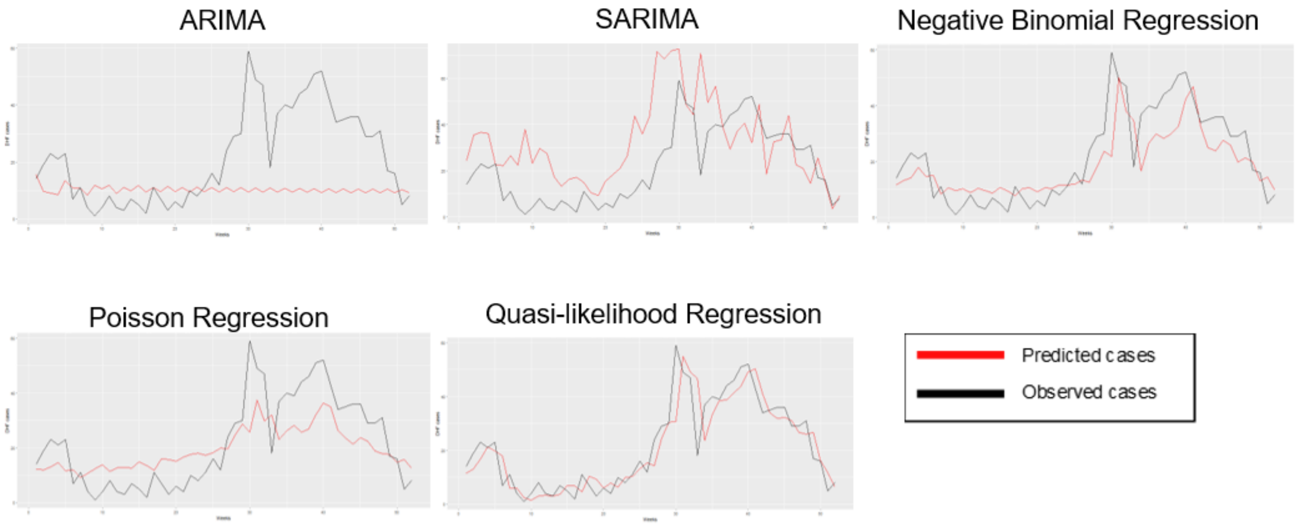

Figure 173: Plots between dengue cases and weeks, the black line represents the observed dengue cases, and the red line represents the predicted dengue cases of the best fit model of each technique over the test set data starting from January 2014 to December 2014.

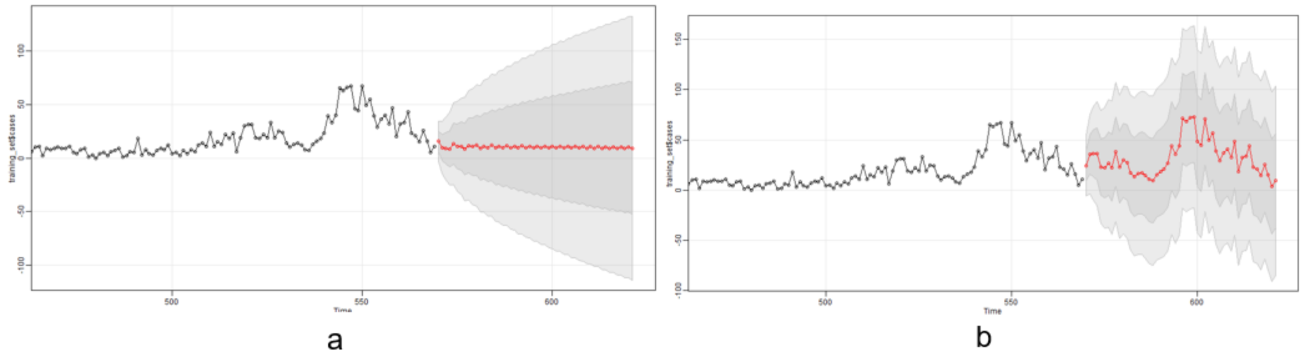

Figure 174: (a) Plot between dengue incidences over weekly time by the best model of ARIMA and (b) SARIMA time series analysis, the black line represents training set data starting from January 2012 to December 2013, and the red line represents the forecasted dengue incidences from January 2014 to December 2014.

Table 58: Coefficients and significant values of best fit GLM models, Negative Binomial, Poisson and Quasi-likelihood regression model of Narathiwat. The table summarizes coefficients of each independent variables which are composed in best fit model of each method. The significant of each variable is labelled by asterisks under the coefficients. The most important factor is marked as three asterisks which p-value ranges from 0 to 0.001. The second important factor is marked as two asterisks which p-value ranges from 0.001 to 0.01. The third important factor is marked as an asterisk which p-value ranges from 0.01 to 0.1. The least important is also marked as a dot which p-value ranges from 0.1 to 1.

**Independent variables**	**Lag**	**Coefficients/Significant**		
		NB	Poisson	Quasi
Intercept		-21.89	-2.9579324 ***	-122.2
Cases	1	0.02906 ***	0.0145749 ***	0.7987 ***
	2			0.1370 **
	3			
Average Pressure	0	0.02173		0.7879
	1			-0.7083
	2			
	3			
Minimum Temperature	0	-0.07731	-0.0747975 ***	
	1			
	2			
	3	-0.01684	-0.0157358	1.142
Maximum Temperature	0			
	1			
	2		0.0648830 ***	
	3	0.1046 ***	0.1161876 ***	0.5402
Relative Humidity	0			
	1			
	2	0.01049	0.0200706 ***	
	3			
Precipitation	0			-0.06263 .
	1			
	2			
	3	0.003579	0.0033224 **	
Vaporization	0			
	1			-0.3373
	2			
	3			
Wind Direction	0			
	1			
	2			
	3		0.0010418 ***	0.007296
Wind Power	0			
	1			
	2			
	3			

Nong Khai

Nong Khai is located in the northeastern continent of Thailand at coordinate of 15°02′05″N 102°44′40″E. Nong Khai covers an area of 3,027 km^2. Total population are 517,260 people. The density of population is approximately 171.0 people per km^2. Weather in Nong Khai has tropical savanna climate under the South Asian monsoon system. Temperature is high in April approximately 43.3°C and starts the low temperature from December to February (4.3-17.2°C). Winters are dry and warm. The monsoon season begins from May through October. The highest rainfall presents in August around 323.2 mm. Humidity is in range from 64-85 percent throughout the year. The highest sunshine hours are in March.

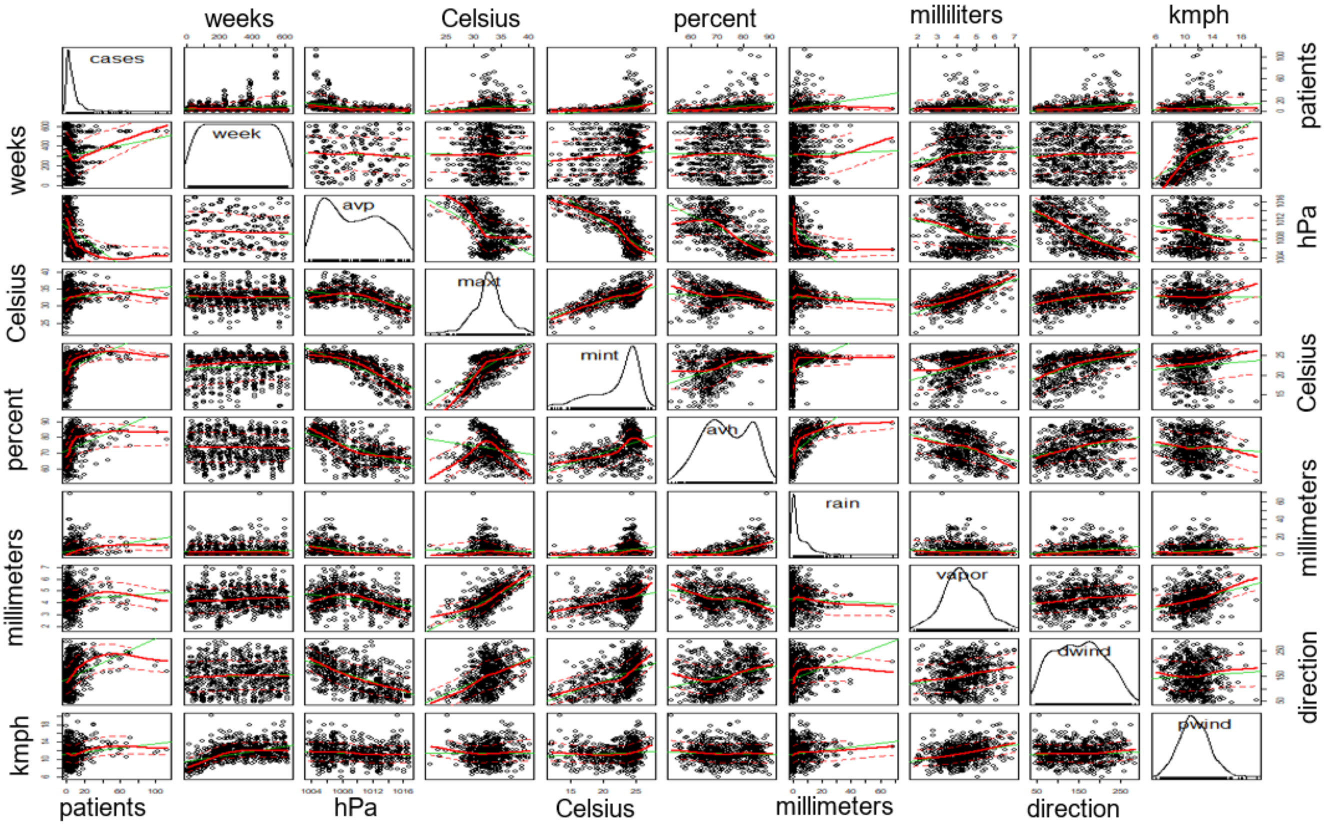

Figure 175: Scatter plot between dengue cases (cases) and selected independent variables, which are the weekly period starting from January 2001 – December 2013 (week), average pressure (avp), maximum temperature (maxt), minimum temperature (mint), average humidity (avh), precipitation (rain), vaporization of water (vapor), wind direction (dwind), and wind power (pwind). The plot visualizes pairwise hundred relationships of training set in Nong Khai.

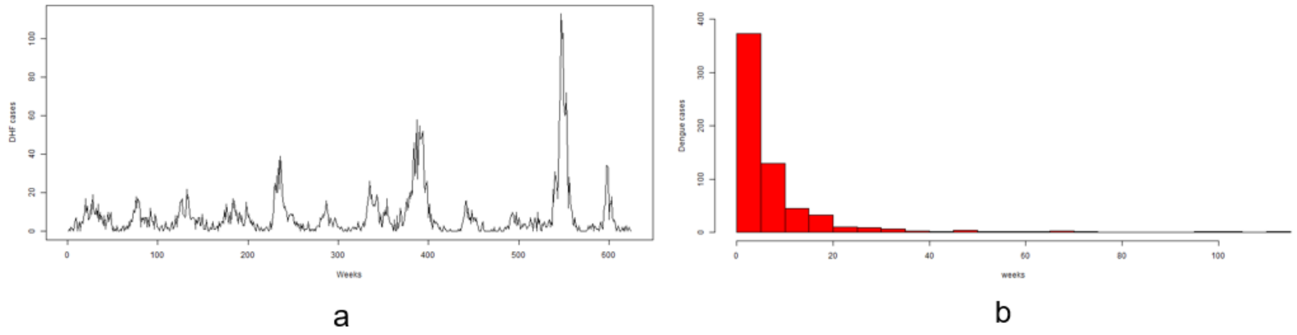

Figure 176: (a) Line plot between dengue incidences and weeks, the plot shows trends of dengue incidences in each year as stationary time series. (b) Histogram of dengue incidences in Nong Khai starting from January 2001 to December 2013 (624 weeks).

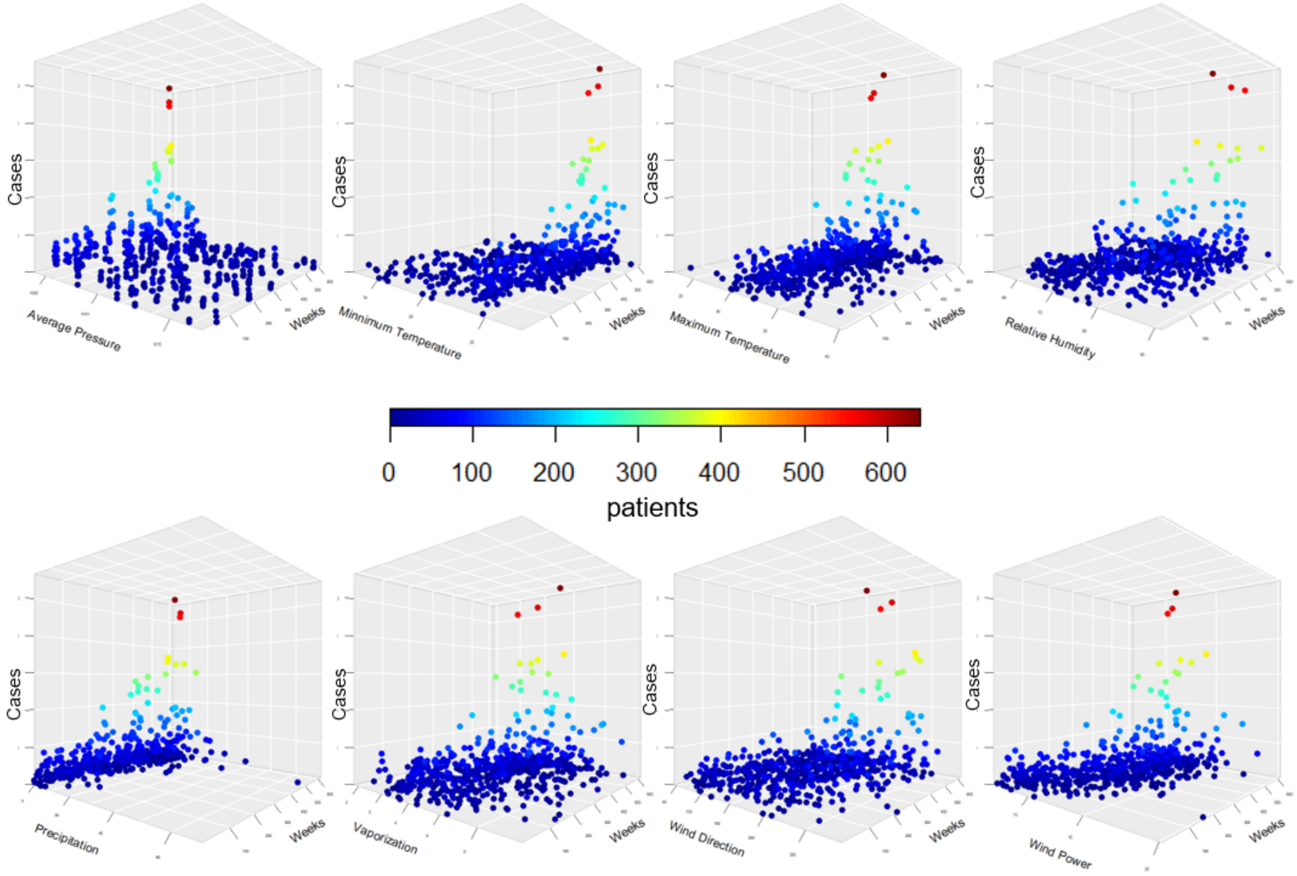

Figure 177: Three-dimensional scatter plot between dengue incidences and weather effects starting from January 2001 to December 2013 of Nong Khai.

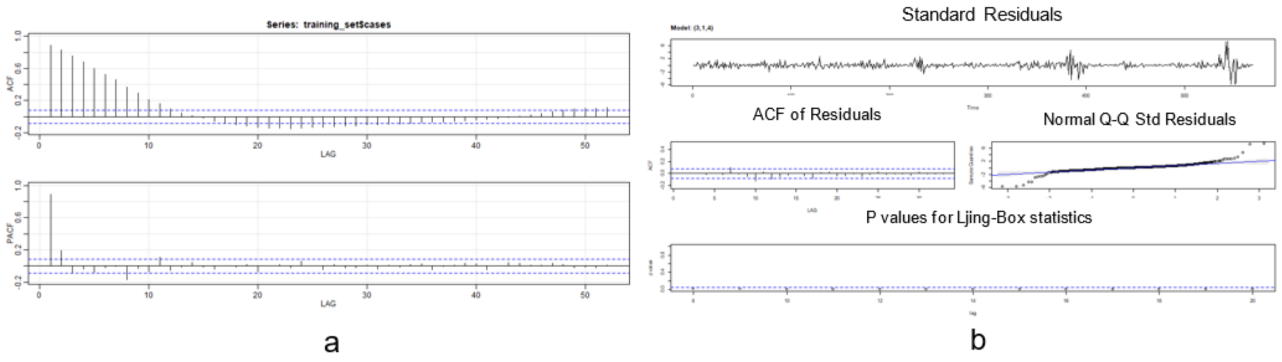

Figure 178: (a) Two plots between lag-time of dengue incidences and ACF and PACF relationship calculated from ARIMA model (b) Summary plots of time series analysis, multiple plots include the plot of predicted model over the time, the plot of ACF residual over lag-time of dengue incidences, residual Q-Q plot of standard residual, and p-value for Ljung-Box statistics of PACF relationship in Nong Khai over the training data starting from January 2001 to December 2013.

For Nong Khai, the best model is based on Poisson regression method. The correlation coefficient on the test set in 2014 is 0.746 (95%CI: 0.6856, 0.8064). The significant of the variables associated with p-value statistical calculation are shown in 59. The best model of Nong Khai uses 8 variables. The most significant variables are 1-week-lag cases, 1-week-lag average pressure, 3-week-lag maximum temperature, 2-week-lag relative humidity, current week and 3-week-lag wind direction. Other variables which have less significant are, current week and 2-week-lag precipitation. Time series methods by ARIMA and SARIMA yield the correlation coeffect of -0.3431212 and -13.5079 respectively.

Table 59: Comparison table of all methods by the highest correlation coefficient (R^2) and the lowest prediction error (RMSE) in nongkhai.

**Methods**	**R-squared (R^2)**	**Root mean square error (RMSE)**
Poisson Regression	0.7463212	4.150292
Negative Binomial Regression	0.3129972	6.829923
Quasi-likelihood Regression	0.6463159	4.900544
ARIMA (3,1,4)	-0.3431212	9.549797
SARIMA $(2,0,1)(0,2,0)_{52}$	-13.5079	31.3862

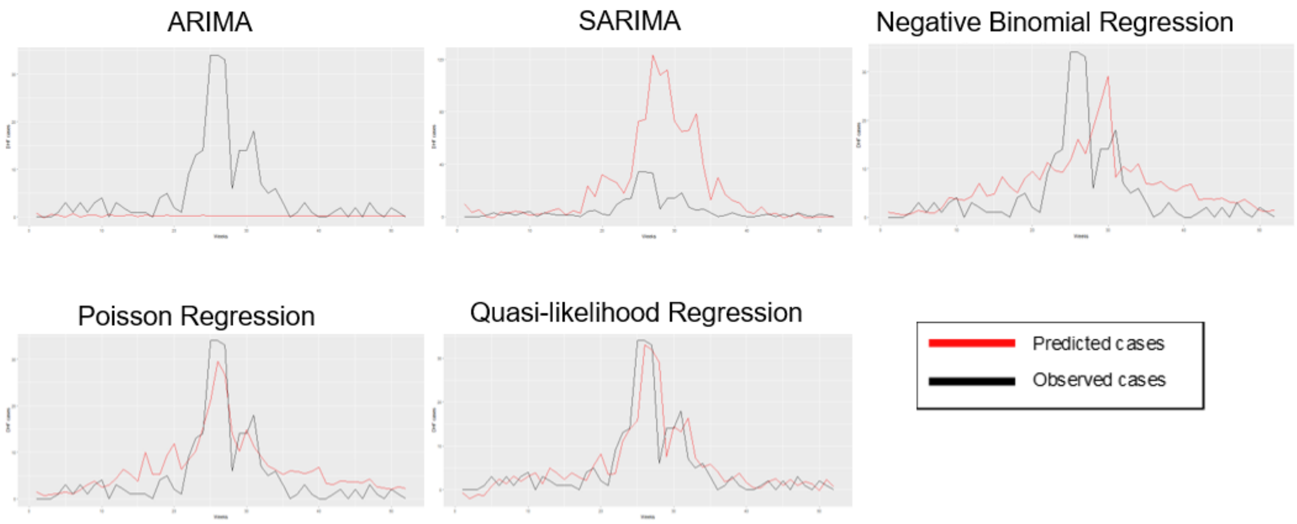

Figure 179: Plots between dengue cases and weeks, the black line represents the observed dengue cases, and the red line represents the predicted dengue cases of the best fit model of each technique over the test set data starting from January 2014 to December 2014.

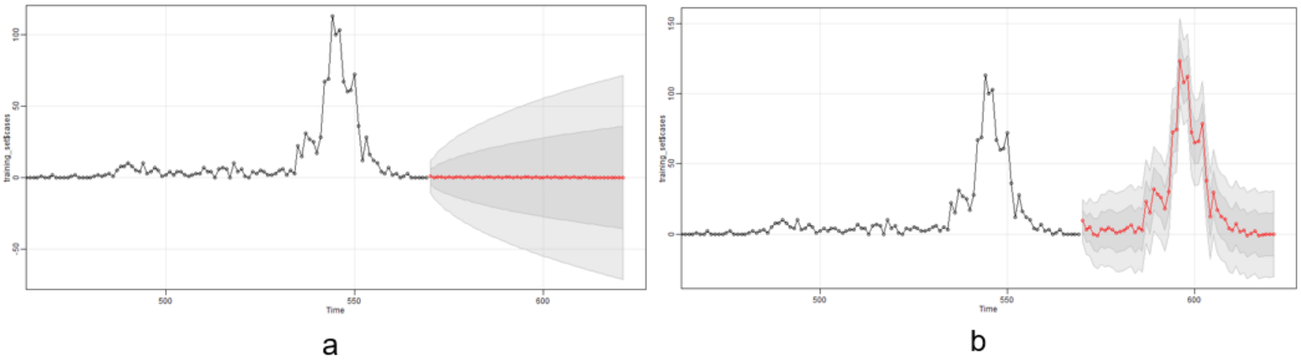

Figure 180: (a) Plot between dengue incidences over weekly time by the best model of ARIMA and (b) SARIMA time series analysis, the black line represents training set data starting from January 2012 to December 2013, and the red line represents the forecasted dengue incidences from January 2014 to December 2014.

Table 60: Coefficients and significant values of best fit GLM models, Negative Binomial, Poisson and Quasi-likelihood regression model of Nong Khai. The table summarizes coefficients of each independent variables which are composed in best fit model of each method. The significant of each variable is labelled by asterisks under the coefficients. The most important factor is marked as three asterisks which p-value ranges from 0 to 0.001. The second important factor is marked as two asterisks which p-value ranges from 0.001 to 0.01. The third important factor is marked as an asterisk which p-value ranges from 0.01 to 0.1. The least important is also marked as a dot which p-value ranges from 0.1 to 1.

**Independent variables**	**Lag**	**Coefficients/Significant**		
		NB	Poisson	Quasi
Intercept		80.084438 ***	88.7282712 ***	160.307842
Cases	1		0.0280068 ***	0.864546 ***
	2			
	3	0.038838 ***		
Average Pressure	0			-0.170452
	1		-0.0918982 ***	
	2	-0.083429 ***		
	3			
Minimum Temperature	0			
	1			
	2			
	3	0.071274 ***		-0.128808
Maximum Temperature	0	0.123460 ***		
	1			
	2			
	3		0.1176696 ***	0.379663 *
Relative Humidity	0			
	1			
	2		0.0206027 ***	0.037860
	3			
Precipitation	0		-0.0051936 *	-0.014214
	1			
	2		-0.0043511	
	3			
Vaporization	0	-0.180208 **		
	1			
	2			
	3	0.061610		
Wind Direction	0		0.0021067 ***	0.003937
	1			
	2			
	3		-0.0011012 ***	-0.008381
Wind Power	0	0.027998		
	1			
	2			
	3			0.111968

Pathum Thani

Pathum Thani is a province located in the central region of Thailand at coordinate of 14°03′00″N 100°29′0″E. Pathum Thani covers an area of 1,526 km^2. Total population are 1,075,058 people. The density of population is 704.0 people per km^2. Weather in Pathum Thani follows tropical savanna climate system. The highest temperature is in April approximately 40.0°C. The low temperature presents in winter from December to March. The monsoon season starts from May through August. The highest sunshine hours are in January.

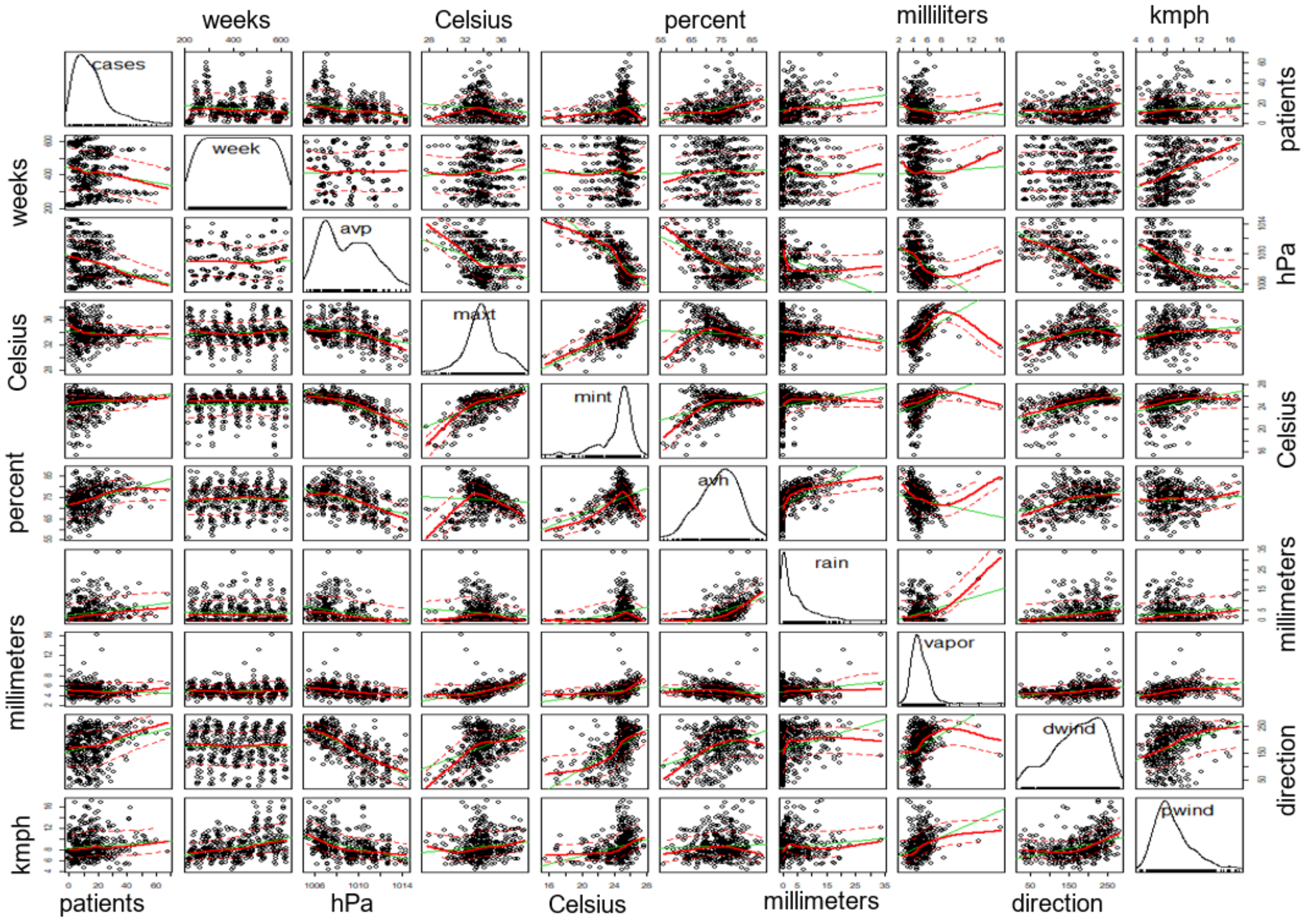

Figure 181: Scatter plot between dengue cases (cases) and selected independent variables, which are the weekly period starting from January 2001 – December 2013 (week), average pressure (avp), maximum temperature (maxt), minimum temperature (mint), average humidity (avh), precipitation (rain), vaporization of water (vapor), wind direction (dwind), and wind power (pwind). The plot visualizes pairwise hundred relationships of training set in Pathum Thani.

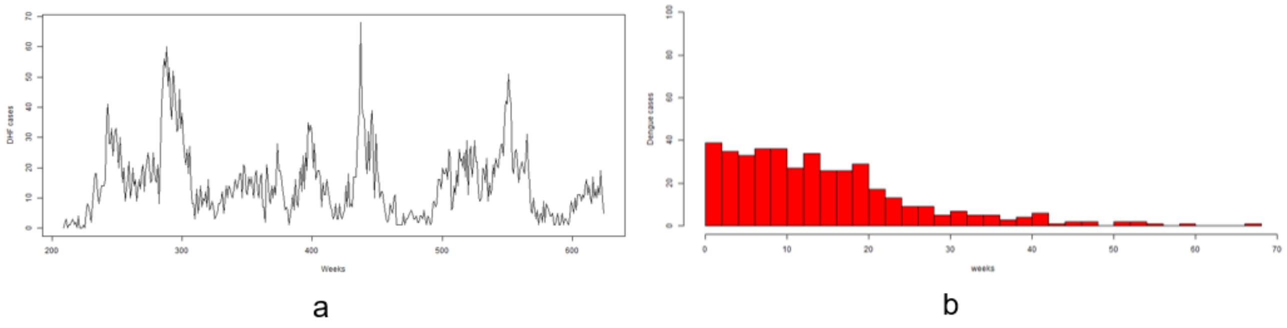

Figure 182: (a) Line plot between dengue incidences and weeks, the plot shows trends of dengue incidences in each year as stationary time series. (b) Histogram of dengue incidences in Pathum Thani starting from January 2001 to December 2013 (624 weeks).

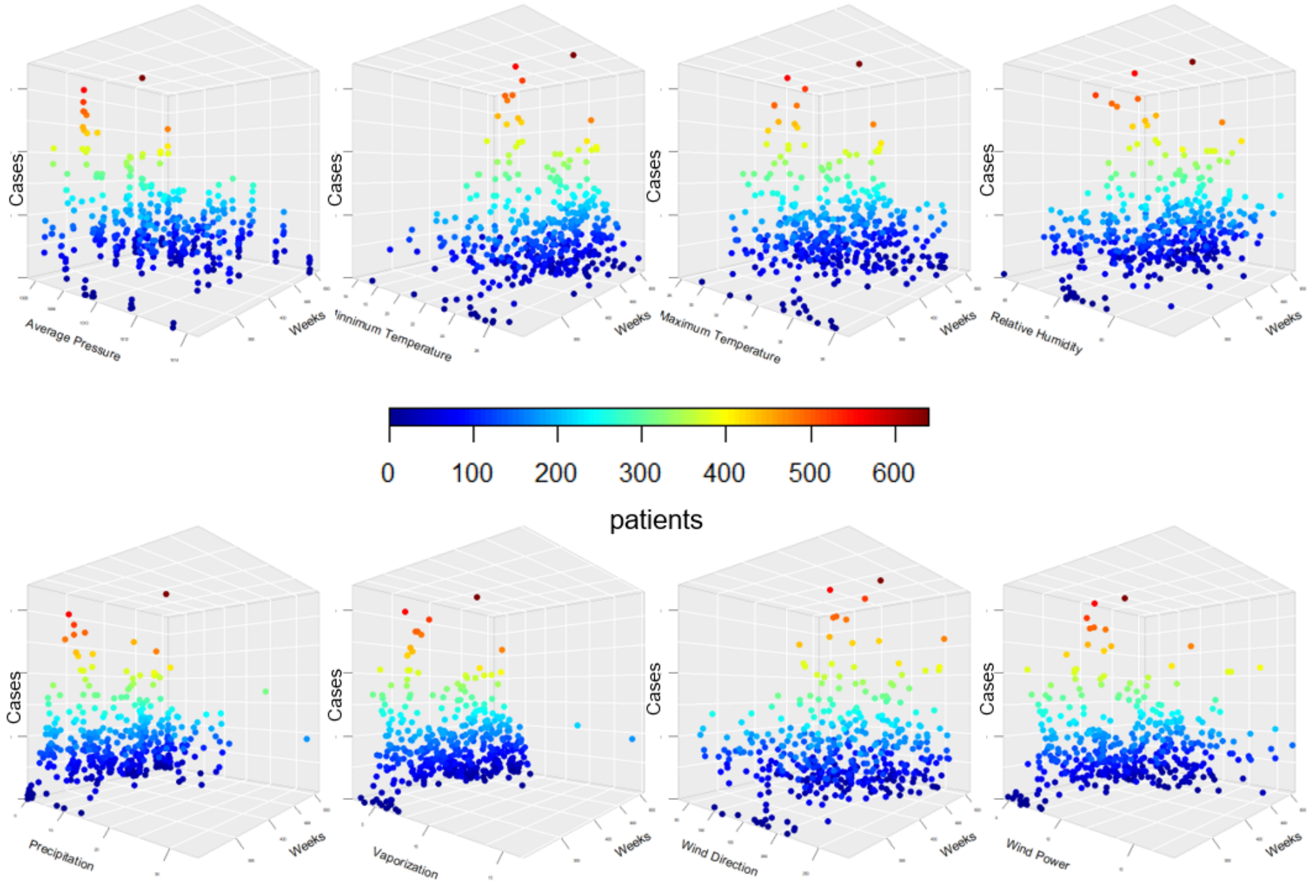

Figure 183: Three-dimensional scatter plot between dengue incidences and weather effects starting from January 2001 to December 2013 of Pathum Thani.

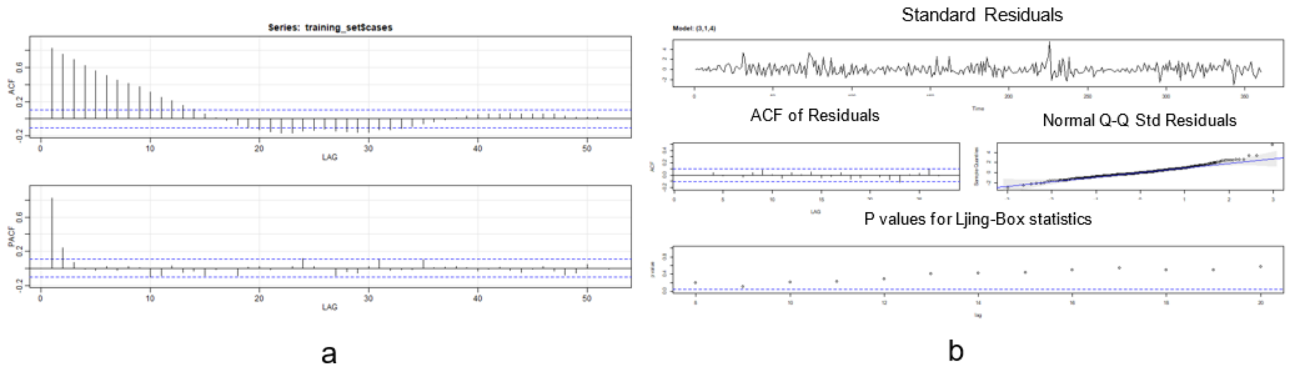

Figure 184: (a) Two plots between lag-time of dengue incidences and ACF and PACF relationship calculated from ARIMA model (b) Summary plots of time series analysis, multiple plots include the plot of predicted model over the time, the plot of ACF residual over lag-time of dengue incidences, residual Q-Q plot of standard residual, and p-value for Ljung-Box statistics of PACF relationship in Pathum Thani over the training data starting from January 2001 to December 2013.

The best model of Pathum Thani is based on quasi-likelihood method. The correlation coefficient on the test set in 2014 is 0.574 (95%CI: 0.4761, 0.6719). The best model consists of 8 variables. The most significant variables are 2-week-lag cases, 3-week-lag cases and 1-week-lag minimum temperature. Other variables which have less significant are, 3-week-lag minimum temperature, 3-week-lag maximum temperature, 1-week-lag and 3-week-lag precipitation, and 1-week-lag vaporization. Time series methods by ARIMA and SARIMA yield the correlation coefficient of -1.927344 and -12.26281 respectively.

Table 61: Comparison table of all methods by the highest correlation coefficient (R^2) and the lowest prediction error (RMSE) in Pathum Thani.

**Methods**	**R-squared (R^2)**	**Root mean square error (RMSE)**
Poisson Regression	0.3133683	3.871706
Negative Binomial Regression	0.4669447	3.411352
Quasi-likelihood Regression	0.5738493	3.050156
ARIMA (3,1,4)	-1.927344	7.994242
SARIMA $(2,0,1)(0,2,0)_{52}$	-12.26281	17.01603

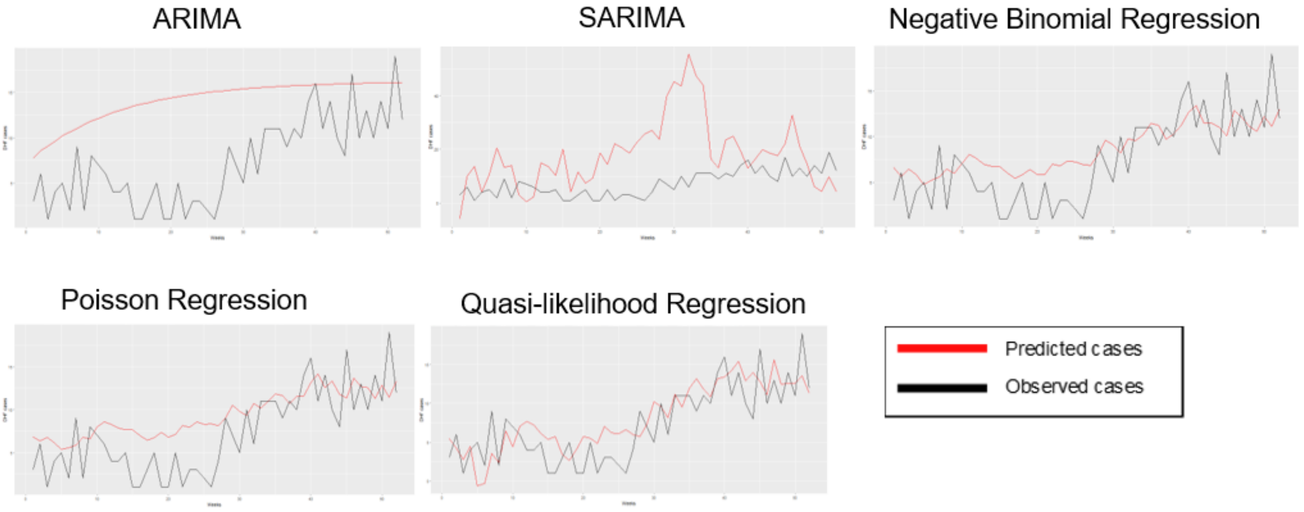

Figure 185: Plots between dengue cases and weeks, the black line represents the observed dengue cases, and the red line represents the predicted dengue cases of the best fit model of each technique over the test set data starting from January 2014 to December 2014.

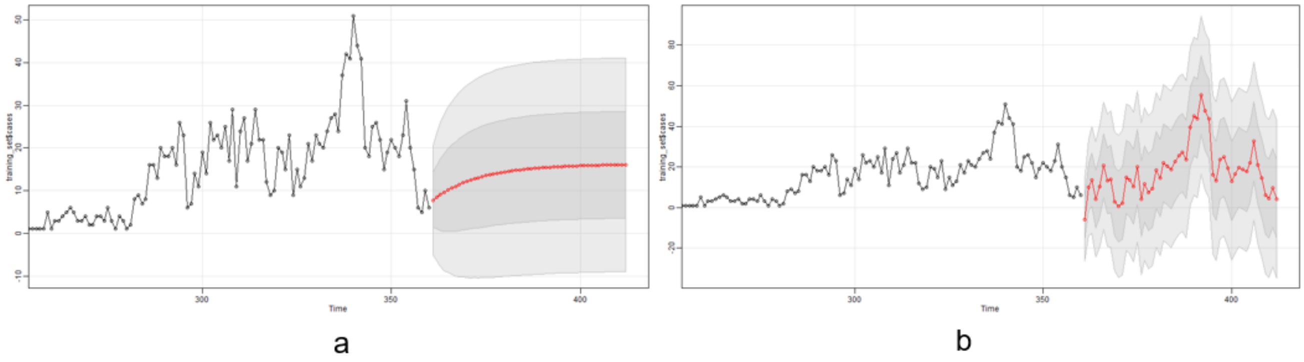

Figure 186: (a) Plot between dengue incidences over weekly time by the best model of ARIMA and (b) SARIMA time series analysis, the black line represents training set data starting from January 2012 to December 2013, and the red line represents the forecasted dengue incidences from January 2014 to December 2014.

Table 62: Coefficients and significant values of best fit GLM models, Negative Binomial, Poisson and Quasi-likelihood regression model of Pathum Thani. The table summarizes coefficients of each independent variables which are composed in best fit model of each method. The significant of each variable is labelled by asterisks under the coefficients. The most important factor is marked as three asterisks which p-value ranges from 0 to 0.001. The second important factor is marked as two asterisks which p-value ranges from 0.001 to 0.01. The third important factor is marked as an asterisk which p-value ranges from 0.01 to 0.1. The least important is also marked as a dot which p-value ranges from 0.1 to 1.

**Independent variables**	**Lag**	**Coefficients/Significant**		
		NB	Poisson	Quasi
Intercept		1.606360 **	1.263837 ***	-7.08602
Cases	1	0.032865 ***	0.026598 ***	
	2	0.015202 ***	0.008372 ***	0.55489 ***
	3		0.004474 **	0.21206 ***
Average Pressure	0			
	1			
	2			
	3			
Minimum Temperature	0			
	1	0.034052	0.037988 **	0.61713 *
	2			
	3	0.044643 .	0.043462 **	0.35630
Maximum Temperature	0			
	1			
	2			
	3	-0.048310 *	-0.031934 **	-0.36383
Relative Humidity	0			
	1			
	2			
	3			
Precipitation	0			
	1			0.05258
	2			
	3	-0.003617		-0.06034
Vaporization	0			
	1	-0.010033	-0.019141 .	-0.14827
	2			
	3		-0.017513	
Wind Direction	0			
	1			
	2			
	3			
Wind Power	0			
	1			
	2			
	3			

Pattani

Pattani is a province located in southern Thailand at 6°51′59″ N 101°15′03″ E. Pattani covers an area of 1,940 km^2. Total population are 686,186 people. The density of population is 354.0 people per km^2. Weather in Pattani has tropical savanna climate under the South Asian monsoon system which has shorter winter period. Temperature in Pattani has the highest of 38°C in May and the lowest of 18.6°C in January. Pattani has a dry season that runs from December through March and a wet season that covers the other eight months. The rainy season begins with the arrival of the southwest monsoon around mid-May. Precipitation occurs from mid-May to August. Rainfall are roughly 406.6 mm in November. Humidity presents in the range of 78-86 percent throughout the year. February is the highest sunshine hours.

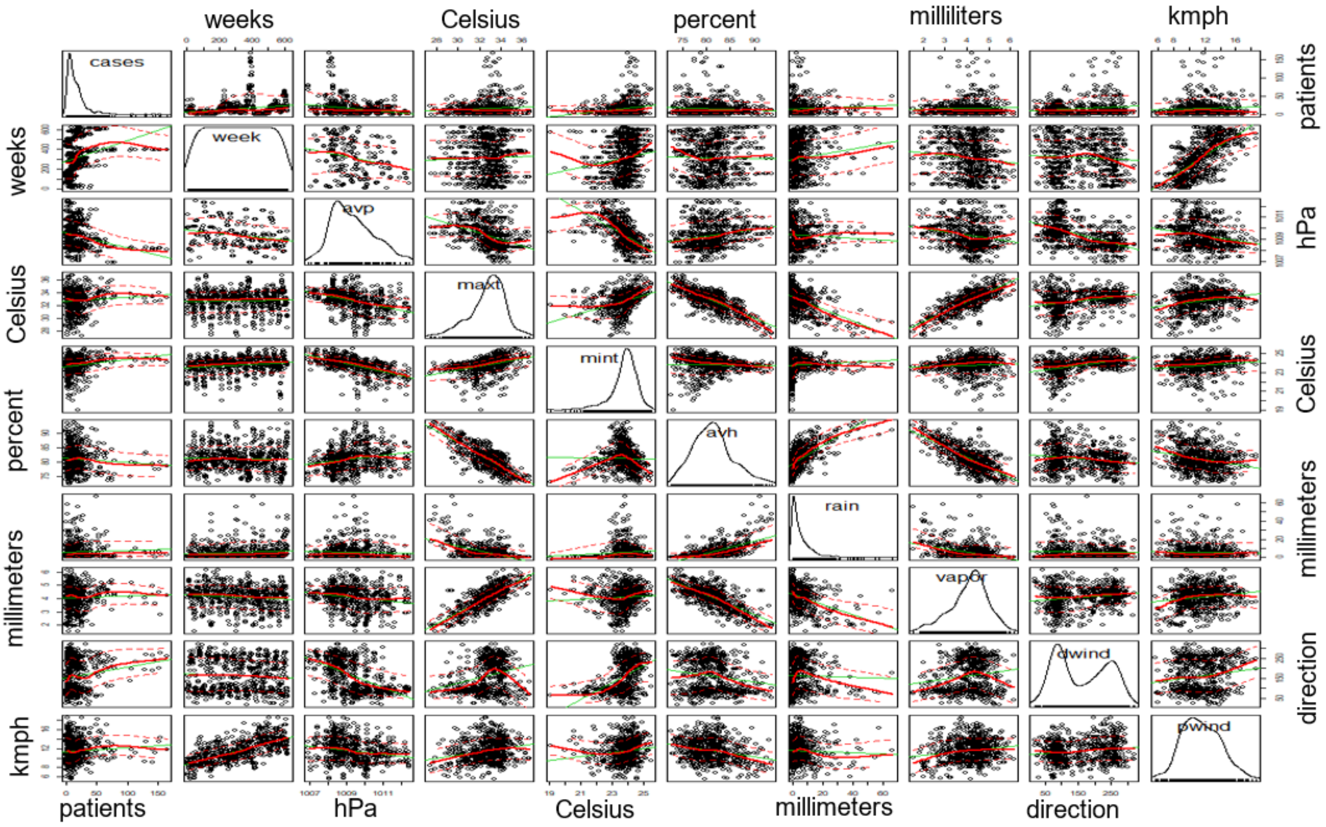

Figure 187: Scatter plot between dengue cases (cases) and selected independent variables, which are the weekly period starting from January 2001 – December 2013 (week), average pressure (avp), maximum temperature (maxt), minimum temperature (mint), average humidity (avh), precipitation (rain), vaporization of water (vapor), wind direction (dwind), and wind power (pwind). The plot visualizes pairwise hundred relationships of training set in Pattani.

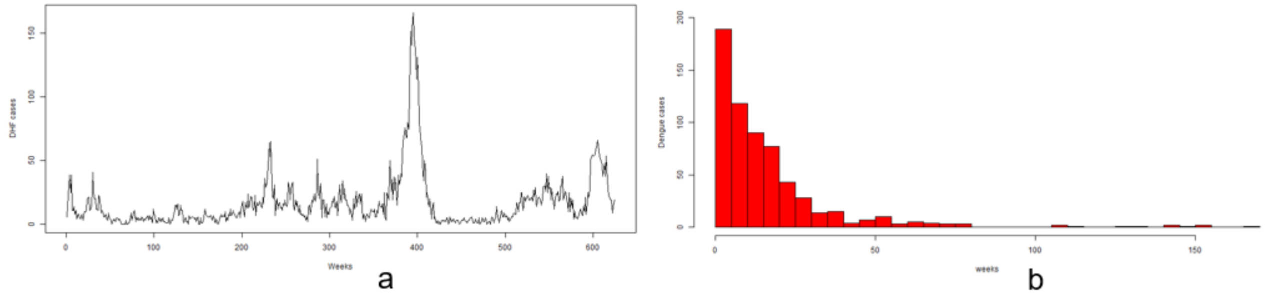

Figure 188: (a) Line plot between dengue incidences and weeks, the plot shows trends of dengue incidences in each year as stationary time series. (b) Histogram of dengue incidences in Pattani starting from January 2001 to December 2013 (624 weeks).

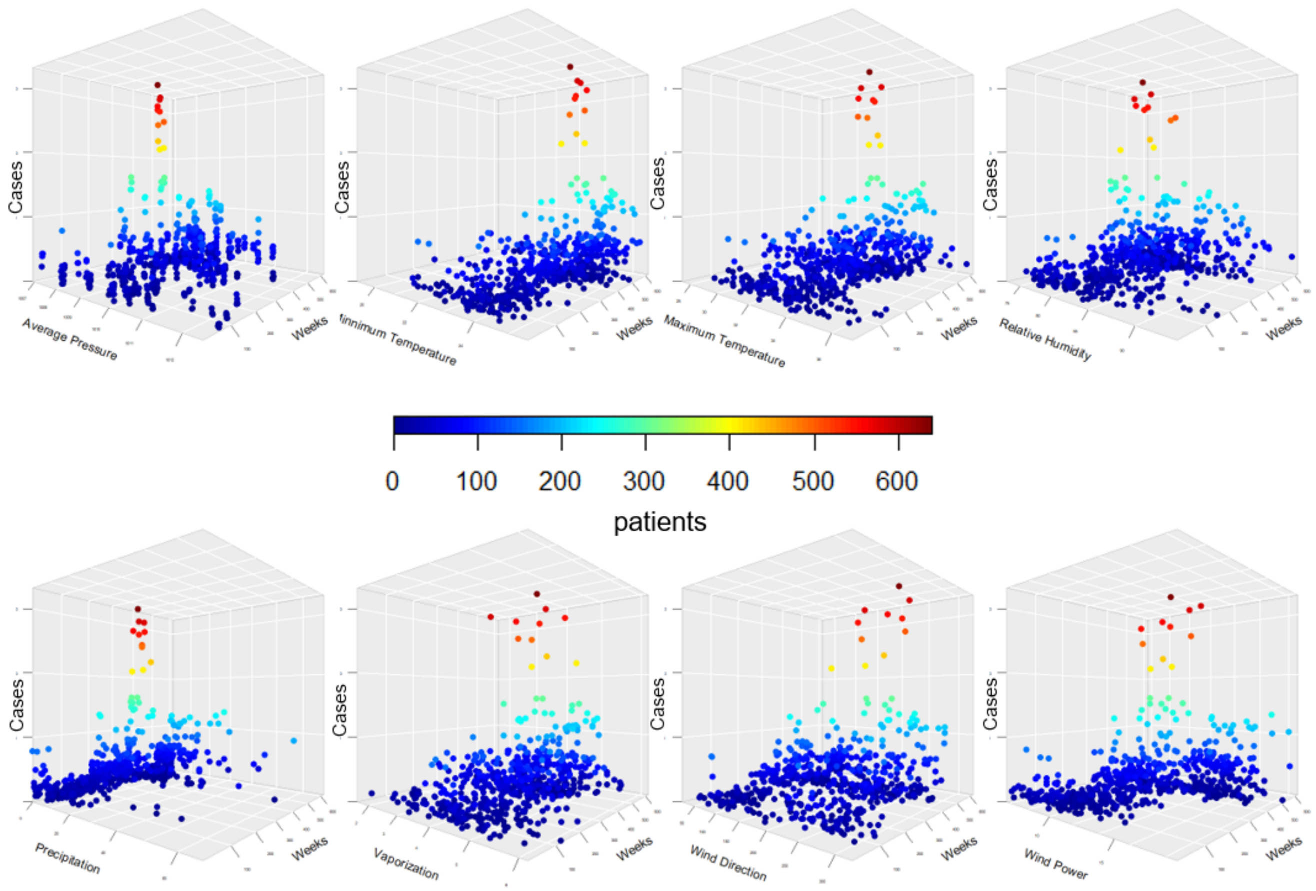

Figure 189: Three-dimensional scatter plot between dengue incidences and weather effects starting from January 2001 to December 2013 of Pattani.

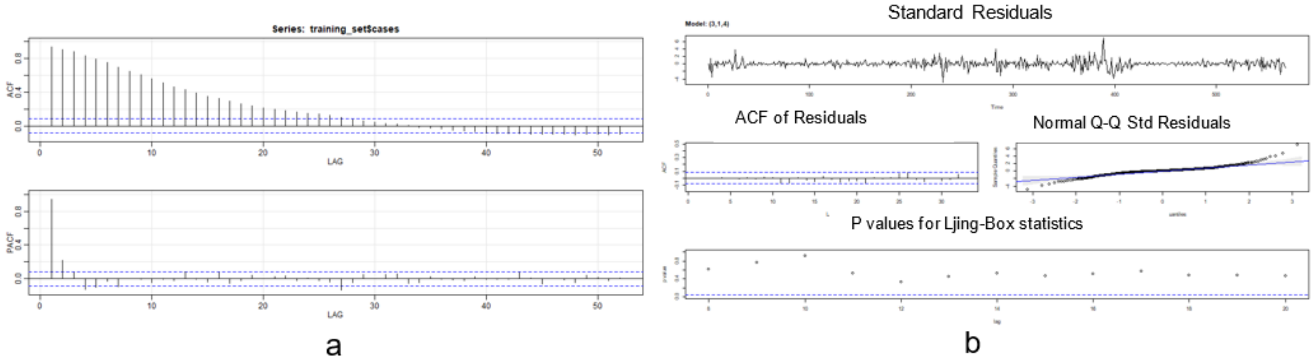

Figure 190: (a) Two plots between lag-time of dengue incidences and ACF and PACF relationship calculated from ARIMA model (b) Summary plots of time series analysis, multiple plots include the plot of predicted model over the time, the plot of ACF residual over lag-time of dengue incidences, residual Q-Q plot of standard residual, and p-value for Ljung-Box statistics of PACF relationship in Pattani over the training data starting from January 2001 to December 2013.

For Pattani, the best model is based on quasi-likelihood method. The correlation coefficient on the test set in 2014 is 0.882 (95%CI: 0.8079, 0.9561). The significant of the variables associated with p-value statistical calculation are shown in Table PTN2. The best model uses 8 variables. The most significant variables are 1-week-lag cases, following by 3-week-lag precipitation, 2-week-lag cases, current week minimum temperature and 2-week-lag maximum temperature. Other variables which have less significant are, current week precipitation, wind direction and wind power. Time series methods by ARIMA and SARIMA yield the correlation coefficient of -0.7078189 and -0.08945556 respectively.

Table 63: Comparison table of all methods by the highest correlation coefficient (R^2) and the lowest prediction error (RMSE) in pattani.

Methods	R-squared (R^2)	Root mean square error (RMSE)
Poisson Regression	0.5592165	12.94211
Negative Binomial Regression	0.8188598	8.296594
Quasi-likelihood Regression	0.8820925	6.693652
ARIMA (3,1,4)	-0.7078189	25.47495
SARIMA $(2,0,1)(0,2,0)_{52}$	-0.08945556	20.34685

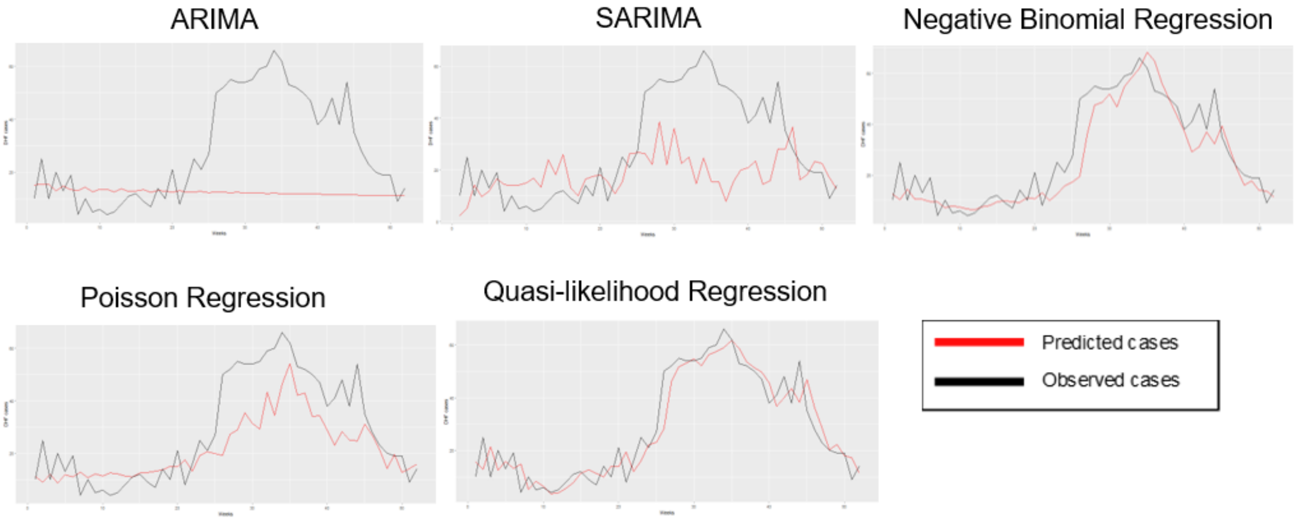

Figure 191: Plots between dengue cases and weeks, the black line represents the observed dengue cases, and the red line represents the predicted dengue cases of the best fit model of each technique over the test set data starting from January 2014 to December 2014.

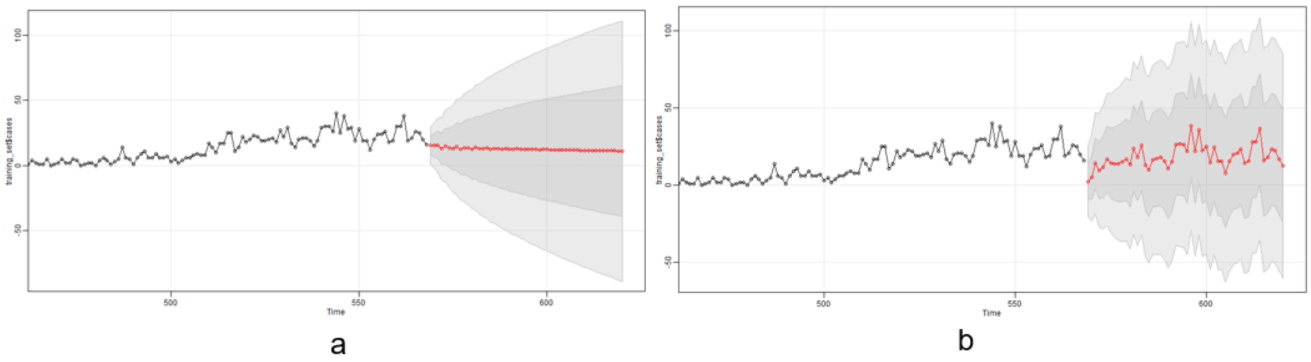

Figure 192: (a) Plot between dengue incidences over weekly time by the best model of ARIMA and (b) SARIMA time series analysis, the black line represents training set data starting from January 2012 to December 2013, and the red line represents the forecasted dengue incidences from January 2014 to December 2014.

Table 64: Coefficients and significant values of best fit GLM models, Negative Binomial, Poisson and Quasi-likelihood regression model of Pattani. The table summarizes coefficients of each independent variables which are composed in best fit model of each method. The significant of each variable is labelled by asterisks under the coefficients. The most important factor is marked as three asterisks which p-value ranges from 0 to 0.001. The second important factor is marked as two asterisks which p-value ranges from 0.001 to 0.01. The third important factor is marked as an asterisk which p-value ranges from 0.01 to 0.1. The least important is also marked as a dot which p-value ranges from 0.1 to 1.

**Independent variables**	**Lag**	**Coefficients/Significant**		
		NB	Poisson	Quasi
Intercept		0.7442805	1.9433372 ***	-254.99211
Cases	1	0.0265281 ***	0.0209798 ***	0.7327500 ***
	2	0.0109392 **		0.2139400 ***
	3			
Average Pressure	0			1.35774000 .
	1			-1.10629
	2			
	3			
Minimum Temperature	0			
	1	0.0662156		0.9796700 .
	2			
	3			
Maximum Temperature	0			
	1			
	2			
	3			
Relative Humidity	0		-0.0084907 .	
	1	-0.0053775		-0.26508000 **
	2			
	3			
Precipitation	0		0.0082748 ***	
	1		0.0029255 .	
	2			-0.04374
	3	0.0064568 .	0.0109445 ***	0.09059000 *
Vaporization	0			
	1		0.0964404 ***	
	2		0.0758319 ***	
	3			
Wind Direction	0			
	1	-0.0004413		
	2			
	3			
Wind Power	0		0.0112888 *	
	1			
	2			
	3			

Phang nga

Phang nga is a province located in southern region of Thailand at 8°27′52″N 98°31′54″E. Phang nga covers an area of 4,171 km^2. Total population are 261,370 people. The density of population is 63.0 people per km^2. Weather in Phang nga has tropical savanna climate under the South Asian monsoon system which has shorter winter period. Phang nga has a dry season that runs from December through March and a wet season that covers the other eight months. The rainy season begins with the arrival of the southwest monsoon around mid-May. Precipitation occurs from mid-May to August. Rainfall are roughly 250 mm in October. Humidity presents in the range of 76-82 percent throughout the year. January is the highest sunshine hours.

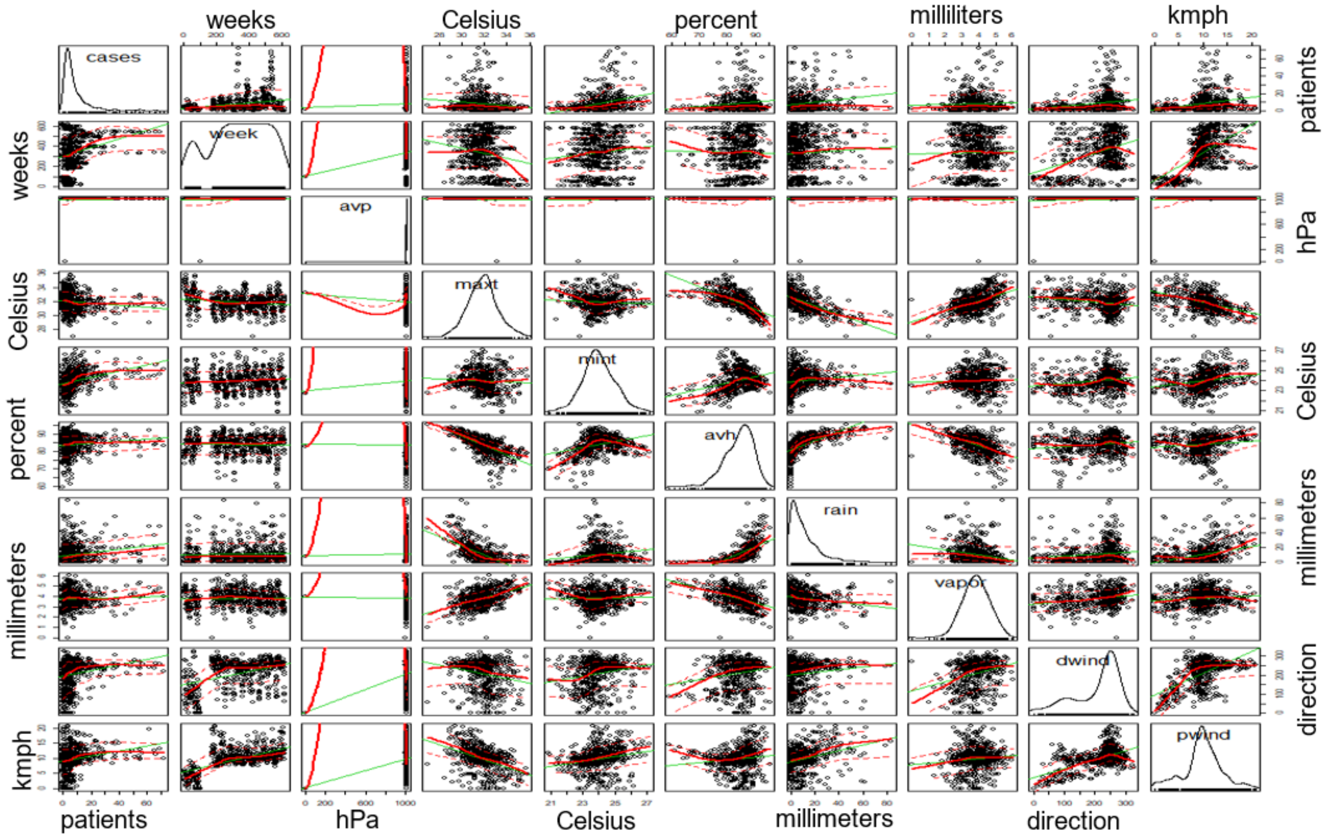

Figure 193: Scatter plot between dengue cases (cases) and selected independent variables, which are the weekly period starting from January 2001 – December 2013 (week), average pressure (avp), maximum temperature (maxt), minimum temperature (mint), average humidity (avh), precipitation (rain), vaporization of water (vapor), wind direction (dwind), and wind power (pwind). The plot visualizes pairwise hundred relationships of training set in Phang nga.

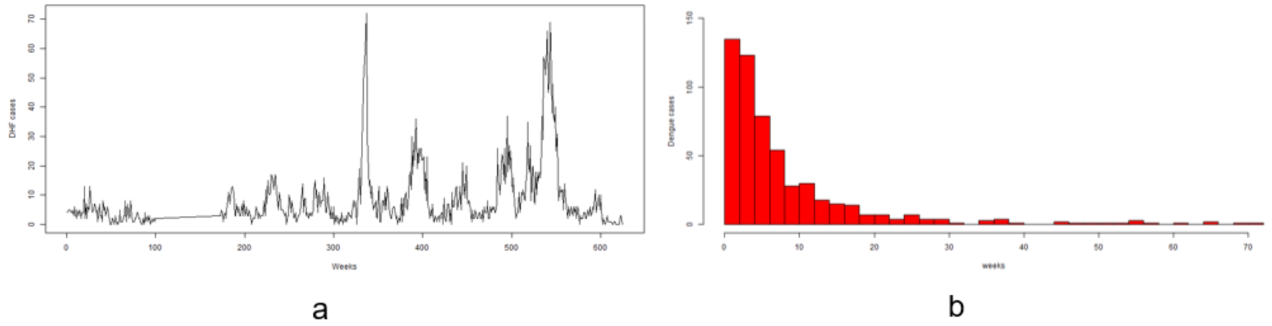

Figure 194: (a) Line plot between dengue incidences and weeks, the plot shows trends of dengue incidences in each year as stationary time series. (b) Histogram of dengue incidences in Phang nga starting from January 2001 to December 2013 (624 weeks).

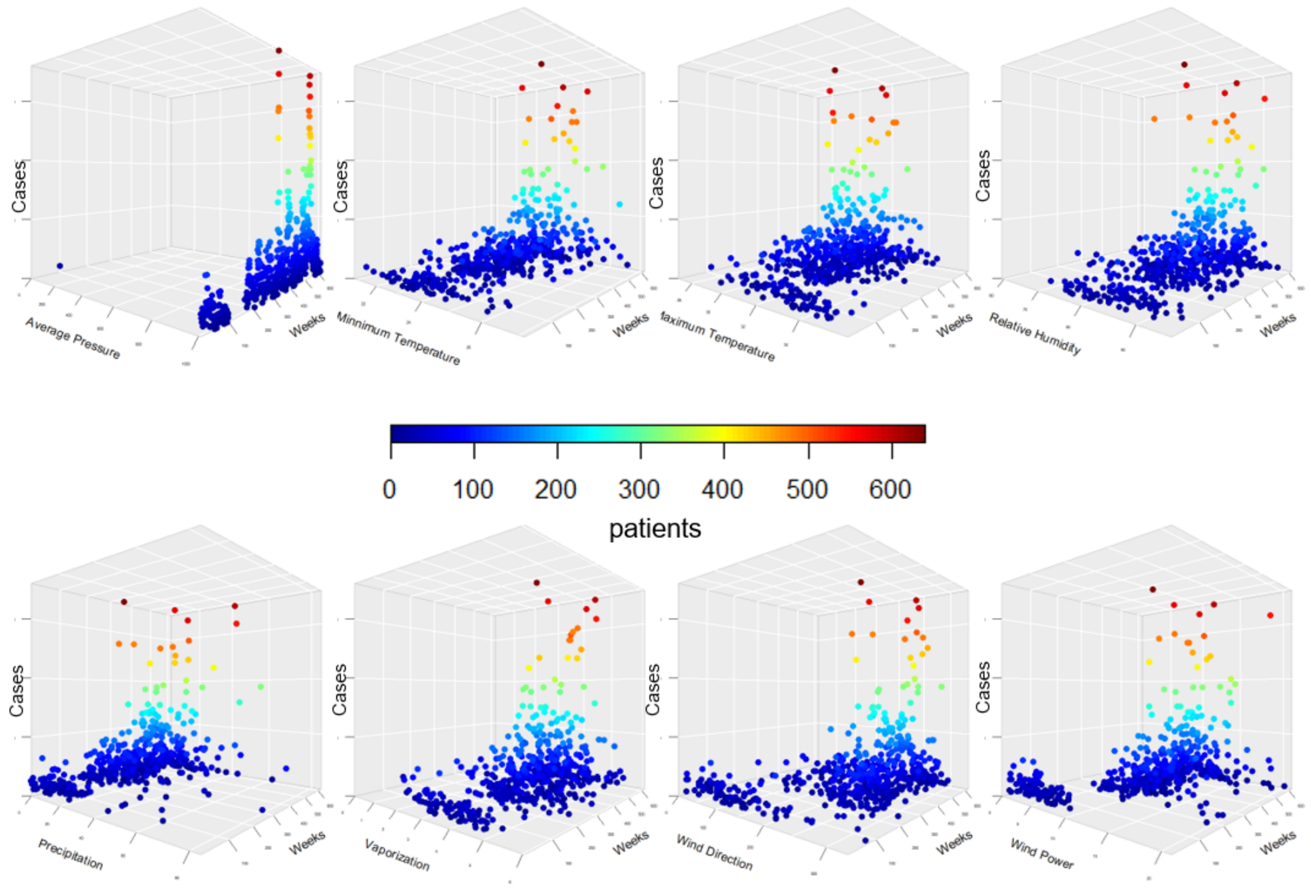

Figure 195: Three-dimensional scatter plot between dengue incidences and weather effects starting from January 2001 to December 2013 of Phang nga.

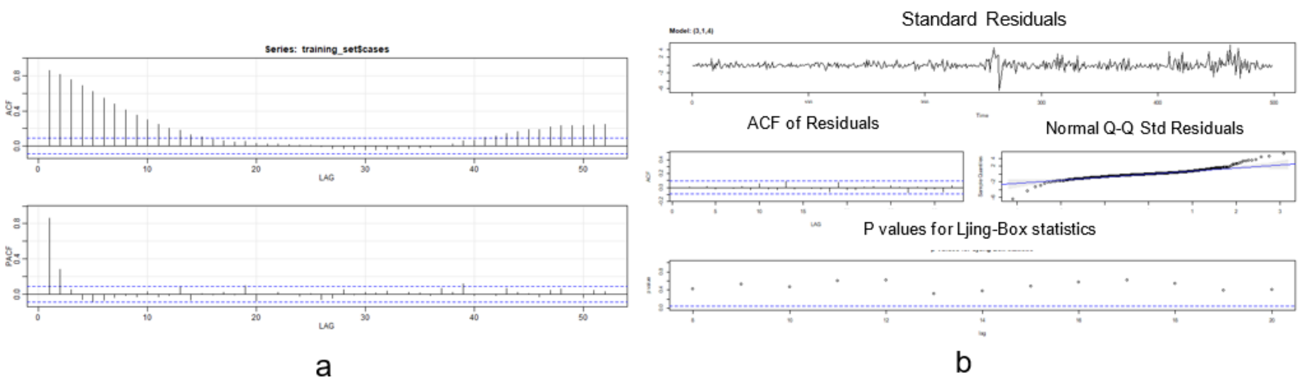

Figure 196: (a) Two plots between lag-time of dengue incidences and ACF and PACF relationship calculated from ARIMA model (b) Summary plots of time series analysis, multiple plots include the plot of predicted model over the time, the plot of ACF residual over lag-time of dengue incidences, residual Q-Q plot of standard residual, and p-value for Ljung-Box statistics of PACF relationship in Phang nga over the training data starting from January 2001 to December 2013.

The best model of Phang nga province is based on quasi-likelihood method. The correlation coefficient on the test set in 2014 is 0.43 (95%CI: 0.3456, 0.5144). The significant of the variables associated with p-value statistical calculation are shown in Table PNG2. The model consists of 9 variables. The most significant variables are 1-week-lag cases, 2-week-lag cases, following by 3-week-lag vaporization. Other variables which have less significant are, 3-week-lag cases, 2-week-lag relative humidity, 1-week-lag and 2-week-lag wind power. Time series methods by ARIMA and SARIMA yield the correlation coeffect of -22.37356 and -91.4538 respectively.

Table 65: Comparison table of all methods by the highest correlation coefficient (R^2) and the lowest prediction error (RMSE) in Phang nga.

**Methods**	**R-squared (R^2)**	**Root mean square error (RMSE)**
Poisson Regression	-0.3942351	3.472355
Negative Binomial Regression	0.02183544	2.908453
Quasi-likelihood Regression	0.4300687	2.220073
ARIMA (3,1,4)	-22.37356	14.21734
SARIMA $(2,0,1)(0,2,0)_{52}$	-91.4538	28.27603

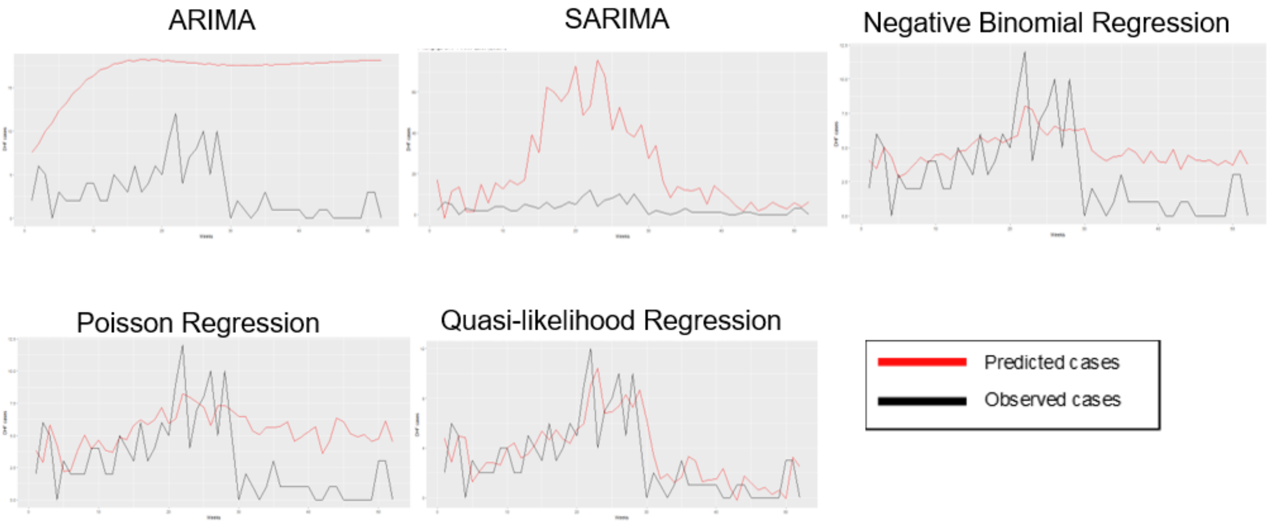

Figure 197: Plots between dengue cases and weeks, the black line represents the observed dengue cases, and the red line represents the predicted dengue cases of the best fit model of each technique over the test set data starting from January 2014 to December 2014.

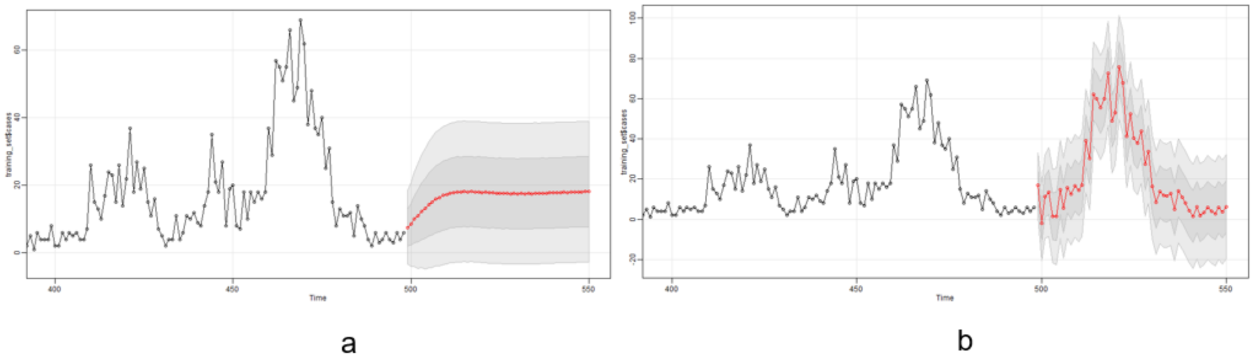

Figure 198: (a) Plot between dengue incidences over weekly time by the best model of ARIMA and (b) SARIMA time series analysis, the black line represents training set data starting from January 2012 to December 2013, and the red line represents the forecasted dengue incidences from January 2014 to December 2014.

Table 66: Coefficients and significant values of best fit GLM models, Negative Binomial, Poisson and Quasi-likelihood regression model of Phang nga. The table summarizes coefficients of each independent variables which are composed in best fit model of each method. The significant of each variable is labelled by asterisks under the coefficients. The most important factor is marked as three asterisks which p-value ranges from 0 to 0.001. The second important factor is marked as two asterisks which p-value ranges from 0.001 to 0.01. The third important factor is marked as an asterisk which p-value ranges from 0.01 to 0.1. The least important is also marked as a dot which p-value ranges from 0.1 to 1.

**Independent variables**	**Lag**	**Coefficients/Significant**		
		NB	Poisson	Quasi
Intercept		-2.192837 .	-2.812501 ***	-7.0928877
Cases	1	0.035838 ***	0.029164 ***	0.5996685 ***
	2	0.022187 ***	0.013195 ***	0.2515243 ***
	3	0.004756	0.005070 **	0.0509073
Average Pressure	0			
	1			
	2			0.0008456
	3			
Minimum Temperature	0			
	1			
	2			
	3			
Maximum Temperature	0			
	1	0.040841		
	2			
	3			
Relative Humidity	0			
	1			
	2	0.022633	0.047301 ***	0.0581902
	3			
Precipitation	0			
	1		-0.001100	
	2		-0.011894 ***	
	3			
Vaporization	0		0.065597 ***	
	1			
	2			
	3	0.090573	0.074402 ***	0.6632063 *
Wind Direction	0			
	1			
	2			
	3			
Wind Power	0			
	1			0.0734004
	2			-0.1038835
	3			

Phatthalung

Phatthalung is located in southern region of Thailand at 7°37′N 100○05′E. Phatthalung covers an area of 3,424 km^2. Total population are 474,192 people. The density of population is 152.0 people per km^2. Weather in Phatthalung has tropical savanna climate under the South Asian monsoon system which has shorter winter period. Phatthalung has a dry season that runs from December through March and a wet season that covers the other eight months. The rainy season begins with the arrival of the southwest monsoon around mid-May. Precipitation occurs from mid-May to August. Rainfall are roughly 250 mm in October. Humidity presents in the range of 76-82 percent throughout the year. January is the highest sunshine hours

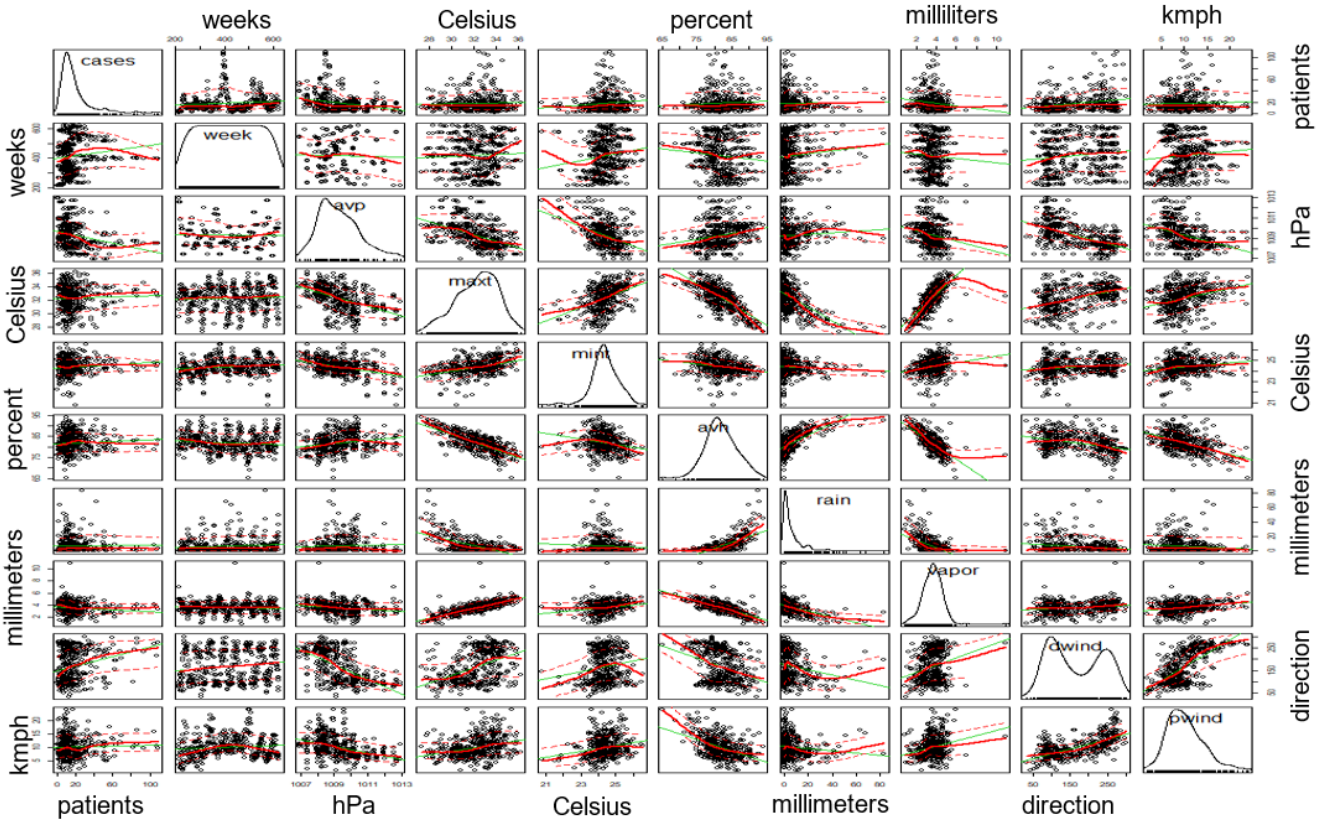

Figure 199: Scatter plot between dengue cases (cases) and selected independent variables, which are the weekly period starting from January 2001 – December 2013 (week), average pressure (avp), maximum temperature (maxt), minimum temperature (mint), average humidity (avh), precipitation (rain), vaporization of water (vapor), wind direction (dwind), and wind power (pwind). The plot visualizes pairwise hundred relationships of training set in Phatthalung.

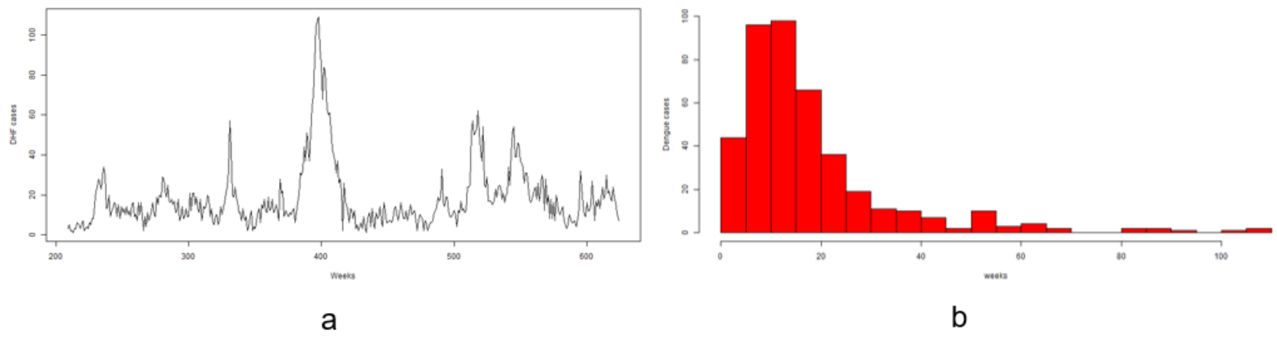

Figure 200: (a) Line plot between dengue incidences and weeks, the plot shows trends of dengue incidences in each year as stationary time series. (b) Histogram of dengue incidences in Phatthalung starting from January 2001 to December 2013 (624 weeks).

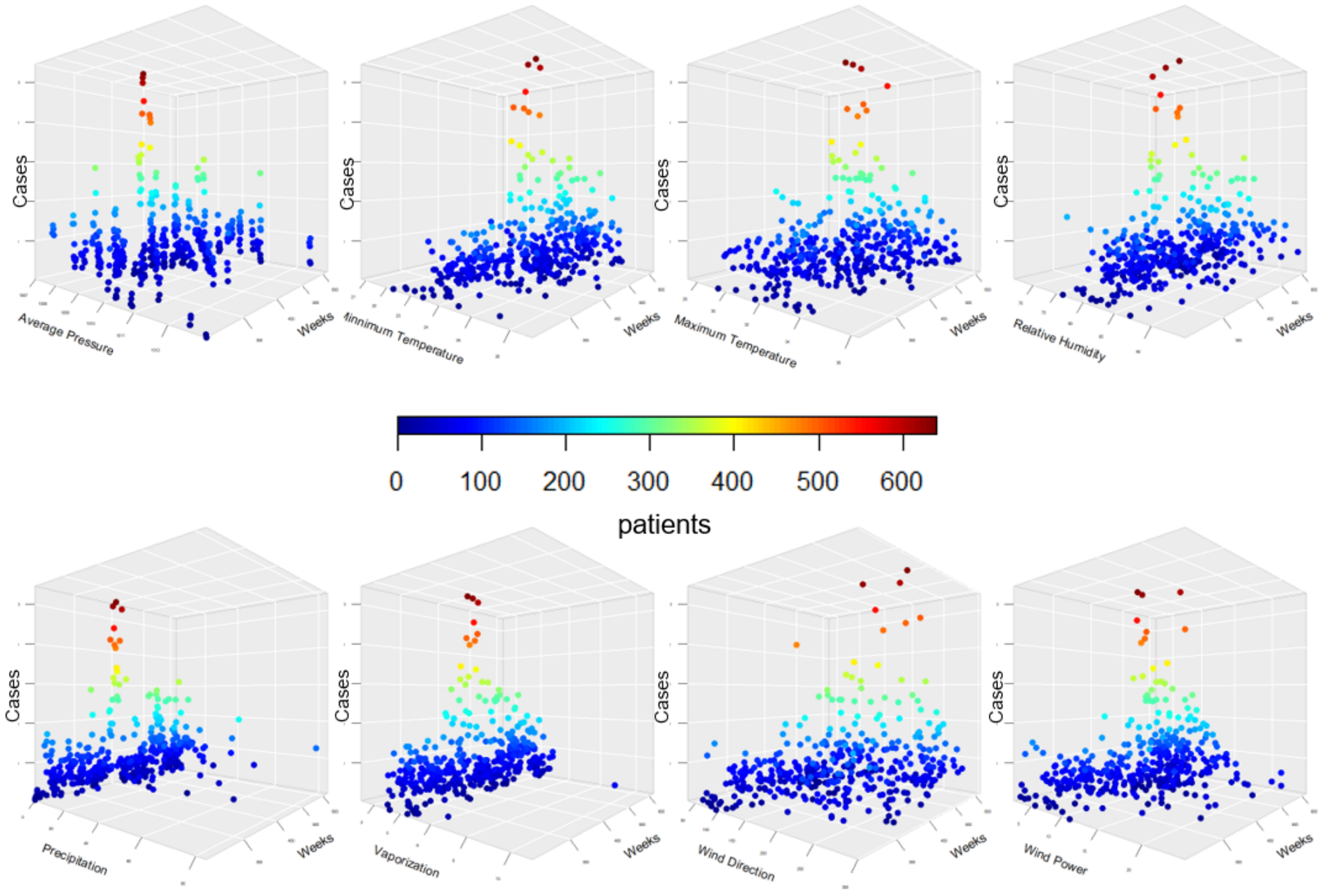

Figure 201: Three-dimensional scatter plot between dengue incidences and weather effects starting from January 2001 to December 2013 of Phatthalung.

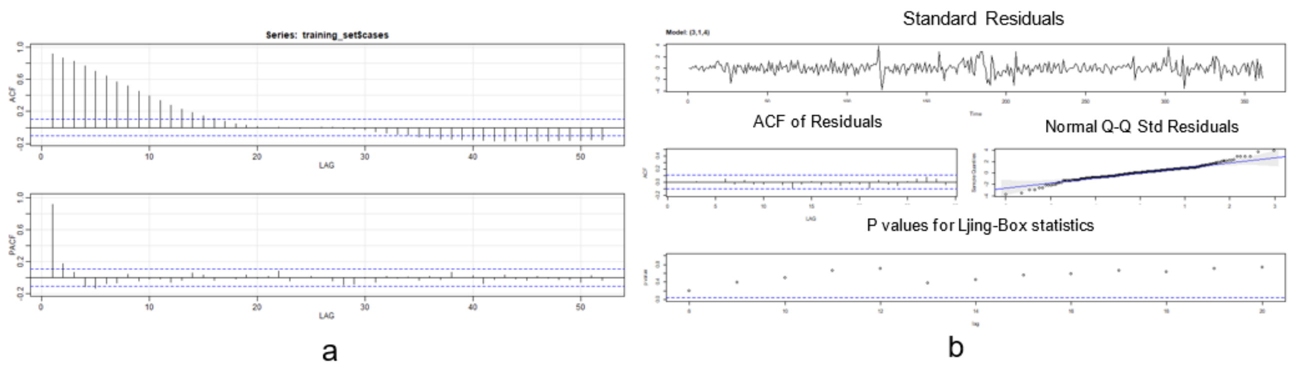

Figure 202: (a) Two plots between lag-time of dengue incidences and ACF and PACF relationship calculated from ARIMA model (b) Summary plots of time series analysis, multiple plots include the plot of predicted model over the time, the plot of ACF residual over lag-time of dengue incidences, residual Q-Q plot of standard residual, and p-value for Ljung-Box statistics of PACF relationship in Phatthalung over the training data starting from January 2001 to December 2013.

For Phatthalung, the best model is based on negative binomial regression method. The correlation coefficient on the test set in 2014 is 0.434 (95%CI: 0.3324, 0.5356). The significant of the variables associated with p-value statistical calculation are shown in Table 69. The best model of Phatthalung uses 8 variables. The most significant variables are 1-week-lag cases, following by 3-week-lag wind direction and 1-week-lag wind power. Other variables which have less significant are, 3-week-lag relative humidity, 2-week-lag and 3-week-lag precipitation, and current week wind direction. Time series methods by ARIMA and SARIMA yield the correlation coefficient of -0.4666963 and -3.033398 respectively.

Table 67: Comparison table of all methods by the highest correlation coefficient (R^2) and the lowest prediction error (RMSE) in phatthalung.

Methods	R-squared (R^2)	Root mean square error (RMSE)
Poisson Regression	0.4117486	5.194515
Negative Binomial Regression	0.4340985	5.09488
Quasi-likelihood Regression	0.2810719	5.74257
ARIMA (3,1,4)	-0.4666963	8.202262
SARIMA $(2,0,1)(0,2,0)_{52}$	-3.033398	13.60188

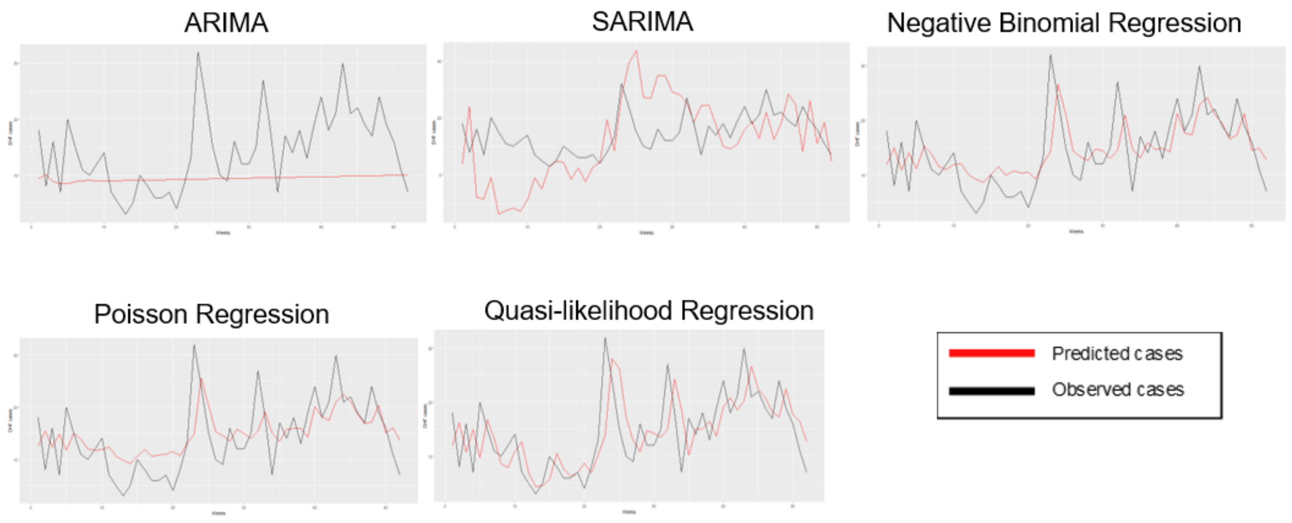

Figure 203: Plots between dengue cases and weeks, the black line represents the observed dengue cases, and the red line represents the predicted dengue cases of the best fit model of each technique over the test set data starting from January 2014 to December 2014.

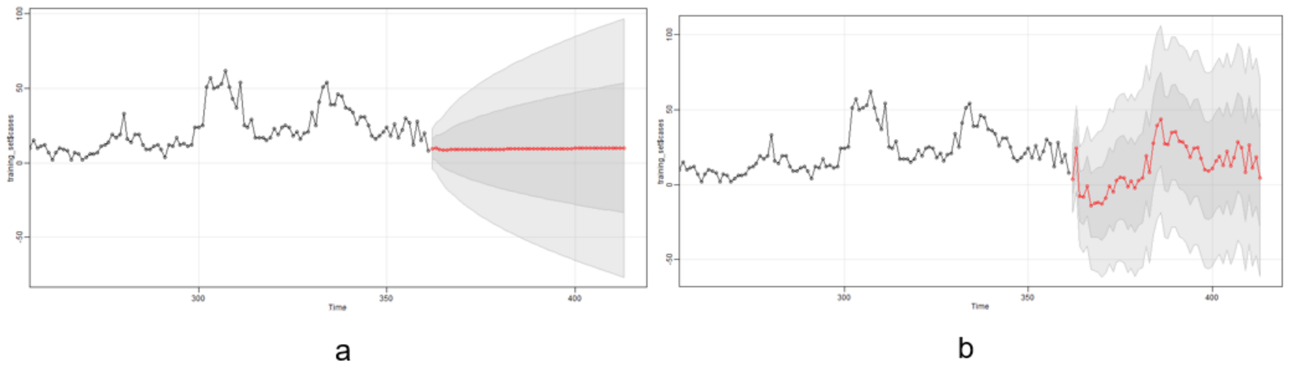

Figure 204: (a) Plot between dengue incidences over weekly time by the best model of ARIMA and (b) SARIMA time series analysis, the black line represents training set data starting from January 2012 to December 2013, and the red line represents the forecasted dengue incidences from January 2014 to December 2014.

Table 68: Coefficients and significant values of best fit GLM models, Negative Binomial, Poisson and Quasi-likelihood regression model of Phatthalung. The table summarizes coefficients of each independent variables which are composed in best fit model of each method. The significant of each variable is labelled by asterisks under the coefficients. The most important factor is marked as three asterisks which p-value ranges from 0 to 0.001. The second important factor is marked as two asterisks which p-value ranges from 0.001 to 0.01. The third important factor is marked as an asterisk which p-value ranges from 0.01 to 0.1. The least important is also marked as a dot which p-value ranges from 0.1 to 1.

**Independent variables**	**Lag**	**Coefficients/Significant**		
		NB	Poisson	Quasi
Intercept		1.6120682<br>*	1.4851051<br>***	-35.890559<br>.
Cases	1	0.0307435<br>***	0.0248341<br>***	0.7627448<br>***
	2			0.167501<br>**
	3			
Average Pressure	0			
	1			
	2			
	3			
Minimum Temperature	0			
	1			
	2			
	3			0.964733<br>.
Maximum Temperature	0			
	1			
	2			
	3			
Relative Humidity	0			
	1			
	2			
	3	0.0066339	0.0096666<br>*	0.157567
Precipitation	0			
	1			
	2	0.0029413	0.0033402<br>**	
	3	-0.0030721	-0.0037802<br>*	-0.077759<br>.
Vaporization	0	-0.0308667	-0.0472444<br>**	
	1			
	2			
	3			
Wind Direction	0	0.0009832	0.0009682<br>***	0.010211
	1			
	2			-0.014255<br>.
	3	0.0008463<br>*	0.0002936	
Wind Power	0			
	1	-0.0178325<br>*		
	2			0.197317
	3			

Phayao

Phayao is located in the northern region of Thailand at coordinate of 19°09′55″N 99°54′13″E. Phayao covers an area of 6,335 km^2. Total population are 484,454 people. The density of population is 76.0 people per km^2. Weather in Phayao has tropical savanna climate under the South Asian monsoon system Temperature is in the range from the low of 5.7°C in January to the high of 42.0°C in April. The monsoon season runs with the arrival of the southwest monsoon from late-April through early October. The humidity presents the average of 58-84 percent. Precipitation starts to rise from mid-May to August. The highest precipitation is in August of 204.0 mm. The longest sunshine hours are in March.

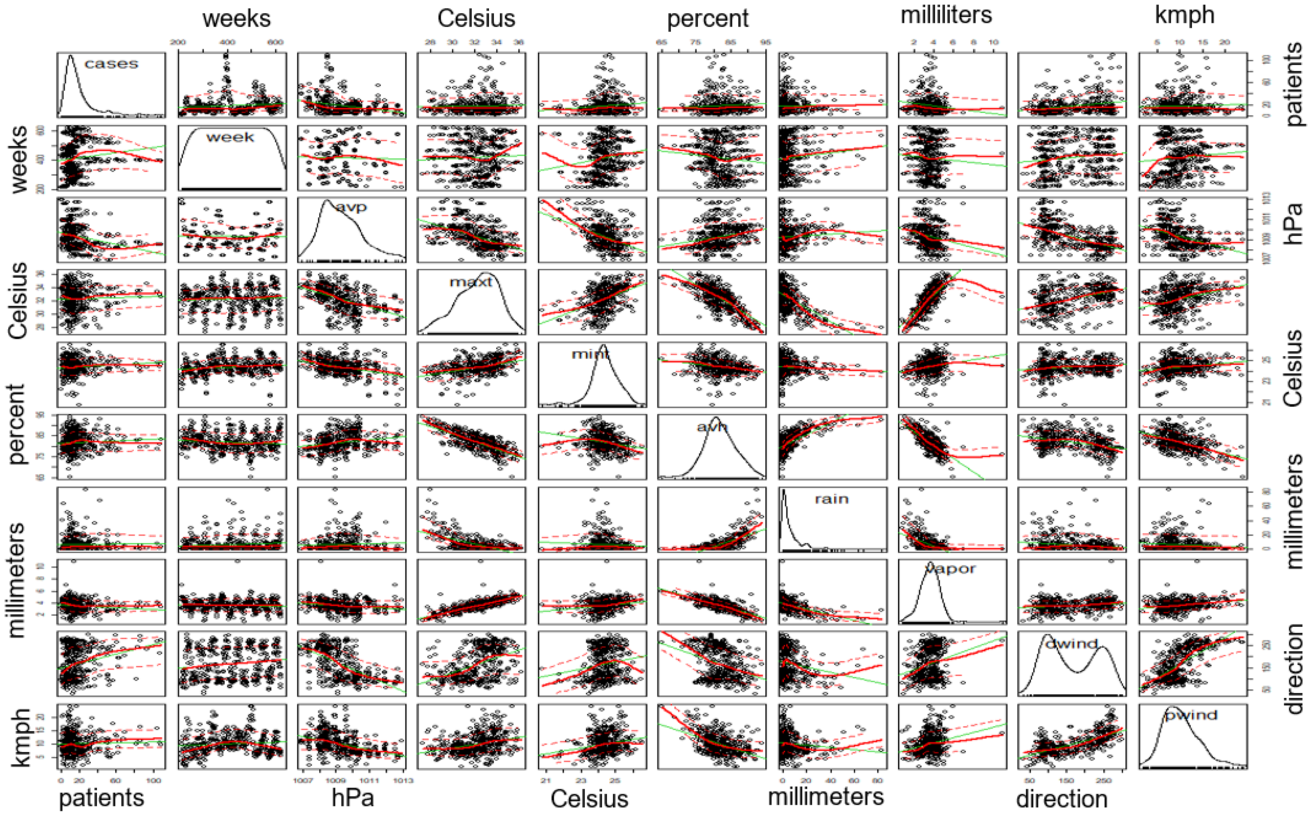

Figure 205: Scatter plot between dengue cases (cases) and selected independent variables, which are the weekly period starting from January 2001 – December 2013 (week), average pressure (avp), maximum temperature (maxt), minimum temperature (mint), average humidity (avh), precipitation (rain), vaporization of water (vapor), wind direction (dwind), and wind power (pwind). The plot visualizes pairwise hundred relationships of training set in Phayao.

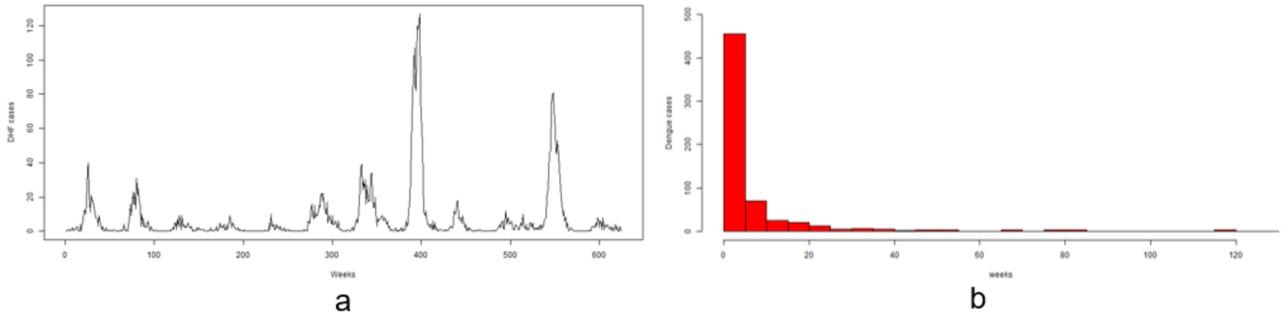

Figure 206: (a) Line plot between dengue incidences and weeks, the plot shows trends of dengue incidences in each year as stationary time series. (b) Histogram of dengue incidences in Phayao starting from January 2001 to December 2013 (624 weeks).

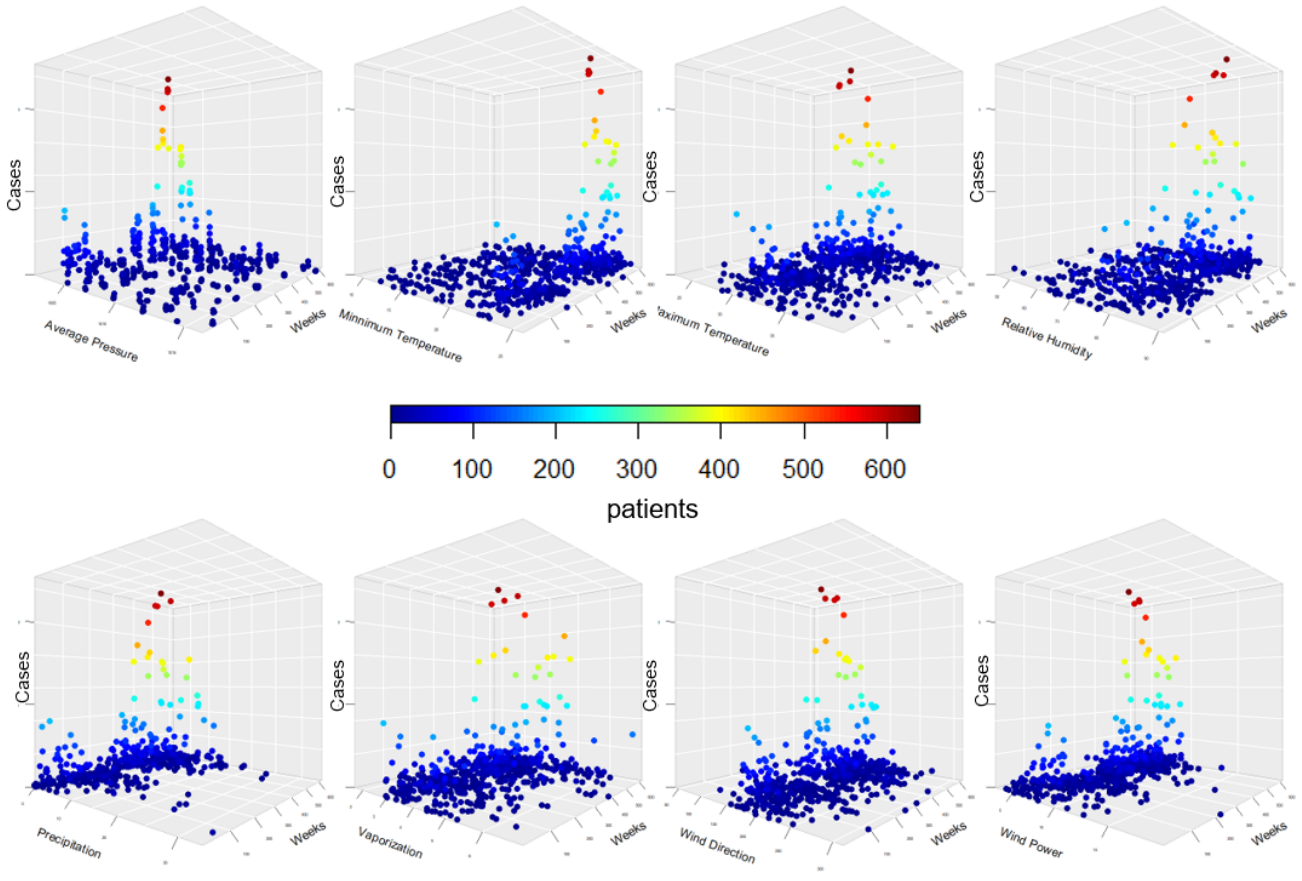

Figure 207: Three-dimensional scatter plot between dengue incidences and weather effects starting from January 2001 to December 2013 of Phayao.

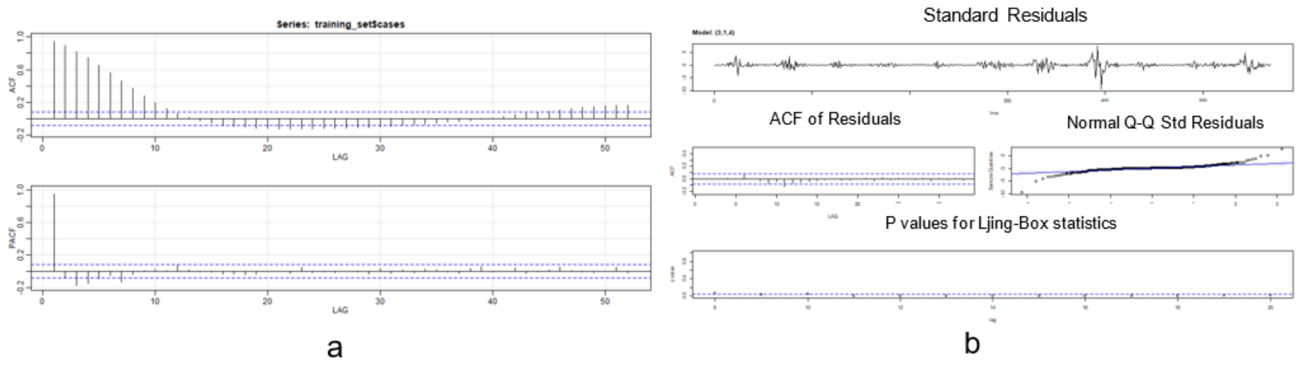

Figure 208: (a) Two plots between lag-time of dengue incidences and ACF and PACF relationship calculated from ARIMA model (b) Summary plots of time series analysis, multiple plots include the plot of predicted model over the time, the plot of ACF residual over lag-time of dengue incidences, residual Q-Q plot of standard residual, and p-value for Ljung-Box statistics of PACF relationship in Phayao over the training data starting from January 2001 to December 2013.

The best model of Phayao is based on negative binomial regression method. The correlation coefficient on the test set in 2014 is 0.473 (95%CI: 0.3813, 0.5646). The model consists of 6 variables, which are 2-week-lag cases, 2-week-lag relative, 1-week and 3-week-lag wind power, 3-week-lag wind direction and current week wind power. The variables are ranked by the significant as shown in table 71. Time series methods by ARIMA and SARIMA yield the correlation coefficient of -1.030557 and -194.4827 respectively.

Table 69: Comparison table of all methods by the highest correlation coefficient (R^2) and the lowest prediction error (RMSE) in Phayao.

**Methods**	**R-squared (R^2)**	**Root mean square error (RMSE)**
Poisson Regression	-0.08024975	2.205158
Negative Binomial Regression	0.4732057	1.53992
Quasi-likelihood Regression	0.3084113	1.764418
ARIMA (3,1,4)	-1.030557	3.023328
SARIMA $(2,0,1)(0,2,0)_{52}$	-194.4827	29.66414

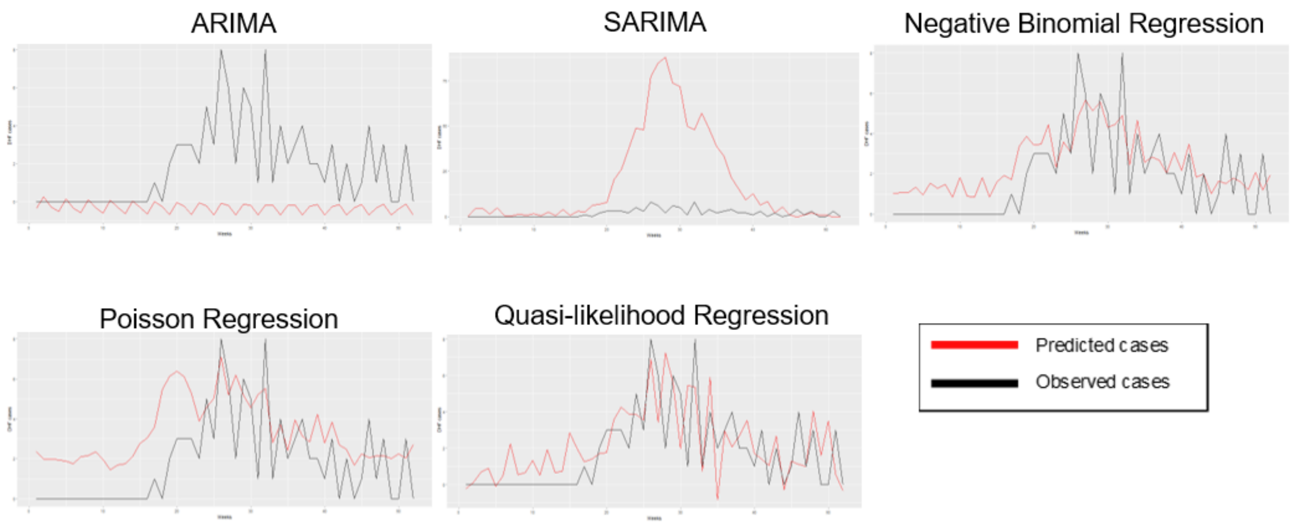

Figure 209: Plots between dengue cases and weeks, the black line represents the observed dengue cases, and the red line represents the predicted dengue cases of the best fit model of each technique over the test set data starting from January 2014 to December 2014.

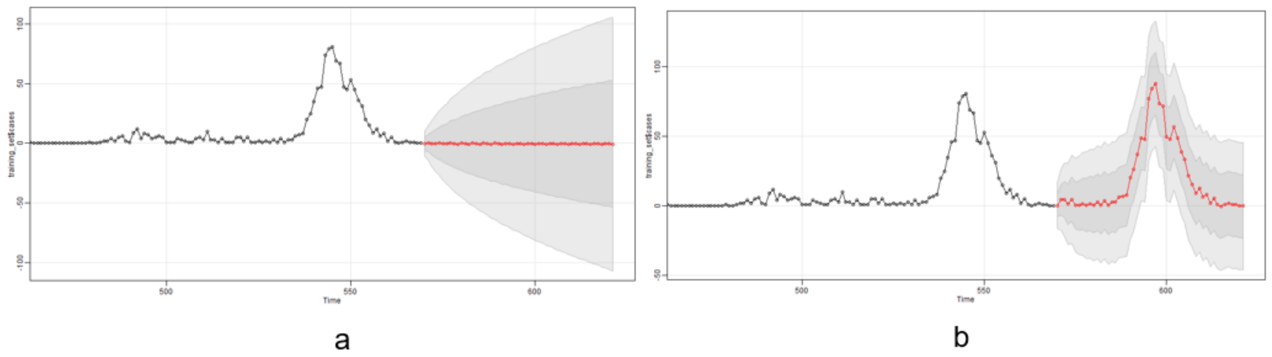

Figure 210: (a) Plot between dengue incidences over weekly time by the best model of ARIMA and (b) SARIMA time series analysis, the black line represents training set data starting from January 2012 to December 2013, and the red line represents the forecasted dengue incidences from January 2014 to December 2014.

Table 70: Coefficients and significant values of best fit GLM models, Negative Binomial, Poisson and Quasi-likelihood regression model of Phayao. The table summarizes coefficients of each independent variables which are composed in best fit model of each method. The significant of each variable is labelled by asterisks under the coefficients. The most important factor is marked as three asterisks which p-value ranges from 0 to 0.001. The second important factor is marked as two asterisks which p-value ranges from 0.001 to 0.01. The third important factor is marked as an asterisk which p-value ranges from 0.01 to 0.1. The least important is also marked as a dot which p-value ranges from 0.1 to 1.

**Independent variables**	**Lag**	**Coefficients/Significant**		
		NB	Poisson	Quasi
Intercept		-3.588155 ***	-4.6250497 ***	-0.3307058
Cases	1			
	2	0.057731 ***	0.0291438 ***	0.8923514 ***
	3			
Average Pressure	0			
	1			
	2			
	3			
Minimum Temperature	0			
	1			
	2			
	3			
Maximum Temperature	0			
	1			
	2			
	3			
Relative Humidity	0		0.0259572 ***	
	1			
	2	0.033233 ***	0.0140255 ***	
	3			
Precipitation	0		-0.0277190 ***	-0.1101286
	1			-0.0645447
	2			
	3			0.0199278
Vaporization	0			
	1			
	2			
	3			
Wind Direction	0			
	1		0.0993669 ***	-0.0112915
	2		0.1071520 ***	0.0007774
	3	-0.004408 **	0.1015528 ***	
Wind Power	0	0.055936 .	0.0764706 ***	0.4773530 **
	1	0.140398 ***		
	2			
	3	0.153204 ***		

Phetchabun

Phetchabun is a province located in the northern region of Thailand at 16°25′01″N 101°09′12″E. Phetchabun covers an area of 12,668 km^2. Total population are 995,807 people. The density of population is 79.0 people per km^2. Weather in Phetchabun has tropical savanna climate under the South Asian monsoon system Temperature is in the range from the low of 5.6°C in January to the high of 42.6°C in April. The rainy season runs with the arrival of the southwest monsoon from late-April through early October. The humidity presents the average of 63-85 percent. Precipitation starts to rise from mid-May to August. The highest precipitation is in September of 205.7 mm. The longest sunshine hours are in March.

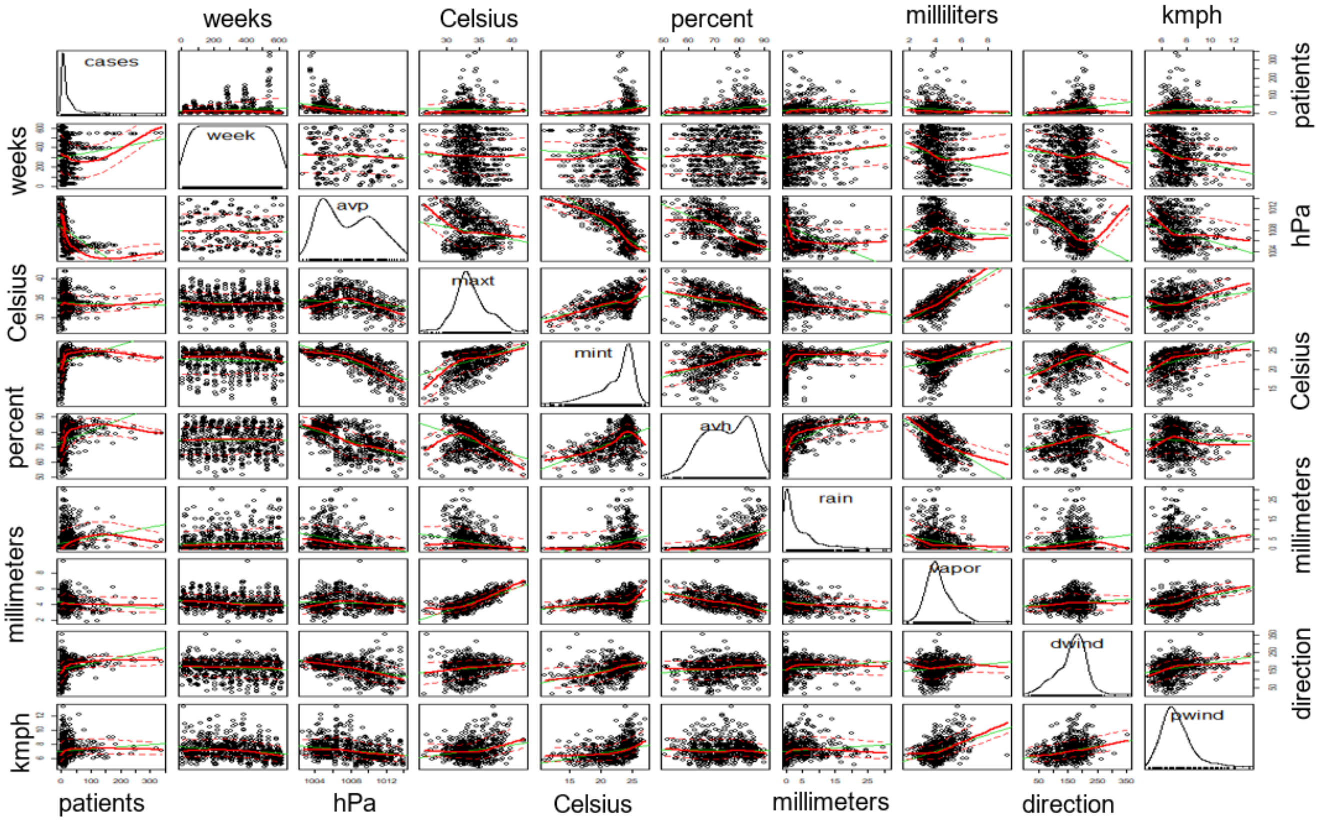

Figure 211: Scatter plot between dengue cases (cases) and selected independent variables, which are the weekly period starting from January 2001 – December 2013 (week), average pressure (avp), maximum temperature (maxt), minimum temperature (mint), average humidity (avh), precipitation (rain), vaporization of water (vapor), wind direction (dwind), and wind power (pwind). The plot visualizes pairwise hundred relationships of training set in Phetchabun.

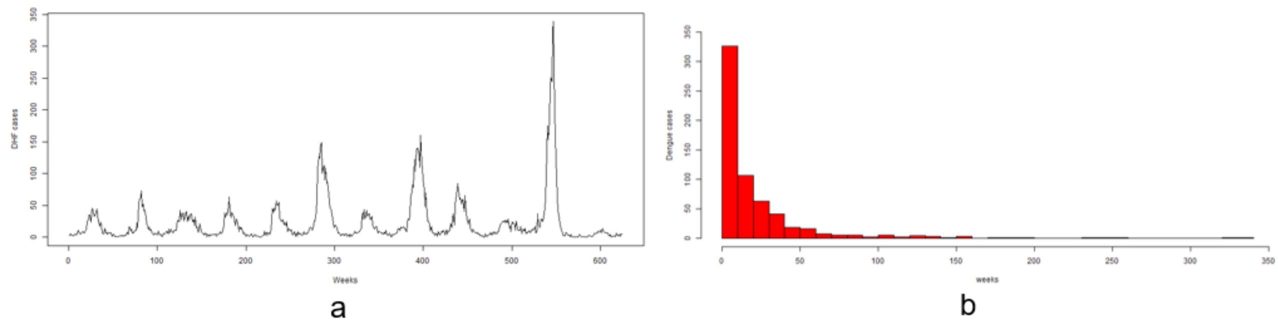

Figure 212: (a) Line plot between dengue incidences and weeks, the plot shows trends of dengue incidences in each year as stationary time series. (b) Histogram of dengue incidences in Phetchabun starting from January 2001 to December 2013 (624 weeks).

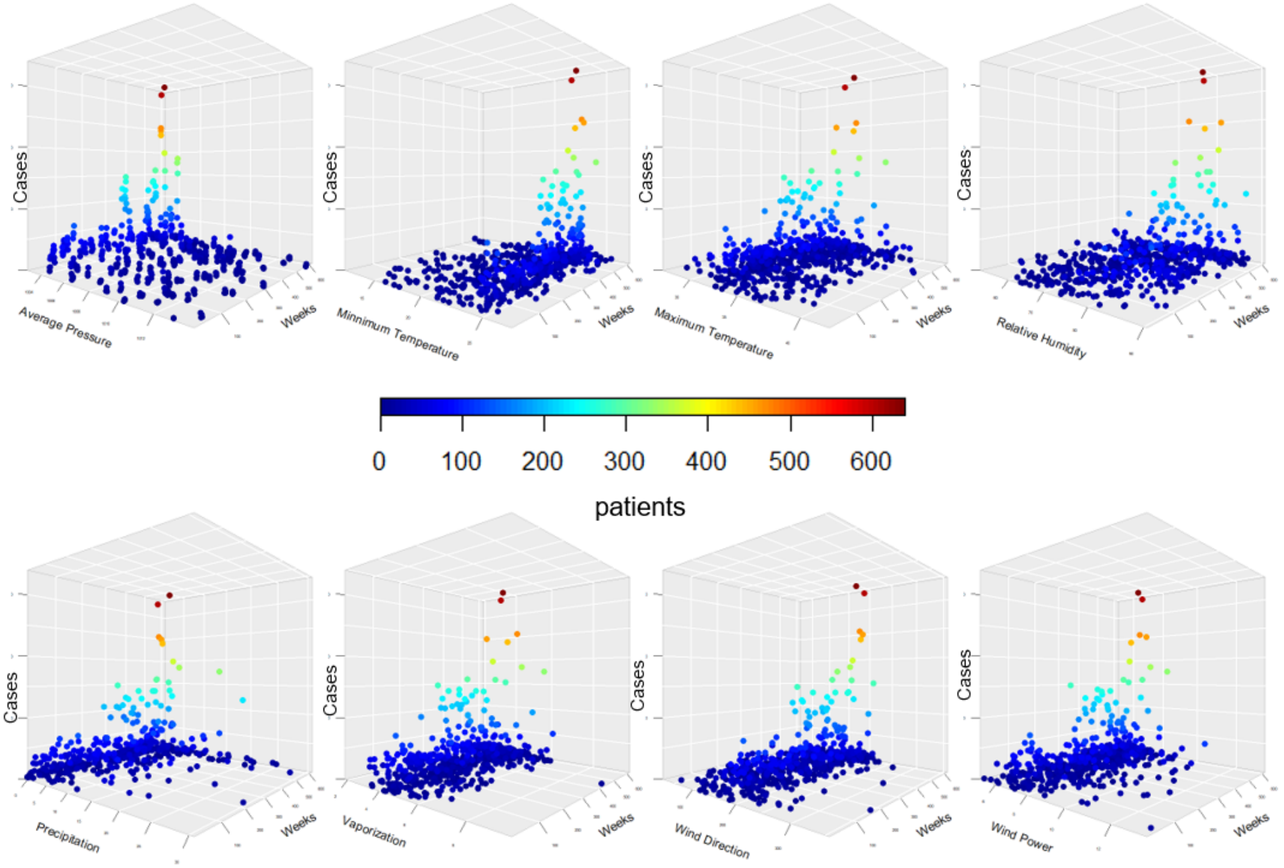

Figure 213: Three-dimensional scatter plot between dengue incidences and weather effects starting from January 2001 to December 2013 of Phetchabun.

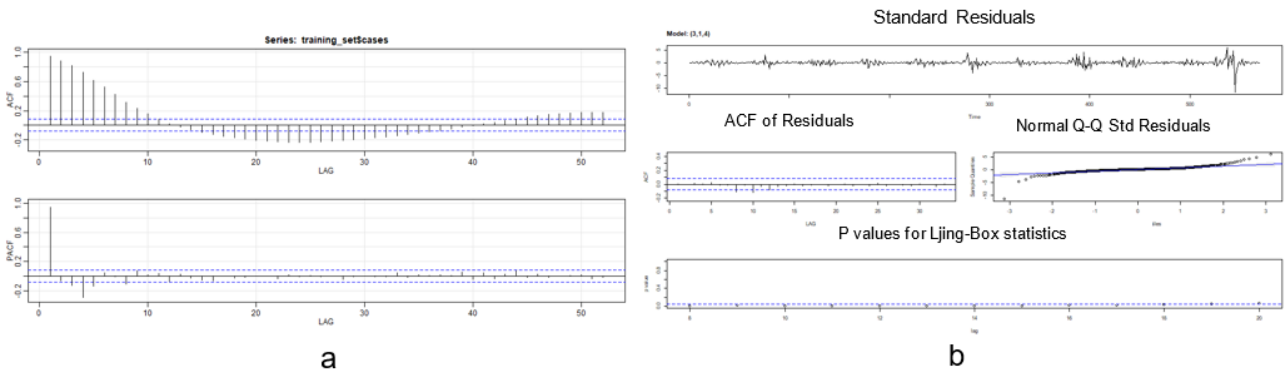

Figure 214: (a) Two plots between lag-time of dengue incidences and ACF and PACF relationship calculated from ARIMA model (b) Summary plots of time series analysis, multiple plots include the plot of predicted model over the time, the plot of ACF residual over lag-time of dengue incidences, residual Q-Q plot of standard residual, and p-value for Ljung-Box statistics of PACF relationship in Phetchabun over the training data starting from January 2001 to December 2013.

For Phetchabun, the best model is based on quasi-likelihood method. The correlation coefficient on the test set in 2014 is 0.521 (95%CI: 0.3898, 0.6522). The significant of the variables associated with p-value statistical calculation are shown in Table PCB2. The model of uses 7 variables. The most significant variable is 1-week-lag cases. Other variables which have less significant are, 3-week-lag average pressure, 2-week-lag minimum temperature, 2-week-lag precipitation, 1-week-lag and 3-week-lag wind direction, and 2-week-lag wind power. Time series methods by ARIMA and SARIMA yield the correlation coefficient of -1.383239 and -1018.971 respectively.

Table 71: Comparison table of all methods by the highest correlation coefficient (R^2) and the lowest prediction error (RMSE) in phetchabun.

**Methods**	**R-squared (R^2)**	**Root mean square error (RMSE)**
Poisson Regression	-3.781279	7.595515
Negative Binomial Regression	-0.7142792	4.548058
Quasi-likelihood Regression	0.5212401	2.4035
ARIMA (3,1,4)	-1.383239	5.362521
SARIMA $(2,0,1)(0,2,0)_{52}$	-1018.971	110.9377

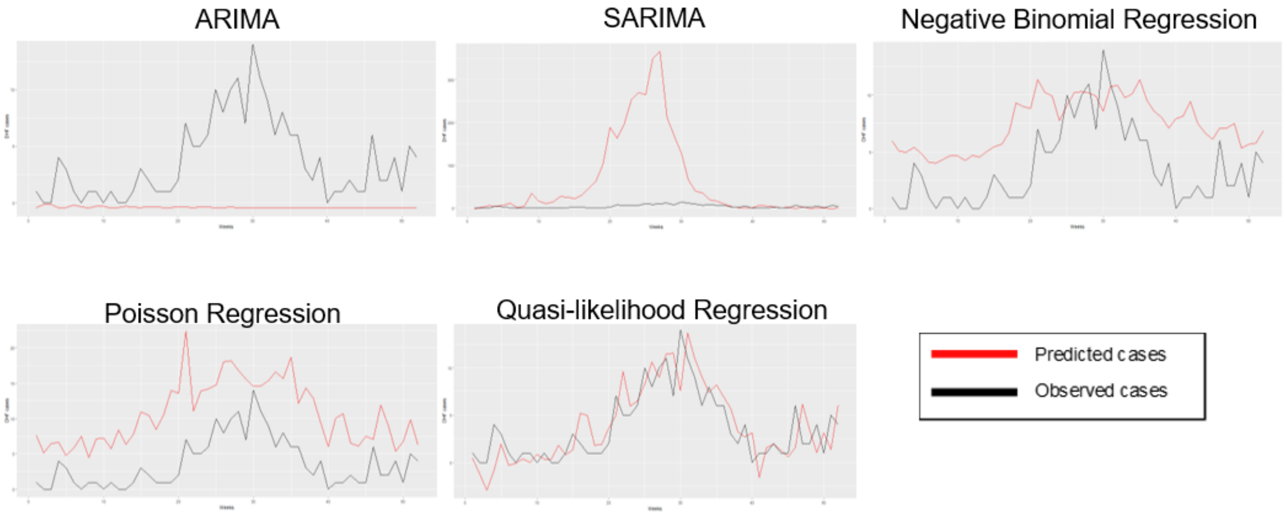

Figure 215: Plots between dengue cases and weeks, the black line represents the observed dengue cases, and the red line represents the predicted dengue cases of the best fit model of each technique over the test set data starting from January 2014 to December 2014.

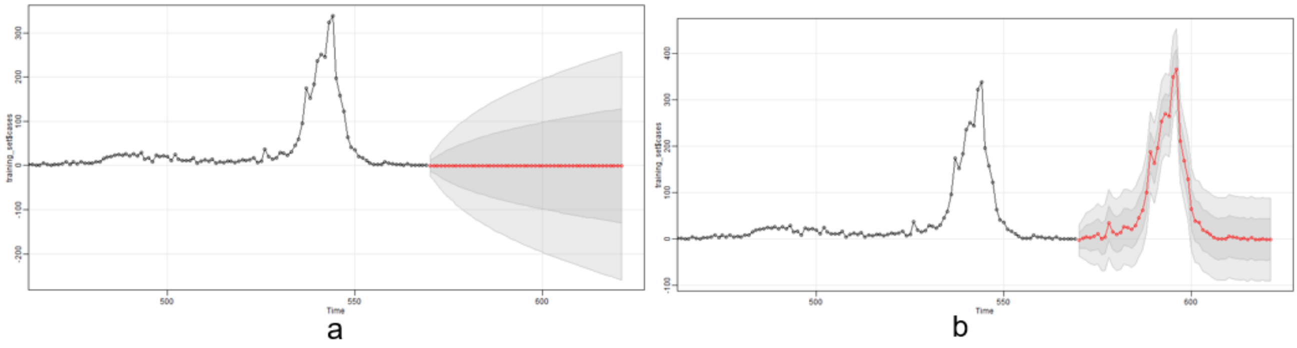

Figure 216: (a) Plot between dengue incidences over weekly time by the best model of ARIMA and (b) SARIMA time series analysis, the black line represents training set data starting from January 2012 to December 2013, and the red line represents the forecasted dengue incidences from January 2014 to December 2014.

Table 72: Coefficients and significant values of best fit GLM models, Negative Binomial, Poisson and Quasi-likelihood regression model of Phetchabun. The table summarizes coefficients of each independent variables which are composed in best fit model of each method. The significant of each variable is labelled by asterisks under the coefficients. The most important factor is marked as three asterisks which p-value ranges from 0 to 0.001. The second important factor is marked as two asterisks which p-value ranges from 0.001 to 0.01. The third important factor is marked as an asterisk which p-value ranges from 0.01 to 0.1. The least important is also marked as a dot which p-value ranges from 0.1 to 1.

**Independent variables**	**Lag**	**Coefficients/Significant**		
		NB	Poisson	Quasi
Intercept		-1.7347579 ***	-2.1084141 ***	-151.3
Cases	1	0.0228344 ***	0.0086927 ***	0.93900 ***
	2			
	3		0.0027256 ***	
Average Pressure	0			
	1			
	2			
	3			0.1385
Minimum Temperature	0			
	1			
	2			0.3097
	3			
Maximum Temperature	0			
	1			
	2			
	3			
Relative Humidity	0			
	1			
	2			
	3	0.0259492 ***	0.0262438 ***	
Precipitation	0			
	1			
	2			-0.009851
	3			
Vaporization	0			
	1			
	2			
	3			
Wind Direction	0	0.0051725 ***		
	1			0.01568
	2			
	3			-0.003311
Wind Power	0	0.0625097 *	0.333331 ***	
	1	0.0699793 *	0.0575874 ***	
	2	0.0685437 *	0.0706242 ***	0.5795
	3	0.0646591 *	0.0945012 ***	

Phetchaburi

Phetchaburi is a province located in southern Thailand at 13°06′43″N 99°56′45″E. Phetchaburi covers an area of 6,225 km^2. Total population are 474,192 people. The density of population is 76.0 people per km^2. Weather in Phetchaburi has tropical savanna climate under the South Asian monsoon system which has shorter winter period. Temperature in Phetchaburi has an average annual high of 32°C and an annual low of 12.4°C. Phetchaburi has a dry season that runs from December through March and a wet season that covers the other eight months. The rainy season begins with the arrival of the southwest monsoon around mid-May. Precipitation occurs from mid-May to August. Rainfall are roughly 278.4 mm in October. Humidity presents in the range of 76-82 percent throughout the year. March is the highest sunshine hours.

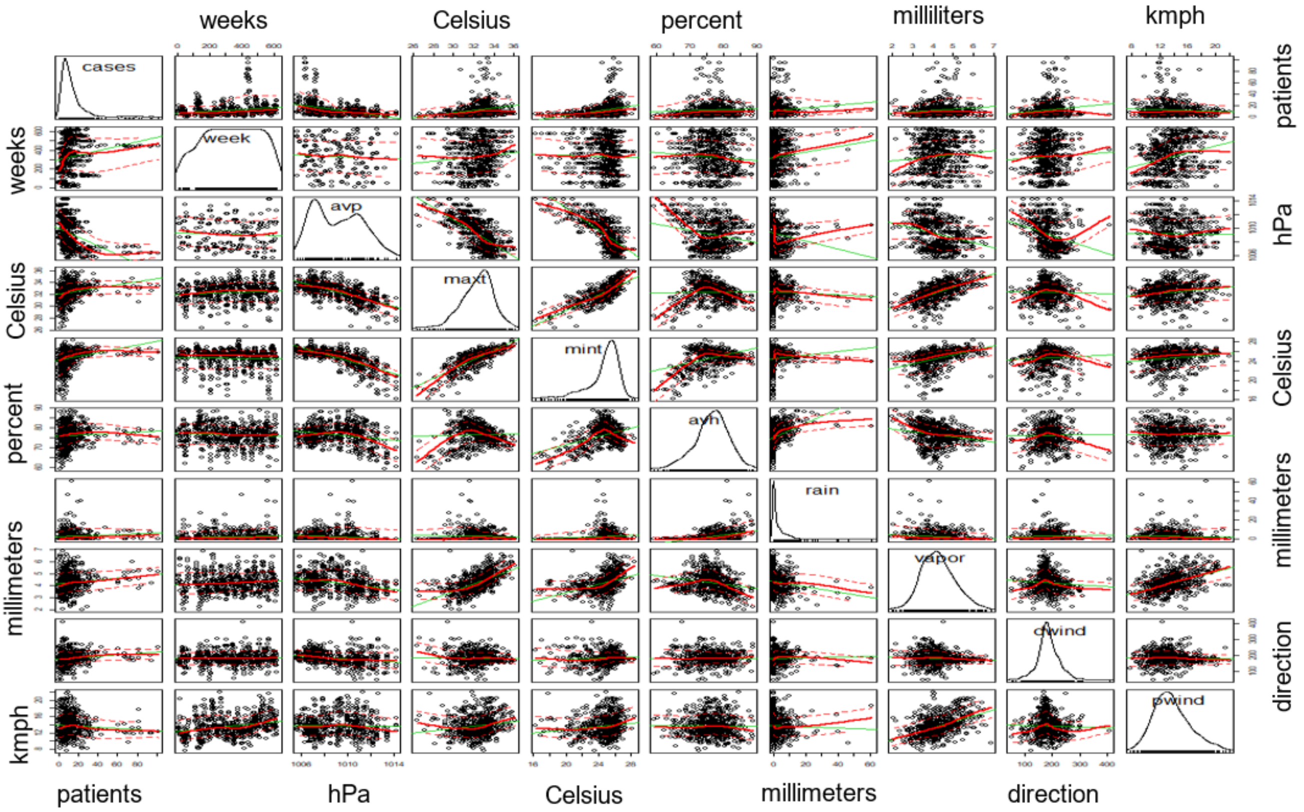

Figure 217: Scatter plot between dengue cases (cases) and selected independent variables, which are the weekly period starting from January 2001 – December 2013 (week), average pressure (avp), maximum temperature (maxt), minimum temperature (mint), average humidity (avh), precipitation (rain), vaporization of water (vapor), wind direction (dwind), and wind power (pwind). The plot visualizes pairwise hundred relationships of training set in Phetchaburi.

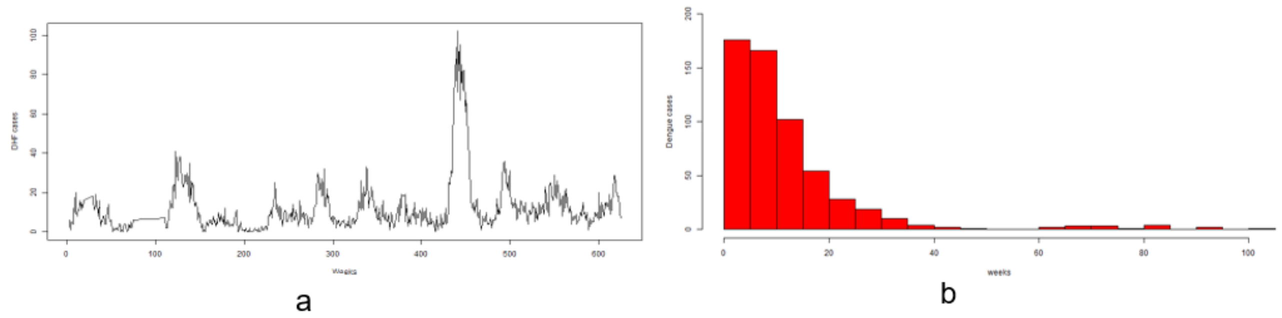

Figure 218: (a) Line plot between dengue incidences and weeks, the plot shows trends of dengue incidences in each year as stationary time series. (b) Histogram of dengue incidences in Phetchaburi starting from January 2001 to December 2013 (624 weeks).

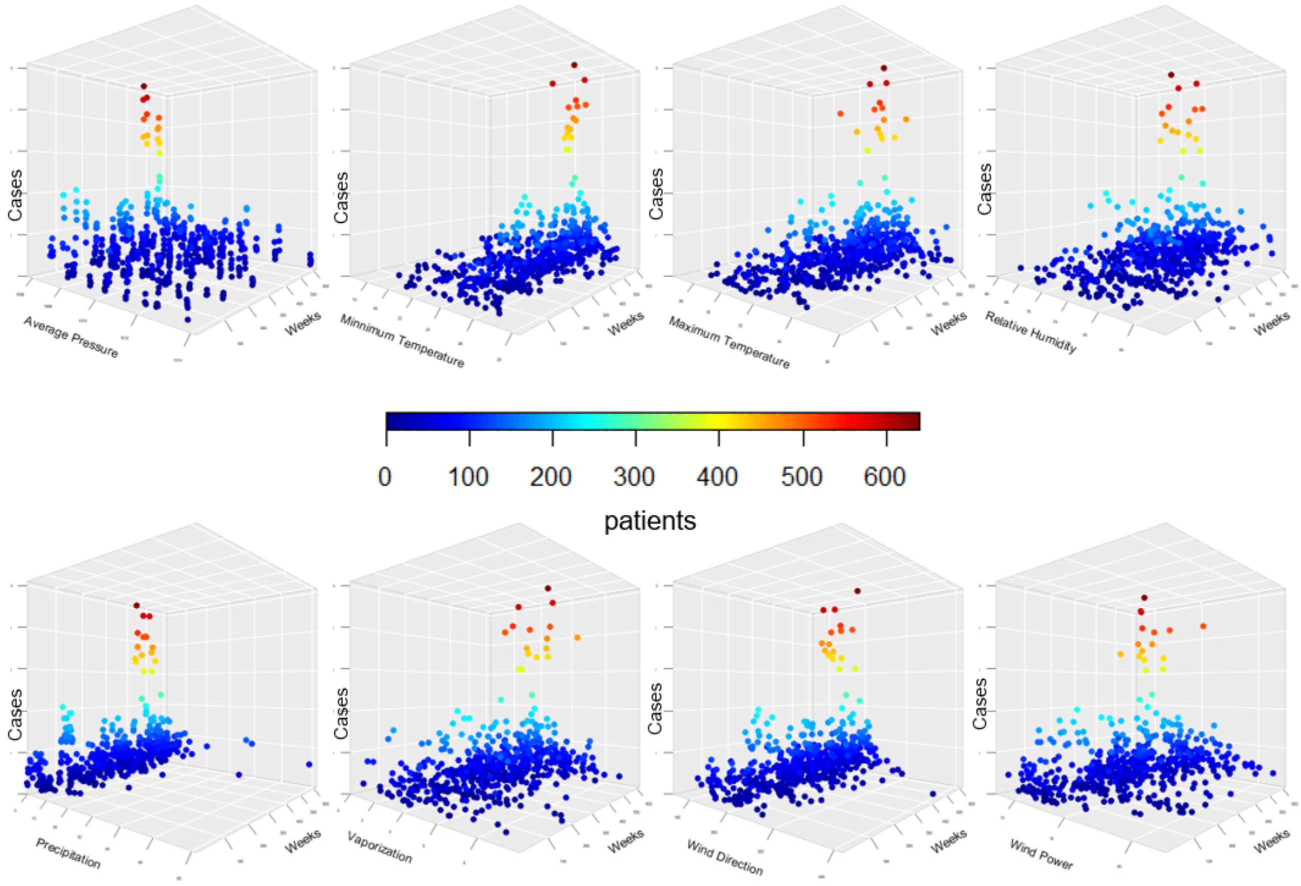

Figure 219: Three-dimensional scatter plot between dengue incidences and weather effects starting from January 2001 to December 2013 of Phetchaburi.

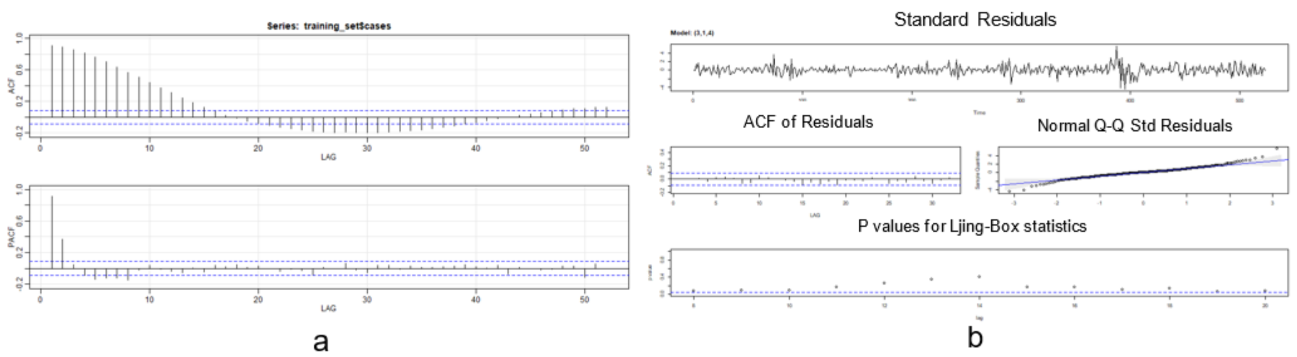

Figure 220: (a) Two plots between lag-time of dengue incidences and ACF and PACF relationship calculated from ARIMA model (b) Summary plots of time series analysis, multiple plots include the plot of predicted model over the time, the plot of ACF residual over lag-time of dengue incidences, residual Q-Q plot of standard residual, and p-value for Ljung-Box statistics of PACF relationship in Phetchaburi over the training data starting from January 2001 to December 2013.

For Phetchaburi, the best model is based on quasi-likelihood method. The correlation coefficient on the test set in 2014 is 0.524 (95%CI: 0.4320, 0.6159). The significant of the variables associated with p-value statistical calculation are shown in Table 73. The model uses 9 variables. The most significant variables are 1-week-lag cases, 3-week-lag cases and current week minimum temperature. Other variables are, 1-week-lag average pressure, 2-week-lag and 3-week-lag minimum temperature, current week, 1-week-lag and 2-week-lag precipitation. Time series methods by ARIMA and SARIMA yield the correlation coefficient of -2.876896 and -2.048135 respectively.

Table 73: Comparison table of all methods by the highest correlation coefficient (R^2) and the lowest prediction error (RMSE) in Phetchaburi.

**Methods**	**R-squared (R^2)**	**Root mean square error (RMSE)**
Poisson Regression	0.4602962	4.404316
Negative Binomial Regression	0.5006171	4.236601
Quasi-likelihood Regression	0.5236738	4.137642
ARIMA (3,1,4)	-2.876896	11.80437
SARIMA $(2,0,1)(0,2,0)_{52}$	-2.048135	10.46689

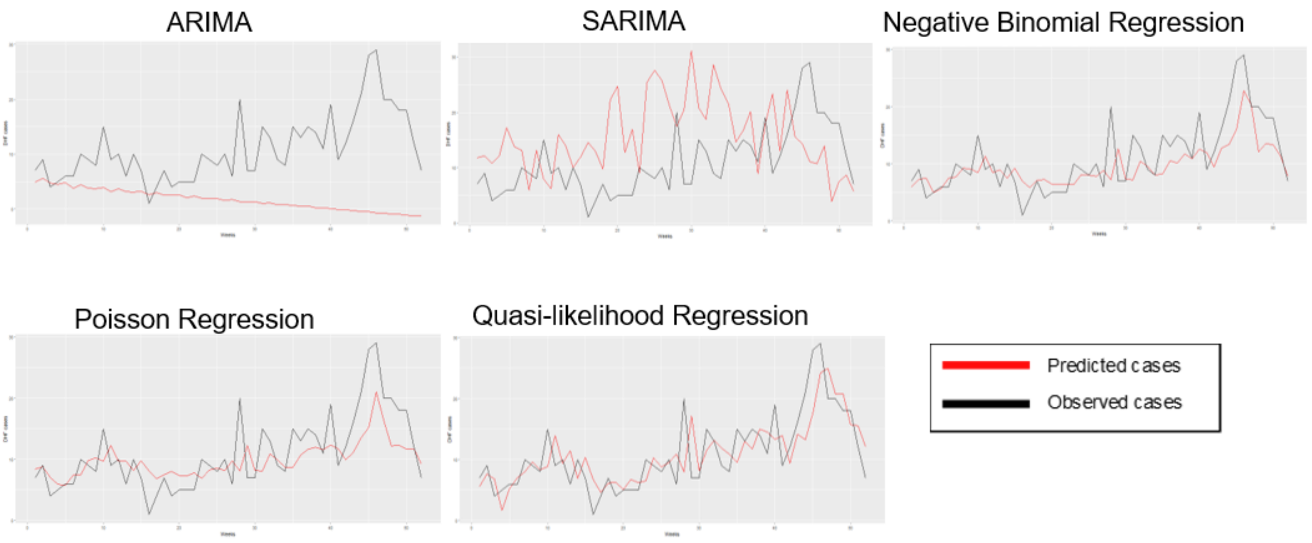

Figure 221: Plots between dengue cases and weeks, the black line represents the observed dengue cases, and the red line represents the predicted dengue cases of the best fit model of each technique over the test set data starting from January 2014 to December 2014.

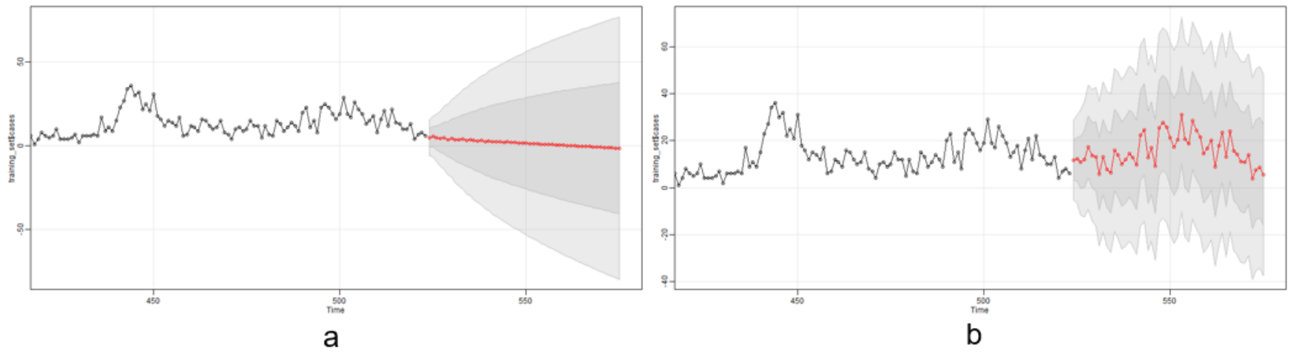

Figure 222: (a) Plot between dengue incidences over weekly time by the best model of ARIMA and (b) SARIMA time series analysis, the black line represents training set data starting from January 2012 to December 2013, and the red line represents the forecasted dengue incidences from January 2014 to December 2014.

Table 74: Coefficients and significant values of best fit GLM models, Negative Binomial, Poisson and Quasi-likelihood regression model of Phetchaburi. The table summarizes coefficients of each independent variables which are composed in best fit model of each method. The significant of each variable is labelled by asterisks under the coefficients. The most important factor is marked as three asterisks which p-value ranges from 0 to 0.001. The second important factor is marked as two asterisks which p-value ranges from 0.001 to 0.01. The third important factor is marked as an asterisk which p-value ranges from 0.01 to 0.1. The least important is also marked as a dot which p-value ranges from 0.1 to 1.

**Independent variables**	**Lag**	**Coefficients/Significant**		
		NB	Poisson	Quasi
Intercept		0.377717	-0.1880547	253.39608
Cases	1	0.044445 ***	0.0310893 ***	0.7068400 ***
	2			
	3			0.210810 ***
Average Pressure	0			
	1			-0.25197
	2			
	3			
Minimum Temperature	0			0.5100700 *
	1			
	2			-0.33254
	3			-0.09864
Maximum Temperature	0			
	1			
	2			
	3			
Relative Humidity	0			
	1	0.016224 **	0.0108146 ***	
	2			
	3		0.0132252 ***	
Precipitation	0	0.005620	0.0032883	0.02830
	1	-0.004824	-0.0022353	-0.07872
	2			0.03176
	3	0.006726		
Vaporization	0			
	1			
	2			
	3			
Wind Direction	0			
	1			
	2		0.0015824 ***	
	3			
Wind Power	0	0.006131		
	1			
	2			
	3			

Phichit

Phichit is a province located in northern region of Thailand at coordinate of 16°26′35″N 100°20′48″E. Phichit covers an area of 4,531 km^2. Total population are 547,543 people. The density of population is 120.8 people per km^2. Weather in Phichit follows tropical savanna climate under the South Asian monsoon system. Temperature is in the range from the low in January to the high in April. The rainy season runs from May through October. The humidity presents the average of 62-81 percent throughout the year. Precipitation starts to rise from mid-May to August. The highest precipitation is in August. The longest sunshine hours are in January.

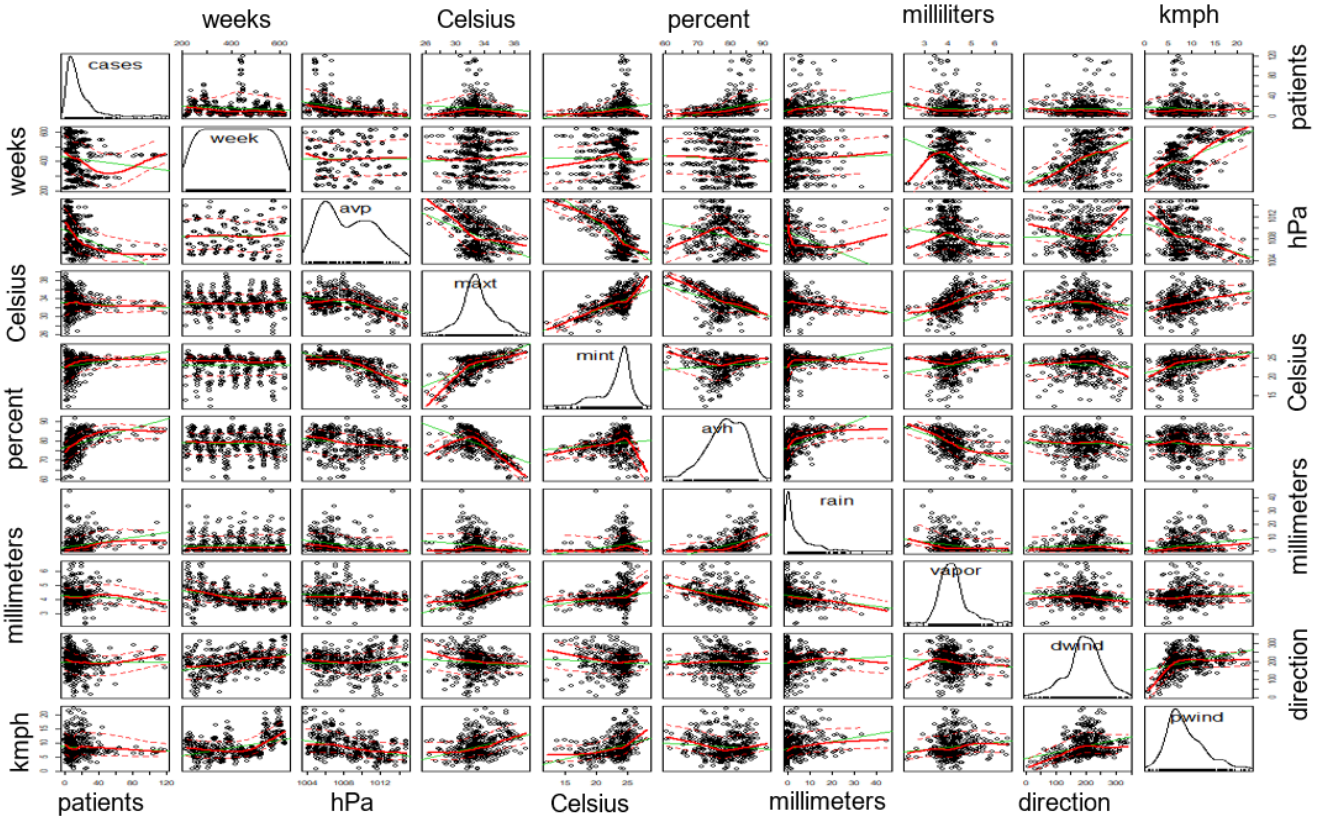

Figure 223: Scatter plot between dengue cases (cases) and selected independent variables, which are the weekly period starting from January 2001 – December 2013 (week), average pressure (avp), maximum temperature (maxt), minimum temperature (mint), average humidity (avh), precipitation (rain), vaporization of water (vapor), wind direction (dwind), and wind power (pwind). The plot visualizes pairwise hundred relationships of training set in Phichit.

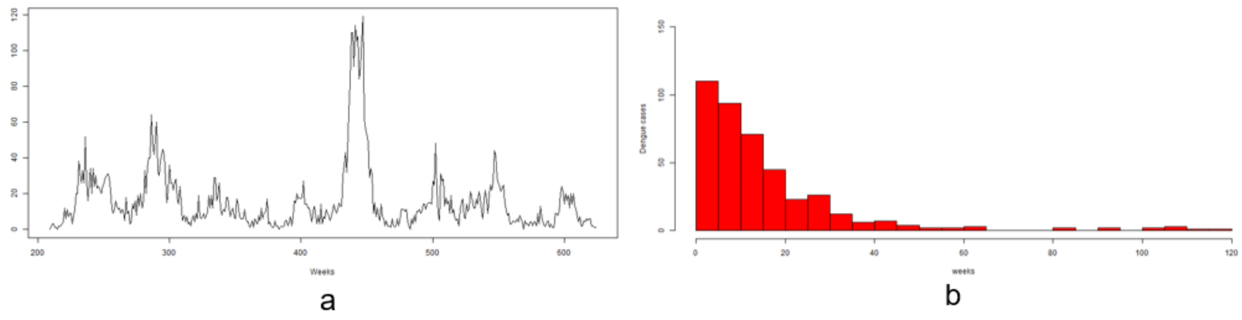

Figure 224: (a) Line plot between dengue incidences and weeks, the plot shows trends of dengue incidences in each year as stationary time series. (b) Histogram of dengue incidences in Phichit starting from January 2001 to December 2013 (624 weeks).

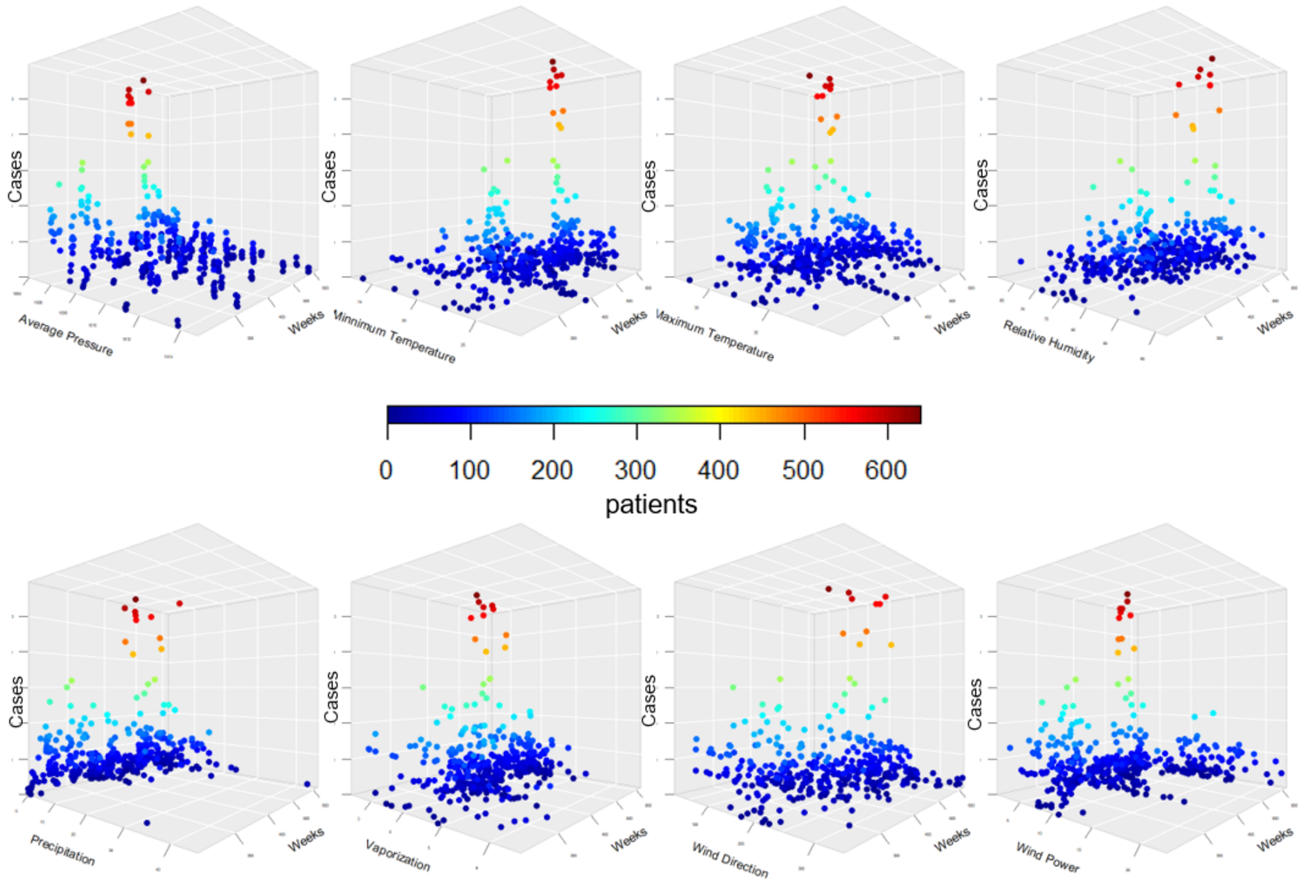

Figure 225: Three-dimensional scatter plot between dengue incidences and weather effects starting from January 2001 to December 2013 of Phichit.

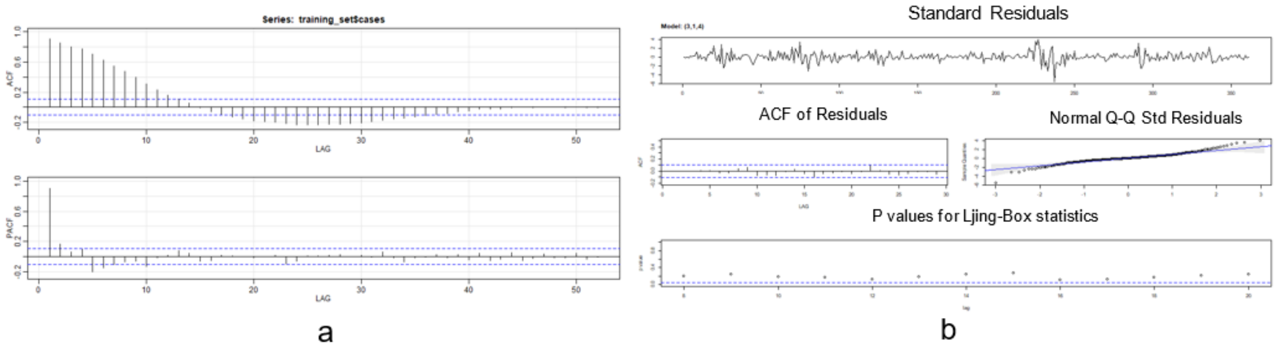

Figure 226: (a) Two plots between lag-time of dengue incidences and ACF and PACF relationship calculated from ARIMA model (b) Summary plots of time series analysis, multiple plots include the plot of predicted model over the time, the plot of ACF residual over lag-time of dengue incidences, residual Q-Q plot of standard residual, and p-value for Ljung-Box statistics of PACF relationship in Phichit over the training data starting from January 2001 to December 2013.

For Phichit, the best model is based on Poisson regression method. The correlation coefficient on the test set in 2014 is 0.715 (95%CI: 0.6376, 0.7924). The best model consists of 8 variables. The most significant variables are current week average pressure, 2-week-lag average pressure, current week and 2-week-lag maximum temperature, 2-week-lag vaporization, current week and 2-week-lag wind power. Other variables which have less significant is current week wind direction. Time series methods by ARIMA and SARIMA yield the correlation coefficient of -1.078646 and -1.9310470 respectively.

Table 75: Comparison table of all methods by the highest correlation coefficient (R^2) and the lowest prediction error (RMSE) in Phichit.

**Methods**	**R-squared (R^2)**	**Root mean square error (RMSE)**
Poisson Regression	0.7154899	3.407421
Negative Binomial Regression	0.4434865	4.765569
Quasi-likelihood Regression	0.6943876	3.531526
ARIMA (3,1,4)	-1.078646	9.21016
SARIMA $(2,0,1)(0,2,0)_{52}$	-1.931047	10.93675

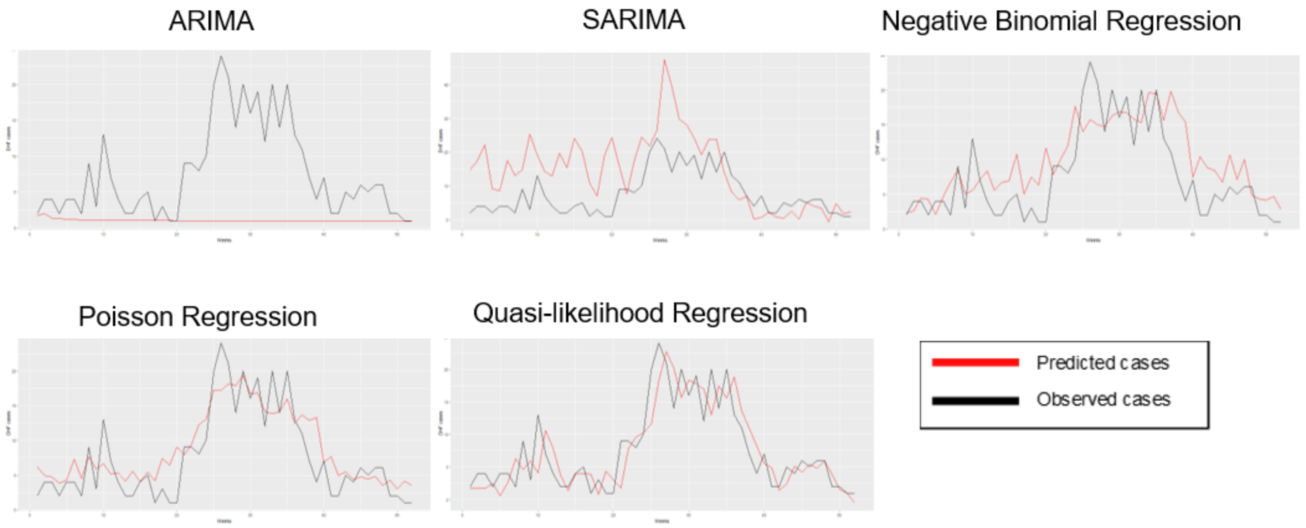

Figure 227: Plots between dengue cases and weeks, the black line represents the observed dengue cases, and the red line represents the predicted dengue cases of the best fit model of each technique over the test set data starting from January 2014 to December 2014.

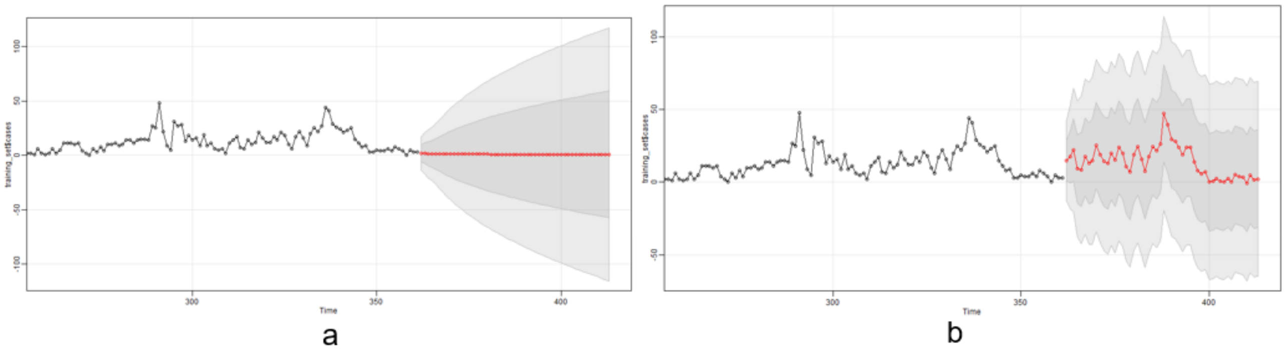

Figure 228: (a) Plot between dengue incidences over weekly time by the best model of ARIMA and (b) SARIMA time series analysis, the black line represents training set data starting from January 2012 to December 2013, and the red line represents the forecasted dengue incidences from January 2014 to December 2014.

Table 76: Coefficients and significant values of best fit GLM models, Negative Binomial, Poisson and Quasi-likelihood regression model of Phichit. The table summarizes coefficients of each independent variables which are composed in best fit model of each method. The significant of each variable is labelled by asterisks under the coefficients. The most important factor is marked as three asterisks which p-value ranges from 0 to 0.001. The second important factor is marked as two asterisks which p-value ranges from 0.001 to 0.01. The third important factor is marked as an asterisk which p-value ranges from 0.01 to 0.1. The least important is also marked as a dot which p-value ranges from 0.1 to 1.

**Independent variables**	**Lag**	**Coefficients/Significant**		
		NB	Poisson	Quasi
Intercept		170.9 ***	284.70000 ***	281.647031
Cases	1			0.728567 ***
	2			0.161275 **
	3			
Average Pressure	0	-0.1734 ***	-0.172100 ***	
	1			-0.755294 *
	2		-0.1019000 ***	
	3			0.457230
Minimum Temperature	0			
	1			
	2			
	3			
Maximum Temperature	0	-0.03666	-0.070990 ***	
	1			
	2	0.03791	-0.104600 ***	
	3			
Relative Humidity	0			
	1			0.173574 .
	2	0.07803 ***		
	3			
Precipitation	0			
	1			
	2			
	3			
Vaporization	0			
	1			1.588354 *
	2	0.1557 *	0.1038000 ***	
	3			
Wind Direction	0		0.0004086	
	1			
	2			0.007645
	3			
Wind Power	0	0.0006551	-0.022640 ***	
	1			
	2	-0.04130 **	-0.0397900 ***	-0.126867
	3			

Phitsanulok

Phitsanulok is a province located in the northern region of Thailand at coordinate of 16°48′57″N 100°15′49″E. Phitsanulok covers an area of 10,816 km^2. Total population are 858,988 people. The density of population is 79.0 people per km^2. Weather in Phitsanulok has tropical savanna climate under the South Asian monsoon system. Temperature is in the range from the low of 3.9°C in January to the high of 41.8°C in April. The rainy season runs from May through October. The humidity presents the average of 62-81 percent throughout the year. Precipitation starts to rise from mid-May to August. The highest precipitation is in August of 247.6 mm. The longest sunshine hours are in January.

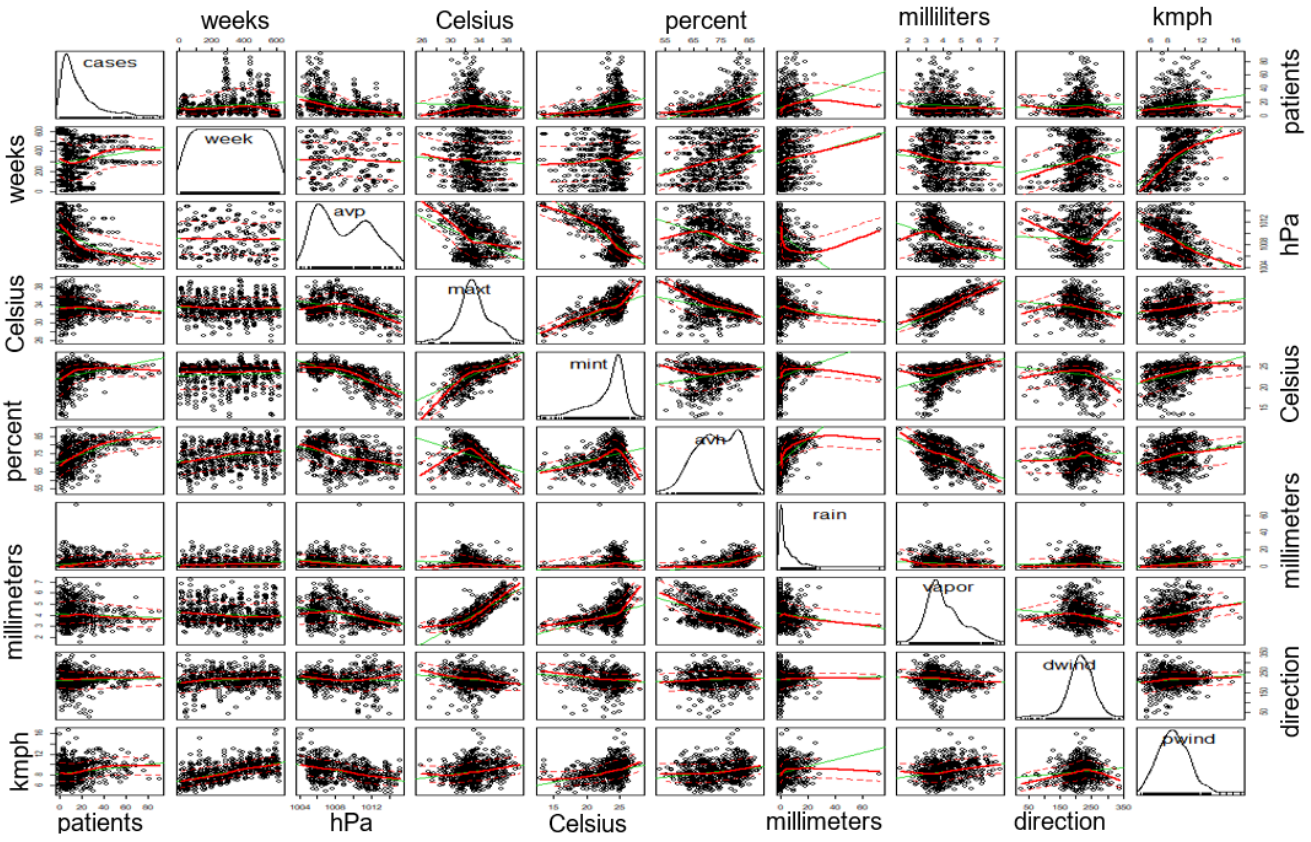

Figure 229: Scatter plot between dengue cases (cases) and selected independent variables, which are the weekly period starting from January 2001 – December 2013 (week), average pressure (avp), maximum temperature (maxt), minimum temperature (mint), average humidity (avh), precipitation (rain), vaporization of water (vapor), wind direction (dwind), and wind power (pwind). The plot visualizes pairwise hundred relationships of training set in Phitsanulok.

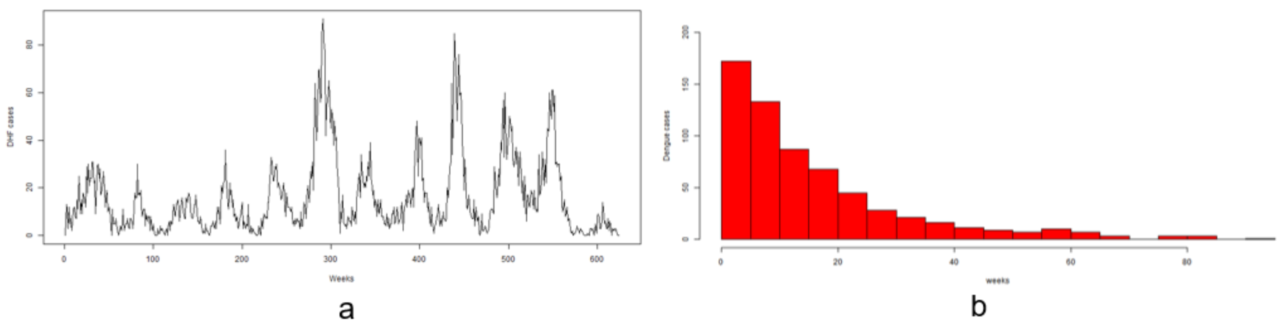

Figure 230: (a) Line plot between dengue incidences and weeks, the plot shows trends of dengue incidences in each year as stationary time series. (b) Histogram of dengue incidences in Phitsanulok starting from January 2001 to December 2013 (624 weeks).

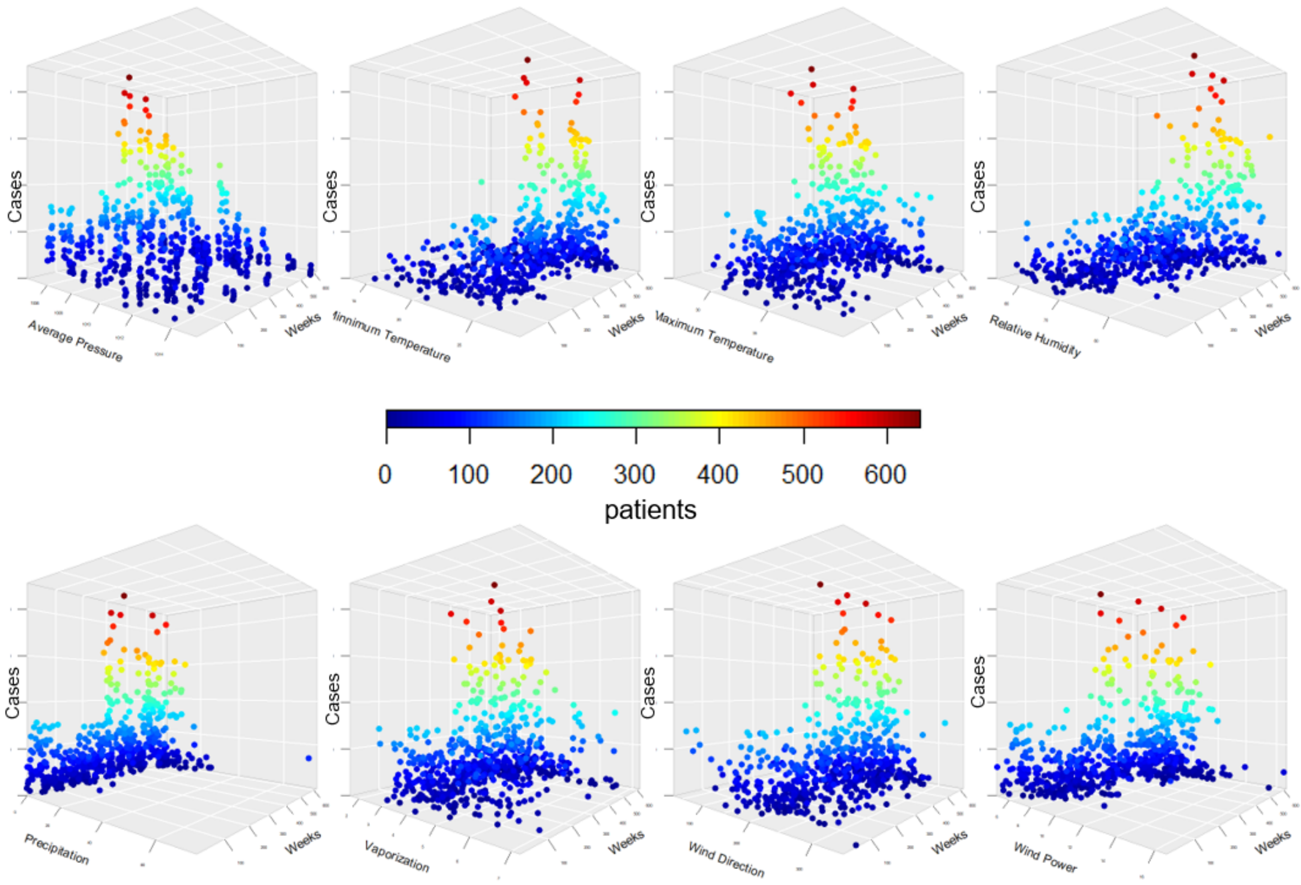

Figure 231: Three-dimensional scatter plot between dengue incidences and weather effects starting from January 2001 to December 2013 of Phitsanulok.

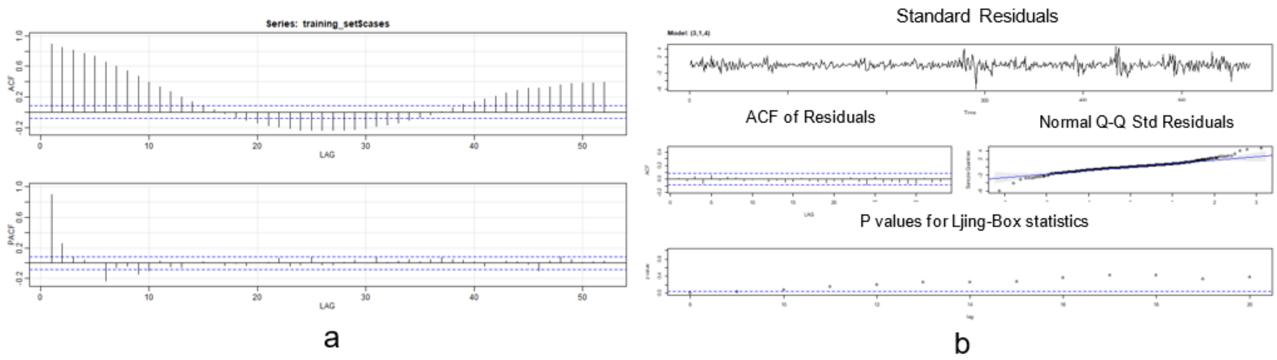

Figure 232: (a) Two plots between lag-time of dengue incidences and ACF and PACF relationship calculated from ARIMA model (b) Summary plots of time series analysis, multiple plots include the plot of predicted model over the time, the plot of ACF residual over lag-time of dengue incidences, residual Q-Q plot of standard residual, and p-value for Ljung-Box statistics of PACF relationship in Phitsanulok over the training data starting from January 2001 to December 2013.

Phitsanulok results the best model based on quasi-likelihood method. The correlation coefficient on the test set in 2014 is 0.22 (95%CI: 0.1033, 0.3367). The model uses 8 variables, which are 1-week-lag cases, 2-week-lag cases, following by 3-week-lag cases and 2-week-lag vaporization. Other variables which have less significant are, 1-week-lag precipitation, 1-week-lag, 2-week-lag and 3-week-lag wind direction. Time series methods by ARIMA and SARIMA yield the correlation coefficient of -1.302682 and -79.36553 respectively.

Table 77: Comparison table of all methods by the highest correlation coefficient (R^2) and the lowest prediction error (RMSE) in Phitsanulok.

Methods	R-squared (R^2)	Root mean square error (RMSE)
Poisson Regression	-3.732338	6.20957
Negative Binomial Regression	-1.829798	4.80177
Quasi-likelihood Regression	0.2207083	2.519844
ARIMA (3,1,4)	-1.302682	4.331523
SARIMA $(2,0,1)(0,2,0)_{52}$	-79.36553	25.58931

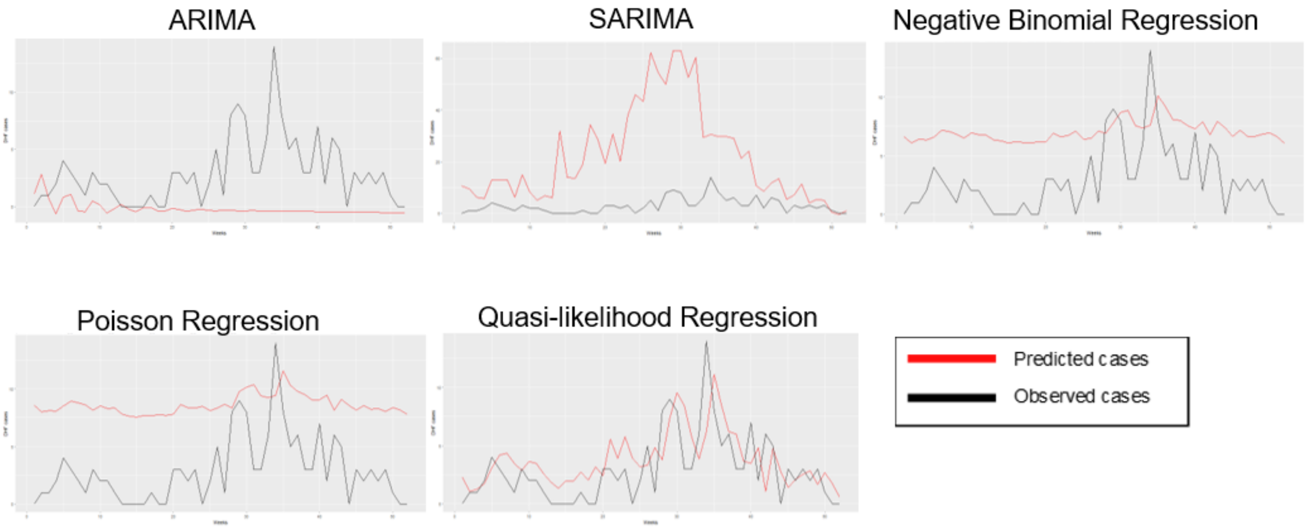

Figure 233: Plots between dengue cases and weeks, the black line represents the observed dengue cases, and the red line represents the predicted dengue cases of the best fit model of each technique over the test set data starting from January 2014 to December 2014.

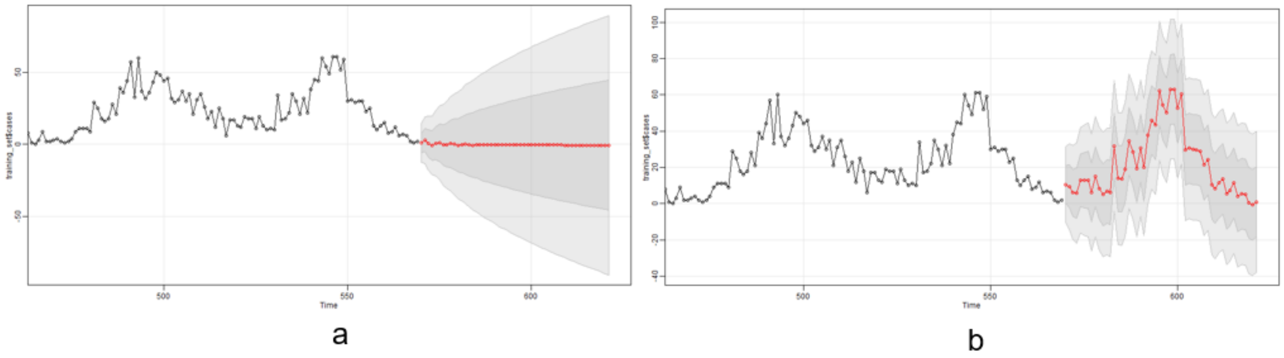

Figure 234: (a) Plot between dengue incidences over weekly time by the best model of ARIMA and (b) SARIMA time series analysis, the black line represents training set data starting from January 2012 to December 2013, and the red line represents the forecasted dengue incidences from January 2014 to December 2014.

Table 78: Coefficients and significant values of best fit GLM models, Negative Binomial, Poisson and Quasi-likelihood regression model of Phitsanulok. The table summarizes coefficients of each independent variables which are composed in best fit model of each method. The significant of each variable is labelled by asterisks under the coefficients. The most important factor is marked as three asterisks which p-value ranges from 0 to 0.001. The second important factor is marked as two asterisks which p-value ranges from 0.001 to 0.01. The third important factor is marked as an asterisk which p-value ranges from 0.01 to 0.1. The least important is also marked as a dot which p-value ranges from 0.1 to 1.

**Independent variables**	**Lag**	**Coefficients/Significant**		
		NB	Poisson	Quasi
Intercept		1.8113472 ***	1.9079349 ***	-6.389207 *
Cases	1	0.0280835 ***	0.0225972 ***	0.626000 ***
	2	0.0120833 ***	0.0049418 ***	0.202289 ***
	3	0.0047756	0.0069316 ***	0.112012 **
Average Pressure	0			
	1			
	2			
	3			
Minimum Temperature	0			
	1			
	2			
	3			
Maximum Temperature	0			
	1			
	2			
	3			
Relative Humidity	0			
	1			
	2			
	3			
Precipitation	0			
	1			-0.065649
	2			
	3			
Vaporization	0			
	1			
	2			0.938187 **
	3			
Wind Direction	0	-0.0004847	-0.0001924	
	1	0.0004815	0.0002726	0.007492
	2		0.0001940	0.001862
	3		0.0003858	0.008758
Wind Power	0			
	1			
	2			
	3			

Phrae

Phrae is a province located in northern region of Thailand, in the mountainous geography at 18°08′43″N 100°08′31″E. Phrae covers an area of 6,539 km^2. Total population are 454,083 people. The density of population is 69.0 km^2. Weather in Phrae has tropical savanna climate under the South Asian monsoon system which controls more tropical wet and dry climate. Temperature is in the range from the low of 5.8 °C in January to the high of 43.6°C in April. The rainy season runs with the arrival of the southwest monsoon around mid-May through October. The humidity presents the average of 62-84 percent. Precipitation starts to rise from mid-May to August. The highest precipitation is in August of 205.5 mm. The longest sunshine hours are in January.

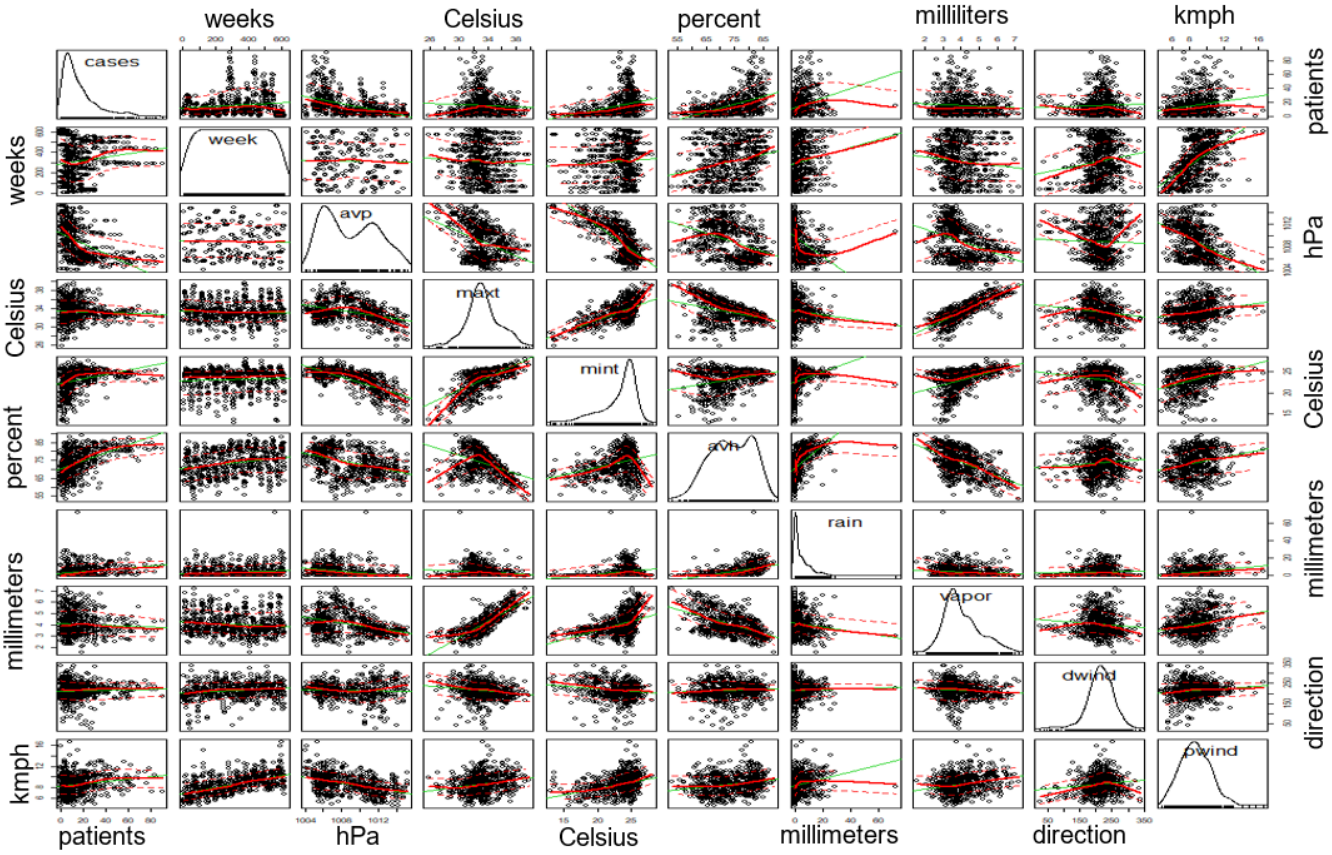

Figure 235: Scatter plot between dengue cases (cases) and selected independent variables, which are the weekly period starting from January 2001 – December 2013 (week), average pressure (avp), maximum temperature (maxt), minimum temperature (mint), average humidity (avh), precipitation (rain), vaporization of water (vapor), wind direction (dwind), and wind power (pwind). The plot visualizes pairwise hundred relationships of training set in Phrae.

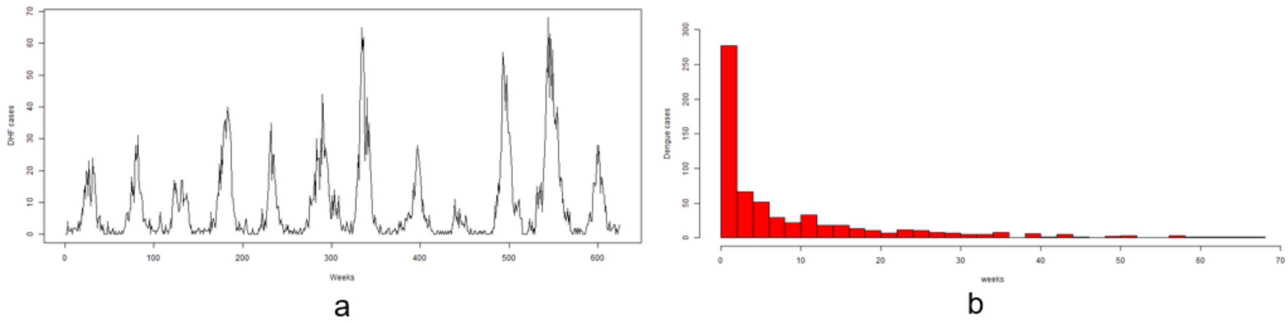

Figure 236: (a) Line plot between dengue incidences and weeks, the plot shows trends of dengue incidences in each year as stationary time series. (b) Histogram of dengue incidences in Phrae starting from January 2001 to December 2013 (624 weeks).

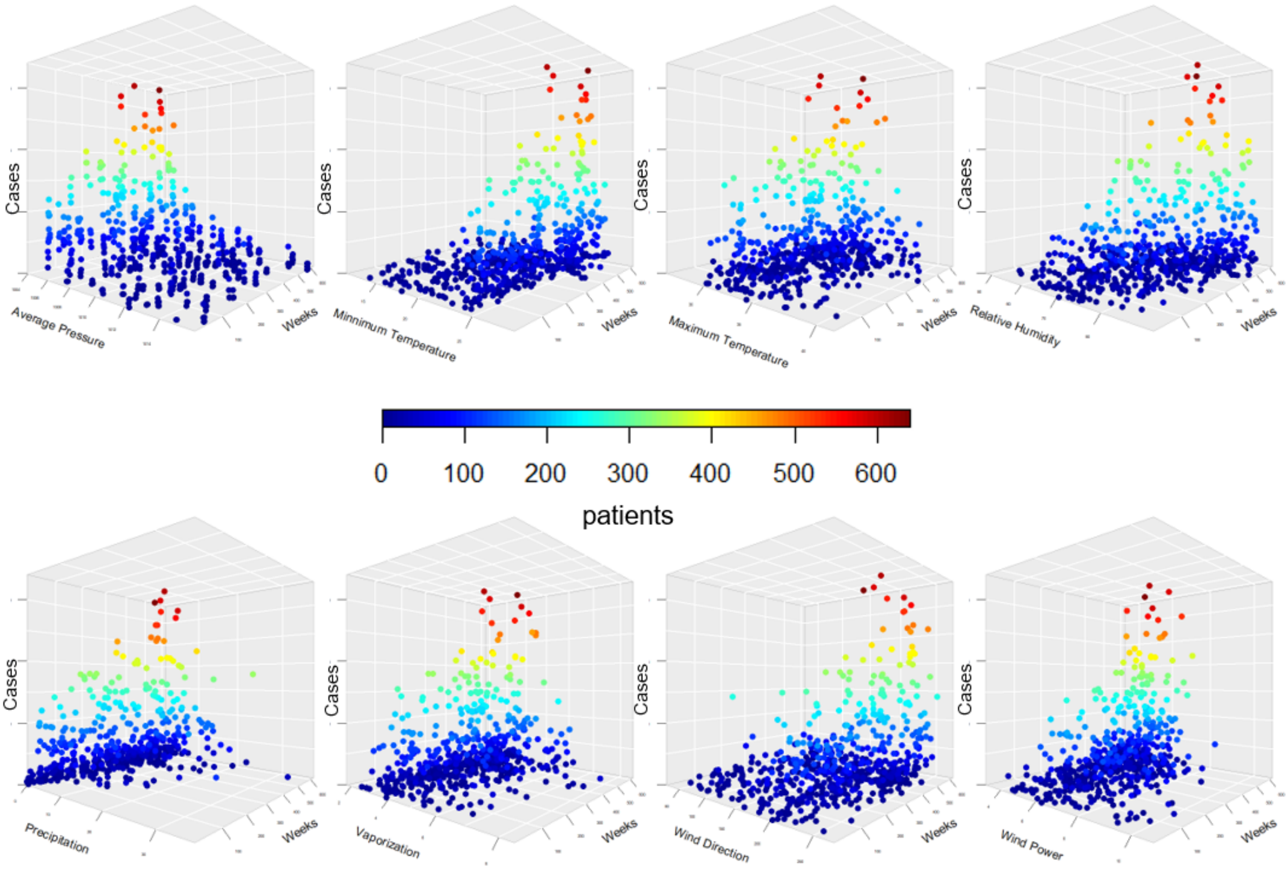

Figure 237: Three-dimensional scatter plot between dengue incidences and weather effects starting from January 2001 to December 2013 of Phrae.

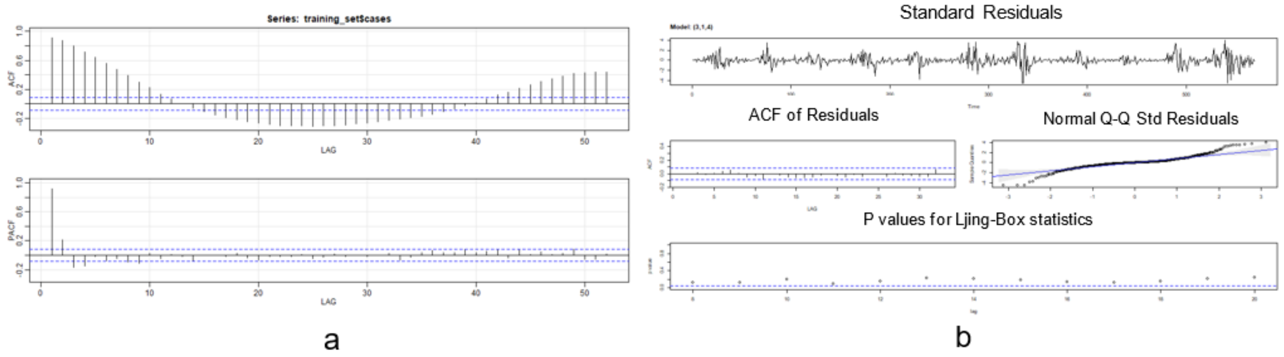

Figure 238: (a) Two plots between lag-time of dengue incidences and ACF and PACF relationship calculated from ARIMA model (b) Summary plots of time series analysis, multiple plots include the plot of predicted model over the time, the plot of ACF residual over lag-time of dengue incidences, residual Q-Q plot of standard residual, and p-value for Ljung-Box statistics of PACF relationship in Phrae over the training data starting from January 2001 to December 2013.

For Phrae, the best model yields correlation coefficient on the test set in 2014 of 0.864 (95%CI: 0.8123, 0.9157). the model is based on Poisson regression method. The significant of the variables associated with p-value statistical calculation are shown in Table PHE2. The best model of Phrae consists of 8 variables. The most significant variables are 1-week-lag cases, 2-week-lag cases, current week average pressure, 3-week-lag wind direction, following by 3-week-lag average pressure and current week wind direction. Other variable is 2-week-lag average pressure. Time series methods by ARIMA and SARIMA yield the correlation coefficient of -0.679133 and -6.1080220 respectively.

Table 79: Comparison table of all methods by the highest correlation coefficient (R^2) and the lowest prediction error (RMSE) in Phrae.

Methods	R-squared (R^2)	Root mean square error (RMSE)
Poisson Regression	0.863809	2.787641
Negative Binomial Regression	0.8581161	2.845308
Quasi-likelihood Regression	0.8342904	3.07494
ARIMA (3,1,4)	-0.679133	9.788255
SARIMA $(2,0,1)(0,2,0)_{52}$	-6.108022	20.13896

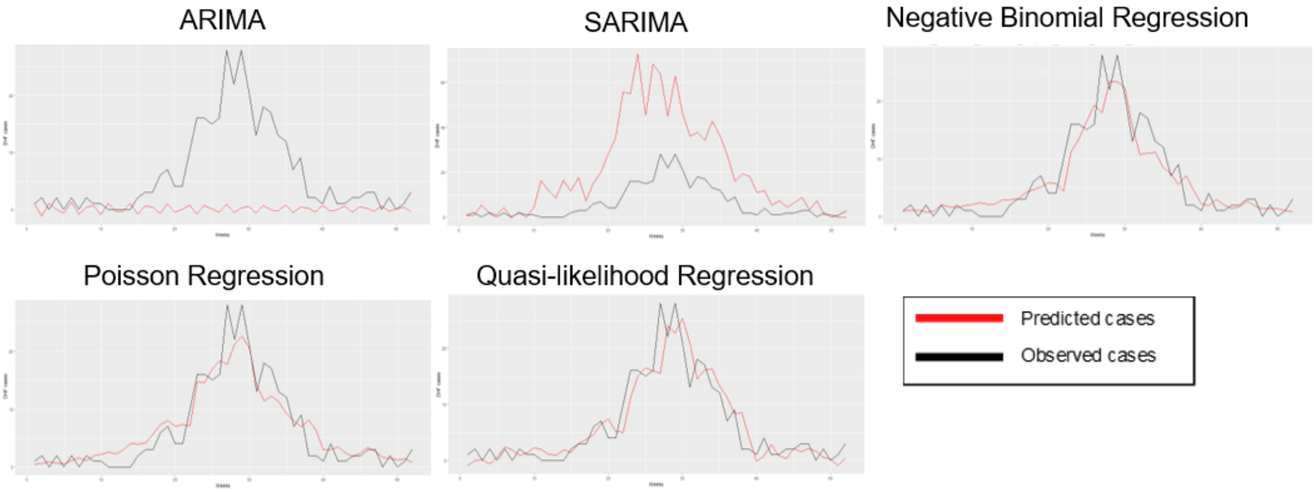

Figure 239: Plots between dengue cases and weeks, the black line represents the observed dengue cases, and the red line represents the predicted dengue cases of the best fit model of each technique over the test set data starting from January 2014 to December 2014.

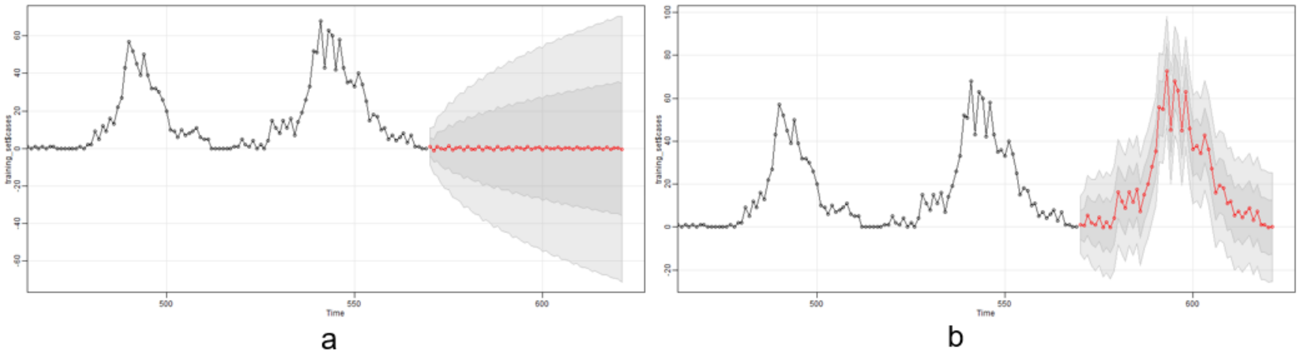

Figure 240: (a) Plot between dengue incidences over weekly time by the best model of ARIMA and (b) SARIMA time series analysis, the black line represents training set data starting from January 2012 to December 2013, and the red line represents the forecasted dengue incidences from January 2014 to December 2014.

Table 80: Coefficients and significant values of best fit GLM models, Negative Binomial, Poisson and Quasi-likelihood regression model of Phrae. The table summarizes coefficients of each independent variables which are composed in best fit model of each method. The significant of each variable is labelled by asterisks under the coefficients. The most important factor is marked as three asterisks which p-value ranges from 0 to 0.001. The second important factor is marked as two asterisks which p-value ranges from 0.001 to 0.01. The third important factor is marked as an asterisk which p-value ranges from 0.01 to 0.1. The least important is also marked as a dot which p-value ranges from 0.1 to 1.

**Independent variables**	**Lag**	**Coefficients/Significant**		
		NB	Poisson	Quasi
Intercept		225.8 ***	165.5000 ***	349.613992 **
Cases	1	0.03816 ***	0.023860 ***	0.677564 ***
	2	0.01486 **	0.013350 ***	0.222128 ***
	3			
Average Pressure	0	-0.1647 ***	-0.152100 ***	-0.640443 ***
	1			0.298919
	2		0.02706	
	3	-0.05745 **	-0.039600 *	
Minimum Temperature	0			
	1			
	2		0.096760 ***	
	3			
Maximum Temperature	0			
	1			
	2			
	3			
Relative Humidity	0			
	1			
	2			-0.038543
	3			
Precipitation	0			
	1	0.003887		
	2			
	3	0.008595		
Vaporization	0			
	1			
	2			
	3			
Wind Direction	0		0.001044 *	
	1			
	2			-0.007765 .
	3	-0.003276 ***	-0.002509 ***	
Wind Power	0			
	1			0.155683
	2			-0.121592
	3			

Phuket

Phuket is a province located in southern region of Thailand at 7°95′19″N 98°33′81″E. Phuket covers an area of 543 km^2. Total population are 378,364 people. The density of population is 697.0 per km^2. Weather in Phuket has tropical savanna climate under the South Asian monsoon system which has shorter winter period. Temperature in Phuket has an average annual high of 32°C and an annual low of 25°C. Phuket has a dry season that runs from December through March and a wet season that covers the other eight months. The rainy season begins with the arrival of the southwest monsoon around mid-May. Precipitation occurs from mid-May to August. Rainfall is averaged roughly 2,200 mm annually

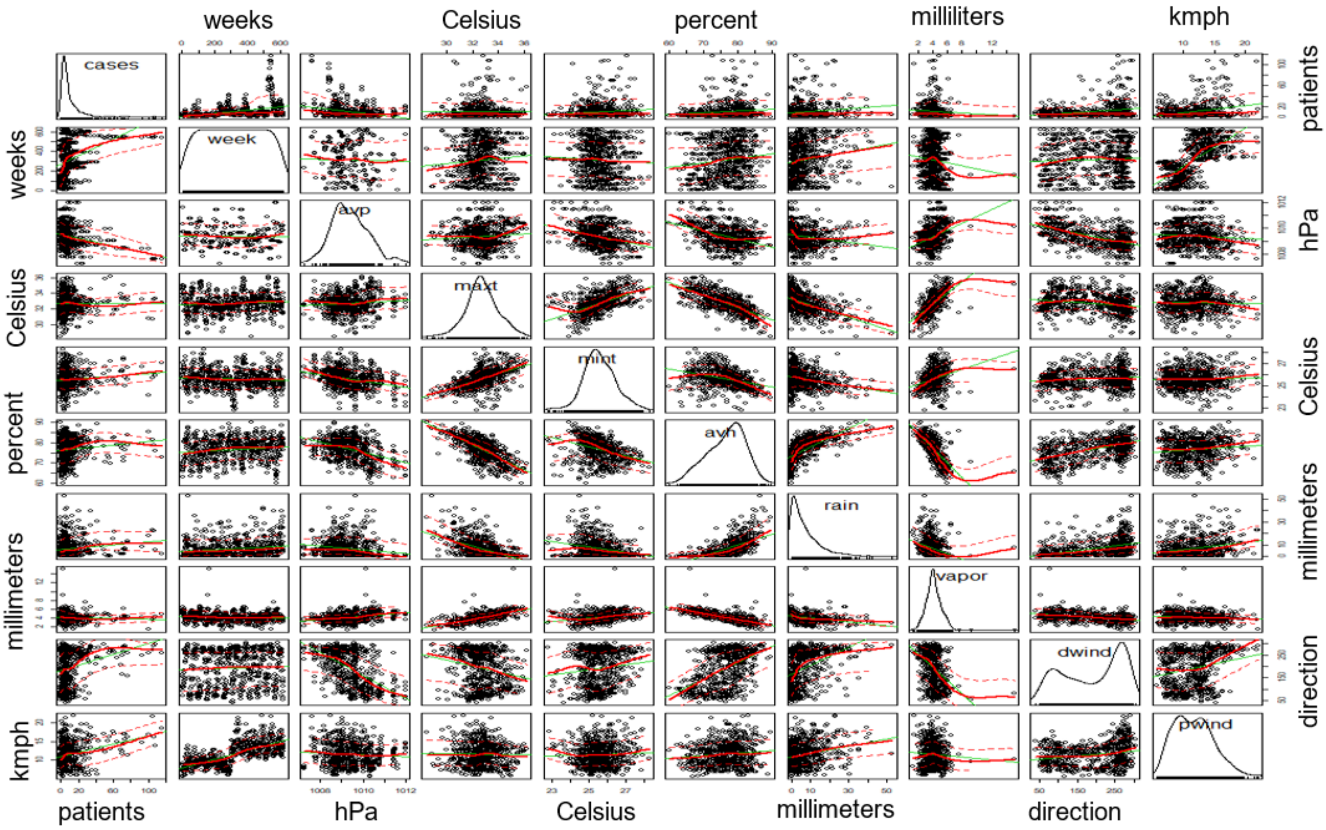

Figure 241: Scatter plot between dengue cases (cases) and selected independent variables, which are the weekly period starting from January 2001 – December 2013 (week), average pressure (avp), maximum temperature (maxt), minimum temperature (mint), average humidity (avh), precipitation (rain), vaporization of water (vapor), wind direction (dwind), and wind power (pwind). The plot visualizes pairwise hundred relationships of training set in Phuket.

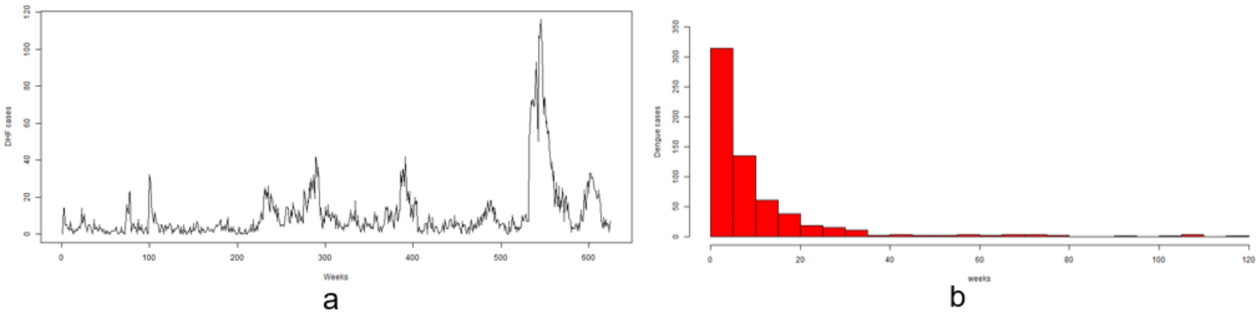

Figure 242: (a) Line plot between dengue incidences and weeks, the plot shows trends of dengue incidences in each year as stationary time series. (b) Histogram of dengue incidences in Phuket starting from January 2001 to December 2013 (624 weeks).

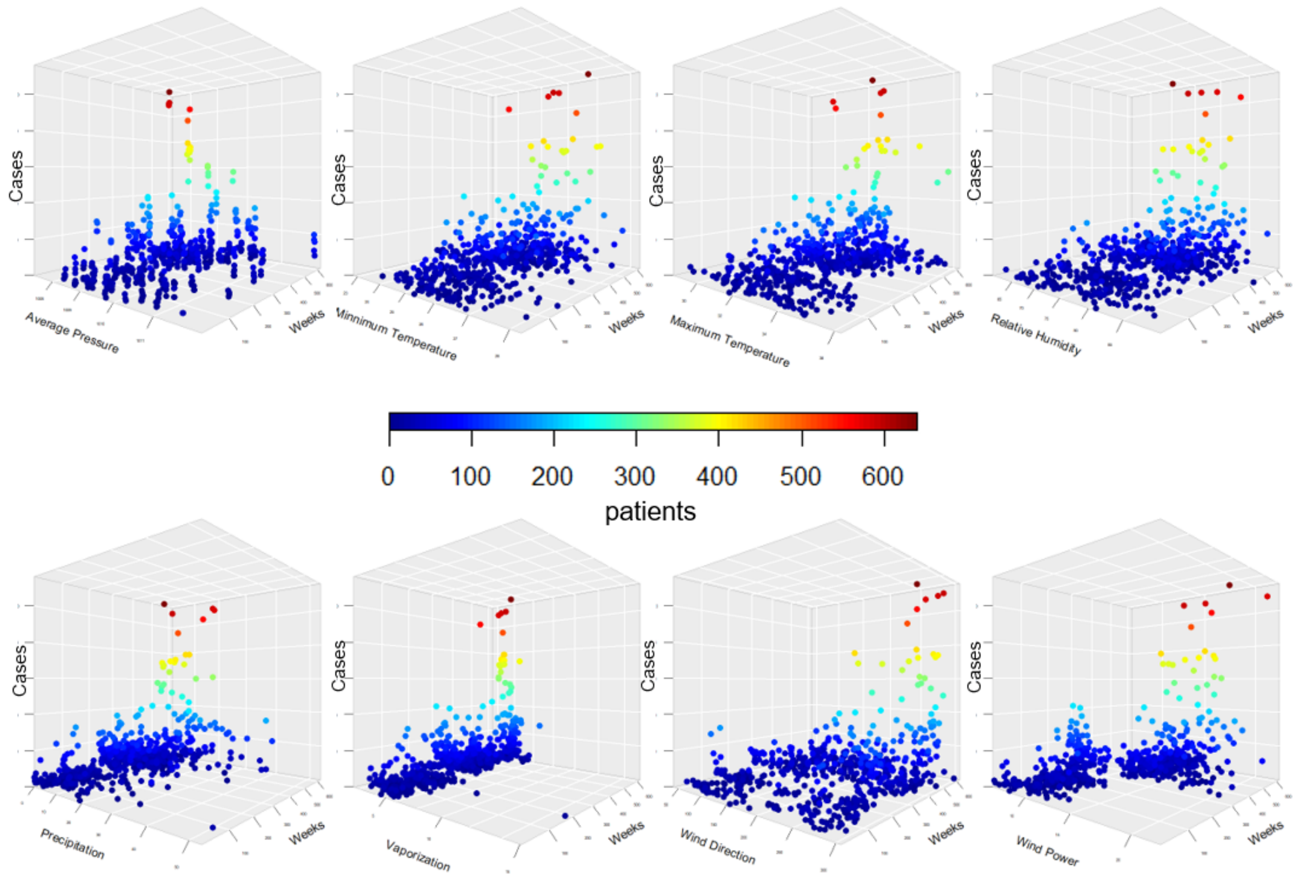

Figure 243: Three-dimensional scatter plot between dengue incidences and weather effects starting from January 2001 to December 2013 of Phuket.

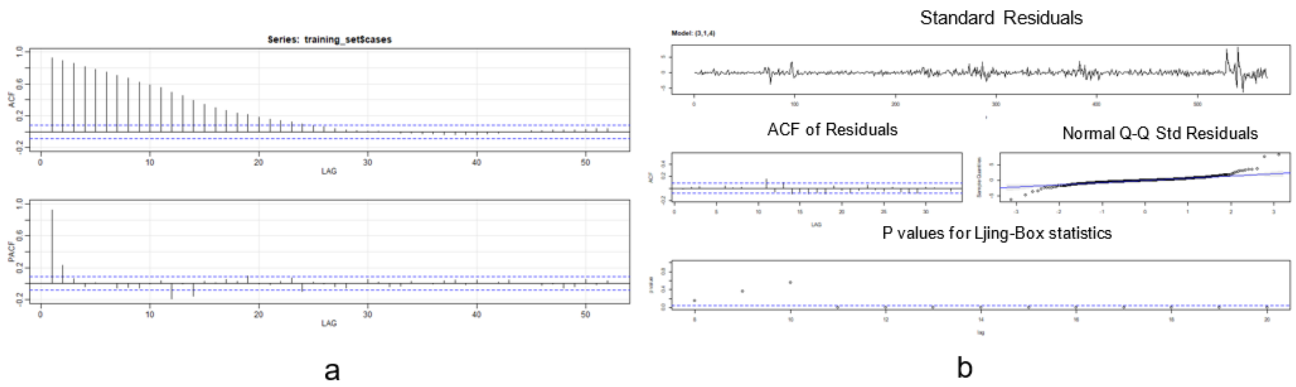

Figure 244: (a) Two plots between lag-time of dengue incidences and ACF and PACF relationship calculated from ARIMA model (b) Summary plots of time series analysis, multiple plots include the plot of predicted model over the time, the plot of ACF residual over lag-time of dengue incidences, residual Q-Q plot of standard residual, and p-value for Ljung-Box statistics of PACF relationship in Phuket over the training data starting from January 2001 to December 2013.

For Phuket, the best prediction model presents the correlation coefficient of 0.791 (95%CI: 0.7234, 0.8586) by using quasi-likelihood method. The model comprises of 8 variables. The most significant variables are 1-week-lag cases, 3-week-lag cases and 1-week-lag minimum temperature. Other components are 1-week-lag average pressure, 2 and 3 -week-lag relative humidity, current and 2-week-lag wind power. Comparison to ARIMA and SARIMA, the correlation coefficient results 0.132 and 0 respectively.

Table 81: Comparison table of all methods by the highest correlation coefficient (R^2) and the lowest prediction error (RMSE) in phuket.

**Methods**	**R-squared (R^2)**	**Root mean square error (RMSE)**
Poisson Regression	0.557543	6.370165
Negative Binomial Regression	0.512598	6.685889
Quasi-likelihood Regression	0.794111	4.345417
ARIMA (3,1,4)	0.131787	10.18821
SARIMA $(2,0,1)(0,2,0)_{52}$	-25.87973	49.65099

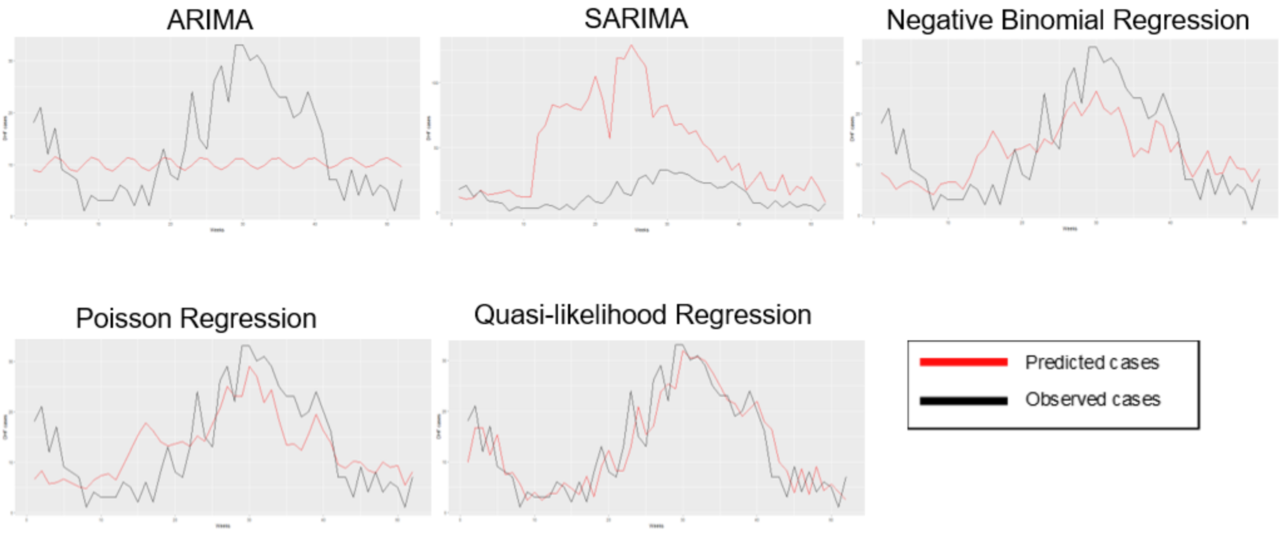

Figure 245: Plots between dengue cases and weeks, the black line represents the observed dengue cases, and the red line represents the predicted dengue cases of the best fit model of each technique over the test set data starting from January 2014 to December 2014.

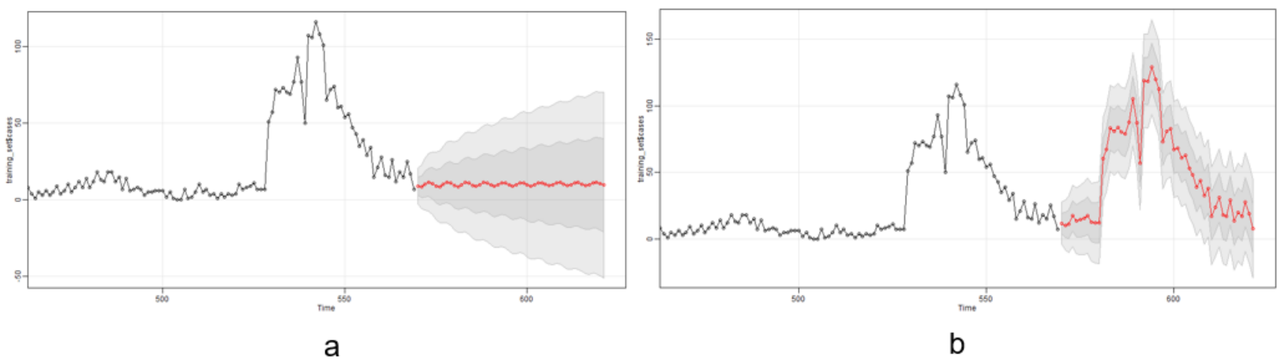

Figure 246: (a) Plot between dengue incidences over weekly time by the best model of ARIMA and (b) SARIMA time series analysis, the black line represents training set data starting from January 2012 to December 2013, and the red line represents the forecasted dengue incidences from January 2014 to December 2014.

Table 82: Coefficients and significant values of best fit GLM models, Negative Binomial, Poisson and Quasi-likelihood regression model of Phuket. The table summarizes coefficients of each independent variables which are composed in best fit model of each method. The significant of each variable is labelled by asterisks under the coefficients. The most important factor is marked as three asterisks which p-value ranges from 0 to 0.001. The second important factor is marked as two asterisks which p-value ranges from 0.001 to 0.01. The third important factor is marked as an asterisk which p-value ranges from 0.01 to 0.1. The least important is also marked as a dot which p-value ranges from 0.1 to 1.

**Independent variables**	**Lag**	**Coefficients/Significant**		
		NB	Poisson	Quasi
Intercept		-4.79388 **	-5.84740 ***	158.0551
Cases	1			0.776810 ***
	2			
	3			0.159250 ***
Average Pressure	0			
	1			-0.17810
	2			
	3			
Minimum Temperature	0			
	1			0.804740 *
	2	0.103840	0.123909 ***	
	3	0.122955 .	0.111589 ***	
Maximum Temperature	0			
	1			
	2			
	3			
Relative Humidity	0			
	1			
	2			-0.05380
	3			0.07410
Precipitation	0			
	1			
	2			
	3	0.009674	0.006567 ***	
Vaporization	0		0.050165 ***	
	1			
	2			
	3	-0.05641		
Wind Direction	0		0.002697 ***	
	1	0.002869 ***		
	2			
	3		0.000809 **	
Wind Power	0			-0.13434
	1			
	2	0.072547 ***	0.056055 ***	0.15294
	3		0.040521 ***	

Pra Nakhon Sri Ayutthaya

Pra Nakhon Sri Ayutthaya is located in the central region of Thailand at 14°20′52″N 100°33′38″E. Pra Nakhon Sri Ayutthaya covers an area of 2,557 km^2. Total population are 803,599 people. The density of population is 314.0 people per km^2. Weather at Pra Nakhon Sri Ayutthaya has tropical savanna climate under the South Asian monsoon system. Temperature is in the range from the low of 2.4°C in December to the high of 35.9°C in April. The monsoon season begins with the arrival of the southwest monsoon around mid-May. August is the wettest month, with an average rainfall of 260.0 mm. Relative humidity is high in rainy season from May-September.

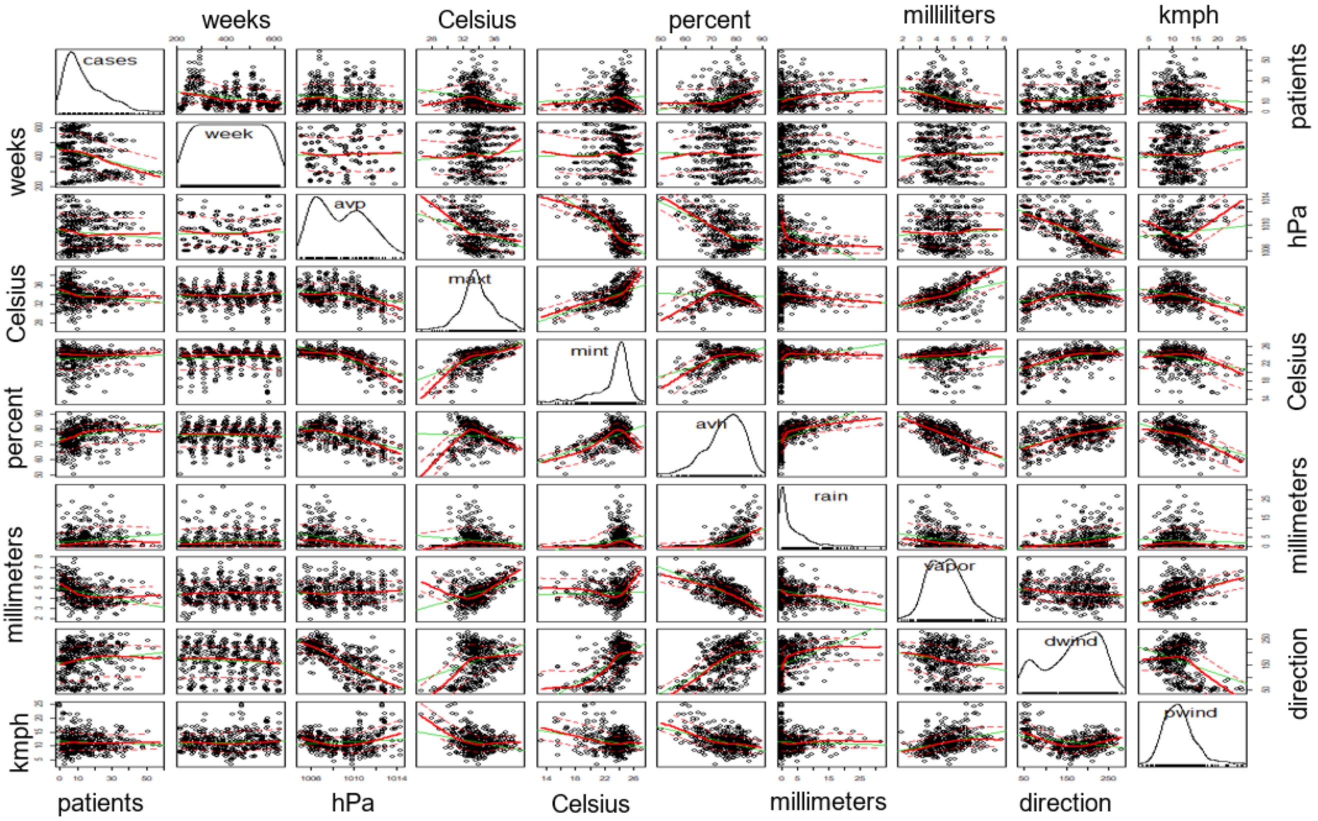

Figure 247: Scatter plot between dengue cases (cases) and selected independent variables, which are the weekly period starting from January 2001 – December 2013 (week), average pressure (avp), maximum temperature (maxt), minimum temperature (mint), average humidity (avh), precipitation (rain), vaporization of water (vapor), wind direction (dwind), and wind power (pwind). The plot visualizes pairwise hundred relationships of training set in Pra Nakhon Sri Ayutthaya.

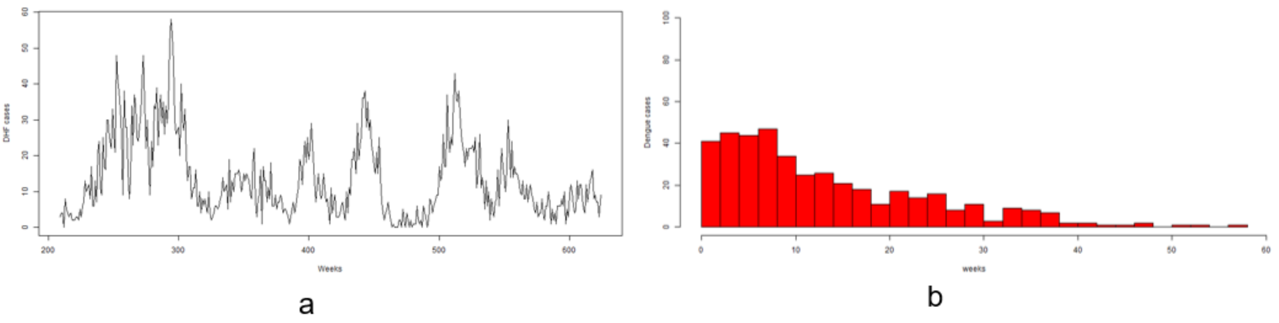

Figure 248: (a) Line plot between dengue incidences and weeks, the plot shows trends of dengue incidences in each year as stationary time series. (b) Histogram of dengue incidences in Pra Nakhon Sri Ayutthaya starting from January 2001 to December 2013 (624 weeks).

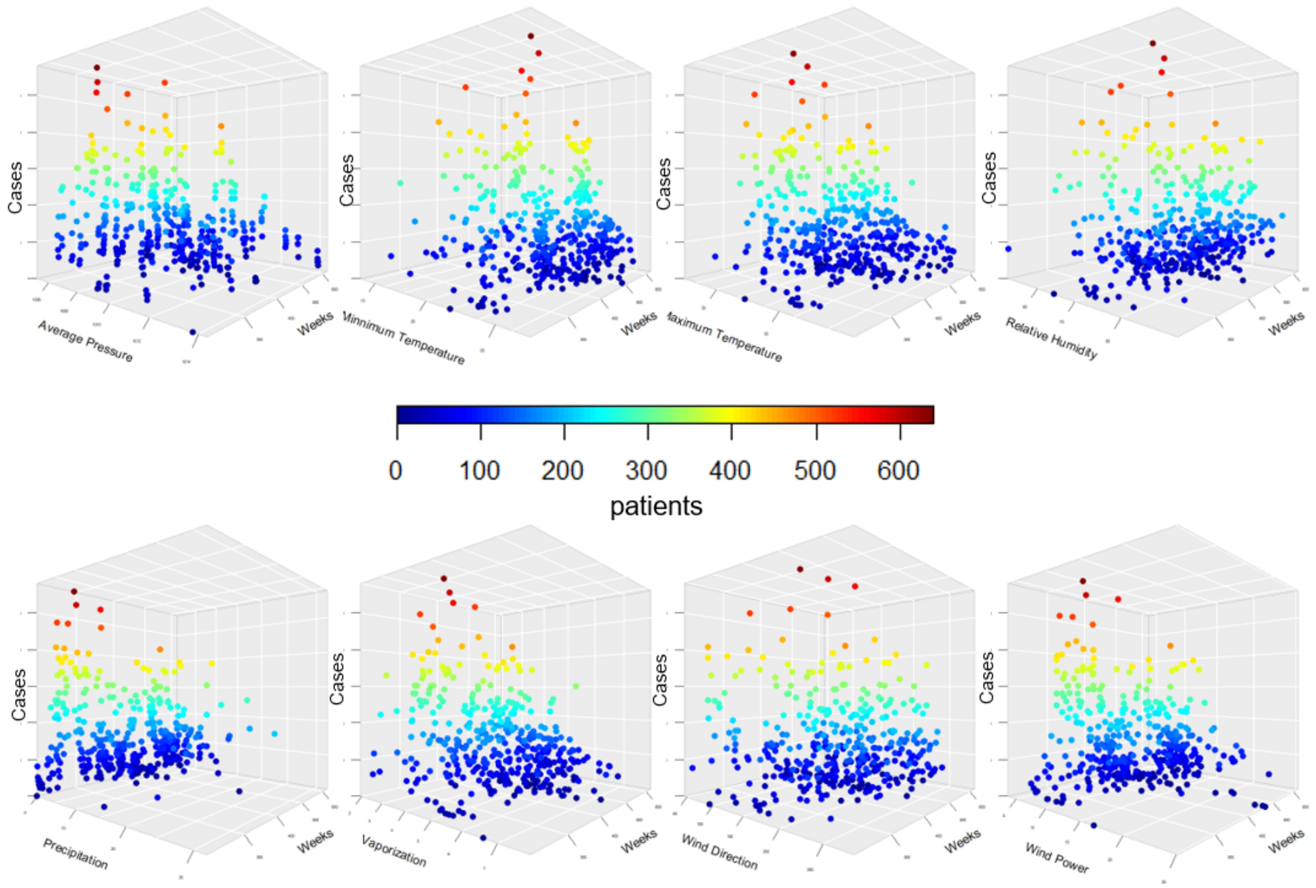

Figure 249: Three-dimensional scatter plot between dengue incidences and weather effects starting from January 2001 to December 2013 of Pra Nakhon Sri Ayutthaya.

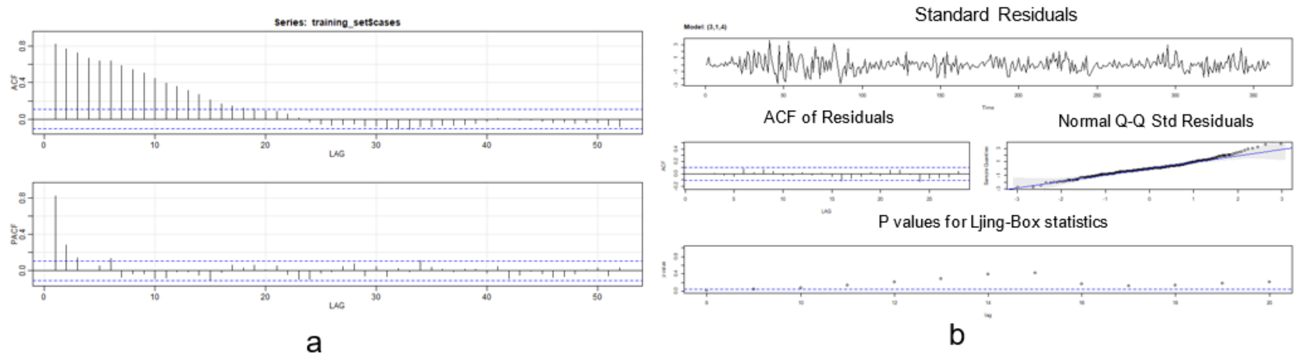

Figure 250: (a) Two plots between lag-time of dengue incidences and ACF and PACF relationship calculated from ARIMA model (b) Summary plots of time series analysis, multiple plots include the plot of predicted model over the time, the plot of ACF residual over lag-time of dengue incidences, residual Q-Q plot of standard residual, and p-value for Ljung-Box statistics of PACF relationship in Pra Nakhon Sri Ayutthaya over the training data starting from January 2001 to December 2013.

For Pra Nakhon Sri Ayutthaya, the best model of is based on Negative Binomial regression method. The correlation coefficient on the test set in 2014 is 0.225 (95%CI: 0.1153, 0.3346). The best model uses 7 variables. The most significant variables are 1-week-lag cases, 2-week-lag cases, following by 2-week-lag average pressures, current week vaporization and 1-week-lag wind power. Other variables are 1-week-lag precipitation and 3-week-lag wind power. Time series methods by ARIMA and SARIMA yield the correlation coefficient of -1.331878 and -4.514609 respectively.

Table 83: Comparison table of all methods by the highest correlation coefficient (R^2) and the lowest prediction error (RMSE) in Pra Nakhon Sri Ayutthaya.

Methods	R-squared (R^2)	Root mean square error (RMSE)
Poisson Regression	0.1101838	3.326773
Negative Binomial Regression	0.225659	3.10341
Quasi-likelihood Regression	0.1697905	3.213415
ARIMA (3,1,4)	-1.331878	5.385499
SARIMA $(2,0,1)(0,2,0)_{52}$	-4.514609	8.281907

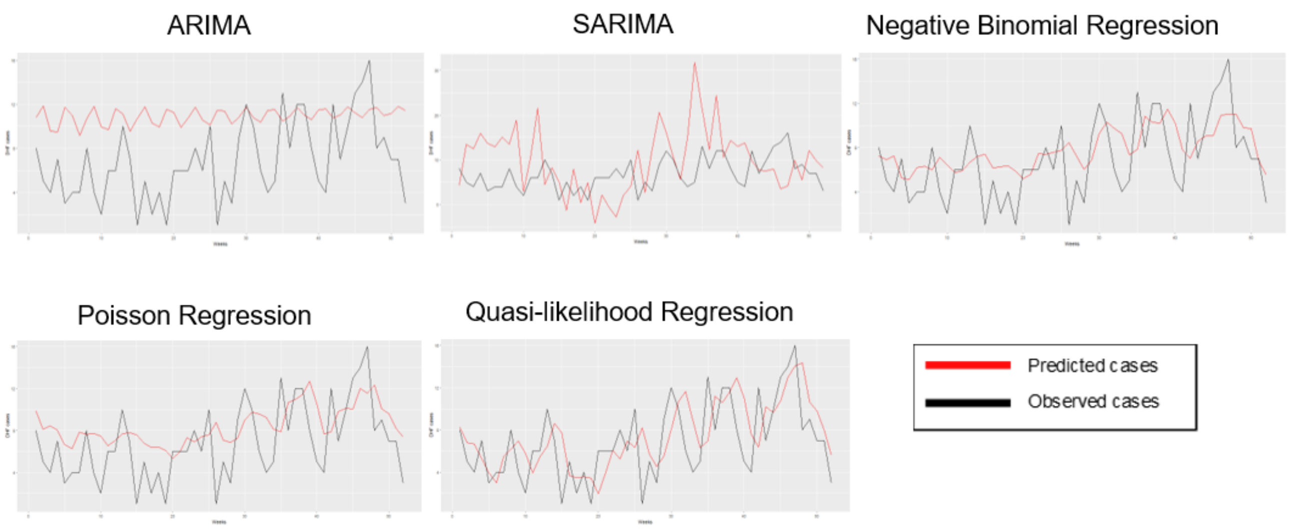

Figure 251: Plots between dengue cases and weeks, the black line represents the observed dengue cases, and the red line represents the predicted dengue cases of the best fit model of each technique over the test set data starting from January 2014 to December 2014.

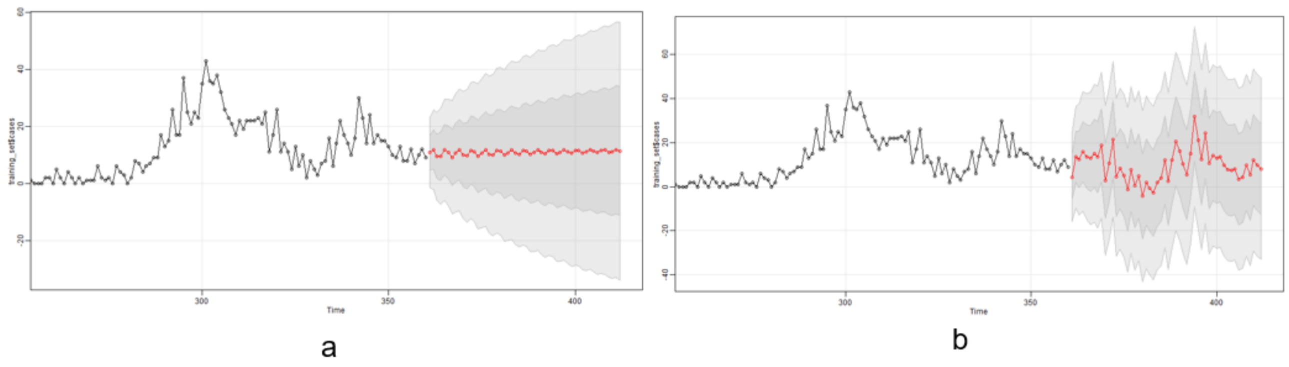

Figure 252: (a) Plot between dengue incidences over weekly time by the best model of ARIMA and (b) SARIMA time series analysis, the black line represents training set data starting from January 2012 to December 2013, and the red line represents the forecasted dengue incidences from January 2014 to December 2014.

Table 84: Coefficients and significant values of best fit GLM models, Negative Binomial, Poisson and Quasi-likelihood regression model of Pra Nakhon Sri Ayutthaya. The table summarizes coefficients of each independent variables which are composed in best fit model of each method. The significant of each variable is labelled by asterisks under the coefficients. The most important factor is marked as three asterisks which p-value ranges from 0 to 0.001. The second important factor is marked as two asterisks which p-value ranges from 0.001 to 0.01. The third important factor is marked as an asterisk which p-value ranges from 0.01 to 0.1. The least important is also marked as a dot which p-value ranges from 0.1 to 1.

**Independent variables**	**Lag**	**Coefficients/Significant**		
		NB	Poisson	Quasi
Intercept		29.812071 *	22.659166 *	2.14928
Cases	1	0.0319860 ***	0.028922 ***	0.56037 ***
	2	0.0232780 ***	0.015945 ***	0.29398 ***
	3			
Average Pressure	0		-0.019152 .	
	1			
	2	-0.027502 *		
	3			
Minimum Temperature	0		0.010593	
	1			0.34838
	2			
	3			
Maximum Temperature	0			
	1			
	2			
	3		-0.038280 ***	-0.39265 .
Relative Humidity	0		-0.007997 .	0.05803
	1			
	2		0.006262 *	0.05276
	3			-0.05864
Precipitation	0			
	1	0.003962	0.007224 *	
	2			
	3			
Vaporization	0	-0.072857 *	-0.072224 **	
	1			
	2			
	3			
Wind Direction	0			
	1			
	2			
	3			
Wind Power	0			
	1	-0.016378 .		
	2			0.10363
	3	0.009455		

Prachin Buri

Prachin Buri is located at the eastern of Thailand at 14°03′24″N 101°22′26″E. Prachin Buri covers an area of 4,762 km^2. The total populations are 479,314 people. The density of population is 101.0 people per km^2. Weather in Prachin Buri has tropical savanna climate system. In winter occurs from December to January. Temperatures are in the range 5.0°C in December to 42.2°C in April. The highest rainfall presents in August of 358.5 mm. Relative humidity are high in rainy season and low in winter approximately 65-83 percent. January is the highest month of sunshine hours.

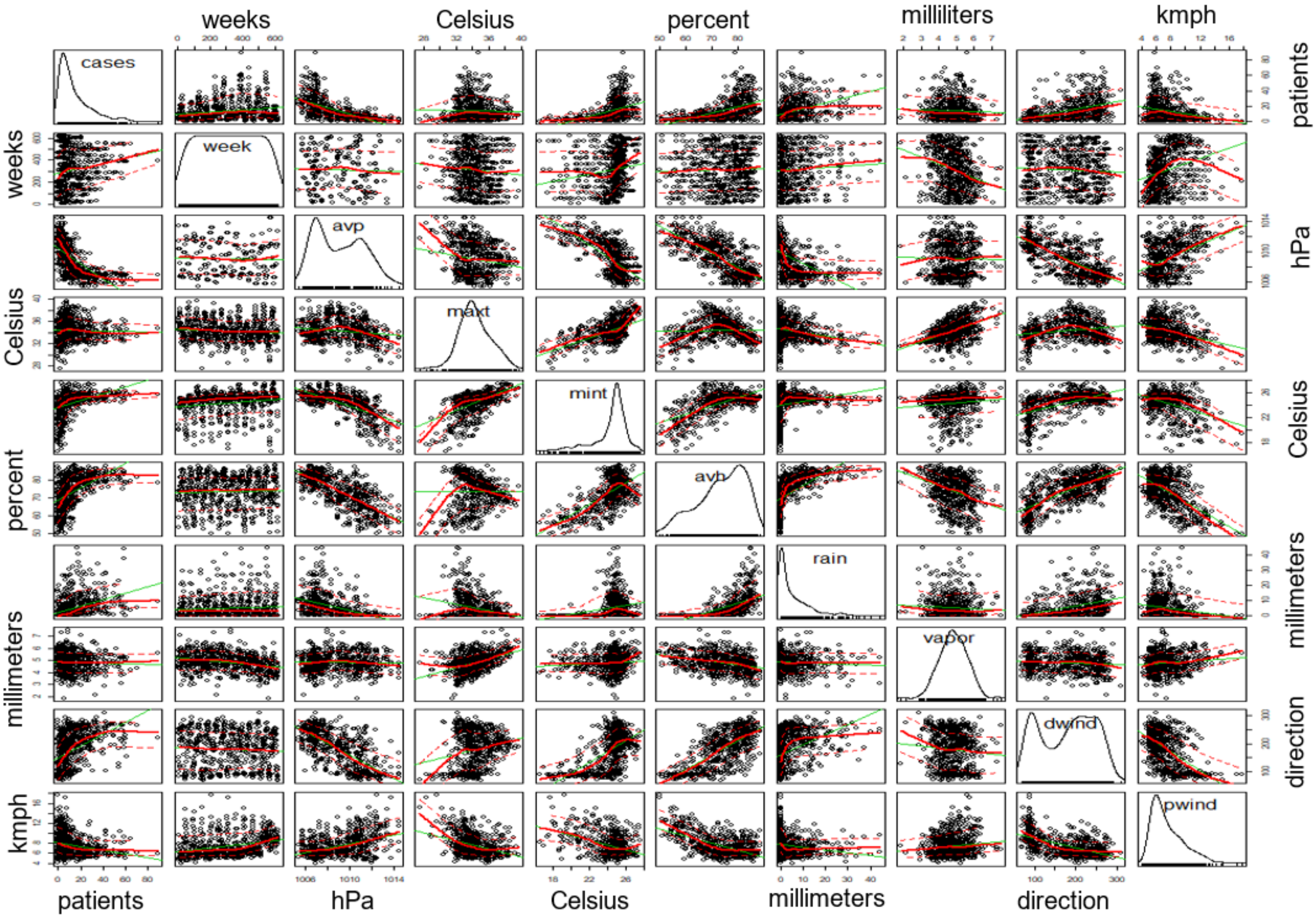

Figure 253: Scatter plot between dengue cases (cases) and selected independent variables, which are the weekly period starting from January 2001 – December 2013 (week), average pressure (avp), maximum temperature (maxt), minimum temperature (mint), average humidity (avh), precipitation (rain), vaporization of water (vapor), wind direction (dwind), and wind power (pwind). The plot visualizes pairwise hundred relationships of training set in Prachin Buri.

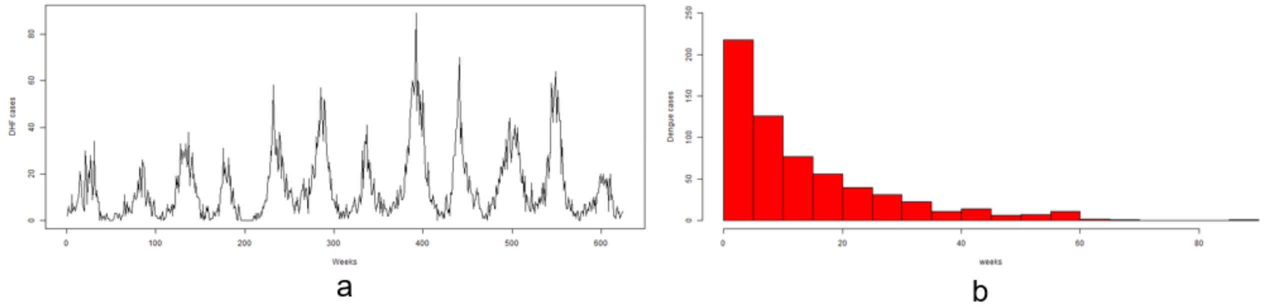

Figure 254: (a) Line plot between dengue incidences and weeks, the plot shows trends of dengue incidences in each year as stationary time series. (b) Histogram of dengue incidences in Prachin Buri starting from January 2001 to December 2013 (624 weeks).

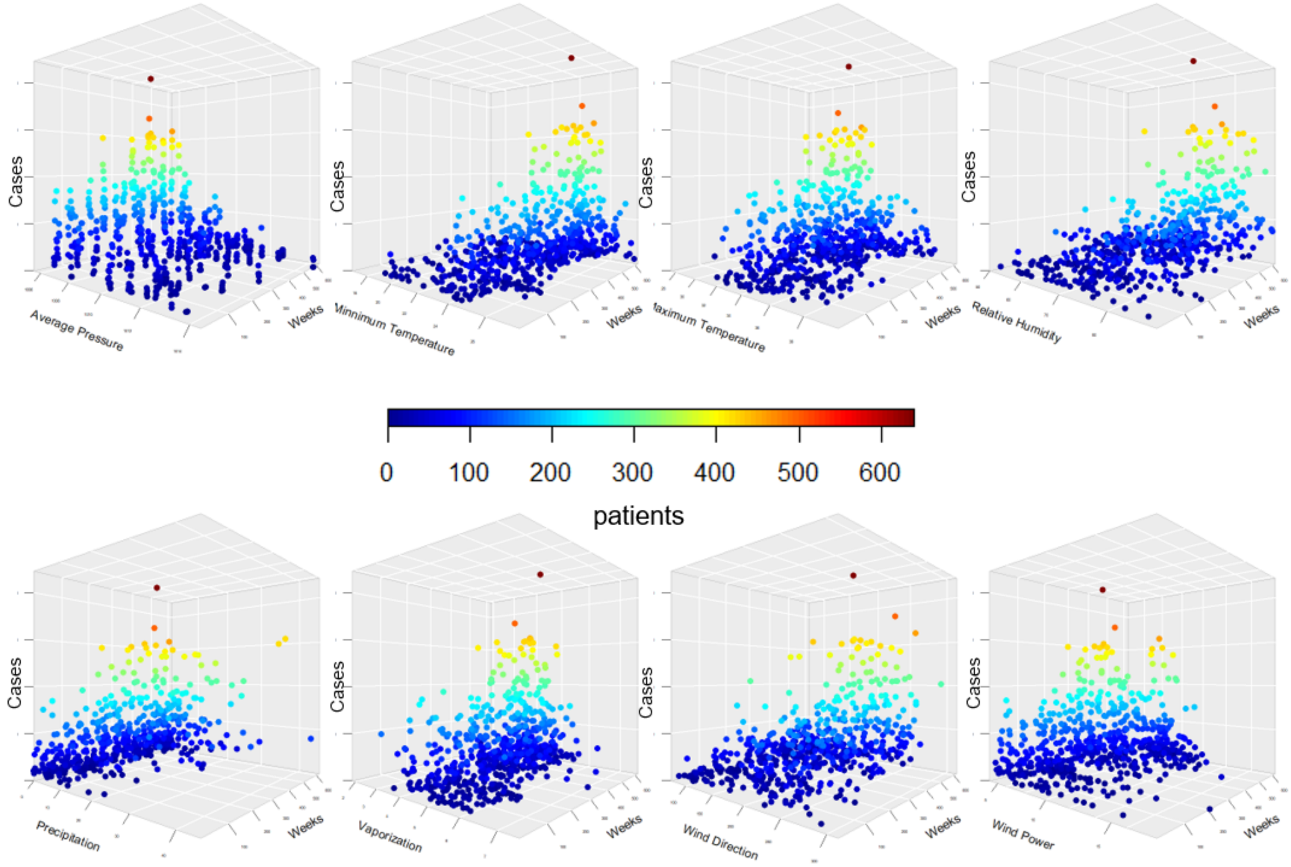

Figure 255: Three-dimensional scatter plot between dengue incidences and weather effects starting from January 2001 to December 2013 of Prachin Buri.

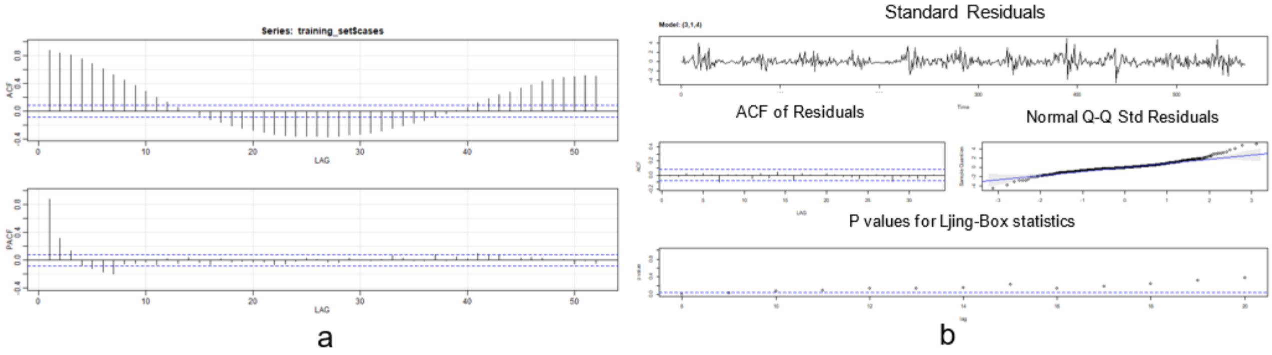

Figure 256: (a) Two plots between lag-time of dengue incidences and ACF and PACF relationship calculated from ARIMA model (b) Summary plots of time series analysis, multiple plots include the plot of predicted model over the time, the plot of ACF residual over lag-time of dengue incidences, residual Q-Q plot of standard residual, and p-value for Ljung-Box statistics of PACF relationship in Prachin Buri over the training data starting from January 2001 to December 2013.

For Prachin Buri, the best model is based on Negative Binomial regression method. The correlation coefficient on the test set in 2014 is 0.759 (95%CI: 0.6982, 0.8198). The significant of the variables associated with p-value statistical calculation are shown in 85. The best model uses 6 variables. The most significant variables are 3-week-lag cases and 3-week-lag average pressure, following by 3-week-lag vaporization and current week wind power. Other variables are, 3-week-lag relative humidity and 1-week-lag wind direction. Time series methods by ARIMA and SARIMA yield the correlation coefficient of -4.742199 and -10.85235 respectively.

Table 85: Comparison table of all methods by the highest correlation coefficient (R^2) and the lowest prediction error (RMSE) in Prachin Buri.

Methods	R-squared (R^2)	Root mean square error (RMSE)
Poisson Regression	0.7445354	2.944393
Negative Binomial Regression	0.75917	2.858813
Quasi-likelihood Regression	0.6501609	3.445598
ARIMA (3,1,4)	-4.742199	13.95949
SARIMA $(2,0,1)(0,2,0)_{52}$	-10.85235	20.05546

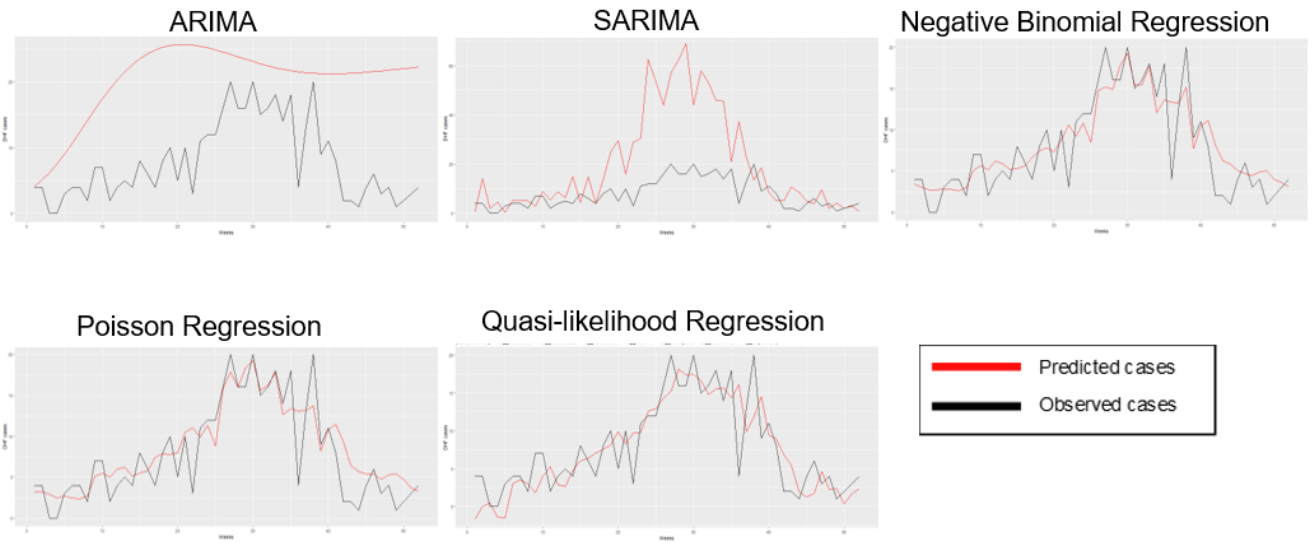

Figure 257: Plots between dengue cases and weeks, the black line represents the observed dengue cases, and the red line represents the predicted dengue cases of the best fit model of each technique over the test set data starting from January 2014 to December 2014.

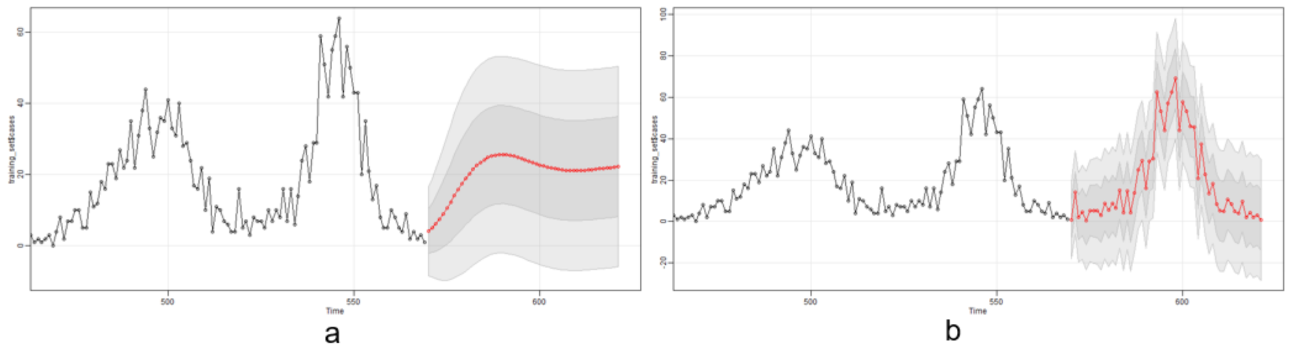

Figure 258: (a) Plot between dengue incidences over weekly time by the best model of ARIMA and (b) SARIMA time series analysis, the black line represents training set data starting from January 2012 to December 2013, and the red line represents the forecasted dengue incidences from January 2014 to December 2014.

Table 86: Coefficients and significant values of best fit GLM models, Negative Binomial, Poisson and Quasi-likelihood regression model of Prachin Buri. The table summarizes coefficients of each independent variables which are composed in best fit model of each method. The significant of each variable is labelled by asterisks under the coefficients. The most important factor is marked as three asterisks which p-value ranges from 0 to 0.001. The second important factor is marked as two asterisks which p-value ranges from 0.001 to 0.01. The third important factor is marked as an asterisk which p-value ranges from 0.01 to 0.1. The least important is also marked as a dot which p-value ranges from 0.1 to 1.

**Independent variables**	**Lag**	**Coefficients/Significant**		
		NB	Poisson	Quasi
Intercept		142.70000 ***	183.7 ***	1198 ***
Cases	1			0.4933 ***
	2			0.2210 ***
	3	0.0329700 ***	0.02552 ***	0.1496 ***
Average Pressure	0			-1.177 ***
	1			
	2			
	3	-0.1403000 ***	-0.1809 ***	
Minimum Temperature	0			
	1			
	2			
	3			
Maximum Temperature	0			
	1			
	2			
	3			
Relative Humidity	0			
	1			-7.380
	2			
	3	0.005450	0.004929 *	
Precipitation	0			
	1			
	2			
	3			
Vaporization	0		0.04675 **	
	1			
	2			
	3	0.0853200 *	0.08255 ***	0.2333
Wind Direction	0			
	1	0.0009157		
	2			
	3			-0.01431 *
Wind Power	0	-0.0320200 *	-0.01849 *	
	1		-0.01204	-0.1490
	2			
	3			

Prachuap Khiri Khan

Prachuap Khiri Khan is located in the central region of Thailand at coordinate of 11°49′N 99°48′E. Prachuap Khiri Khan covers an area of 6,368 km^2. Total population are 525,107 people. The density of population is 82.0 people per km^2. Weather in Prachuap Khiri Khan follows tropical savanna climate system. The highest temperature is in April approximately 40.0°C. The low temperature presents in winter from December to March approximately 15-24.4°C. The monsoon season starts from May through August. October and November are the wettest months (227.8 and 154.5 mm). The highest sunshine hours are in January.

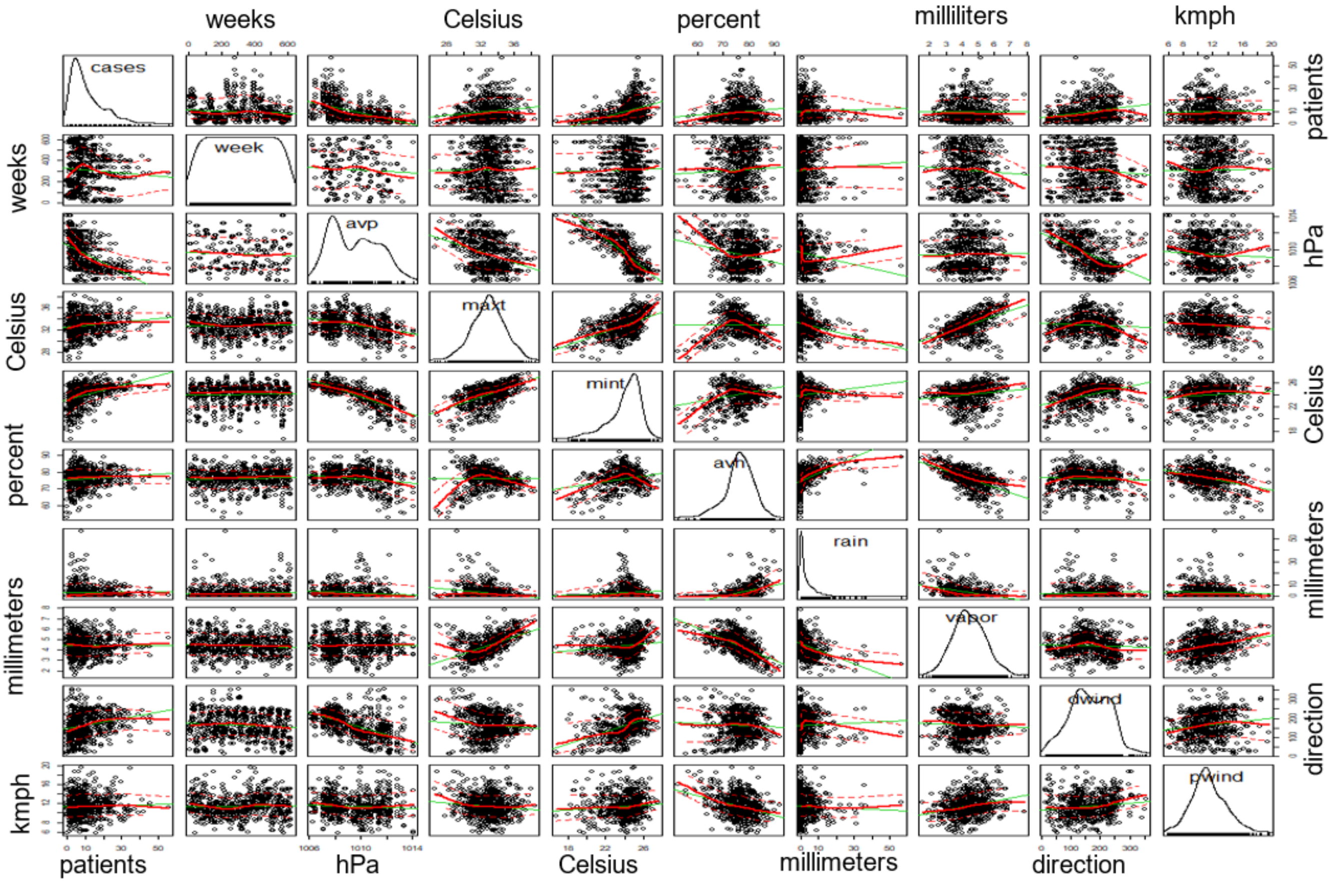

Figure 259: Scatter plot between dengue cases (cases) and selected independent variables, which are the weekly period starting from January 2001 – December 2013 (week), average pressure (avp), maximum temperature (maxt), minimum temperature (mint), average humidity (avh), precipitation (rain), vaporization of water (vapor), wind direction (dwind), and wind power (pwind). The plot visualizes pairwise hundred relationships of training set in Prachuap Khiri Khan.

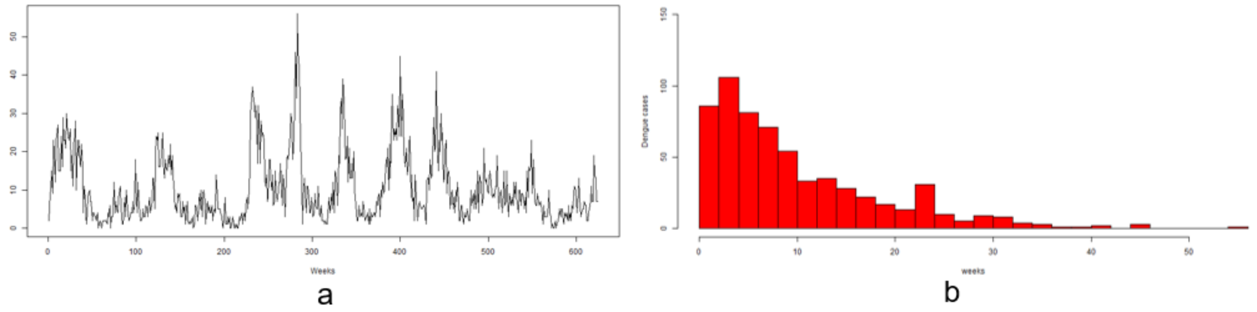

Figure 260: (a) Line plot between dengue incidences and weeks, the plot shows trends of dengue incidences in each year as stationary time series. (b) Histogram of dengue incidences in Prachuap Khiri Khan starting from January 2001 to December 2013 (624 weeks).

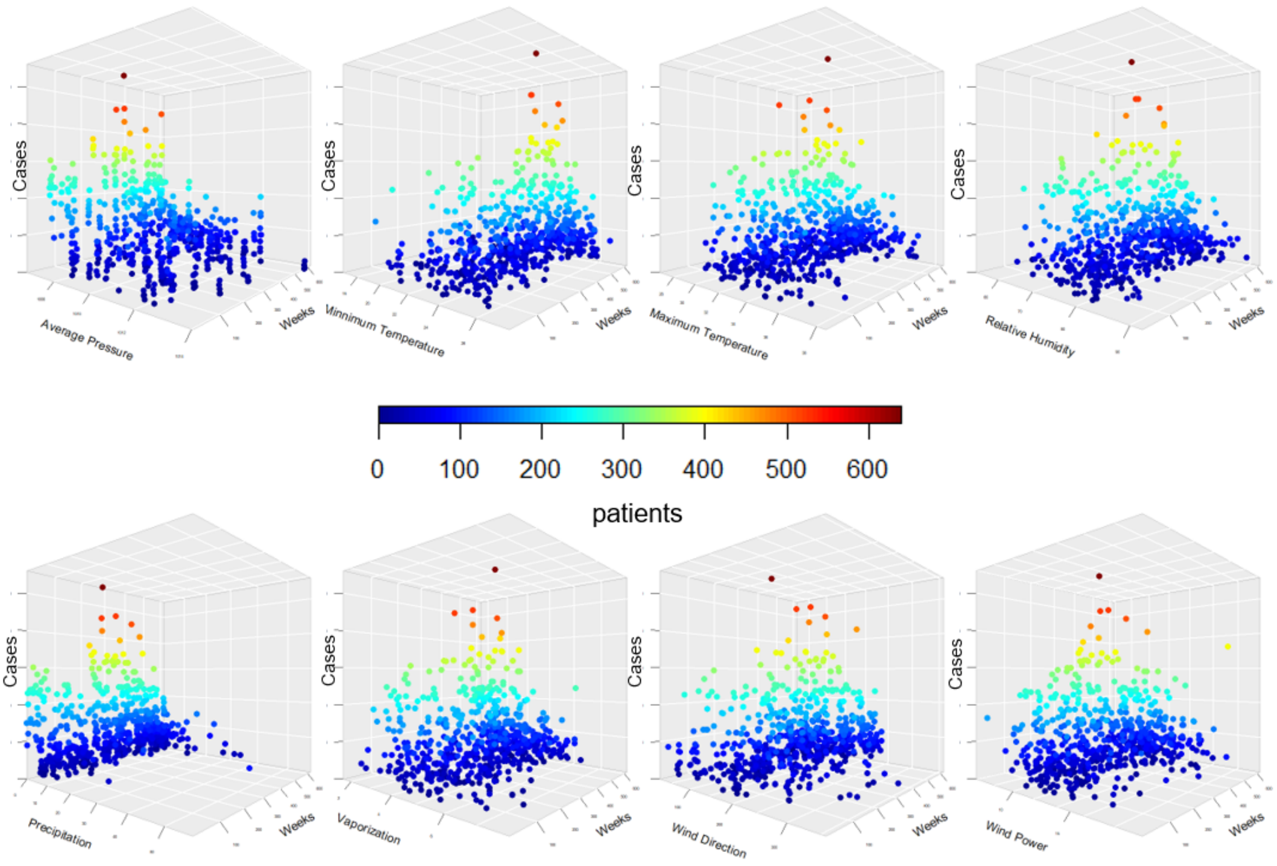

Figure 261: Three-dimensional scatter plot between dengue incidences and weather effects starting from January 2001 to December 2013 of Prachuap Khiri Khan.

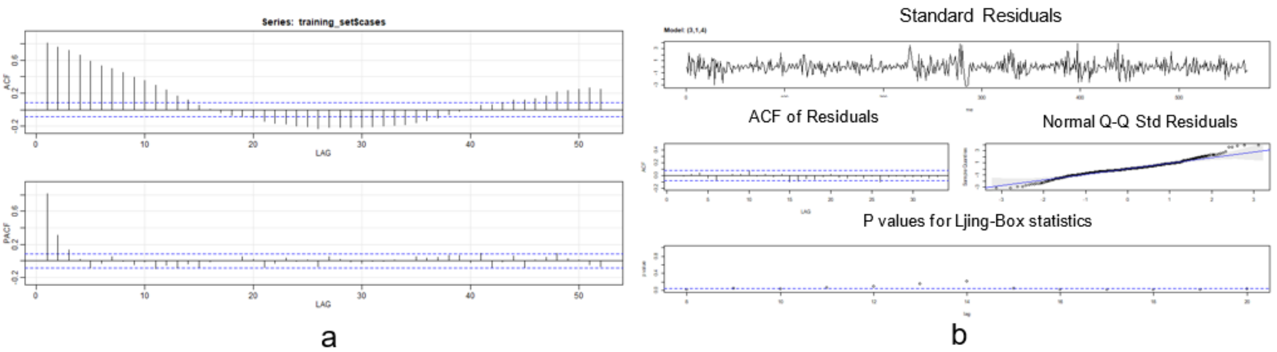

Figure 262: (a) Two plots between lag-time of dengue incidences and ACF and PACF relationship calculated from ARIMA model (b) Summary plots of time series analysis, multiple plots include the plot of predicted model over the time, the plot of ACF residual over lag-time of dengue incidences, residual Q-Q plot of standard residual, and p-value for Ljung-Box statistics of PACF relationship in Prachuap Khiri Khan over the training data starting from January 2001 to December 2013.

For Prachuap Khiri Khan, the best model is based on quasi-likelihood method. The correlation coefficient on the test set in 2014 is 0.481 (95%CI: 0.3864, 0.5756). The best model consists of 8 variables. The most significant variables are 1-week-lag cases, 3-week-lag cases, 1-week-lag average pressure. Other variables are, 3-week-lag minimum temperature, 3-week-lag relative humidity, 2-week-lag wind direction, current week and 3-week-lag wind power. Time series methods by ARIMA and SARIMA yield the correlation coefficient of -1.321327 and -0.8404309 respectively.

Table 87: Comparison table of all methods by the highest correlation coefficient (R^2) and the lowest prediction error (RMSE) in Prachuap Khiri Khan.

**Methods**	**R-squared (R^2)**	**Root mean square error (RMSE)**
Poisson Regression	0.3040017	3.240005
Negative Binomial Regression	0.04232067	3.800595
Quasi-likelihood Regression	0.4809778	2.797915
ARIMA (3,1,4)	-1.321327	5.917111
SARIMA $(2,0,1)(0,2,0)_{52}$	-0.8404309	5.268674

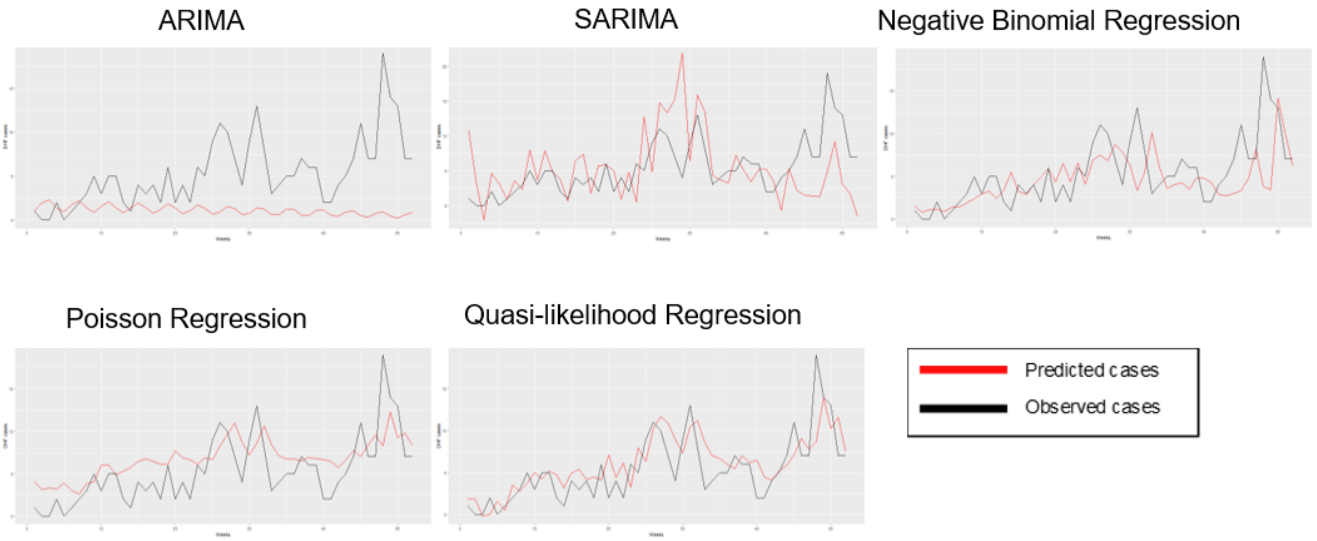

Figure 263: Plots between dengue cases and weeks, the black line represents the observed dengue cases, and the red line represents the predicted dengue cases of the best fit model of each technique over the test set data starting from January 2014 to December 2014.

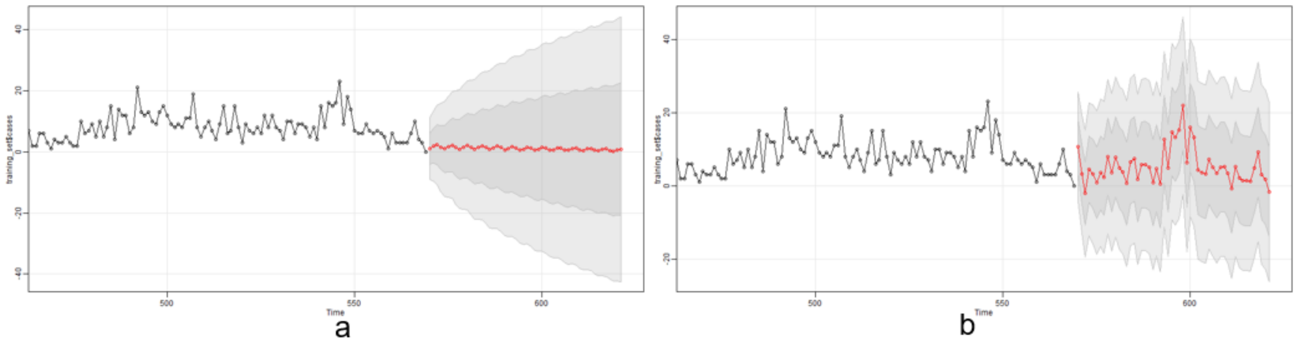

Figure 264: (a) Plot between dengue incidences over weekly time by the best model of ARIMA and (b) SARIMA time series analysis, the black line represents training set data starting from January 2012 to December 2013, and the red line represents the forecasted dengue incidences from January 2014 to December 2014.

Table 88: Coefficients and significant values of best fit GLM models, Negative Binomial, Poisson and Quasi-likelihood regression model of Prachuap Khiri Khan. The table summarizes coefficients of each independent variables which are composed in best fit model of each method. The significant of each variable is labelled by asterisks under the coefficients. The most important factor is marked as three asterisks which p-value ranges from 0 to 0.001. The second important factor is marked as two asterisks which p-value ranges from 0.001 to 0.01. The third important factor is marked as an asterisk which p-value ranges from 0.01 to 0.1. The least important is also marked as a dot which p-value ranges from 0.1 to 1.

**Independent variables**	**Lag**	**Coefficients/Significant**		
		NB	Poisson	Quasi
Intercept		102.183918 *	-1.7115739 ***	783.8581 ***
Cases	1		0.0334367 ***	0.591619 ***
	2	0.111579 ***	0.0197699 ***	
	3			0.214449 ***
Average Pressure	0	-0.104246 *		
	1			-0.771545 ***
	2			
	3			
Minimum Temperature	0			
	1			
	2			
	3		0.0830674 ***	-0.280377
Maximum Temperature	0			
	1			
	2			
	3	0.060884 *		
Relative Humidity	0	-0.004427	0.0014448	
	1			
	2	0.023815 *		
	3		0.0139128 ***	0.054232
Precipitation	0			
	1		-0.0050143 .	
	2			
	3			
Vaporization	0			
	1			
	2	0.094592 *		
	3	-0.012624		
Wind Direction	0			
	1			
	2			-0.004765
	3		-0.0002877	
Wind Power	0			-0.092045
	1			
	2			
	3		0.0206727 ***	0.132583

Ranong

Ranong is in the southern region of Thailand at coordinate of 9°57′43″N 98°38′20″E. Ranong covers an area of 3,298 km^2. Total population are 177,089 people. The density of population is 54.0 people per km^2. General weather in Ranong are under tropical monsoon climate. The temperatures are stable throughout the year, which the highest temperature is in March approximately 39.6°C. Ranong has a short dry season in January to March. The highest rainfall presents in August around 789.1 mm. Humidity is in range from 70-86 percent throughout the year. The highest sunshine hours are in January.

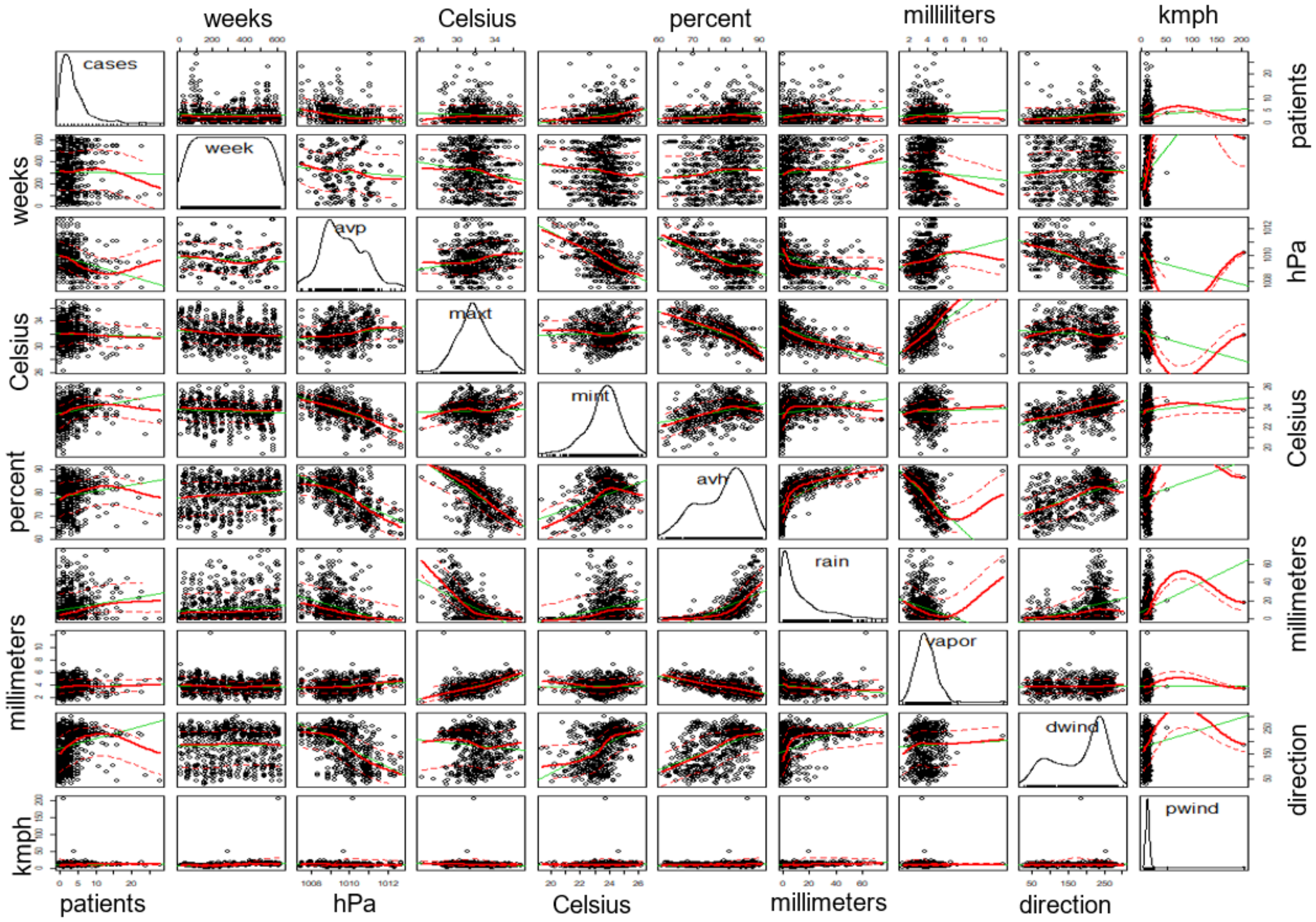

Figure 265: Scatter plot between dengue cases (cases) and selected independent variables, which are the weekly period starting from January 2001 – December 2013 (week), average pressure (avp), maximum temperature (maxt), minimum temperature (mint), average humidity (avh), precipitation (rain), vaporization of water (vapor), wind direction (dwind), and wind power (pwind). The plot visualizes pairwise hundred relationships of training set in Ranong.

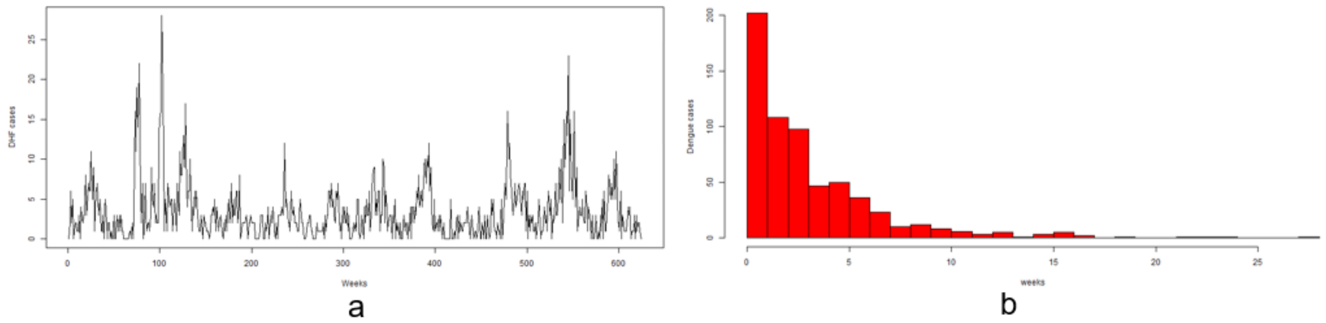

Figure 266: (a) Line plot between dengue incidences and weeks, the plot shows trends of dengue incidences in each year as stationary time series. (b) Histogram of dengue incidences in Ranong starting from January 2001 to December 2013 (624 weeks).

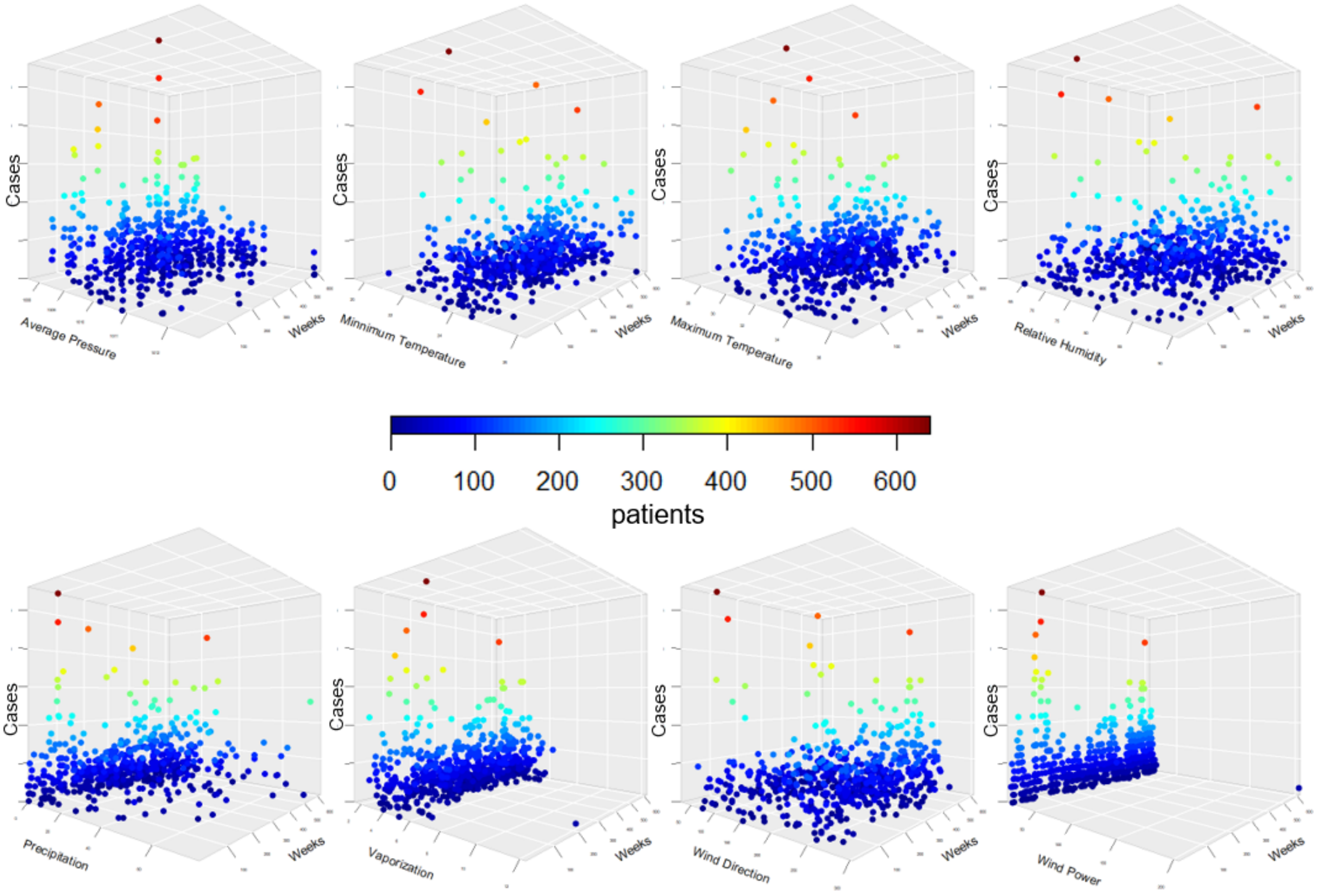

Figure 267: Three-dimensional scatter plot between dengue incidences and weather effects starting from January 2001 to December 2013 of Ranong.

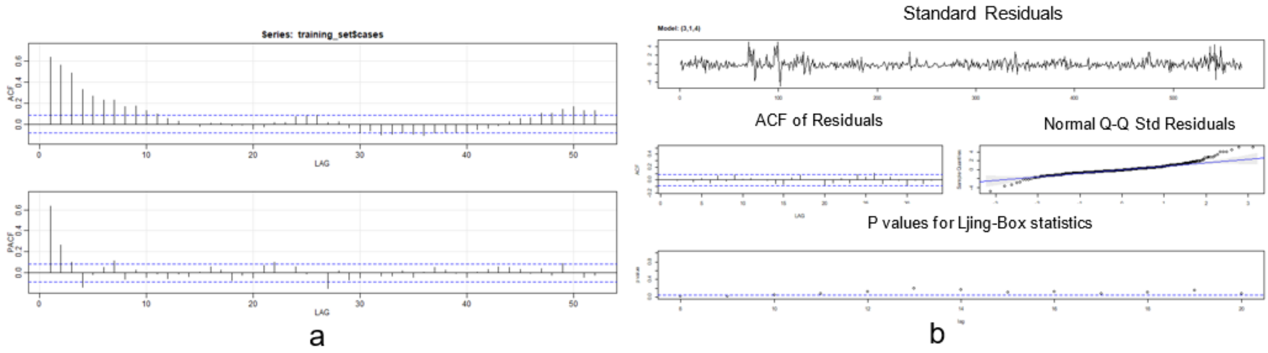

Figure 268: (a) Two plots between lag-time of dengue incidences and ACF and PACF relationship calculated from ARIMA model (b) Summary plots of time series analysis, multiple plots include the plot of predicted model over the time, the plot of ACF residual over lag-time of dengue incidences, residual Q-Q plot of standard residual, and p-value for Ljung-Box statistics of PACF relationship in Ranong over the training data starting from January 2001 to December 2013.

The best model of Ranong is based on Poisson regression method. The correlation coefficient on the test set in 2014 is 0.506 (95%CI: 0.4004, 0.6116). The significant of the variables associated with p-value statistical calculation are shown in Table RAN2. The best model of Ranong uses 8 variables. The most significant variables are 1-week-lag cases, following by 3-week-lag precipitation, 2-week-lag cases, current week minimum temperature and 2-week-lag maximum temperature. Other variables which have less significant are, current week precipitation, wind direction and wind power. Time series methods by ARIMA and SARIMA yield the correlation coeffect of 0.03523283 and -2.742441 respectively.

Table 89: Comparison table of all methods by the highest correlation coefficient (R^2) and the lowest prediction error (RMSE) in Ranong.

Methods	R-squared (R^2)	Root mean square error (RMSE)
Poisson Regression	0.5060206	1.915044
Negative Binomial Regression	0.4834839	1.958242
Quasi-likelihood Regression	0.4249991	2.066135
ARIMA (3,1,4)	0.03523283	2.676306
SARIMA $(2,0,1)(0,2,0)_{52}$	-2.742441	5.271108

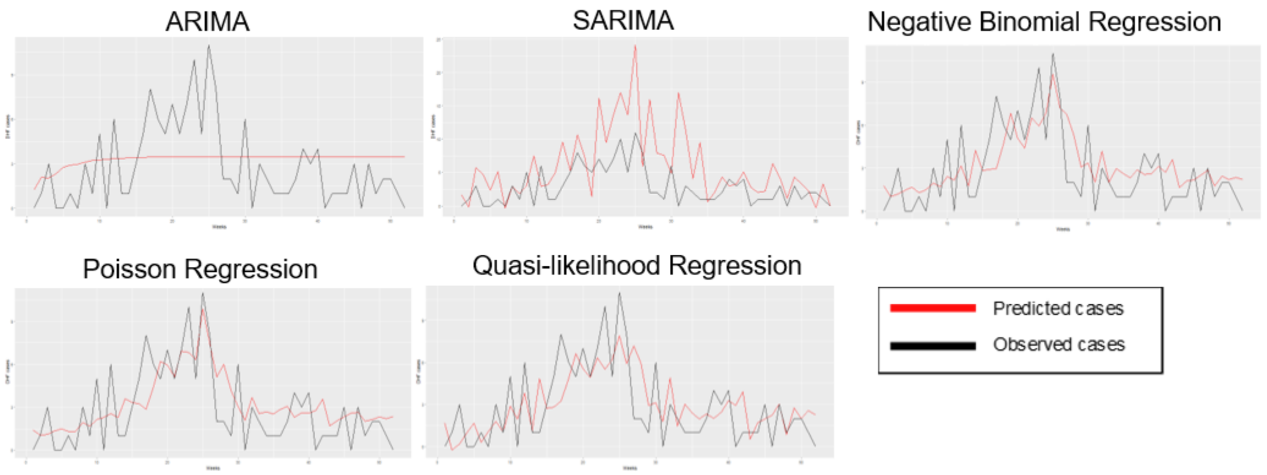

Figure 269: Plots between dengue cases and weeks, the black line represents the observed dengue cases, and the red line represents the predicted dengue cases of the best fit model of each technique over the test set data starting from January 2014 to December 2014.

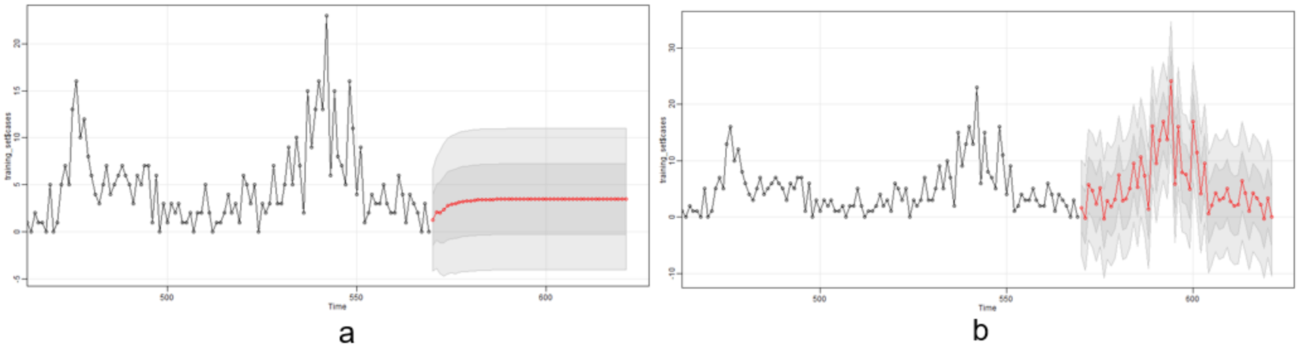

Figure 270: (a) Plot between dengue incidences over weekly time by the best model of ARIMA and (b) SARIMA time series analysis, the black line represents training set data starting from January 2012 to December 2013, and the red line represents the forecasted dengue incidences from January 2014 to December 2014.

Table 90: Coefficients and significant values of best fit GLM models, Negative Binomial, Poisson and Quasi-likelihood regression model of Ranong. The table summarizes coefficients of each independent variables which are composed in best fit model of each method. The significant of each variable is labelled by asterisks under the coefficients. The most important factor is marked as three asterisks which p-value ranges from 0 to 0.001. The second important factor is marked as two asterisks which p-value ranges from 0.001 to 0.01. The third important factor is marked as an asterisk which p-value ranges from 0.01 to 0.1. The least important is also marked as a dot which p-value ranges from 0.1 to 1.

**Independent variables**	**Lag**	**Coefficients/Significant**		
		NB	Poisson	Quasi
Intercept		102.183918 *	104.544925 ***	339.355840 .
Cases	1			
	2	0.111579 ***	0.0699400 ***	0.537387 ***
	3		0.03894900 ***	
Average Pressure	0	-0.104246 *	-0.1078470 ***	-0.342851 *
	1			
	2			
	3			
Minimum Temperature	0			
	1			
	2			
	3			
Maximum Temperature	0			
	1			
	2			
	3	0.060884 *	0.08756300 ***	0.193579 .
Relative Humidity	0	-0.004427		-0.033399
	1	0.023815 *	0.02502900 ***	0.055197
	2			
	3			
Precipitation	0			
	1			
	2			
	3			
Vaporization	0			
	1		0.06551200 **	
	2	0.094592 *	0.05947200 *	0.143593
	3	-0.012624	-0.046767	-0.098719
Wind Direction	0			
	1			
	2			
	3			0.001711
Wind Power	0			
	1			
	2			
	3			

Ratchaburi

Ratchaburi is located in the central region of Thailand at coordinate of 13°32′08″N 99°48′48″E. Ratchaburi covers an area of 5,196 km^2. Total population are 853,217 people. The density of population is 164.0 people per km^2. Weather in Ratchaburi are under tropical wet and dry climate. The highest temperature is in April approximately 40.0°C. The low temperature presents in winter from December to March. The monsoon season starts from May through August. The highest rainfall presents in September. The highest sunshine hours are in March.

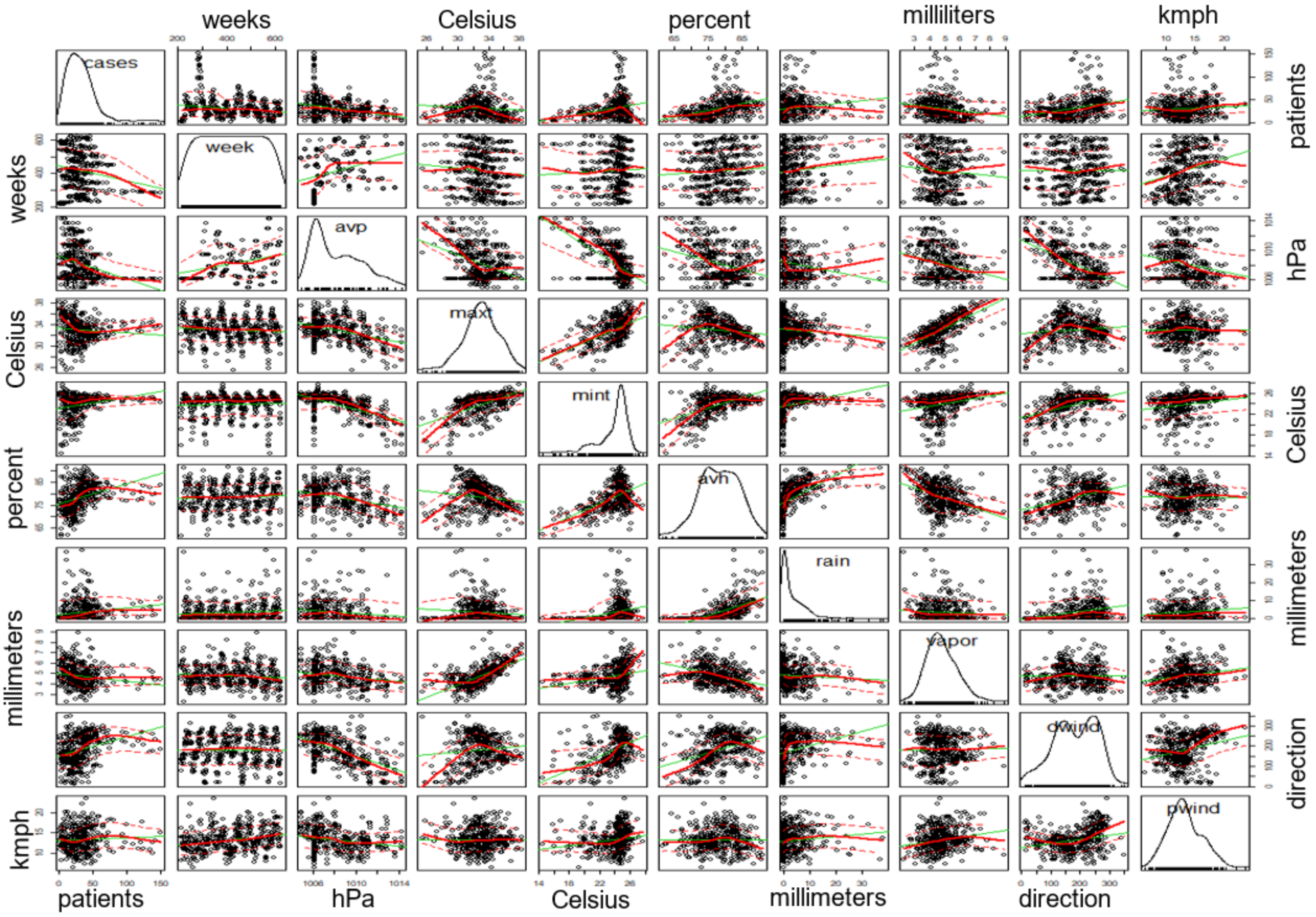

Figure 271: Scatter plot between dengue cases (cases) and selected independent variables, which are the weekly period starting from January 2001 – December 2013 (week), average pressure (avp), maximum temperature (maxt), minimum temperature (mint), average humidity (avh), precipitation (rain), vaporization of water (vapor), wind direction (dwind), and wind power (pwind). The plot visualizes pairwise hundred relationships of training set in Ratchaburi.

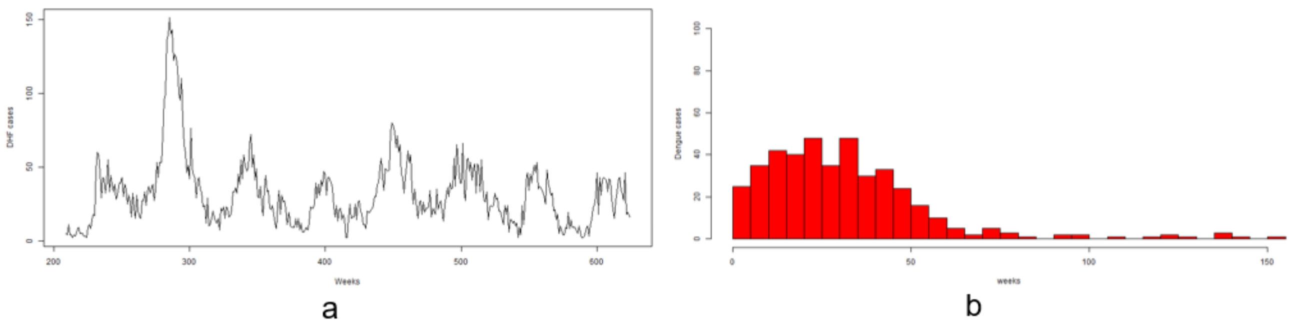

Figure 272: (a) Line plot between dengue incidences and weeks, the plot shows trends of dengue incidences in each year as stationary time series. (b) Histogram of dengue incidences in Ratchaburi starting from January 2001 to December 2013 (624 weeks).

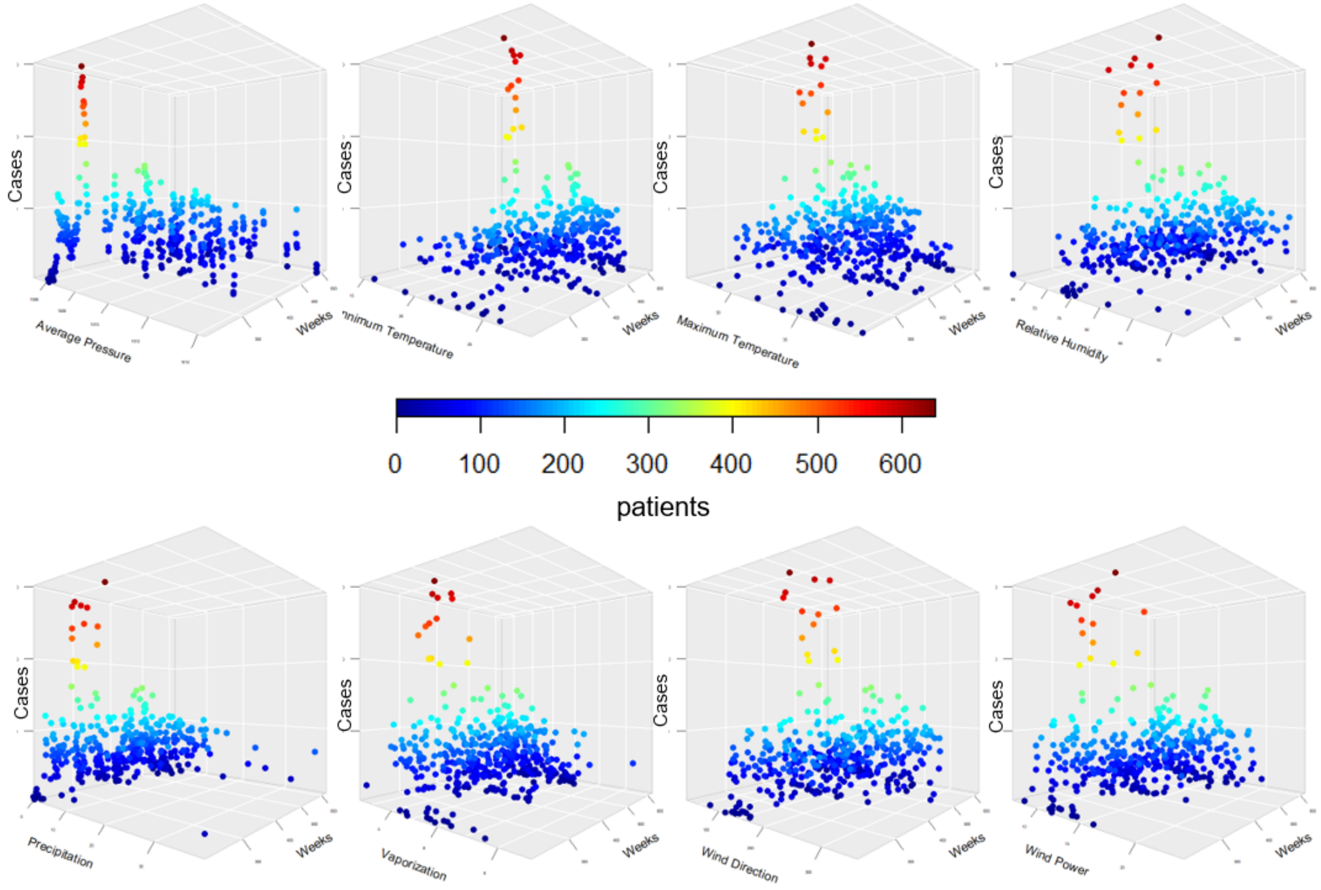

Figure 273: Three-dimensional scatter plot between dengue incidences and weather effects starting from January 2001 to December 2013 of Ratchaburi.

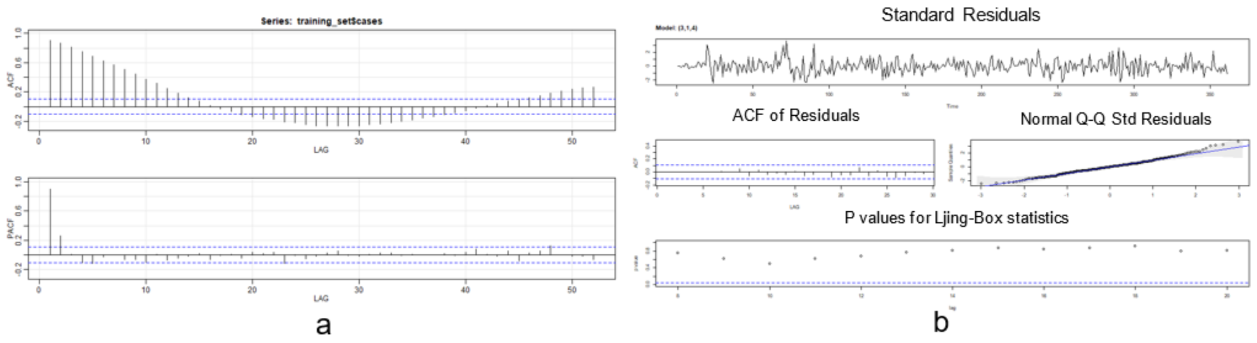

Figure 274: (a) Two plots between lag-time of dengue incidences and ACF and PACF relationship calculated from ARIMA model (b) Summary plots of time series analysis, multiple plots include the plot of predicted model over the time, the plot of ACF residual over lag-time of dengue incidences, residual Q-Q plot of standard residual, and p-value for Ljung-Box statistics of PACF relationship in Ratchaburi over the training data starting from January 2001 to December 2013.

For Ratchaburi, the best model is based on quasi-likelihood method. The correlation coefficient on the test set in 2014 is 0.739 (94%CI: 0.6841, 0.7939). The best model of Ratchaburi uses 9 variables. The most significant variables are 1-week-lag cases and 2-week-lag cases, following by current week average pressure, current week maximum temperature and 3-week-lag vaporization. Other variables are 2-week-lag cases, current week minimum temperature and 2-week-lag maximum temperature, current week vaporization, 2-week-lag wind direction and 3-week-lag wind power. Time series methods by ARIMA and SARIMA yield the correlation coefficient of -1.006417 and 0.39787030 respectively.

Table 91: Comparison table of all methods by the highest correlation coefficient (R^2) and the lowest prediction error (RMSE) in Ratchaburi.

Methods	R-squared (R^2)	Root mean square error (RMSE)
Poisson Regression	0.6832631	8.045005
Negative Binomial Regression	0.7123263	7.667027
Quasi-likelihood Regression	0.7395857	7.294732
ARIMA (3,1,4)	-1.006417	20.24825
SARIMA $(2,0,1)(0,2,0)_{52}$	0.3978703	11.09231

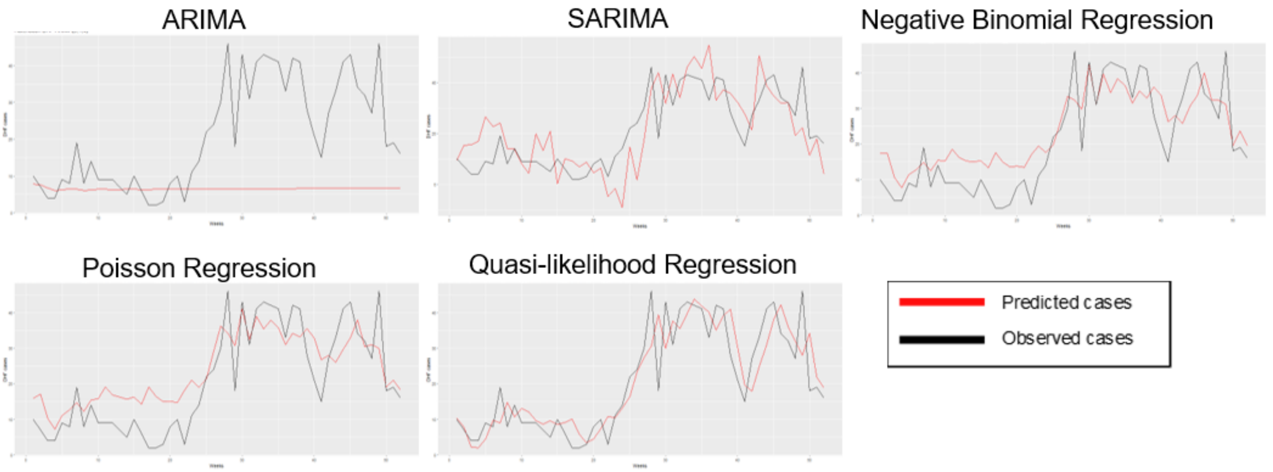

Figure 275: Plots between dengue cases and weeks, the black line represents the observed dengue cases, and the red line represents the predicted dengue cases of the best fit model of each technique over the test set data starting from January 2014 to December 2014.

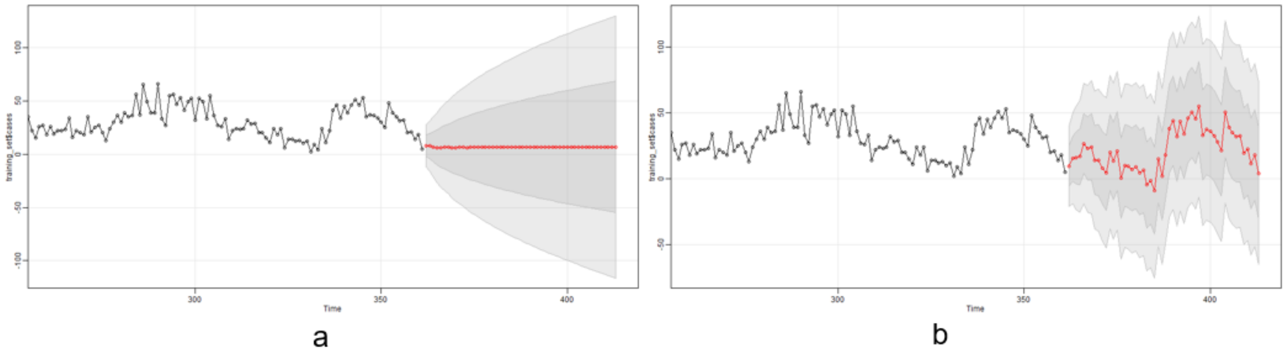

Figure 276: (a) Plot between dengue incidences over weekly time by the best model of ARIMA and (b) SARIMA time series analysis, the black line represents training set data starting from January 2012 to December 2013, and the red line represents the forecasted dengue incidences from January 2014 to December 2014.

Table 92: Coefficients and significant values of best fit GLM models, Negative Binomial, Poisson and Quasi-likelihood regression model of Ratchaburi. The table summarizes coefficients of each independent variables which are composed in best fit model of each method. The significant of each variable is labelled by asterisks under the coefficients. The most important factor is marked as three asterisks which p-value ranges from 0 to 0.001. The second important factor is marked as two asterisks which p-value ranges from 0.001 to 0.01. The third important factor is marked as an asterisk which p-value ranges from 0.01 to 0.1. The least important is also marked as a dot which p-value ranges from 0.1 to 1.

**Independent variables**	**Lag**	**Coefficients/Significant**		
		NB	Poisson	Quasi
Intercept		52.5497729 ***	65.290675 ***	984.99544 **
Cases	1			0.6204430 ***
	2	0.0166612 ***	0.014133 ***	0.2860640 ***
	3			
Average Pressure	0			-0.978821 **
	1			
	2			
	3	-0.0486801 ***	-0.061399 ***	
Minimum Temperature	0	0.0851299 ***	0.089369 ***	
	1			
	2			
	3			
Maximum Temperature	0			0.7433340 *
	1			
	2	-0.0425665 **	-0.038937 ***	-0.301423
	3	-0.0244931	-0.025485 **	
Relative Humidity	0			
	1			
	2			
	3			
Precipitation	0			
	1			
	2			
	3			
Vaporization	0	-0.0377815 .	-0.028859 **	-1.014331
	1			
	2			
	3	-0.0647620 *	-0.066687 ***	-1.150076 .
Wind Direction	0			
	1			
	2			-0.006000
	3			
Wind Power	0			
	1		-0.003595	
	2			
	3			0.133220

Rayong

Rayong is located in the southern continent of Thailand at coordinate of 12°40′27″N 101°16′44″E. Rayong covers an area of 3,552 km^2. Total population are 674,393 people. The density of population is 190.0 people per km^2. General weather in Rayong are under tropical monsoon climate. The highest temperature is in April approximately 40.0°C. Temperatures are stable throughout the year. Rayong has a short dry season in February and March. The highest rainfall presents in September around 255.2 mm. Humidity is in range from 74-82 percent throughout the year. The highest sunshine hours are in January.

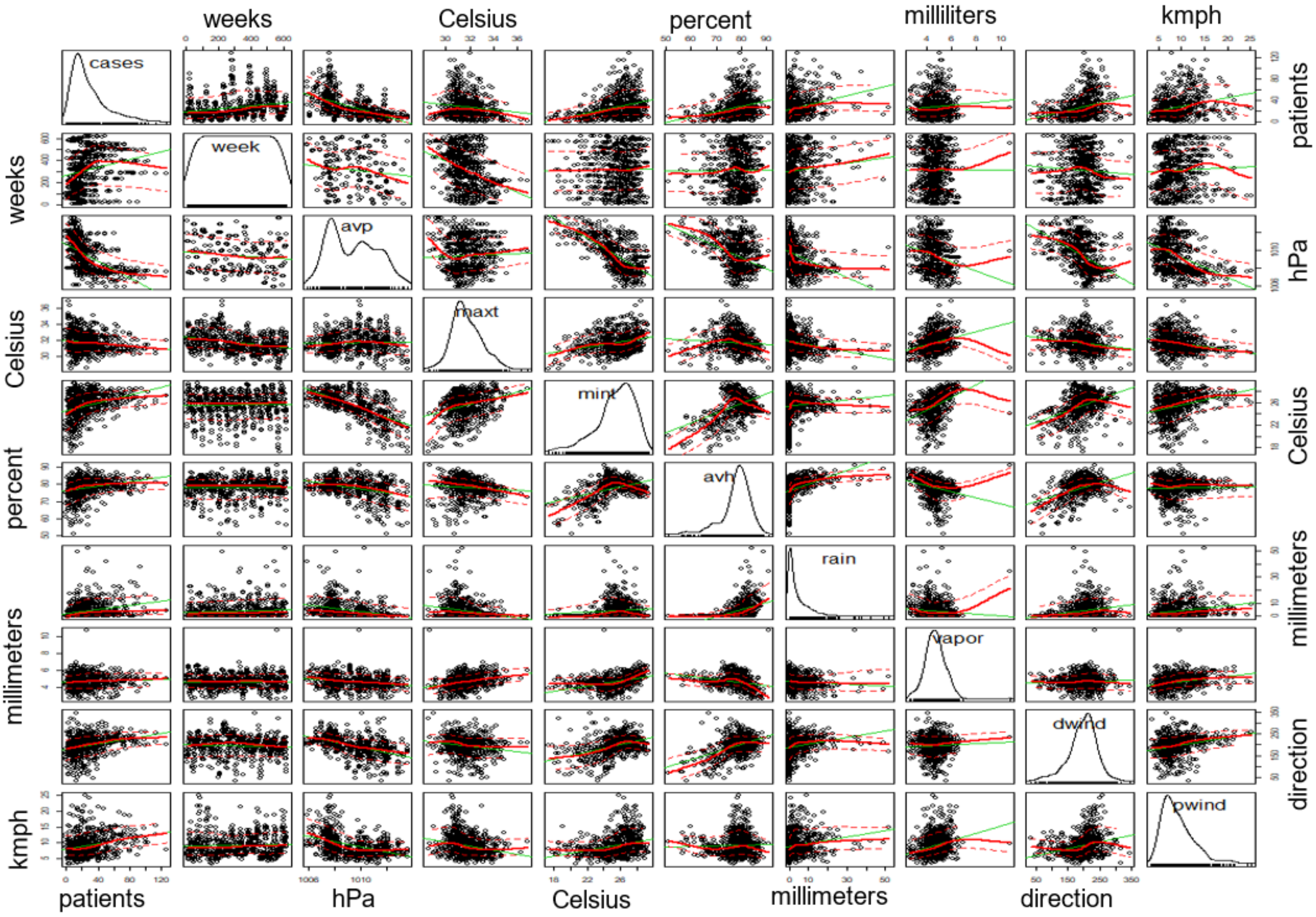

Figure 277: Scatter plot between dengue cases (cases) and selected independent variables, which are the weekly period starting from January 2001 – December 2013 (week), average pressure (avp), maximum temperature (maxt), minimum temperature (mint), average humidity (avh), precipitation (rain), vaporization of water (vapor), wind direction (dwind), and wind power (pwind). The plot visualizes pairwise hundred relationships of training set in Rayong.

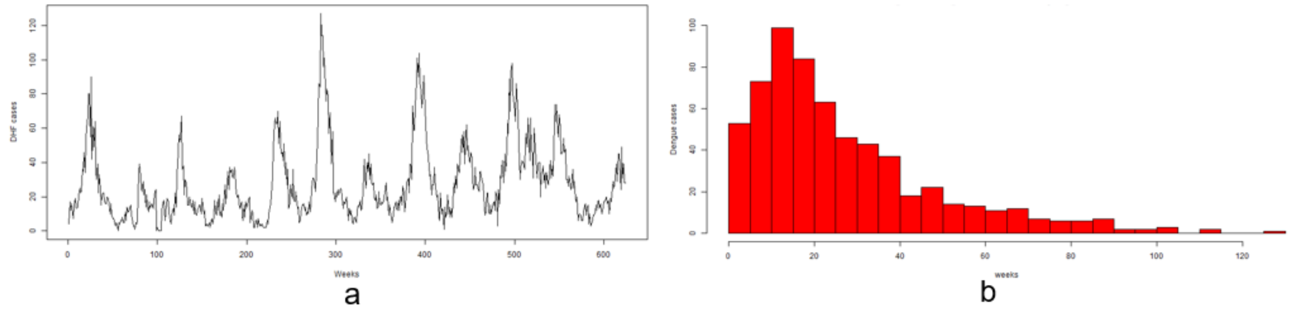

Figure 278: (a) Line plot between dengue incidences and weeks, the plot shows trends of dengue incidences in each year as stationary time series. (b) Histogram of dengue incidences in Rayong starting from January 2001 to December 2013 (624 weeks).

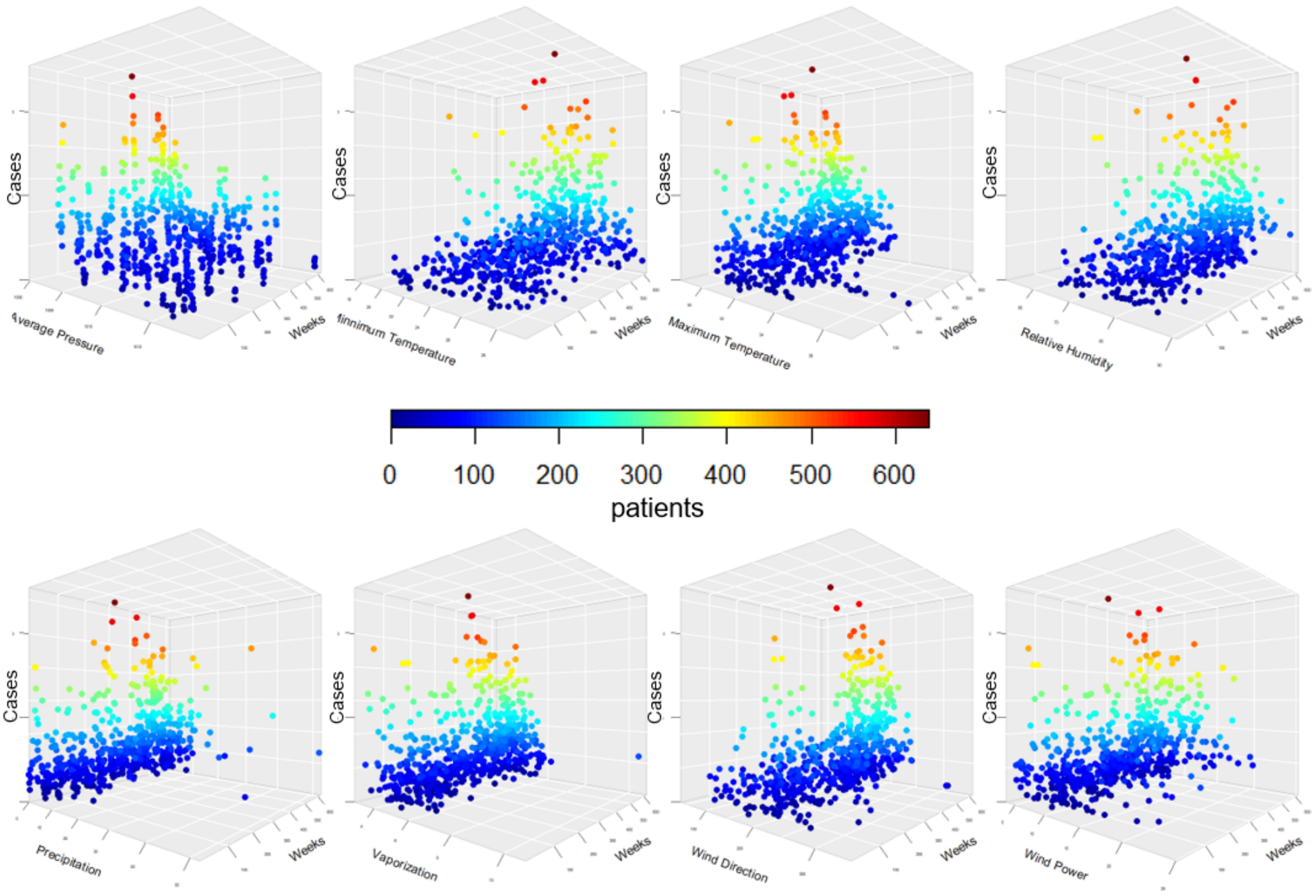

Figure 279: Three-dimensional scatter plot between dengue incidences and weather effects starting from January 2001 to December 2013 of rayong.

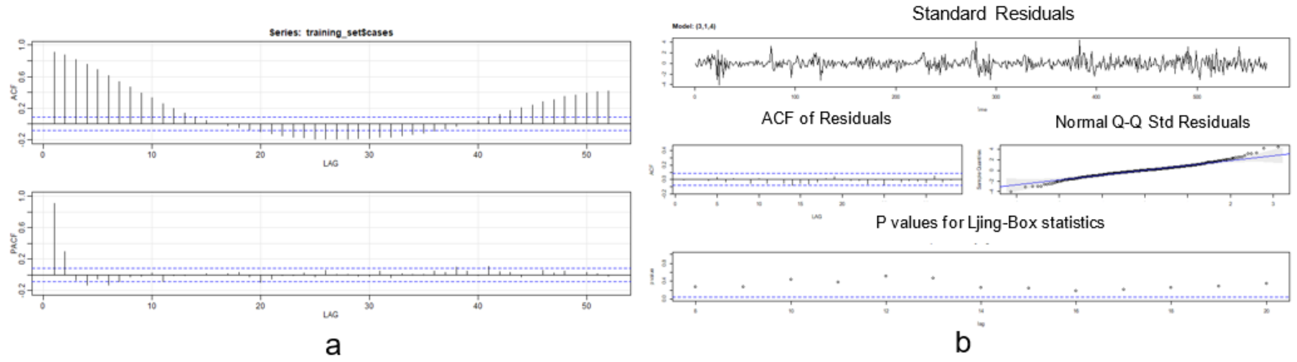

Figure 280: (a) Two plots between lag-time of dengue incidences and ACF and PACF relationship calculated from ARIMA model (b) Summary plots of time series analysis, multiple plots include the plot of predicted model over the time, the plot of ACF residual over lag-time of dengue incidences, residual Q-Q plot of standard residual, and p-value for Ljung-Box statistics of PACF relationship in Rayong over the training data starting from January 2001 to December 2013.

For Rayong, the best model is based on Negative Binomial regression method. The correlation coefficient on the test set in 2014 is 0.714 (95%CI: 0.6389, 0.7890). The significant of the variables associated with p-value statistical calculation are shown in Table RAY2. The model of Rayong uses 6 variables. The most significant variables are 2-week-lag cases, following by 1-week-lag relative humidity, 3-week-lag cases, 2-week-lag maximum temperature. Other variables which have less significant are, 2-week-lag precipitation, and 2-week-lag wind power. Time series methods by ARIMA and SARIMA yield the correlation coefficient of -1.682122 and -2.585119 respectively.

Table 93: Comparison table of all methods by the highest correlation coefficient (R^2) and the lowest prediction error (RMSE) in Rayong.

Methods	R-squared (R^2)	Root mean square error (RMSE)
Poisson Regression	0.6836268	6.404242
Negative Binomial Regression	0.7144017	6.084792
Quasi-likelihood Regression	0.6790041	6.45086
ARIMA (3,1,4)	-1.682122	18.64692
SARIMA $(2,0,1)(0,2,0)_{52}$	-2.585119	21.55856

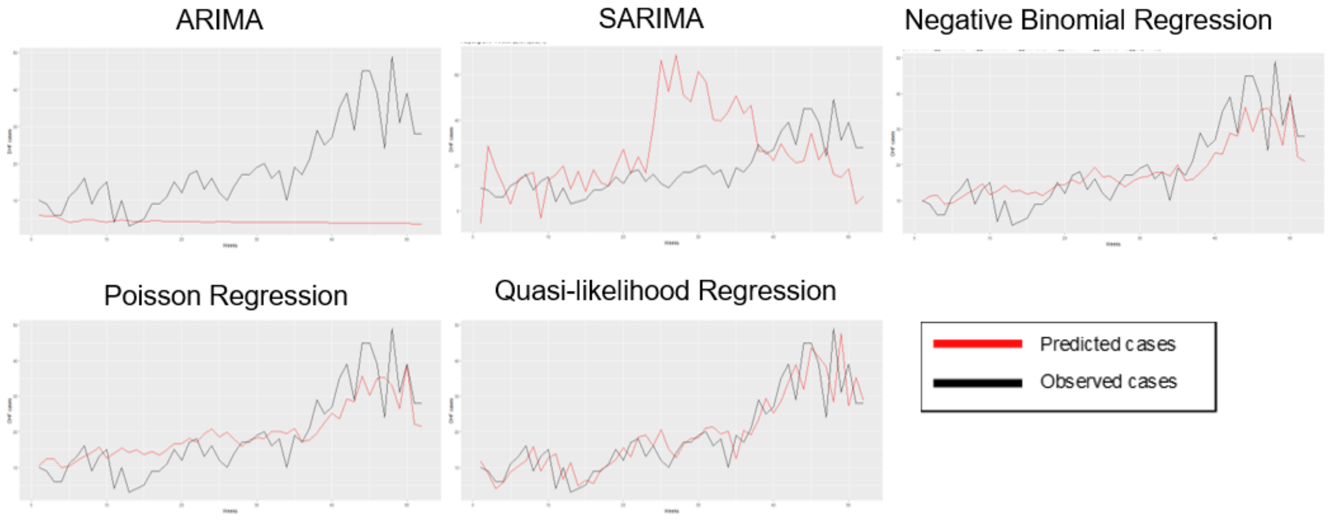

Figure 281: Plots between dengue cases and weeks, the black line represents the observed dengue cases, and the red line represents the predicted dengue cases of the best fit model of each technique over the test set data starting from January 2014 to December 2014.

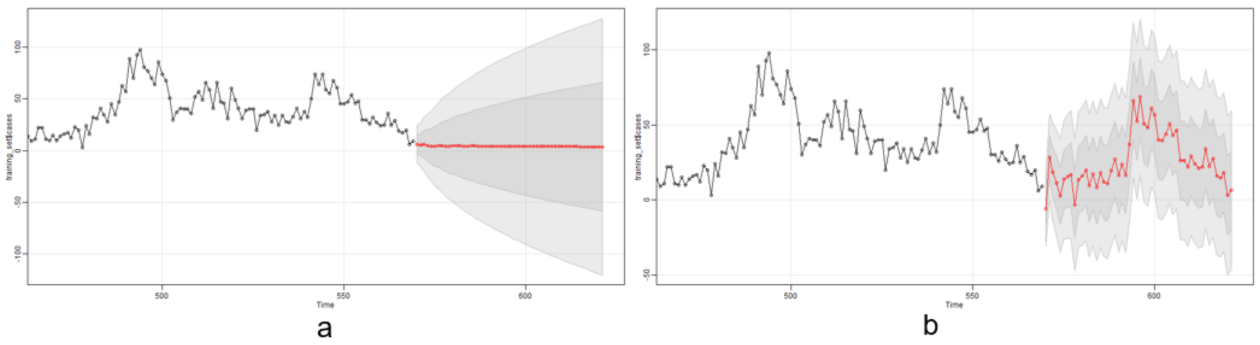

Figure 282: (a) Plot between dengue incidences over weekly time by the best model of ARIMA and (b) SARIMA time series analysis, the black line represents training set data starting from January 2012 to December 2013, and the red line represents the forecasted dengue incidences from January 2014 to December 2014.

Table 94: Coefficients and significant values of best fit GLM models, Negative Binomial, Poisson and Quasi-likelihood regression model of Rayong. The table summarizes coefficients of each independent variables which are composed in best fit model of each method. The significant of each variable is labelled by asterisks under the coefficients. The most important factor is marked as three asterisks which p-value ranges from 0 to 0.001. The second important factor is marked as two asterisks which p-value ranges from 0.001 to 0.01. The third important factor is marked as an asterisk which p-value ranges from 0.01 to 0.1. The least important is also marked as a dot which p-value ranges from 0.1 to 1.

**Independent variables**	**Lag**	**Coefficients/Significant**		
		NB	Poisson	Quasi
Intercept		-0.722142	-1.0730036 ***	-30.721882 *
Cases	1			0.804538 ***
	2	0.021535 ***	0.0181051 ***	
	3	0.005612 **	0.0039093 ***	0.096935 *
Average Pressure	0			
	1			
	2			
	3			
Minimum Temperature	0			
	1			
	2			
	3			
Maximum Temperature	0		0.0130936	
	1			
	2	0.054362 **	0.0588573 ***	
	3			0.512020
Relative Humidity	0			0.248801 **
	1	0.016485 ***	0.0154476 ***	-0.053344
	2			-0.002525
	3			
Precipitation	0			
	1			-0.002819
	2	0.004148	0.0025987 *	
	3			
Vaporization	0			
	1			
	2			
	3			
Wind Direction	0			
	1			
	2			
	3			
Wind Power	0			
	1			0.241829 *
	2	0.007087	0.0136683 ***	
	3			

Roi Et

Roi Et is a province located in the northeastern continent of Thailand at coordinate of 16°03′11″N 103°39′04″E. Roi Et covers an area of 8,299 km^2. Total population are 1,308,318 people. The density of population is approximately 158.0 people per km^2. Weather in Roi Et has tropical savanna climate under the South Asian monsoon system. High temperature is in April approximately 41.2°C and starts to low temperature from December to February (2.1-19.2°C). Winters are dry and warm. The monsoon season begins from May through October. The highest rainfall presents in August around 252.2 mm. Humidity is in range from 61-77 percent throughout the year. The highest sunshine hours are in January.

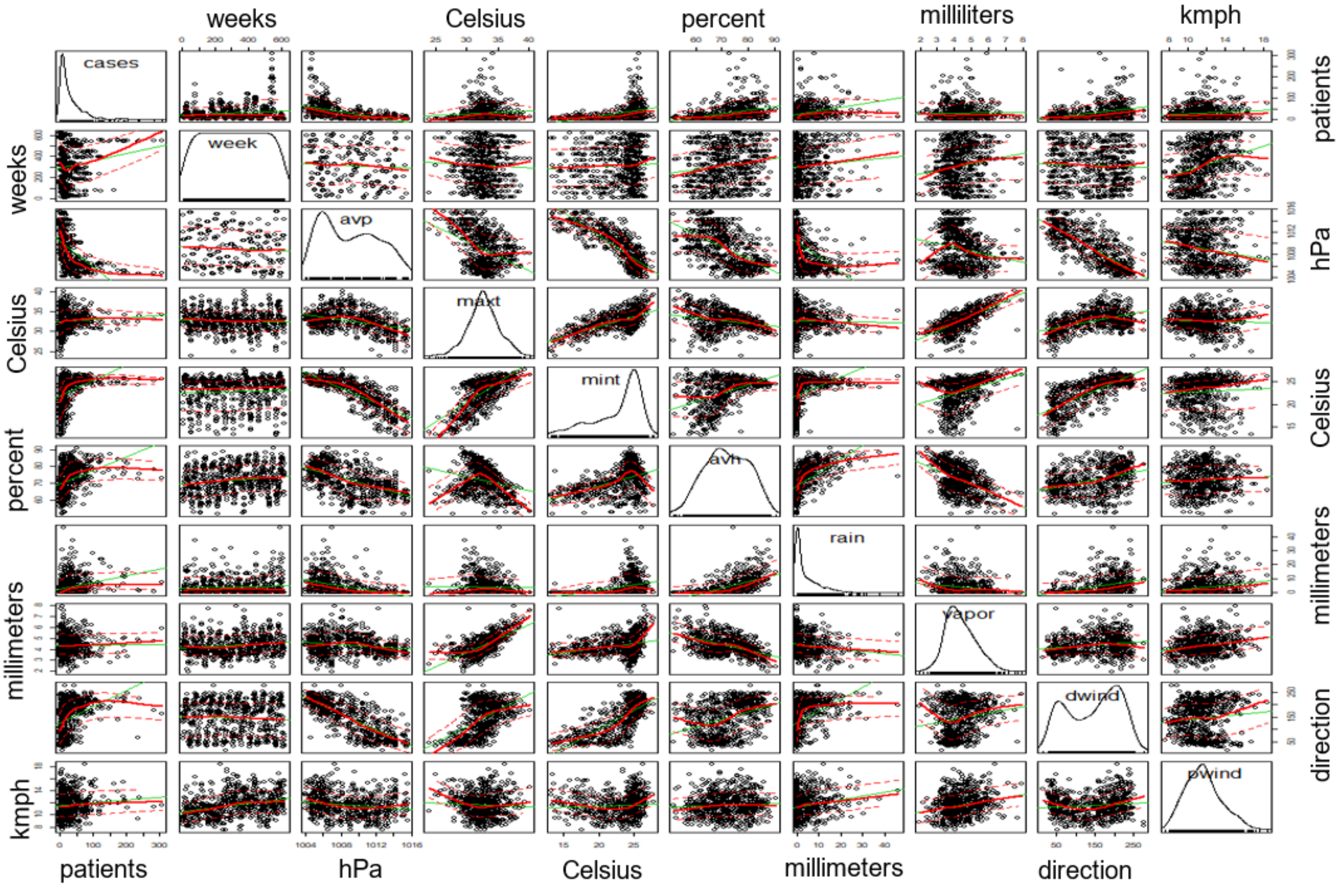

Figure 283: Scatter plot between dengue cases (cases) and selected independent variables, which are the weekly period starting from January 2001 – December 2013 (week), average pressure (avp), maximum temperature (maxt), minimum temperature (mint), average humidity (avh), precipitation (rain), vaporization of water (vapor), wind direction (dwind), and wind power (pwind). The plot visualizes pairwise hundred relationships of training set in Roiet.

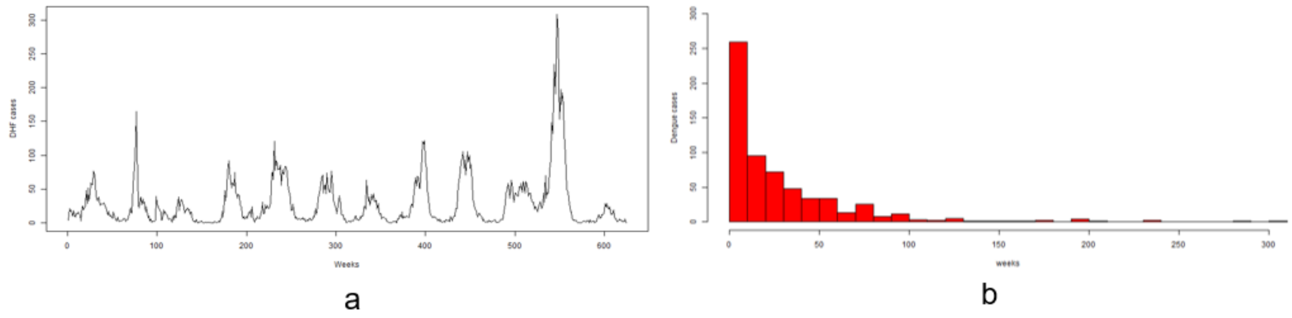

Figure 284: (a) Line plot between dengue incidences and weeks, the plot shows trends of dengue incidences in each year as stationary time series. (b) Histogram of dengue incidences in Roiet starting from January 2001 to December 2013 (624 weeks).

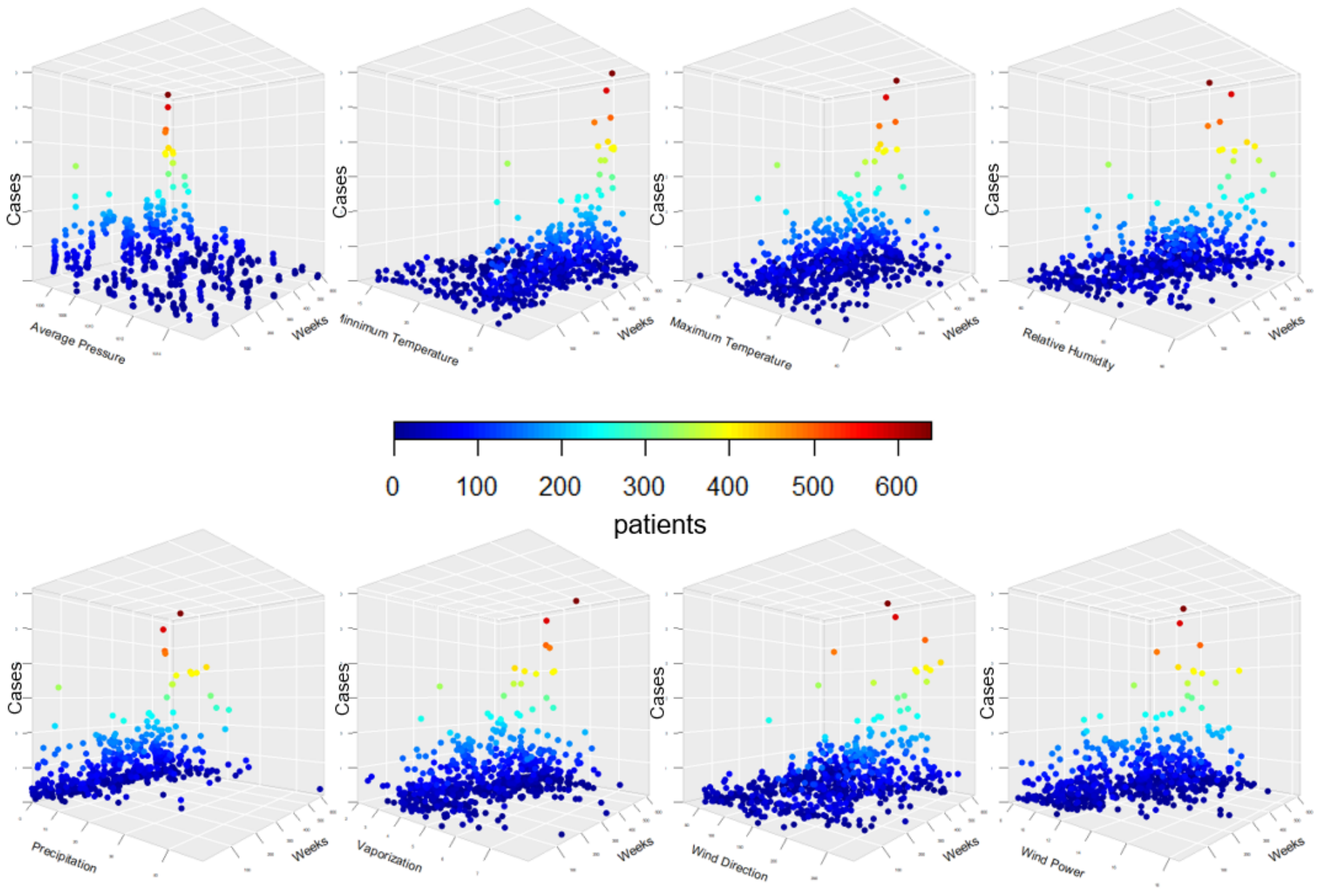

Figure 285: Three-dimensional scatter plot between dengue incidences and weather effects starting from January 2001 to December 2013 of Roiet.

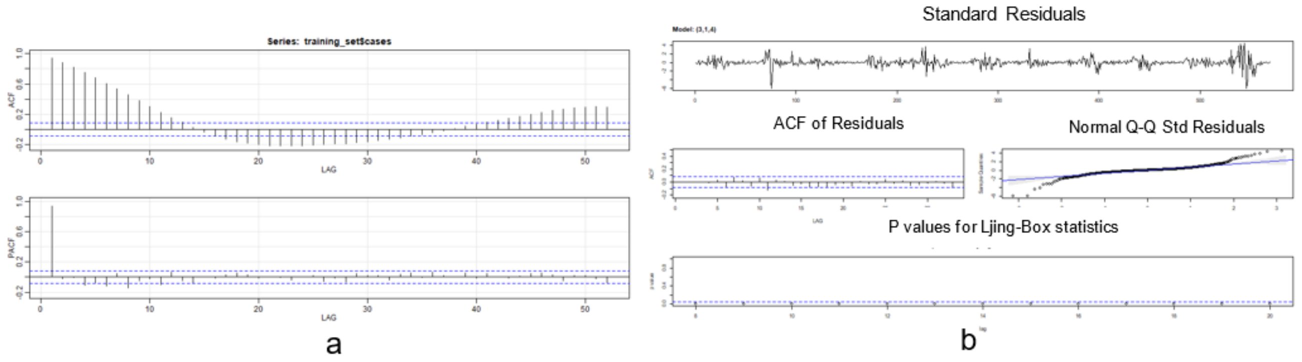

Figure 286: (a) Two plots between lag-time of dengue incidences and ACF and PACF relationship calculated from ARIMA model (b) Summary plots of time series analysis, multiple plots include the plot of predicted model over the time, the plot of ACF residual over lag-time of dengue incidences, residual Q-Q plot of standard residual, and p-value for Ljung-Box statistics of PACF relationship in Roiet over the training data starting from January 2001 to December 2013.

The best model of Roiet is based on quasi-likelihood method. The correlation coefficient on the test set in 2014 is 0.724 (95%CI: 0.6746, 0.8349). The best model of roiet uses 8 variables. The most significant variables are 1-week-lag cases, following by 3-week-lag precipitation, 2-week-lag cases, current week minimum temperature and 2-week-lag maximum temperature. Other variables which have less significant are, current week precipitation, wind direction and wind power. Time series methods by ARIMA and SARIMA yield the correlation coefficient of 0.288 and unpredictable respectively.

Table 95: Comparison table of all methods by the highest correlation coefficient (R^2) and the lowest prediction error (RMSE) in Roiet.

**Methods**	**R-squared (R^2)**	**Root mean square error (RMSE)**
Poisson Regression	-1.4985	12.37693
Negative Binomial Regression	0.3222446	6.446288
Quasi-likelihood Regression	0.7549926	3.875813
ARIMA (3,1,4)	-1.200983	11.61667
SARIMA $(2,0,1)(0,2,0)_{52}$	-203.1175	111.8699

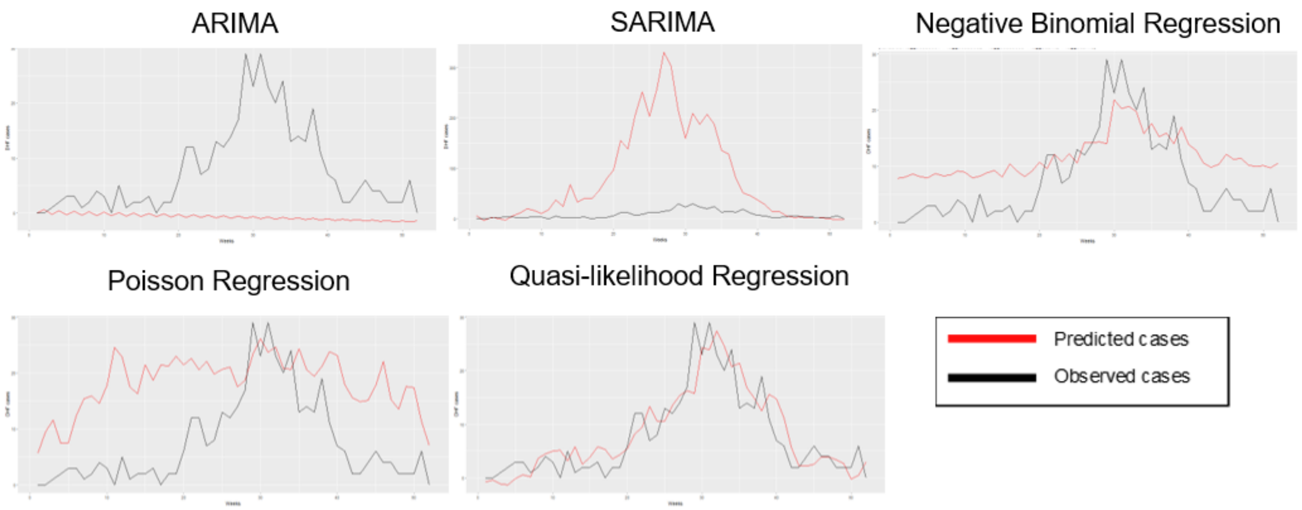

Figure 287: Plots between dengue cases and weeks, the black line represents the observed dengue cases, and the red line represents the predicted dengue cases of the best fit model of each technique over the test set data starting from January 2014 to December 2014.

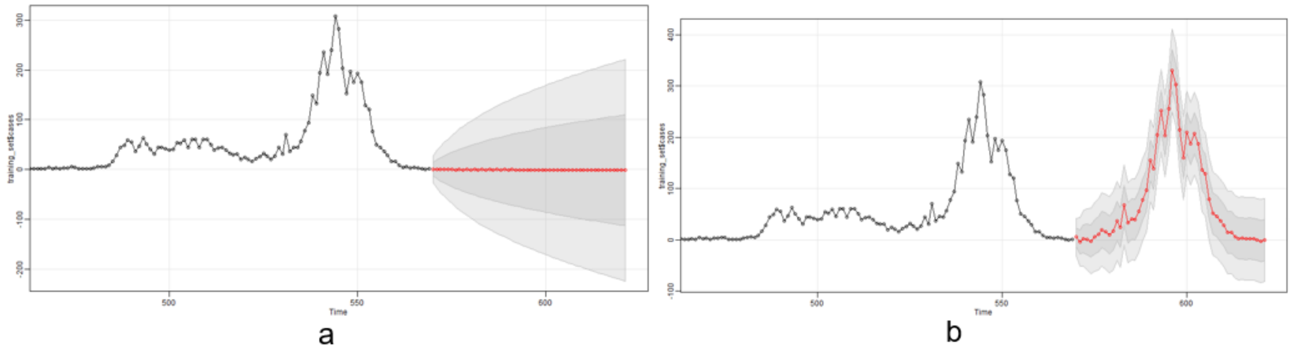

Figure 288: (a) Plot between dengue incidences over weekly time by the best model of ARIMA and (b) SARIMA time series analysis, the black line represents training set data starting from January 2012 to December 2013, and the red line represents the forecasted dengue incidences from January 2014 to December 2014.

Table 96: Coefficients and significant values of best fit GLM models, Negative Binomial, Poisson and Quasi-likelihood regression model of Roiet. The table summarizes coefficients of each independent variables which are composed in best fit model of each method. The significant of each variable is labelled by asterisks under the coefficients. The most important factor is marked as three asterisks which p-value ranges from 0 to 0.001. The second important factor is marked as two asterisks which p-value ranges from 0.001 to 0.01. The third important factor is marked as an asterisk which p-value ranges from 0.01 to 0.1. The least important is also marked as a dot which p-value ranges from 0.1 to 1.

**Independent variables**	**Lag**	**Coefficients/Significant**		
		NB	Poisson	Quasi
Intercept		1.360221 ***	-1.540 ***	854.1501 ***
Cases	1	0.023180 ***	0.01249 ***	0.497920 ***
	2	0.004043		0.257250 ***
	3	-0.001372		0.086150 *
Average Pressure	0			
	1			
	2			-0.83637 ***
	3			
Minimum Temperature	0			
	1			
	2			
	3			
Maximum Temperature	0			
	1		0.08315 ***	
	2		0.08830 **	
	3			
Relative Humidity	0			-0.11776 *
	1			
	2	0.019350 **		
	3	-0.007674		
Precipitation	0			-0.04876
	1			
	2			
	3			-0.02610
Vaporization	0			
	1		-0.1716 ***	
	2		-0.1193 ***	
	3			
Wind Direction	0			
	1			
	2			
	3			
Wind Power	0			
	1			
	2			
	3			0.06794

Sa Kaeo

Sa Kaeo is located in the eastern region of Thailand at 13°49′14″N 102°03′32″E. Sa Kaeo covers an area of 7,195 km^2. The total populations are 552,187 people. The density of population is 83.2 people per km^2. Weather in Sa Kaeo has tropical monsoon climate system. Rainfall is light and infrequent. In winter occurs from December to January. Temperatures are in the range from the lowest at 5.0°C in December to highest at 40.4°C in April. The highest rainfall presents in September of 266.8 mm. Relative humidity are high in rainy season and low in winter around 68-87 percent. January is the highest month of sunshine hours

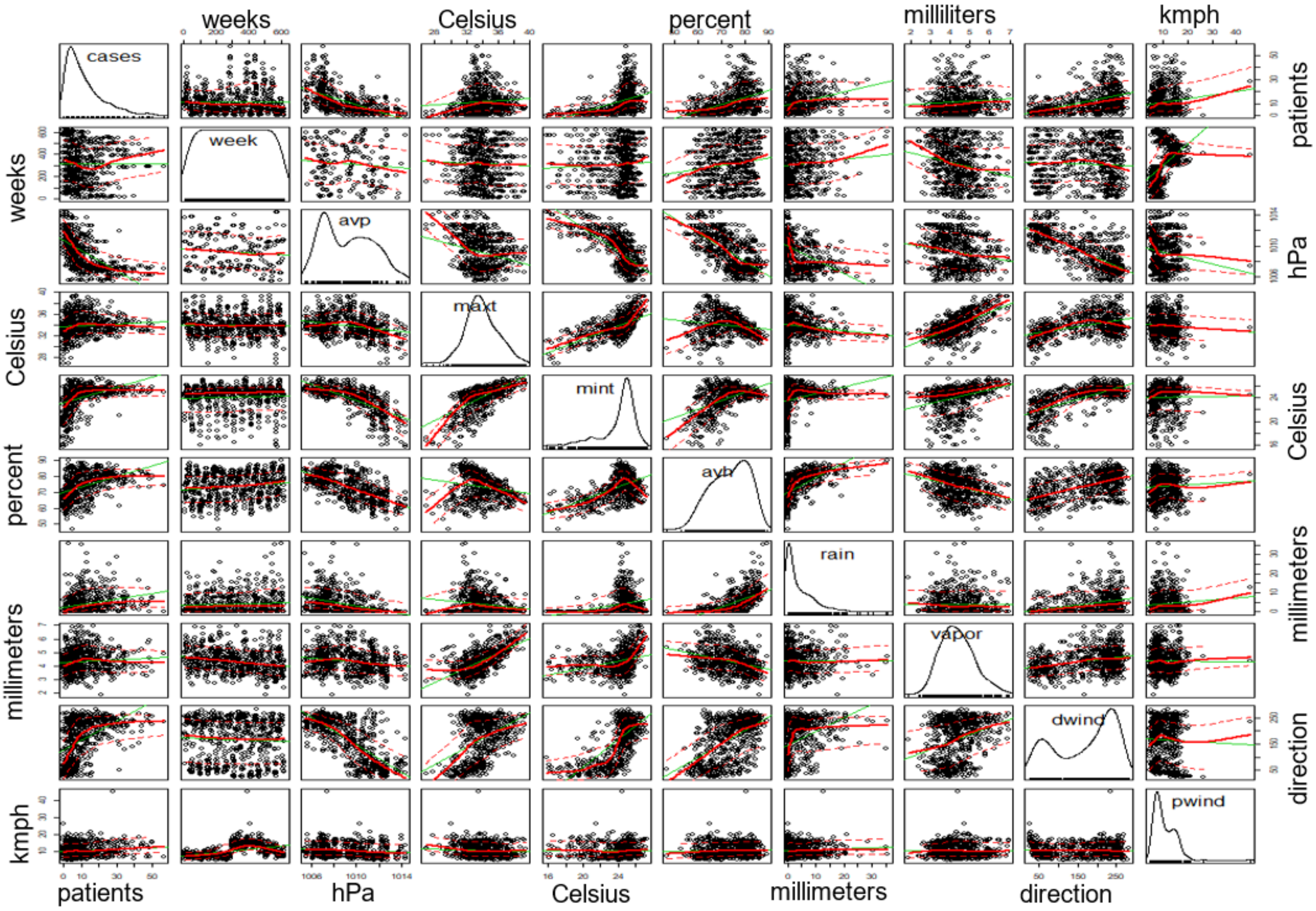

Figure 289: Scatter plot between dengue cases (cases) and selected independent variables, which are the weekly period starting from January 2001 – December 2013 (week), average pressure (avp), maximum temperature (maxt), minimum temperature (mint), average humidity (avh), precipitation (rain), vaporization of water (vapor), wind direction (dwind), and wind power (pwind). The plot visualizes pairwise hundred relationships of training set in Sa Kaeo.

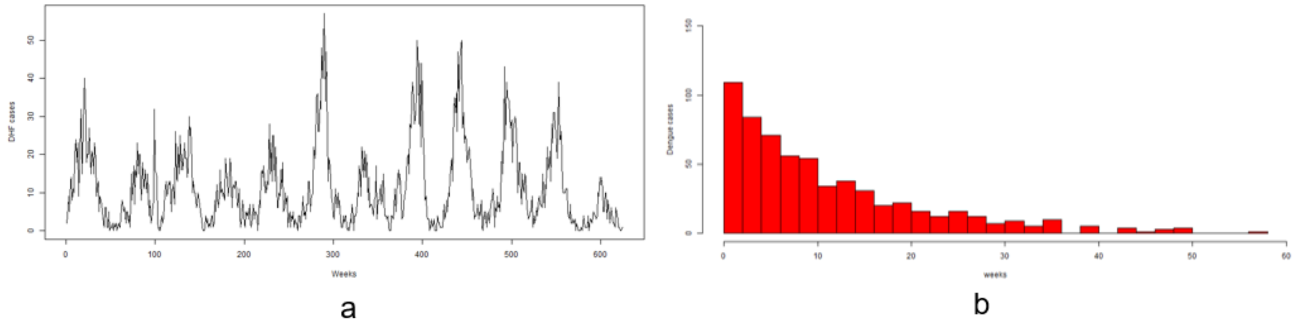

Figure 290: (a) Line plot between dengue incidences and weeks, the plot shows trends of dengue incidences in each year as stationary time series. (b) Histogram of dengue incidences in Sa Kaeo starting from January 2001 to December 2013 (624 weeks).

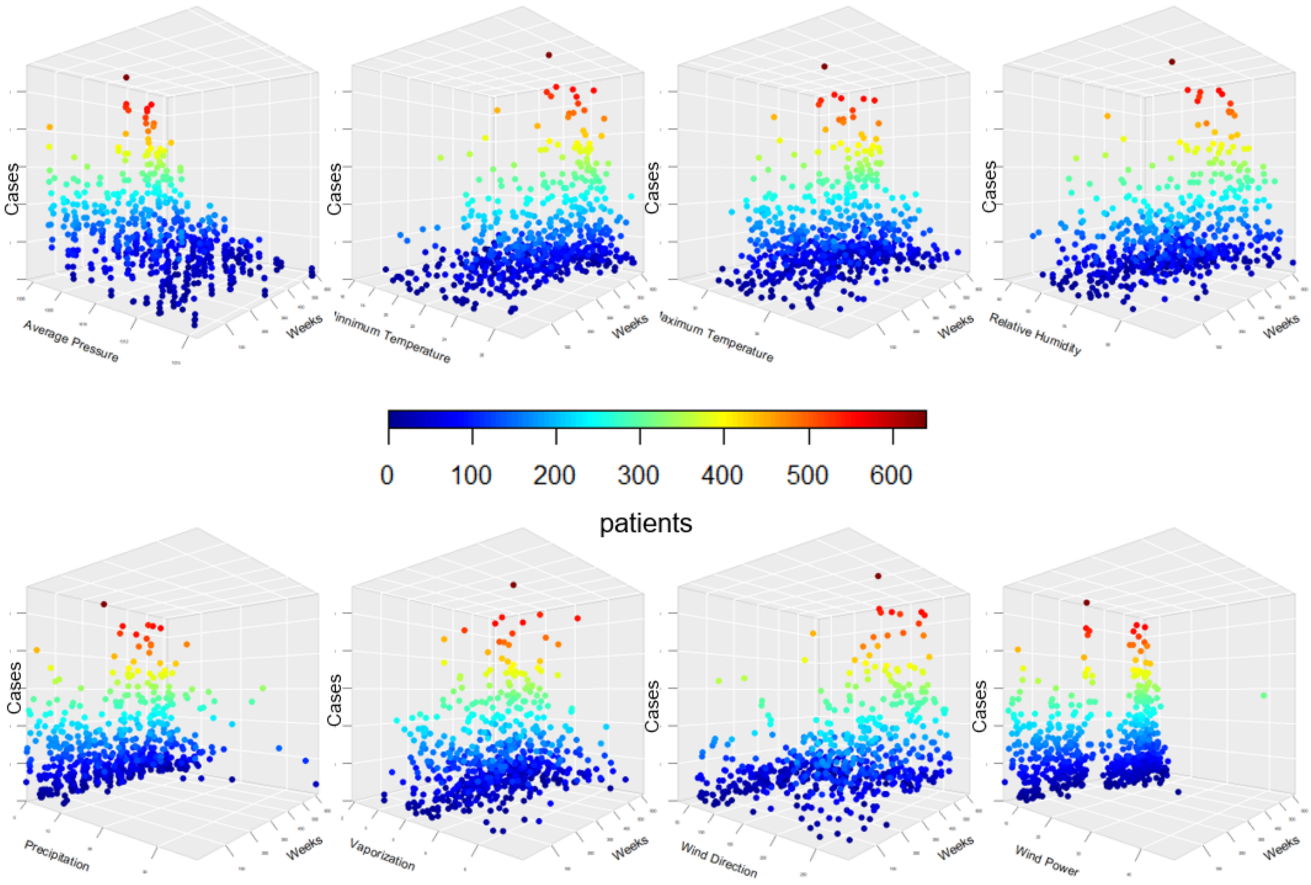

Figure 291: Three-dimensional scatter plot between dengue incidences and weather effects starting from January 2001 to December 2013 of Sa Kaeo.

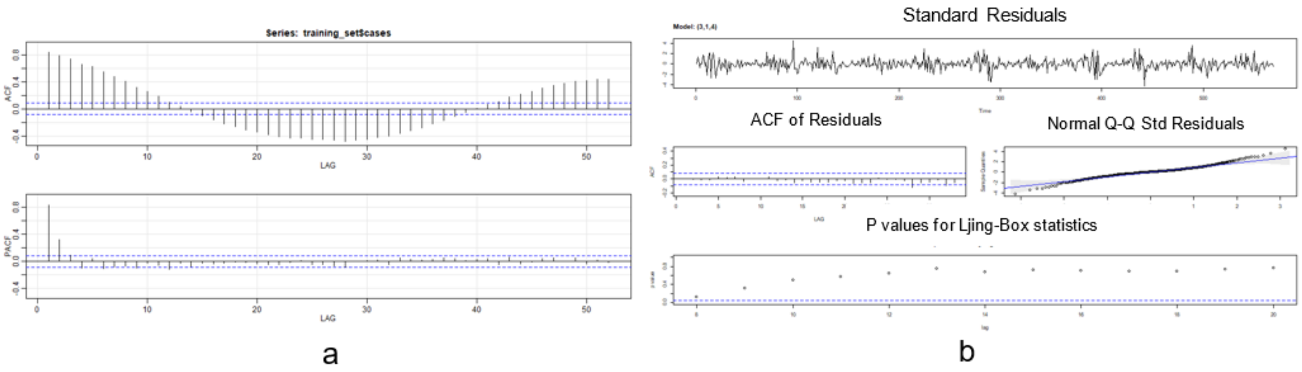

Figure 292: (a) Two plots between lag-time of dengue incidences and ACF and PACF relationship calculated from ARIMA model (b) Summary plots of time series analysis, multiple plots include the plot of predicted model over the time, the plot of ACF residual over lag-time of dengue incidences, residual Q-Q plot of standard residual, and p-value for Ljung-Box statistics of PACF relationship in Sa Kaeo over the training data starting from January 2001 to December 2013.

For Sa Keao, the best model is based on quasi-likelihood method. The correlation coefficient on the test set in 2014 is 0.594 (95%CI: 0.1682, 0.3578). The significant of the variables associated with p-value statistical calculation are shown in Table SKA2. The model consists of 8 variables. The most significant variables are 1-week-lag cases, 2-week-lag cases, 3-week-lag average pressure, 3-week-lag cases and current week relative humidity. Other variables which have less significant are, current week and 3-week-lag precipitation, 3-week-lag wind direction. Time series methods by ARIMA and SARIMA yield the correlation coefficient of -1.063103 and -7.763103 respectively.

Table 97: Comparison table of all methods by the highest correlation coefficient (R^2) and the lowest prediction error (RMSE) in Sa Kaeo.

Methods	R-squared (R^2)	Root mean square error (RMSE)
Poisson Regression	0.1004336	3.503886
Negative Binomial Regression	0.317485	3.052035
Quasi-likelihood Regression	0.2633236	3.170821
ARIMA (3,1,4)	-1.063103	5.306325
SARIMA $(2,0,1)(0,2,0)_{52}$	-7.763103	10.9361

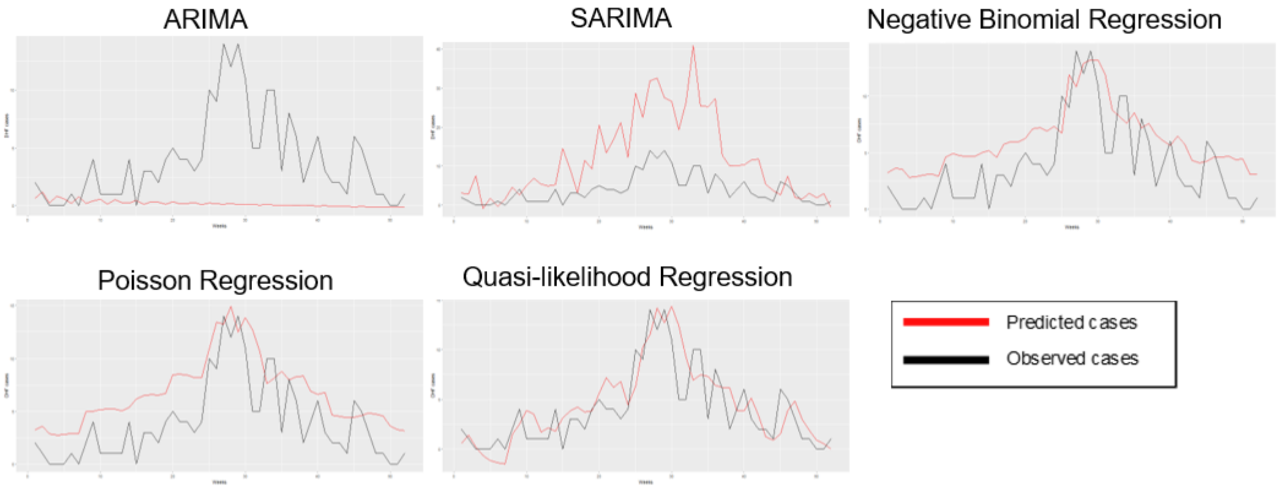

Figure 293: Plots between dengue cases and weeks, the black line represents the observed dengue cases, and the red line represents the predicted dengue cases of the best fit model of each technique over the test set data starting from January 2014 to December 2014.

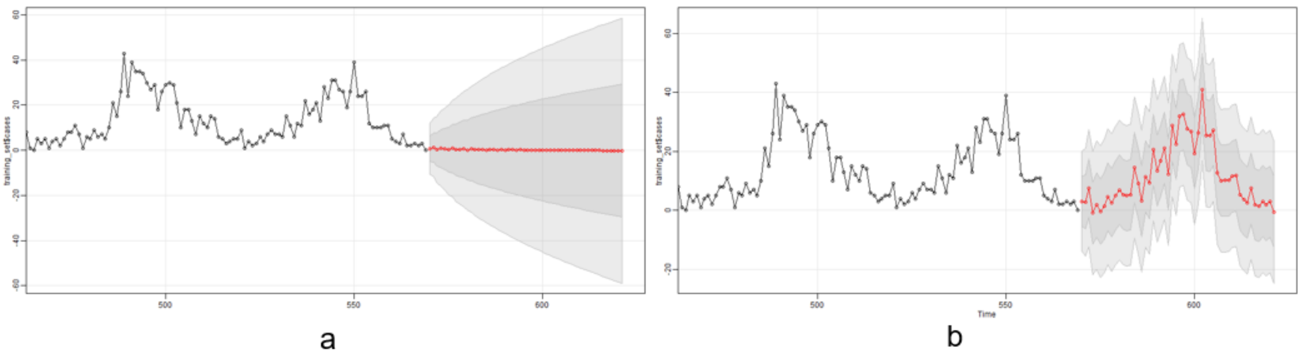

Figure 294: (a) Plot between dengue incidences over weekly time by the best model of ARIMA and (b) SARIMA time series analysis, the black line represents training set data starting from January 2012 to December 2013, and the red line represents the forecasted dengue incidences from January 2014 to December 2014.

Table 98: Coefficients and significant values of best fit GLM models, Negative Binomial, Poisson and Quasi-likelihood regression model of Sa Kaeo. The table summarizes coefficients of each independent variables which are composed in best fit model of each method. The significant of each variable is labelled by asterisks under the coefficients. The most important factor is marked as three asterisks which p-value ranges from 0 to 0.001. The second important factor is marked as two asterisks which p-value ranges from 0.001 to 0.01. The third important factor is marked as an asterisk which p-value ranges from 0.01 to 0.1. The least important is also marked as a dot which p-value ranges from 0.1 to 1.

**Independent variables**	**Lag**	**Coefficients/Significant**		
		NB	Poisson	Quasi
Intercept		123.761444 ***	163.799229 ***	793.783507 ***
Cases	1	0.03482700 ***	0.023726 ***	0.733322 ***
	2	0.01423000 ***	0.013833 ***	
	3	0.005128	0.005065 **	
Average Pressure	0		-0.004077	
	1			
	2		-0.159408 ***	-0.779903 ***
	3	-0.1201910 ***	-0.012483 ***	
Minimum Temperature	0			
	1			
	2			
	3			
Maximum Temperature	0			
	1			
	2			
	3			
Relative Humidity	0			
	1			-0.056003
	2			
	3	-0.0121000 **		
Precipitation	0			
	1			
	2	0.00161900		
	3		0.006674 *	
Vaporization	0			
	1			
	2			
	3			
Wind Direction	0			0.001420
	1			0.011740 *
	2			
	3			-0.007507
Wind Power	0			-0.035182
	1			
	2			
	3			

Sakon Nakhon

Sakon Nakhon is a province located the northeastern continent of Thailand at coordinate of 17°09′23″N 104°08′44″E. Sakon Nakhon covers an area of 9,606 km^2. Total population are 1,138,609 people. The density of population is approximately 119.0 people per km^2. Weather in Sakon Nakhon has tropical savanna climate under the South Asian monsoon system. High temperature is in April approximately 41.8°C and starts to low the temperature from December to March (-1.4-7.5°C). The monsoon season begins from May through October. The highest rainfall presents in August around 357.9 mm. Humidity is in range from 63-84 percent throughout the year. The highest sunshine hours are in January.

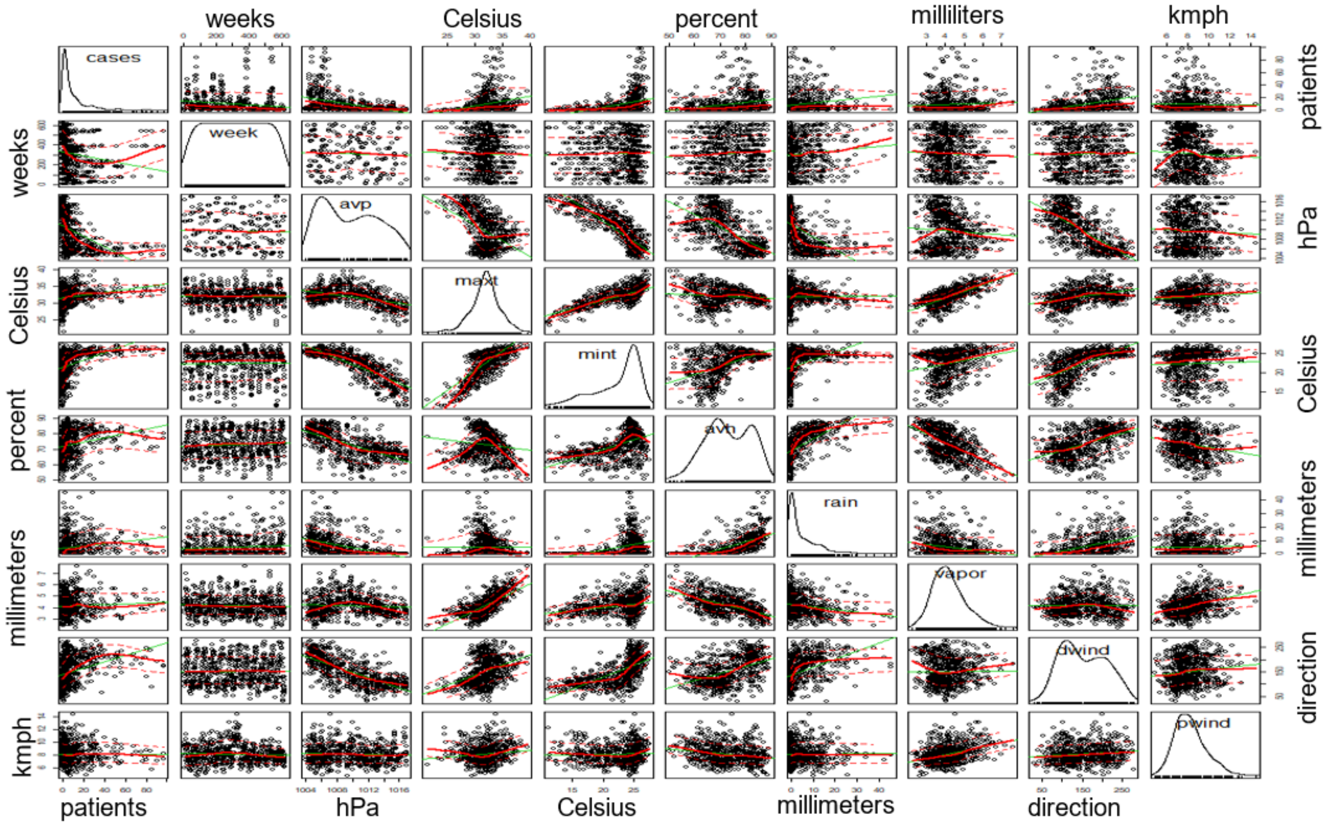

Figure 295: Scatter plot between dengue cases (cases) and selected independent variables, which are the weekly period starting from January 2001 – December 2013 (week), average pressure (avp), maximum temperature (maxt), minimum temperature (mint), average humidity (avh), precipitation (rain), vaporization of water (vapor), wind direction (dwind), and wind power (pwind). The plot visualizes pairwise hundred relationships of training set in Sakon Nakhon.

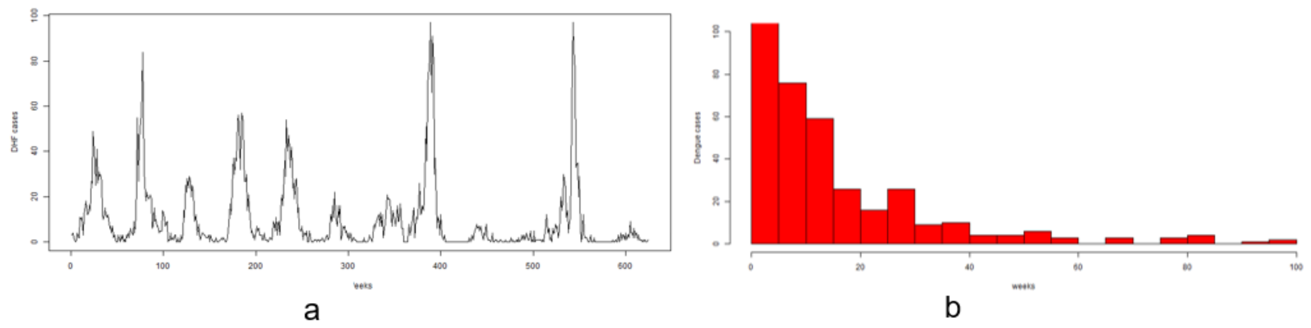

Figure 296: (a) Line plot between dengue incidences and weeks, the plot shows trends of dengue incidences in each year as stationary time series. (b) Histogram of dengue incidences in Sakon Nakhon starting from January 2001 to December 2013 (624 weeks).

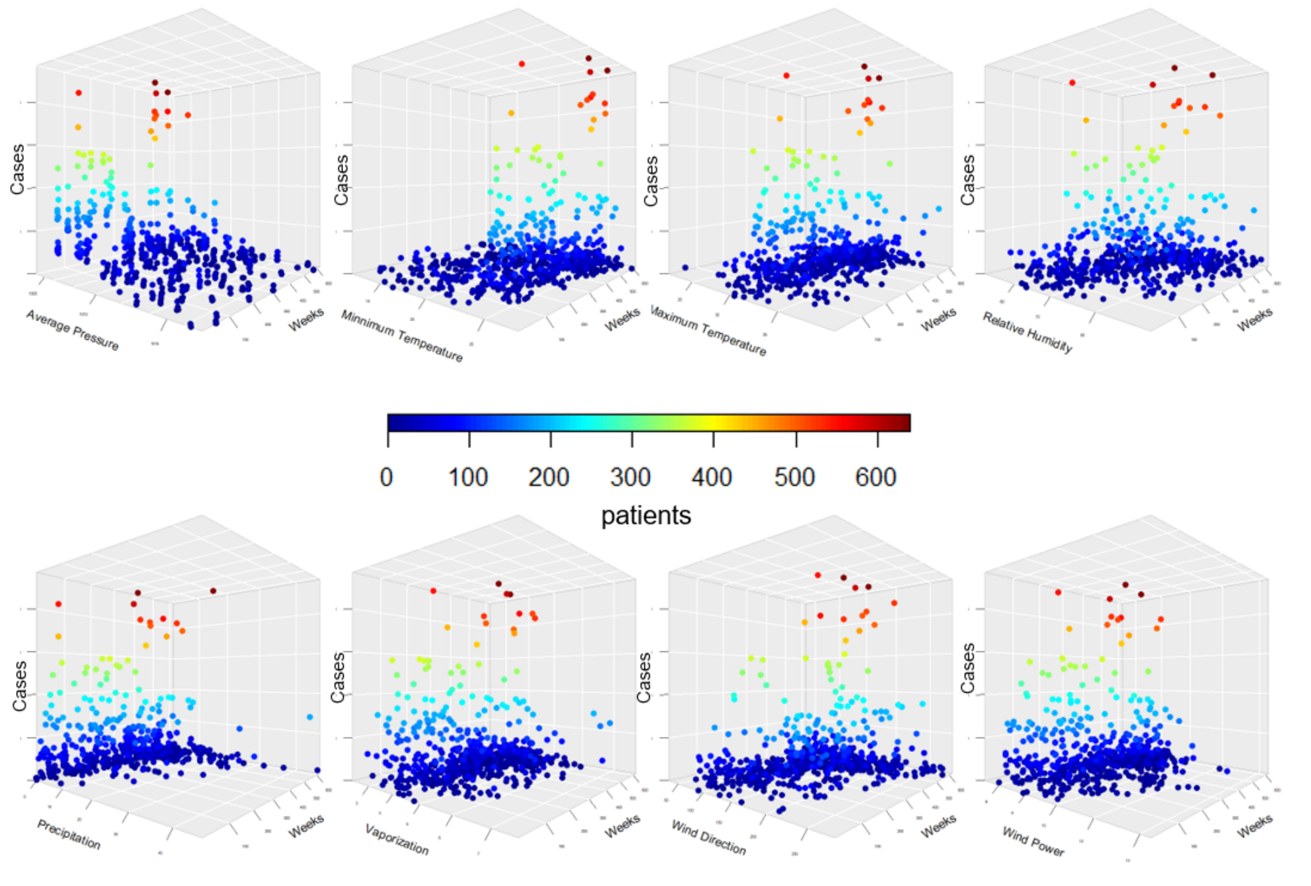

Figure 297: Three-dimensional scatter plot between dengue incidences and weather effects starting from January 2001 to December 2013 of Sakon Nakhon.

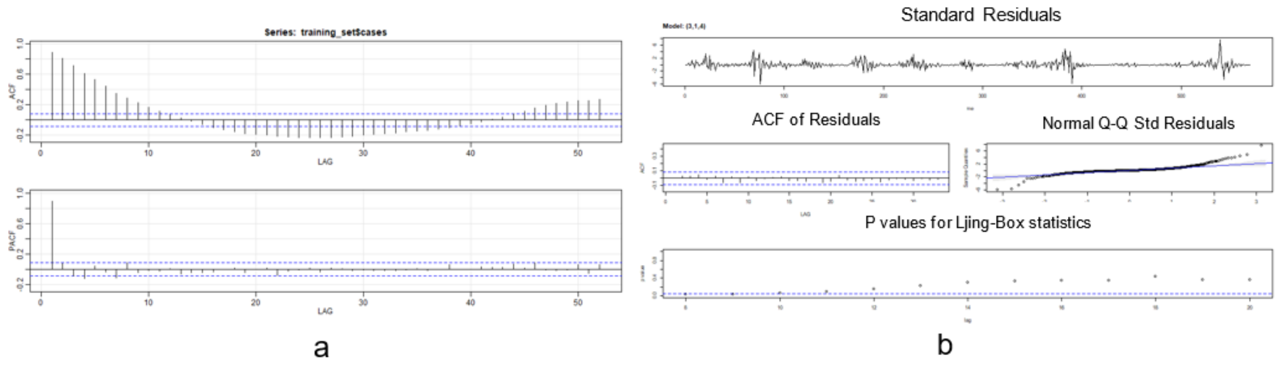

Figure 298: (a) Two plots between lag-time of dengue incidences and ACF and PACF relationship calculated from ARIMA model (b) Summary plots of time series analysis, multiple plots include the plot of predicted model over the time, the plot of ACF residual over lag-time of dengue incidences, residual Q-Q plot of standard residual, and p-value for Ljung-Box statistics of PACF relationship in Sakon Nakhon over the training data starting from January 2001 to December 2013.

For Sakon Nakhon, the best model is based on quasi-likelihood method. The correlation coefficient on the test set in 2014 is 0.026 (95% CI: 0.06597, 0.1180). The significant of the variables associated with p-value statistical calculation are shown in Table 99. The best model uses 8 variables. The most significant variable is 1-week-lag cases. Other variables which have less significant are, 2-week-lag cases, 1-week-lag relative humidity, current week and 1-week-lag precipitation, 1-week-lag and 2-week-lag wind power. Time series methods by ARIMA and SARIMA yield the correlation coefficient of -5.12898 and -221.6847 respectively.

Table 99: Comparison table of all methods by the highest correlation coefficient (R^2) and the lowest prediction error (RMSE) in Sakon Nakhon.

Methods	R-squared (R^2)	Root mean square error (RMSE)
Poisson Regression	-3.106152	3.779748
Negative Binomial Regression	-0.4803247	2.269467
Quasi-likelihood Regression	0.02574772	1.841115
ARIMA (3,1,4)	-5.12898	4.617846
SARIMA $(2,0,1)(0,2,0)_{52}$	-221.6847	27.83496

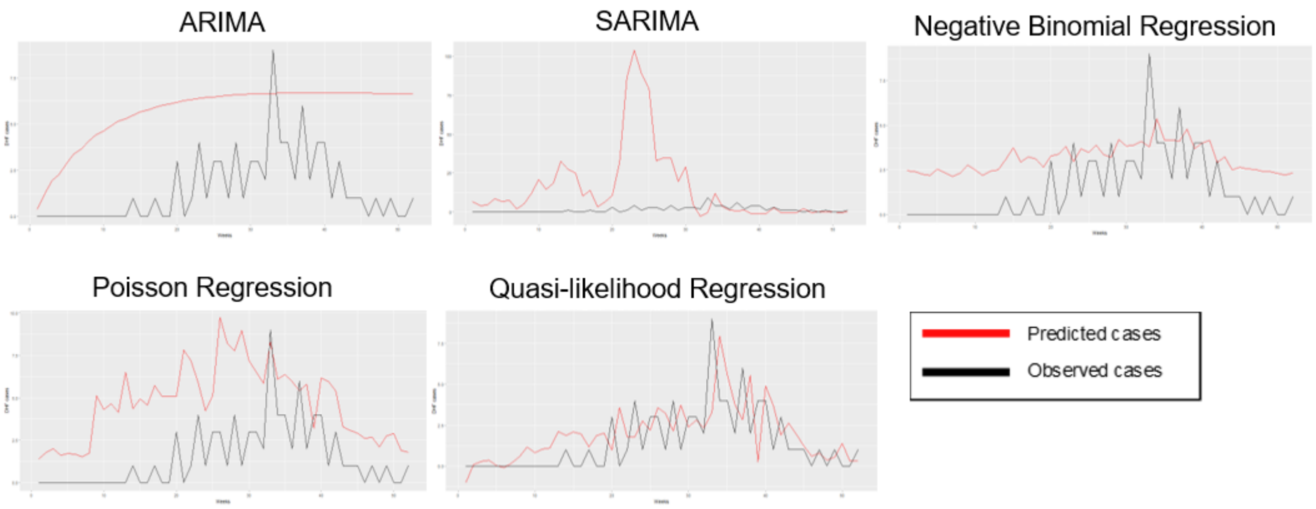

Figure 299: Plots between dengue cases and weeks, the black line represents the observed dengue cases, and the red line represents the predicted dengue cases of the best fit model of each technique over the test set data starting from January 2014 to December 2014.

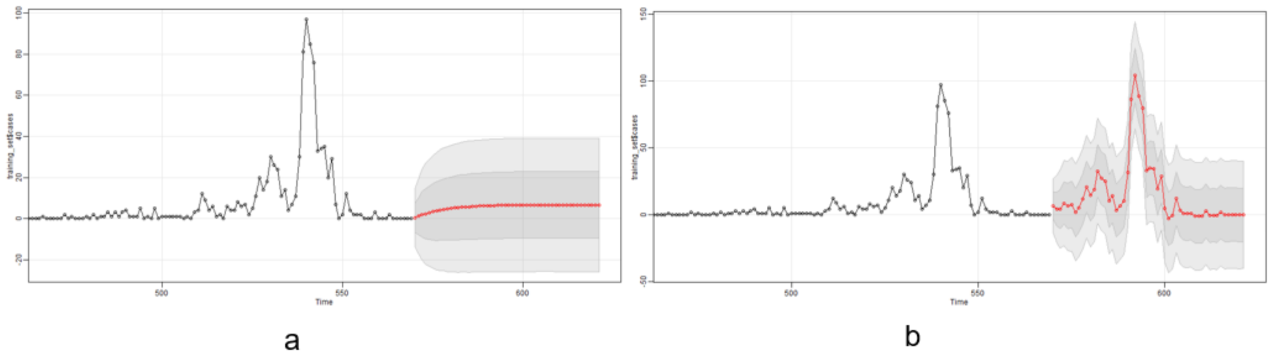

Figure 300: (a) Plot between dengue incidences over weekly time by the best model of ARIMA and (b) SARIMA time series analysis, the black line represents training set data starting from January 2012 to December 2013, and the red line represents the forecasted dengue incidences from January 2014 to December 2014.

Table 100: Coefficients and significant values of best fit GLM models, Negative Binomial, Poisson and Quasi-likelihood regression model of Sakon Nakhon. The table summarizes coefficients of each independent variables which are composed in best fit model of each method. The significant of each variable is labelled by asterisks under the coefficients. The most important factor is marked as three asterisks which p-value ranges from 0 to 0.001. The second important factor is marked as two asterisks which p-value ranges from 0.001 to 0.01. The third important factor is marked as an asterisk which p-value ranges from 0.01 to 0.1. The least important is also marked as a dot which p-value ranges from 0.1 to 1.

**Independent variables**	**Lag**	**Coefficients/Significant**		
		NB	Poisson	Quasi
Intercept		-1.045248 *	181.2 ***	159.19124
Cases	1	0.055215 ***	0.02826 ***	0.8237400 ***
	2	0.015406 **	0.003727 ***	0.06737
	3			
Average Pressure	0			
	1			
	2			
	3		-0.1769 ***	-0.16064
Minimum Temperature	0			
	1			
	2			
	3			
Maximum Temperature	0			
	1			
	2			
	3			
Relative Humidity	0		-0.02175 ***	
	1			0.0221500
	2	0.012265 *		
	3			
Precipitation	0		-0.01416 ***	-0.03749
	1			-0.07741
	2			
	3			
Vaporization	0			
	1			
	2			
	3			
Wind Direction	0			
	1			
	2			
	3			
Wind Power	0	0.010470		
	1		0.03087 **	0.18061
	2	0.039754		
	3	0.094527 **	0.05093 ***	

Satun

Satun is a province located in the southern region of Thailand at coordinate of 6°36′53″N 100°04′05″E. Satun province covers an area of 2,479 km^2. Total population are 312,673 people. The density of population is 126.0 people per km^2. General weather in Satun are under tropical monsoon climate. The highest temperature is in April approximately 38.9°C. Temperatures are stable throughout the year. Satun has a short dry season in February and March. The highest rainfall presents in October around 339.0 mm. Humidity is in range from 69-85 percent throughout the year. The highest sunshine hours are in January.

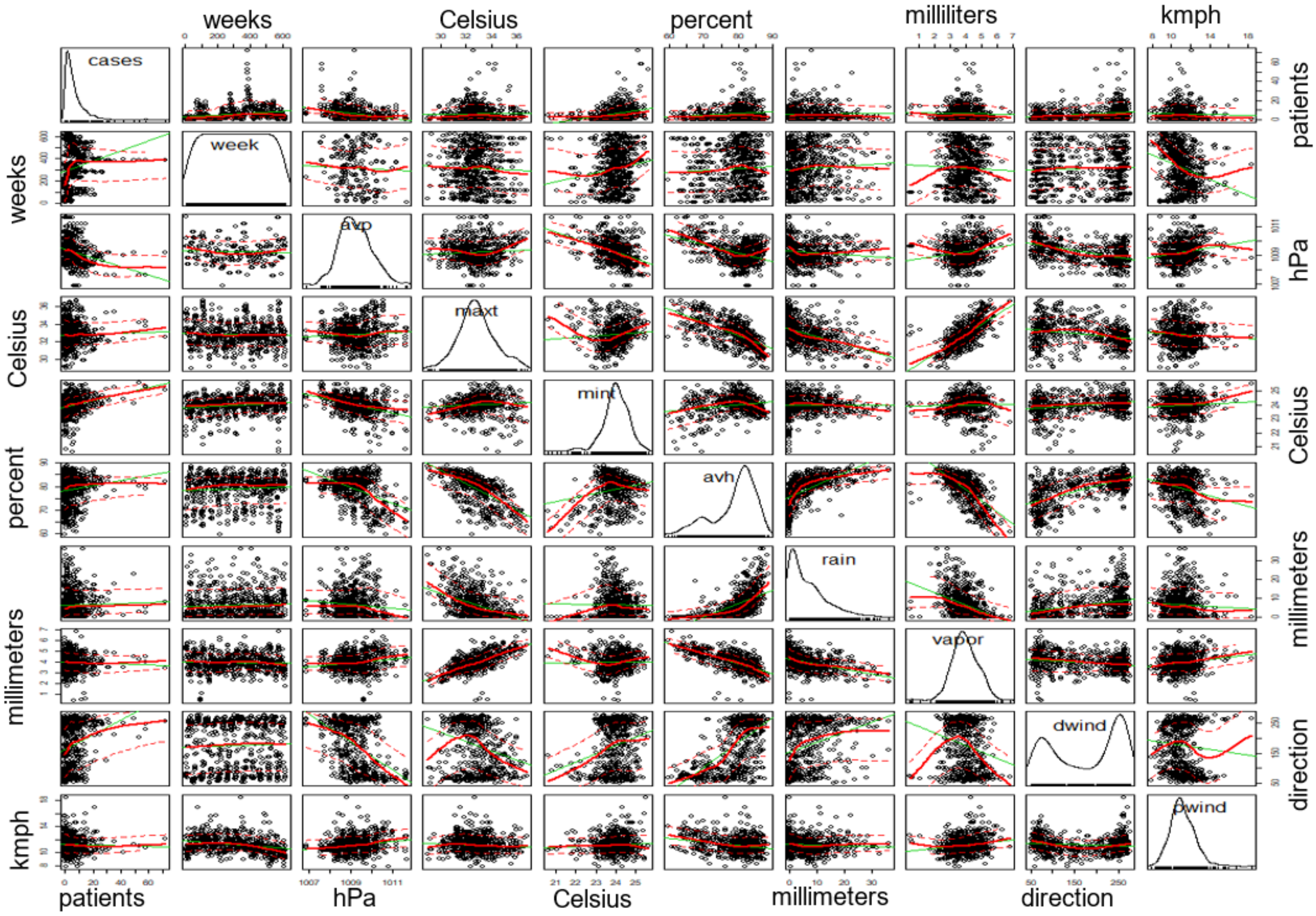

Figure 301: Scatter plot between dengue cases (cases) and selected independent variables, which are the weekly period starting from January 2001 – December 2013 (week), average pressure (avp), maximum temperature (maxt), minimum temperature (mint), average humidity (avh), precipitation (rain), vaporization of water (vapor), wind direction (dwind), and wind power (pwind). The plot visualizes pairwise hundred relationships of training set in Satun.

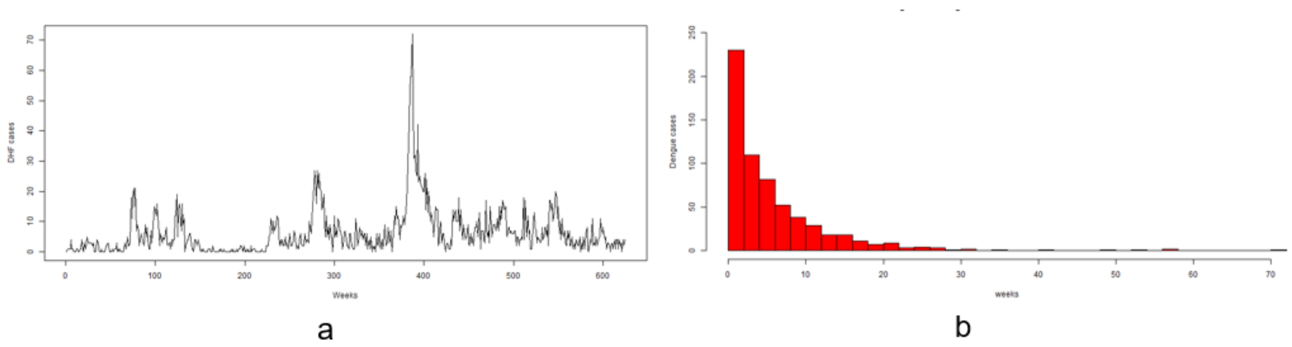

Figure 302: (a) Line plot between dengue incidences and weeks, the plot shows trends of dengue incidences in each year as stationary time series. (b) Histogram of dengue incidences in Satun starting from January 2001 to December 2013 (624 weeks).

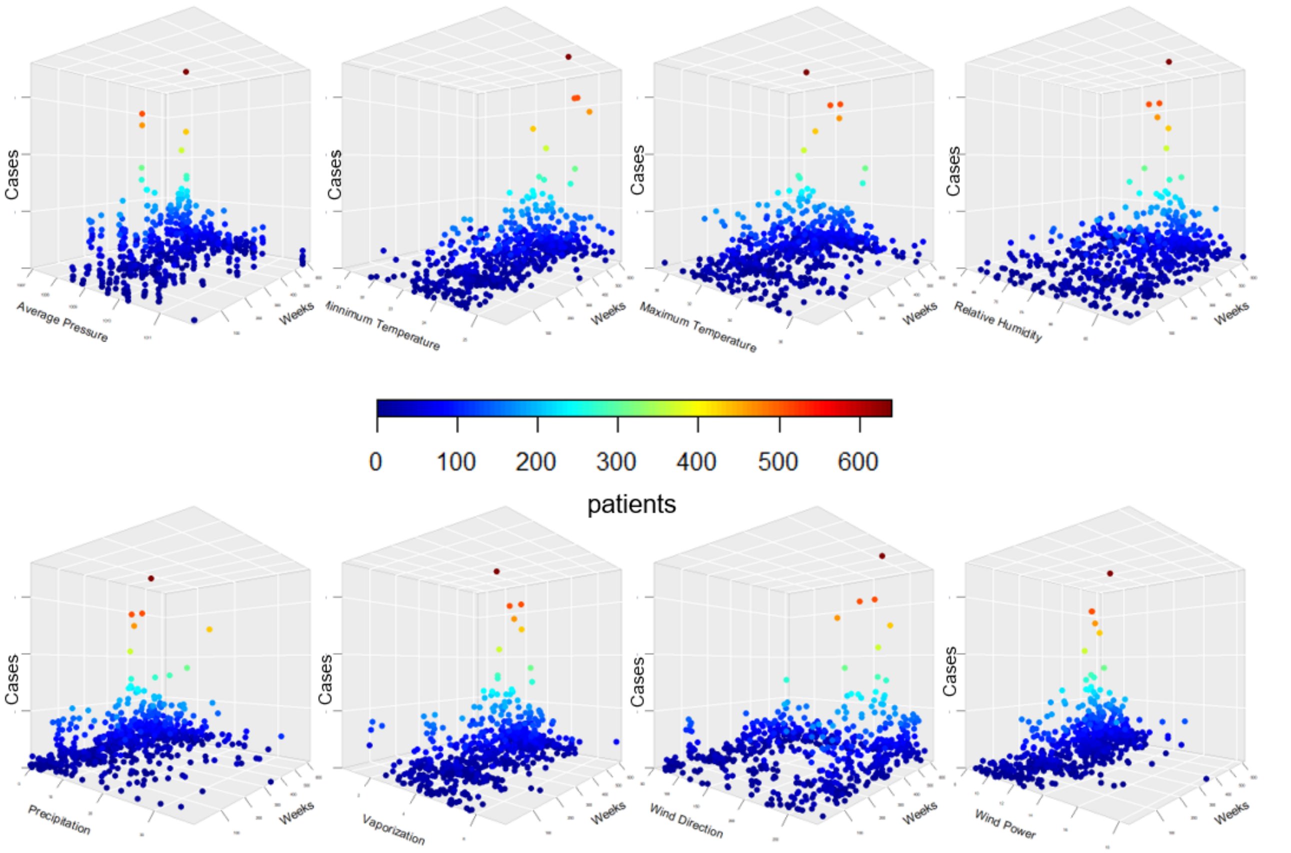

Figure 303: Three-dimensional scatter plot between dengue incidences and weather effects starting from January 2001 to December 2013 of Satun.

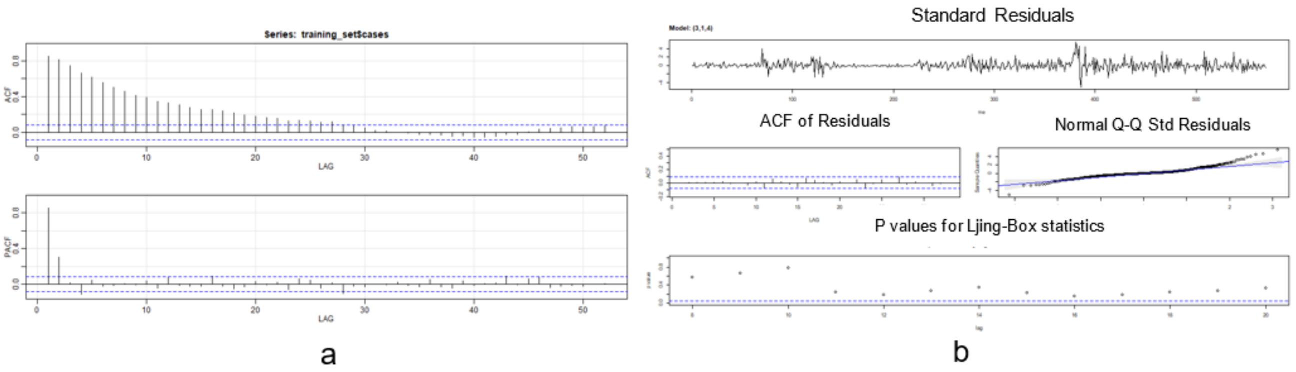

Figure 304: (a) Two plots between lag-time of dengue incidences and ACF and PACF relationship calculated from ARIMA model (b) Summary plots of time series analysis, multiple plots include the plot of predicted model over the time, the plot of ACF residual over lag-time of dengue incidences, residual Q-Q plot of standard residual, and p-value for Ljung-Box statistics of PACF relationship in Satun over the training data starting from January 2001 to December 2013.

The best model of Satun is based on Negative Binomial regression method. The correlation coefficient on the test set in 2014 is 0.270 (95%CI: 0.1607, 0.3813). The significant of the variables associated with p-value statistical calculation are shown in 101. The model uses 7 variables, which are 1-week-lag cases, 2-week-lag cases, 2-week-lag minimum temperature and current week maximum temperature significantly. Other variables which have less significant are, current week average pressure, 2-week-lag maximum temperature and 2-week-lag precipitation. Time series methods by ARIMA and SARIMA yield the correlation coefficient of -5.460416 and -6.87267 respectively.

Table 101: Comparison table of all methods by the highest correlation coefficient (R^2) and the lowest prediction error (RMSE) in Satun.

Methods	R-squared (R^2)	Root mean square error (RMSE)
Poisson Regression	-1.50361	3.930811
Negative Binomial Regression	0.2705424	2.121773
Quasi-likelihood Regression	-5.488136	6.327888
ARIMA (3,1,4)	-5.460416	6.314356
SARIMA $(2,0,1)(0,2,0)_{52}$	-6.87267	6.970434

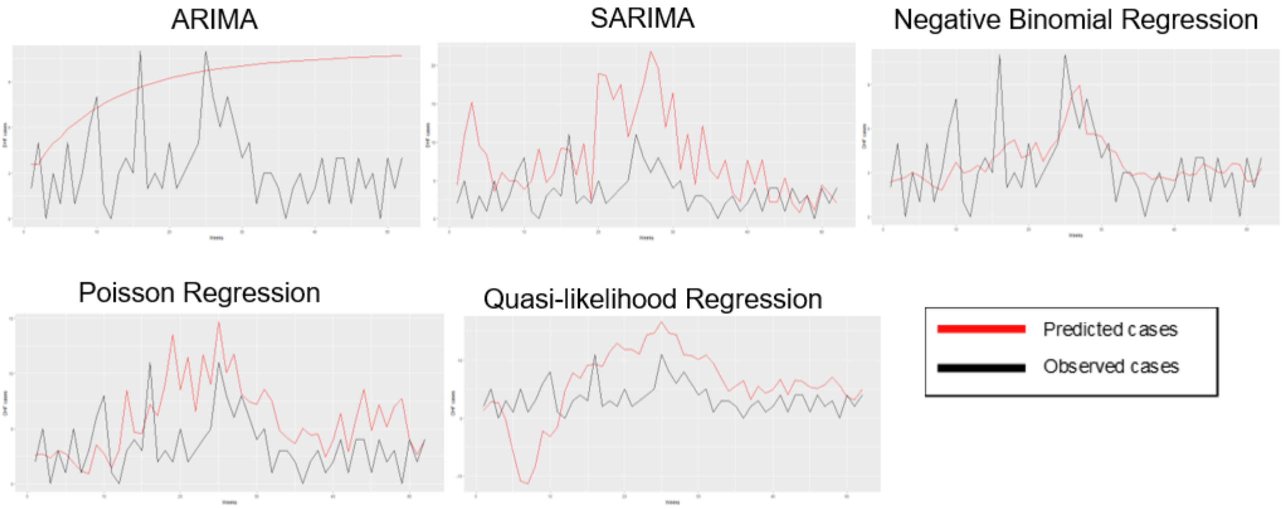

Figure 305: Plots between dengue cases and weeks, the black line represents the observed dengue cases, and the red line represents the predicted dengue cases of the best fit model of each technique over the test set data starting from January 2014 to December 2014.

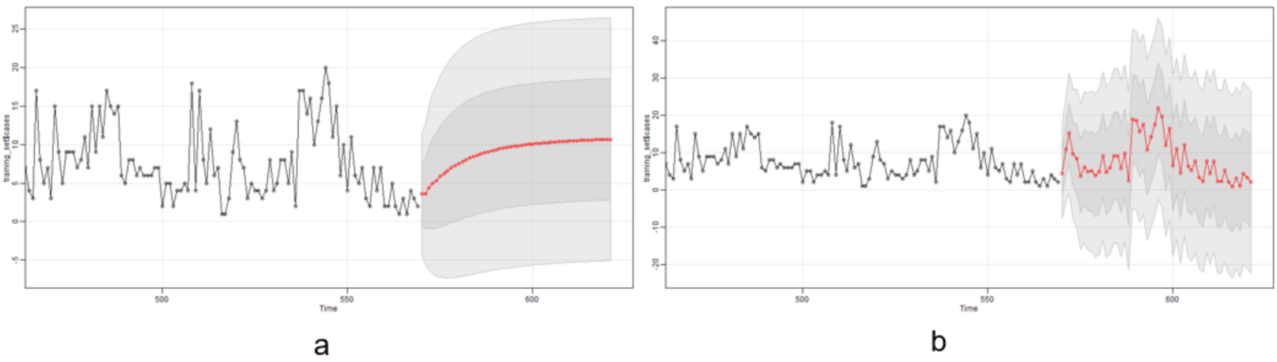

Figure 306: (a) Plot between dengue incidences over weekly time by the best model of ARIMA and (b) SARIMA time series analysis, the black line represents training set data starting from January 2012 to December 2013, and the red line represents the forecasted dengue incidences from January 2014 to December 2014.

Table 102: Coefficients and significant values of best fit GLM models, Negative Binomial, Poisson and Quasi-likelihood regression model of Satun. The table summarizes coefficients of each independent variables which are composed in best fit model of each method. The significant of each variable is labelled by asterisks under the coefficients. The most important factor is marked as three asterisks which p-value ranges from 0 to 0.001. The second important factor is marked as two asterisks which p-value ranges from 0.001 to 0.01. The third important factor is marked as an asterisk which p-value ranges from 0.01 to 0.1. The least important is also marked as a dot which p-value ranges from 0.1 to 1.

**Independent variables**	**Lag**	**Coefficients/Significant**		
		NB	Poisson	Quasi
Intercept		69.236557	-15.853472 ***	936.725780 .
Cases	1	0.0523360 ***		
	2	0.0383010 ***		
	3		0.043092 ***	
Average Pressure	0	-0.073469		
	1			
	2			
	3			-1.082075 *
Minimum Temperature	0		0.152490 ***	1.586293 **
	1			1.675683 **
	2	0.2151570 ***	0.419442 ***	2.544525 ***
	3			
Maximum Temperature	0	0.0556470 .		0.761465 *
	1			
	2	-0.031890		
	3			
Relative Humidity	0		0.022267 ***	
	1			
	2			
	3		0.022485 ***	
Precipitation	0		-0.010149 **	
	1			
	2	-0.005893	-0.010221 ***	
	3			
Vaporization	0			-0.561819
	1			
	2			
	3			-0.384566
Wind Direction	0			0.007208
	1			
	2			
	3			
Wind Power	0			
	1			
	2		-0.109880 ***	
	3			

Si Sa Ket

Si Sa Ket is a province located in the northeastern continent of Thailand at coordinate of 15°06′25″N 104°19′46″E. Si Sa Ket covers an area of 8,840 km^2. Total population are 1,465,213 people. The density of population is 166.0 people per km^2. General weather in Si Sa Ket are under tropical savanna climate under the South Asian monsoon system. Temperature is in the range from low in December to high in April. The rainy season begins with the arrival of the southwest monsoon around mid-May.

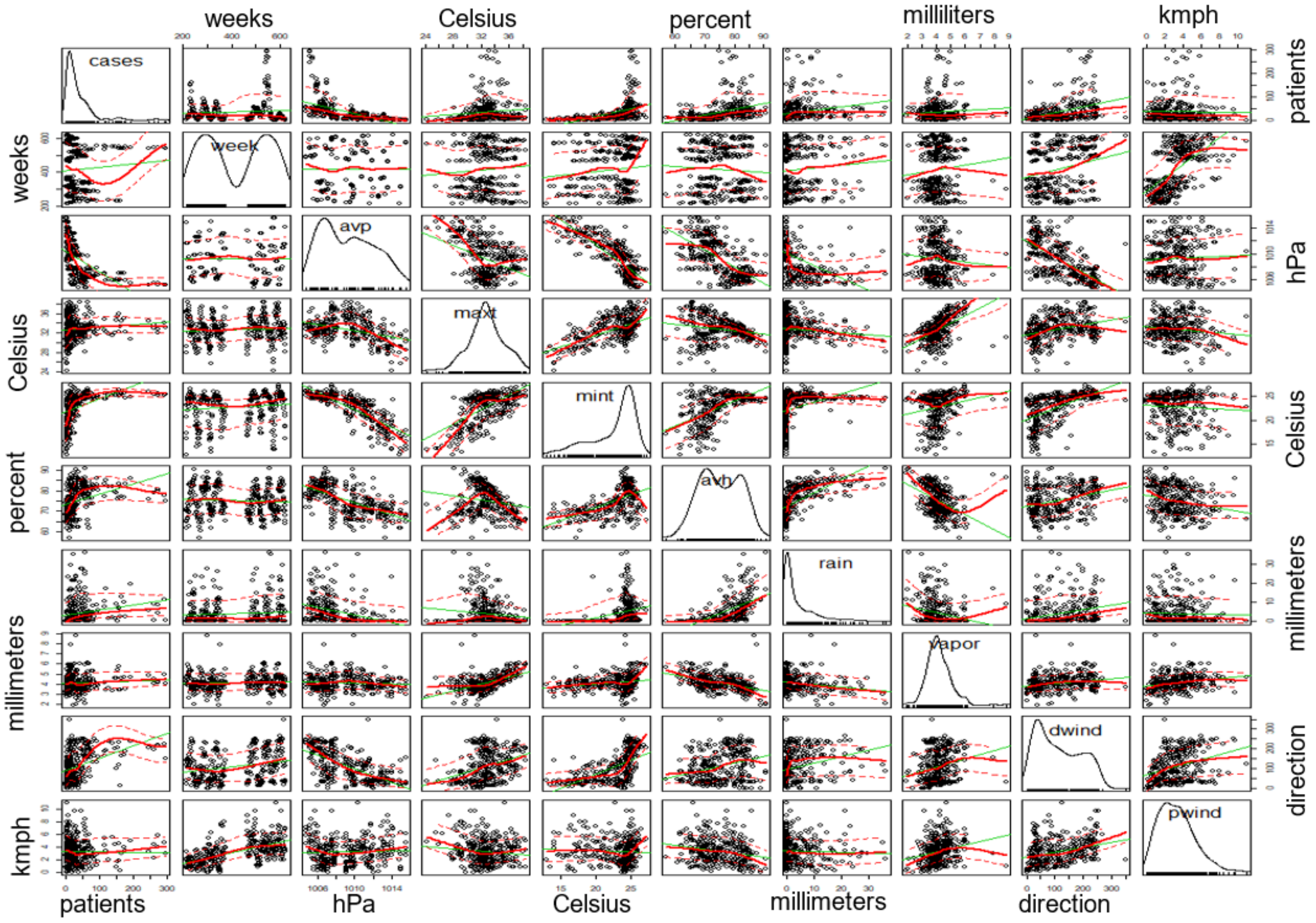

Figure 307: Scatter plot between dengue cases (cases) and selected independent variables, which are the weekly period starting from January 2001 – December 2013 (week), average pressure (avp), maximum temperature (maxt), minimum temperature (mint), average humidity (avh), precipitation (rain), vaporization of water (vapor), wind direction (dwind), and wind power (pwind). The plot visualizes pairwise hundred relationships of training set in Si Sa Ket.

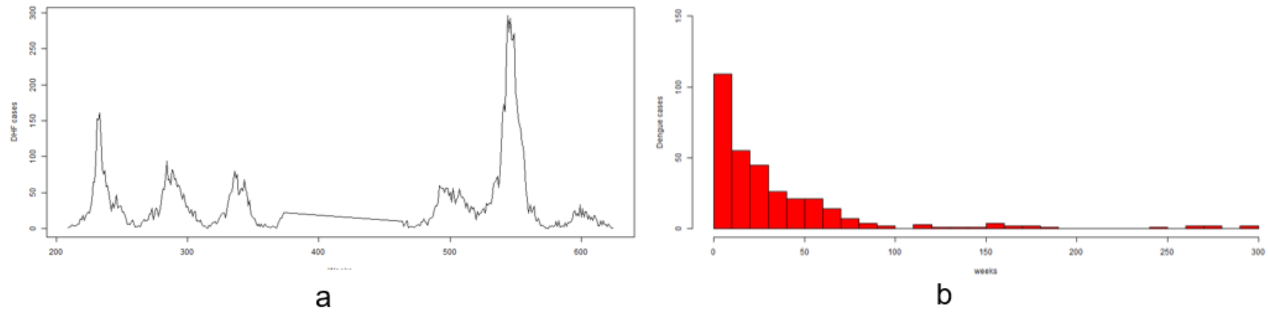

Figure 308: (a) Line plot between dengue incidences and weeks, the plot shows trends of dengue incidences in each year as stationary time series. (b) Histogram of dengue incidences in Si Sa Ket starting from January 2001 to December 2013 (624 weeks).

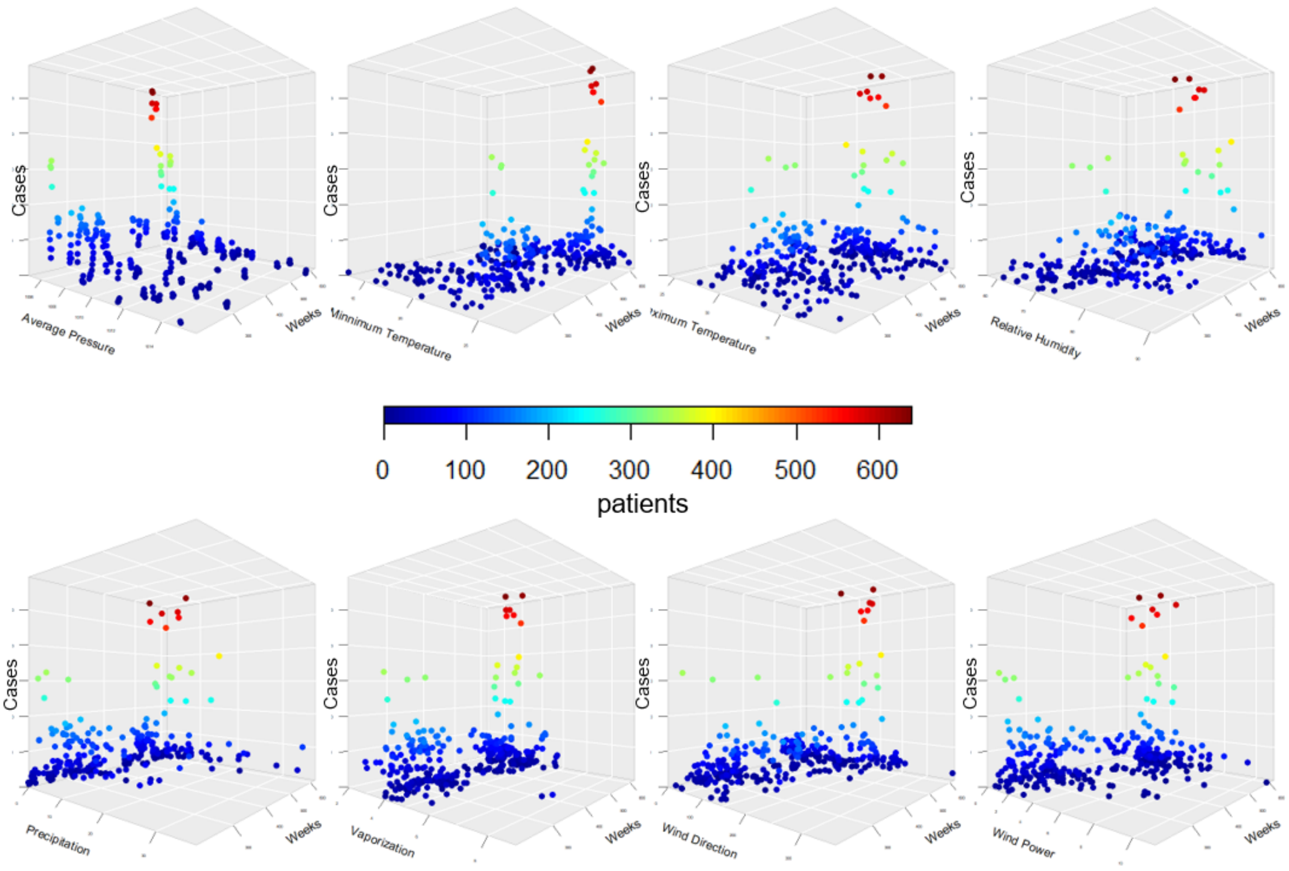

Figure 309: Three-dimensional scatter plot between dengue incidences and weather effects starting from January 2001 to December 2013 of Si Sa Ket.

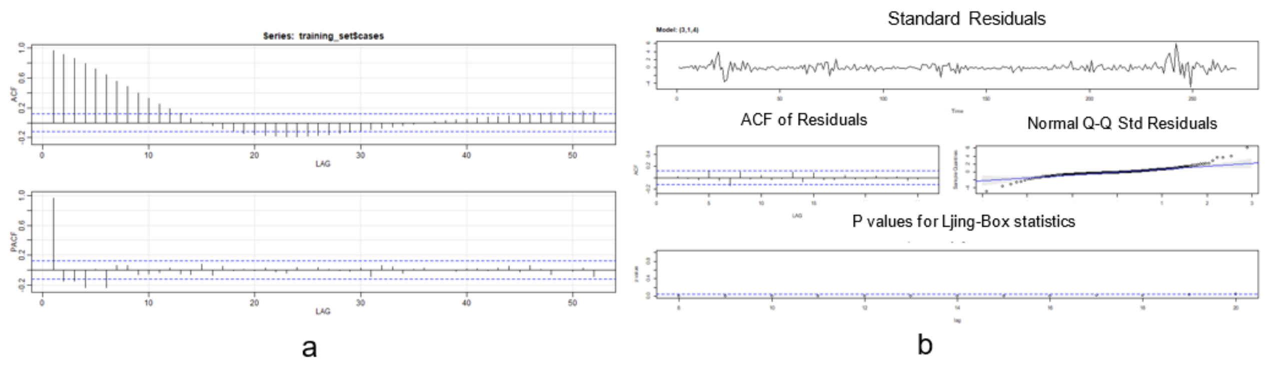

Figure 310: (a) Two plots between lag-time of dengue incidences and ACF and PACF relationship calculated from ARIMA model (b) Summary plots of time series analysis, multiple plots include the plot of predicted model over the time, the plot of ACF residual over lag-time of dengue incidences, residual Q-Q plot of standard residual, and p-value for Ljung-Box statistics of PACF relationship in Si Sa Ket over the training data starting from January 2001 to December 2013.

For Si Sa Ket, the best model is based on quasi-likelihood method. The correlation coefficient on the test set in 2014 is 0.565 (95%CI: 0.4841, 0.6459). The best model uses 7 variables. The most significant variables are 2-week-lag cases, 2-week-lag relative humidity and 3-week-lag average pressure. Other variables which has less significant are, 3-week-lag precipitation, current week, 1-week-lag and 2-week-lag wind power. The significant of the variables associated with p-value statistical calculation are shown in 103. Time series methods by ARIMA and SARIMA yield the correlation coefficient of -38.24149 and -368.3108 respectively.

Table 103: Comparison table of all methods by the highest correlation coefficient (R^2) and the lowest prediction error (RMSE) in Si Sa Ket.

**Methods**	**R-squared (R^2)**	**Root mean square error (RMSE)**
Poisson Regression	0.01359814	8.002606
Negative Binomial Regression	-1.204278	11.96293
Quasi-likelihood Regression	0.5651806	5.313234
ARIMA (3,1,4)	-38.24149	50.47511
SARIMA $(2,0,1)(0,2,0)_{52}$	-368.3108	154.8462

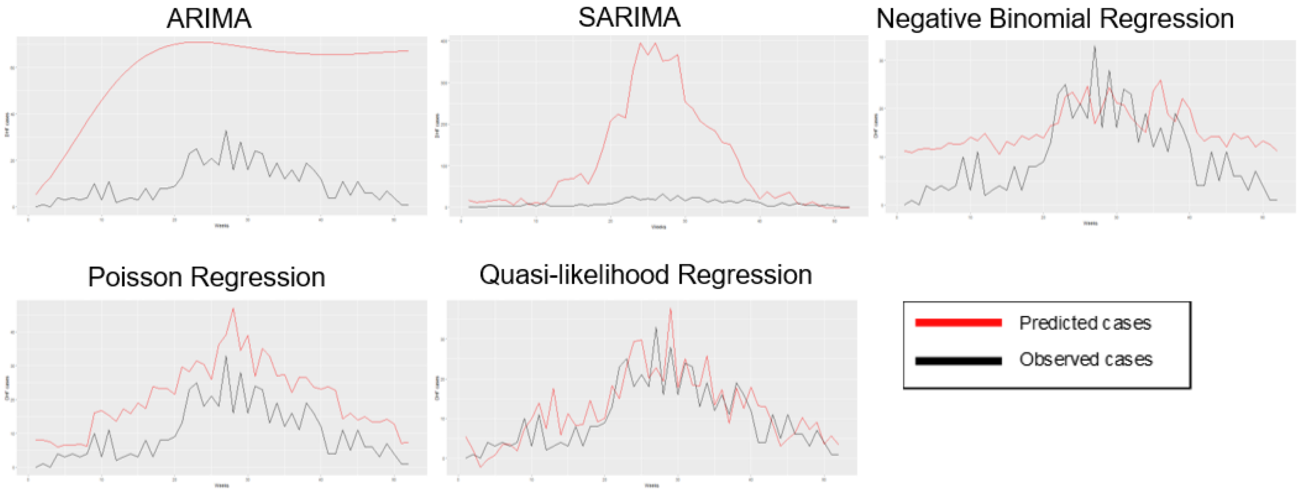

Figure 311: Plots between dengue cases and weeks, the black line represents the observed dengue cases, and the red line represents the predicted dengue cases of the best fit model of each technique over the test set data starting from January 2014 to December 2014.

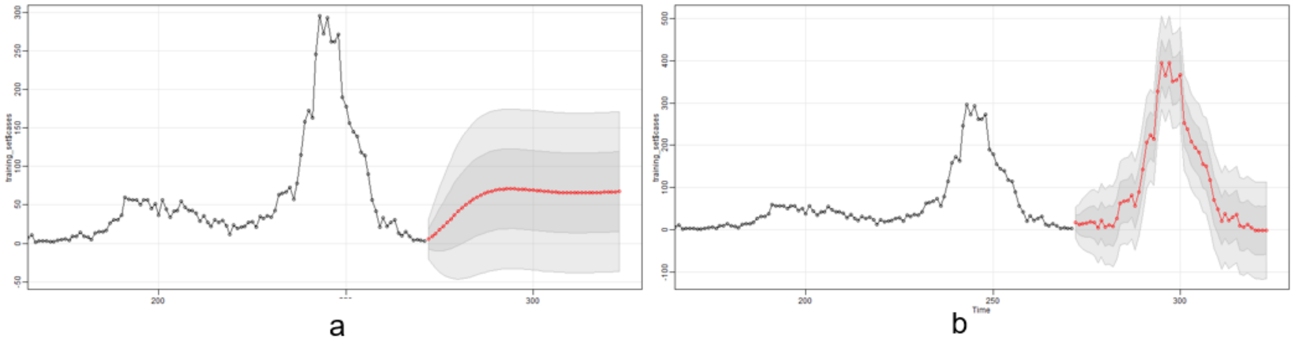

Figure 312: (a) Plot between dengue incidences over weekly time by the best model of ARIMA and (b) SARIMA time series analysis, the black line represents training set data starting from January 2012 to December 2013, and the red line represents the forecasted dengue incidences from January 2014 to December 2014.

Table 104: Coefficients and significant values of best fit GLM models, Negative Binomial, Poisson and Quasi-likelihood regression model of Si Sa Ket. The table summarizes coefficients of each independent variables which are composed in best fit model of each method. The significant of each variable is labelled by asterisks under the coefficients. The most important factor is marked as three asterisks which p-value ranges from 0 to 0.001. The second important factor is marked as two asterisks which p-value ranges from 0.001 to 0.01. The third important factor is marked as an asterisk which p-value ranges from 0.01 to 0.1. The least important is also marked as a dot which p-value ranges from 0.1 to 1.

**Independent variables**	**Lag**	**Coefficients/Significant**		
		NB	Poisson	Quasi
Intercept		2.3870668 ***	204.9 ***	1341.45526 .
Cases	1	0.0174542 ***	0.008151 ***	
	2			0.91641000 ***
	3			
Average Pressure	0			
	1			
	2			
	3		-0.1988 ***	-1.2668500 .
Minimum Temperature	0			
	1			
	2			
	3			
Maximum Temperature	0			
	1			
	2			
	3			
Relative Humidity	0			
	1			
	2		-0.01423 ***	-0.7788700 **
	3			
Precipitation	0	0.0211319 ***	-0.008025 ***	
	1			
	2			
	3		-0.0002507	0.20867
Vaporization	0			
	1			
	2			
	3			
Wind Direction	0			
	1			
	2	0.0582161		
	3			
Wind Power	0		-0.02236 **	0.13603
	1	-0.0018732	-0.01189	-0.11670
	2	-0.0317373	-0.01525	-0.57462
	3	-0.0082802		

Songkhla

Songkhla is located in the southern region of Thailand at coordinate of 7°12′22″N 100°35′48″E. Songkhla covers an area of 7,394 km^2. Total population are 1,401,303 people. The density of population is 190.0 people per km^2. General weather in Songkhla are under tropical monsoon climate. The highest temperature is in May approximately 38.6°C. Temperatures are very warm to hot throughout the year. Songkhla has a short dry season in February and March. The highest rainfall presents in November around 545.9 mm. Humidity is in range from 77-84 percent throughout the year. The highest sunshine hours are in March.

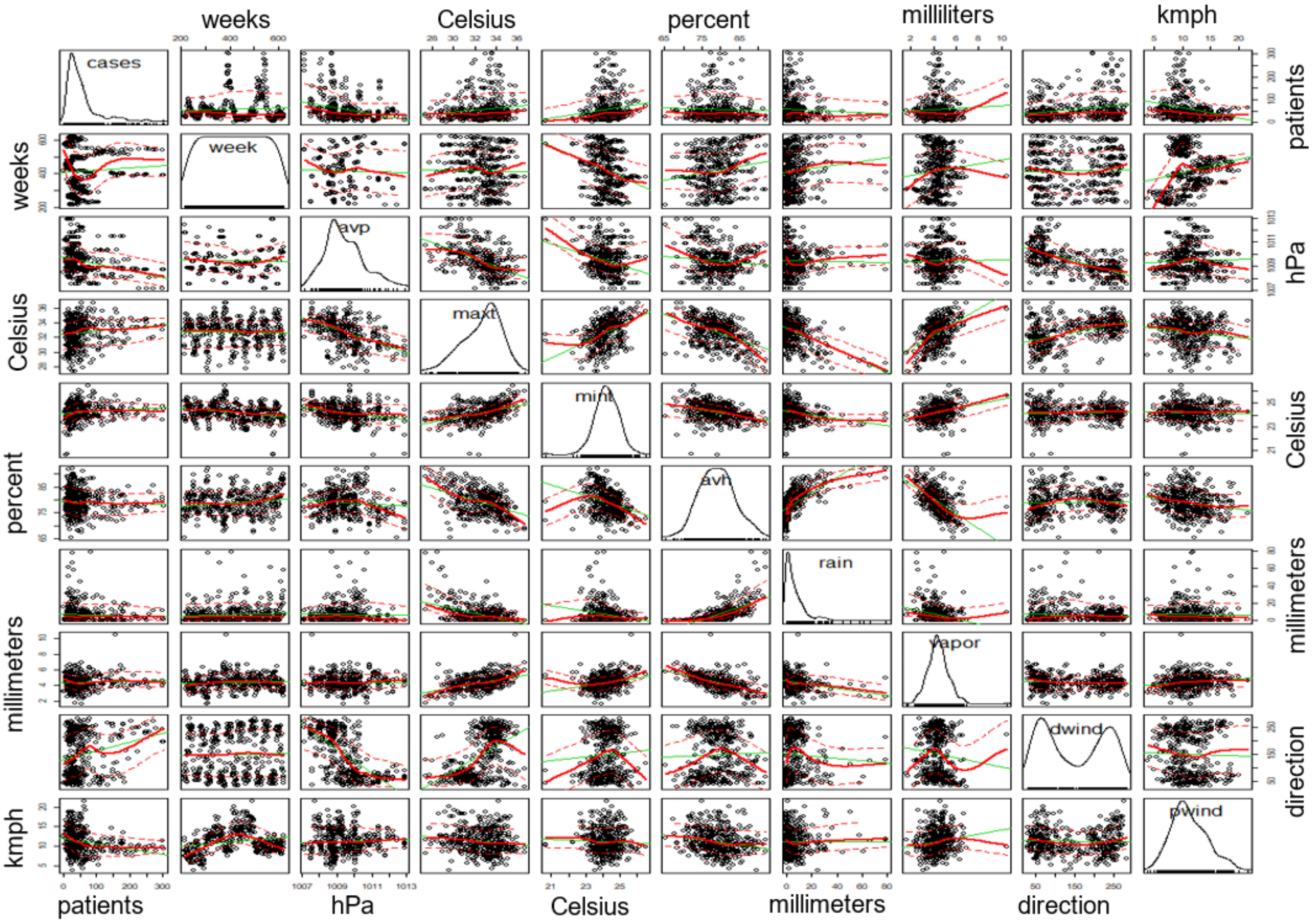

Figure 313: Scatter plot between dengue cases (cases) and selected independent variables, which are the weekly period starting from January 2001 – December 2013 (week), average pressure (avp), maximum temperature (maxt), minimum temperature (mint), average humidity (avh), precipitation (rain), vaporization of water (vapor), wind direction (dwind), and wind power (pwind). The plot visualizes pairwise hundred relationships of training set in Songkhla.

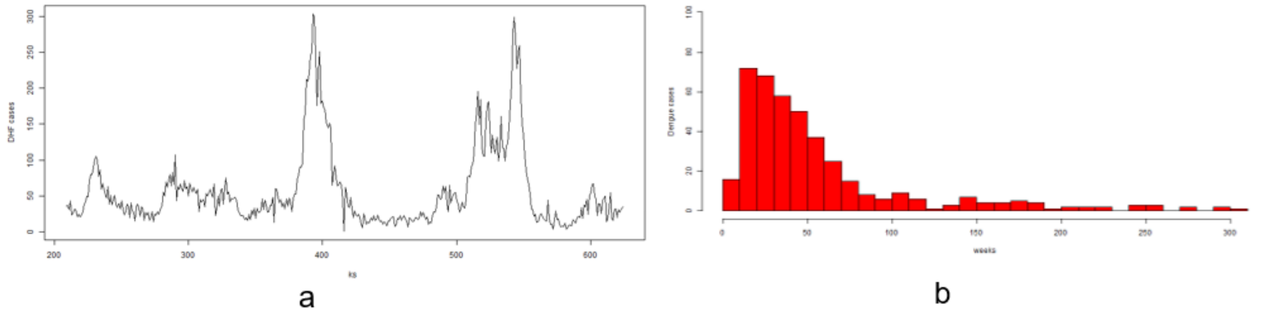

Figure 314: (a) Line plot between dengue incidences and weeks, the plot shows trends of dengue incidences in each year as stationary time series. (b) Histogram of dengue incidences in Songkhla starting from January 2001 to December 2013 (624 weeks).

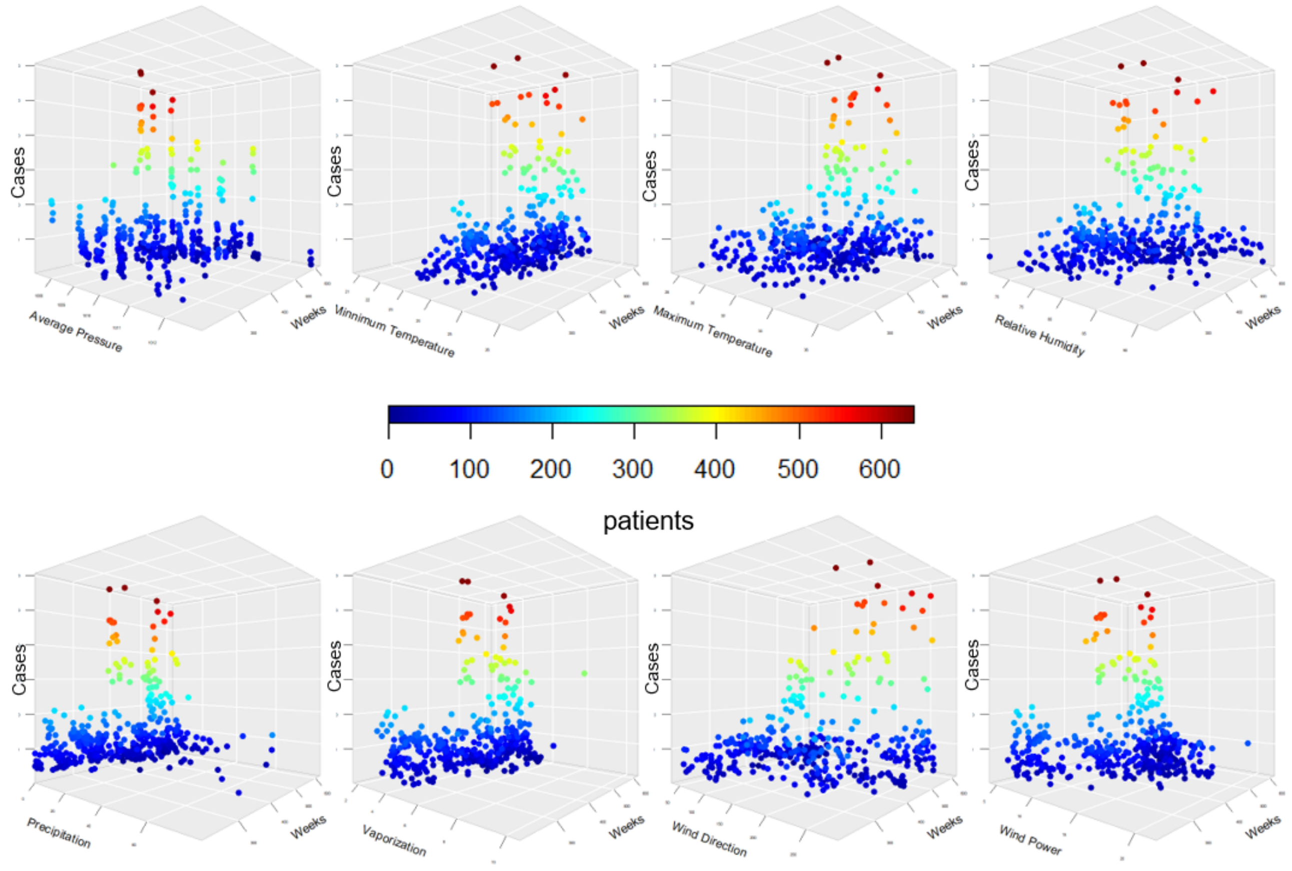

Figure 315: Three-dimensional scatter plot between dengue incidences and weather effects starting from January 2001 to December 2013 of Songkhla.

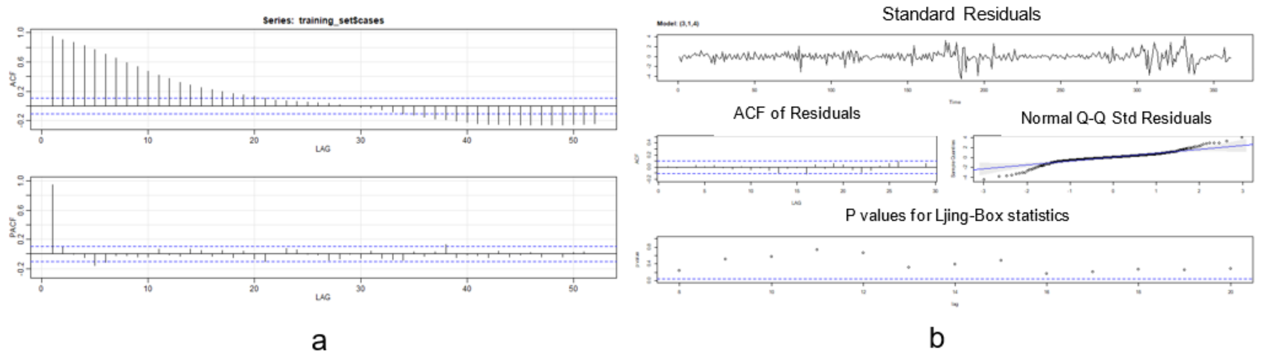

Figure 316: (a) Two plots between lag-time of dengue incidences and ACF and PACF relationship calculated from ARIMA model (b) Summary plots of time series analysis, multiple plots include the plot of predicted model over the time, the plot of ACF residual over lag-time of dengue incidences, residual Q-Q plot of standard residual, and p-value for Ljung-Box statistics of PACF relationship in Songkhla over the training data starting from January 2001 to December 2013.

For Songkhla, the best model is based on Negative Binomial regression method. The correlation coefficient on the test set in 2014 is 0.510 (95%CI: 0.4087, 0.6113). The best model of Songkhla consists of 7 variables. The most significant variables are 1-week-lag cases, following by 3-week-lag cases, current week minimum temperature and 3-week-lag vaporization. Other variables which have less significant are, 3-week-lag minimum temperature, 2-week-lag and 3-week-lag relative humidity. The significant of the variables associated with p-value statistical calculation are shown in Table 107. Time series methods by ARIMA and SARIMA yield the correlation coefficient of -3.970954 and -27.59692 respectively.

Table 105: Comparison table of all methods by the highest correlation coefficient (R^2) and the lowest prediction error (RMSE) in Songkhla.

Methods	R-squared (R^2)	Root mean square error (RMSE)
Poisson Regression	0.31609	13.17938
Negative Binomial Regression	0.5100423	11.15513
Quasi-likelihood Regression	0.4404804	11.92073
ARIMA (3,1,4)	-3.970954	35.53164
SARIMA $(2,0,1)(0,2,0)_{52}$	-27.59692	85.22264

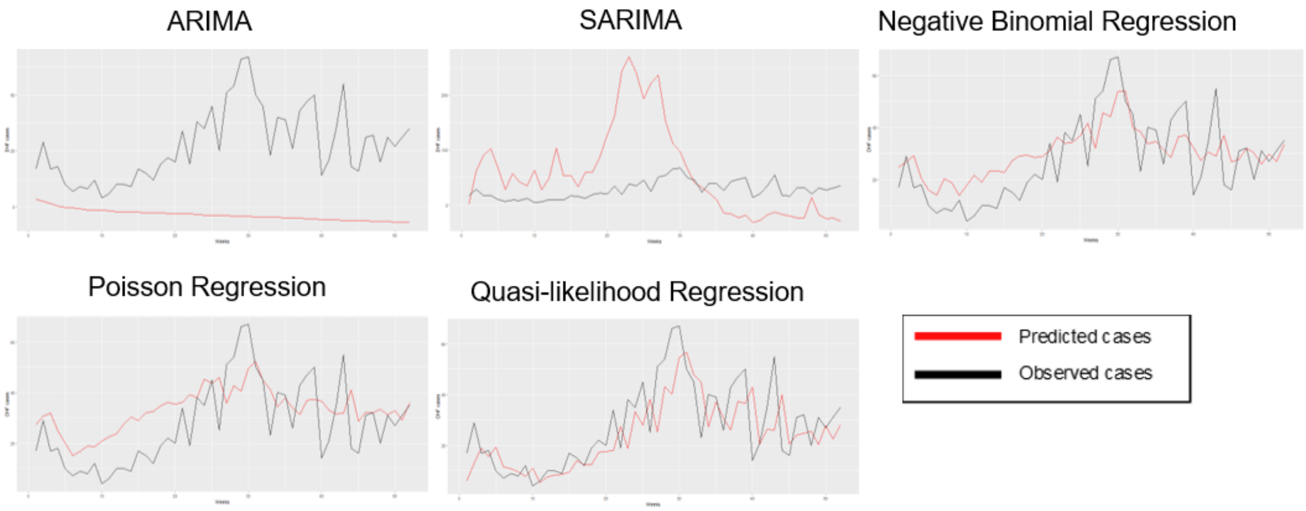

Figure 317: Plots between dengue cases and weeks, the black line represents the observed dengue cases, and the red line represents the predicted dengue cases of the best fit model of each technique over the test set data starting from January 2014 to December 2014.

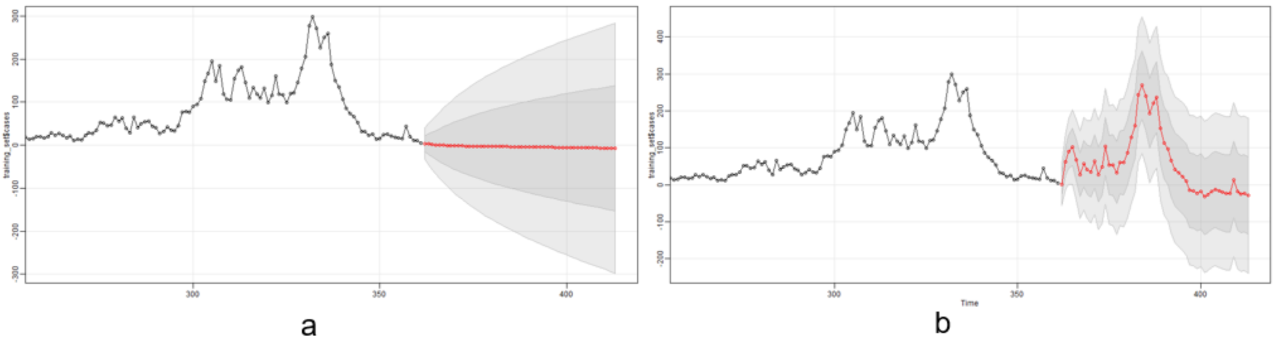

Figure 318: (a) Plot between dengue incidences over weekly time by the best model of ARIMA and (b) SARIMA time series analysis, the black line represents training set data starting from January 2012 to December 2013, and the red line represents the forecasted dengue incidences from January 2014 to December 2014.

Table 106: Coefficients and significant values of best fit GLM models, Negative Binomial, Poisson and Quasi-likelihood regression model of Songkhla. The table summarizes coefficients of each independent variables which are composed in best fit model of each method. The significant of each variable is labelled by asterisks under the coefficients. The most important factor is marked as three asterisks which p-value ranges from 0 to 0.001. The second important factor is marked as two asterisks which p-value ranges from 0.001 to 0.01. The third important factor is marked as an asterisk which p-value ranges from 0.01 to 0.1. The least important is also marked as a dot which p-value ranges from 0.1 to 1.

**Independent variables**	**Lag**	**Coefficients/Significant**		
		NB	Poisson	Quasi
Intercept		-1.6545742	-2.713 ***	189.23349 *
Cases	1	0.0093943 ***	0.007439 ***	0.63041 ***
	2		-0.0002918	
	3	0.0023858 **	0.002297 ***	0.24580 ***
Average Pressure	0			
	1			-0.62270 ***
	2			
	3			0.4405 **
Minimum Temperature	0	0.1560220 ***	0.09625 ***	
	1		0.06825 ***	
	2		0.05072 ***	
	3	0.0649368 .	0.04866 ***	
Maximum Temperature	0			
	1			
	2			-0.16458
	3		-0.01216 *	
Relative Humidity	0			
	1			
	2	0.0109433 .		
	3	0.0126677 .		
Precipitation	0		0.00009872	
	1			
	2		0.004022 ***	
	3			
Vaporization	0			
	1			
	2			0.58469 .
	3	-0.0808976 **		-0.26648
Wind Direction	0			
	1			
	2			
	3			
Wind Power	0			
	1			
	2			
	3			

Sukhothai

Sukhothai is a province located in the south-northern region of Thailand at coordinate of 17°00′51″N 99°49′19″E. Sukhothai covers an area of 6,596 km^2. Total population are 602,460 people in region. The density of population is 91.0 people per km^2. Weather in Sukhothai are under tropical savanna climate under the South Asian monsoon system. Temperature is in the range from low in December to high in April. The rainy season begins with the arrival of the southwest monsoon around mid-May

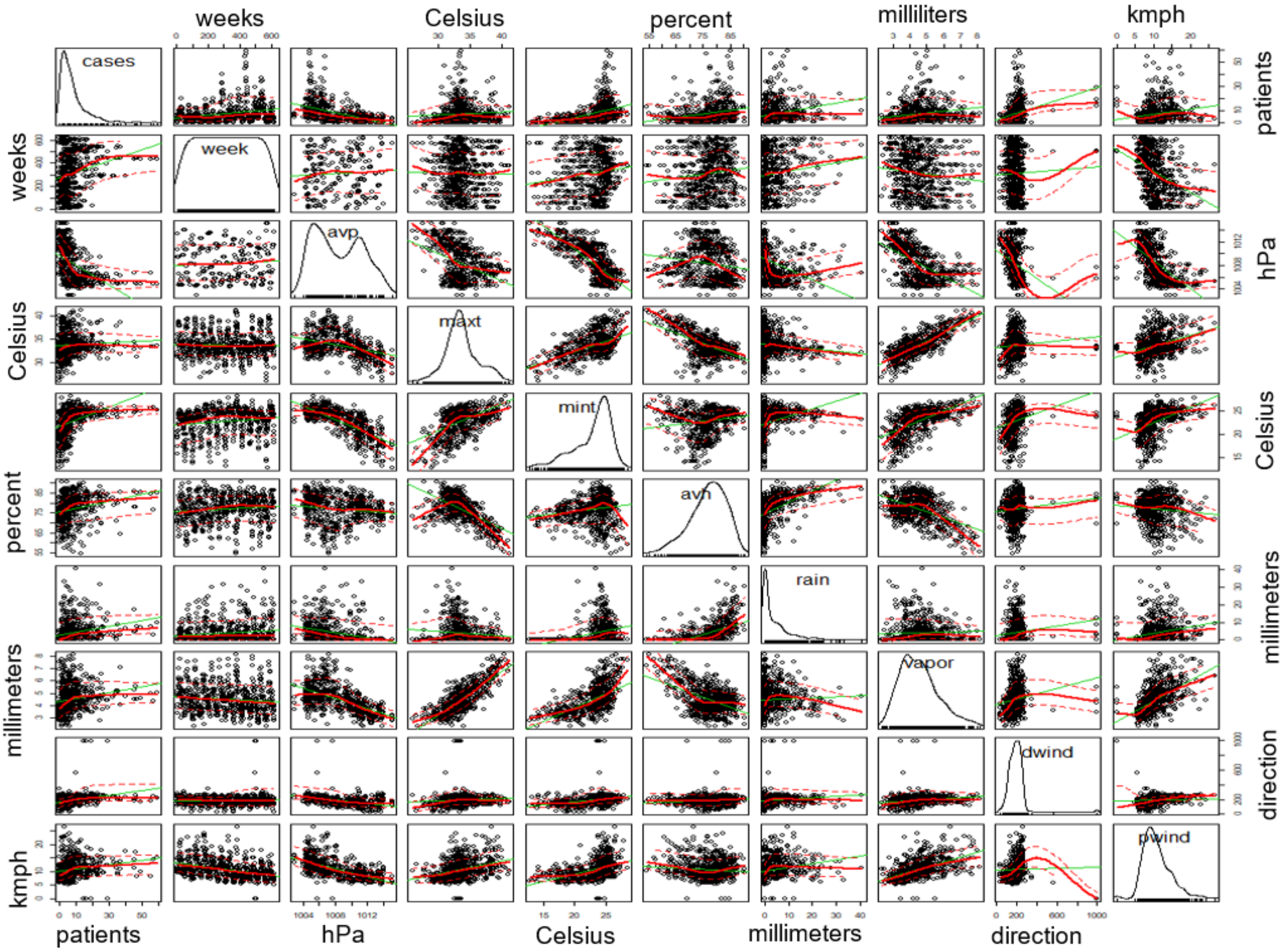

Figure 319: Scatter plot between dengue cases (cases) and selected independent variables, which are the weekly period starting from January 2001 – December 2013 (week), average pressure (avp), maximum temperature (maxt), minimum temperature (mint), average humidity (avh), precipitation (rain), vaporization of water (vapor), wind direction (dwind), and wind power (pwind). The plot visualizes pairwise hundred relationships of training set in Sukhothai.

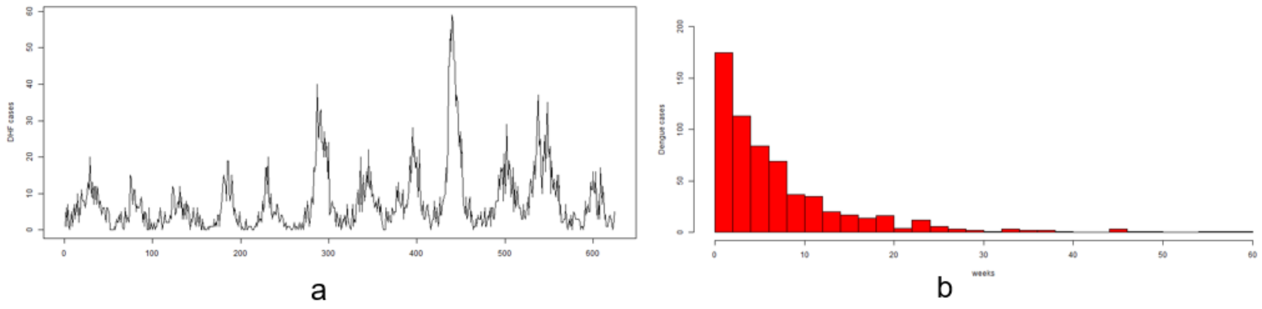

Figure 320: (a) Line plot between dengue incidences and weeks, the plot shows trends of dengue incidences in each year as stationary time series. (b) Histogram of dengue incidences in Sukhothai starting from January 2001 to December 2013 (624 weeks).

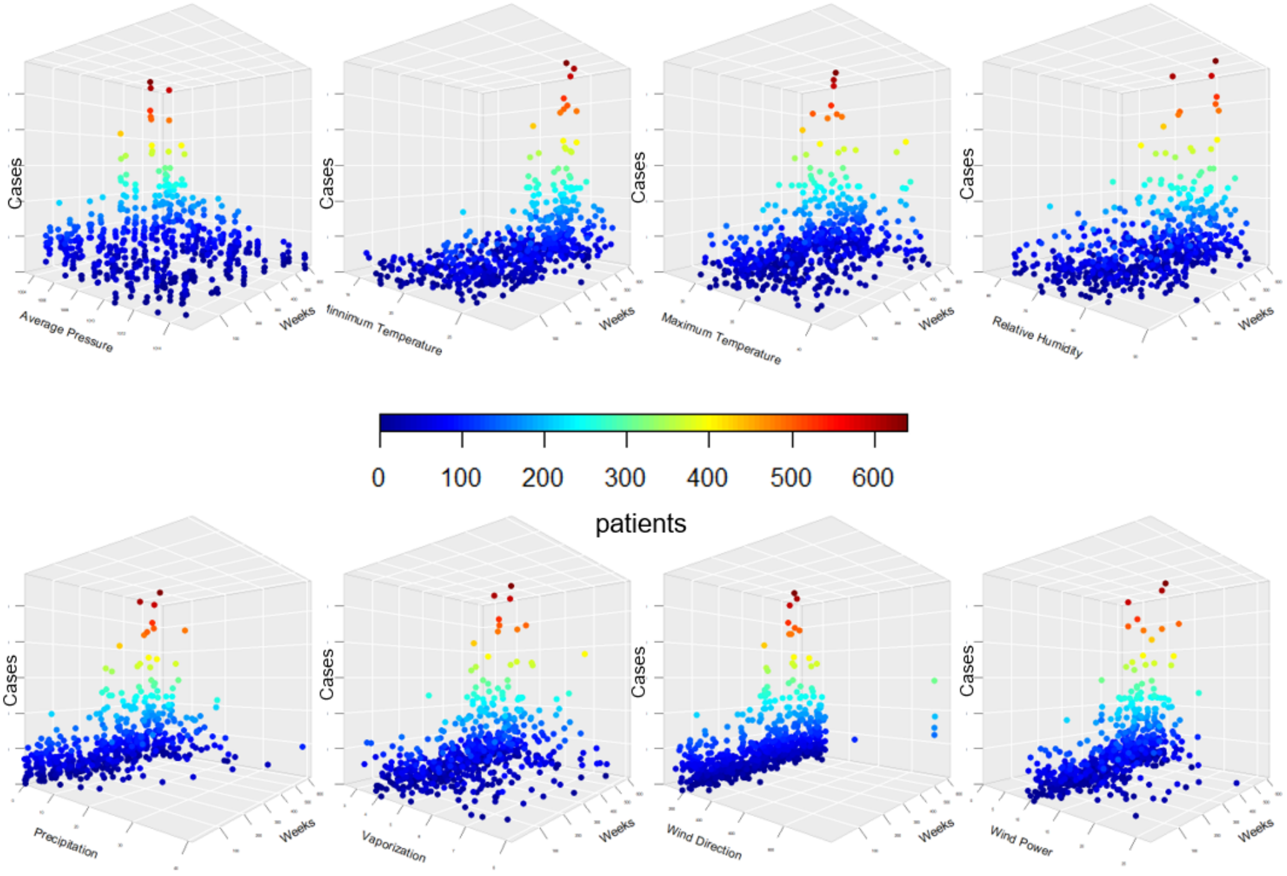

Figure 321: Three-dimensional scatter plot between dengue incidences and weather effects starting from January 2001 to December 2013 of Sukhothai.

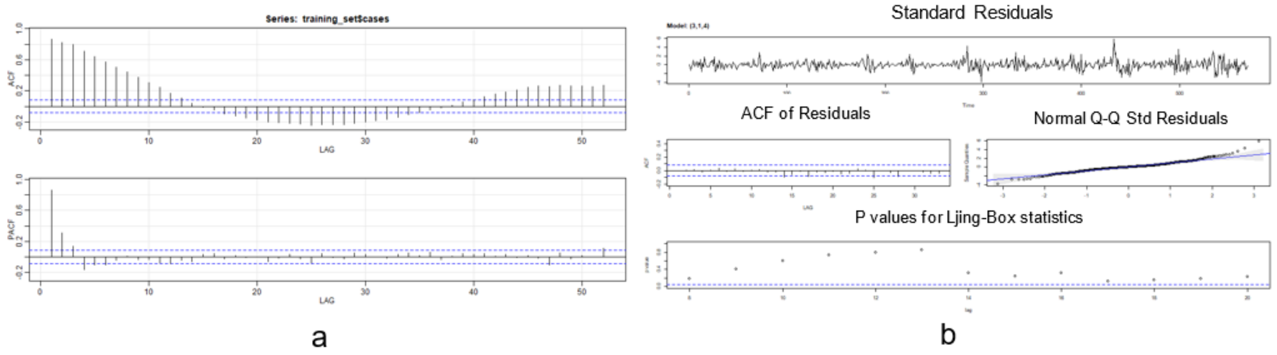

Figure 322: (a) Two plots between lag-time of dengue incidences and ACF and PACF relationship calculated from ARIMA model (b) Summary plots of time series analysis, multiple plots include the plot of predicted model over the time, the plot of ACF residual over lag-time of dengue incidences, residual Q-Q plot of standard residual, and p-value for Ljung-Box statistics of PACF relationship in Sukhothai over the training data starting from January 2001 to December 2013.

For Sukhothai, the best model is based on Negative Binomial regression method. The correlation coefficient on the test set in 2014 is 0.642 (95%CI: 0.5503, 0.7337). The significant of the variables associated with p-value statistical calculation are shown in 107. The best model uses 6 variables. The most significant variables are 3-week-lag cases, following by 3-week-lag precipitation, 2-week-lag cases, current week minimum temperature, 1-week-lag average pressure, 1-week-lag wind power and 2-week-lag precipitation. Other variables which have less significant are, current week and 3-week-lag relative humidity. Time series methods by ARIMA and SARIMA yield the correlation coefficient of -1.823073 and -6.425356 respectively.

Table 107: Comparison table of all methods by the highest correlation coefficient (R^2) and the lowest prediction error (RMSE) in sukhothai.

**Methods**	**R-squared (R^2)**	**Root mean square error (RMSE)**
Poisson Regression	0.344959	3.616425
Negative Binomial Regression	0.6417966	2.6743
Quasi-likelihood Regression	0.5247333	3.080449
ARIMA (3,1,4)	-1.823073	7.507689
SARIMA $(2,0,1)(0,2,0)_{52}$	-6.425356	12.17598

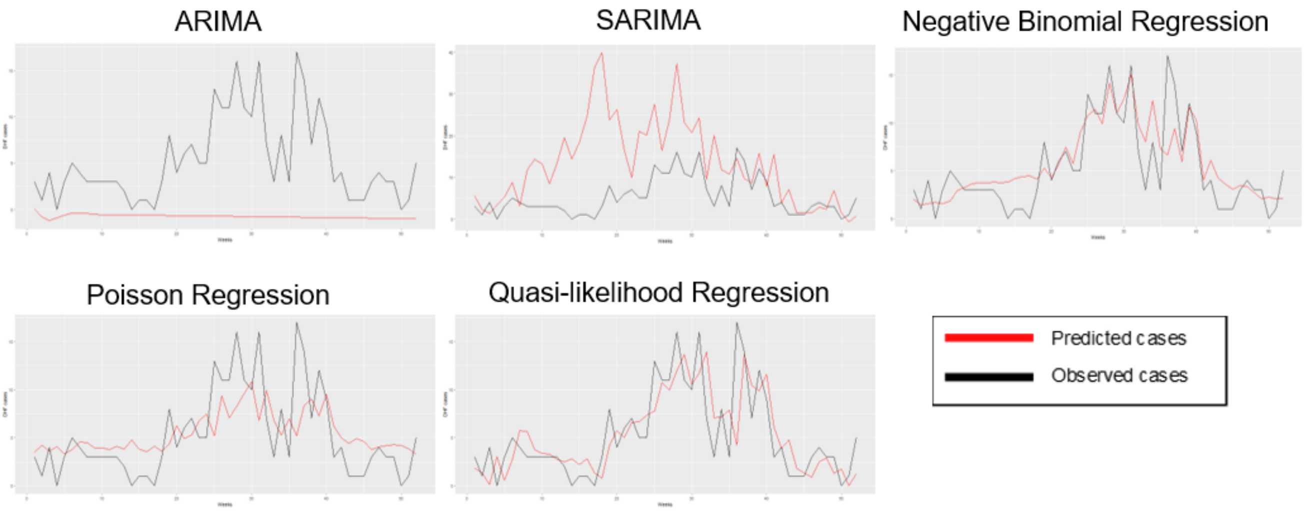

Figure 323: Plots between dengue cases and weeks, the black line represents the observed dengue cases, and the red line represents the predicted dengue cases of the best fit model of each technique over the test set data starting from January 2014 to December 2014.

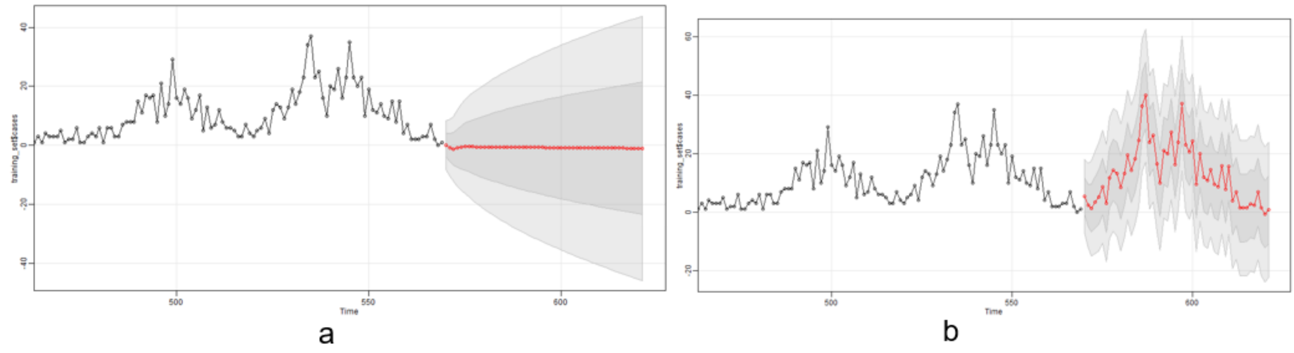

Figure 324: (a) Plot between dengue incidences over weekly time by the best model of ARIMA and (b) SARIMA time series analysis, the black line represents training set data starting from January 2012 to December 2013, and the red line represents the forecasted dengue incidences from January 2014 to December 2014.

Table 108: Coefficients and significant values of best fit GLM models, Negative Binomial, Poisson and Quasi-likelihood regression model of Sukhothai. The table summarizes coefficients of each independent variables which are composed in best fit model of each method. The significant of each variable is labelled by asterisks under the coefficients. The most important factor is marked as three asterisks which p-value ranges from 0 to 0.001. The second important factor is marked as two asterisks which p-value ranges from 0.001 to 0.01. The third important factor is marked as an asterisk which p-value ranges from 0.01 to 0.1. The least important is also marked as a dot which p-value ranges from 0.1 to 1.

**Independent variables**	**Lag**	**Coefficients/Significant**		
		NB	Poisson	Quasi
Intercept		153.13877 ***	0.4785563 *	189.23349 *
Cases	1		0.0401336 ***	0.63041 ***
	2		0.0130963 ***	
	3	0.0529880 ***		0.24580 ***
Average Pressure	0			
	1	-0.150314 ***		-0.62270 ***
	2			
	3			0.44005 **
Minimum Temperature	0			
	1			
	2			
	3			
Maximum Temperature	0			
	1			
	2			-0.16458
	3			
Relative Humidity	0	-0.004503		
	1		0.0002071	
	2		0.0049982 .	
	3	0.004381		
Precipitation	0		0.0145146 ***	
	1			
	2	0.0116320 *		
	3			
Vaporization	0			
	1			
	2			0.58469 .
	3			-0.26648
Wind Direction	0			
	1			
	2			
	3		0.0009340 ***	
Wind Power	0		0.0282312 ***	
	1	-0.024055 **		
	2			
	3			

Suphan Buri

Suphan Buri is in the central region of Thailand at coordinate of 14°28′03″N 100°07′01″E. Suphan Buri province covers an area of 5,358 km^2. Total population are 849,053 people. The density of population is 158.0 people per km^2. General weather in Suphan Buri are under tropical wet and dry climate. The highest temperature is in April approximately 41.2°C. The low temperature presents in winter from December to March approximately 3.7-18.9°C. The monsoon season starts from May through October. The highest rainfall presents in September around 223.4 mm. Humidity is in range from 71-80 percent throughout the year. The highest sunshine hours are in March.

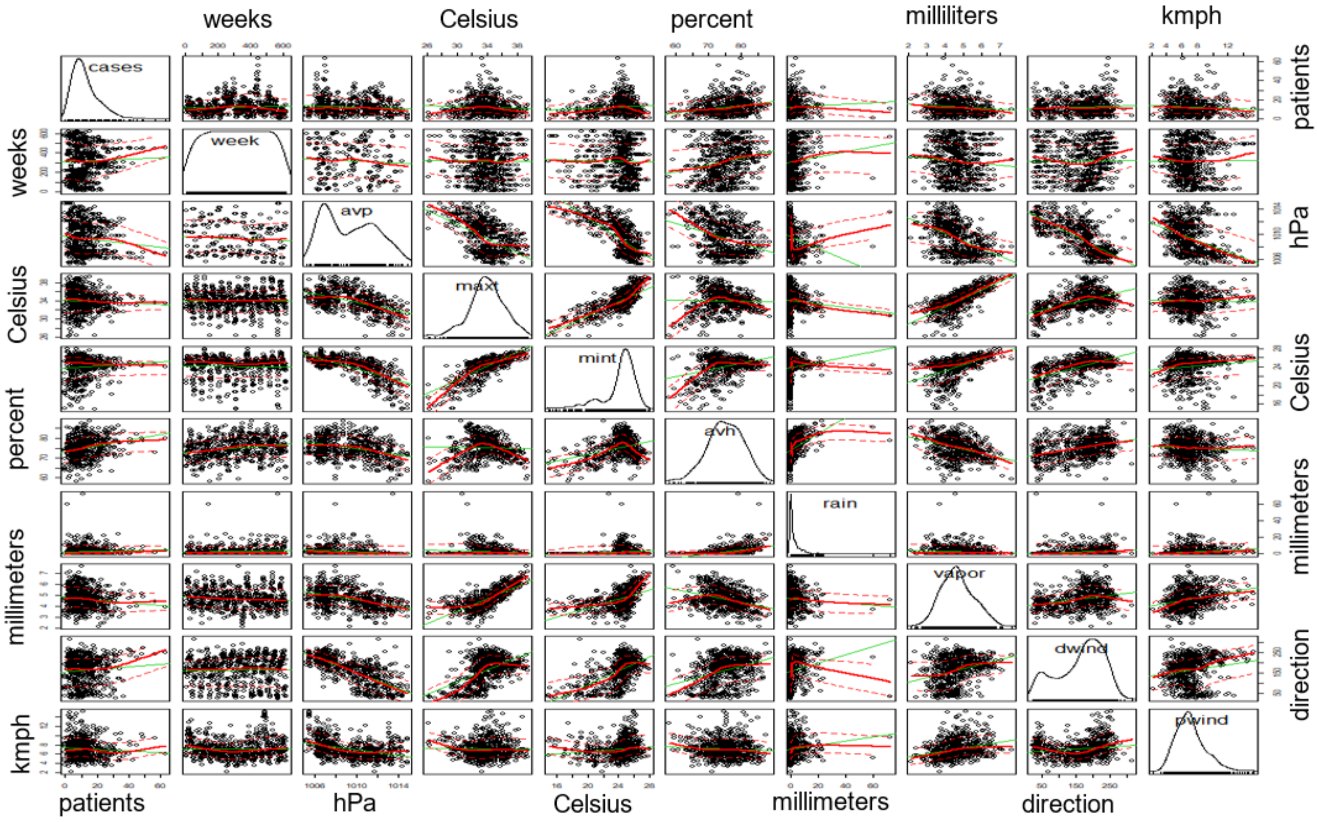

Figure 325: Scatter plot between dengue cases (cases) and selected independent variables, which are the weekly period starting from January 2001 – December 2013 (week), average pressure (avp), maximum temperature (maxt), minimum temperature (mint), average humidity (avh), precipitation (rain), vaporization of water (vapor), wind direction (dwind), and wind power (pwind). The plot visualizes pairwise hundred relationships of training set in Suphan Buri.

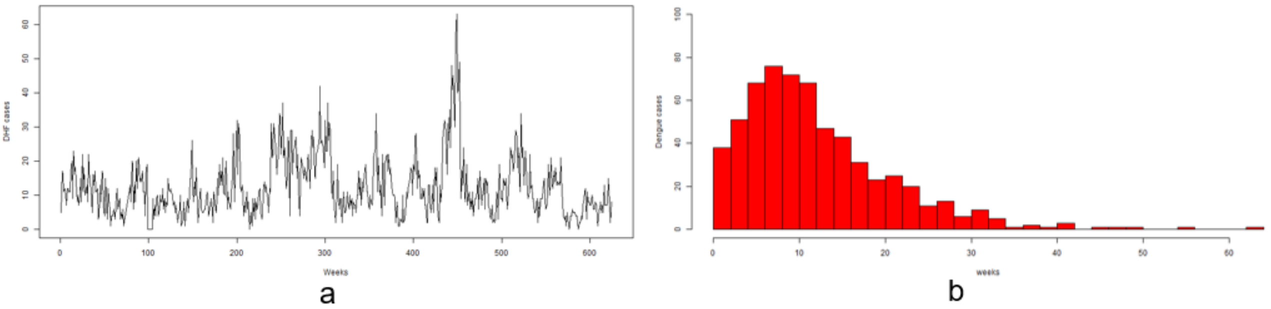

Figure 326: (a) Line plot between dengue incidences and weeks, the plot shows trends of dengue incidences in each year as stationary time series. (b) Histogram of dengue incidences in Suphan Buri starting from January 2001 to December 2013 (624 weeks).

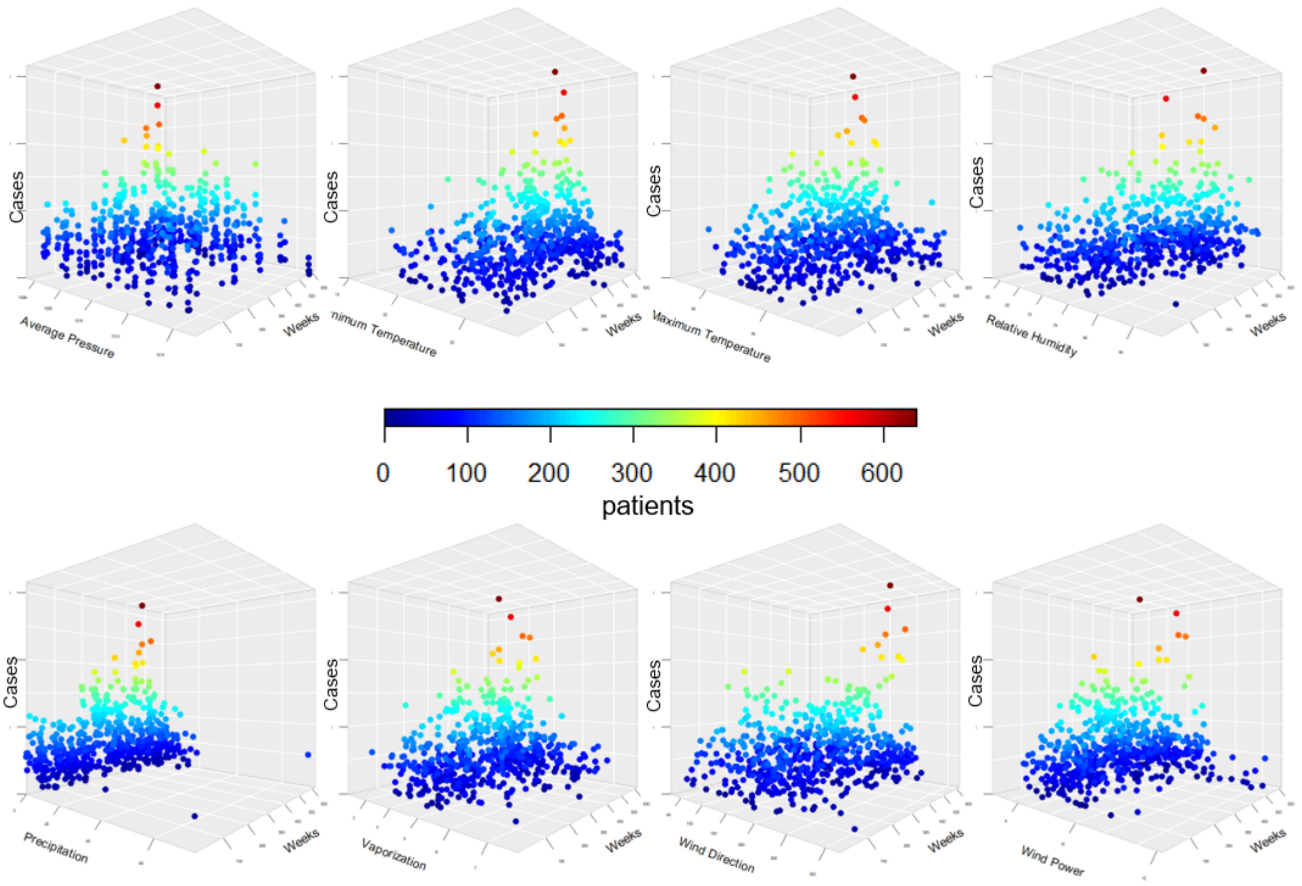

Figure 327: Three-dimensional scatter plot between dengue incidences and weather effects starting from January 2001 to December 2013 of Suphan Buri.

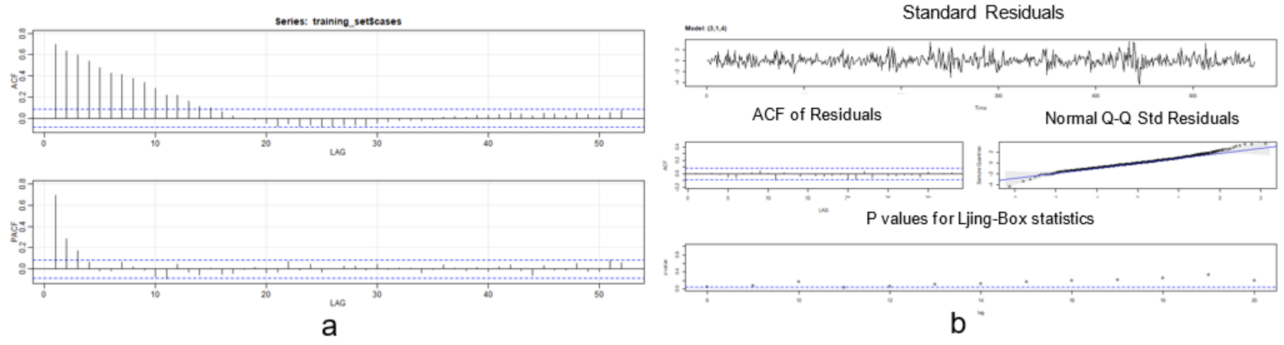

Figure 328: (a) Two plots between lag-time of dengue incidences and ACF and PACF relationship calculated from ARIMA model (b) Summary plots of time series analysis, multiple plots include the plot of predicted model over the time, the plot of ACF residual over lag-time of dengue incidences, residual Q-Q plot of standard residual, and p-value for Ljung-Box statistics of PACF relationship in Suphan Buri over the training data starting from January 2001 to December 2013.

For Suphan Buri, the best model is based on quasi-likelihood method. The correlation coefficient on the test set in 2014 yields 0.299 (95%CI: 0.1809, 0.4171). The significant of the variables associated with p-value statistical calculation are shown in Table SPB2. The best model of Suphan Buri uses 8 variables. The most significant variables are 1-week-lag cases, following by 2-week-lag cases, 3-week-lag cases, current week minimum temperature and 3-week-lag maximum temperature. Other variables which have less significant are, 1-week-lag minimum temperature, 2-week-lag maximum temperature and current week precipitation. Time series methods by ARIMA and SARIMA yield the correlation coefficient of -0.2826744 and -4.67896 respectively.

Table 109: Comparison table of all methods by the highest correlation coefficient (R^2) and the lowest prediction error (RMSE) in Suphan Buri.

**Methods**	**R-squared (R^2)**	**Root mean square error (RMSE)**
Poisson Regression	-0.2063553	3.598119
Negative Binomial Regression	-0.03847492	3.338381
Quasi-likelihood Regression	0.2993784	2.742077
ARIMA (3,1,4)	-0.2826744	3.710189
SARIMA $(2,0,1)(0,2,0)_{52}$	-4.67896	7.806786

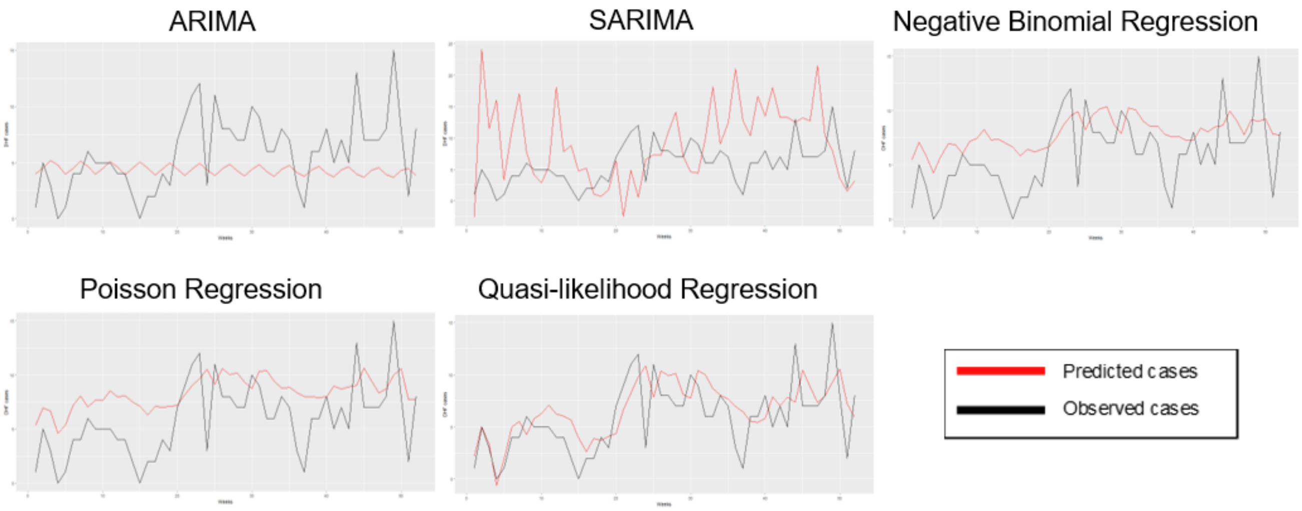

Figure 329: Plots between dengue cases and weeks, the black line represents the observed dengue cases, and the red line represents the predicted dengue cases of the best fit model of each technique over the test set data starting from January 2014 to December 2014.

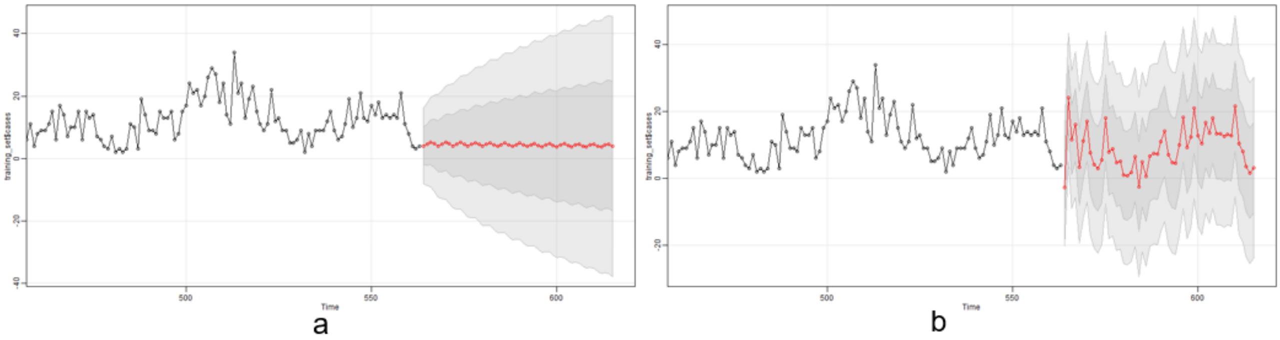

Figure 330: (a) Plot between dengue incidences over weekly time by the best model of ARIMA and (b) SARIMA time series analysis, the black line represents training set data starting from January 2012 to December 2013, and the red line represents the forecasted dengue incidences from January 2014 to December 2014.

Table 110: Coefficients and significant values of best fit GLM models, Negative Binomial, Poisson and Quasi-likelihood regression model of Suphan Buri. The table summarizes coefficients of each independent variables which are composed in best fit model of each method. The significant of each variable is labelled by asterisks under the coefficients. The most important factor is marked as three asterisks which p-value ranges from 0 to 0.001. The second important factor is marked as two asterisks which p-value ranges from 0.001 to 0.01. The third important factor is marked as an asterisk which p-value ranges from 0.01 to 0.1. The least important is also marked as a dot which p-value ranges from 0.1 to 1.

**Independent variables**	**Lag**	**Coefficients/Significant**		
		NB	Poisson	Quasi
Intercept		1.008148	-4.488459	4.94647
Cases	1	0.026729 ***	0.023671 ***	0.42370 ***
	2	0.014559 ***	0.010492 ***	0.20755 ***
	3	0.010971 ***	0.011511 ***	0.17960 ***
Average Pressure	0	0.001036	0.006439	
	1			
	2			
	3			
Minimum Temperature	0		0.046236 ***	0.53431 **
	1		0.041406 ***	0.27962
	2			
	3	0.025182		
Maximum Temperature	0	0.053183 ***	-0.022598 *	
	1			
	2			-0.20925
	3	-0.063580 ***	-0.043405 ***	-0.44175 **
Relative Humidity	0			
	1			
	2			
	3			
Precipitation	0			-0.01154
	1			
	2			
	3			
Vaporization	0			
	1			
	2			
	3			
Wind Direction	0			
	1			
	2			
	3			
Wind Power	0			
	1			
	2			
	3			

Surin

Surat Thani is located in the southern continent of Thailand at coordinate of 12°36′31″N 102°16′14″E. Surat Thani covers an area of 12,891 km^2. Total population are 1,040,230 people. The density of population is 81.0 people per km^2. General weather in Surat Thani follows tropical savanna climate under the South Asian monsoon system. The highest temperature is in May approximately 39.8°C. Temperature is stable throughout the year, although the pre-monsoon months (March–May) are rainy season. The highest rainfall presents in November around 329.9 mm. Humidity is in range from 75-87 percent throughout the year. The highest sunshine hours are in February.

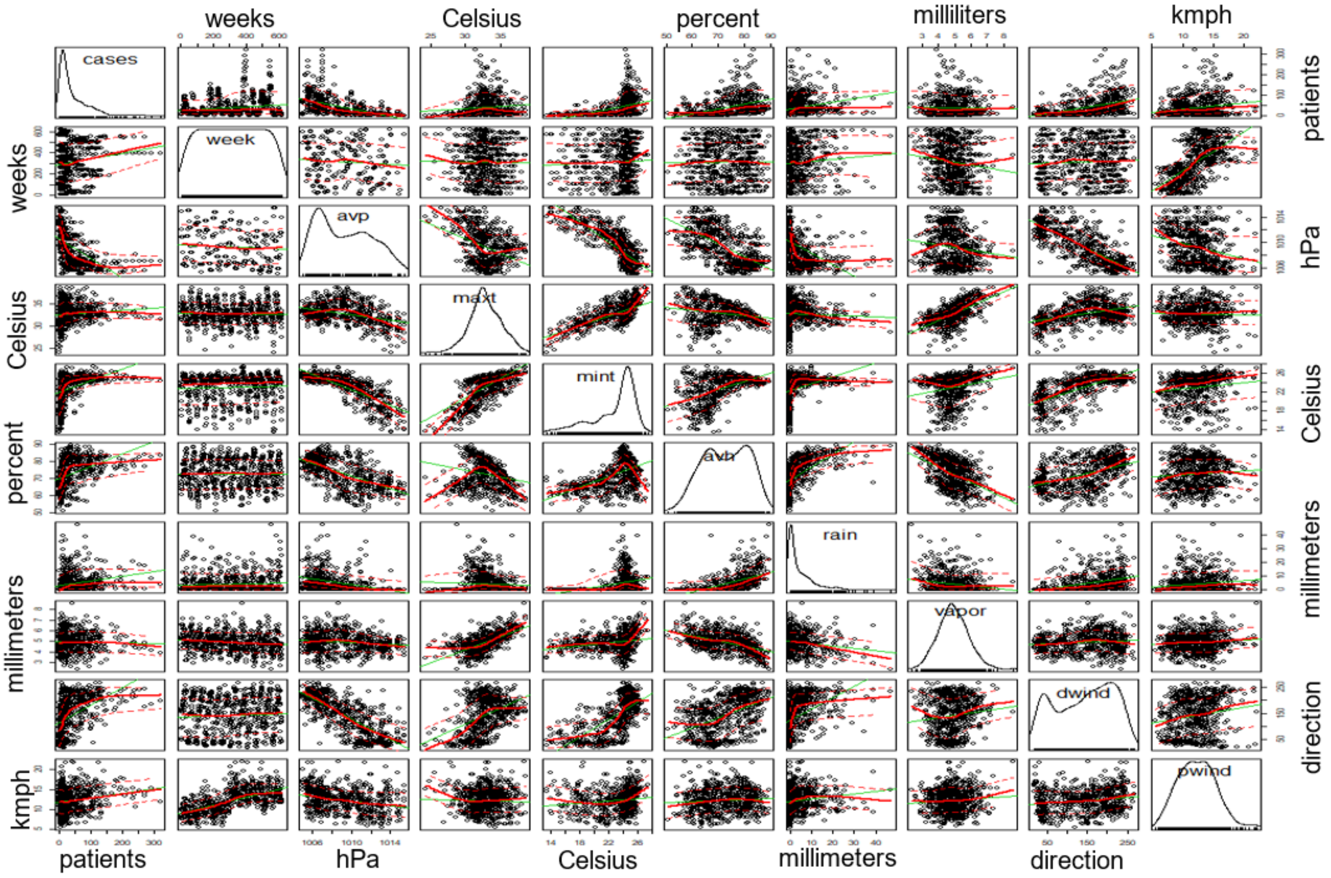

Figure 331: Scatter plot between dengue cases (cases) and selected independent variables, which are the weekly period starting from January 2001 – December 2013 (week), average pressure (avp), maximum temperature (maxt), minimum temperature (mint), average humidity (avh), precipitation (rain), vaporization of water (vapor), wind direction (dwind), and wind power (pwind). The plot visualizes pairwise hundred relationships of training set in Surin.

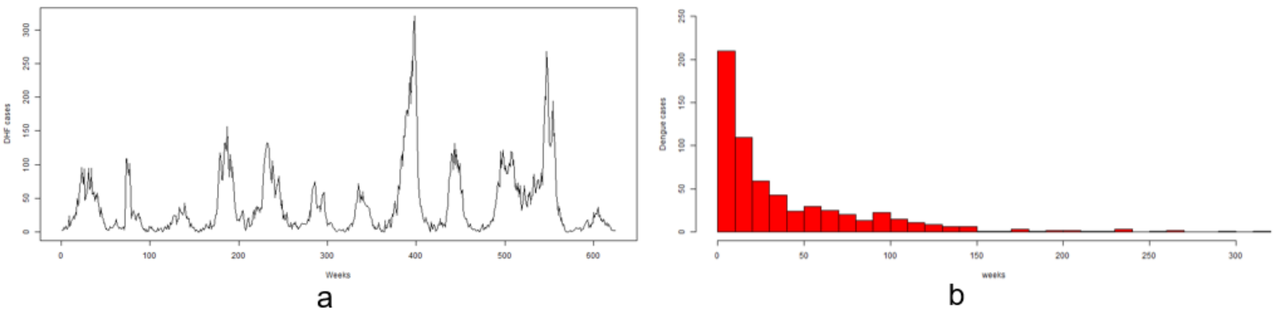

Figure 332: (a) Line plot between dengue incidences and weeks, the plot shows trends of dengue incidences in each year as stationary time series. (b) Histogram of dengue incidences in Surin starting from January 2001 to December 2013 (624 weeks).

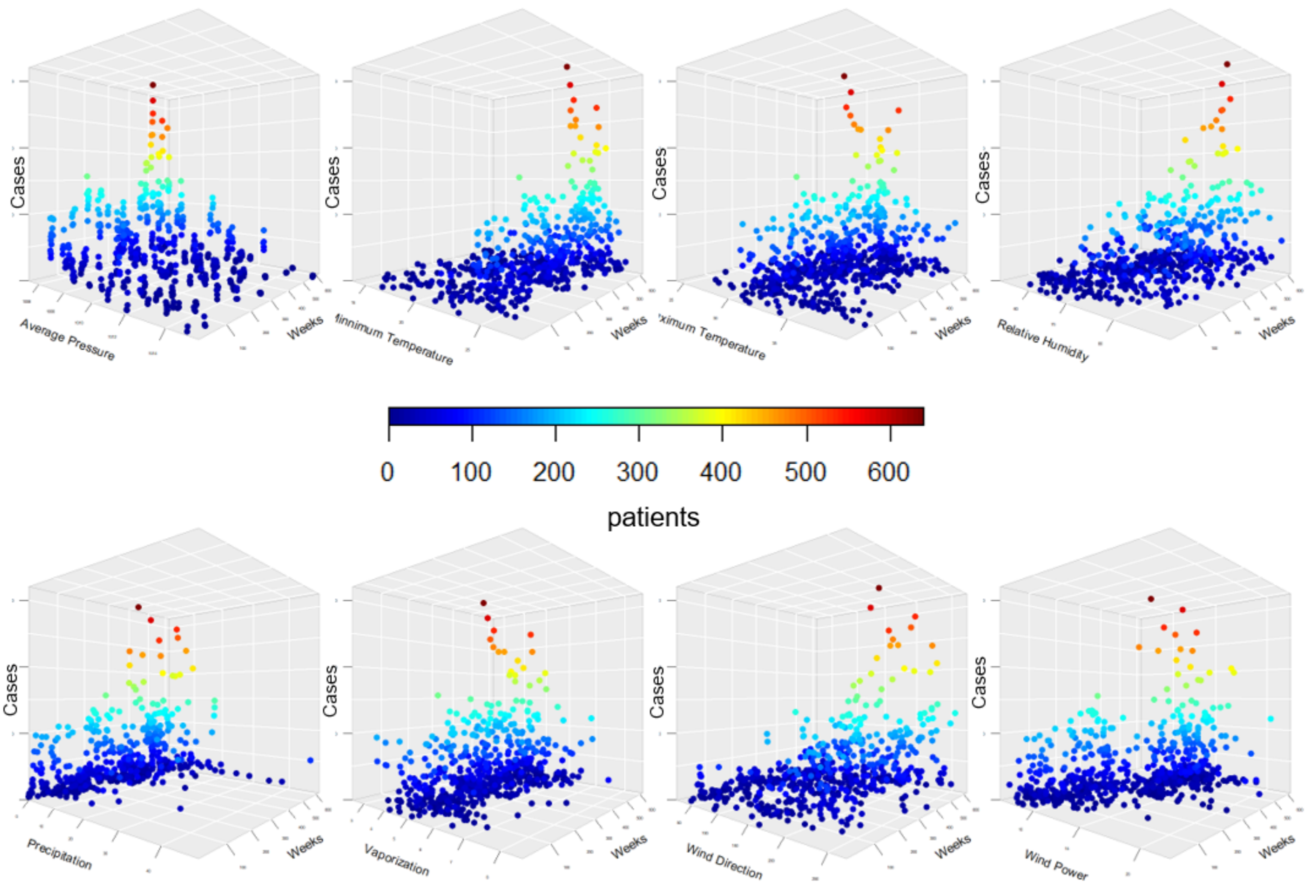

Figure 333: Three-dimensional scatter plot between dengue incidences and weather effects starting from January 2001 to December 2013 of Surin.

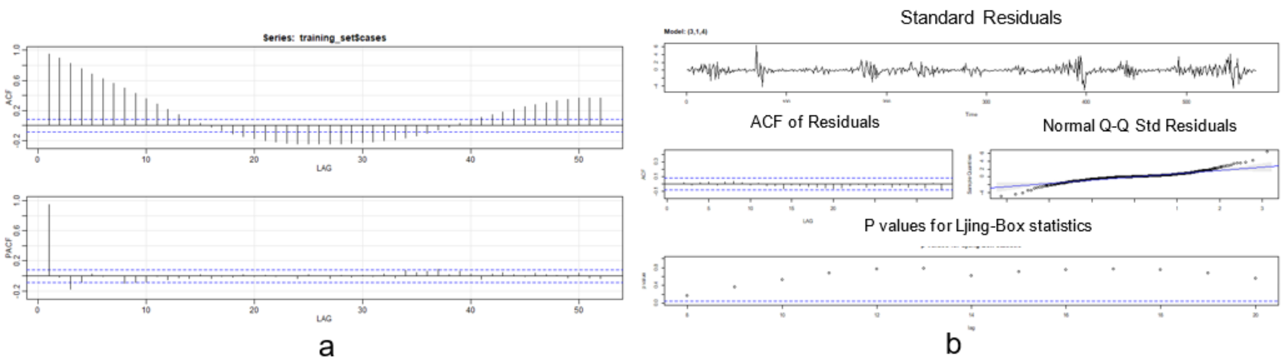

Figure 334: (a) Two plots between lag-time of dengue incidences and ACF and PACF relationship calculated from ARIMA model (b) Summary plots of time series analysis, multiple plots include the plot of predicted model over the time, the plot of ACF residual over lag-time of dengue incidences, residual Q-Q plot of standard residual, and p-value for Ljung-Box statistics of PACF relationship in Surin over the training data starting from January 2001 to December 2013.

For Surat Thani, the best model is based on quasi-likelihood method. The correlation coefficient on the test set in 2014 is 0.692 (95%CI: 0.6076, 0.7767). The significant of the variables associated with p-value statistical calculation are shown in 111. The best model uses 8 variables. The most significant variables are 1-week-lag cases, following by 3-week-lag precipitation, current week relative humidity and 3-week-lag average pressure. Other variables which have less significant are, 1-week-lag, 2-week-lag and current week precipitation, and 3-week-lag vaporization. Time series methods by ARIMA and SARIMA yield the correlation coefficient of -0.1403335 and -12.18763 respectively.

Table 111: Comparison table of all methods by the highest correlation coefficient (R^2) and the lowest prediction error (RMSE) in Surin.

Methods	R-squared (R^2)	Root mean square error (RMSE)
Poisson Regression	-0.770308	11.67319
Negative Binomial Regression	0.4834351	6.305616
Quasi-likelihood Regression	0.7006388	4.819672
ARIMA (3,1,4)	-1.397337	13.58406
SARIMA $(2,0,1)(0,2,0)_{52}$	-101.2339	88.70793

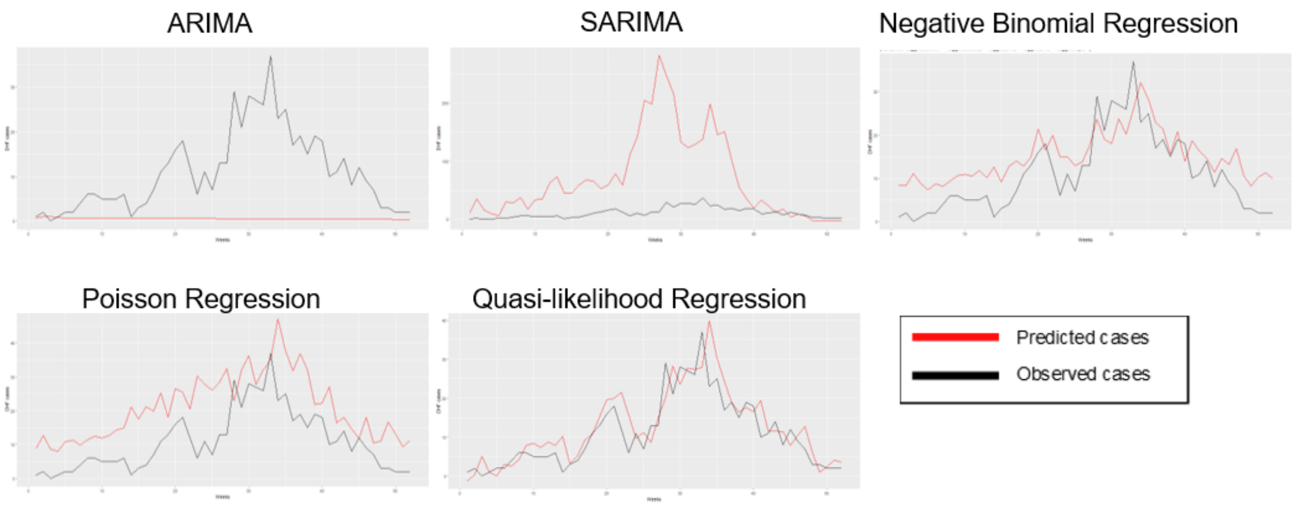

Figure 335: Plots between dengue cases and weeks, the black line represents the observed dengue cases, and the red line represents the predicted dengue cases of the best fit model of each technique over the test set data starting from January 2014 to December 2014.

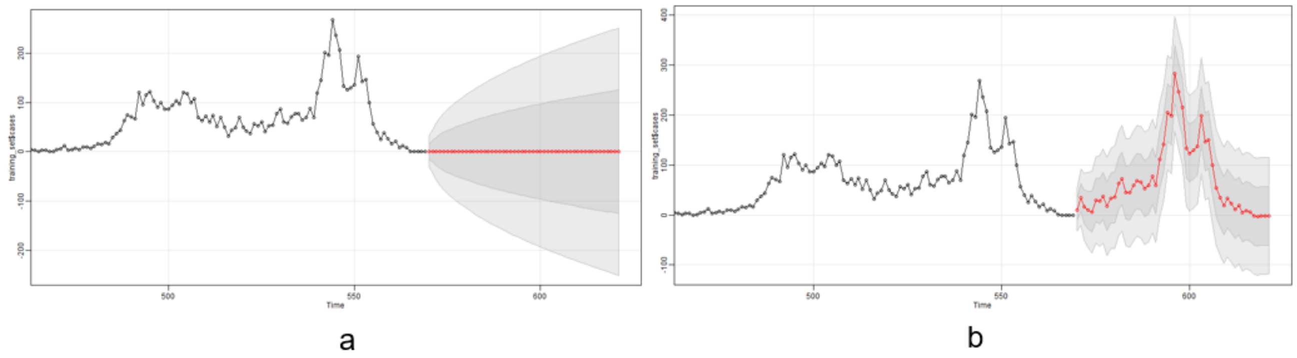

Figure 336: (a) Plot between dengue incidences over weekly time by the best model of ARIMA and (b) SARIMA time series analysis, the black line represents training set data starting from January 2012 to December 2013, and the red line represents the forecasted dengue incidences from January 2014 to December 2014.

Table 112: Coefficients and significant values of best fit GLM models, Negative Binomial, Poisson and Quasi-likelihood regression model of Surin. The table summarizes coefficients of each independent variables which are composed in best fit model of each method. The significant of each variable is labelled by asterisks under the coefficients. The most important factor is marked as three asterisks which p-value ranges from 0 to 0.001. The second important factor is marked as two asterisks which p-value ranges from 0.001 to 0.01. The third important factor is marked as an asterisk which p-value ranges from 0.01 to 0.1. The least important is also marked as a dot which p-value ranges from 0.1 to 1.

**Independent variables**	**Lag**	**Coefficients/Significant**		
		NB	Poisson	Quasi
Intercept		-0.344561	-3.265 ***	-16.96268 .
Cases	1	0.016528 ***		0.9402600 ***
	2	0.002801	0.01015 ***	0.1577700 **
	3			-0.163900 ***
Average Pressure	0			
	1			
	2			
	3			
Minimum Temperature	0			
	1			
	2			
	3			
Maximum Temperature	0			
	1			
	2			
	3			
Relative Humidity	0		0.04477 ***	
	1			
	2	0.028330 ***		0.10976
	3	-0.002912	0.01568 ***	
Precipitation	0		-0.01279 ***	
	1			
	2			
	3		-0.009532 ***	-0.02920
Vaporization	0		0.2303 ***	
	1	0.193435 ***		2.4229100 **
	2			
	3		0.1627 ***	
Wind Direction	0			
	1			
	2			
	3			
Wind Power	0			
	1			
	2			
	3			

Tak

Tak is located in north-west region of Thailand at coordinate 16°52′16″N 99°07′30″E. Tak covers an area of 16,407 km2. Total population are 539,553 people. The density of population is approximately 33.0 people per km^2. General climate in Tak has tropical savanna climate under the South Asian monsoon system. Temperature is in the range from the low of 6°C in December to the high of 44.0°C in April. The rainy season runs from May through October with heavy rain around 215.5 *mm* in September. Humidity presents from 49-82 percent throughout the year. The highest sunshine hour is in March.

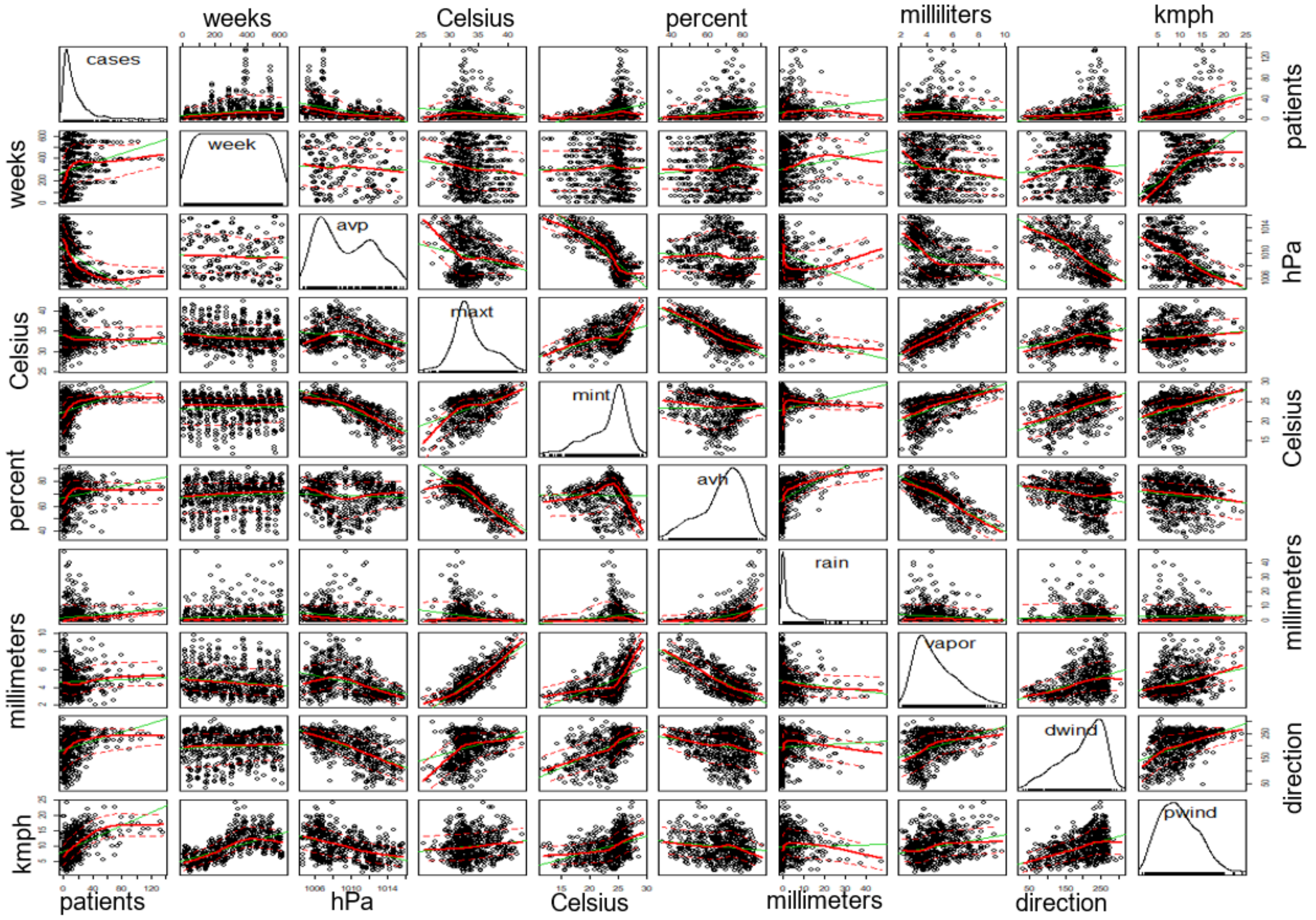

Figure 337: Scatter plot between dengue cases (cases) and selected independent variables, which are the weekly period starting from January 2001 – December 2013 (week), average pressure (avp), maximum temperature (maxt), minimum temperature (mint), average humidity (avh), precipitation (rain), vaporization of water (vapor), wind direction (dwind), and wind power (pwind). The plot visualizes pairwise hundred relationships of training set in Tak.

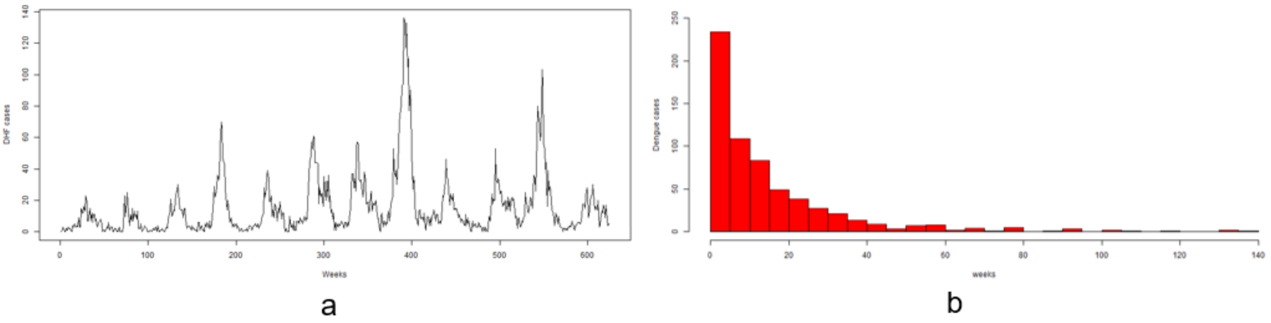

Figure 338: (a) Line plot between dengue incidences and weeks, the plot shows trends of dengue incidences in each year as stationary time series. (b) Histogram of dengue incidences in Tak starting from January 2001 to December 2013 (624 weeks).

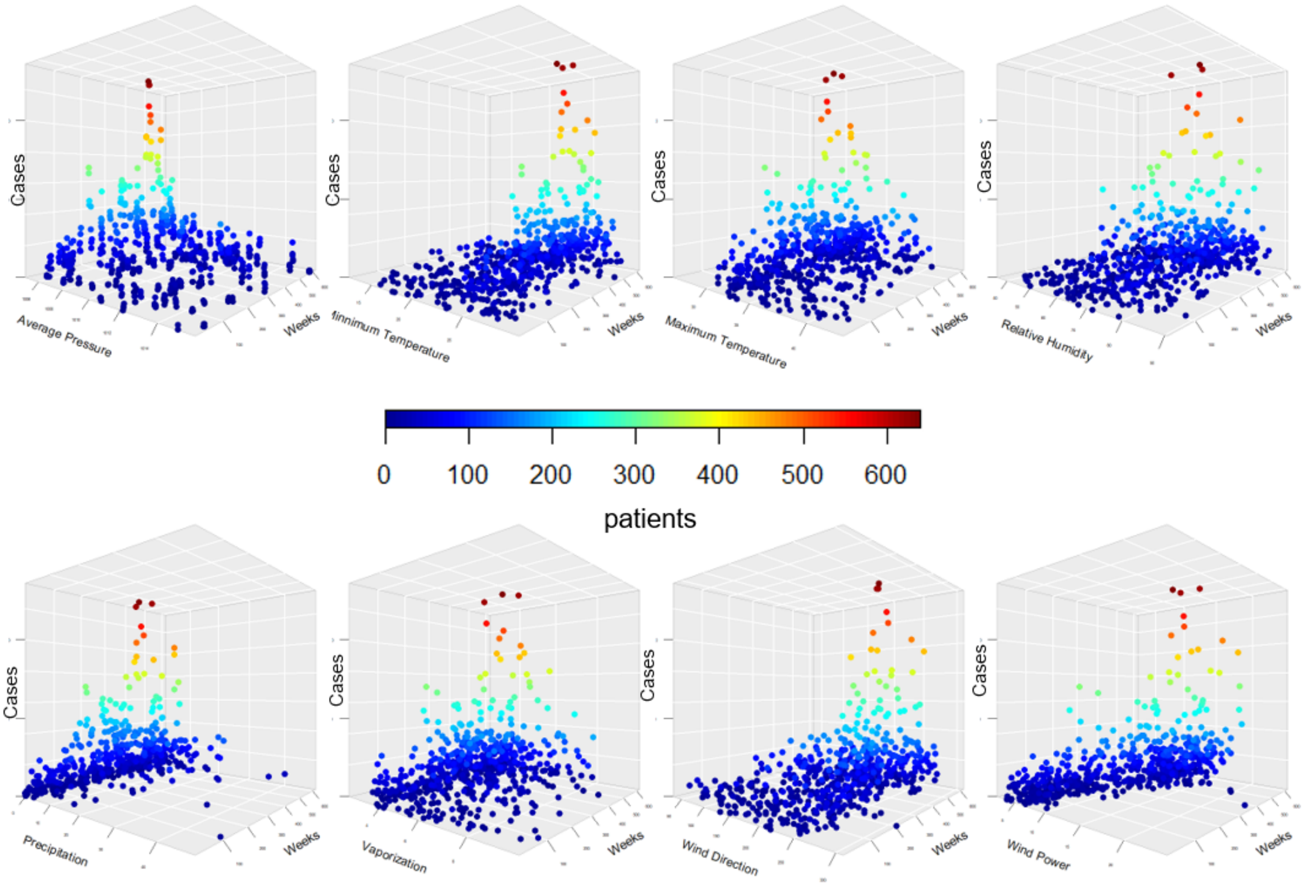

Figure 339: Three-dimensional scatter plot between dengue incidences and weather effects starting from January 2001 to December 2013 of Tak.

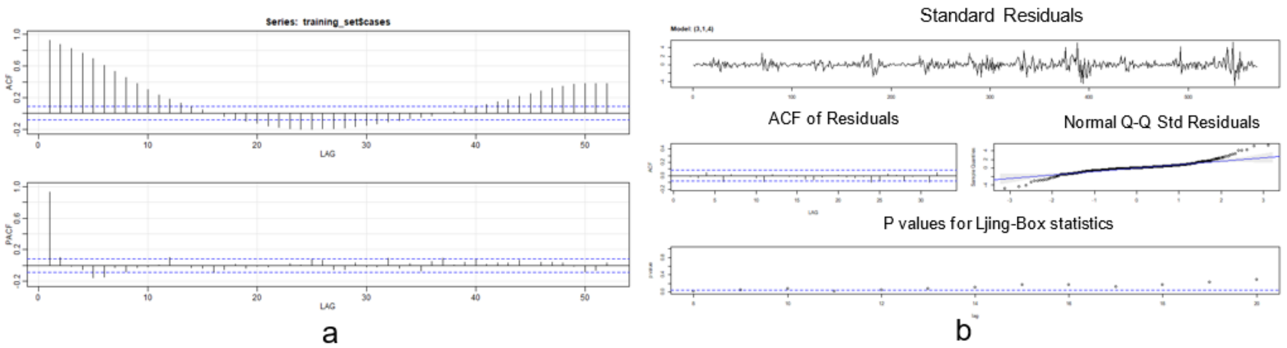

Figure 340: (a) Two plots between lag-time of dengue incidences and ACF and PACF relationship calculated from ARIMA model (b) Summary plots of time series analysis, multiple plots include the plot of predicted model over the time, the plot of ACF residual over lag-time of dengue incidences, residual Q-Q plot of standard residual, and p-value for Ljung-Box statistics of PACF relationship in Tak over the training data starting from January 2001 to December 2013.

or Tak, the best model is based on Negative Binomial regression. The correlation coefficient on the test set in 2014 is 0.769 (95%CI: 0.7174, 0.8206). The best model uses 6 weather variables. Total variables are significant to the predictive model, which are 1-week-lag cases, 3-week-lag maximum temperature, 3-week-lag relative humidity, 1-week-lag wind direction, 3-week-lag wind power and 2-week-lag wind direction significantly. Time series methods by ARIMA and SARIMA yield the correlation coefficient of -2.522444 and -12.90824 respectively.

Table 113: Comparison table of all methods by the highest correlation coefficient (R^2) and the lowest prediction error (RMSE) in Tak.

**Methods**	**R-squared (R^2)**	**Root mean square error (RMSE)**
Poisson Regression	0.725344	4.095152
Negative Binomial Regression	0.7691459	3.754432
Quasi-likelihood Regression	0.6448005	4.657058
ARIMA (3,1,4)	-2.522444	14.66552
SARIMA $(2,0,1)(0,2,0)_{52}$	-12.90824	29.14148

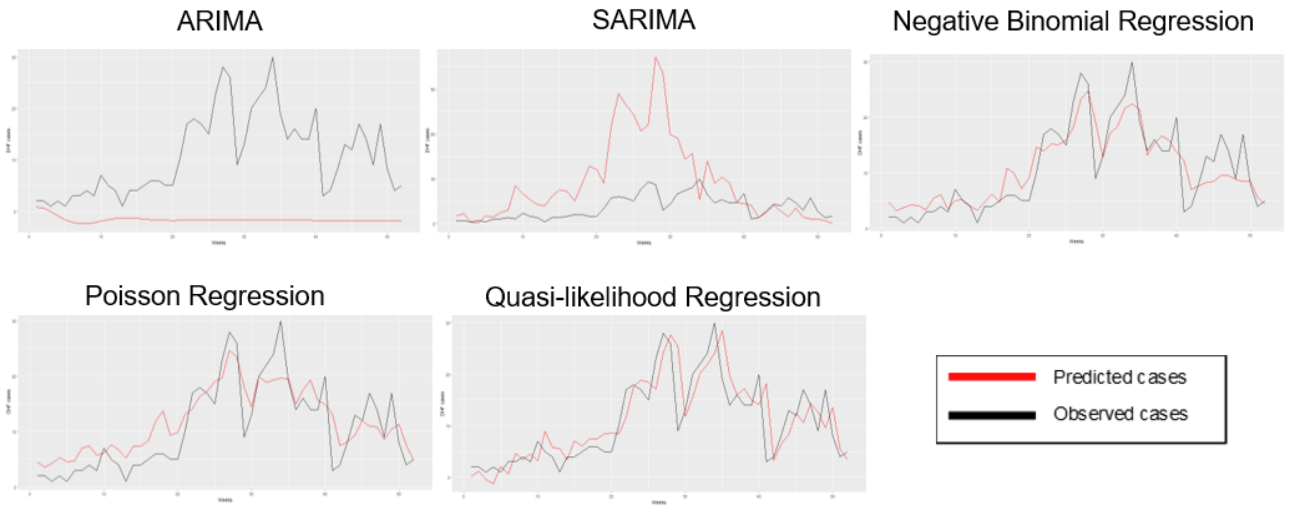

Figure 341: Plots between dengue cases and weeks, the black line represents the observed dengue cases, and the red line represents the predicted dengue cases of the best fit model of each technique over the test set data starting from January 2014 to December 2014.

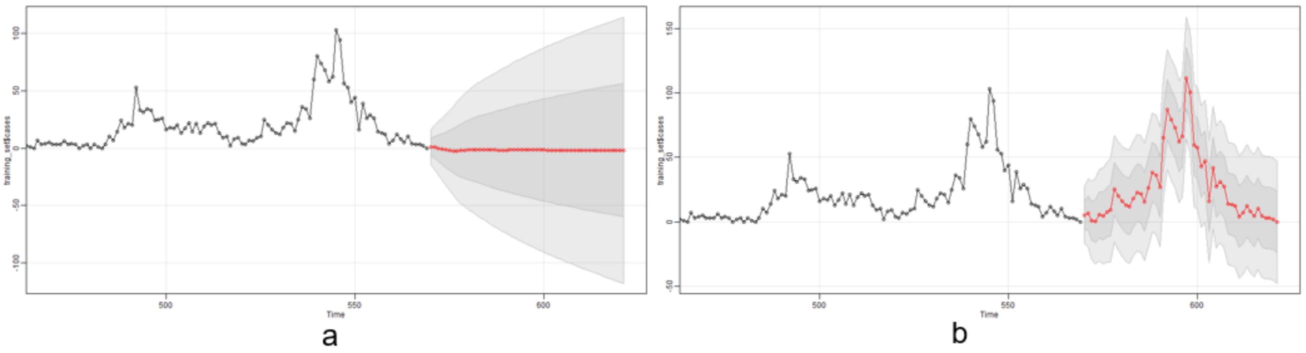

Figure 342: (a) Plot between dengue incidences over weekly time by the best model of ARIMA and (b) SARIMA time series analysis, the black line represents training set data starting from January 2012 to December 2013, and the red line represents the forecasted dengue incidences from January 2014 to December 2014.

Table 114: Coefficients and significant values of best fit GLM models, Negative Binomial, Poisson and Quasi-likelihood regression model of Tak. The table summarizes coefficients of each independent variables which are composed in best fit model of each method. The significant of each variable is labelled by asterisks under the coefficients. The most important factor is marked as three asterisks which p-value ranges from 0 to 0.001. The second important factor is marked as two asterisks which p-value ranges from 0.001 to 0.01. The third important factor is marked as an asterisk which p-value ranges from 0.01 to 0.1. The least important is also marked as a dot which p-value ranges from 0.1 to 1.

**Independent variables**	**Lag**	**Coefficients/Significant**		
		NB	Poisson	Quasi
Intercept		-4.8945636 ***	-3.1655549 ***	259.729426
Cases	1	0.0278467 ***	0.0222517 ***	0.855174 ***
	2			
	3			0.039809
Average Pressure	0			
	1			-0.260116
	2			
	3			
Minimum Temperature	0			0.386843 .
	1			-0.189416
	2			
	3			
Maximum Temperature	0		0.0083187	
	1			
	2			
	3	0.0815574 ***	0.0607138 ***	
Relative Humidity	0			
	1			
	2			
	3			
Precipitation	0			0.022927
	1			
	2			
	3			
Vaporization	0			
	1	0.0022856 ***	0.0022214 ***	
	2	0.0016987 **	0.0039086 ***	
	3			-0.009102
Wind Direction	0			
	1			
	2			
	3			
Wind Power	0			
	1			0.165850 .
	2			
	3	0.0777165		

Trang

Trang is a province located in the southern region of Thailand at 7°33′27″N 99°36′37″E. Trang covers an area of 4,918 km^2. Total population are 638,746 people. The density of population is 129.8 people per one km^2. Weather in Trang is under tropical monsoon climate system. Temperature is highest in March around 40.5°C. General range from the low temperature in Trang are from 15-17°C in December to February. The year is divided into a short dry season, from January to March, and a long-wet season from April to December, with the heaviest rain in September. The highest precipitation is in September. And the most sunshine hours present in February. Humidity presents from 72-88 percent throughout the year.

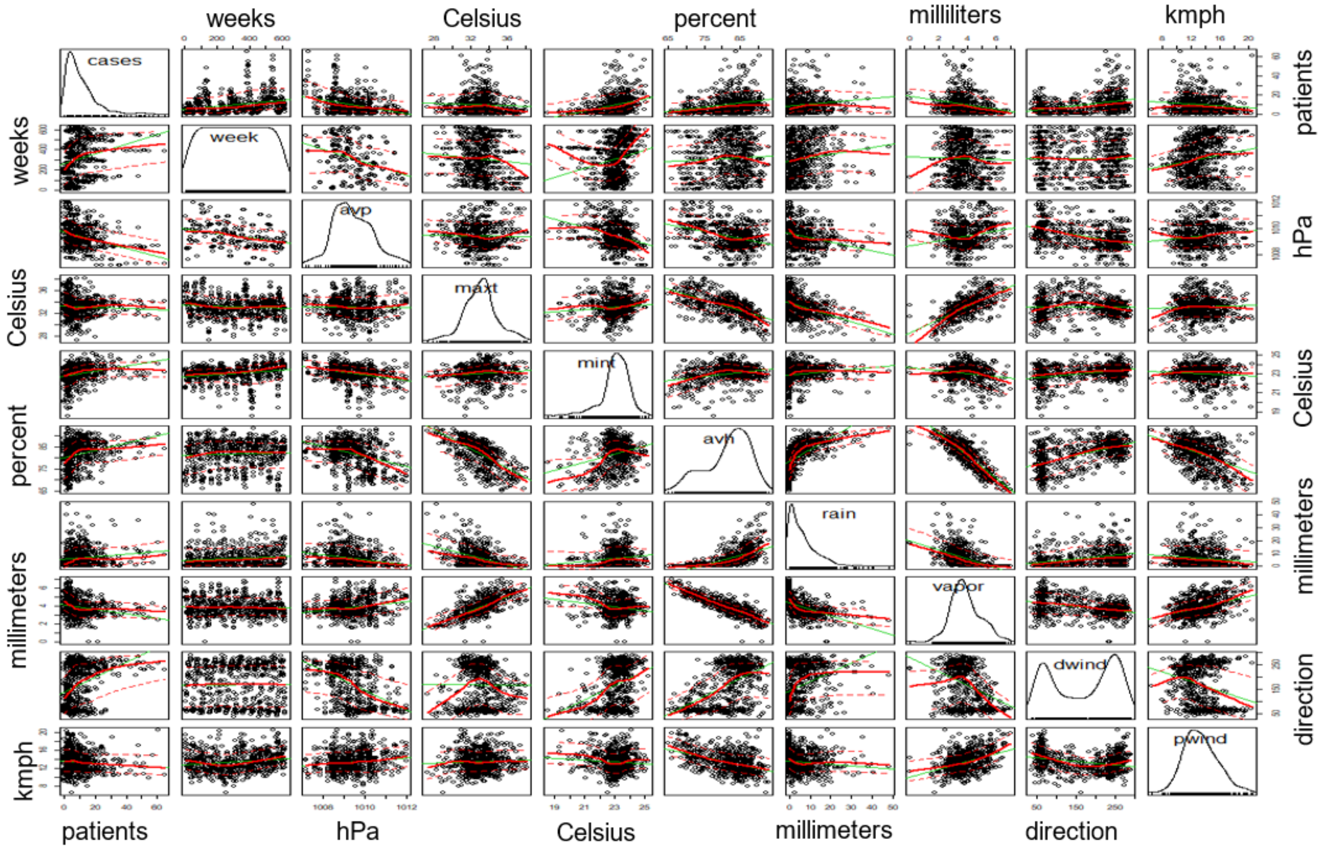

Figure 343: Scatter plot between dengue cases (cases) and selected independent variables, which are the weekly period starting from January 2001 – December 2013 (week), average pressure (avp), maximum temperature (maxt), minimum temperature (mint), average humidity (avh), precipitation (rain), vaporization of water (vapor), wind direction (dwind), and wind power (pwind). The plot visualizes pairwise hundred relationships of training set in Trang.

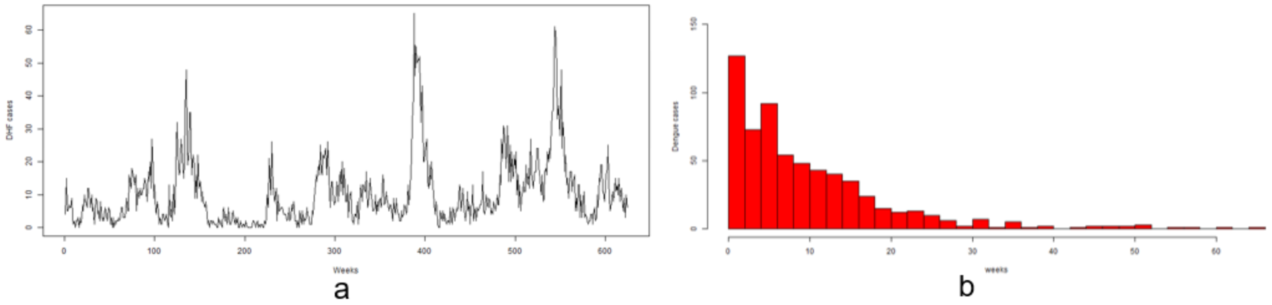

Figure 344: (a) Line plot between dengue incidences and weeks, the plot shows trends of dengue incidences in each year as stationary time series. (b) Histogram of dengue incidences in Trang starting from January 2001 to December 2013 (624 weeks).

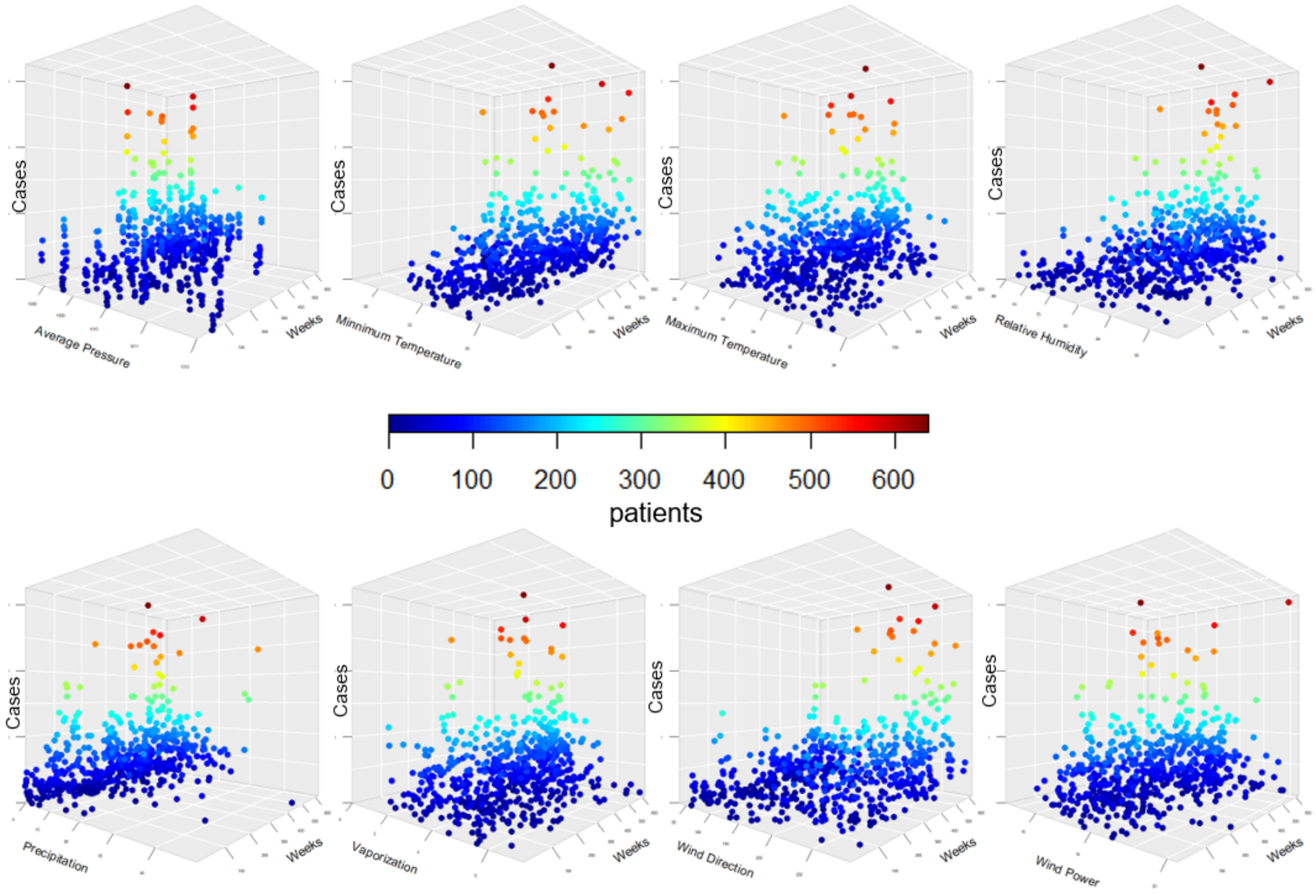

Figure 345: Three-dimensional scatter plot between dengue incidences and weather effects starting from January 2001 to December 2013 of Trang.

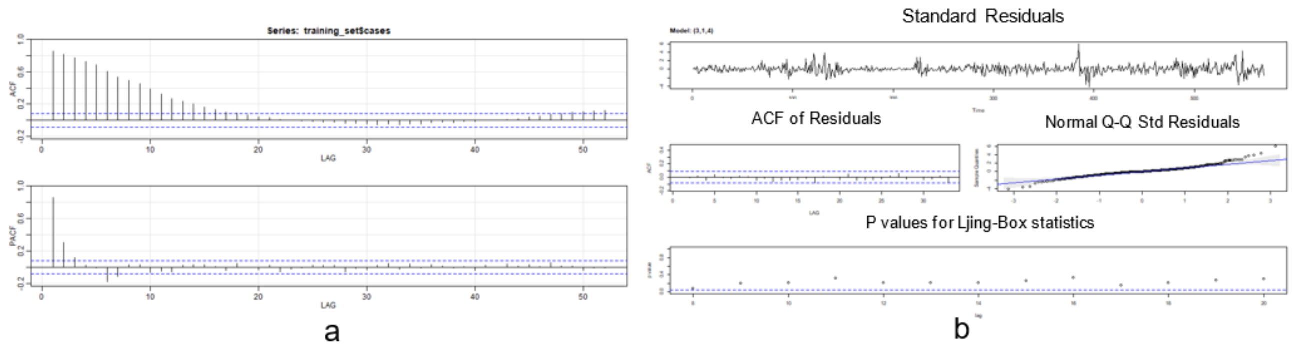

Figure 346: (a) Two plots between lag-time of dengue incidences and ACF and PACF relationship calculated from ARIMA model (b) Summary plots of time series analysis, multiple plots include the plot of predicted model over the time, the plot of ACF residual over lag-time of dengue incidences, residual Q-Q plot of standard residual, and p-value for Ljung-Box statistics of PACF relationship in Trang over the training data starting from January 2001 to December 2013.

For Trang, the best model is based on Poisson regression method. The correlation coefficient on the test set in 2014 is 0.534 (95%CI: 0.4570, 0.6110). The best model consists of 7 variables. Total variables are significant, which are 1-week-lag cases, 1-week-lag average pressure, current week maximum temperature, current week precipitation, 1-week-lag vaporization, 3-week-lag wind direction and 3-week-lag vaporization significantly. Time series methods by ARIMA and SARIMA yield the correlation coefficient of -0.1408429 and -8.966976 respectively.

Table 115: Comparison table of all methods by the highest correlation coefficient (R^2) and the lowest prediction error (RMSE) in Trang.

**Methods**	**R-squared (R^2)**	**Root mean square error (RMSE)**
Poisson Regression	0.5337086	3.676351
Negative Binomial Regression	0.5305381	3.688828
Quasi-likelihood Regression	0.4547942	3.975286
ARIMA (3,1,4)	-0.1408429	5.750441
SARIMA $(2,0,1)(0,2,0)_{52}$	-8.966976	16.99691

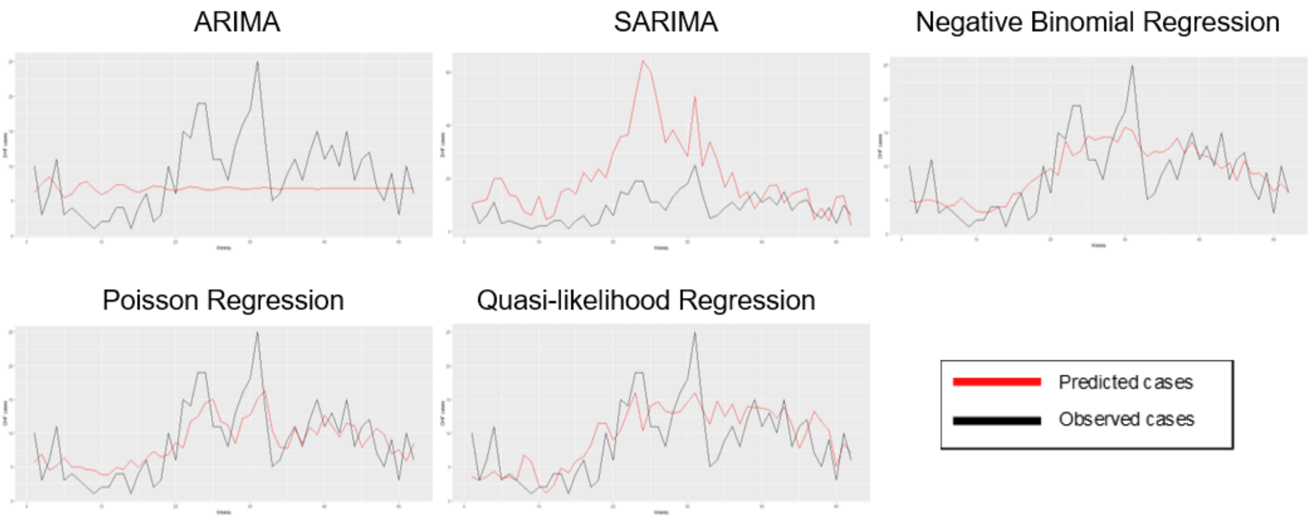

Figure 347: Plots between dengue cases and weeks, the black line represents the observed dengue cases, and the red line represents the predicted dengue cases of the best fit model of each technique over the test set data starting from January 2014 to December 2014.

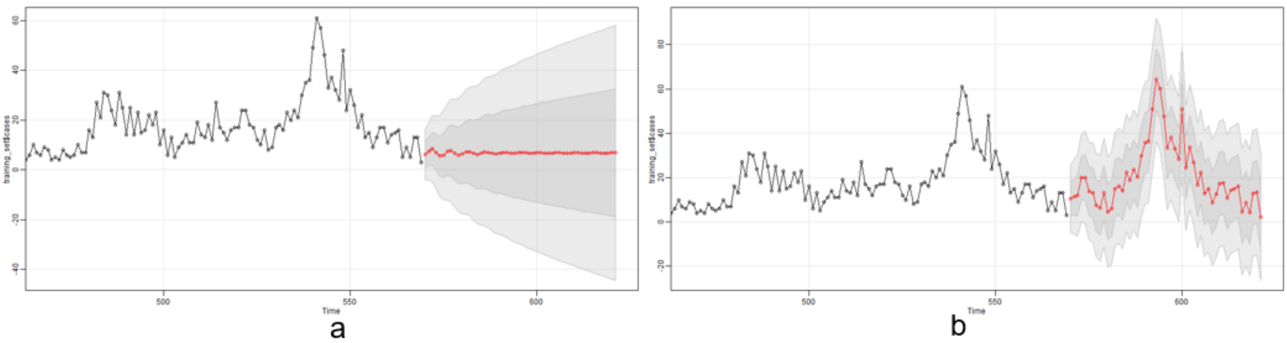

Figure 348: (a) Plot between dengue incidences over weekly time by the best model of ARIMA and (b) SARIMA time series analysis, the black line represents training set data starting from January 2012 to December 2013, and the red line represents the forecasted dengue incidences from January 2014 to December 2014.

Table 116: Coefficients and significant values of best fit GLM models, Negative Binomial, Poisson and Quasi-likelihood regression model of Trang. The table summarizes coefficients of each independent variables which are composed in best fit model of each method. The significant of each variable is labelled by asterisks under the coefficients. The most important factor is marked as three asterisks which p-value ranges from 0 to 0.001. The second important factor is marked as two asterisks which p-value ranges from 0.001 to 0.01. The third important factor is marked as an asterisk which p-value ranges from 0.01 to 0.1. The least important is also marked as a dot which p-value ranges from 0.1 to 1.

**Independent variables**	**Lag**	**Coefficients/Significant**		
		NB	Poisson	Quasi
Intercept		2.3921010 ***	84.7476063 ***	-29.389383
Cases	1		0.04130730 ***	
	2			
	3			
Average Pressure	0		-0.0837036 ***	
	1			
	2			
	3			
Minimum Temperature	0			
	1			
	2			
	3			
Maximum Temperature	0		0.05635600 ***	0.975770 **
	1			
	2			
	3			
Relative Humidity	0			
	1			
	2			0.111624
	3			
Precipitation	0		0.00721340 ***	
	1			
	2	-0.0061690		-0.071939
	3			-0.075106
Vaporization	0			0.116028 .
	1		-0.1039130 ***	
	2	-0.1548429 ***		-1.303685
	3		-0.0537221 **	-0.674926
Wind Direction	0	0.0021215 **		
	1			
	2	0.0014232		
	3	0.0014784 .	0.00093550 ***	0.036758 ***
Wind Power	0			
	1			
	2			
	3	-0.0271206		

Trat

Trat is located in the eastern region of Thailand at coordinates 12°24′N 102°31′E. Trat covers an area of 2,819 km^2. Total population are 224,730 people. The density of population is 80.0 people per one km^2. Trat geography is around by the island. General weather in Trat has tropical savanna climate under the South Asian monsoon system. Temperature is in the range from low in December to high in April. The rainy season begins with the arrival of the southwest monsoon around mid-May.

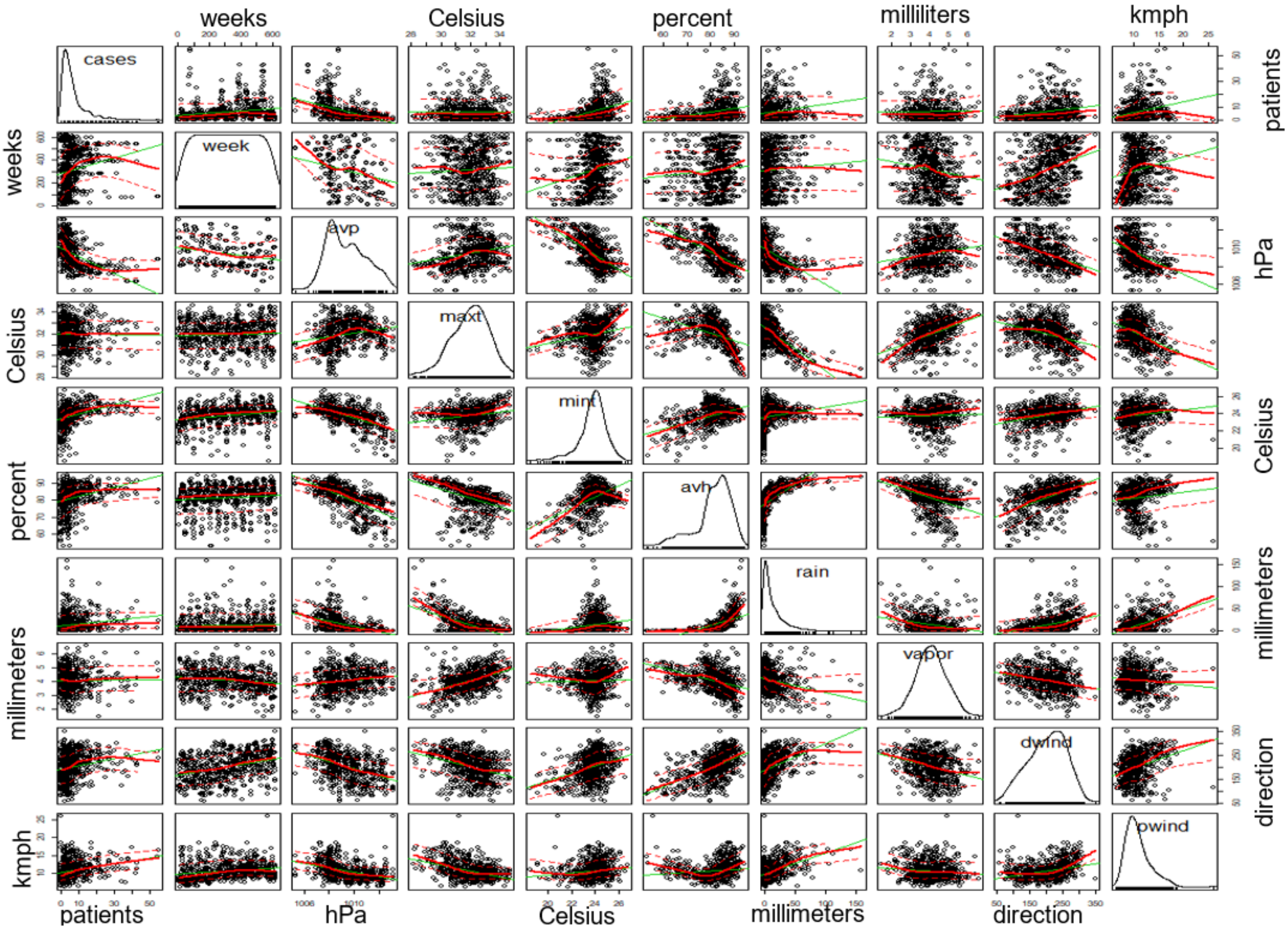

Figure 349: Scatter plot between dengue cases (cases) and selected independent variables, which are the weekly period starting from January 2001 – December 2013 (week), average pressure (avp), maximum temperature (maxt), minimum temperature (mint), average humidity (avh), precipitation (rain), vaporization of water (vapor), wind direction (dwind), and wind power (pwind). The plot visualizes pairwise hundred relationships of training set in Trat.

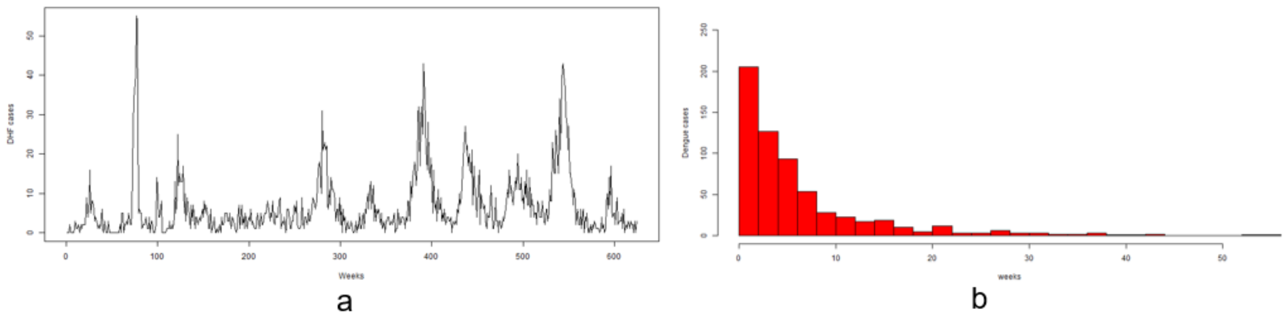

Figure 350: (a) Line plot between dengue incidences and weeks, the plot shows trends of dengue incidences in each year as stationary time series. (b) Histogram of dengue incidences in Trat starting from January 2001 to December 2013 (624 weeks).

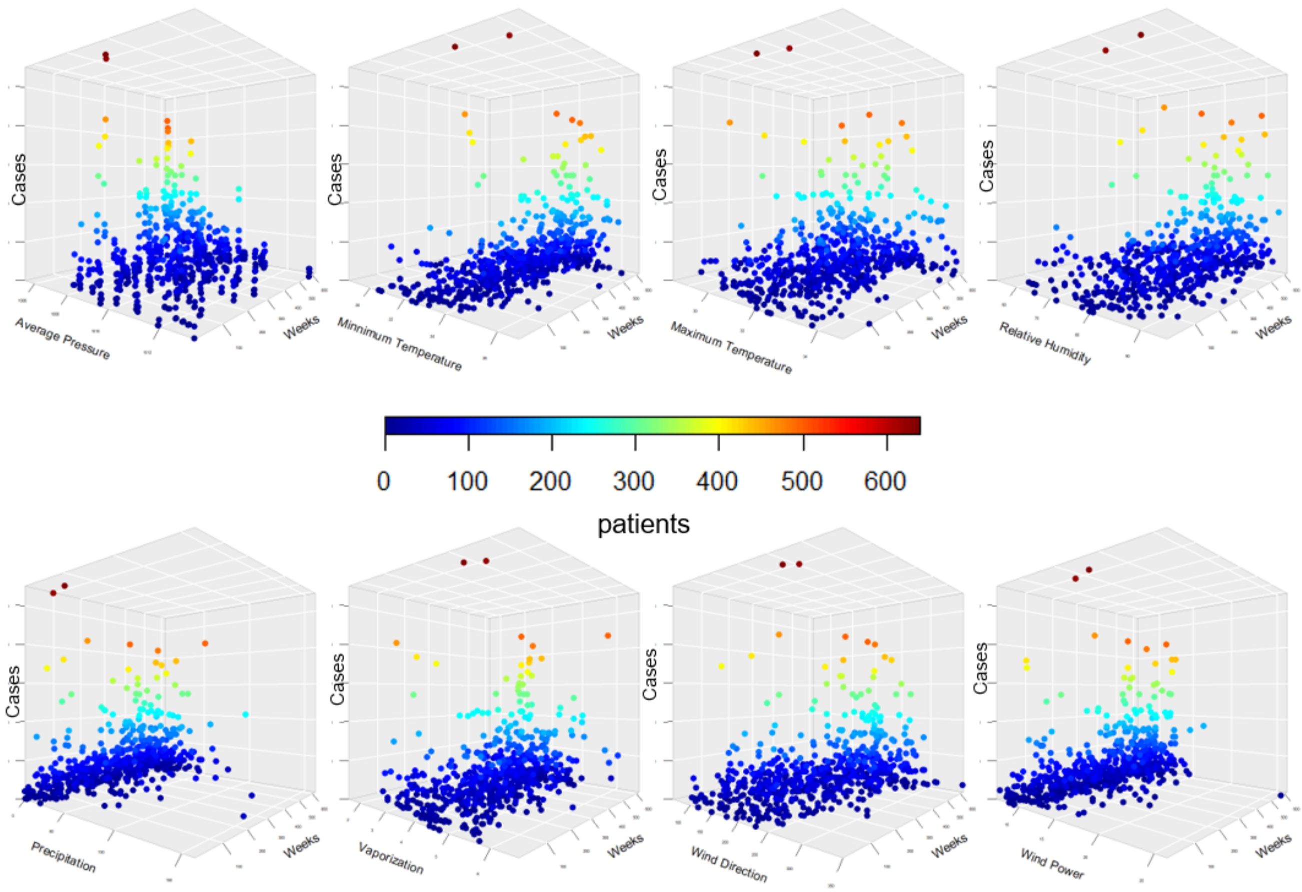

Figure 351: Three-dimensional scatter plot between dengue incidences and weather effects starting from January 2001 to December 2013 of Trat.

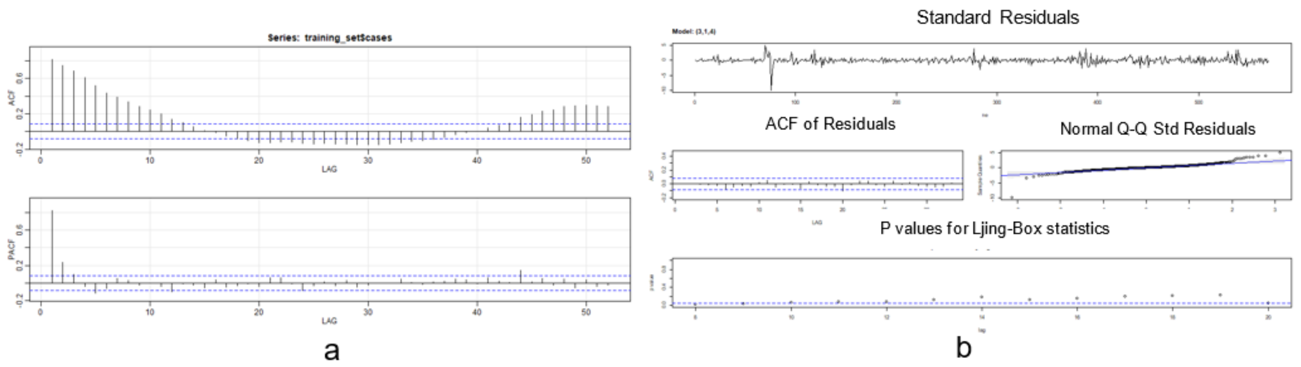

Figure 352: (a) Two plots between lag-time of dengue incidences and ACF and PACF relationship calculated from ARIMA model (b) Summary plots of time series analysis, multiple plots include the plot of predicted model over the time, the plot of ACF residual over lag-time of dengue incidences, residual Q-Q plot of standard residual, and p-value for Ljung-Box statistics of PACF relationship in Trat over the training data starting from January 2001 to December 2013.

The best model of trat province is based on Negative Binomial method. The correlation coefficient on the test set in 2014 yields 0.541 (95%CI: 0.3493, 0.6549). The significant of the variables associated with p-value statistical calculation are shown in Table 121. The best model of Trat consists of 6 variables. The most significant variable are 2-week-lag cases, following by negatively 1-week-lag average pressure and 3-week-lag vaporization. Other variables which has less significant are, current week vaporization and 1-week-lag wind power. Time series methods by ARIMA and SARIMA yield the correlation coefficient of -0.423504 and -20.1188 respectively.

Table 117: Comparison table of all methods by the highest correlation coefficient (R^2) and the lowest prediction error (RMSE) in Trat.

Methods	R-squared (R^2)	Root mean square error (RMSE)
Poisson Regression	0.4952895	2.431702
Negative Binomial Regression	0.5413623	2.318057
Quasi-likelihood Regression	0.4817833	2.464024
ARIMA (3,1,4)	-0.423504	4.083839
SARIMA $(2,0,1)(0,2,0)_{52}$	-20.1188	15.72982

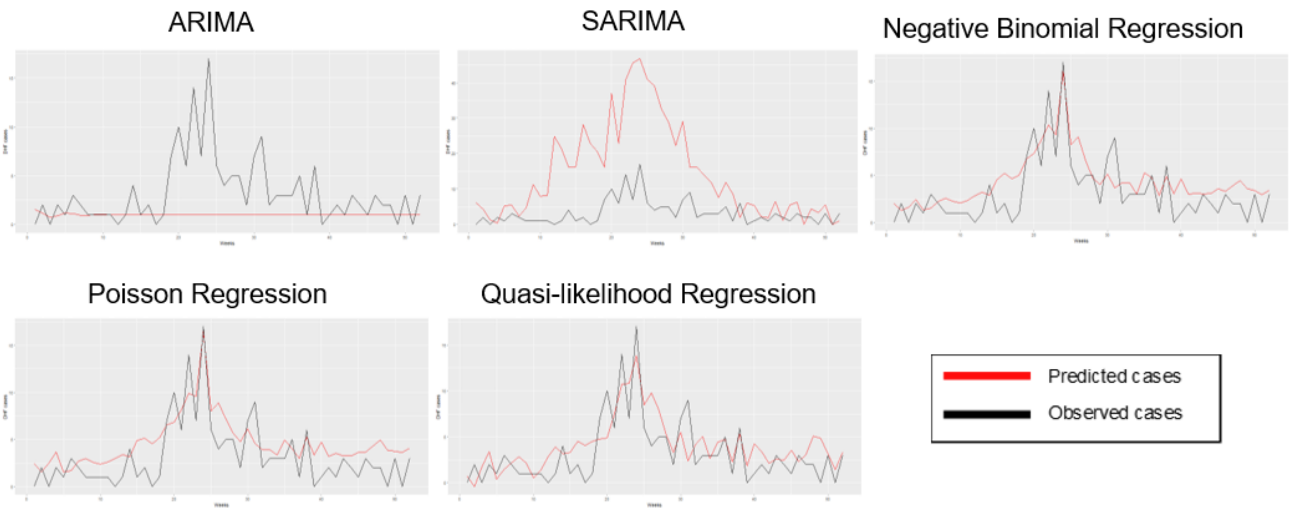

Figure 353: Plots between dengue cases and weeks, the black line represents the observed dengue cases, and the red line represents the predicted dengue cases of the best fit model of each technique over the test set data starting from January 2014 to December 2014.

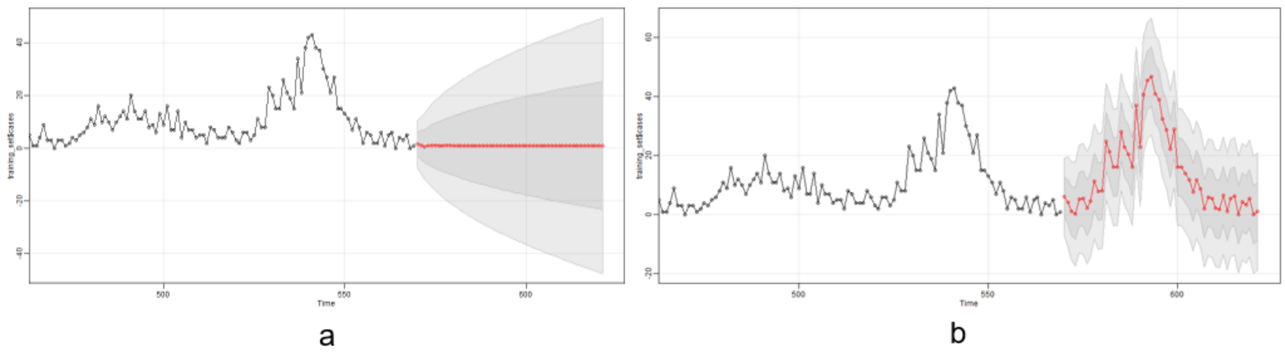

Figure 354: (a) Plot between dengue incidences over weekly time by the best model of ARIMA and (b) SARIMA time series analysis, the black line represents training set data starting from January 2012 to December 2013, and the red line represents the forecasted dengue incidences from January 2014 to December 2014.

Table 118: Coefficients and significant values of best fit GLM models, Negative Binomial, Poisson and Quasi-likelihood regression model of Trat. The table summarizes coefficients of each independent variables which are composed in best fit model of each method. The significant of each variable is labelled by asterisks under the coefficients. The most important factor is marked as three asterisks which p-value ranges from 0 to 0.001. The second important factor is marked as two asterisks which p-value ranges from 0.001 to 0.01. The third important factor is marked as an asterisk which p-value ranges from 0.01 to 0.1. The least important is also marked as a dot which p-value ranges from 0.1 to 1.

**Independent variables**	**Lag**	**Coefficients/Significant**		
		NB	Poisson	Quasi
Intercept		203.08469 ***	206.843111 ***	-38.67574 ***
Cases	1			
	2	0.0600120 ***	0.044607 ***	0.51364 ***
	3			0.23274 ***
Average Pressure	0			
	1	-0.205003 ***	-0.208318 ***	
	2			
	3			
Minimum Temperature	0			
	1			
	2			
	3			
Maximum Temperature	0			
	1			
	2	0.1360570 ***	0.122438 ***	0.86930 ***
	3			
Relative Humidity	0			0.086068 **
	1			
	2			
	3			
Precipitation	0			
	1		-0.003107 **	
	2			
	3			
Vaporization	0	0.052576		
	1			
	2			
	3	0.0930170 *	0.095044 ***	0.77955 **
Wind Direction	0			
	1			
	2			
	3			
Wind Power	0		0.021747 **	0.14966
	1	0.013075	0.030402 ***	0.08135
	2			
	3			

Ubon Ratchathani

Ubon Ratchathani is a province located in the northeastern continent of Thailand at 15°13′41″N 104°51′34″E. Ubon Ratchathani covers an area of 15,745 km^2. Total population are 1,844,669 people. The density of population is 117.0 people per km^2. Weather in Udon Ratchathani has tropical wet and dry climate. The highest temperature present in April around 42.0°C. the lowest temperature mostly presents from of December to March (8.9-10.3°C). The winter are dry and warm. The monsoon season begins from late-April to October. Humidity is around 62-82 percent throughout the year. The highest sunshine hours are in January.

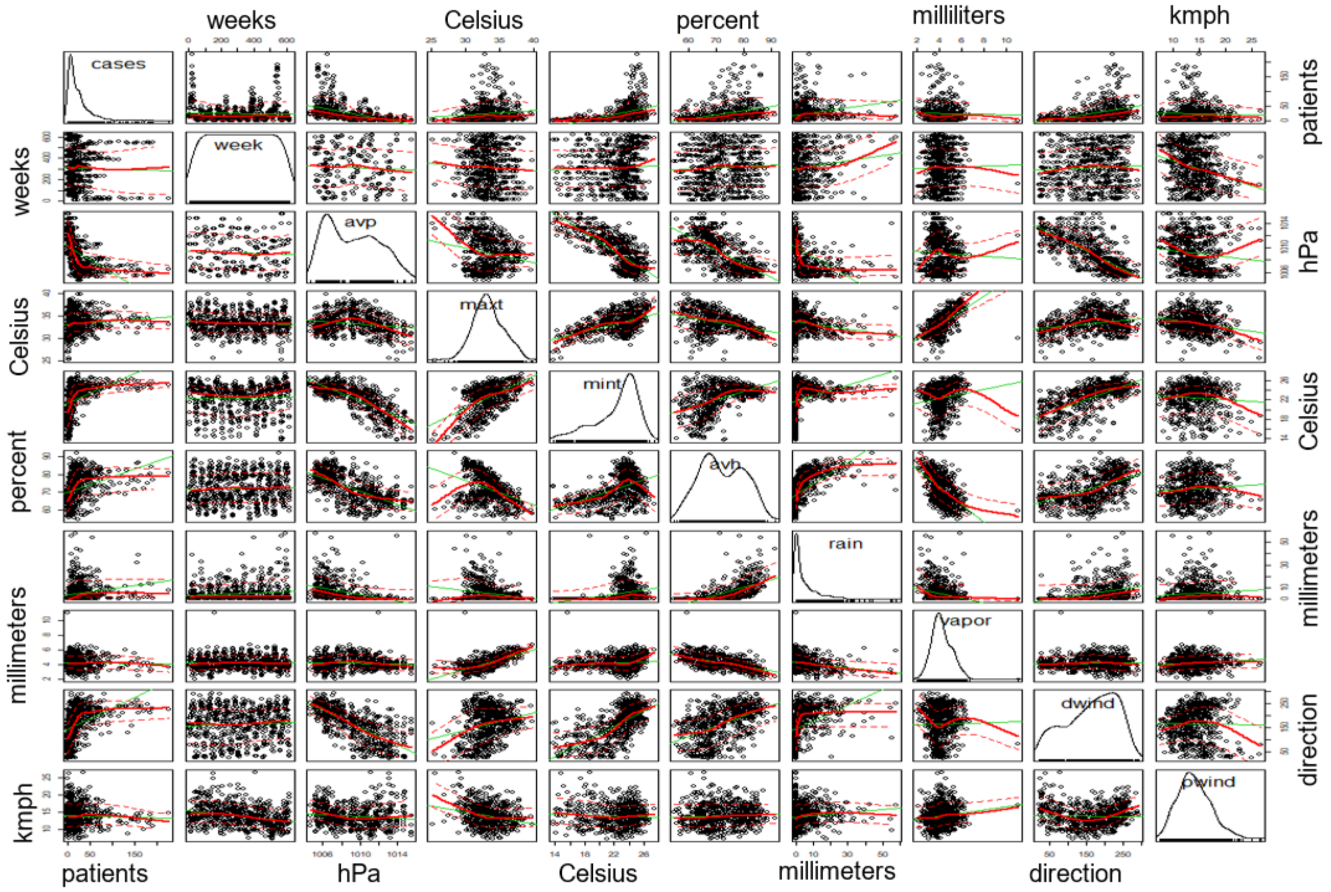

Figure 355: Scatter plot between dengue cases (cases) and selected independent variables, which are the weekly period starting from January 2001 – December 2013 (week), average pressure (avp), maximum temperature (maxt), minimum temperature (mint), average humidity (avh), precipitation (rain), vaporization of water (vapor), wind direction (dwind), and wind power (pwind). The plot visualizes pairwise hundred relationships of training set in Ubon Ratchathani.

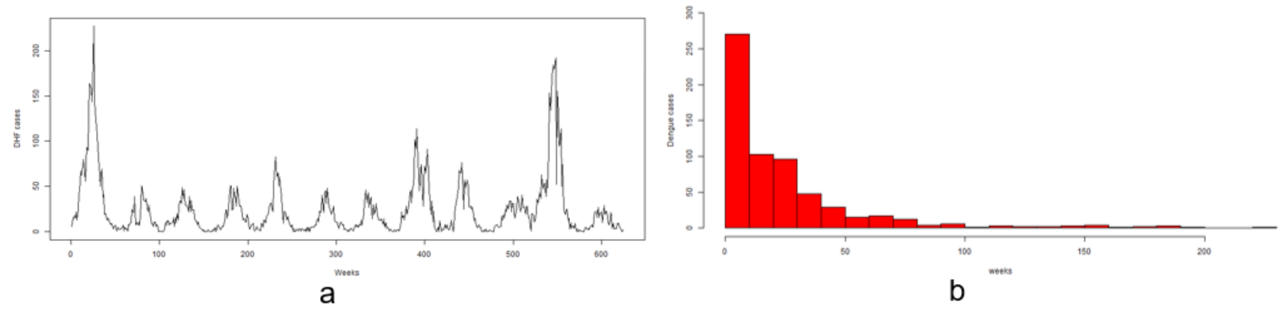

Figure 356: (a) Line plot between dengue incidences and weeks, the plot shows trends of dengue incidences in each year as stationary time series. (b) Histogram of dengue incidences in Ubon Ratchathani starting from January 2001 to December 2013 (624 weeks).

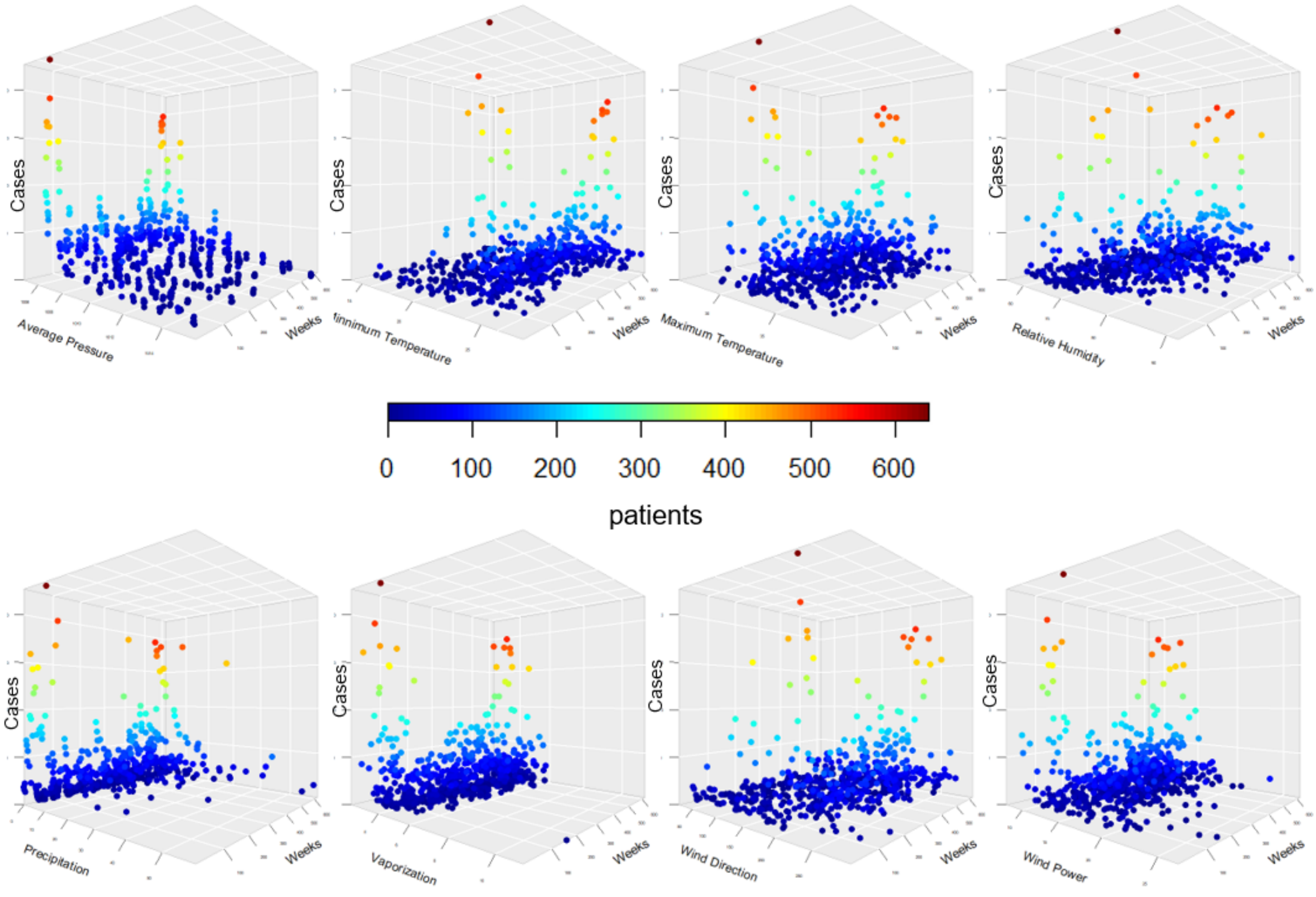

Figure 357: Three-dimensional scatter plot between dengue incidences and weather effects starting from January 2001 to December 2013 of Ubon Ratchathani.

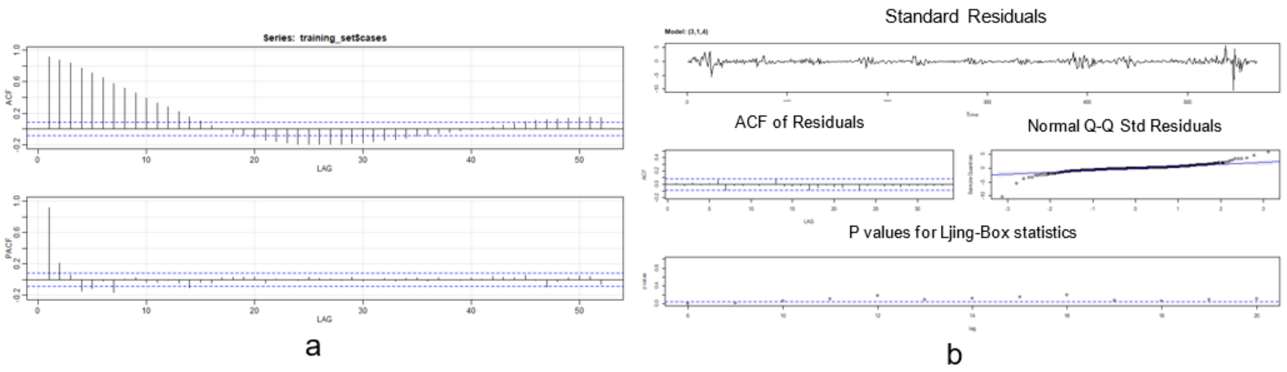

Figure 358: (a) Two plots between lag-time of dengue incidences and ACF and PACF relationship calculated from ARIMA model (b) Summary plots of time series analysis, multiple plots include the plot of predicted model over the time, the plot of ACF residual over lag-time of dengue incidences, residual Q-Q plot of standard residual, and p-value for Ljung-Box statistics of PACF relationship in Ubon Ratchathani over the training data starting from January 2001 to December 2013.

The best model of Ubon Ratchathani is based on quasi-likelihood method. The correlation coefficient on the test set in 2014 is 0.682 (95% CI: 0.6007, 0.7633). The climate factors are sorted by p-value the significant. The best model of Ubon Ratchathani consists of 8 variables, which are 1-week-lag cases, 2-week-lag cases, 3-week-lag vaporization, current week and 3-week-lag relative humidity, 3-week-lag wind direction, 1-week-lag and wind power significantly. Time series methods by ARIMA and SARIMA yield the correlation coefficient of -1.042117 and -69.05007 by fitting the model.

Table 119: Comparison table of all methods by the highest correlation coefficient (R^2) and the lowest prediction error (RMSE) in Ubon Ratchathani.

**Methods**	**R-squared (R^2)**	**Root mean square error (RMSE)**
Poisson Regression	0.3592484	6.805872
Negative Binomial Regression	0.6770023	4.832131
Quasi-likelihood Regression	0.6817921	4.796168
ARIMA (3,1,4)	-1.042117	12.15008
SARIMA $(2,0,1)(0,2,0)_{52}$	-69.05007	71.16119

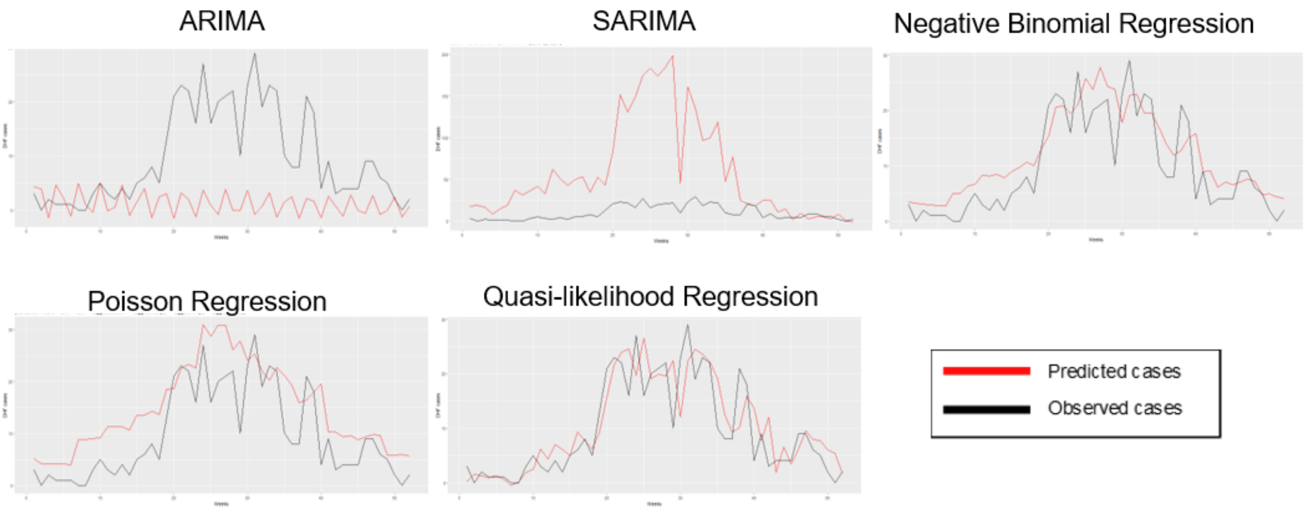

Figure 359: Plots between dengue cases and weeks, the black line represents the observed dengue cases, and the red line represents the predicted dengue cases of the best fit model of each technique over the test set data starting from January 2014 to December 2014.

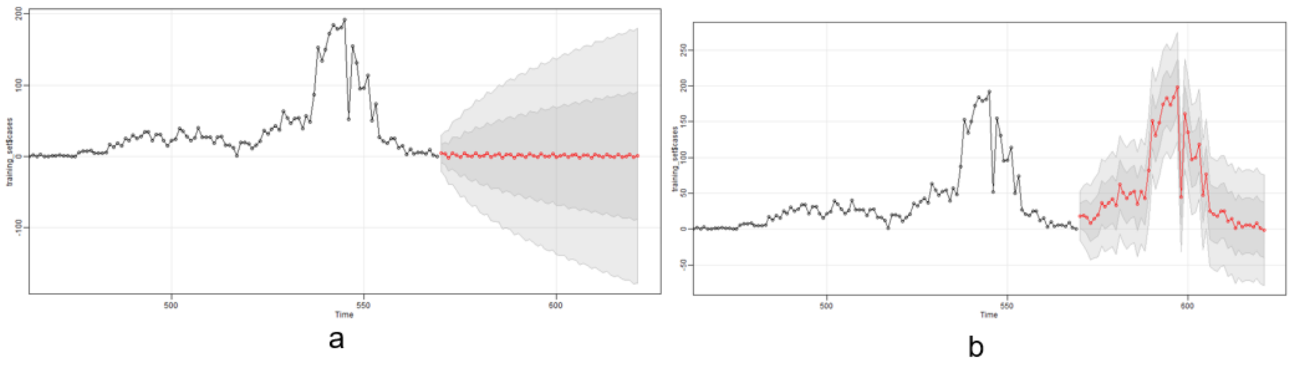

Figure 360: (a) Plot between dengue incidences over weekly time by the best model of ARIMA and (b) SARIMA time series analysis, the black line represents training set data starting from January 2012 to December 2013, and the red line represents the forecasted dengue incidences from January 2014 to December 2014.

Table 120: Coefficients and significant values of best fit GLM models, Negative Binomial, Poisson and Quasi-likelihood regression model of Ubon Ratchathani. The table summarizes coefficients of each independent variables which are composed in best fit model of each method. The significant of each variable is labelled by asterisks under the coefficients. The most important factor is marked as three asterisks which p-value ranges from 0 to 0.001. The second important factor is marked as two asterisks which p-value ranges from 0.001 to 0.01. The third important factor is marked as an asterisk which p-value ranges from 0.01 to 0.1. The least important is also marked as a dot which p-value ranges from 0.1 to 1.

**Independent variables**	**Lag**	**Coefficients/Significant**		
		NB	Poisson	Quasi
Intercept		202.674463 ***	193.9 ***	-7.6077545
Cases	1	0.019702 ***	0.008962 ***	0.7732081 ***
	2		0.002831 ***	0.1675919 ***
	3	0.002537	0.0015556 ***	
Average Pressure	0			
	1			
	2			
	3			
Minimum Temperature	0			
	1	-0.147762 ***	-0.1870 ***	
	2		-0.002117	
	3	-0.049866 .		
Maximum Temperature	0			
	1			
	2			
	3			
Relative Humidity	0	-0.014697 **	-0.007986 ***	-0.1530012
	1			
	2			
	3			0.1361718
Precipitation	0			
	1			
	2		-0.002689 *	
	3			
Vaporization	0			
	1			
	2			
	3			2.5985630 **
Wind Direction	0			
	1			
	2			
	3			-0.0002942
Wind Power	0			
	1			0.0293076
	2			-0.0695990
	3			

Udon Thani

Udon Thani is in the north of northeastern continent of Thailand. Udon Thani locates at 17°25′0″N 102°45′00″E, covers an area of 11,730 km^2. Total population are 1,570,300 people. The density of population is 134.0 people per km^2. Weather in Udon Thani has tropical savanna climate system under the South Asian monsoon. Temperature is in the range from the low of 4.2°C in December to the high of 43.0°C in April. The rainy season begins from around late-April through early-October. The highest rainfall month is August. The highest sunshine hours are in March of 275.9 hours per month. Humidity presents around 60-81 percent throughout the year.

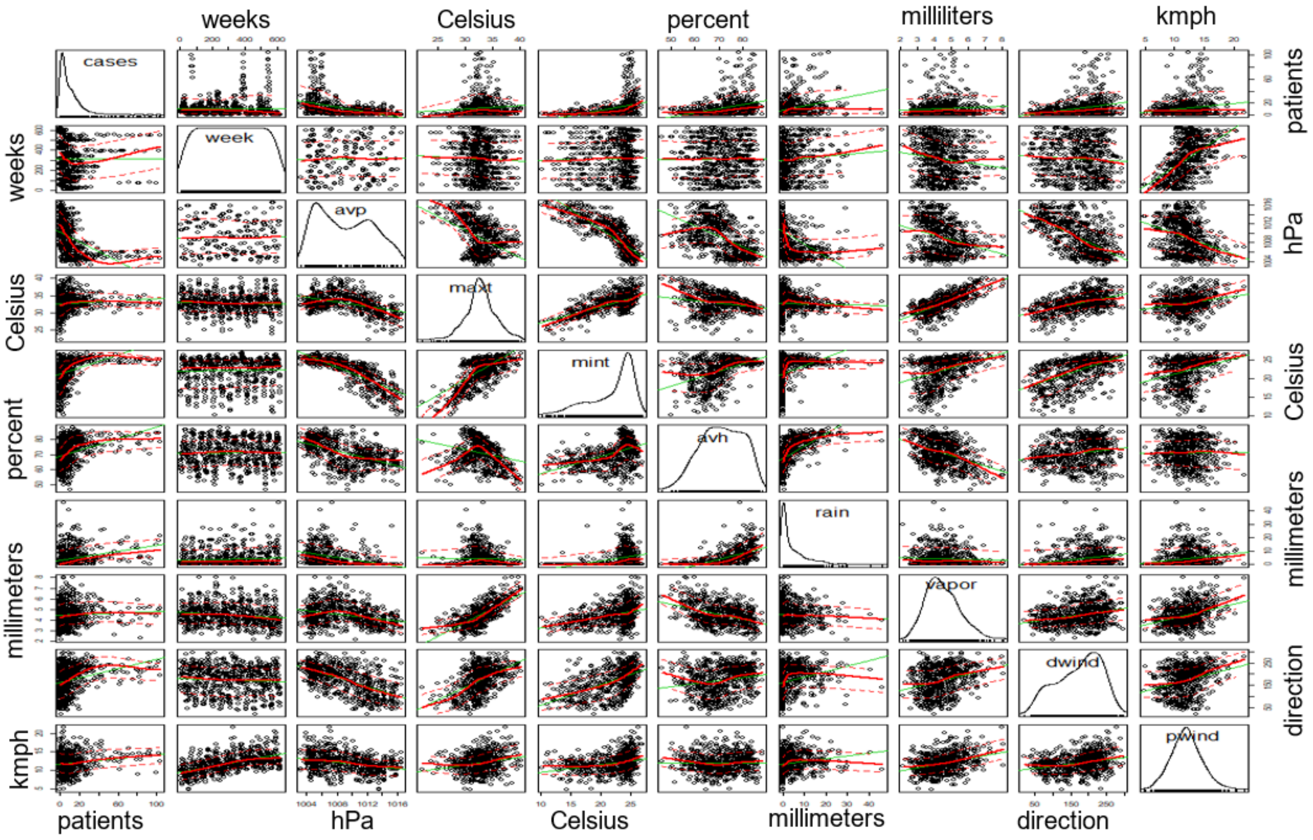

Figure 361: Scatter plot between dengue cases (cases) and selected independent variables, which are the weekly period starting from January 2001 – December 2013 (week), average pressure (avp), maximum temperature (maxt), minimum temperature (mint), average humidity (avh), precipitation (rain), vaporization of water (vapor), wind direction (dwind), and wind power (pwind). The plot visualizes pairwise hundred relationships of training set in Udon Thani.

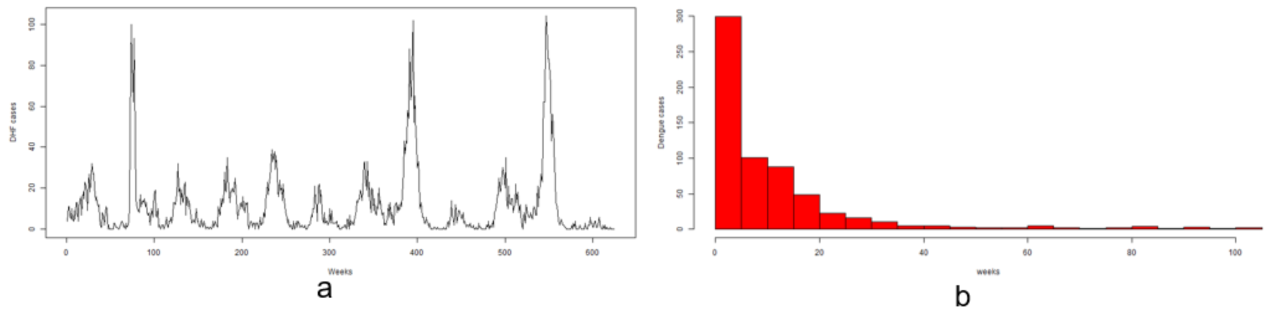

Figure 362: (a) Line plot between dengue incidences and weeks, the plot shows trends of dengue incidences in each year as stationary time series. (b) Histogram of dengue incidences in Udon Thani starting from January 2001 to December 2013 (624 weeks).

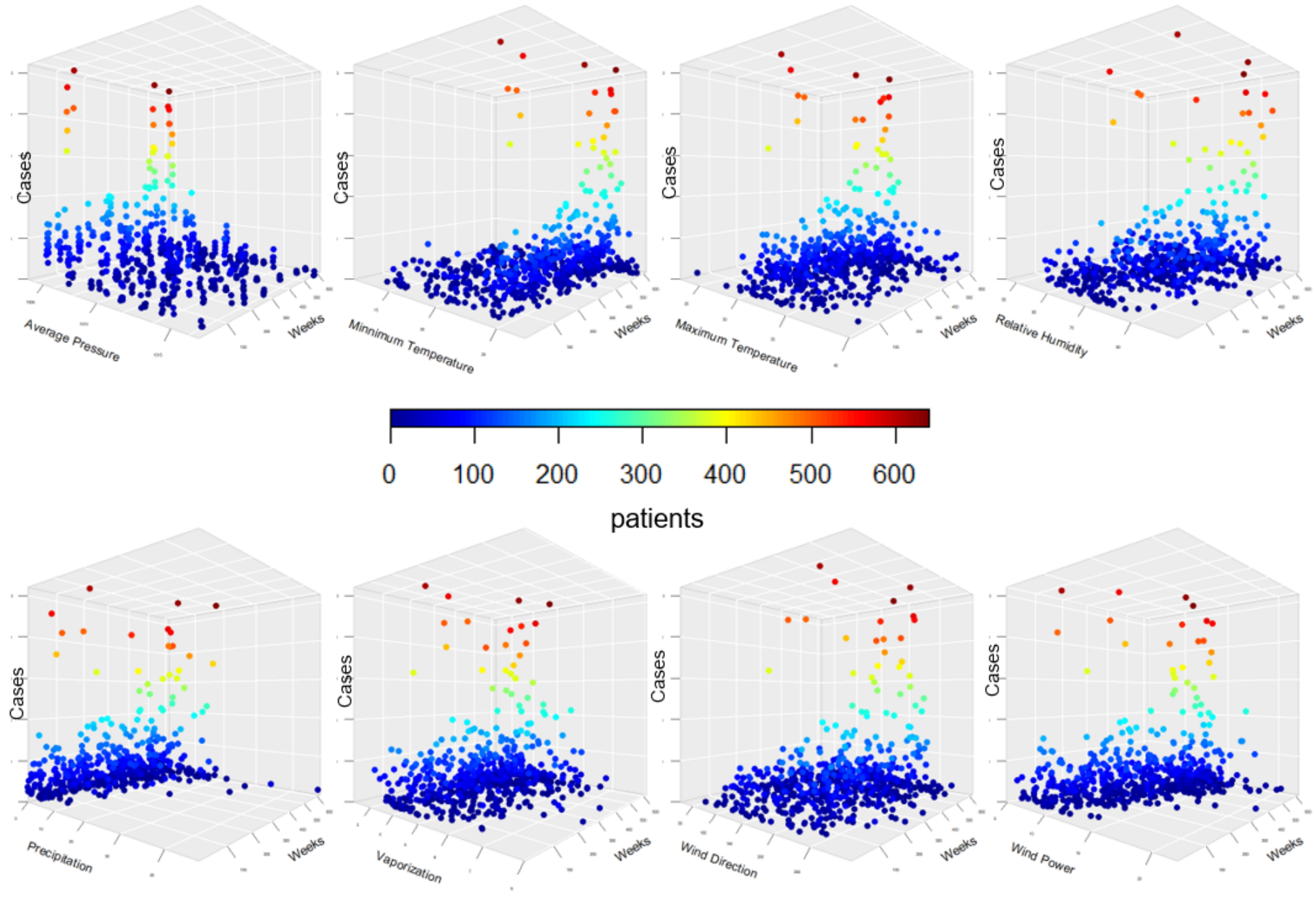

Figure 363: Three-dimensional scatter plot between dengue incidences and weather effects starting from January 2001 to December 2013 of Udon Thani.

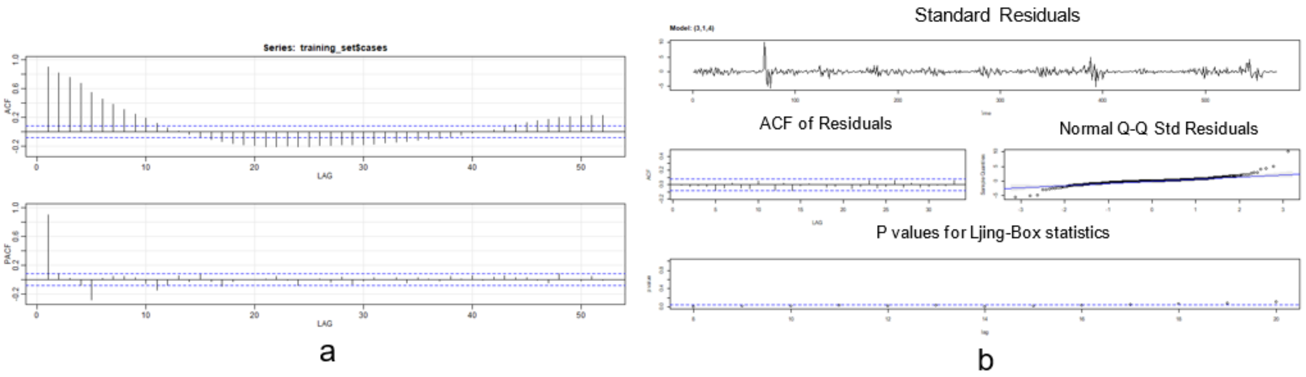

Figure 364: (a) Two plots between lag-time of dengue incidences and ACF and PACF relationship calculated from ARIMA model (b) Summary plots of time series analysis, multiple plots include the plot of predicted model over the time, the plot of ACF residual over lag-time of dengue incidences, residual Q-Q plot of standard residual, and p-value for Ljung-Box statistics of PACF relationship in Udon Thani over the training data starting from January 2001 to December 2013.

For Udon Thani, the best model is based on quasi-likelihood method. The correlation coefficient on the test set in 2014 is 0.257 (95%CI: 0.1185, 0.3955). The best model of Udon Thani uses 7 variables. The most significant variables are 1-week-lag cases, 3-week-lag vaporization and 1-week-lag relative humidity. Other variables which have less significant are, 2-week-lag cases, 3-week-lag cases, negatively current week precipitation and negatively 3-week-lag precipitation. Time series methods by ARIMA and SARIMA yield the correlation coefficient of -1.735292 and -635.8725 respectively.

Table 121: Comparison table of all methods by the highest correlation coefficient (R^2) and the lowest prediction error (RMSE) in Udon Thani.

Methods	R-squared (R^2)	Root mean square error (RMSE)
Poisson Regression	-7.258296	4.48524
Negative Binomial Regression	-3.69476	3.381794
Quasi-likelihood Regression	-0.2573279	1.750108
ARIMA (3,1,4)	-1.735292	2.581323
SARIMA $(2,0,1)(0,2,0)_{52}$	-635.8725	39.38826

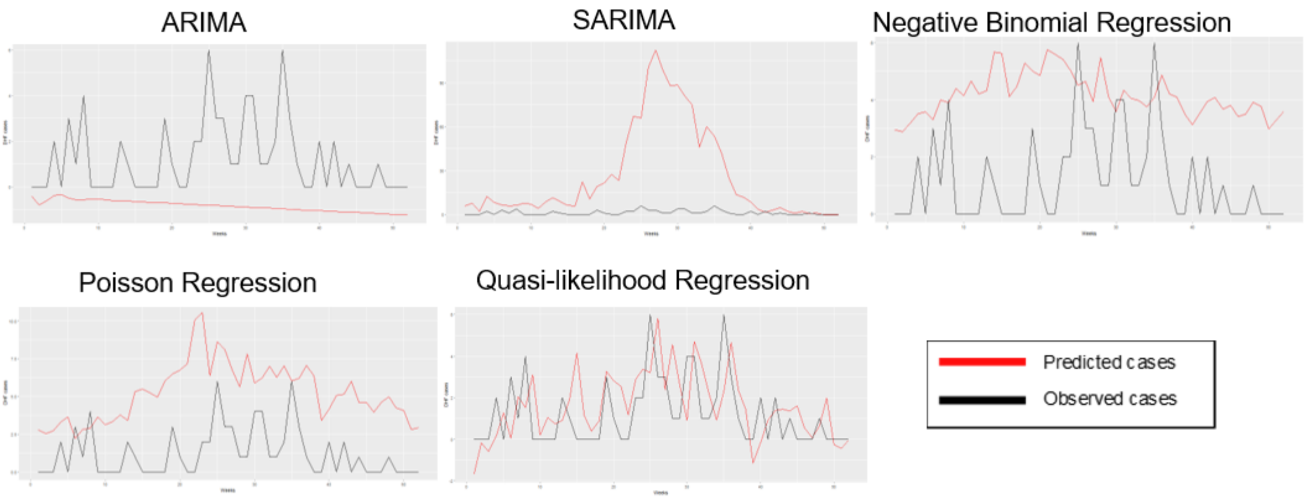

Figure 365: Plots between dengue cases and weeks, the black line represents the observed dengue cases, and the red line represents the predicted dengue cases of the best fit model of each technique over the test set data starting from January 2014 to December 2014.

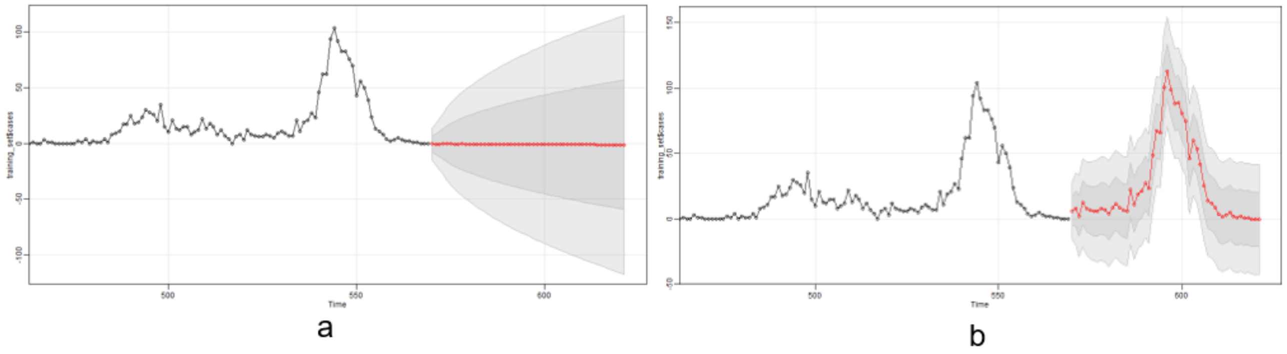

Figure 366: (a) Plot between dengue incidences over weekly time by the best model of ARIMA and (b) SARIMA time series analysis, the black line represents training set data starting from January 2012 to December 2013, and the red line represents the forecasted dengue incidences from January 2014 to December 2014.

Table 122: Coefficients and significant values of best fit GLM models, Negative Binomial, Poisson and Quasi-likelihood regression model of Udon Thani. The table summarizes coefficients of each independent variables which are composed in best fit model of each method. The significant of each variable is labelled by asterisks under the coefficients. The most important factor is marked as three asterisks which p-value ranges from 0 to 0.001. The second important factor is marked as two asterisks which p-value ranges from 0.001 to 0.01. The third important factor is marked as an asterisk which p-value ranges from 0.01 to 0.1. The least important is also marked as a dot which p-value ranges from 0.1 to 1.

**Independent variables**	**Lag**	**Coefficients/Significant**		
		NB	Poisson	Quasi
Intercept		0.544655 **	-3.3669842 ***	-8.34786 *
Cases	1	0.042554 ***	0.0248310 ***	0.81925 ***
	2			0.05974
	3	0.011974 ***	0.0047168 ***	0.02084
Average Pressure	0			
	1			
	2			
	3			
Minimum Temperature	0			
	1			
	2			
	3			
Maximum Temperature	0			
	1			
	2			
	3			
Relative Humidity	0			
	1			0.08004 .
	2			
	3		0.0453241 ***	
Precipitation	0			-0.03960
	1			
	2			
	3		-0.0152280 ***	-0.05528
Vaporization	0			
	1	0.079282 .	0.0477211 ***	
	2	0.127896 **	0.0935767 ***	0.94013 **
	3		0.3018190 ***	
Wind Direction	0			
	1			
	2			
	3			
Wind Power	0			
	1	-0.003444		
	2			
	3			

Uttaradit

Uttaradit is located in the northern continent of Thailand at 13°45′N 100°28′E. Uttaradit covers an area of 7,839 km^2. Total population are 460,400 people. The density of population is 59.0 per km^2. Weather in Uttaradit has tropical savanna climate under the South Asian monsoon system. The highest temperature is around 44.5°C in summer (April). The lowest temperature in range from 8.2-10.4°C in December to January. The monsoon season begins with the arrival of the southwest monsoon around mid-May through October. The highest rainfall is in August approximately 263.4 *mm*. In winter, the air is dry and warm. Humidity is in the range of 62-83 throughout the year. The highest sunshine hours present March.

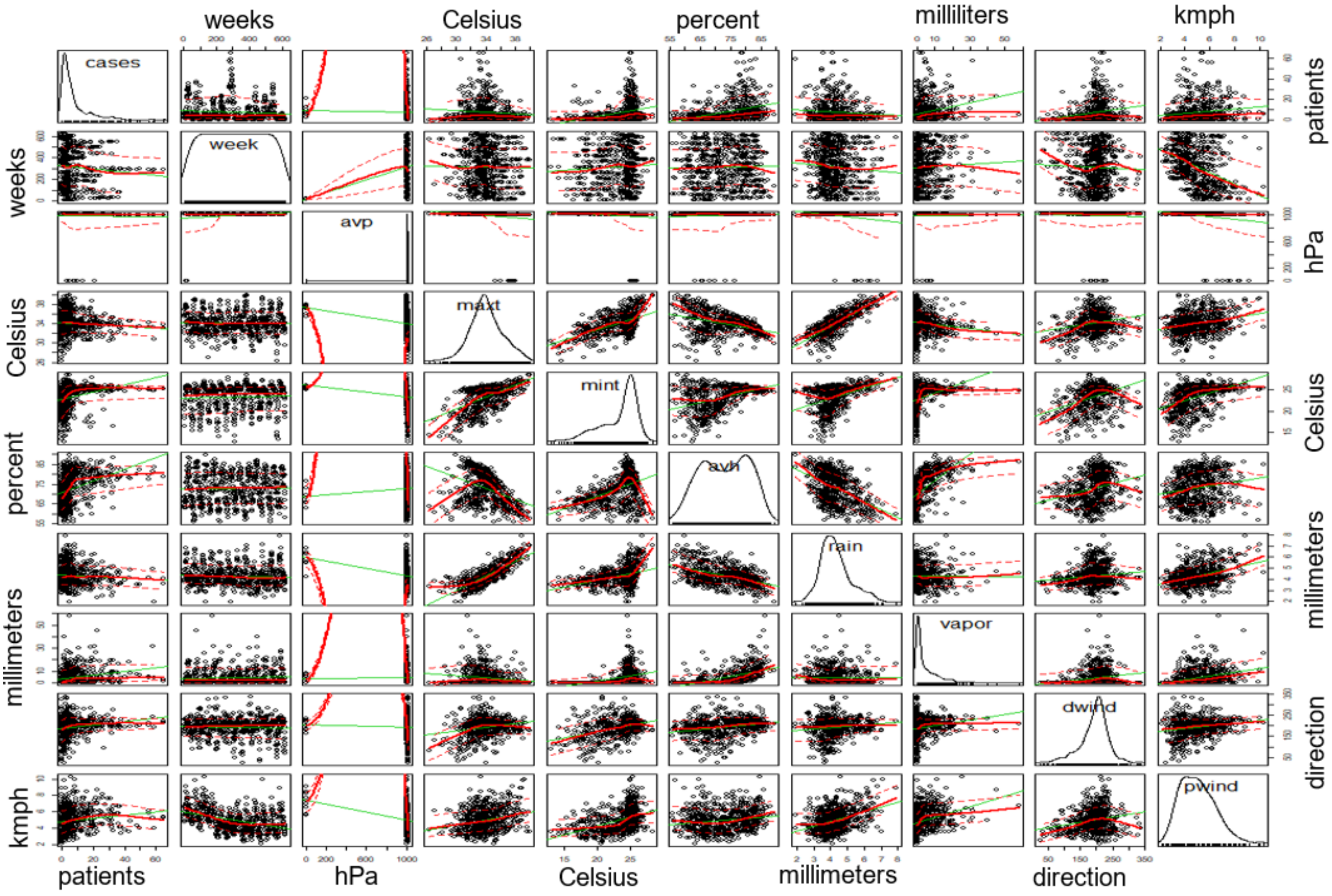

Figure 367: Scatter plot between dengue cases (cases) and selected independent variables, which are the weekly period starting from January 2001 – December 2013 (week), average pressure (avp), maximum temperature (maxt), minimum temperature (mint), average humidity (avh), precipitation (rain), vaporization of water (vapor), wind direction (dwind), and wind power (pwind). The plot visualizes pairwise hundred relationships of training set in Uttaradit.

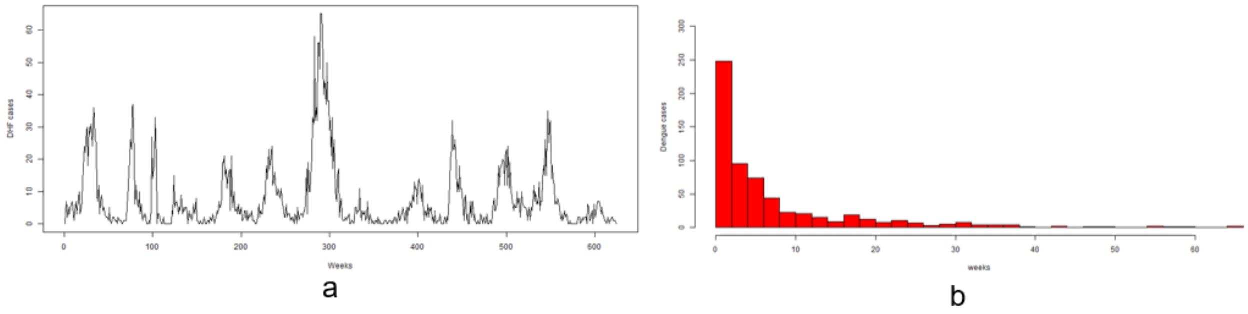

Figure 368: (a) Line plot between dengue incidences and weeks, the plot shows trends of dengue incidences in each year as stationary time series. (b) Histogram of dengue incidences in Uttaradit starting from January 2001 to December 2013 (624 weeks).

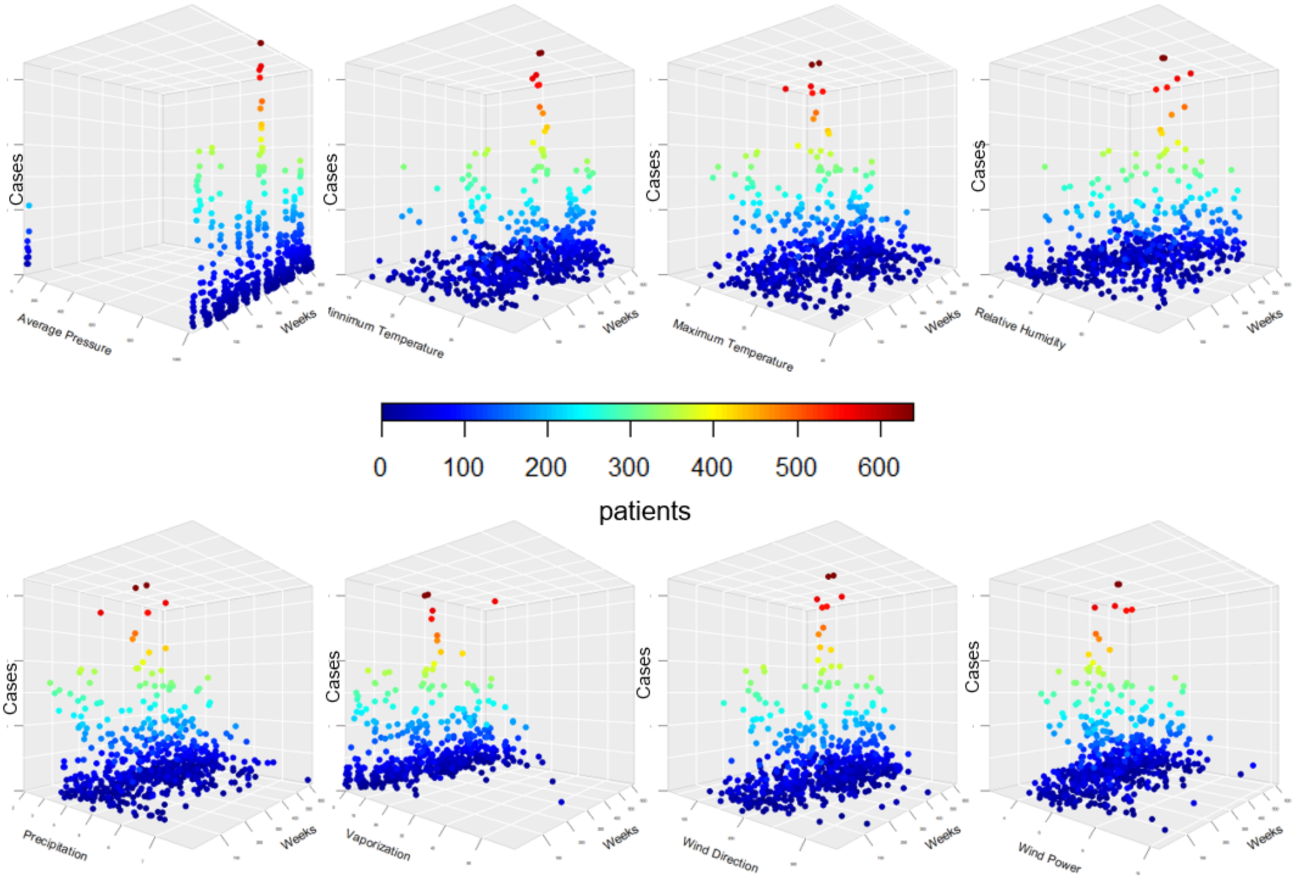

Figure 369: Three-dimensional scatter plot between dengue incidences and weather effects starting from January 2001 to December 2013 of Uttaradit.

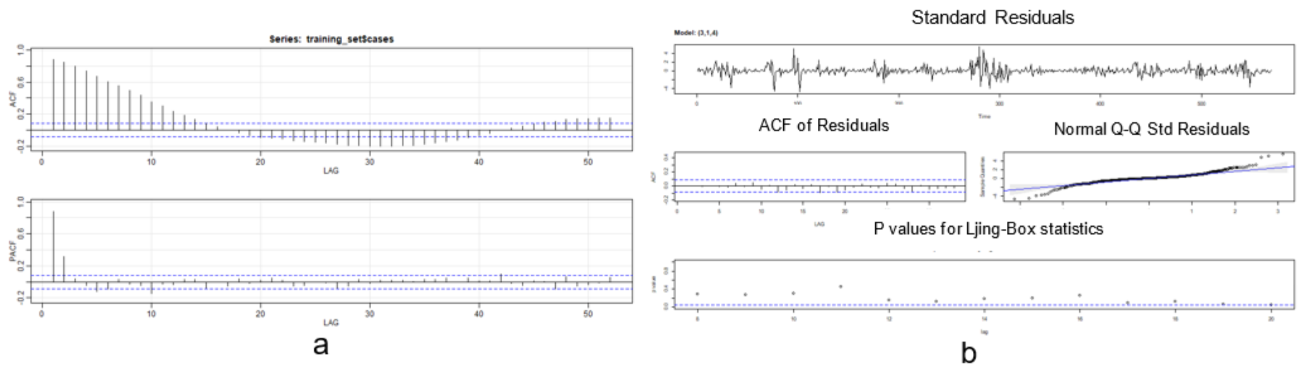

Figure 370: (a) Two plots between lag-time of dengue incidences and ACF and PACF relationship calculated from ARIMA model (b) Summary plots of time series analysis, multiple plots include the plot of predicted model over the time, the plot of ACF residual over lag-time of dengue incidences, residual Q-Q plot of standard residual, and p-value for Ljung-Box statistics of PACF relationship in Uttaradit over the training data starting from January 2001 to December 2013.

The best model of Uttaradit is based on quasi-likelihood method. The model consists of 9 variables which 1-week-lag cases and 3-week-lag cases variables are the most significant. These two variables influence the correlation coefficient on the test set of 0.454 (95%CI: 0.3423, 0.5657). Other variables which have less significant are, average pressure, 3-week-lag relative humidity, 1-week-lag vaporization, 3-week-lag wind direction, 1-week-lag wind power and 3-week-lag wind power. In time series methods by ARIMA and SARIMA yield the correlation coefficient of -0.6136434 and -21.96161 respectively.

Table 123: Comparison table of all methods by the highest correlation coefficient (R^2) and the lowest prediction error (RMSE) in Uttaradit.

**Methods**	**R-squared (R^2)**	**Root mean square error (RMSE)**
Poisson Regression	-0.5070841	2.558865
Negative Binomial Regression	0.2114817	1.850907
Quasi-likelihood Regression	0.4345301	1.567413
ARIMA (3,1,4)	-0.6136434	2.647783
SARIMA $(2,0,1)(0,2,0)_{52}$	-21.96161	9.988029

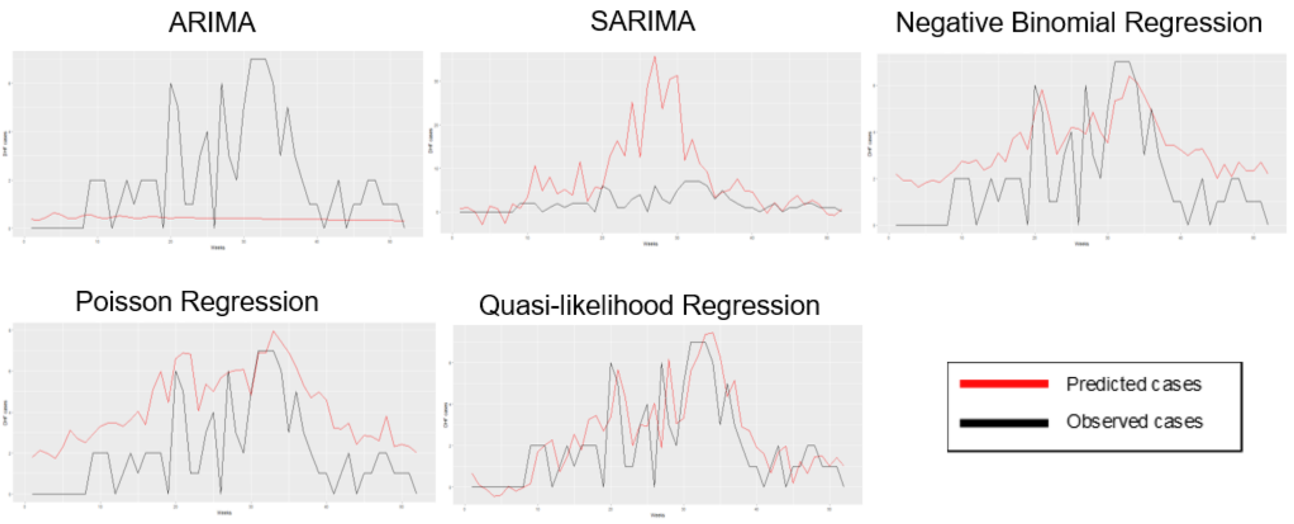

Figure 371: Plots between dengue cases and weeks, the black line represents the observed dengue cases, and the red line represents the predicted dengue cases of the best fit model of each technique over the test set data starting from January 2014 to December 2014.

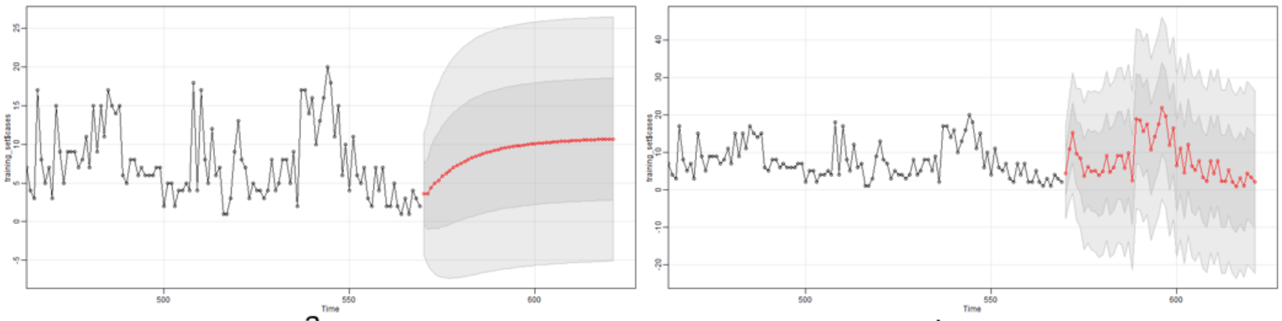

a b

Figure 372: (a) Plot between dengue incidences over weekly time by the best model of ARIMA and (b) SARIMA time series analysis, the black line represents training set data starting from January 2012 to December 2013, and the red line represents the forecasted dengue incidences from January 2014 to December 2014.

Table 124: Coefficients and significant values of best fit GLM models, Negative Binomial, Poisson and Quasi-likelihood regression model of Uttaradit. The table summarizes coefficients of each independent variables which are composed in best fit model of each method. The significant of each variable is labelled by asterisks under the coefficients. The most important factor is marked as three asterisks which p-value ranges from 0 to 0.001. The second important factor is marked as two asterisks which p-value ranges from 0.001 to 0.01. The third important factor is marked as an asterisk which p-value ranges from 0.01 to 0.1. The least important is also marked as a dot which p-value ranges from 0.1 to 1.

**Independent variables**	**Lag**	**Coefficients/Significant**		
		NB	Poisson	Quasi
Intercept		0.0209795	0.1095	0.0373811
Cases	1	0.0622195<br>***	0.03159<br>***	0.7143937<br>***
	2		0.01757<br>***	
	3	0.0185506<br>***	0.004872<br>*	0.1940417<br>***
Average Pressure	0			-0.0005346
	1			
	2			
	3		-0.0003649<br>***	
Minimum Temperature	0			
	1			
	2			
	3			
Maximum Temperature	0			
	1			
	2			
	3			
Relative Humidity	0			
	1			
	2			
	3			-0.0252717
Precipitation	0			
	1			
	2			
	3			
Vaporization	0			
	1			-0.0326844
	2			
	3			
Wind Direction	0		0.003328<br>***	
	1			
	2			
	3	0.0020936<br>*	0.001973<br>***	0.0022153
Wind Power	0			
	1			0.1434927
	2			
	3	0.1180754<br>***	0.1111<br>***	0.4114756

Yala

Yala is a province located in southern region of Thailand at 6°32′33″N 101°16′59″E. Yala covers an area of 4,521.1 km^2. Total population are 511,911 people. The density of population is 110 per km^2. Weather in Yala behaves tropical savanna climate under the South Asian monsoon system. The highest temperature often occurs in April from 40.0°C. Low temperature presents from December-February in the range of 16.0 to 17.4 °C. The rainy season begins with the arrival of the southwest monsoon around mid-May. In Yala, June is the wettest month, the average rainfall presents around 106.0 mm.

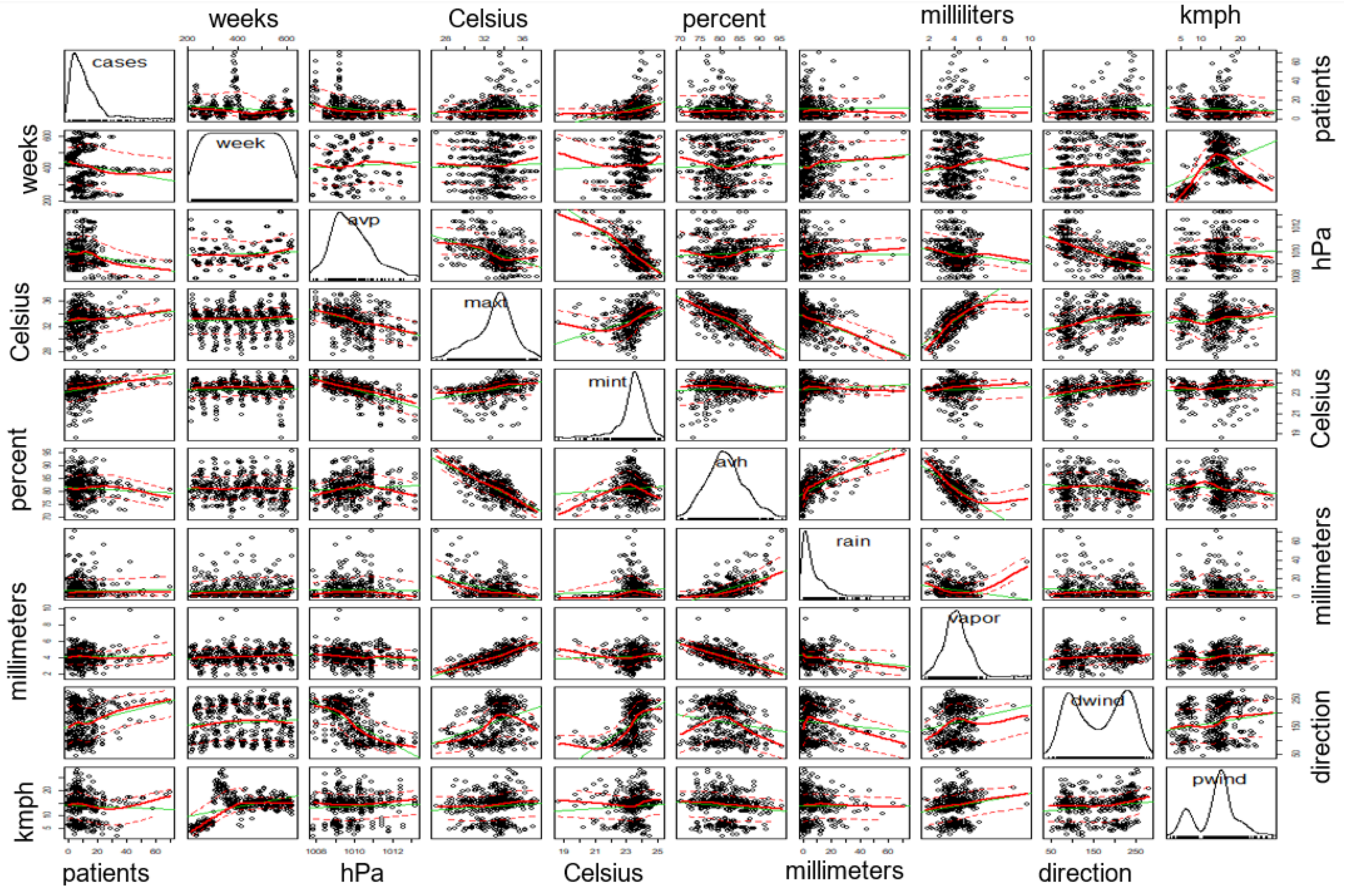

Figure 373: Scatter plot between dengue cases (cases) and selected independent variables, which are the weekly period starting from January 2001 – December 2013 (week), average pressure (avp), maximum temperature (maxt), minimum temperature (mint), average humidity (avh), precipitation (rain), vaporization of water (vapor), wind direction (dwind), and wind power (pwind). The plot visualizes pairwise hundred relationships of training set in Yala.

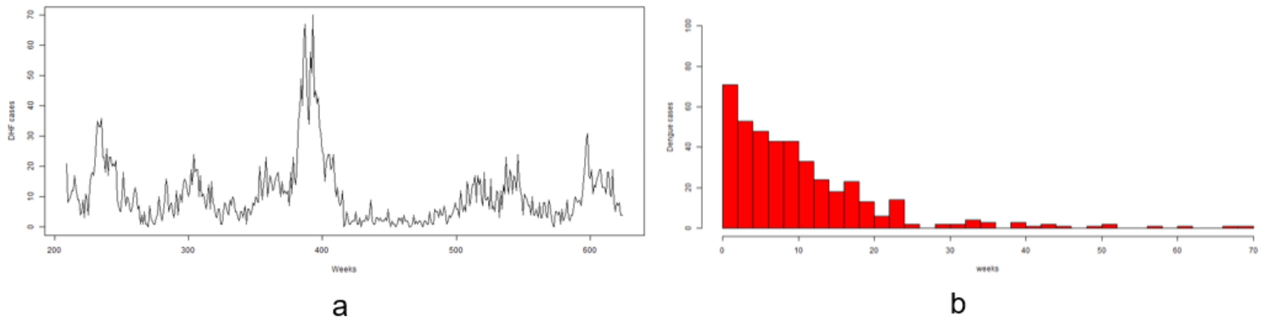

Figure 374: (a) Line plot between dengue incidences and weeks, the plot shows trends of dengue incidences in each year as stationary time series. (b) Histogram of dengue incidences in Yala starting from January 2001 to December 2013 (624 weeks).

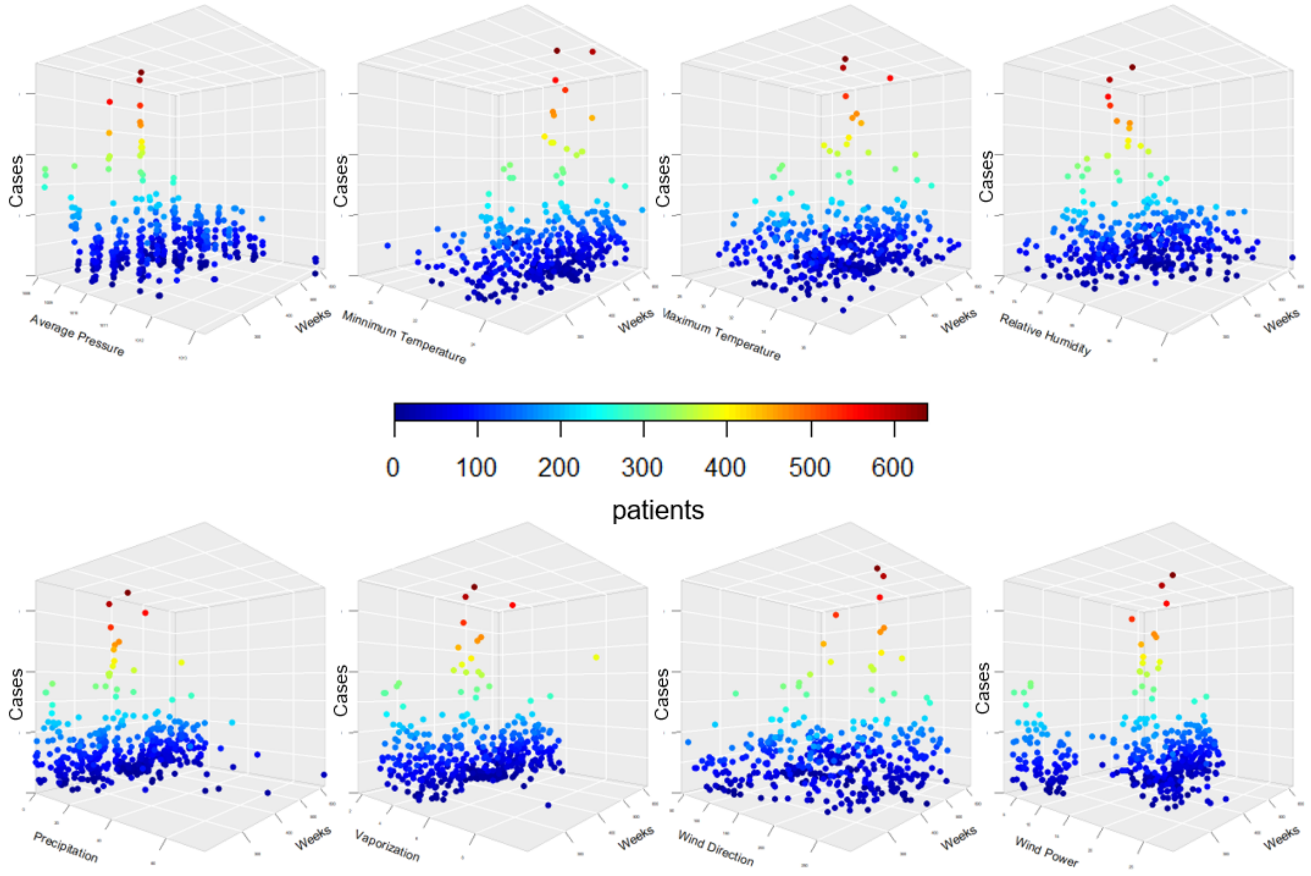

Figure 375: Three-dimensional scatter plot between dengue incidences and weather effects starting from January 2001 to December 2013 of Yala.

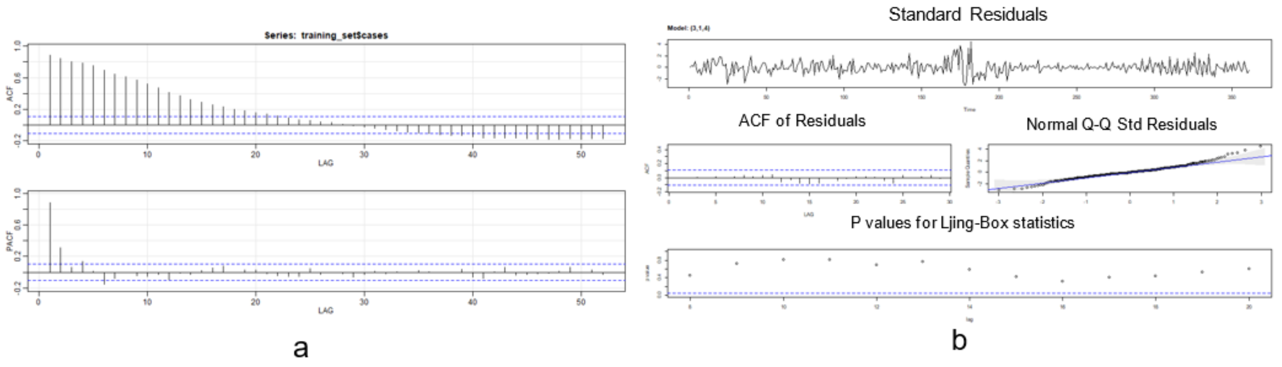

Figure 376: (a) Two plots between lag-time of dengue incidences and ACF and PACF relationship calculated from ARIMA model (b) Summary plots of time series analysis, multiple plots include the plot of predicted model over the time, the plot of ACF residual over lag-time of dengue incidences, residual Q-Q plot of standard residual, and p-value for Ljung-Box statistics of PACF relationship in Yala over the training data starting from January 2001 to December 2013.

The best model of Yala is based on quasi-likelihood method. The correlation coefficient on the test set in 2014 is 0.6308 (95%CI: 0.5405, 0.7214). The best model of Yala uses 6 variables. The significant of the variables associated with p-value statistical calculation are 1-week-lag cases and 3-week-lag minimum temperature. Other variables which have less significant are, 2-week-lag average pressure, 1-week-lag minimum temperature, 3-week-lag minimum temperature and 3-week-lag precipitation. Time series methods by ARIMA and SARIMA yield the correlation coeffect of -1.669 and -0.949 which are unpredictable.

Table 125: Comparison table of all methods by the highest correlation coefficient (R^2) and the lowest prediction error (RMSE) in Yala.

**Methods**	**R-squared (R^2)**	**Root mean square error (RMSE)**
Poisson Regression	0.6234138	4.166418
Negative Binomial Regression	0.5527849	4.540345
Quasi-likelihood Regression	0.6308746	4.124939
ARIMA (3,1,4)	-1.66979	11.09351
SARIMA $(2,0,1)(0,2,0)_{52}$	-0.949653	9.48002

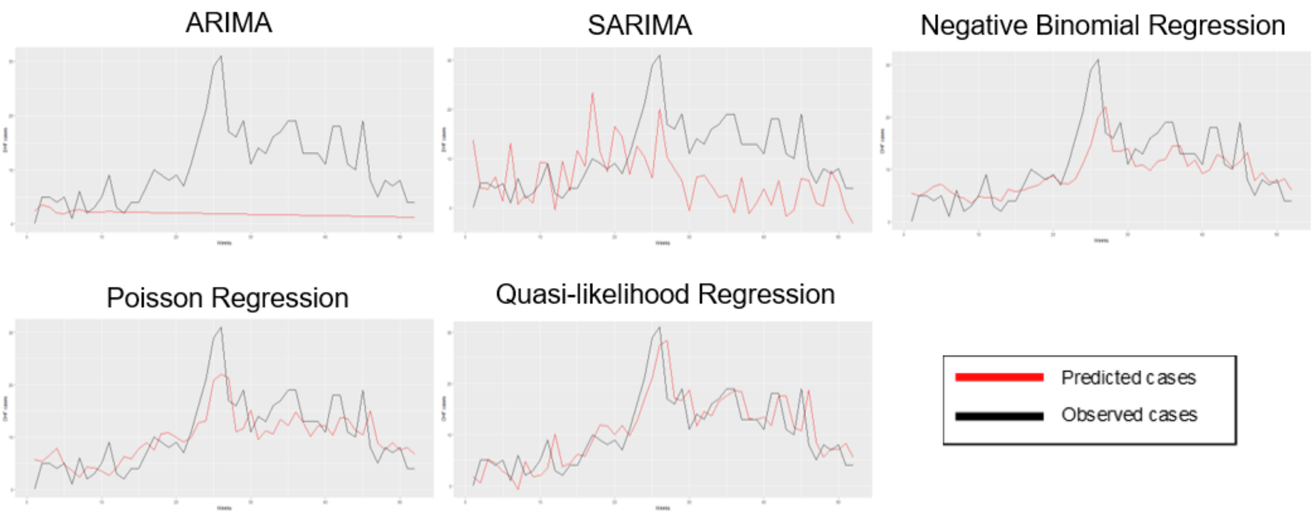

Figure 377: Plots between dengue cases and weeks, the black line represents the observed dengue cases, and the red line represents the predicted dengue cases of the best fit model of each technique over the test set data starting from January 2014 to December 2014.

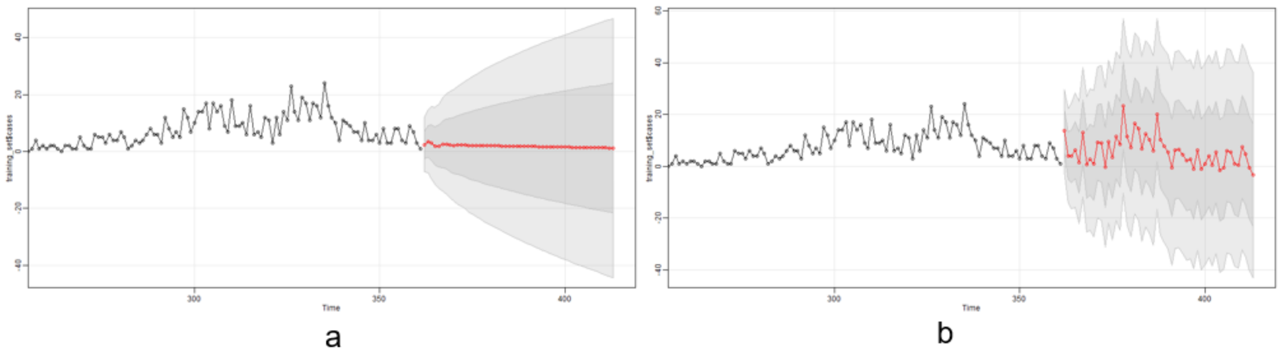

Figure 378: (a) Plot between dengue incidences over weekly time by the best model of ARIMA and (b) SARIMA time series analysis, the black line represents training set data starting from January 2012 to December 2013, and the red line represents the forecasted dengue incidences from January 2014 to December 2014.

Table 126: Coefficients and significant values of best fit GLM models, Negative Binomial, Poisson and Quasi-likelihood regression model of Yala. The table summarizes coefficients of each independent variables which are composed in best fit model of each method. The significant of each variable is labelled by asterisks under the coefficients. The most important factor is marked as three asterisks which p-value ranges from 0 to 0.001. The second important factor is marked as two asterisks which p-value ranges from 0.001 to 0.01. The third important factor is marked as an asterisk which p-value ranges from 0.01 to 0.1. The least important is also marked as a dot which p-value ranges from 0.1 to 1.

**Independent variables**	**Lag**	**Coefficients/Significant**		
		NB	Poisson	Quasi
Intercept		-127.9<br>**	-196.6<br>***	-628.15097
Cases	1	0.04147<br>***	0.04104<br>***	0.8621600<br>***
	2			
	3	0.01981<br>***		
Average Pressure	0	-0.01562		
	1			
	2			0.58512
	3	0.01396<br>**	0.1880<br>***	
Minimum Temperature	0			
	1		0.1012<br>**	0.82305
	2		0.2168<br>***	
	3	0.1307<br>*	0.02624	0.23118
Maximum Temperature	0		0.007928	
	1	0.04340		
	2			
	3			0.4230400<br>*
Relative Humidity	0	0.001120		
	1			
	2			
	3			
Precipitation	0			
	1			
	2			
	3		0.03472<br>.	0.01215
Vaporization	0			
	1	-0.1044<br>*		
	2			
	3			
Wind Direction	0			
	1			
	2			
	3		0.0007351<br>*	
Wind Power	0			
	1			
	2			
	3			
